# Supplementary material for: Organocatalytic atroposelective construction of axially chiral N, N- and N, S-1,2-azoles through novel ring formation approach
Source: Nat Commun. 2022 Apr 11;13:1933. doi: 10.1038/s41467-022-29557-1 (PMC9001698; doi:10.1038/s41467-022-29557-1)
Supplement: Supplementary file 1 — Supplementary Information [file 41467_2022_29557_MOESM1_ESM.pdf]

Supplementary Information for

Organocatalytic atroposelective construction of axially chiral N, N- and N, S-1,2-azoles through novel ring formation approach

Yu Chang[1,2], Chuandong Xie[1,2], Hong Liu[1], Shengli Huang[1], Pengfei Wang[1], Wenling Qin[1]* and Hailong Yan[1]*

[1]*Chongqing Key Laboratory of Natural Product Synthesis and Drug Research, School of Pharmaceutical Sciences, Chongqing University, Chongqing 401331, P. R. China.*

[2]These authors contributed equally: Yu Chang, Chuandong Xie.

Corresponding author e-mail: wenling.qin@cqu.edu.cn

yhl198151@cqu.edu.cn

Table of Contents

I. General information 2

II. General procedure for the synthesis of substrate 3

III. Optimization of the reaction conditions 13

IV. General procedure for the asymmetric reaction 17

V. Racemization studies 65

VI. Control experiments and plausible catalytic cycle 69

VII. ^{1}H and ^{13}C NMR spectra 74

VIII. X-ray crystallographic information 187

IX. COX-2 inhibitory assay 191

X. Cell culture 191

XI. Anti-proliferation assay 191

XII. Cell apoptosis analysis 192

XIII. Cell cycle analysis 192

XIV. ROS measurement 193

XV. Mitochondrial membrane potential evaluation 193

XVI. Western blot analysis 194

XVII. Druglikeness and activity cliff 194

XVIII. Supplementary References 196

I. General information

^{1}H and ^{13}C NMR spectra were recorded on Agilent 400MR DD2 (400 MHz) spectrometer. Chemical shifts were reported in parts per million (ppm), and tetramethylsilane or the residual solvent peak was used as an internal reference: ^{1}H (tetramethylsilane δ 0.00 ppm, acetone δ 2.05 ppm, Dimethyl sulfoxide δ 2.50 ppm), ^{13}C (chloroform δ 77.00 ppm, acetone δ 29.70 ppm, Dimethyl sulfoxide δ 39.52 ppm). All deuterated reagents were purchased from Adamas-beta®. Data are reported as follows: chemical shift, multiplicity (s = singlet, d = doublet, t = triplet, q = quartet, m = multiplet, br = broad), coupling constants (Hz) and integration. Enantiomeric excesses (ee) were determined by HPLC analysis on Hitachi Chromaster using DAICEL CHIRALCEL AD-H, 4.6 mm *Φ* × 250 mmL, DAICEL CHIRALCEL OD-H, 4.6 mm *Φ* × 250 mmL, DAICEL CHIRALCEL OJ-H, 4.6 mm *Φ* × 250 mmL, DAICEL CHIRALCEL IA-H, 4.6 mm *Φ* × 250 mmL, DAICEL CHIRALCEL IB-H, 4.6 mm *Φ* × 250 mmL and DAICEL CHIRALCEL IC-H, 4.6 mm *Φ* × 250 mmL. High resolution mass spectra (HRMS) were performed on Bruker Solarix 7.0 T. X-ray crystallography analysis of single crystal was performed on an Agilent SuperNova-CCD X-Ray diffractometer. Optical rotations were measured on a Rudolph Autopol I polarimeter and are reported as follows: $[\alpha]_D^{20}$ (*c* in g per 100 mL solvent). Unless otherwise stated, all reagents were purchased from commercial suppliers and used without further purification. (PE: Petroleum ether; EA: Ethyl acetate). The instruments, reagents and materials used in the biological evaluation experiment can be found in **IX-XVII** of contents.

II. General procedure for the synthesis of substrate

General procedure for the synthesis of 1a-1u, 1x

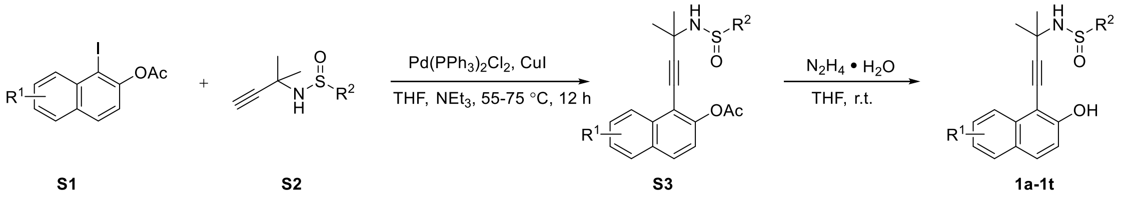

Starting materials **S1** and **S2** were prepared according to the known literatures.[1],[2],[3],[4]

Step1：S2 (1.5 equiv) was dissolved in THF (3.0 mmol/mL) and dropwise added to a solution of the **S1** (1.0 equiv), $Pd(PPh_3)_2Cl_2$ (0.02 equiv), CuI (0.04 equiv), and NEt_3 (5.0 equiv) in THF (0.45 mmol/mL) at 55-75 ºC under N_2 atmosphere. The mixture was stirred for 12 h. Then the mixture was filtered through a pad of celite. Removal of the solvent under reduced pressure. The crude product was purified by column chromatography on silica gel (PE: EA = 5:1) to afford **S3**.

Step2：S3 (1.0 equiv) was dissolved in THF (0.02 mmol/mL) and hydrazinium hydroxide solution (3.0 equiv) was dropwise added in the mixture. The mixture stirred 15 min in room temperature. After the reaction was completed by TLC monitored. The reaction was quenched by pouring into cooled saturated NH_4Cl aqueous and the mixture was extracted with EA. The organic phases were combined, washed with brine, dried over Na_2SO_4, and concentrated to afford the crude product. The purification of the crude mixture by flash column chromatography (PE/EA = 3:1-8:1) provided **1a-1u, 1x**.

(±)**-1v** was prepared according to the literature.[5] **1w** was prepared according to the literature.[2]

General procedure for the synthesis of 3a-3p

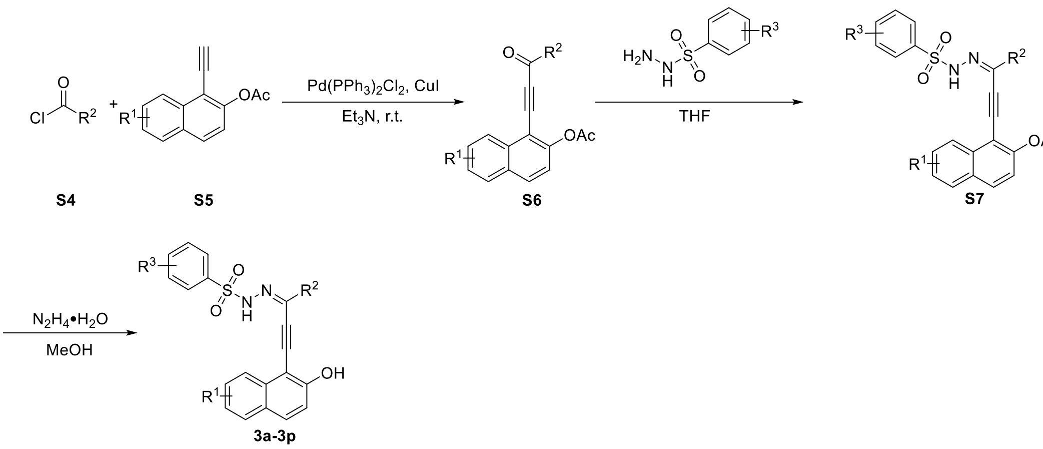

Step1： The preparation of **S3** was followed the literature procedure.[6] A mixture of the corresponding acyl chloride **S4** (1.2 equiv), $Pd(PPh_3)_2Cl_2$ (0.02 equiv) and NEt_3 (1.2 equiv) in anhydrous THF were stirred for 10 min at 25 °C under N_2 atmosphere. CuI (0.04 equiv) was then added and the reaction mixture was stirred for another 10 min. Terminal alkyne **S5** (1.0 equiv) was then added in one portion, the resulting mixture was stirred at 25 °C for 5 h. After the reaction was complete, EA was added, and the resulting solution was washed with 0.1N HCl in a separatory funnel. After the layers were separated, the organic phase was dried over Na_2SO_4 and evaporated on a rotary evaporator to give the crude product, which was purified by flash chromatography on silica gel using PE/EA as the eluent to afford the corresponding *α, β*-alkynic ketones **S6**.

Step2： This step was carried out according to some literature methods[7],[8],[9] with some modification. To a solution of **S6** (1.0 equiv) and benzenesulfonyl hydrazide in a mixture of THF (1.0 mmol/mL) and stirred for 48 h. The reaction mixture was extracted with EA and the combined organic layer was washed with H_2O and brine, dried over Na_2SO_4, filtered, and concentrated. The crude product was purified by flash chromatography (PE/EA = 20:1) to afford **S7**.

Analytical dates of 3a-3p

(*Z*)-*N*'-(1-(2-hydroxynaphthalen-1-yl)-4,4-dimethylpent-1-yn-3-ylidene)-4-methylbenzenesulfonohydrazide (3a)

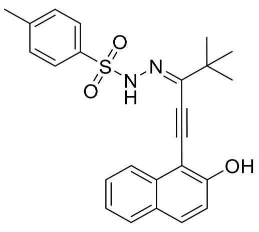

3a

Appearance: white solid.

^{1}H NMR (400 MHz, $CDCl_3$) δ 7.98 (d, *J* = 8.3 Hz, 1H), 7.89 (d, *J* = 8.2 Hz, 2H), 7.76 (t, *J* = 9.2 Hz, 2H), 7.53 (t, *J* = 7.6 Hz, 1H), 7.36 (t, *J* = 7.5 Hz, 1H), 7.30 (d, *J* = 8.9 Hz, 1H), 7.26 (d, *J* = 7.1 Hz, 2H), 2.37 (s, 3H), 1.26 (s, 9H).

^{13}C NMR (100 MHz, $CDCl_3$) δ 157.96, 148.21, 143.93, 135.49, 129.51, 128.40, 128.20, 127.88, 127.74, 124.37, 124.27, 116.86, 101.55, 100.75, 100.69, 89.56, 88.88, 37.12, 28.21, 21.54.

HRMS (ESI) m/z Calcd for $[C_{24}H_{24}N_2NaO_3S, M + Na]^+$: 443.1400, Found: 443.1407.

(*Z*)-*N*'-(4-(2-hydroxynaphthalen-1-yl)but-3-yn-2-ylidene)-4-methylbenzenesulfonohydrazide (3b)

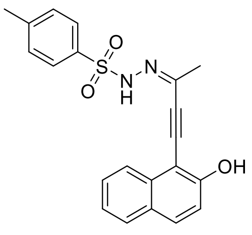

3b

Appearance: white solid.

^{1}H NMR (400 MHz, acetone-*d*$_6$) δ 10.17 (s, 1H), 9.71 (s, 1H), 8.07 (d, *J* = 8.4 Hz, 1H), 7.98 (d, *J* = 9.0 Hz, 1H), 7.90 (d, *J* = 8.1 Hz, 1H), 7.82 (d, *J* = 8.2 Hz, 2H), 7.60 (t, *J* = 7.5 Hz, 1H), 7.42 (dd, *J* = 14.7, 8.0 Hz, 4H), 2.41 (s, 3H), 2.19 (s, 3H).

.**^{13}C NMR** (100 MHz, acetone-*d*$_6$) δ 206.19, 159.79, 144.74, 137.60, 135.46, 134.24, 133.53, 130.46, 129.39, 129.03, 128.89, 128.48, 125.13, 125.06, 118.03, 101.79, 98.42, 90.78, 29.85, 22.21, 21.44.

HRMS (ESI) m/z Calcd for $[C_{21}H_{18}N_2NaO_3S, M + Na]^+$: 401.0930, Found: 401.0935.

(*Z*)-*N*'-(1-(2-hydroxynaphthalen-1-yl)pent-1-yn-3-ylidene)-4-methylbenzenesulfonohydrazide (3c)

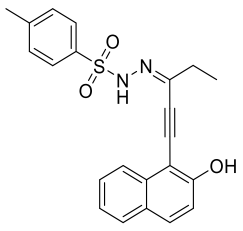

3c

Appearance: white solid.

^{1}H NMR (400 MHz, $CDCl_3$) δ 7.98 (d, *J* = 8.1 Hz, 1H), 7.90 (d, *J* = 7.0 Hz, 2H), 7.74 (t, *J* = 9.0 Hz, 2H), 7.52 (t, *J* = 7.0 Hz, 1H), 7.33 (dd, *J* = 19.0, 8.2 Hz, 2H), 7.24 (d, *J* = 7.3 Hz, 2H), 2.52 (q, *J* = 7.2, 6.6 Hz, 2H), 2.35 (s, 3H), 1.24 (t, *J* = 7.5 Hz, 3H).

^{13}C NMR (100 MHz, $CDCl_3$) δ 158.27, 144.01, 141.68, 135.45, 132.87, 132.55, 129.66, 128.33, 128.05, 127.63, 124.33, 124.22, 116.84, 101.23, 99.84, 89.83, 28.71, 21.52, 11.53.

HRMS (ESI) m/z Calcd for $[C_{22}H_{20}N_2NaO_3S, M + Na]^+$: 415.1087, Found: 415.1080.

(*Z*)-*N*'-(1-(2-hydroxynaphthalen-1-yl)-4-methylpent-1-yn-3-ylidene)-4-methylbenzenesulfonohydrazide (3d)

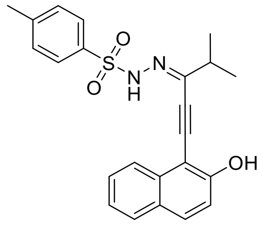

3d

Appearance: white solid.

^{1}H NMR (400 MHz, $CDCl_3$) δ 7.98 (d, *J* = 8.2 Hz, 1H), 7.91 (d, *J* = 8.1 Hz, 2H), 7.75 (t, *J* = 9.0 Hz, 2H), 7.53 (d, *J* = 7.2 Hz, 1H), 7.34 (dd, *J* = 10.9, 7.2 Hz, 2H), 7.24 (d, *J* = 6.1 Hz, 2H), 2.79 (dt, *J* = 13.4, 6.7 Hz, 1H), 2.35 (s, 3H), 1.23 (d, J = 6.7 Hz, 6H).

^{13}C NMR (100 MHz, $CDCl_3$) δ 158.25, 146.14, 144.01, 135.38, 132.84, 132.54, 129.62, 128.36, 128.09, 127.82, 127.64, 124.31, 124.23, 116.86, 101.32, 100.75, 88.86, 34.07, 21.53, 20.55.

HRMS (ESI) m/z Calcd for $[C_{23}H_{22}N_2NaO_3S, M + Na]^+$: 429.1243, Found: 429.1240.

(*Z*)-*N*'-(4-ethyl-1-(2-hydroxynaphthalen-1-yl)hex-1-yn-3-ylidene)-4-methylbenzenesulfonohydrazide (3e)

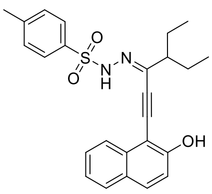

3e

Appearance: white solid.

^{1}H NMR (400 MHz, $CDCl_3$) δ 9.91 (s, 1H), 8.62 (s, 1H), 7.96 (d, *J* = 8.3 Hz, 1H), 7.90 (d, *J* = 8.2 Hz, 2H), 7.77 (dd, *J* = 14.8, 8.6 Hz, 2H), 7.53 (t, *J* = 7.6 Hz, 1H), 7.36 (dd, *J* = 8.0, 4.9 Hz, 2H), 7.23 (d, *J* = 8.1 Hz, 2H), 2.34 (m, 4H), 1.61 (dq, *J* = 13.8, 6.8 Hz, 4H), 0.71 (t, *J* = 7.4 Hz, 6H).

^{13}C NMR (100 MHz, $CDCl_3$) δ 158.45, 145.48, 144.01, 135.24, 132.86, 132.63, 129.98, 129.65, 128.39, 128.31, 128.10, 127.86, 127.68, 124.31, 124.25, 116.90, 101.34, 100.64, 88.60, 48.51, 26.35, 21.49, 11.51.

HRMS (ESI) m/z Calcd for $[C_{25}H_{26}N_2NaO_3S, M + Na]^+$: 457.1556, Found: 457.1558.

(*Z*)-*N*'-(1-cyclopentyl-3-(2-hydroxynaphthalen-1-yl)prop-2-yn-1-ylidene)-4-methylbenzenesulfonohydrazide (3f)

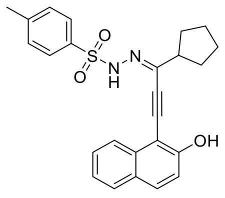

3f

Appearance: white solid.

^{1}H NMR (600 MHz, $CDCl_3$) δ 9.71 (s, 1H), 8.09 (s, 1H), 7.96 (d, J = 8.2 Hz, 1H), 7.90 (d, J = 7.9 Hz, 2H), 7.78 (d, J = 9.5 Hz, 1H), 7.76 (d, J = 8.3 Hz, 1H), 7.53 (t, J = 7.8 Hz, 1H), 7.37 (t, J = 7.7 Hz, 1H), 7.31 (d, J = 8.8 Hz, 1H), 7.28 – 7.24 (m, 2H), 2.95 (p, J = 8.0 Hz, 1H), 2.37 (s, 3H), 1.96 – 1.88 (m, 2H), 1.84 – 1.73 (m, 4H), 1.68 – 1.59 (m, 2H).

^{13}C NMR (150 MHz, $CDCl_3$) δ 158.05, 144.61, 144.00, 135.57, 132.93, 132.57, 129.65, 128.42, 128.21, 127.92, 127.72, 124.34, 116.84, 101.37, 99.87, 89.25, 44.90, 31.16, 25.55, 21.56.

HRMS (ESI) m/z Calcd for $[C_{25}H_{24}N_2NaO_3S, M + Na]^+$: 455.1400, Found: 455.1405.

(*Z*)-*N*'-(1-cyclohexyl-3-(2-hydroxynaphthalen-1-yl)prop-2-yn-1-ylidene)-4-methylbenzenesulfonohydrazide (3g)

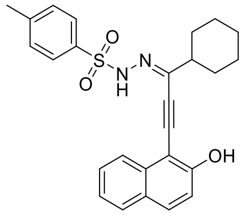

3g

Appearance: yellow solid.

^{1}H NMR (400 MHz, $CDCl_3$) δ 9.83 (s, 1H), 8.37 (s, 1H), 7.98 (d, *J* = 8.3 Hz, 1H), 7.90 (d, *J* = 8.2 Hz, 2H), 7.76 (t, *J* = 9.1 Hz, 2H), 7.54 (t, *J* = 7.5 Hz, 1H), 7.41 – 7.30 (m, 2H), 7.28 – 7.21 (m, 2H), 2.48 (t, *J* = 11.5, 3.3 Hz, 1H), 2.36 (s, 3H), 1.89 (dd, *J* = 12.3 Hz, 2H), 1.79 (dd, *J* = 12.5 Hz, 2H), 1.70 (dd, *J* = 10.9 Hz, 1H), 1.56 – 1.43 (m, 2H), 1.30 (td, *J* = 23.2, 21.8, 13.2 Hz, 3H).

^{13}C NMR (100 MHz, $CDCl_3$) δ 158.16, 145.23, 143.99, 135.46, 132.86, 132.53, 129.65, 128.38, 128.13, 127.84, 127.65, 124.38, 124.26, 116.85, 101.40, 100.51, 89.35, 43.29, 30.72, 25.78, 25.63, 21.56.

HRMS (ESI) m/z Calcd for $[C_{26}H_{26}N_2NaO_3S, M + Na]^+$: 469.1556, Found: 469.1561.

***N*'-((*Z*)-1-((3*r*,5*r*,7*r*)-adamantan-1-yl)-3-(2-hydroxynaphthalen-1-yl) prop-2-yn-1-ylidene)-4 methylbenzene-sulfonohydrazide (3h)**

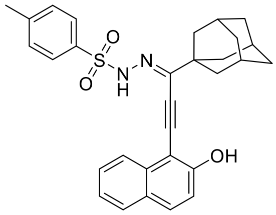

3h

Appearance: white solid.

^{1}H NMR (400 MHz, $CDCl_3$) δ 9.79 (s, 1H), 8.35 (s, 1H), 7.99 (d, *J* = 8.3 Hz, 1H), 7.91 (d, *J* = 8.0 Hz, 2H), 7.75 (t, *J* = 8.1 Hz, 2H), 7.54 (t, *J* = 7.5 Hz, 1H), 7.35 (t, *J* = 7.8 Hz, 2H), 7.26 (d, *J* = 8.4 Hz, 2H), 2.37 (s, 3H), 2.07 (m, 3H), 1.89 (m, 6H), 1.75 (m, *J* = 12.4 Hz, 6H).

^{13}C NMR (100 MHz, $CDCl_3$) δ 158.05, 148.60, 143.92, 135.41, 132.88, 132.38, 129.54, 128.37, 128.14, 127.81, 127.68, 124.38, 124.20, 116.88, 101.56, 100.94, 88.74, 40.36, 38.75, 36.61, 28.20, 21.56.

HRMS (ESI) m/z Calcd for $[C_{30}H_{30}N_2NaO_3S, M + Na]^+$: 521.1869, Found: 521.1863.

(*Z*)-*N*'-(3-(2-hydroxynaphthalen-1-yl)-1-phenylprop-2-yn-1-ylidene)-4-methylbenzenesulfonohydrazide (3i)

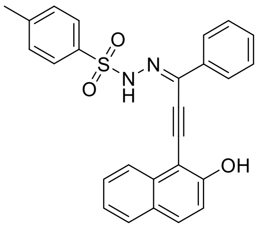

3i

Appearance: white solid.

^{1}H NMR (400 MHz, $CDCl_3$) δ 10.27 (s, 1H), 8.61 (s, 1H), 8.09 (d, *J* = 8.1 Hz, 1H), 7.99 (dd, *J* = 13.7, 7.3 Hz, 4H), 7.77 (dd, *J* = 15.7, 8.5 Hz, 2H), 7.57 (t, *J* = 7.2 Hz, 1H), 7.50 – 7.30 (m, 5H), 7.26 (d, *J* = 8.2 Hz, 3H), 2.34 (s, 3H).

^{13}C NMR (100 MHz, $CDCl_3$) δ 158.12, 144.27, 136.92, 135.55, 133.63, 132.86, 132.74, 130.07, 129.82, 128.46, 128.20, 127.98, 127.71, 126.70, 124.50, 124.39, 116.78, 101.74, 101.51, 88.86, 21.56.

HRMS (ESI) m/z Calcd for $[C_{26}H_{20}N_2NaO_3S, M + Na]^+$: 463.1087, Found: 463.1080.

(*Z*)-*N*'-(1-(2-hydroxynaphthalen-1-yl)-4,4-dimethylpent-1-yn-3-ylidene) benzenesulfonohydrazide (3j)

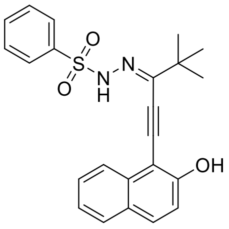

3j

Appearance: white solid.

^{1}H NMR (400 MHz, $CDCl_3$) δ 9.73 (s, 1H), 8.25 (s, 1H), 8.00 (dd, *J* = 15.7, 8.2 Hz, 3H), 7.77 (dd, *J* = 12.4, 8.7 Hz, 2H), 7.52 (dq, *J* = 21.6, 6.9 Hz, 4H), 7.37 (t, *J* = 7.5 Hz, 1H), 7.31 (d, *J* = 8.9 Hz, 1H), 1.25 (s, 9H).

^{13}C NMR (100 MHz, $CDCl_3$) δ 157.93, 148.54, 138.37, 133.10, 132.95, 132.51, 128.91, 128.41, 128.20, 127.93, 127.71, 124.38, 124.32, 116.78, 101.53, 100.87, 88.88, 37.13, 28.18.

HRMS (ESI) m/z Calcd for $[C_{23}H_{22}N_2NaO_3S, M + Na]^+$: 429.1243, Found: 429.1248.

(*Z*)-*N*'-(3-(2-hydroxynaphthalen-1-yl)-1-(p-tolyl)prop-2-yn-1-ylidene) benzenesulfonohydrazide (3k)

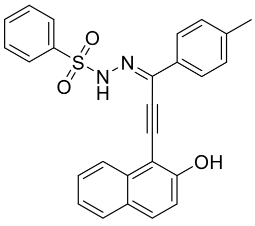

3k

Appearance: white solid.

^{1}H NMR (400 MHz, acetone-*d$_6$*) δ 8.20 (d, *J* = 8.3 Hz, 1H), 8.03 (dd, *J* = 12.2, 8.3 Hz, 3H), 7.93 (t, *J* = 9.1 Hz, 3H), 7.63 (p, *J* = 7.0 Hz, 4H), 7.50 – 7.41 (m, 2H), 7.30 (d, *J* = 7.9 Hz, 2H), 2.37 (s, 3H).

^{13}C NMR (100 MHz, acetone-*d$_6$*) δ 206.24, 160.12, 141.17, 140.40, 136.83, 134.14, 134.01, 133.69, 132.25, 130.18, 130.03, 129.43, 129.04, 129.01, 128.45, 127.21, 125.15, 118.00, 101.87, 101.69, 88.99, 30.42, 30.22, 30.03, 29.84, 29.65, 29.45, 29.26, 21.35.

HRMS (ESI) m/z Calcd for $[C_{26}H_{20}N_2NaO_3S, M + Na]^+$: 463.1087, Found: 463.1082.

(*Z*)-*N*'-(1-(2-hydroxynaphthalen-1-yl)-4,4-dimethylpent-1-yn-3-ylidene)-2,4,6-trimethylbenzenesulfonohydrazide (3l)

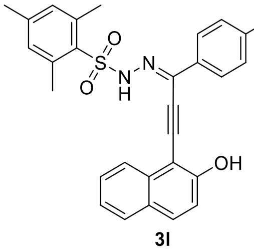

3l

Appearance: white solid.

^{1}H NMR (400 MHz, acetone-*d$_6$*) δ 8.23 (d, *J* = 8.3 Hz, 1H), 8.01 (d, *J* = 9.0 Hz, 1H), 7.90 (dd, *J* = 14.2, 8.2 Hz, 3H), 7.66 (t, *J* = 7.6 Hz, 1H), 7.52 – 7.41 (m, 2H), 7.28 (d, *J* = 7.9 Hz, 2H), 7.02 (s, 2H), 2.77 (s, 6H), 2.35 (s, 3H), 2.23 (s, 3H).

^{13}C NMR (100 MHz, acetone-*d$_6$*) δ 206.36, 160.19, 143.83, 141.01, 140.58, 135.41, 134.42, 134.11, 133.71, 132.65, 132.19, 130.21, 129.47, 129.08, 129.02, 126.98, 125.17, 125.08, 117.87, 101.95, 101.83, 88.67, 29.85, 23.36, 21.34, 20.87.

HRMS (ESI) m/z Calcd for $[C_{29}H_{26}N_2NaO_3S, M + Na]^+$: 505.1556, Found: 505.1550.

(*Z*)-*N*'-(1-(2-hydroxynaphthalen-1-yl)-4,4-dimethylpent-1-yn-3-ylidene)-4-(trifluoromethyl) benzenesulfonohydra-zide (3m)

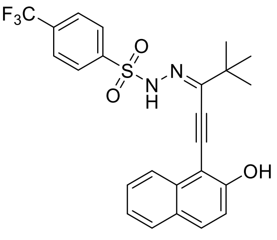

3m

Appearance: white solid.

^{1}H NMR (400 MHz, $CDCl_3$) δ 9.86 (s, 1H), 8.14 (d, *J* = 7.9 Hz, 2H), 8.09 (d, *J* = 9.1 Hz, 1H), 7.98 (d, *J* = 8.1 Hz, 1H), 7.83 – 7.68 (m, 4H), 7.54 (t, *J* = 7.3 Hz, 1H), 7.39 (q, *J* = 7.7, 7.2 Hz, 1H), 7.31 (d, *J* = 8.9 Hz, 1H), 1.27 (s, 9H).

^{13}C NMR (100 MHz, $CDCl_3$) δ 157.97, 149.34, 141.88, 134.80, 134.47, 132.94, 132.61, 128.29 (q, *J* = 22.4 Hz), 128.25, 127.99, 126.00 (q, *J* = 3.5 Hz), 124.47, 124.35 (q, *J* = 8.3 Hz), 121.83 (q, *J* = 240.1 Hz), 116.74, 101.47, 88.74, 77.00, 37.17, 29.85, 28.14.

HRMS (ESI) m/z Calcd for $[C_{24}H_{21}F_3N_2NaO_3S, M + Na]^+$: 497.1117, Found: 497.1113.

(*Z*)-4-chloro-*N*'-(1-(2-hydroxynaphthalen-1-yl)-4,4-dimethylpent-1-yn-3-ylidene) benzenesulfonohydrazide (3n)

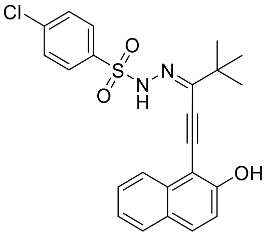

3n

Appearance: white solid.

^{1}H NMR (400 MHz, $CDCl_3$) δ 9.83 (s, 1H), 8.20 (s, 1H), 7.96 (t, *J* = 8.5 Hz, 3H), 7.76 (dd, *J* = 12.3, 8.8 Hz, 2H), 7.53 (t, *J* = 7.5 Hz, 1H), 7.43 (d, *J* = 8.4 Hz, 2H), 7.39 – 7.28 (m, 2H), 1.27 (s, 9H).

^{13}C NMR (100 MHz, $CDCl_3$) δ 157.88, 149.14, 139.73, 136.68, 132.88, 132.61, 129.24, 129.12, 128.40, 128.17, 127.96, 124.37, 116.70, 101.47, 101.27, 88.81, 37.15, 28.18.

HRMS (ESI) m/z Calcd for $[C_{23}H_{21}ClN_2NaO_3S, M + Na]^+$: 463.0854, Found: 463.0850.

(*Z*)-*N*'-(1-(7-bromo-2-hydroxynaphthalen-1-yl)-4,4-dimethylpent-1-yn-3-ylidene)-4-chlorobenzenesulfono-hydrazide (3o)

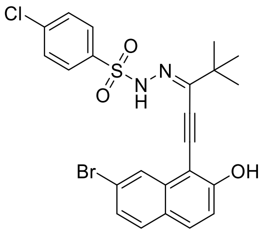

3o

Appearance: white solid.

^{1}H NMR (400 MHz, acetone-*d*$_6$) δ 10.47 (s, 1H), 9.66 (s, 1H), 8.19 (s, 1H), 8.00 (d, *J* = 9.1 Hz, 1H), 7.97 – 7.91 (m, 2H), 7.86 (d, *J* = 8.6 Hz, 1H), 7.67 (d, *J* = 8.6 Hz, 2H), 7.53 (dd, *J* = 8.7, 2.0 Hz, 1H), 7.44 (d, *J* = 9.0 Hz, 1H), 1.28 (s, 9H).

^{13}C NMR (100 MHz, acetone-*d*$_6$) δ 206.20, 160.56, 148.30, 139.65, 138.82, 135.45, 133.46, 131.46, 130.30, 130.06, 128.24, 127.45, 127.11, 123.05, 118.61, 101.30, 100.24, 89.08, 37.97, 29.85, 28.47.

HRMS (ESI) m/z Calcd for $[C_{23}H_{20}BrClN_2NaO_3S, M + Na]^+$: 540.9959, Found: 540.9952.

(*Z*)-*N*'-(1-(7-bromo-2-hydroxynaphthalen-1-yl)-4,4-dimethylpent-1-yn-3-ylidene)-4-methylbenzenesulfono-hydrazide (3p)

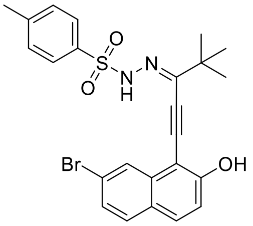

3p

Appearance: yellow solid.

^{1}H NMR (400 MHz, $CDCl_3$) δ 9.76 (s, 1H), 8.62 (s, 1H), 8.06 (s, 1H), 7.91 (d, J = 7.9 Hz, 2H), 7.71 (d, J = 8.9 Hz, 1H), 7.58 (d, J = 8.6 Hz, 1H), 7.39 (dd, J = 19.1, 8.7 Hz, 2H), 7.27 (d, J = 8.4 Hz, 2H), 2.38 (s, 3H), 1.27 (s, 9H).

^{13}C NMR (100 MHz, $CDCl_3$) δ 158.66, 148.41, 144.13, 135.20, 134.08, 132.26, 129.91, 129.60, 127.68, 126.71, 126.51, 122.46, 117.29, 100.85, 100.23, 89.10, 37.08, 28.19, 21.58.

HRMS (ESI) m/z Calcd for $[C_{24}H_{23}BrN_2NaO_3S, M + Na]^+$: 521.0505, Found: 521.0509.

General procedure for the synthesis of 3q-3aa

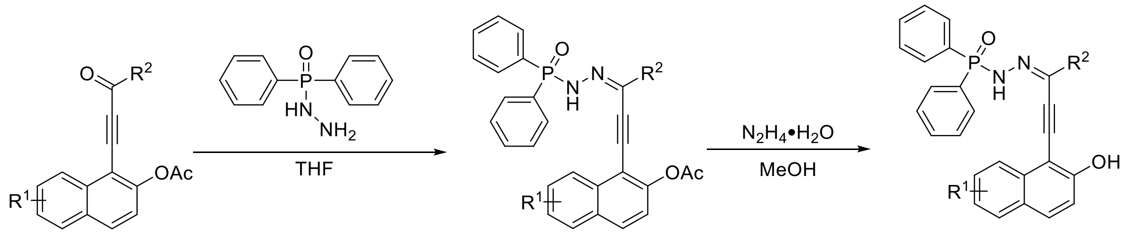

Starting materials **S6** from **General procedure for the synthesis of 3a-3p**

Step1: This step was carried out according to reported literature methods[7],[8] with some modification. Dissolve crude **S6** (1.0 equiv) and *P, P*-Diphenylphosphinic hydrazide in THF (1.0 mmol/mL). The mixture stirred 48 hours in room temperature. The reaction was quenched by pouring into cooled saturated NH_4Cl aqueous and the mixture was extracted with EA. The organic phases were combined, washed with brine, dried over Na_2SO_4, and concentrated to afford the crude product. The crude product was purified by flash chromatography (PE/EA = 20:1) to afford **S8**.

Step2: This step was carried out according to a literature method[9] with some modification. Dissolve crude **S8** (1.0 equiv) in MeOH and hydrazinium hydroxide solution (3.0 equiv) was dropwise added in the mixture. The mixture stirred 15 min in room temperature. After the reaction was completed by TLC monitored. The reaction was quenched by pouring into cooled saturated NH_4Cl aqueous and the mixture was extracted with EA. The organic phases were combined, washed with brine, dried over Na_2SO_4, and concentrated to afford the crude product. The purification of the crude mixture by flash column chromatography (PE/EA = 10:1) provided **3q-3aa**.

Analytical dates of 3a-3p

(*Z*)-*N*'-(1-(2-hydroxynaphthalen-1-yl)-4,4-dimethylpent-1-yn-3-ylidene)-P,P-diphenylphosphinic hydrazide (3q)

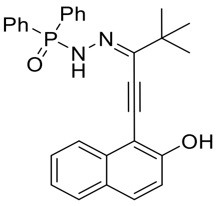

3q

Appearance: white solid.

^{1}H NMR (400 MHz, DMSO-d_6) δ 11.06 (s, 1H), 8.48 (d, J = 20.7 Hz, 1H), 7.97 (d, J = 8.3 Hz, 1H), 7.94 – 7.75 (m, 6H), 7.64 – 7.58 (m, 2H), 7.54 (t, J = 7.7 Hz, 5H), 7.38 (t, J = 7.4 Hz, 1H), 7.28 (d, J = 9.0 Hz, 1H), 1.20 (s, 9H).

^{13}C NMR (100 MHz, DMSO-d_6) δ 159.24, 144.10, 143.95, 133.12, 132.67, 132.07, 131.73, 131.64, 131.40, 128.60, 128.48, 127.96, 127.33, 123.81, 123.48, 117.71, 100.48, 99.37, 87.62, 39.50, 36.87, 28.24.

HRMS (ESI) m/z Calcd for $[C_{29}H_{27}N_2NaO_2P, M + Na]^+$: 489.1702, Found: 489.1707.

(*Z*)-*N*'-(4-ethyl-1-(2-hydroxynaphthalen-1-yl)hex-1-yn-3-ylidene)-P,Pdiphenylphosphinic hydrazide (3r)

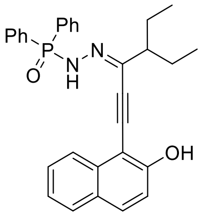

3r

Appearance: white solid.

^{1}H NMR (400 MHz, DMSO-d_6) δ 10.91 (s, 1H), 8.60 (d, *J* = 21.1 Hz, 1H), 8.02 – 7.77 (m, 7H), 7.67 – 7.50 (m, 7H), 7.40 (t, *J* = 7.4 Hz, 1H), 7.28 (d, *J* = 9.0 Hz, 1H), 2.27 – 2.16 (t, 1H), 1.63 (dtq, *J* = 20.4, 13.2, 7.2 Hz, 4H), 0.83 (t, *J* = 7.3 Hz, 6H).

^{13}C NMR (100 MHz, DMSO-d_6) δ 159.22, 140.64, 140.49, 133.15, 132.60, 132.16, 132.13, 132.11, 131.81, 131.71, 131.33, 128.68, 128.60, 128.55, 127.99, 127.35, 123.85, 123.42, 117.77, 100.39, 98.12, 87.49, 48.53, 39.50, 25.94, 11.73.

HRMS (ESI) m/z Calcd for [$C_{30}H_{29}N_2NaO_2P$, M + Na]$^+$: 503.1859, Found: 503.1854.

(*Z*)-*N*'-(1-cyclopropyl-3-(2-hydroxynaphthalen-1-yl)prop-2-yn-1-ylidene)-P,P-diphenylphosphinic hydrazide (3s)

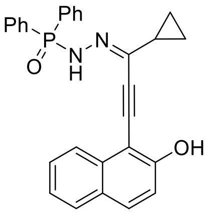

3s

Appearance: white solid.

^{1}H NMR (400 MHz, DMSO-d_6) δ 11.02 (s, 1H), 8.48 (d, *J* = 19.7 Hz, 1H), 8.00 – 7.70 (m, 7H), 7.54 (dd, *J* = 41.2, 6.8 Hz, 7H), 7.41 – 7.33 (m, 1H), 7.28 (d, *J* = 8.7 Hz, 1H), 1.86 (s, 1H), 0.89 (d, *J* = 9.6 Hz, 4H).

^{13}C NMR (100 MHz, DMSO-d_6) δ 159.46, 140.10, 139.94, 133.12, 132.54, 132.25, 132.12, 131.87, 131.78, 131.26, 128.77, 128.58, 127.94, 127.29, 123.83, 123.45, 117.78, 100.06, 97.85, 85.94, 39.50, 15.68, 6.03.

HRMS (ESI) m/z Calcd for [$C_{28}H_{23}N_2NaO_2P$, M + Na]$^+$: 473.1389, Found: 473.1382.

(*Z*)-*N*'-(1-cyclobutyl-3-(2-hydroxynaphthalen-1-yl)prop-2-yn-1-ylidene)-P,P-diphenylphosphinic hydrazide (3t)

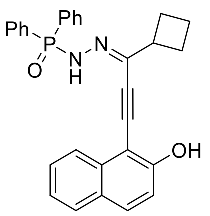

3t

Appearance: white solid.

^{1}H NMR (400 MHz, DMSO-d_6) δ 10.88 (s, 1H), 8.53 (d, *J* = 20.3 Hz, 1H), 7.99 (d, *J* = 8.3 Hz, 1H), 7.94 – 7.75 (m, 6H), 7.61 – 7.48 (m, 6H), 7.36 (t, *J* = 7.4 Hz, 1H), 7.26 (d, *J* = 9.0 Hz, 1H), 3.29 (m, *J* = 8.4 Hz, 1H), 2.32 – 2.20 (m, 2H), 2.19 – 2.09 (m, 2H), 1.99 – 1.87 (m, 1H), 1.86 – 1.76 (m, 1H).

^{13}C NMR (100 MHz, DMSO-d_6): δ 159.18, 140.44, 140.29, 133.21, 132.50, 132.22, 132.17, 132.14, 131.86, 131.77, 131.22, 128.76, 128.63, 128.61, 127.97, 127.39, 123.88, 123.55, 100.37, 98.44, 87.91, 39.50, 26.31, 17.91.

HRMS (ESI) m/z Calcd for [$C_{29}H_{25}N_2NaO_2P$, M + Na]$^+$: 487.1546, Found: 487.1541.

(*Z*)-*N*'-(1-cyclohexyl-3-(2-hydroxynaphthalen-1-yl)prop-2-yn-1-ylidene)-P,P-diphenylphosphinic hydrazide (3u)

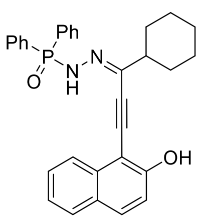

3u

Appearance: white solid.

^{1}H NMR (400 MHz, DMSO-d_6) δ 11.00 (s, 1H), 8.51 (d, *J* = 20.4 Hz, 1H), 7.97 (d, *J* = 8.3 Hz, 1H), 7.93 – 7.65 (m, 6H), 7.64 – 7.48 (m, 6H), 7.38 (t, *J* = 7.3 Hz, 1H), 7.27 (d, *J* = 8.9 Hz, 1H), 2.40 (m, 1H), 1.89 – 1.78 (m, 2H), 1.77 – 1.68 (m, 2H), 1.67 – 1.58 (m, 1H), 1.48 (m, *J* = 12.2, 3.2 Hz, 2H), 1.37 – 1.11 (m, 3H).

^{13}C NMR (100 MHz, DMSO-d_6) δ 159.20, 141.38, 141.23, 133.15, 132.58, 132.11, 131.80, 131.71, 131.31, 128.71, 128.58, 127.95, 127.34, 123.83, 123.52, 117.77, 100.40, 98.53, 88.09, 43.16, 39.50, 30.59, 25.54, 25.15.

HRMS (ESI) m/z Calcd for [$C_{31}H_{29}N_2NaO_2P$, M + Na]$^+$: 515.1859, Found: 515.1853.

***N'*-((*Z*)-1-((3*r*,5*r*,7*r*)-adamantan-1-yl)-3-(2-hydroxynaphthalen-1-yl)prop-2-yn-1ylidene)-P, P-diphenylphosphinic hydrazide (3v)**

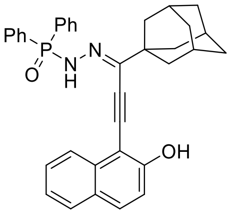
3v

Appearance: white solid.

^{1}H NMR (400 MHz, DMSO-*d*$_6$) δ 10.97 (s, 1H), 8.49 (d, *J* = 20.7 Hz, 1H), 8.00 (d, *J* = 8.3 Hz, 1H), 7.90 (dd, *J* = 13.8, 8.6 Hz, 2H), 7.82 (dd, *J* = 11.7, 7.5 Hz, 4H), 7.66 – 7.50 (m, 7H), 7.39 (t, *J* = 7.4 Hz, 1H), 7.27 (d, *J* = 9.0 Hz, 1H), 2.02 (m, 3H), 1.84 (m, 6H), 1.69 (m, *J* = 12.0 Hz, 6H).

^{13}C NMR (100 MHz, DMSO-*d*$_6$) δ 159.14, 144.55, 144.40, 133.09, 132.69, 132.07, 132.03, 131.73, 131.64, 131.42, 128.63, 128.50, 127.97, 127.36, 123.84, 123.51, 117.69, 100.54, 99.24, 87.46, 40.32, 39.50, 38.47, 36.21, 27.70.

HRMS (ESI) m/z Calcd for [$C_{35}H_{33}N_2NaO_2P$, M + Na]$^+$: 567.2172, Found: 567.2168.

(*Z*)-*N'*-(3-(2-hydroxynaphthalen-1-yl)-1-(p-tolyl)prop-2-yn-1-ylidene)-P,P-diphenylphosphinic hydrazide (3w)

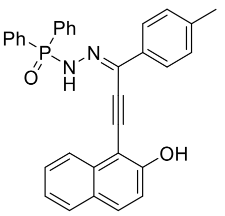
3w

Appearance: white solid.

^{1}H NMR (400 MHz, DMSO-*d*$_6$) δ 11.16 (s, 1H), 8.99 (d, *J* = 21.0 Hz, 1H), 8.10 (d, *J* = 8.3 Hz, 1H), 8.02 – 7.82 (m, 6H), 7.74 (d, *J* = 7.9 Hz, 2H), 7.69 – 7.49 (m, 7H), 7.40 (t, *J* = 7.4 Hz, 1H), 7.33 (d, *J* = 9.0 Hz, 1H), 7.26 (d, *J* = 7.8 Hz, 2H), 2.31 (s, 3H).

^{13}C NMR (100 MHz, DMSO-*d*$_6$) δ 159.48, 139.11, 134.19, 134.03, 133.12, 132.39, 132.28, 131.88, 131.79, 131.14, 129.37, 128.78, 128.65, 128.06, 127.39, 125.61, 123.92, 123.78, 117.71, 100.38, 99.95, 87.57, 39.50, 20.92.

HRMS (ESI) m/z Calcd for [$C_{32}H_{25}N_2NaO_2P$, M + Na]$^+$: 523.1546, Found: 523.1540.

(*Z*)-*N'*-(1-cyclopropyl-3-(2-hydroxy-7-methoxynaphthalen-1-yl) prop-2-yn-1-ylidene)-P, P-diphenylphosphinic hydrazide (3x)

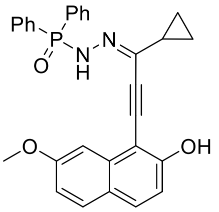
3x

Appearance: white solid.

^{1}H NMR (400 MHz, DMSO-*d*$_6$) δ 10.91 (s, 1H), 8.47 (d, *J* = 19.5 Hz, 1H), 7.97 – 7.69 (m, 6H), 7.68 – 7.44 (m, 6H), 7.24 (s, 1H), 7.06 (d, *J* = 8.9 Hz, 1H), 7.01 (d, *J* = 8.3 Hz, 1H), 3.82 (s, 3H), 1.90 – 1.81 (m, 1H), 1.07 – 0.82 (m, 4H).

^{13}C NMR (100 MHz, DMSO-*d*$_6$) δ 160.22, 159.07, 140.17, 140.01, 135.06, 132.55, 132.13, 132.06, 131.86, 131.77, 131.27, 130.27, 128.78, 128.66, 122.42, 115.76, 115.13, 102.61, 99.40, 98.32, 85.80, 54.95, 15.70, 5.96.

HRMS (ESI) m/z Calcd for [$C_{29}H_{25}N_2NaO_3P$, M + Na]$^+$: 503.1495, Found: 503.1491.

(*Z*)-*N'*-(1-(7-bromo-2-hydroxynaphthalen-1-yl)-4,4-dimethylpent-1-yn-3-ylidene)-P, P-diphenylphosphinic hydrazide (3y)

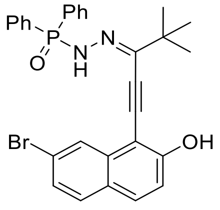
3y

Appearance: white solid.

^{1}H NMR (400 MHz, DMSO-*d*$_6$) δ 11.21 (s, 1H), 8.52 (d, *J* = 20.6 Hz, 1H), 8.14 (s, 1H), 7.94 (d, *J* = 9.0 Hz, 1H), 7.89 – 7.75 (m, 5H), 7.65 – 7.48 (m, 7H), 7.29 (d, *J* = 9.0 Hz, 1H), 1.19 (s, 9H).

^{13}C NMR (100 MHz, DMSO-*d*$_6$) δ 159.80, 143.66, 143.51, 134.51, 132.66, 132.08, 131.73, 131.63, 131.39, 130.86, 128.61, 128.48, 126.78, 125.90, 125.62, 121.73, 118.38, 99.88, 98.24, 87.87, 36.94, 28.19.

HRMS (ESI) m/z Calcd for [$C_{29}H_{26}BrN_2NaO_2P$, M + Na]$^+$: 567.0807, Found: 567.0801.

(*Z*)-*N'*-(1-(6-bromo-2-hydroxynaphthalen-1-yl)-4,4-dimethylpent-1-yn-3-ylidene)-P, P-diphenylphosphinic hydrazide (3z)

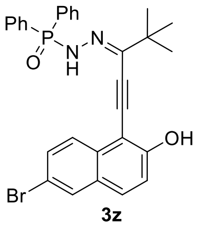

3z

Appearance: white solid.

^{1}H NMR (400 MHz, DMSO-d_6) δ 11.53 (s, 1H), 8.49 (d, *J* = 20.7 Hz, 1H), 8.14 (s, 1H), 7.98 – 7.67 (m, 6H), 7.58 (dd, *J* = 19.6, 13.4 Hz, 7H), 7.41 – 7.21 (m, 2H), 1.19 (s, 9H).

^{13}C NMR (100 MHz, DMSO-d_6) δ 159.88, 144.00, 143.85, 132.63, 132.03, 131.82, 131.74, 131.65, 131.36, 131.06, 130.61, 130.30, 128.59, 128.47, 125.75, 119.05, 116.40, 100.67, 98.77, 87.77, 39.50, 36.89, 28.23.

HRMS (ESI) m/z Calcd for [$C_{29}H_{26}BrN_2NaO_2P$, M + Na]$^+$: 567.0807, Found: 567.0812.

(*Z*)-*N'*-(1-(7-bromo-2-hydroxynaphthalen-1-yl)-4-ethylhex-1-yn-3-ylidene)-P,P-diphenylphosphinic hydrazide (3aa)

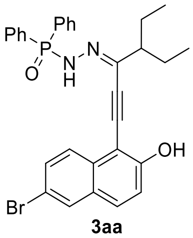

3aa

Appearance: white solid.

^{1}H NMR (400 MHz, DMSO-d_6) δ 11.10 (s, 1H), 8.64 (d, *J* = 21.0 Hz, 1H), 8.15 (s, 1H), 7.97 – 7.74 (m, 6H), 7.71 – 7.45 (m, 7H), 7.31 (d, *J* = 9.1 Hz, 1H), 2.18 (t, *J* = 9.4, 5.2 Hz, 1H), 1.71 – 1.46 (m, 4H), 0.80 (t, *J* = 7.3 Hz, 6H).

^{13}C NMR (100 MHz, DMSO-d_6) δ 159.83, 140.26, 140.11, 134.63, 132.61, 132.19, 132.11, 131.82, 131.72, 131.33, 130.87, 128.69, 128.57, 126.79, 125.91, 125.56, 121.76, 118.54, 99.76, 97.01, 87.78, 48.60, 39.50, 25.98, 11.73.

HRMS (ESI) m/z Calcd for [$C_{30}H_{28}BrN_2NaO_2P$, M + Na]$^+$: 581.0964, Found: 581.0959.

III. Optimization of the reaction conditions

Supplementary Table 1. Optimization of the reaction conditions for 2a [a]

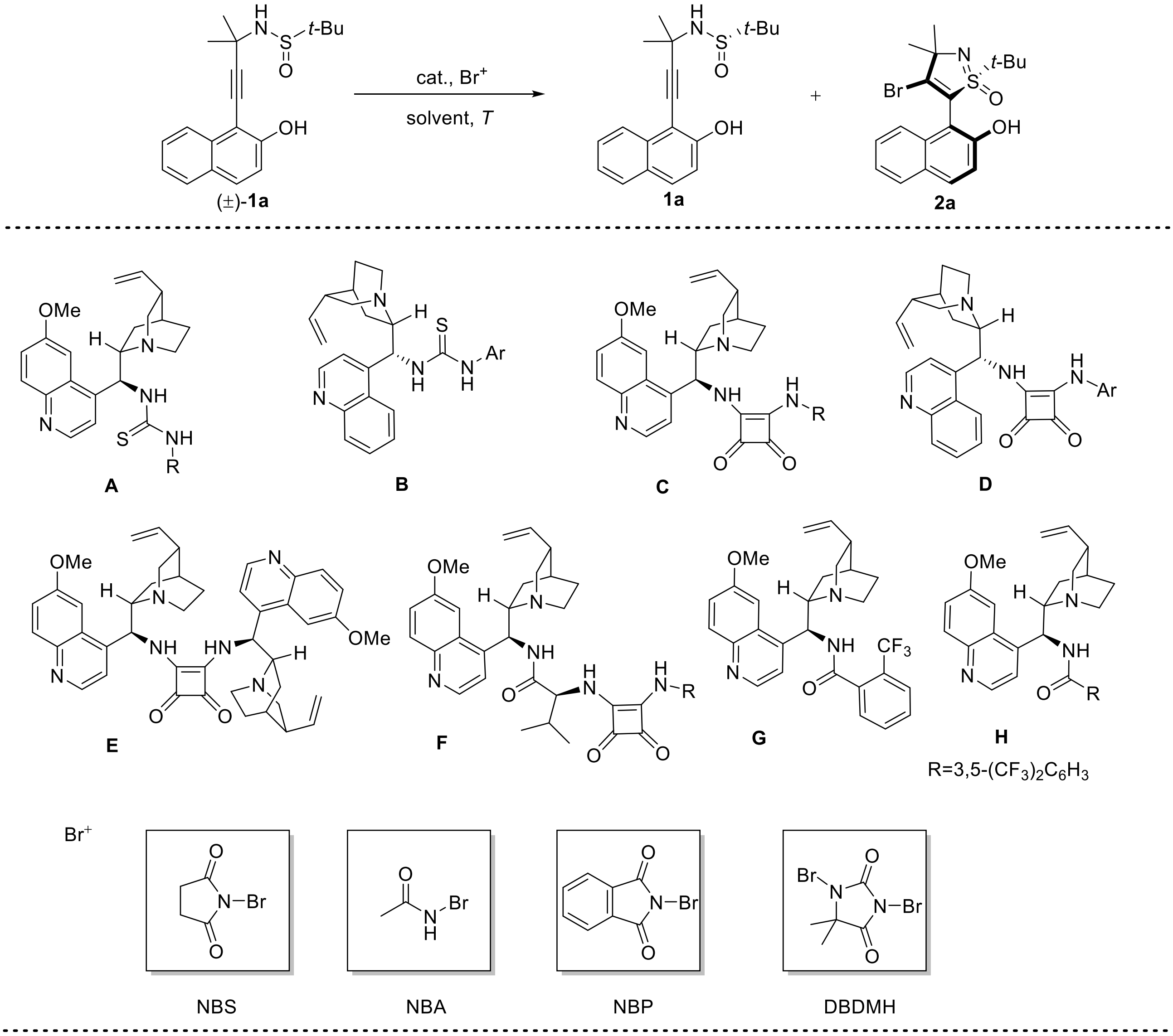

entry	catalyst	solvent	additive	*T* (°C)	d.r.[b]	ee (%)[c]		conv. (%)[d]	S[e]
						**1a**	**2a**		
1	**A**	toluene	NBS	-40	>20:1	< 5	< 5	-	-
2	**B**	toluene	NBS	-40	>20:1	16	36	31	**2**
3	**C**	toluene	NBS	-40	>20:1	9	-18	34	**2**
4	**D**	toluene	NBS	-40	>20:1	68	86	44	**27**
5	**E**	toluene	NBS	-40	>20:1	26	50	34	**4**
6	**F**	toluene	NBS	-40	>20:1	-9	-13	41	**1**
7	**G**	toluene	NBS	-40	>20:1	26	50	34	**4**
8	**H**	toluene	NBS	-40	>20:1	-9	-13	41	**1**
	**F**	*m*-xylene	NBS	-40	>20:1	48	77	38	**12**
8	**F**	Mesitylene	NBS	-40	>20:1	22	58	28	**5**
9	**F**	*i*-PrOH	NBS	-40	>20:1	12	56	18	**4**
10	**F**	CH_3CN	NBS	-40	>20:1	25	90	22	**24**
11	**F**	EA	NBS	-40	>20:1	50	73	41	**10**
12	**F**	DCM	NBS	-40	>20:1	74	84	47	**25**
13	**F**	THF	NBS	-40	>20:1	16	46	26	**3**
14	**F**	DCM	NBS	0	>20:1	41	79	34	**13**
15	**F**	DCM	NBS	-40	>20:1	74	84	47	**25**
16	**F**	DCM	NBS	-60	>20:1	53	93	36	**47**
14	**F**	DCM	NBS	-78	>20:1	76	95	44	**90**
15	**F**	DCM[f]	NBS	-78	>20:1	70	89	44	**36**
16	**F**	DCM[g]	NBS	-78	>20:1	80	93	46	**68**
17	**F**	DCM[g]	NBA	-78	>20:1	3	17	15	**2**
18	**F**	DCM[g]	NBP	-78	>20:1	71	96	43	**104**
19	**F**	DCM[g]	DMDBH	-78	>20:1	38	58	40	**5**

[a]Reaction conditions: (±)-**1a** (0.05 mmol), catalyst (10 mol%) in solvent (1.0 mL) at corresponding temperature for 10 min, then brominating reagents (0.0275 mmol, 0.55 equiv) were added at corresponding temperature, after the reaction was completed monitored by TLC, unless otherwise specified. Notes: *N*-bromosuccinimide (NBS); *N*-bromoacetamide (NBA); *N*-bromophthalimide (NBP); 1,3-dibromo-5,5-dimethylhydantoin (DBDMH). [b]Diastereomeric ratio (d.r.) was determined by HPLC. [c]Enantiomeric excess (ee) were determined by HPLC. [d]Conversion ratio was calculated by the methods of Fiaud: conv. = $ee_{1a}/(ee_{1a} + ee_{2a})$. [e]Selectivity values were calculated by the methods of Fiaud: $S = \ln[(1 - conv.)(1 - ee_{1a})]/\ln[(1 - conv.)(1 + ee_{1a})]$. [f]0.5 mL DCM. [g]2.0 mL DCM.

Supplementary Table 2. Optimization of the reaction conditions for 4a[a]

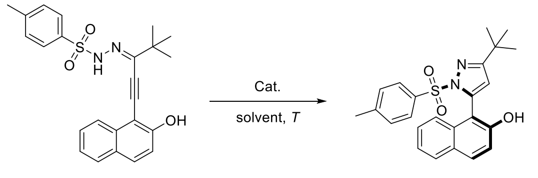

entry	catalyst	solvent	yield (%)[b]	ee (%)[c]
1	**A**	DCM	80	83
2	**B**	DCM	75	-60
3	**C**	DCM	83	91
4	**D**	DCM	81	-85
5	**E**	DCM	79	89
6	**F**	DCM	70	-25
7	**C**	DCE	80	79
8	**C**	chloroform	83	75
9	**C**	toluene	75	68
10	**C**	triflurotoluene	70	61
11	**C**	acetone	82	47
12	**C**	THF	81	20
13	**C**	1,4-dioxane	70	26
14	**C**	acetonitrile	75	10
15	**C**	DCM[d]	80	89
16	**C**	DCM[e]	82	87

[a]Reaction conditions: **3a** (0.05 mmol), catalyst (10 mol%) in solvent (2.0 mL) at 25 °C for 12 h, unless otherwise specified. [b]Isolated yield after purification by silica gel column chromatography. [c]Enantiomeric excess (ee) were determined by HPLC. [d]1.0 mL DCM. [e]4.0 mL DCM.

Supplementary Table 3. Optimization of the reaction conditions for 4q[a]

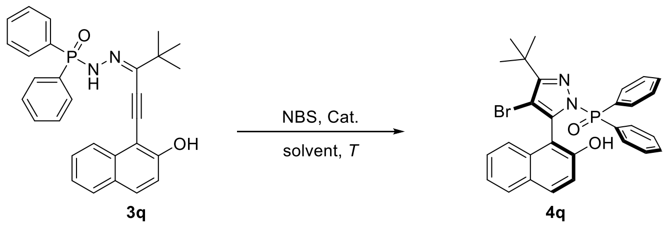

entry	catalyst	solvent	T (°C)	yield (%)[b]	ee (%)[c]
1	**A**	DCM	-40	75	11
2	**B**	DCM	-40	77	-13
3	**C**	DCM	-40	77	24
4	**D**	DCM	-40	73	-24
5	**E**	DCM	-40	75	55
6	**E**	DCE	-40	71	40
7	**E**	chloroform	-40	75	64
8	**E**	toluene	-40	74	96
9	**E**	chlorobenzene	-40	70	71
10	**E**	acetone	-40	75	49
11	**E**	acetonitrile	-40	70	78
12	**E**	THF	-40	72	13
13	**E**	toluene	-60	75	92
14	**E**	toluene	-20	76	92
15	**E**	toluene	25	74	94
16	**E**	toluene[d]	25	74	94
17	**E**	toluene[e]	25	75	90
18	**E**[f]	toluene	25	74	92
19	**E**[g]	toluene	25	76	91

[a]Reaction conditions: 3a (0.05 mmol), catalyst (20 mol%), NBS (0.0525 mmol) in solvent (2.0 mL) at corresponding temperature for 1 h, unless otherwise specified. [b]Isolated yields after purification by silica gel column chromatography. [c]The ee value was determined by HPLC analysis. [d]1.0 mL toluene. [e]4.0 mL toluene. [f]10 mol% catalyst. [g]5 mol% catalyst.

IV. General procedure for the asymmetric reaction

General procedure for the asymmetric synthesis of compounds 2a-2x

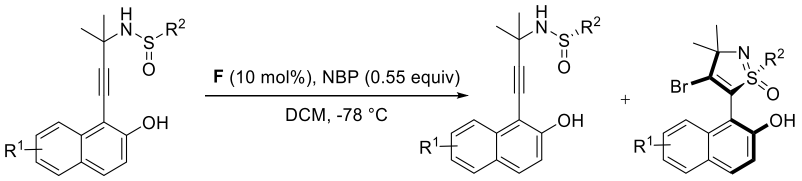

(±)-**1a-1x** (*S*)-**1a-1x** (*aS, S*)-**2a-2x**

A flame-dried Schlenk tube equipped with a magnetic stirring bar, was charged with (±)-**1a-1x** (0.3 mmol, 1.0 equiv), and catalyst **F** (10 mol%), DCM (12.0 mL) was injected into the tube at -78 ºC. After stirring for 10min, the NBP (0.165 mmol, 0.55 equiv) was added into the mixture, after the reaction was completed, monitored by TLC. The reaction was quenched with saturated ammonium chloride solution, and extracted with DCM (3×5.0 mL), all organic layer was remove under reduced pressure, and the residue was purified by column chromatography(PE:EA=3:1-6:1) to afford (*S*)-**1a-1x** and (*aS*, *S*)-**2a-2x**. Racemic samples were prepared without the addition of the catalyst.

Analytical dates of 1a-1x and 2a-2x

(*S*)-*N*-(4-(2-hydroxynaphthalen-1-yl)-2-methylbut-3-yn-2-yl)-2-methylpropane-2-sulfinamide (1a)

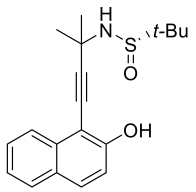

1a

Appearance: white solid.

Yield: 46%, 45 mg. **ee** = 71%.

^{1}H NMR (400 MHz, $CDCl_3$) δ 9.35 (s, 1H), 8.01 (d, J = 8.3 Hz, 1H), 7.70 (d, J = 8.1 Hz, 1H), 7.66 (d, J = 8.9 Hz, 1H), 7.47 (t, J = 7.6 Hz, 1H), 7.29 (t, J = 7.5 Hz, 1H), 7.15 (d, J = 8.9 Hz, 1H), 3.56 (s, 1H), 1.67 (s, 3H), 1.66 (s, 3H), 1.25 (s, 9H).

^{13}C NMR (100 MHz, $CDCl_3$) δ 158.80, 133.46, 130.09, 127.97, 127.78, 126.69, 124.37, 123.25, 118.39, 103.13, 101.53, 78.64, 55.83, 49.97, 31.39, 29.82, 22.26.

HRMS (ESI) m/z Calcd for $[C_{19}H_{23}NNaO_2S, M + Na]^+$: 352.1342, Found: 352.1347.

Optical Rotation: $[\alpha]_D^{25}$= 118.3° (*c* = 1.0, DCM).

HPLC analysis: Chiralcel IA-H (Hexane/*i*-PrOH = 93:7, flow rate = 1.0 mL/min, wave length = 254 nm), t_R = 5.640 min (minor), t_R = 8.700 min (major).

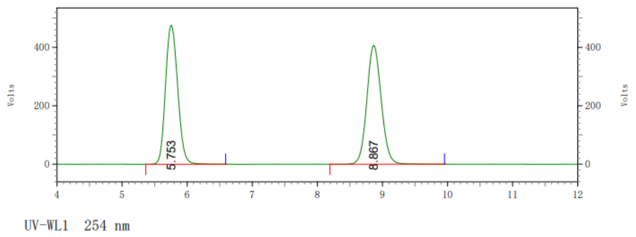

UV-WL1 254 nm

RetTime (min)	Width (min)	Height (Volts)	Area	Area (%)
5.753	1.23	1901740	24785289	49.768
8.867	1.76	1626633	25015993	50.232

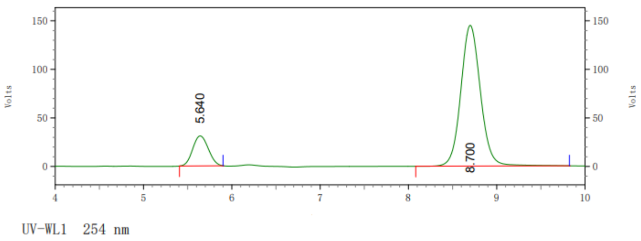

UV-WL1 254 nm

RetTime (min)	Width (min)	Height (Volts)	Area	Area (%)
5.640	0.49	123388	1515992	14.463
8.700	1.74	580128	8965763	85.537

(*aS*, *S*)-4-bromo-1-(tert-butyl)-5-(2-hydroxynaphthalen-1-yl)-3,3-dimethyl-3H-1λ[6]-isothiazole 1-oxide(2a)

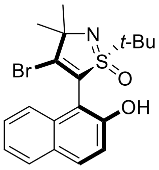

2a

Appearance: white solid.

Yield: 44%, 50 mg. **ee** = 96%.

^{1}H NMR (400 MHz, $CDCl_3$) δ 9.00 (s, 1H), 7.84 (d, *J* = 8.5 Hz, 2H), 7.54 – 7.47 (m, 1H), 7.45 (d, *J* = 8.2 Hz, 1H), 7.39 (t, *J* = 7.1 Hz, 1H), 7.25 (d, *J* = 9.0 Hz, 1H), 1.71 (s, 3H), 1.63 (s, 3H), 1.34 (s, 9H).

^{13}C NMR (100 MHz, $CDCl_3$) δ 156.74, 154.30, 132.27, 130.50, 130.11, 129.06, 127.31, 123.66, 123.36, 120.16, 108.28, 63.12, 30.67, 29.00, 24.27.

HRMS (ESI) m/z Calcd for [$C_{19}H_{22}BrNNaO_2S$, M + Na]$^+$: 430.0447, Found: 430.0452.

Optical Rotation: 150.4° (*c* = 1.0, DCM).

HPLC analysis: Chiralcel OJ-H (Hexane/*i*-PrOH = 96.5:3.5, flow rate = 1.0 mL/min, wave length = 254 nm), t_R = 5.170 min (major), t_R = 7.397 min (minor).

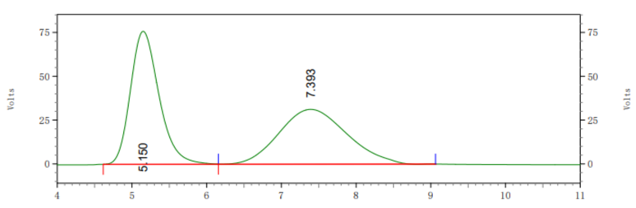

UV-WL1 254 nm

RetTime (min)	Width (min)	Height (Volts)	Area	Area (%)
5.150	1.54	303526	8073915	50.541
7.393	2.91	125076	7900975	49.459

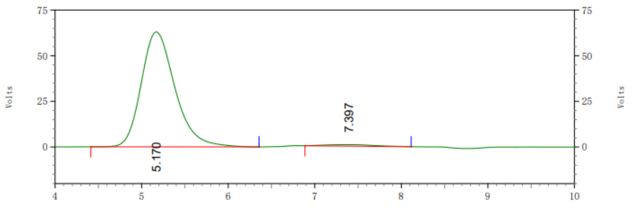

UV-WL1 254 nm

RetTime (min)	Width (min)	Height (Volts)	Area	Area (%)
5.170	1.94	251731	6819333	98.197
7.397	1.23	3273	125180	1.803

(*S*)-*N*-(4-(2-hydroxy-3-methoxynaphthalen-1-yl)-2-methylbut-3-yn-2-yl)-2-methylpropane-2-sulfinamide(1b)

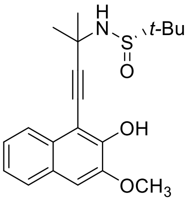

1b

Appearance: white solid.

Yield: 53%, 56 mg. **ee** = 31%.

^{1}H NMR (400 MHz, $CDCl_3$) δ 9.63 (s, 1H), 7.94 (d, *J* = 8.3 Hz, 1H), 7.68 – 7.60 (m, 1H), 7.36 (ddd, *J* = 8.2, 6.9, 1.4 Hz, 1H), 7.30 (ddd, *J* = 8.1, 6.9, 1.5 Hz, 1H), 7.08 (s, 1H), 3.97 (s, 3H), 3.53 (s, 1H), 1.69 (s, 6H), 1.26 (s, 9H).

^{13}C NMR (100 MHz, $CDCl_3$) δ 151.02, 148.94, 128.61, 128.23, 126.62, 124.42, 124.21, 123.90, 107.51, 103.07, 102.62, 78.76, 55.81, 50.19, 31.44, 30.01, 22.37.

HRMS (ESI) m/z Calcd for [$C_{20}H_{25}NNaO_3S$, M + Na]$^+$: 382.1447, Found: 382.1442.

Optical Rotation: $[\alpha]_D^{25}$= 67.4° (*c* = 1.0, DCM).

HPLC analysis: Chiralcel AD-H (Hexane/*i*-PrOH = 90: 10, flow rate = 1.0 mL/min, wave length = 254 nm), t_R = 9.243 min (minor), t_R = 10.463 min (major).

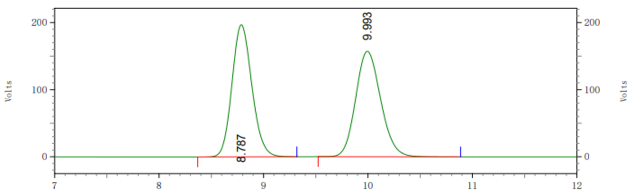

UV-WL1 254 nm

RetTime (min)	Width (min)	Height (Volts)	Area	Area (%)
8.787	0.95	787446	10455246	49.946
9.993	1.36	628558	10477718	50.054

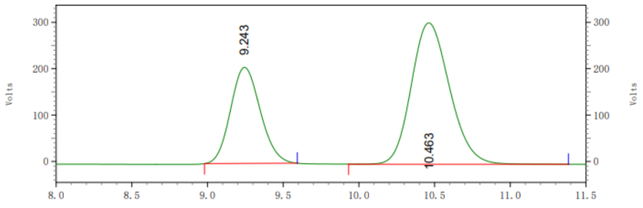

UV-WL1 254 nm

RetTime (min)	Width (min)	Height (Volts)	Area	Area (%)
9.243	0.61	828804	11347357	34.549
10.463	1.45	1219113	21496763	65.451

(*aS,S*)-4-bromo-1-(tert-butyl)-5-(2-hydroxy-3-methoxynaphthalen-1-yl)-3,3-dimethyl-3H-1λ[6]-isothiazole 1-oxide(2b)

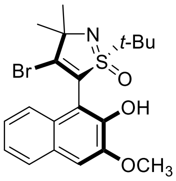

2b

Appearance: white solid.

Yield: 23%, 30 mg. **ee** = 96%.

^{1}H NMR (400 MHz, $CDCl_3$) δ 9.17 (s, 1H), 7.76 (dd, *J* = 6.7, 3.4 Hz, 1H), 7.38 (d, *J* = 3.4 Hz, 3H), 7.23 (s, 1H), 4.02 (s, 3H), 1.71 (s, 3H), 1.62 (s, 3H), 1.34 (s, 9H).

^{13}C NMR (100 MHz, $CDCl_3$) δ 157.04, 149.35, 146.38, 129.82, 129.42, 127.63, 125.27, 124.91, 124.29, 123.18, 109.51, 108.89, 63.16, 55.87, 30.67, 28.88, 24.29.

HRMS (ESI) m/z Calcd for $[C_{20}H_{24}BrNNaO_3S, M + Na]^+$: 460.0552, Found: 460.0559.

Optical Rotation: $[\alpha]_D^{25}$= 124.7° (*c* = 1.0, DCM).

HPLC analysis: Chiralcel OD-H (Hexane/*i*-PrOH = 85:15, flow rate = 1.0 mL/min, wave length = 254 nm), t_R = 5.297 min (major), t_R = 5.873 min (minor).

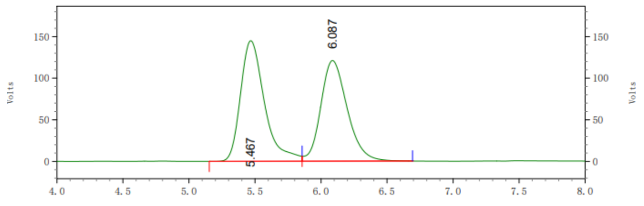

UV-WL1 254 nm

RetTime (min)	Width (min)	Height (Volts)	Area	Area (%)
5.467	0.70	579401	7305429	52.092
6.087	0.84	483364	6718780	47.908

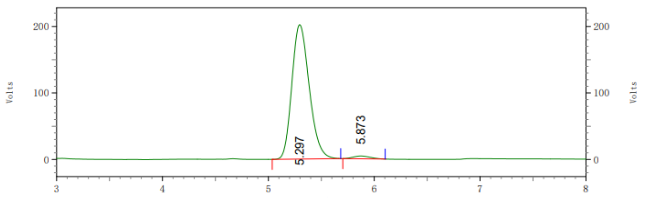

UV-WL1 254 nm

RetTime (min)	Width (min)	Height (Volts)	Area	Area (%)
5.297	0.65	808147	9218780	97.998
5.873	0.40	16619	188332	2.002

(*S*)-*N*-(4-(6-ethyl-2-hydroxynaphthalen-1-yl)-2-methylbut-3-yn-2-yl)-2-methylpropane-2-sulfinamide (1c)

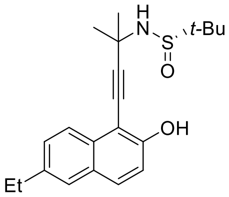

1c

Appearance: white solid.

Yield: 46%, 49 mg. **ee** = 82%.

^{1}H NMR (400 MHz, $CDCl_3$) δ 9.18 (s, 1H), 7.93 (d, *J* = 8.5 Hz, 1H), 7.61 (d, *J* = 8.9 Hz, 1H), 7.50 (d, *J* = 1.7 Hz, 1H), 7.34 (dd, *J* = 8.6, 1.8 Hz, 1H), 7.11 (d, *J* = 8.9 Hz, 1H), 3.52 (s, 1H), 2.76 (q, *J* = 7.6 Hz, 2H), 1.68 (d, *J* = 3.1 Hz, 6H), 1.30 (d, *J* = 7.6 Hz, 3H), 1.27 (s, 9H).

^{13}C NMR (100 MHz, $CDCl_3$) δ 158.34, 139.12, 131.91, 129.70, 128.10, 127.87, 125.89, 124.44, 118.38, 102.95, 101.49, 78.96, 55.91, 50.10, 31.58, 29.93, 28.66, 22.39, 15.59.

HRMS (ESI) m/z Calcd for $[C_{21}H_{27}NNaO_2S, M + Na]^+$: 380.1655, Found: 380.1650.

Optical Rotation: $[\alpha]_D^{25}$= 15.0° (*c* = 1.0, DCM).

HPLC analysis: Chiralcel OD-H (Hexane/*i*-PrOH = 85:15, flow rate = 1.0 mL/min, wave length = 254 nm), t_R = 3.970 min (minor), t_R = 5.193 min (major).

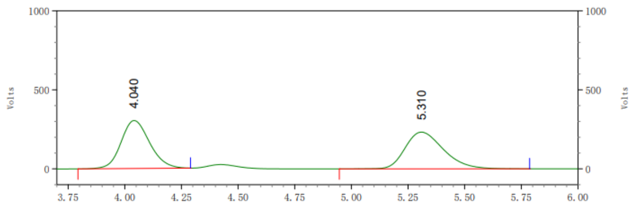

UV-WL1 254 nm

RetTime (min)	Width (min)	Height (Volts)	Area	Area (%)
4.040	0.50	1214698	10669782	49.314
5.310	0.84	930258	10966502	50.686

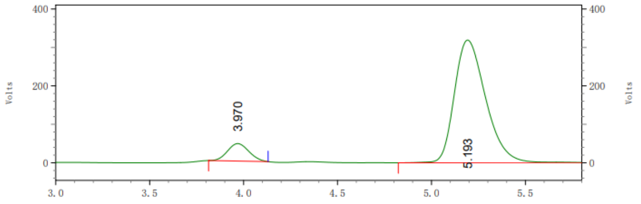

UV-WL1 254 nm

RetTime (min)	Width (min)	Height (Volts)	Area	Area (%)
3.970	0.32	182031	1441745	8.914
5.193	0.99	1275697	14731348	91.086

(*aS*, *S*)-4-bromo-1-(tert-butyl)-5-(6-ethyl-2-hydroxynaphthalen-1-yl)-3,3-dimethyl-3H-1λ^6-isothiazole 1-oxide (2c)

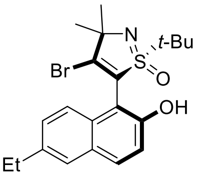

2c

Appearance: white solid.

Yield: 45%, 58.7 mg. **ee** = 90%.

^{1}H NMR (400 MHz, CDCl$_3$) δ 8.91 (s, 1H), 7.78 (d, *J* = 8.8 Hz, 1H), 7.63 (s, 1H), 7.37 (s, 2H), 7.22 (d, *J* = 8.9 Hz, 1H), 2.80 (q, *J* = 7.4 Hz, 2H), 1.71 (s, 3H), 1.62 (s, 3H), 1.34 (s, 12H).

^{13}C NMR (100 MHz, CDCl$_3$) δ 156.50, 153.64, 139.42, 134.26, 131.80, 130.25, 129.26, 128.81, 128.45, 126.83, 123.54, 123.38, 120.06, 108.16, 63.09, 30.66, 29.01, 28.44, 24.29, 15.18.

HRMS (ESI) m/z Calcd for [$C_{21}H_{26}BrNNaO_2S$, M + Na]$^+$: 458.0760, Found: 458.0754.

Optical Rotation: $[\alpha]_D^{25}$= 130.5° (*c* = 1.0, DCM).

HPLC analysis: Chiralcel AD-H (Hexane/*i*-PrOH = 99:1, flow rate = 1.0 mL/min, wave length = 254 nm), t_R = 14.673 min (minor), t_R = 16.830 min (major).

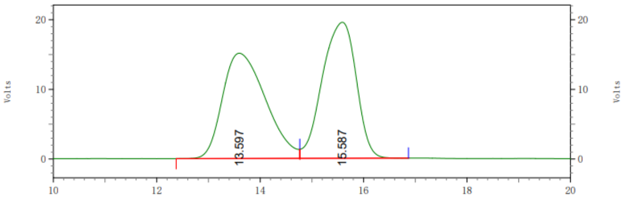

UV-WL1 254 nm

RetTime (min)	Width (min)	Height (Volts)	Area	Area (%)
13.597	2.39	60429	3490104	49.510
15.587	2.10	78073	3559217	50.490

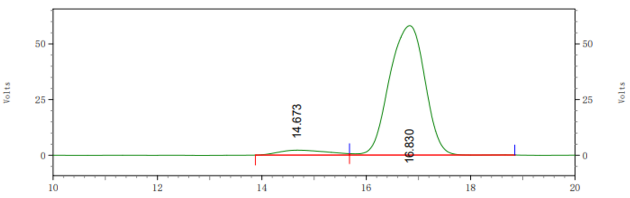

UV-WL1 254 nm

RetTime (min)	Width (min)	Height (Volts)	Area	Area (%)
14.673	1.80	8834	574099	4.983
16.830	3.17	232421	10947153	95.017

(*S*)-*N*-(4-(2-hydroxy-6-phenylnaphthalen-1-yl)-2-methylbut-3-yn-2-yl)-2-methylpropane-2-sulfinamide (1d)

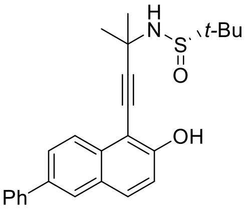

1d

Appearance: white solid.

Yield: 50%, 60.8 mg. **ee** = 74%.

^{1}H NMR (400 MHz, CDCl$_3$) δ 9.36 (s, 1H), 8.07 (d, *J* = 8.6 Hz, 1H), 7.96 – 7.90 (m, 1H), 7.80 – 7.70 (m, 2H), 7.68 (d, *J* = 7.8 Hz, 2H), 7.46 (t, *J* = 7.6 Hz, 2H), 7.35 (t, *J* = 7.4 Hz, 1H), 7.18 (d, *J* = 8.9 Hz, 1H), 3.55 (s, 1H), 1.70 (d, *J* = 2.5 Hz, 6H), 1.28 (s, 9H).

^{13}C NMR (100 MHz, CDCl$_3$) δ 159.04, 141.07, 136.11, 132.80, 130.47, 128.78, 128.14, 127.15, 127.02, 126.35, 126.03, 125.06, 118.97, 103.19, 101.59, 78.78, 55.96, 50.13, 31.56, 29.92, 22.40.

HRMS (ESI) m/z Calcd for [$C_{25}H_{27}NNaO_2S$, M + Na]$^+$: 428.1655, Found: 428.1660.

Optical Rotation: $[\alpha]_D^{25}$= 44.0° (*c* = 1.0, DCM).

HPLC analysis: Chiralcel IA-H (Hexane/*i*-PrOH = 85:15, flow rate = 1.0 mL/min, wave length = 254 nm), t_R = 4.903 min (minor), t_R = 8.563 min (major).

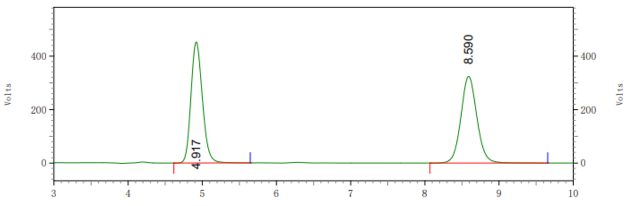

UV-WL1 254 nm

RetTime (min)	Width (min)	Height (Volts)	Area	Area (%)
4.917	1.03	1810252	19400616	49.935
8.590	1.59	1296265	19450744	50.065

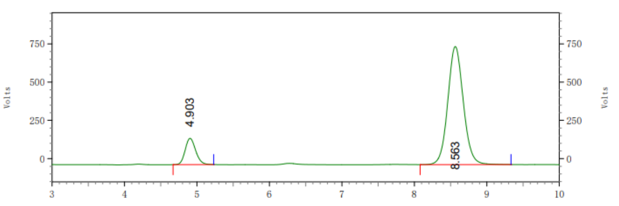

UV-WL1 254 nm

RetTime (min)	Width (min)	Height (Volts)	Area	Area (%)
4.903	0.56	686050	6919365	13.087
8.563	1.25	3087489	45952768	86.913

(*aS, S*)-4-bromo-1-(tert-butyl)-5-(2-hydroxy-6-phenylnaphthalen-1-yl)-3,3-dimethyl-3H-1λ^6-isothiazole 1-oxide (2d)

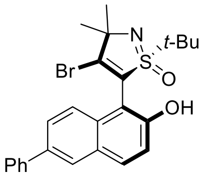

2d

Appearance: white solid.

Yield: 42%, 60.8 mg. **ee** = 95%.

^{1}H NMR (400 MHz, $CDCl_3$) δ 9.04 (s, 1H), 8.08 – 8.01 (m, 1H), 7.88 (dd, *J* = 12.2, 7.4 Hz, 2H), 7.83 – 7.73 (m, 2H), 7.71 (d, *J* = 7.7 Hz, 2H), 7.58 – 7.43 (m, 3H), 7.37 (t, *J* = 7.3 Hz, 1H), 7.26 (s, 1H), 1.73 (s, 3H), 1.64 (s, 3H), 1.36 (s, 9H).

^{13}C NMR (100 MHz, $CDCl_3$) δ 167.93, 156.80, 154.39, 140.37, 136.40, 134.26, 132.54, 130.08, 129.69, 129.34, 128.88, 127.34, 127.09, 126.90, 126.85, 123.99, 123.54, 120.63, 108.25, 63.16, 30.67, 29.02, 24.31.

HRMS (ESI) m/z Calcd for $[C_{25}H_{26}BrNNaO_2S, M + Na]^+$: 506.0760, Found: 506.0767.

Optical Rotation: $[\alpha]_D^{25}$= 50.4° (*c* = 1.0, DCM).

HPLC analysis: Chiralcel IC-H (Hexane/*i*-PrOH = 85:15, flow rate = 1.0 mL/min, wave length = 254 nm), t_R = 8.737 min (minor), t_R = 13.577 min (major).

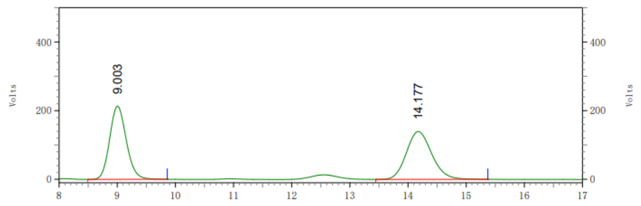

UV-WL1 254 nm

RetTime (min)	Width (min)	Height (Volts)	Area	Area (%)
9.003	1.36	853553	17011544	50.096
14.177	1.93	557356	16946367	49.904

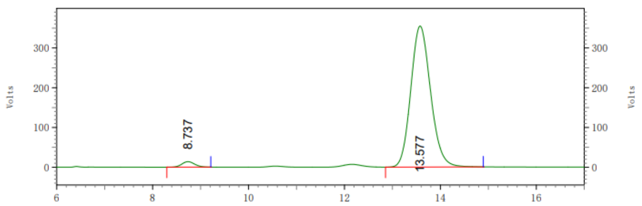

UV-WL1 254 nm

RetTime (min)	Width (min)	Height (Volts)	Area	Area (%)
8.737	0.92	55268	1036658	2.484
13.577	2.04	1418646	40693703	97.516

(*S*)-*N*-(4-(6-bromo-2-hydroxynaphthalen-1-yl)-2-methylbut-3-yn-2-yl)-2-methylpropane-2-sulfinamide (1e)

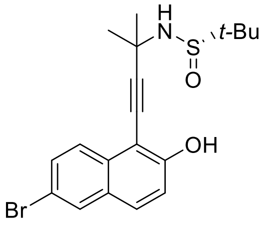

1e

Appearance: white solid.

Yield: 49%, 60 mg. **ee** = 82%.

^{1}H NMR (400 MHz, $CDCl_3$) δ 9.45 (s, 1H), 7.86 (d, *J* = 8.7 Hz, 2H), 7.56 (d, *J* = 9.0 Hz, 1H), 7.51 (dd, *J* = 8.9, 1.7 Hz, 1H), 7.15 (d, *J* = 9.0 Hz, 1H), 3.54 (s, 1H), 1.68 (d, *J* = 2.6 Hz, 6H), 1.27 (s, 9H).

^{13}C NMR (100 MHz, $CDCl_3$) δ 159.15, 132.10, 129.92, 129.11, 126.33, 119.66, 116.86, 103.53, 101.87, 78.31, 55.97, 50.05, 31.44, 29.81, 22.35.

HRMS (ESI) m/z Calcd for $[C_{19}H_{22}BrNNaO_2S, M + Na]^+$: 430.0447, Found: 430.0450.

Optical Rotation: $[\alpha]_D^{25}$= 34.0° (*c* = 1.0, DCM).

HPLC analysis: Chiralcel IA-H (Hexane/*i*-PrOH = 85:15, flow rate = 1.0 mL/min, wave length = 254 nm), t_R = 4.127 min (minor), t_R = 7.377 min (major).

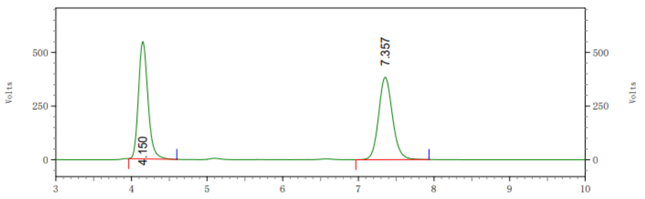

UV-WL1 254 nm

RetTime (min)	Width (min)	Height (Volts)	Area	Area (%)
4.150	0.64	2184268	19147648	49.453
7.357	0.97	1534818	19570930	50.547

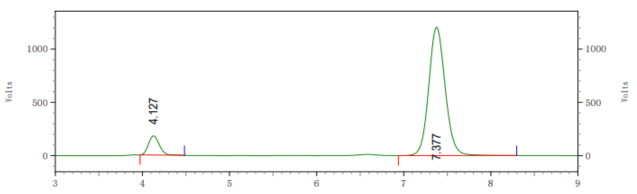

UV-WL1 254 nm

RetTime (min)	Width (min)	Height (Volts)	Area	Area (%)
4.127	0.51	718529	6268743	9.026
7.377	1.36	4818632	63185869	90.974

(*aS, S*)-4-bromo-5-(6-bromo-2-hydroxynaphthalen-1-yl)-1-(tert-butyl)-3,3-dimethyl-3H-1λ⁶-isothiazole 1-oxide (2e)

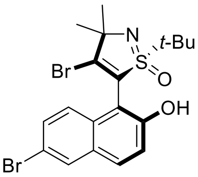

2e

Appearance: white solid.

Yield: 43%, 62.8 mg. **ee** = 94%.

^{1}H NMR (400 MHz, $CDCl_3$) δ 9.09 (s, 1H), 7.99 (d, *J* = 1.7 Hz, 1H), 7.75 (d, *J* = 9.0 Hz, 1H), 7.57 (dd, *J* = 9.0, 1.9 Hz, 1H), 7.31 (d, *J* = 9.0 Hz, 1H), 7.29 – 7.23 (m, 1H), 1.71 (s, 3H), 1.61 (s, 3H), 1.33 (s, 9H).

^{13}C NMR (100 MHz, $CDCl_3$) δ 157.14, 154.63, 131.27, 131.11, 130.53, 130.17, 129.74, 129.12, 125.17, 121.44, 117.40, 108.56, 63.16, 30.63, 28.99, 24.28.

HRMS (ESI) m/z Calcd for $[C_{19}H_{21}Br_2NNaO_2S, M + Na]^+$: 507.9552, Found: 507.9550.

Optical Rotation: $[\alpha]_D^{25}$= 80.4° (*c* = 1.0, DCM).

HPLC analysis: Chiralcel IC-H (Hexane/*i*-PrOH = 94:6, flow rate = 1.0 mL/min, wave length = 254 nm), t_R = 11.577 min (minor), t_R = 16.223 min (major).

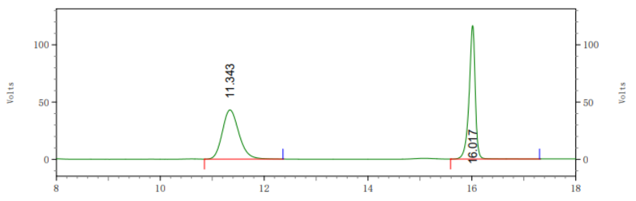

UV-WL1 254 nm

RetTime (min)	Width (min)	Height (Volts)	Area	Area (%)
11.343	1.51	171745	3817846	49.789
16.017	1.71	466228	3850278	50.211

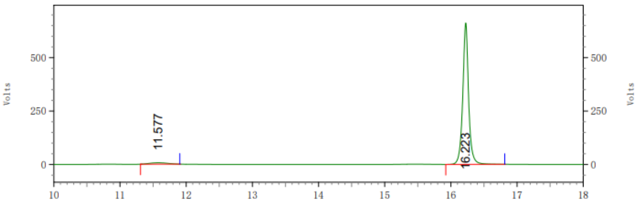

UV-WL1 254 nm

RetTime (min)	Width (min)	Height (Volts)	Area	Area (%)
11.577	0.59	26836	499076	2.974
16.223	0.89	2647788	16282381	97.026

(*S*)-*N*-(4-(2-hydroxy-7-methoxynaphthalen-1-yl)-2-methylbut-3-yn-2-yl)-2-methylpropane-2-sulfinamide (1f)

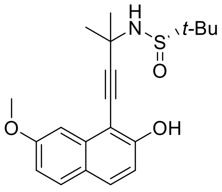

1f

Appearance: white solid.

Yield: 41%, 44 mg. **ee** =82 %.

^{1}H NMR (400 MHz, $CDCl_3$) δ 9.21 (s, 1H), 7.60 (t, *J* = 8.8 Hz, 2H), 7.34 (d, *J* = 1.9 Hz, 1H), 7.06 – 6.89 (m, 2H), 3.92 (s, 3H), 3.53 (s, 1H), 1.68 (d, *J* = 4.6 Hz, 6H), 1.27 (s, 9H).

^{13}C NMR (100 MHz, $CDCl_3$) δ 159.43, 158.70, 135.07, 130.01, 129.67, 123.23, 115.94, 114.89, 104.11, 103.29, 100.87, 78.92, 55.95, 55.14, 50.10, 31.69, 30.02, 22.40.

HRMS (ESI) m/z Calcd for $[C_{20}H_{25}NNaO_3S, M + Na]^+$: 382.1447, Found: 382.1450.

Optical Rotation: $[\alpha]_D^{25}$= 34.3° (*c* = 1.0, DCM).

HPLC analysis: Chiralcel IA-H (Hexane/*i*-PrOH = 85:15, flow rate = 1.0 mL/min, wave length = 254 nm), t_R = 4.377 min (minor), t_R = 5.933 min (major).

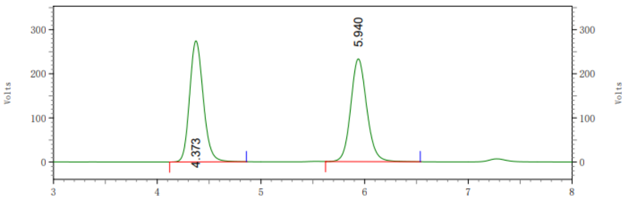

UV-WL1 254 nm

RetTime (min)	Width (min)	Height (Volts)	Area	Area (%)
4.373	0.74	1097604	10037940	50.032
5.940	0.91	931691	10024900	49.968

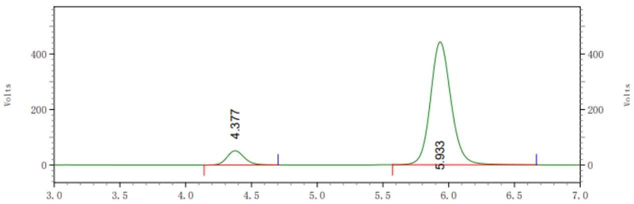

UV-WL1 254 nm

RetTime (min)	Width (min)	Height (Volts)	Area	Area (%)
4.377	0.56	203975	1856076	8.800
5.933	1.09	1771283	19236087	91.200

(*aS,S*)-4-bromo-1-(tert-butyl)-5-(2-hydroxy-7-methoxynaphthalen-1-yl)-3,3-dimethyl-3H-1λ^6-isothiazole 1-oxide (2f)

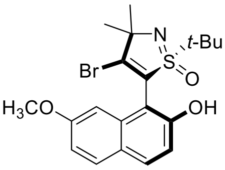

2f

Appearance: white solid.

Yield: 43%, 56 mg. **ee** = 92%.

^{1}H NMR (400 MHz, $CDCl_3$) δ 8.91 (s, 1H), 7.74 (t, *J* = 10.0 Hz, 2H), 7.06 (dd, *J* = 20.2, 8.4 Hz, 2H), 6.77 (s, 1H), 3.81 (s, 3H), 1.72 (s, 3H), 1.61 (s, 3H), 1.36 (s, 9H).

^{13}C NMR (100 MHz, $CDCl_3$) δ 158.90, 156.36, 154.93, 132.00, 130.53, 124.21, 117.47, 115.75, 107.46, 102.91, 63.04, 54.93, 30.47, 29.13, 24.29.

HRMS (ESI) m/z Calcd for $[C_{20}H_{24}BrNNaO_3S, M + Na]^+$: 460.0552, Found: 460.0557.

Optical Rotation: $[\alpha]_D^{25}$= 80.6° (*c* = 1.0, DCM).

HPLC analysis: Chiralcel AD-H (Hexane/*i*-PrOH = 97:3, flow rate = 1.0 mL/min, wave length = 254 nm), t_R = 10.140 min (minor), t_R = 11.063 min (major).

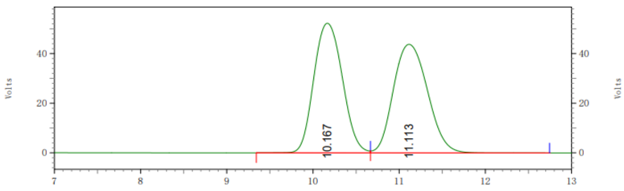

UV-WL1 254 nm

RetTime (min)	Width (min)	Height (Volts)	Area	Area (%)
10.167	1.32	208967	4813021	49.968
11.113	2.08	175014	4819201	50.032

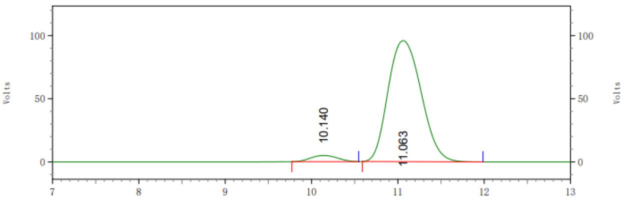

UV-WL1 254 nm

RetTime (min)	Width (min)	Height (Volts)	Area	Area (%)
10.140	0.77	19356	418967	3.940
11.063	1.40	382900	10213678	96.060

(*S*)-*N*-(4-(2-hydroxy-7-phenylnaphthalen-1-yl)-2-methylbut-3-yn-2-yl)-2-methylpropane-2-sulfinamide (1g)

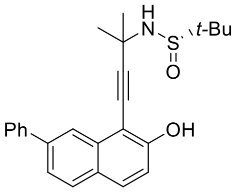

1g

Appearance: white solid.

Yield: 50%, 60.8 mg. **ee** = 66%.

^{1}H NMR (400 MHz, $CDCl_3$) δ 9.33 (s, 1H), 8.20 (s, 1H), 7.79 (d, *J* = 8.4 Hz, 1H), 7.71 (t, *J* = 8.8 Hz, 3H), 7.56 (d, *J* = 8.4 Hz, 1H), 7.50 (t, *J* = 7.5 Hz, 2H), 7.39 (t, *J* = 7.3 Hz, 1H), 7.16 (d, *J* = 9.1 Hz, 1H), 3.56 (s, 1H), 1.70 (d, *J* = 4.8 Hz, 6H), 1.28 (s, 9H).

^{13}C NMR (100 MHz, $CDCl_3$) δ 159.30, 141.59, 139.52, 133.82, 129.91, 128.80, 128.56, 127.50, 127.25, 127.13, 123.14, 122.58, 118.55, 103.52, 101.93, 78.76, 55.95, 50.14, 31.62, 29.91, 22.39.

HRMS (ESI) m/z Calcd for $[C_{25}H_{27}NNaO_2S, M + Na]^+$: 428.1655, Found: 428.1650.

Optical Rotation: $[\alpha]_D^{25}$= 90.2° (*c* = 1.0, DCM).

HPLC analysis: Chiralcel IA-H (Hexane/*i*-PrOH = 85:15, flow rate = 1.0 mL/min, wave length = 254 nm), t_R = 4.197 min (minor), t_R = 6.273 min (major).

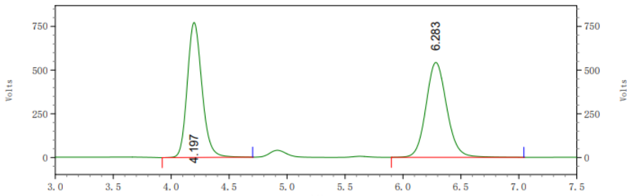

UV-WL1 254 nm

RetTime (min)	Width (min)	Height (Volts)	Area	Area (%)
4.197	0.78	3088904	27260510	50.168
6.283	1.14	2171741	27078184	49.832

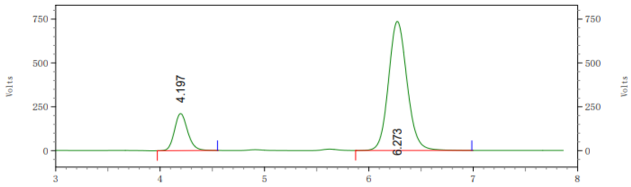

UV-WL1 254 nm

RetTime (min)	Width (min)	Height (Volts)	Area	Area (%)
4.197	0.58	844794	7411338	16.903
6.273	1.11	2943418	36435306	83.097

(aS,S)-4-bromo-1-(tert-butyl)-5-(2-hydroxy-7-phenylnaphthalen-1-yl)-3,3-dimethyl-3H-1λ^6-isothiazole 1-oxide (2g)

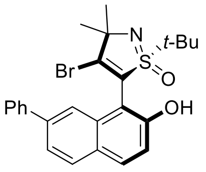

2g

Appearance: white solid.

Yield: 37%, 53 mg. **ee** = 90%.

^{1}H NMR (400 MHz, $CDCl_3$) δ 9.04 (s, 1H), 7.92 – 7.85 (m, 2H), 7.80 – 7.70 (m, 1H), 7.67 (s, 1H), 7.63 (d, J = 8.1 Hz, 2H), 7.47 (t, J = 7.5 Hz, 2H), 7.38 (t, J = 7.3 Hz, 1H), 7.24 (d, J = 8.7 Hz, 1H), 1.73 (s, 3H), 1.63 (s, 3H), 1.35 (s, 9H).

^{13}C NMR (100 MHz, $CDCl_3$) δ 156.77, 154.73, 140.98, 139.78, 134.25, 132.62, 132.01, 130.67, 130.23, 129.56, 129.00, 128.21, 127.59, 127.02, 123.53, 123.37, 121.53, 120.14, 108.36, 63.14, 30.48, 29.05, 24.32.

HRMS (ESI) m/z Calcd for [$C_{25}H_{26}BrNNaO_2S$, M + Na]$^+$: 506.0760, Found: 506.0767.

Optical Rotation: $[\alpha]_D^{25}$= 150.9° (c = 1.0, DCM).

HPLC analysis: Chiralcel AD-H (Hexane/i-PrOH = 95:5, flow rate = 1.0 mL/min, wave length = 254 nm), t_R = 8.560 min (major), t_R = 13.413 min (minor).

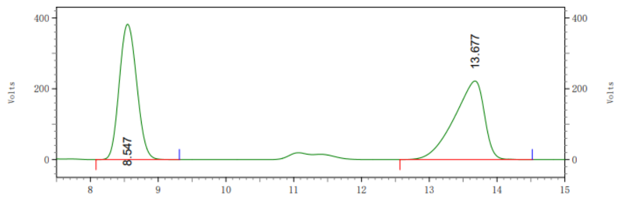

UV-WL1 254 nm

RetTime (min)	Width (min)	Height (Volts)	Area	Area (%)
8.547	1.23	1528303	27456937	49.939
13.677	1.95	887689	27524330	50.061

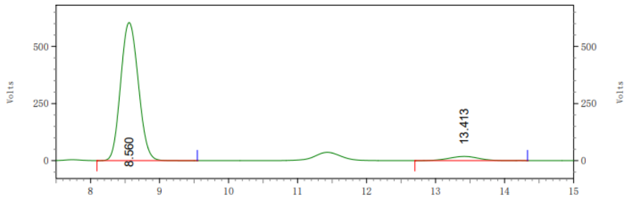

UV-WL1 254 nm

RetTime (min)	Width (min)	Height (Volts)	Area	Area (%)
8.560	1.45	2418588	43098480	95.101
13.413	1.63	73458	2219968	4.899

(S)-N-(4-(7-bromo-2-hydroxynaphthalen-1-yl)-2-methylbut-3-yn-2-yl)-2-methylpropane-2-sulfinamide (1h)

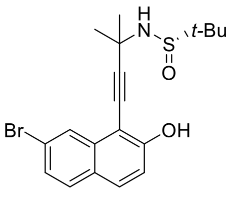

1h

Appearance: white solid.

Yield: 46%, 56 mg. **ee** = 86%.

^{1}H NMR (400 MHz, $CDCl_3$) δ 9.54 (s, 1H), 8.12 (s, 1H), 7.62 (d, J = 9.0 Hz, 1H), 7.56 (d, J = 8.6 Hz, 1H), 7.37 (dd, J = 8.6, 1.7 Hz, 1H), 7.14 (d, J = 8.9 Hz, 1H), 3.55 (s, 1H), 1.70 (s, 6H), 1.27 (s, 9H).

^{13}C NMR (100 MHz, $CDCl_3$) δ 159.61, 134.78, 130.02, 129.64, 126.63, 126.26, 121.35, 118.99, 103.52, 100.97, 78.13, 55.96, 50.06, 31.41, 29.76, 22.33.

HRMS (ESI) m/z Calcd for [$C_{19}H_{22}BrNNaO_2S$, M + Na]$^+$: 430.0447, Found: 430.0442.

Optical Rotation: $[\alpha]_D^{25}$= 67.1° (c = 1.0, DCM).

HPLC analysis: Chiralcel IA-H (Hexane/i-PrOH = 85:15, flow rate = 1.0 mL/min, wave length = 254 nm), t_R = 3.880 min (minor), t_R = 6.830 min (major).

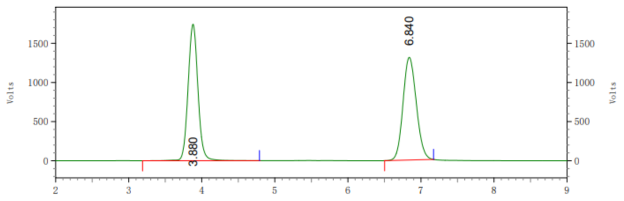

UV-WL1 254 nm

RetTime (min)	Width (min)	Height (Volts)	Area	Area (%)
3.880	1.60	6972153	65410760	49.635
6.840	0.67	5246355	66371668	50.365

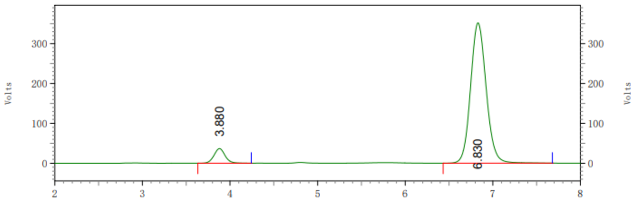

UV-WL1 254 nm

RetTime (min)	Width (min)	Height (Volts)	Area	Area (%)
3.880	0.61	146070	1285592	6.874
6.830	1.25	1407692	17416799	93.126

(*aS,S*)-4-bromo-5-(7-bromo-2-hydroxynaphthalen-1-yl)-1-(tert-butyl)-3,3-dimethyl-3H-1λ[6]-isothiazole 1-oxide (2h)

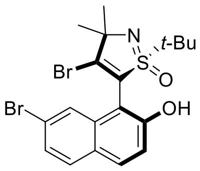

2h

Appearance: white solid.

Yield: 43%, 62.5 mg. **ee** = 93%.

^{1}H NMR (400 MHz, $CDCl_3$) δ 9.10 (s, 1H), 7.80 (d, *J* = 8.9 Hz, 1H), 7.70 (d, *J* = 8.6 Hz, 1H), 7.58 (d, *J* = 1.9 Hz, 1H), 7.46 (dd, *J* = 8.5, 1.8 Hz, 1H), 7.25 (d, *J* = 9.0 Hz, 1H), 1.71 (s, 3H), 1.63 (s, 3H), 1.35 (s, 9H).

^{13}C NMR (100 MHz, $CDCl_3$) δ 157.41, 155.15, 132.12, 131.83, 130.58, 129.61, 127.42, 127.02, 125.87, 121.88, 120.66, 107.59, 63.23, 30.58, 28.99, 24.27.

HRMS (ESI) m/z Calcd for $[C_{19}H_{21}Br_2NNaO_2S, M + Na]^+$: 507.9552, Found: 507.9550.

Optical Rotation: $[\alpha]_D^{25}$= 120.0° (*c* = 1.0, DCM).

HPLC analysis: Chiralcel IA-H (Hexane/*i*-PrOH = 98:2, flow rate = 1.0 mL/min, wave length = 254 nm), t_R = 11.500 min (major), t_R = 12.583 min (minor).

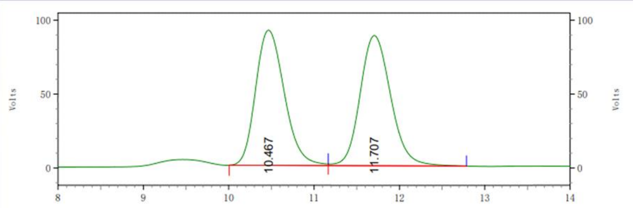

UV-WL1 254 nm

RetTime (min)	Width (min)	Height (Volts)	Area	Area (%)
10.467	1.16	365976	8562274	49.339
11.707	1.62	352464	8791755	50.661

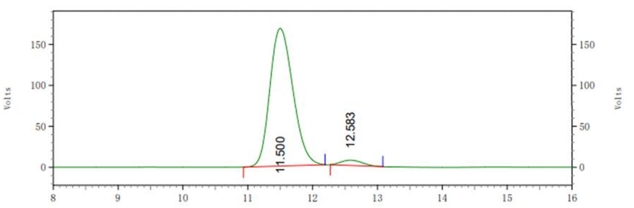

UV-WL1 254 nm

RetTime (min)	Width (min)	Height (Volts)	Area	Area (%)
11.500	1.26	672078	16633634	96.622
12.583	0.81	25327	581541	3.378

(*S*)-*N*-(4-(6-hydroxyquinolin-5-yl)-2-methylbut-3-yn-2-yl)-2-methylpropane-2-sulfinamide (1i)

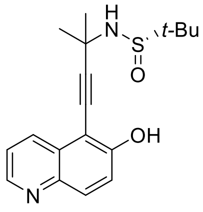

1i

Appearance: white solid.

Yield: 43%, 42.6 mg. **ee** = 97%.

^{1}H NMR (400 MHz, $CDCl_3$) δ 9.62 (s, 1H), 8.73 (dd, *J* = 4.0, 1.6 Hz, 1H), 8.31 (d, *J* = 8.3 Hz, 1H), 7.94 (d, *J* = 9.2 Hz, 1H), 7.42 – 7.30 (m, 2H), 3.63 (s, 1H), 1.68 (d, *J* = 3.3 Hz, 6H), 1.28 (s, 9H).

^{13}C NMR (100 MHz, $CDCl_3$) δ 159.07, 147.48, 143.32, 132.71, 131.56, 128.66, 122.23, 121.49, 103.40, 101.30, 77.89, 56.02, 50.04, 31.36, 29.81, 22.35.

HRMS (ESI) m/z Calcd for $[C_{18}H_{22}N_2NaO_2S, M + Na]^+$: 353.1294, Found: 353.1299.

Optical Rotation: $[\alpha]_D^{25}$= 45.8° (*c* = 1.0, DCM).

HPLC analysis: Chiralcel OD-H (Hexane/*i*-PrOH = 90:10, flow rate = 1.0 mL/min, wave length = 254 nm), t_R = 5.663 min (minor), t_R = 9.177 min (major).

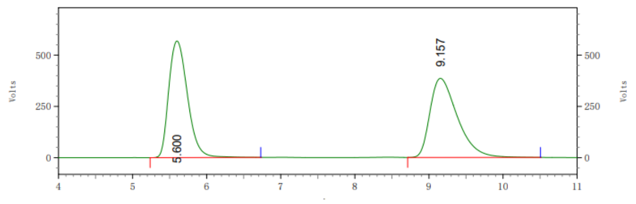

UV-WL1 254 nm

RetTime (min)	Width (min)	Height (Volts)	Area	Area (%)
5.600	1.49	2272323	39895018	50.170
9.157	1.79	1541894	39625082	49.830

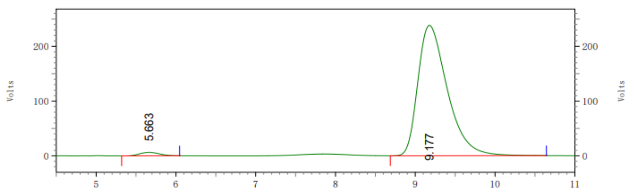

UV-WL1 254 nm

RetTime (min)	Width (min)	Height (Volts)	Area	Area (%)
5.663	0.73	24938	433388	1.746
9.177	1.96	952312	24382050	98.254

(*aS,S*)-4-bromo-1-(tert-butyl)-5-(6-hydroxyquinolin-5-yl)-3,3-dimethyl-3H-1λ⁶-isothiazole 1-oxide (2i)

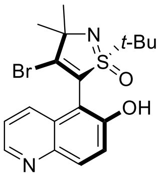

2i

Appearance: white solid.

Yield: 47%, 57.5 mg. **ee** = 93%.

^{1}H NMR (400 MHz, $CDCl_3$) δ 9.20 (s, 1H), 8.83 (d, *J* = 3.0 Hz, 1H), 8.12 (d, *J* = 9.2 Hz, 1H), 7.76 (d, *J* = 8.4 Hz, 1H), 7.48 (d, *J* = 9.2 Hz, 1H), 7.43 (dd, *J* = 8.6, 4.2 Hz, 1H), 1.72 (s, 3H), 1.62 (s, 3H), 1.34 (s, 9H).

^{13}C NMR (100 MHz, $CDCl_3$) δ 157.27, 154.62, 147.83, 144.38, 133.64, 131.51, 129.22, 125.64, 123.92, 121.83, 107.77, 63.14, 30.58, 29.04, 24.26.

HRMS (ESI) m/z Calcd for $[C_{18}H_{21}BrN_2NaO_2S, M + Na]^+$: 431.0399, Found: 431.0393.

Optical Rotation: $[\alpha]_D^{25}$= 190.9° (*c* = 1.0, DCM).

HPLC analysis: Chiralcel IA-H (Hexane/*i*-PrOH = 90:10, flow rate = 1.0 mL/min, wave length = 254 nm), t_R = 8.887 min (minor), t_R = 9.910 min (major).

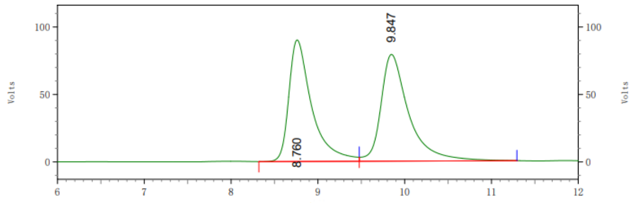

UV-WL1 254 nm

RetTime (min)	Width (min)	Height (Volts)	Area	Area (%)
8.760	1.16	360270	6610462	48.346
9.847	1.82	316387	7062804	51.654

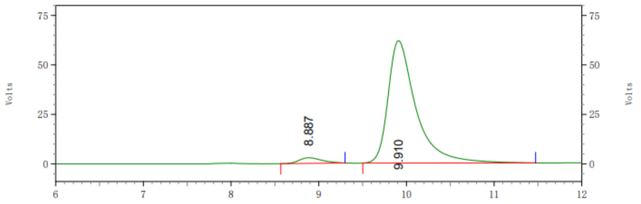

UV-WL1 254 nm

RetTime (min)	Width (min)	Height (Volts)	Area	Area (%)
8.887	0.73	11293	201549	3.556
9.910	1.97	247306	5466311	96.444

(*S*)-*N*-(4-(2-hydroxynaphthalen-1-yl)-2-methylbut-3-yn-2-yl)propane-2-sulfinamide (1j)

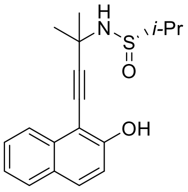

1j

Appearance: white solid.

Yield: 49%, 46 mg. **ee** = 35%.

^{1}H NMR (400 MHz, $CDCl_3$) δ 9.19 (s, 1H), 8.00 (d, *J* = 8.3 Hz, 1H), 7.70 (dd, *J* = 16.9, 8.5 Hz, 2H), 7.47 (t, *J* = 7.5 Hz, 1H), 7.31 (t, *J* = 7.3 Hz, 1H), 7.14 (d, *J* = 8.9 Hz, 1H), 3.97 (s, 1H), 2.87 (p, *J* = 6.7, 5.9 Hz, 1H), 1.69 (d, *J* = 4.6 Hz, 6H), 1.30 (d, *J* = 6.8 Hz, 3H), 1.26 (d, *J* = 6.8 Hz, 3H).

^{13}C NMR (100 MHz, $CDCl_3$) δ 158.96, 133.57, 130.21, 128.06, 127.91, 126.79, 124.45, 123.34, 118.45, 103.28, 101.60, 78.71, 54.40, 50.06, 31.53, 29.91, 15.19, 15.11.

HRMS (ESI) m/z Calcd for $[C_{18}H_{21}NNaO_2S, M + Na]^+$: 338.1185, Found: 338.1181.

Optical Rotation: $[\alpha]_D^{25}$= 4.1° (*c* = 1.0, DCM).

HPLC analysis: Chiralcel OD-H (Hexane/*i*-PrOH = 85:15, flow rate = 1.0 mL/min, wave length = 254 nm), t_R = 4.793 min (minor), t_R = 6.703 min (major).

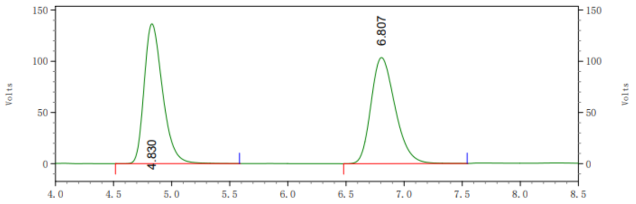

UV-WL1 254 nm

RetTime (min)	Width (min)	Height (Volts)	Area	Area (%)
4.830	1.07	546095	6032249	50.212
6.807	1.06	414239	5981338	49.788

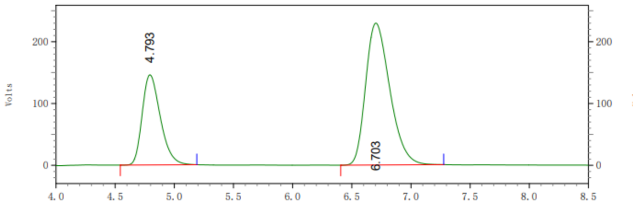

UV-WL1 254 nm

RetTime (min)	Width (min)	Height (Volts)	Area	Area (%)
4.793	0.65	583555	6242092	32.310
6.703	0.87	918163	13077376	67.690

(*aS,S*)-4-bromo-5-(2-hydroxynaphthalen-1-yl)-1-isopropyl-3,3-dimethyl-3H-1λ[6]-isothiazole 1-oxide (2j)

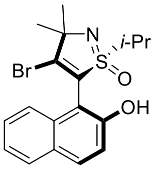

2j

Appearance: white solid.

Yield: 39%, 46 mg. **ee** = 52%.

^{1}H NMR (400 MHz, $CDCl_3$) δ 8.79 (s, 1H), 7.85 (dd, *J* = 8.3, 4.4 Hz, 2H), 7.53 – 7.46 (m, 1H), 7.40 (dd, *J* = 14.5, 7.4 Hz, 2H), 7.29 – 7.24 (m, 1H), 3.42 (p, *J* = 6.9 Hz, 1H), 1.72 (s, 3H), 1.65 (s, 3H), 1.39 (d, *J* = 7.0 Hz, 3H), 1.22 (d, *J* = 6.8 Hz, 3H).

^{13}C NMR (100 MHz, $CDCl_3$) δ 156.79, 154.41, 132.40, 130.87, 130.21, 129.04, 127.35, 123.73, 123.04, 120.19, 106.88, 55.51, 30.54, 29.44, 16.97, 15.77, 14.15.

HRMS (ESI) m/z Calcd for $[C_{18}H_{20}BrNNaO_2S, M + Na]^+$: 416.0290, Found: 416.0290.

Optical Rotation: $[\alpha]_D^{25}$= 2.0° (*c* = 1.0, DCM).

HPLC analysis: Chiralcel IA-H (Hexane/*i*-PrOH = 90:10, flow rate = 1.0 mL/min, wave length = 254 nm), t_R = 6.600 min (minor), t_R = 8.993 min (major).

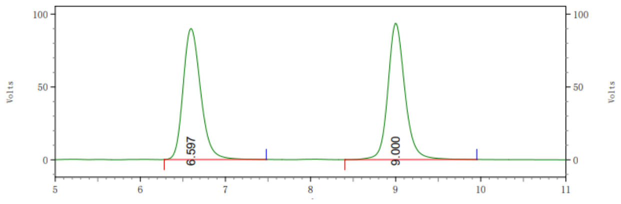

UV-WL1 254 nm

RetTime (min)	Width (min)	Height (Volts)	Area	Area (%)
6.597	1.20	359439	5153419	49.714
9.000	1.55	373929	5212806	50.286

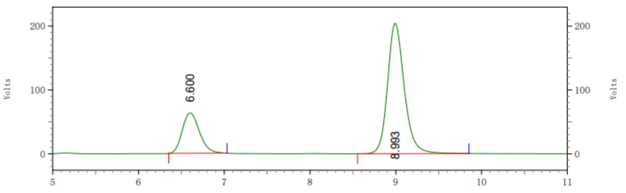

UV-WL1 254 nm

RetTime (min)	Width (min)	Height (Volts)	Area	Area (%)
6.600	0.68	252646	3525023	24.154
8.993	1.30	816064	11068903	75.846

(*S*)-*N*-(4-(2-hydroxynaphthalen-1-yl)-2-methylbut-3-yn-2-yl)benzenesulfinamide (1k)

Appearance: white solid.

Yield: 48%, 50.3 mg. **ee** = 74%.

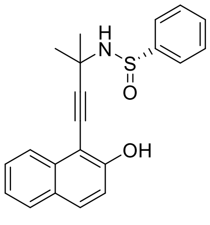

1k

^{1}H NMR (400 MHz, $CDCl_3$) δ 8.97 (s, 1H), 8.02 (d, *J* = 8.3 Hz, 1H), 7.81 – 7.75 (m, 2H), 7.73 (d, *J* = 8.1 Hz, 1H), 7.70 (d, *J* = 8.9 Hz, 1H), 7.55 – 7.45 (m, 4H), 7.32 (t, *J* = 7.5 Hz, 1H), 7.18 (d, *J* = 8.9 Hz, 1H), 4.37 (s, 1H), 1.91 (s, 3H), 1.70 (s, 3H).

^{13}C NMR (100 MHz, $CDCl_3$) δ 158.95, 144.41, 133.56, 131.26, 130.31, 128.95, 128.09, 127.97, 126.85, 125.57, 124.47, 123.42, 118.25, 103.34, 101.65, 78.72, 50.48, 31.73, 29.73.

HRMS (ESI) m/z Calcd for $[C_{21}H_{19}NNaO_2S, M + Na]^+$: 372.1029, Found:. 372.1026

Optical Rotation: $[\alpha]_D^{25}$= 118.2° (*c* =1.0, DCM).

HPLC analysis: Chiralcel AD-H (Hexane/*i*-PrOH = 85:15, flow rate = 1.0 mL/min, wave length = 254 nm), t_R = 7.000 min (minor), t_R = 7.903 min (major).

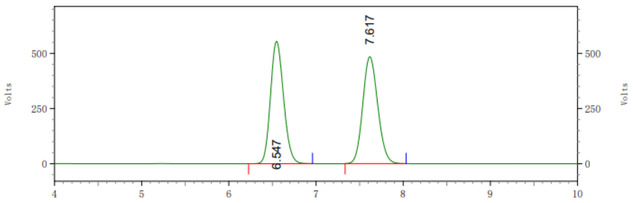

UV-WL1 254 nm

RetTime (min)	Width (min)	Height (Volts)	Area	Area (%)
6.547	0.73	2216420	23093898	49.922
7.617	0.70	1937620	23165857	50.078

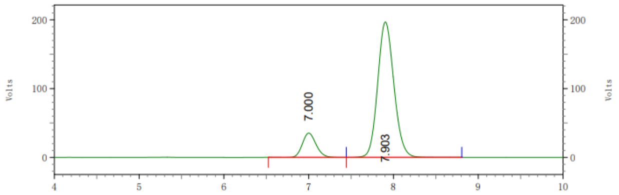

UV-WL1 254 nm

RetTime (min)	Width (min)	Height (Volts)	Area	Area (%)
7.000	0.92	140930	1563951	12.988
7.903	1.36	787412	10477735	87.012

(*aS*, *S*)-4-bromo-5-(2-hydroxynaphthalen-1-yl)-3,3-dimethyl-1-phenyl-3H-isothiazole 1-oxide (2k)

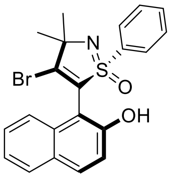

2k

Appearance: white solid.

Yield: 41%, 53 mg. **ee** = 92%.

^{1}H NMR (400 MHz, DMSO-d_6) δ 10.12 (s, 1H), 7.82 (d, J = 8.9 Hz, 1H), 7.68 (d, J = 8.2 Hz, 1H), 7.61 (d, J = 7.8 Hz, 2H), 7.53 (t, J = 7.5 Hz, 1H), 7.38 (t, J = 7.6 Hz, 2H), 7.25 (d, J = 8.9 Hz, 1H), 7.06 (t, J = 7.5 Hz, 1H), 6.82 (t, J = 7.6 Hz, 1H), 6.46 (d, J = 8.4 Hz, 1H), 1.72 (s, 3H), 1.67 (s, 3H).

^{13}C NMR (100 MHz, DMSO-d_6) δ 154.92, 153.37, 139.35, 134.35, 133.49, 132.06, 131.36, 129.51, 129.14, 127.95, 127.23, 126.04, 122.55, 121.98, 118.24, 105.95, 74.33, 30.77, 28.73.

HRMS (ESI) m/z Calcd for [$C_{21}H_{18}BrNNaO_2S$, M + Na]$^+$: 450.0134, Found: 450.0137.

Optical Rotation: $[\alpha]_D^{25}$= 179.0° (c =1.0, DCM).

HPLC analysis: Chiralcel OD-H (Hexane/i-PrOH = 85:15, flow rate = 1.0 mL/min, wave length = 254 nm), t_R = 5.510 min (minor), t_R = 8.270 min (major).

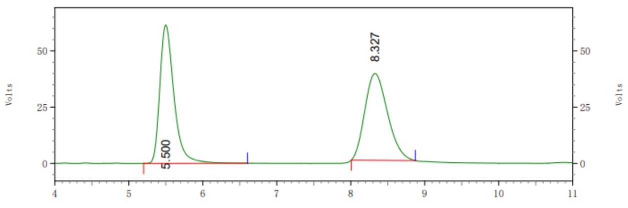

RetTime (min)	Width (min)	Height (Volts)	Area	Area (%)
5.500	1.40	246119	3271941	49.831
8.327	0.87	154321	3294195	50.169

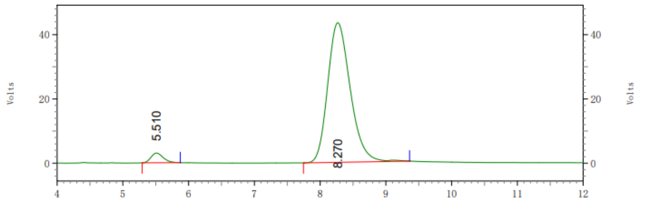

RetTime (min)	Width (min)	Height (Volts)	Area	Area (%)
5.510	0.58	12075	156962	3.655
8.270	1.61	173724	4137742	96.345

(*S*)-*N*-(4-(2-hydroxynaphthalen-1-yl)-2-methylbut-3-yn-2-yl)-4-methylbenzenesulfinamide (1l)

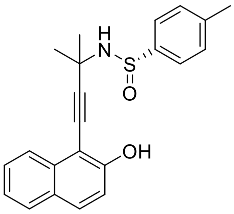

1l

Appearance: white solid.

Yield: 47%, 51 mg. **ee** = 62%.

^{1}H NMR (400 MHz, $CDCl_3$) δ 9.02 (s, 1H), 8.01 (d, J = 8.3 Hz, 1H), 7.71 (dd, J = 13.9, 8.5 Hz, 2H), 7.64 (d, J = 8.2 Hz, 2H), 7.48 (t, J = 7.6 Hz, 1H), 7.31 (t, J = 7.9 Hz, 3H), 7.18 (d, J = 9.0 Hz, 1H), 4.32 (s, 1H), 2.40 (s, 3H), 1.90 (s, 3H), 1.69 (s, 3H).

^{13}C NMR (100 MHz, $CDCl_3$) δ 158.99, 141.74, 141.34, 133.57, 130.25, 129.62, 128.08, 127.96, 126.82, 125.49, 124.48, 123.39, 118.29, 103.40, 101.69, 78.68, 50.36, 31.73, 29.74, 21.32.

HRMS (ESI) m/z Calcd for [$C_{22}H_{21}NNaO_2S$, M + Na]$^+$: 386.1185, Found: 386.1190.

Optical Rotation: $[\alpha]_D^{25}$= 85.9° (c = 1.0, DCM).

HPLC analysis: Chiralcel AD-H (Hexane/i-PrOH = 85:15, flow rate = 1.0 mL/min, wave length = 254 nm), t_R = 9.657 min (minor), t_R = 11.357 min (major).

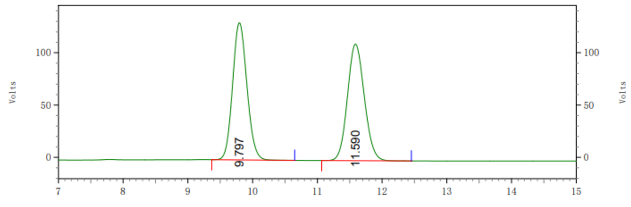

RetTime (min)	Width (min)	Height (Volts)	Area	Area (%)
9.797	1.28	524350	8076873	49.988
11.590	1.39	445548	8080678	50.012

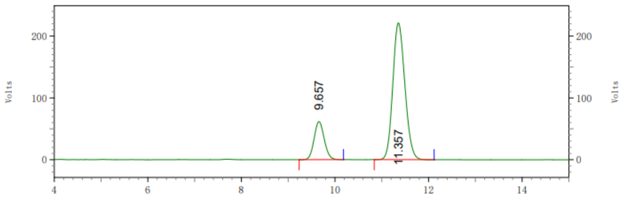

RetTime (min)	Width (min)	Height (Volts)	Area	Area (%)
9.657	0.95	246304	3705883	18.920
11.357	1.28	885001	15881590	81.080

(*aS,S*)-4-bromo-5-(2-hydroxynaphthalen-1-yl)-3,3-dimethyl-1-(p-tolyl)-3H-isothiazole 1-oxide (2l)

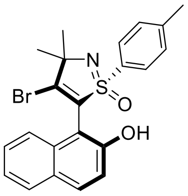

2l

Appearance: white solid.

Yield: 38%, 50 mg. **ee** = 90%.

^{1}H NMR (400 MHz, $CDCl_3$) δ 8.42 (s, 1H), 7.73 (dd, *J* = 11.0, 8.5 Hz, 3H), 7.68 (dd, *J* = 6.3, 3.3 Hz, 1H), 7.25 (s, 1H), 7.24 – 7.19 (m, 2H), 7.12 (d, *J* = 8.0 Hz, 2H), 7.02 – 6.90 (m, 1H), 2.25 (s, 3H), 1.82 (s, 6H).

^{13}C NMR (100 MHz, $CDCl_3$) δ 154.54, 153.91, 145.42, 134.20, 133.74, 133.32, 132.04, 131.63, 130.04, 129.87, 128.67, 128.37, 127.02, 123.48, 122.40, 119.65, 106.80, 30.54, 29.46, 21.49.

HRMS (ESI) m/z Calcd for [C22H20BrNNaO2S, M + Na]$^+$: 464.0290, Found: 464.0295.

Optical Rotation: $[\alpha]_D^{25}$= 70.8° (*c* = 1.0, DCM).

HPLC analysis: Chiralcel OD-H (Hexane/*i*-PrOH = 85:15, flow rate = 1.0 mL/min, wave length = 254 nm), t_R = 5.577 min (minor), t_R = 7.870 min (major).

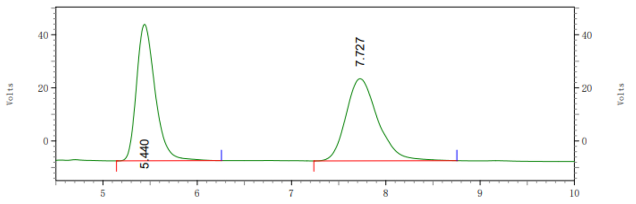

UV-WL1 254 nm

RetTime (min)	Width (min)	Height (Volts)	Area	Area (%)
5.440	1.11	205331	2826031	49.451
7.727	1.52	123512	2888834	50.549

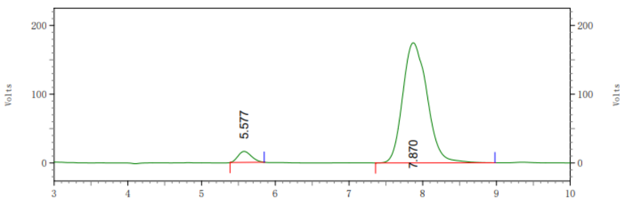

UV-WL1 254 nm

RetTime (min)	Width (min)	Height (Volts)	Area	Area (%)
5.577	0.46	64016	807217	4.776
7.870	1.62	698567	16093988	95.224

(*S*)-4-(tert-butyl)-N-(4-(2-hydroxynaphthalen-1-yl)-2-methylbut-3-yn-2-yl)benzenesulfinamide (1m)

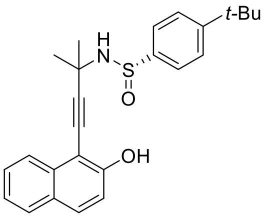

1m

Appearance: white solid.

Yield: 47%, 57mg. **ee** = 70%.

^{1}H NMR (400 MHz, $CDCl_3$) δ 9.04 (s, 1H), 8.02 (d, *J* = 8.3 Hz, 1H), 7.78 – 7.65 (m, 4H), 7.54 (d, *J* = 8.5 Hz, 2H), 7.51 – 7.45 (m, 1H), 7.36 – 7.29 (m, 1H), 7.19 (d, *J* = 8.9 Hz, 1H), 4.31 (s, 1H), 1.91 (s, 3H), 1.70 (s, 3H), 1.35 (s, 9H).

^{13}C NMR (100 MHz, $CDCl_3$) δ 159.00, 158.99, 154.87, 141.24, 133.56, 130.26, 128.08, 127.96, 127.95, 126.82, 125.98, 125.34, 124.48, 123.39, 118.30, 118.29, 103.39, 101.67, 78.67, 50.35, 50.34, 34.94, 34.93, 31.72, 31.18, 31.17, 29.75.

HRMS (ESI) m/z Calcd for [$C_{25}H_{27}NNaO_2S$, M + Na]$^+$: 428.1655, Found: 428.1660.

Optical Rotation: $[\alpha]_D^{25}$= 30.1° (*c* = 1.0, DCM).

HPLC analysis: Chiralcel AD-H (Hexane/*i*-PrOH = 85:15, flow rate = 1.0 mL/min, wave length = 254 nm), t_R = 7.810 min (minor), t_R = 13.637 min (major).

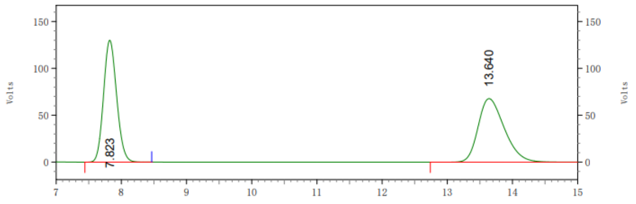

UV-WL1 254 nm

RetTime (min)	Width (min)	Height (Volts)	Area	Area (%)
7.823	1.03	520182	7265207	49.042
13.640	2.57	270899	7549105	50.958

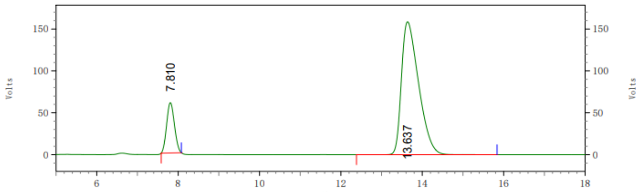

UV-WL1 254 nm

RetTime (min)	Width (min)	Height (Volts)	Area	Area (%)
7.810	0.50	240394	3190285	14.702
13.637	3.45	634413	18510009	85.298

(*aS,S*)-4-bromo-1-(4-(tert-butyl)phenyl)-5-(2-hydroxynaphthalen-1-yl)-3,3-dimethyl-3H-isothiazole 1-oxide (2m)

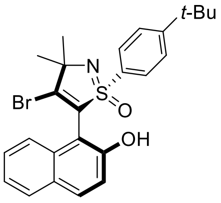

2m

Appearance: white solid.

Yield: 39%, 56.5 mg. **ee** = 91%.

^{1}H NMR (400 MHz, $CDCl_3$) δ 8.29 (s, 1H), 7.90 – 7.83 (m, 1H), 7.75 (t, *J* = 10.6 Hz, 3H), 7.69 (d, *J* = 7.6 Hz, 1H), 7.34 (d, *J* = 8.2 Hz, 2H), 7.19 (dt, *J* = 15.4, 6.7 Hz, 2H), 6.90 (d, *J* = 8.0 Hz, 1H), 1.82 (s, 6H), 1.19 (s, 9H).

^{13}C NMR (100 MHz, $CDCl_3$) δ 167.95, 158.28, 154.65, 153.91, 134.26, 133.72, 133.41, 132.61, 132.02, 131.75, 129.84, 128.66, 128.29, 126.90, 126.23, 123.55, 123.47, 122.44, 119.64, 106.97, 35.12, 30.84, 30.61, 29.39.

HRMS (ESI) m/z Calcd for [$C_{20}H_{17}NNaO_2S$, M + Na]$^+$: 506.0760, Found: 506.0765.

Optical Rotation: $[\alpha]_D^{25}$= 105° (*c* = 1.0, DCM).

HPLC analysis: Chiralcel AD-H (Hexane/*i*-PrOH = 85:15, flow rate = 1.0 mL/min, wave length = 254 nm), t_R = 5.460 min (minor), t_R = 18.550 min (major).

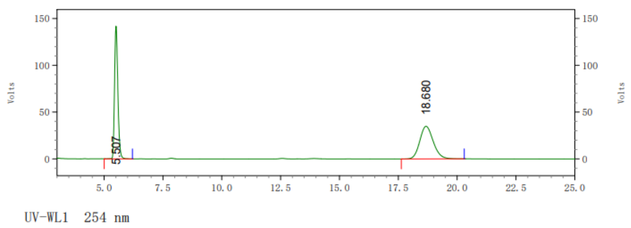

RetTime (min)	Width (min)	Height (Volts)	Area	Area (%)
5.507	1.20	567380	5560294	50.096
18.680	2.68	139438	5538919	49.904

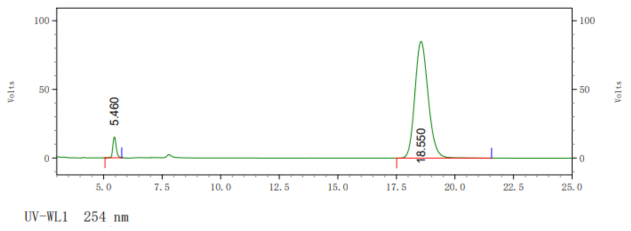

RetTime (min)	Width (min)	Height (Volts)	Area	Area (%)
5.460	0.72	60416	600272	4.282
18.550	4.05	339969	13417138	95.718

(*S*)-4-chloro-N-(4-(2-hydroxynaphthalen-1-yl)-2-methylbut-3-yn-2-yl)benzenesulfinamide (1n)

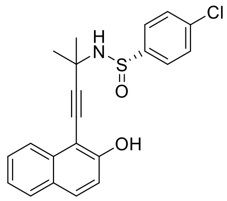

1n

Appearance: white solid.

Yield: 42%, 55 mg. **ee** = 14%.

^{1}H NMR (400 MHz, $CDCl_3$) δ 8.80 (s, 1H), 8.00 (d, *J* = 8.3 Hz, 1H), 7.83 – 7.59 (m, 4H), 7.59 – 7.39 (m, 3H), 7.32 (t, *J* = 7.4 Hz, 1H), 7.16 (d, *J* = 8.9 Hz, 1H), 4.41 (s, 1H), 1.90 (s, 3H), 1.70 (s, 3H).

^{13}C NMR (100 MHz, $CDCl_3$) δ 158.83, 142.86, 137.72, 133.53, 130.41, 129.19, 128.13, 128.00, 127.13, 126.92, 124.44, 123.49, 118.16, 103.18, 101.57, 78.83, 50.61, 31.72, 29.75.

HRMS (ESI) m/z Calcd for [$C_{21}H_{18}ClNNaO_2S$, M + Na]$^+$: 406.0639, Found: 406.0643.

Optical Rotation: $[\alpha]_D^{25}$= 4.5° (*c* = 1.0, DCM).

HPLC analysis: Chiralcel IA-H (Hexane/*i*-PrOH = 85:15, flow rate = 1.0 mL/min, wave length = 254 nm), t_R = 7.930 min (minor), t_R = 12.527 min (major).

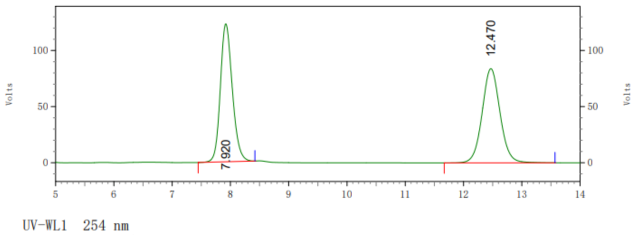

RetTime (min)	Width (min)	Height (Volts)	Area	Area (%)
7.920	0.97	491738	7005502	49.413
12.470	1.90	335722	7171817	50.587

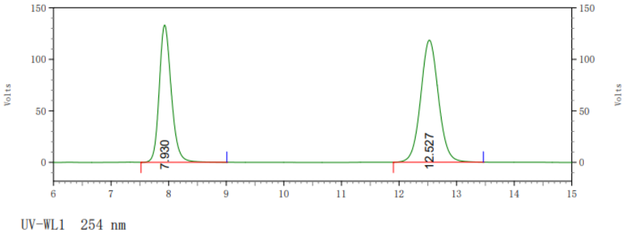

RetTime (min)	Width (min)	Height (Volts)	Area	Area (%)
7.930	1.49	532661	7756825	43.205
12.527	1.56	473508	10196619	56.795

(*aS,S*)-4-bromo-1-(4-chlorophenyl)-5-(2-hydroxynaphthalen-1-yl)-3,3-dimethyl-3H-isothiazole 1-oxide (2n)

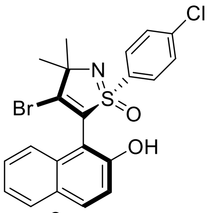

2n

Appearance: white solid.

Yield: 20%, 28 mg. **ee** = 55%.

^{1}H NMR (400 MHz, $CDCl_3$) δ 8.19 (s, 1H), 7.77 (dd, *J* = 8.7, 6.8 Hz, 3H), 7.73 – 7.67 (m, 1H), 7.30 (d, *J* = 8.3 Hz, 2H), 7.28 – 7.18 (m, 3H), 6.93 – 6.86 (m, 1H), 1.82 (s, 6H).

^{13}C NMR (100 MHz, $CDCl_3$) δ 155.55, 153.96, 141.18, 135.22, 133.30, 132.33, 131.51, 131.29, 129.49, 128.73, 128.54, 127.26, 123.70, 122.04, 119.62, 106.35, 30.55, 29.43.

HRMS (ESI) m/z Calcd for $[C_{21}H_{17}BrClNNaO_2S, M + Na]^+$: 483.9744, Found: 483.9740.

Optical Rotation: $[\alpha]_D^{25}$= 9.8° (*c* = 1.0, DCM).

HPLC analysis: Chiralcel OD-H (Hexane/*i*-PrOH = 85:15, flow rate = 1.0 mL/min, wave length = 254 nm), t_R = 4.873 min (minor), t_R = 9.690 min (major).

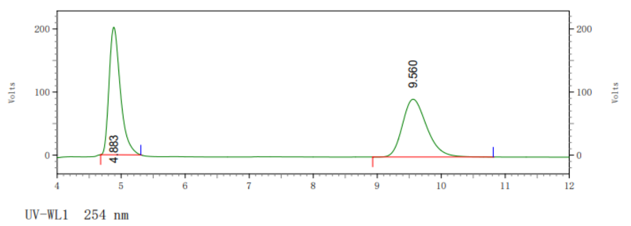

RetTime (min)	Width (min)	Height (Volts)	Area	Area (%)
4.883	0.63	808758	9957865	50.780
9.560	1.88	366175	9651772	49.220

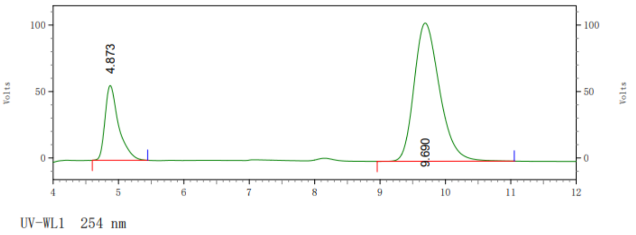

RetTime (min)	Width (min)	Height (Volts)	Area	Area (%)
4.873	0.85	225117	3257253	22.461
9.690	2.10	415729	11244799	77.539

(*S*)-4-fluoro-*N*-(4-(2-hydroxynaphthalen-1-yl)-2-methylbut-3-yn-2-yl)benzenesulfinamide (1o)

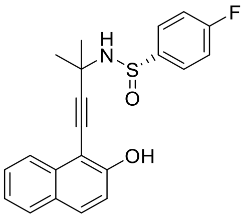

1o

Appearance: white solid.

Yield: 52%, 57 mg. **ee** = 6%.

^{1}H NMR (400 MHz, $CDCl_3$) δ 8.86 (s, 1H), 8.01 (d, *J* = 8.3 Hz, 1H), 7.81 – 7.67 (m, 4H), 7.49 (t, *J* = 7.5 Hz, 1H), 7.32 (t, *J* = 7.4 Hz, 1H), 7.24 – 7.15 (m, 3H), 4.37 (s, 1H), 1.91 (s, 3H), 1.71 (s, 3H).

^{13}C NMR (100 MHz, $CDCl_3$) δ 164.55 (d, *J* = 250.5 Hz), 158.88, 139.88 (d, *J* = 2.8 Hz), 133.52, 130.40, 128.12, 128.00 (d, *J* = 8.6 Hz), 126.90, 124.44, 123.48, 118.20, 116.20 (d, *J* = 22.8 Hz), 103.18, 101.57, 78.80, 50.52, 31.75, 29.72.

HRMS (ESI) m/z Calcd for $[C_{21}H_{18}FNNaO_2S, M + Na]^+$: 390.0934, Found: 390.0930.

Optical Rotation: $[\alpha]_D^{25}$= 2.9° (*c* = 1.0, DCM).

HPLC analysis: Chiralcel OD-H (Hexane/*i*-PrOH = 80:20, flow rate = 1.0 mL/min, wave length = 254 nm), t_R = 4.950 min (minor), t_R = 11.370 min (major).

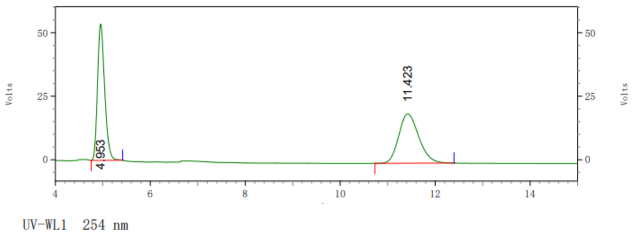

RetTime (min)	Width (min)	Height (Volts)	Area	Area (%)
4.953	0.66	214574	2253276	49.584
11.423	1.66	77829	2291118	50.416

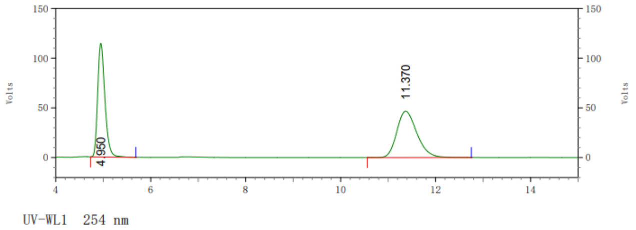

RetTime (min)	Width (min)	Height (Volts)	Area	Area (%)
4.950	0.95	457206	4843761	46.932
11.370	2.19	186354	5477002	53.068

(*aS,S*)-4-bromo-1-(4-fluorophenyl)-5-(2-hydroxynaphthalen-1-yl)-3,3-dimethyl-3H-isothiazole 1-oxide (2o)

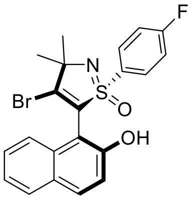

2o

Appearance: peak solid.

Yield: 32%, 43 mg. **ee** = 11%.

^{1}H NMR (400 MHz, $CDCl_3$) δ 8.19 (s, 1H), 7.85 (dd, *J* = 8.5, 5.0 Hz, 2H), 7.78 (d, *J* = 9.0 Hz, 1H), 7.70 (d, *J* = 7.5 Hz, 1H), 7.23 (dd, *J* = 12.0, 6.1 Hz, 3H), 7.01 (t, *J* = 8.4 Hz, 2H), 6.90 (d, *J* = 7.8 Hz, 1H), 1.82 (s, 6H).

^{13}C NMR (100 MHz, $CDCl_3$) δ 166.32 (d, *J* = 256.6 Hz), 155.32, 154.03, 133.53, 132.96 (d, *J* = 9.8 Hz), 132.55 (d, *J* = 2.3 Hz), 132.38, 131.63, 128.81, 128.62, 127.30, 123.78, 122.14, 119.72, 116.65 (d, *J* = 22.7 Hz), 106.54, 77.10, 30.65, 29.51.

HRMS (ESI) m/z Calcd for $[C_{21}H_{17}BrFNNaO_2S, M + Na]^+$: 468.0040, Found: 468.0048.

Optical Rotation: $[\alpha]_D^{25}$= 3.5° (*c* = 1.0, DCM).

HPLC analysis: Chiralcel OD-H (Hexane/*i*-PrOH = 80:20, flow rate = 1.0 mL/min, wave length = 254 nm), t_R = 4.210 min (minor), t_R =6.493 min (major).

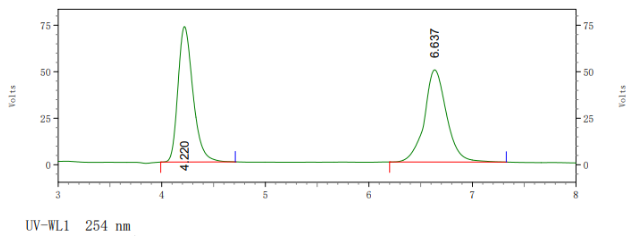

RetTime (min)	Width (min)	Height (Volts)	Area	Area (%)
4.220	0.72	291464	2938088	49.859
6.637	1.13	198293	2954650	50.141

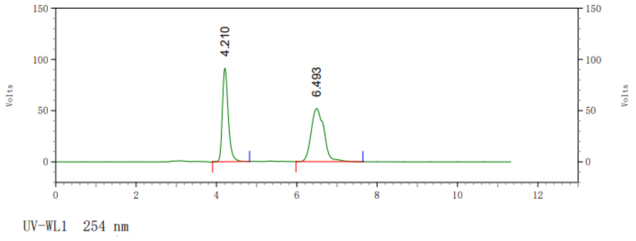

RetTime (min)	Width (min)	Height (Volts)	Area	Area (%)
4.210	0.92	365265	3704522	44.505
6.493	1.67	207244	4619392	55.495

(*S*)-*N*-(4-(6-bromo-2-hydroxynaphthalen-1-yl)-2-methylbut-3-yn-2-yl)benzenesulfinamide (1p)

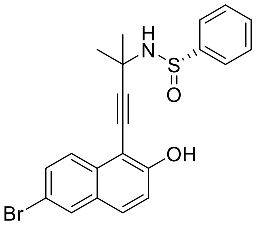

1p

Appearance: white solid.

Yield: 53%, 68 mg. **ee** = 64%.

^{1}H NMR (400 MHz, $CDCl_3$) δ 9.10 (s, 1H), 7.85 (d, *J* = 6.9 Hz, 2H), 7.75 (d, *J* = 4.9 Hz, 2H), 7.59 (d, *J* = 8.9 Hz, 1H), 7.56 – 7.43 (m, 4H), 7.18 (d, *J* = 8.9 Hz, 1H), 4.38 (s, 1H), 1.90 (s, 3H), 1.69 (s, 3H).

^{13}C NMR (100 MHz, $CDCl_3$) δ 159.12, 144.15, 132.04, 131.22, 129.91, 129.19, 128.99, 128.90, 126.31, 125.48, 119.40, 116.94, 103.73, 101.93, 78.15, 50.38, 31.52, 29.60.

HRMS (ESI) m/z Calcd for $[C_{21}H_{18}BrNNaO_2S, M + Na]^+$: 450.0134, Found: 450.0139.

Optical Rotation: $[\alpha]_D^{25}$= 30.4° (*c* = 1.0, DCM).

HPLC analysis: Chiralcel AD-H (Hexane/*i*-PrOH = 85:15, flow rate = 1.0 mL/min, wave length = 254 nm), t_R = 6.837 min (minor), t_R = 10.197 min (major).

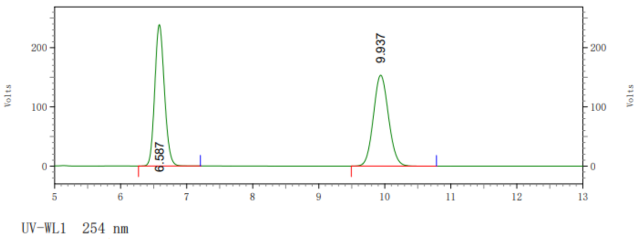

RetTime (min)	Width (min)	Height (Volts)	Area	Area (%)
6.587	0.94	954555	9711409	49.859
9.937	1.29	613649	9766220	50.141

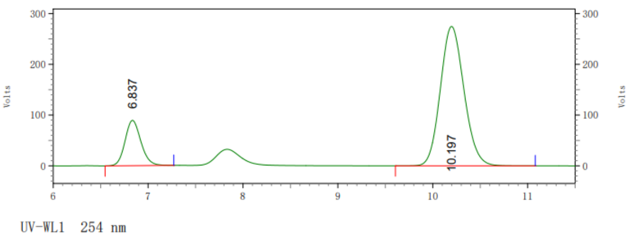

RetTime (min)	Width (min)	Height (Volts)	Area	Area (%)
6.837	0.72	356890	4141632	17.869
10.197	1.47	1098960	19035484	82.131

(*aS,S*)-4-bromo-5-(6-bromo-2-hydroxynaphthalen-1-yl)-3,3-dimethyl-1-phenyl-3H-isothiazole 1-oxide (2p)

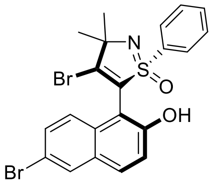

2p

Appearance: white solid.

Yield: 39%, 59 mg. **ee** = 92%.

^{1}H NMR (400 MHz, DMSO-d_6) δ 10.35 (s, 1H), 7.97 (s, 1H), 7.83 (d, *J* = 8.8 Hz, 1H), 7.59 (t, *J* = 9.6 Hz, 3H), 7.42 (t, *J* = 7.0 Hz, 2H), 7.30 (d, *J* = 8.8 Hz, 1H), 6.93 (d, *J* = 8.7 Hz, 1H), 6.38 (d, *J* = 8.8 Hz, 1H), 1.68 (d, *J* = 21.4 Hz, 6H).

^{13}C NMR (100 MHz, DMSO-d_6) δ 155.44, 153.75, 139.12, 133.86, 133.62, 130.73, 130.66, 129.81, 129.50, 129.29, 128.80, 128.47, 124.19, 119.50, 115.28, 106.20, 74.48, 39.52, 30.74, 28.67.

HRMS (ESI) m/z Calcd for $[C_{21}H_{17}Br_2NNaO_2S, M + Na]^+$: 527.9239, Found: 527.9235.

Optical Rotation: $[\alpha]_D^{25}$= 40.6° (*c* = 1.0, DCM).

HPLC analysis: Chiralcel OD-H (Hexane/*i*-PrOH = 85:15, flow rate = 1.0 mL/min, wave length = 254 nm), t_R = 5.123 min (minor), t_R = 7.557 min (major).

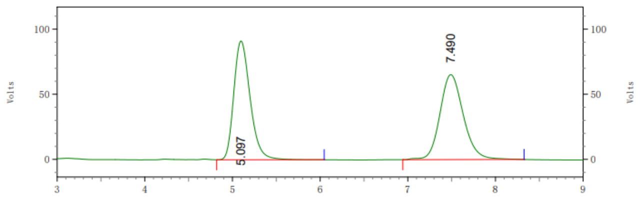

UV-WL1 254 nm

RetTime (min)	Width (min)	Height (Volts)	Area	Area (%)
5.097	1.23	364786	4721863	49.306
7.490	1.38	261151	4854709	50.694

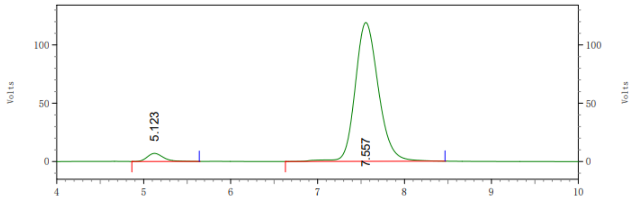

UV-WL1 254 nm

RetTime (min)	Width (min)	Height (Volts)	Area	Area (%)
5.123	0.78	27685	355956	3.863
7.557	1.84	476644	8859723	96.137

(*S*)-*N*-(4-(6-bromo-2-hydroxynaphthalen-1-yl)-2-methylbut-3-yn-2-yl)-4-methylbenzenesulfinamide (1q)

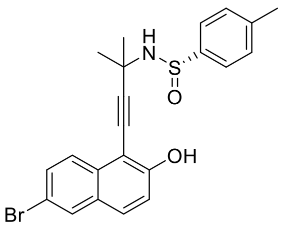

1q

Appearance: white solid.

Yield:47 %, 62 mg. **ee** = 65%.

^{1}H NMR (400 MHz, $CDCl_3$) δ 9.17 (s, 1H), 7.86 (d, *J* = 7.9 Hz, 2H), 7.63 (d, *J* = 7.4 Hz, 2H), 7.59 (d, *J* = 9.0 Hz, 1H), 7.52 (d, *J* = 8.8 Hz, 1H), 7.30 (d, *J* = 7.6 Hz, 2H), 7.18 (d, *J* = 8.8 Hz, 1H), 4.35 (s, 1H), 2.39 (s, 3H), 1.89 (s, 3H), 1.68 (s, 3H).

^{13}C NMR (100 MHz, $CDCl_3$) δ 159.25, 141.81, 141.16, 132.12, 129.96, 129.65, 129.20, 129.06, 126.36, 125.47, 119.48, 116.96, 103.77, 101.96, 78.19, 50.31, 31.63, 29.64, 21.33.

HRMS (ESI) m/z Calcd for $[C_{22}H_{20}BrNNaO_2S, M + Na]^+$: 464.0290, Found: 464.0297.

Optical Rotation: $[\alpha]_D^{25}$= 69.5° (*c* = 1.0, DCM).

HPLC analysis: Chiralcel IA-H (Hexane/*i*-PrOH = 90:10, flow rate = 1.0 mL/min, wave length = 254 nm), t_R = 12.043 min (minor), t_R = 17.410 min (major).

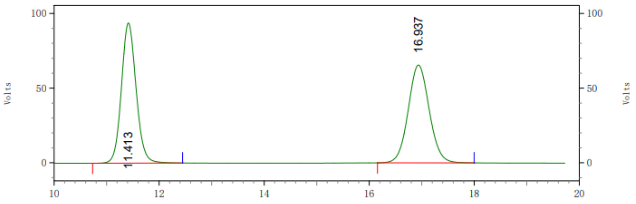

UV-WL1 254 nm

RetTime (min)	Width (min)	Height (Volts)	Area	Area (%)
11.413	1.71	375396	7295780	50.022
16.937	1.84	262048	7289484	49.978

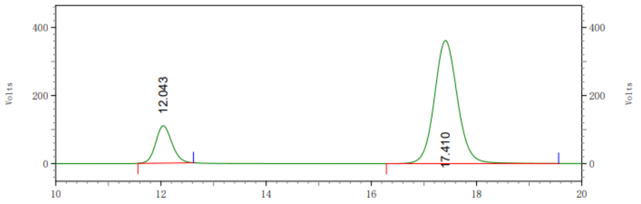

UV-WL1 254 nm

RetTime (min)	Width (min)	Height (Volts)	Area	Area (%)
12.043	1.06	439185	9432749	17.590
17.410	3.27	1446427	44194362	82.410

(*aS,S*)-4-bromo-5-(6-bromo-2-hydroxynaphthalen-1-yl)-3,3-dimethyl-1-(p-tolyl)-3H-isothiazole 1-oxide (2q)

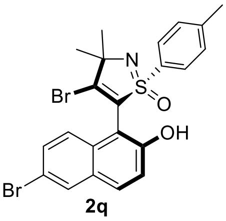

2q

Appearance: white solid.

Yield: 40%, 62 mg. **ee** = 95%.

^{1}H NMR (400 MHz, $CDCl_3$)) δ 8.51 (s, 1H), 7.82 (s, 1H), 7.71 (d, *J* = 8.0 Hz, 2H), 7.65 (d, *J* = 8.9 Hz, 1H), 7.30 – 7.23 (m, 2H), 7.15 (d, *J* = 7.9 Hz, 2H), 6.81 (d, *J* = 8.9 Hz, 1H), 2.29 (s, 3H), 1.81 (s, 6H).

^{13}C NMR (100 MHz, $CDCl_3$) δ 154.84, 154.23, 145.66, 133.36, 133.16, 131.03, 130.36, 130.26, 130.20, 129.98, 129.81, 124.13, 120.90, 117.17, 107.07, 77.32, 76.68, 30.51, 29.42, 21.55.

HRMS (ESI) m/z Calcd for $[C_{22}H_{19}Br_2NNaO_2S, M + Na]^+$: 541.9395, Found: 541.9398.

Optical Rotation: $[\alpha]_D^{25}$= 120.3° (*c* = 1.0, DCM).

HPLC analysis: Chiralcel OD-H (Hexane/*i*-PrOH = 85:15, flow rate = 1.0 mL/min, wave length = 254 nm), t_R = 5.627 min (minor), t_R = 7.717 min (major).

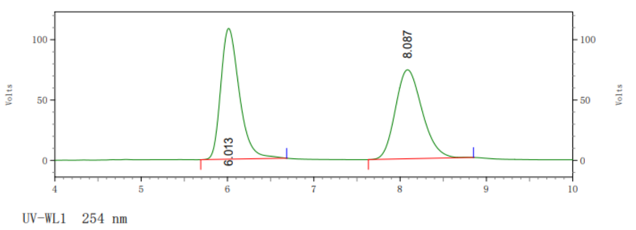

RetTime (min)	Width (min)	Height (Volts)	Area	Area (%)
6.013	1.00	433553	6486410	50.491
8.087	1.22	294996	6360359	49.509

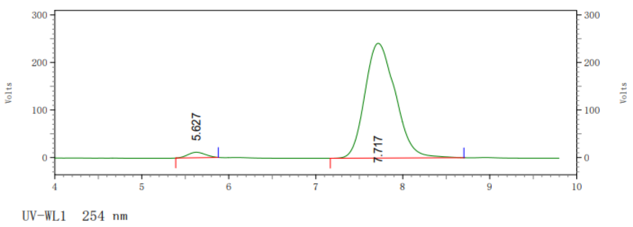

RetTime (min)	Width (min)	Height (Volts)	Area	Area (%)
5.627	0.49	45998	644353	2.660
7.717	1.54	965920	23579260	97.340

(*S*)-*N*-(4-(2-hydroxy-7-methoxynaphthalen-1-yl)-2-methylbut-3-yn-2-yl)-4-methylbenzenesulfinamide (1r)

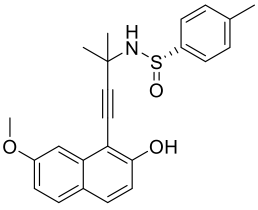

1r

Appearance: white solid.

Yield: 45%, 52.5 mg. **ee** = 64%.

^{1}H NMR (400 MHz, $CDCl_3$) δ 8.90 (s, 1H), 7.61 (s, 4H), 7.30 (d, *J* = 28.0 Hz, 3H), 7.13 – 6.86 (m, 2H), 4.39 (s, 1H), 3.92 (s, 3H), 2.36 (s, 3H), 1.89 (s, 3H), 1.68 (s, 3H).

^{13}C NMR (100 MHz, $CDCl_3$) δ 159.40, 158.72, 141.65, 141.32, 135.06, 130.01, 129.56, 125.44, 123.25, 115.63, 115.00, 104.05, 103.53, 100.92, 78.72, 55.09, 50.34, 31.79, 29.81, 21.26.

HRMS (ESI) m/z Calcd for $[C_{23}H_{23}NNaO_3S, M + Na]^+$: 416.1291, Found: 416.1295.

Optical Rotation: $[\alpha]_D^{25}$= 80.2° (*c* = 1.0, DCM).

HPLC analysis: Chiralcel AD-H (Hexane/*i*-PrOH = 85:15, flow rate = 1.0 mL/min, wave length = 254 nm), t_R = 12.237min (minor), t_R = 13.193 min (major).

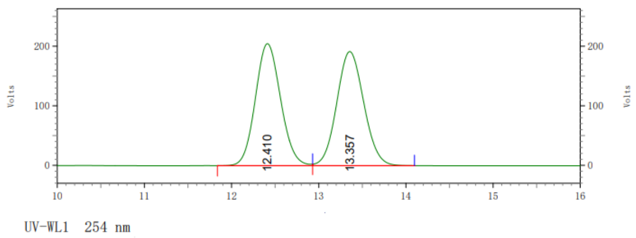

RetTime (min)	Width (min)	Height (Volts)	Area	Area (%)
12.410	1.09	819417	16493089	49.951
13.357	1.17	766302	16525586	50.049

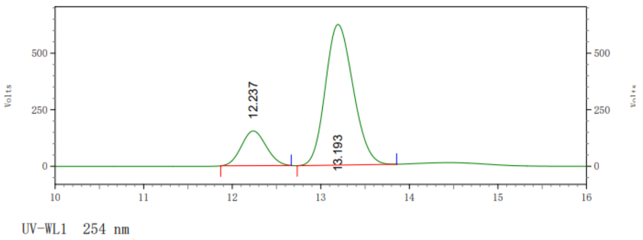

RetTime (min)	Width (min)	Height (Volts)	Area	Area (%)
12.237	0.80	613616	11988719	18.104
13.193	1.12	2485659	54233889	81.896

(*aS,S*)-4-bromo-5-(2-hydroxy-7-methoxynaphthalen-1-yl)-3,3-dimethyl-1-(p-tolyl)-3H-isothiazole 1-oxide (2r)

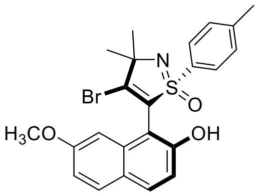

2r

Appearance: white solid.

Yield:38%, 53.6 mg. **ee** = 93%.

^{1}H NMR (400 MHz, $CDCl_3$) δ 8.28 (s, 1H), 7.73 (d, *J* = 8.2 Hz, 2H), 7.66 (d, *J* = 8.9 Hz, 1H), 7.55 (d, *J* = 8.9 Hz, 1H), 7.13 (d, *J* = 8.1 Hz, 2H), 7.09 (d, *J* = 8.9 Hz, 1H), 6.85 (dd, *J* = 8.8, 2.2 Hz, 1H), 6.30 – 6.23 (m, 1H), 3.63 (s, 3H), 2.27 (s, 3H), 1.81 (d, *J* = 6.1 Hz, 6H).

^{13}C NMR (100 MHz, $CDCl_3$) δ 158.60, 154.47, 154.38, 145.48, 134.00, 133.66, 133.15, 131.74, 129.94, 129.85, 129.79, 123.88, 116.90, 115.80, 105.98, 101.39, 54.72, 30.35, 29.50, 21.49.

HRMS (ESI) m/z Calcd for $[C_{23}H_{22}BrNNaO_3S, M + Na]^+$: 494.0396, Found: 494.0390.

Optical Rotation: $[\alpha]_D^{25}$= 44.0° (*c* = 1.0, DCM).

HPLC analysis: Chiralcel OD-H (Hexane/*i*-PrOH = 85:15, flow rate = 1.0 mL/min, wave length = 254 nm), t_R = 6.137 min (minor), t_R = 8.073 min (major).

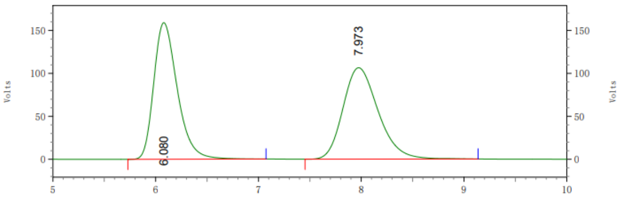

UV-WL1 254 nm

RetTime (min)	Width (min)	Height (Volts)	Area	Area (%)
6.080	1.34	635903	10121766	49.943
7.973	1.68	425614	10144783	50.057

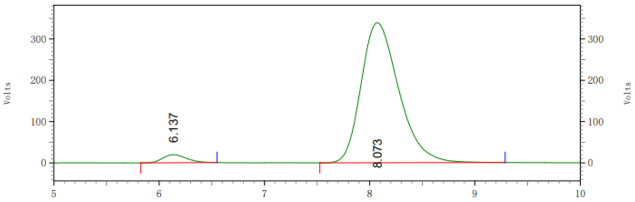

UV-WL1 254 nm

RetTime (min)	Width (min)	Height (Volts)	Area	Area (%)
6.137	0.72	78409	1233038	3.526
8.073	1.76	1356734	33738866	96.474

(*S*)-*N*-(4-(6-hydroxyquinolin-5-yl)-2-methylbut-3-yn-2-yl)benzenesulfinamide (1s)

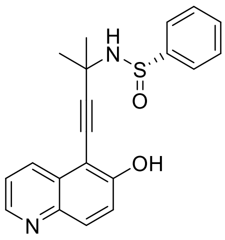

1s

Appearance: white solid.

Yield: 45%, 47 mg. **ee** = 77%.

^{1}H NMR (400 MHz, $CDCl_3$) δ 9.25 (s, 1H), 8.74 (d, *J* = 4.2 Hz, 1H), 8.31 (d, *J* = 8.4 Hz, 1H), 7.96 (d, *J* = 9.3 Hz, 1H), 7.77 (dd, *J* = 6.5, 3.0 Hz, 2H), 7.56 – 7.48 (m, 3H), 7.42 – 7.35 (m, 2H), 4.41 (s, 1H), 1.92 (s, 3H), 1.70 (s, 3H).

^{13}C NMR (100 MHz, $CDCl_3$) δ 159.14, 147.60, 144.17, 143.40, 132.72, 131.71, 131.31, 128.96, 125.54, 122.00, 121.57, 103.60, 101.37, 77.83, 50.40, 31.56, 29.64.

HRMS (ESI) m/z Calcd for $[C_{20}H_{18}N_2NaO_2S, M + Na]^+$: 373.0981, Found: 373.0977.

Optical Rotation: $[\alpha]_D^{25}$= 43.4° (*c* = 1.0, DCM).

HPLC analysis: Chiralcel AD-H (Hexane/*i*-PrOH = 85:15, flow rate = 1.0 mL/min, wave length = 254 nm), t_R = 8.047 min (major), t_R = 9.750 min (minor).

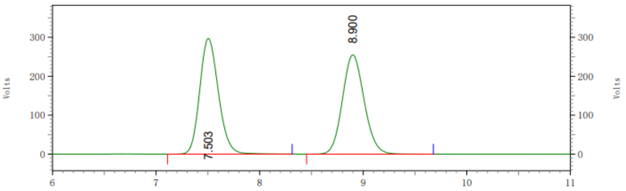

UV-WL1 254 nm

RetTime (min)	Width (min)	Height (Volts)	Area	Area (%)
7.503	1.20	1188887	14930300	50.137
8.900	1.22	1019715	14848777	49.863

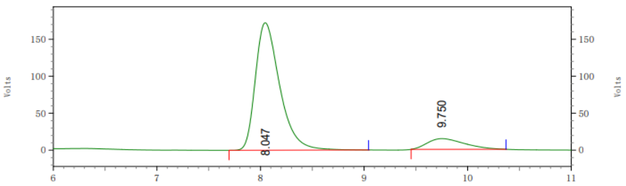

UV-WL1 254 nm

RetTime (min)	Width (min)	Height (Volts)	Area	Area (%)
8.047	1.35	689707	11265082	88.685
9.750	0.92	57773	1437308	11.315

(*aS*,*S*)-4-bromo-5-(6-hydroxyquinolin-5-yl)-3,3-dimethyl-1-phenyl-3H-isothiazole 1-oxide (2s)

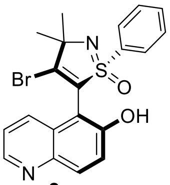

2s

Appearance: white solid.

Yield: 46%, 59 mg. **ee** = 88%.

^{1}H NMR (400 MHz, $CDCl_3$) δ 8.66 (d, *J* = 4.3 Hz, 1H), 8.54 (s, 1H), 8.04 (d, *J* = 9.2 Hz, 1H), 7.84 (d, *J* = 7.7 Hz, 2H), 7.49 (dd, *J* = 8.6, 5.7 Hz, 2H), 7.38 (t, *J* = 7.7 Hz, 2H), 7.22 (d, *J* = 8.3 Hz, 1H), 7.12 (dd, *J* = 8.5, 4.2 Hz, 1H), 1.83 (s, 6H).

^{13}C NMR (100 MHz, $CDCl_3$) δ 155.58, 154.33, 147.77, 143.98, 136.39, 134.39, 133.45, 132.78, 130.50, 129.87, 129.34, 126.73, 123.51, 121.70, 106.32, 30.57, 29.50.

HRMS (ESI) m/z Calcd for $[C_{20}H_{17}BrN_2NaO_2S, M + Na]^+$: 451.0086, Found: 451.0080.

Optical Rotation: $[\alpha]_D^{25}$= 66.3° (*c* = 1.0, DCM).

HPLC analysis: Chiralcel OD-H (Hexane/*i*-PrOH = 85:15, flow rate = 1.0 mL/min, wave length = 254 nm), t_R = 5.717min (minor), t_R =9.113 min (major).

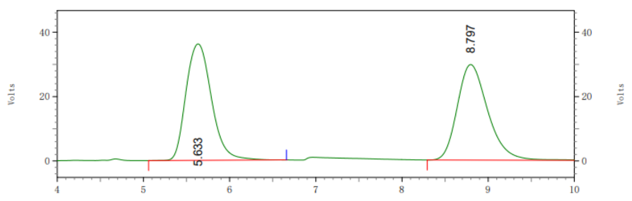

UV-WL1 254 nm

RetTime (min)	Width (min)	Height (Volts)	Area	Area (%)
5.633	1.60	144734	2977433	50.399
8.797	1.94	118775	2930272	49.601

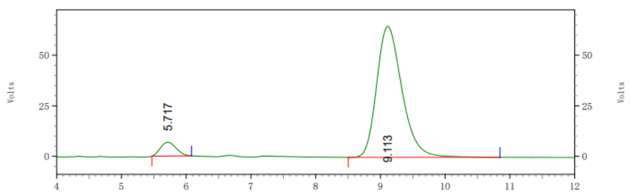

UV-WL1 254 nm

RetTime (min)	Width (min)	Height (Volts)	Area	Area (%)
5.717	0.61	27343	460650	6.138
9.113	2.34	259490	7044694	93.862

(*S*)-*N*-(4-(6-bromo-2-hydroxynaphthalen-1-yl)-2-methylbut-3-yn-2-yl)-4-(tert-butyl)benzenesulfinamide (1t)

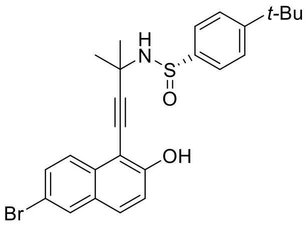

1t

Appearance: white solid.

Yield: 55%, 80 mg. **ee** = 74%.

^{1}H NMR (400 MHz, $CDCl_3$) δ 9.20 (s, 1H), 7.91 – 7.84 (m, 2H), 7.68 (d, *J* = 8.4 Hz, 2H), 7.60 (d, *J* = 9.0 Hz, 1H), 7.56 – 7.49 (m, 3H), 7.19 (d, *J* = 9.0 Hz, 1H), 4.32 (s, 1H), 1.90 (s, 3H), 1.68 (s, 3H), 1.34 (s, 9H).

^{13}C NMR (100 MHz, $CDCl_3$) δ 159.27, 154.97, 141.05, 132.11, 129.97, 129.22, 129.05, 126.37, 126.02, 125.32, 119.50, 116.96, 103.75, 101.94, 78.21, 50.30, 34.96, 31.65, 31.18, 29.64.

HRMS (ESI) m/z Calcd for $[C_{25}H_{26}BrNNaO_2S, M + Na]^+$: 506.0760, Found: 506.0766.

Optical Rotation: $[\alpha]_D^{25}$= 45.4° (*c* = 1.0, DCM).

HPLC analysis: Chiralcel IA-H (Hexane/*i*-PrOH = 85:15, flow rate = 1.0 mL/min, wave length = 254 nm), t_R = 6.713 min (minor), t_R = 11.660 min (major).

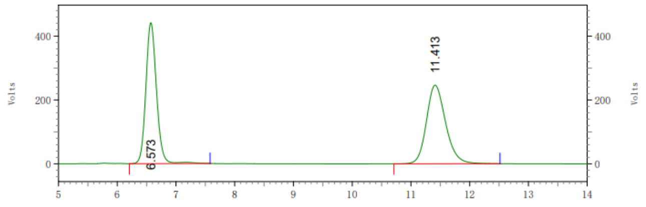

UV-WL1 254 nm

RetTime (min)	Width (min)	Height (Volts)	Area	Area (%)
6.573	1.37	1765033	22253364	49.881
11.413	1.81	984608	22359896	50.119

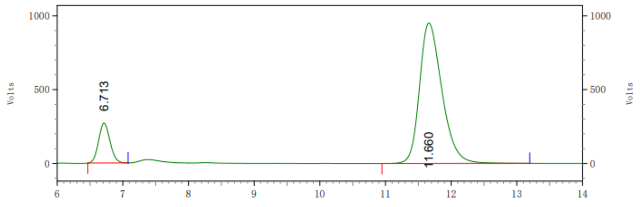

UV-WL1 254 nm

RetTime (min)	Width (min)	Height (Volts)	Area	Area (%)
6.713	0.61	1082588	13071760	12.687
11.660	2.25	3803079	89964662	87.313

(*aS,S*)-4-bromo-5-(6-bromo-2-hydroxynaphthalen-1-yl)-1-(4-(tert-butyl)phenyl)-3,3-dimethyl-3H-isothiazole 1-oxide (2t)

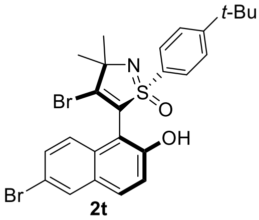

Appearance: white solid.

Yield: 40%, 67.3 mg. **ee** = 97%.

^{1}H NMR (400 MHz, $CDCl_3$) δ 8.39 (s, 1H), 7.83 (d, *J* = 2.0 Hz, 1H), 7.71 (d, *J* = 8.3 Hz, 2H), 7.67 (d, *J* = 8.9 Hz, 1H), 7.36 (d, *J* = 8.3 Hz, 2H), 7.31 – 7.25 (m, 1H), 7.21 (dd, *J* = 9.1, 1.9 Hz, 1H), 6.72 (d, *J* = 8.9 Hz, 1H), 1.81 (d, *J* = 2.9 Hz, 6H), 1.21 (s, 9H).

^{13}C NMR (100 MHz, $CDCl_3$) δ 158.52, 154.26, 133.32, 133.28, 130.99, 130.26, 130.07, 129.78, 129.75, 126.33, 124.15, 120.83, 117.08, 107.15, 35.16, 30.86, 30.58, 29.32.

HRMS (ESI) m/z Calcd for $[C_{25}H_{25}Br_2NNaO_2S, M + Na]^+$: 583.9865, Found: 583.9860.

Optical Rotation: $[\alpha]_D^{25}$= 130.2° (*c* = 1.0, DCM).

HPLC analysis: Chiralcel OD-H (Hexane/*i*-PrOH = 85:15, flow rate = 1.0 mL/min, wave length = 254 nm), t_R = 4.770 min (minor), t_R = 5.337 min (major).

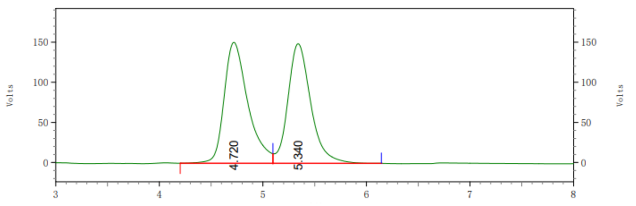

UV-WL1 254 nm

RetTime (min)	Width (min)	Height (Volts)	Area	Area (%)
4.720	0.89	603043	9620342	51.303
5.340	1.05	596421	9131532	48.697

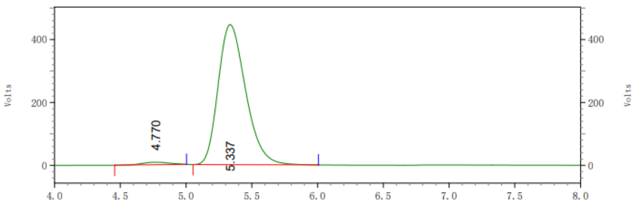

UV-WL1 254 nm

RetTime (min)	Width (min)	Height (Volts)	Area	Area (%)
4.770	0.55	31693	453851	1.720
5.337	0.95	1781156	25930636	98.280

Analytical dates of 2u-2x

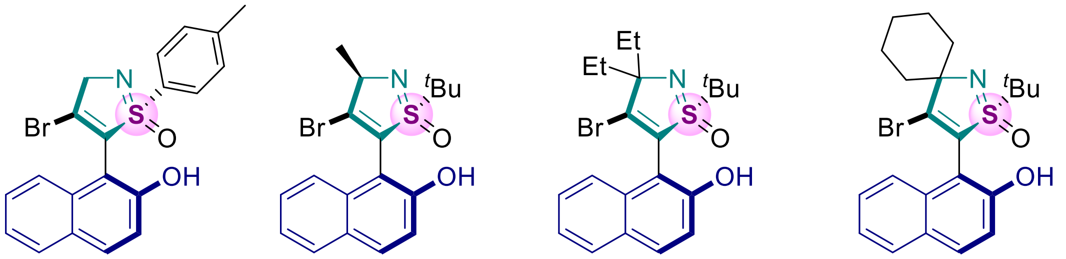

2u	2v	2w	2x
40% ee	80% ee	27% ee	17% ee
d.r. > 20:1	d.r. > 20:1	d.r. > 20:1	d.r. > 20:1
40% yield	45% yield	25% yield	25% yield
45% conv.	48% conv.	28% conv.	29% conv.
S = 3	S = 20	S = 2	S = 1.5

(S)-N-(3-(2-hydroxynaphthalen-1-yl)prop-2-yn-1-yl)-4-methylbenzenesulfinamide(1u)

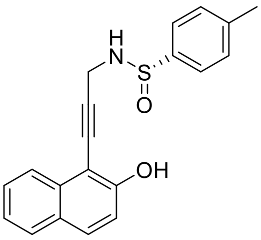

1u

Appearance: white solid.

Yield: 47%, 47 mg. **ee** = 33%.

^{1}H NMR (400 MHz, $CDCl_3$) δ 8.21 (s, 1H), 7.92 (d, *J* = 8.3 Hz, 1H), 7.70 (t, *J* = 9.6 Hz, 2H), 7.64 (d, *J* = 8.0 Hz, 2H), 7.43 (t, *J* = 7.6 Hz, 1H), 7.30 (t, *J* = 8.6 Hz, 3H), 7.17 (d, *J* = 9.0 Hz, 1H), 4.90 (s, 1H), 4.07 (s, 1H), 4.05 (s, 1H), 2.37 (s, 3H).

^{13}C NMR (100 MHz, $CDCl_3$) δ 158.83, 141.86, 139.34, 133.41, 130.35, 129.72, 128.05, 127.95, 126.95, 125.93, 124.52, 123.53, 117.67, 101.84, 97.36, 79.32, 32.95, 21.31.

HRMS (ESI) m/z Calcd for $[C_{20}H_{17}NNaO_2S, M + Na]^+$: 358.0878, Found: 358.0874.

Optical Rotation: $[\alpha]_D^{25}$= 9.05° (*c* = 1.0, DCM).

HPLC analysis: Chiralcel IA-H (Hexane/*i*-PrOH = 85:15, f low rate = 1.0 mL/min, wave length = 254 nm), t_R = 9.310 min (major), t_R = 10.707 min (minor).

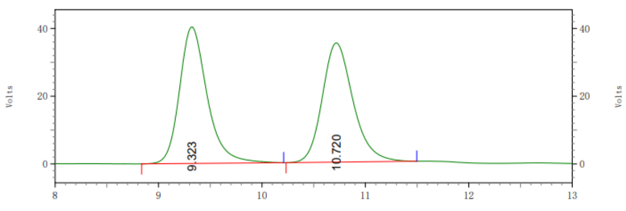

UV-WL1 254 nm

RetTime (min)	Width (min)	Height (Volts)	Area	Area (%)
9.323	1.37	161209	3000773	50.805
10.720	1.26	140751	2905700	49.195

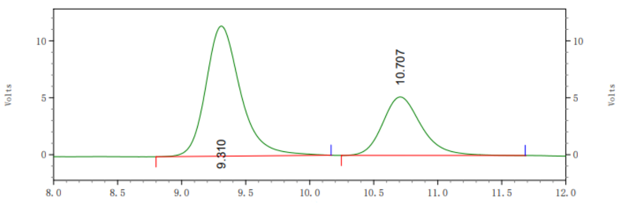

UV-WL1 254 nm

RetTime (min)	Width (min)	Height (Volts)	Area	Area (%)
9.310	1.37	45718	862268	66.599
10.707	1.44	20556	432447	33.401

(*aS,S*)-4-bromo-5-(2-hydroxynaphthalen-1-yl)-1-(*p*-tolyl)-3H-isothiazole 1-oxide(2u)

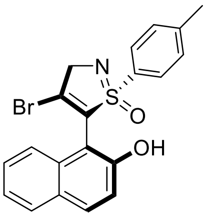

2u

Appearance: Yellow solid.

Yield: 40%, 49 mg. **ee** = 40%.

^{1}H NMR (400 MHz, $CDCl_3$) δ 8.23 (s, 1H), 7.87 (dt, *J* = 7.1, 3.6 Hz, 1H), 7.78 (d, *J* = 8.7 Hz, 1H), 7.72 (d, *J* = 8.3 Hz, 2H), 7.33 – 7.19 (m, 3H), 7.14 (d, *J* = 8.0 Hz, 2H), 7.06 – 6.92 (m, 1H), 5.13 – 4.87 (m, 2H), 2.28 (s, 3H).

^{13}C NMR (100 MHz, $CDCl_3$) δ 167.98, 154.03, 145.67, 143.08, 134.28, 133.72, 132.58, 132.23, 131.67, 130.02, 129.90, 128.67, 128.35, 126.99, 123.56, 122.69, 119.62, 106.16, 66.49, 21.56.

HRMS (ESI) m/z Calcd for $[C_{20}H_{16}BrNNaO_2S, M + Na]^+$: 435.9983, Found: 435.9989.

Optical Rotation: $[\alpha]_D^{25}$= 23.03° (*c* = 1.0, DCM).

HPLC analysis: Chiralcel OD-H (Hexane/*i*-PrOH = 85:15, flow rate = 1.0 mL/min, wave length = 254 nm), t_R = 11.853 min (minor), t_R = 14.923 min (major).

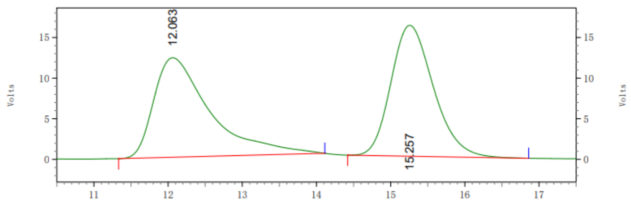

UV-WL1 254 nm

RetTime (min)	Width (min)	Height (Volts)	Area	Area (%)
12.063	2.78	48947	2580270	50.091
15.257	2.44	64368	2570875	49.909

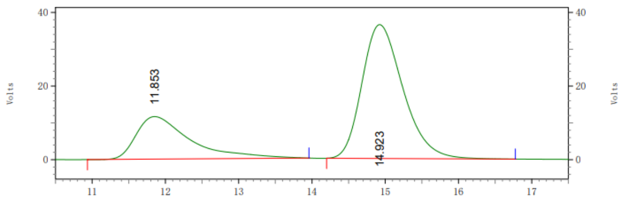

UV-WL1 254 nm

RetTime (min)	Width (min)	Height (Volts)	Area	Area (%)
11.853	3.02	46263	2379034	29.560
14.923	2.58	145451	5669059	70.440

(*S*)-*N*-((*S*)-4-(2-hydroxynaphthalen-1-yl)but-3-yn-2-yl)-2-methylpropane-2-sulfinamide (1v)

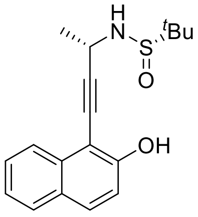

1v

Appearance: white solid.

Yield: 48%, 45 mg. **ee** = 74%.

^{1}H NMR (400 MHz, $CDCl_3$) δ 9.02 (s, 1H), 8.02 (d, *J* = 8.4 Hz, 1H), 7.73 (d, *J* = 8.2 Hz, 1H), 7.69 (d, *J* = 8.9 Hz, 1H), 7.47 (t, *J* = 7.6 Hz, 1H), 7.31 (t, *J* = 7.5 Hz, 1H), 7.15 (dd, *J* = 8.9, 1.7 Hz, 1H), 4.48 (ddt, *J* = 9.8, 7.0, 3.5 Hz, 1H), 3.89 (s, 1H), 1.66 (d, *J* = 6.9 Hz, 3H), 1.27 (s, 9H).

^{13}C NMR (100 MHz, $CDCl_3$) δ 158.77, 133.52, 130.28, 128.07, 127.91, 126.85, 124.48, 123.40, 118.31, 101.61, 100.37, 80.03 , 55.94, 43.06, 22.73, 22.41.

HRMS (ESI) m/z Calcd for $[C_{18}H_{21}NNaO_2S, M + Na]^+$: 338.1191, Found: 338.1185.

Optical Rotation: $[\alpha]_D^{25}$= 33.53° (*c* = 1.0, DCM).

HPLC analysis: Chiralcel IA-H (Hexane/*i*-PrOH = 90:10, flow rate = 1.0 mL/min, wave length = 254 nm), t_R = 7.290 min (minor), t_R = 10.147 min (major).

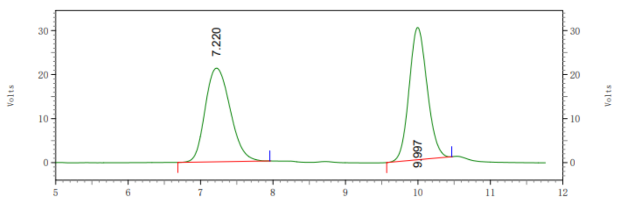

UV-WL1 254 nm

RetTime (min)	Width (min)	Height (Volts)	Area	Area (%)
7.220	1.27	85153	2039454	49.469
9.997	0.90	120315	2083261	50.531

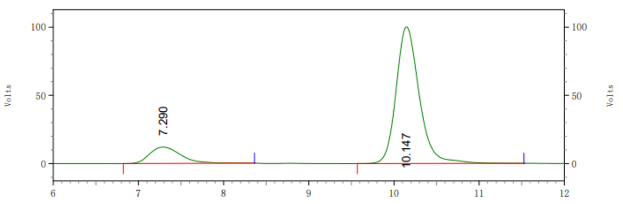

UV-WL1 254 nm

RetTime (min)	Width (min)	Height (Volts)	Area	Area (%)
7.290	1.54	47799	1220671	13.911
10.147	1.96	401016	7554124	86.089

(*aS*, *1S*, *3R*)-4-bromo-1-(tert-butyl)-5-(2-hydroxynaphthalen-1-yl)-3-methyl-3H-1λ^6-isothiazole 1-oxide (2v)

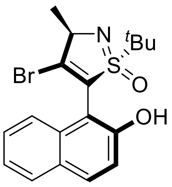

2v

Appearance: white solid.

Yield: 45%, 53 mg. **ee** = 80%.

^{1}H NMR (400 MHz, $CDCl_3$) δ 8.96 (s, 1H), 7.95 – 7.74 (m, 2H), 7.55 – 7.47 (m, 1H), 7.40 (dd, *J* = 16.0, 8.0 Hz, 2H), 7.25 (d, *J* = 8.0 Hz, 1H), 5.01 (q, *J* = 6.9 Hz, 1H), 1.64 (d, *J* = 6.9 Hz, 3H), 1.35 (s, 9H).

^{13}C NMR (100 MHz, $CDCl_3$) δ 154.41, 151.28, 132.37, 131.05, 130.41, 129.07, 128.99, 127.36, 123.67, 123.24, 120.15, 107.85, 71.61, 62.89, 24.21, 21.99.

HRMS (ESI) m/z Calcd for $[C_{18}H_{20}BrNNaO_2S, M + Na]^+$: 416.0296, Found: 416.0289.

Optical Rotation: $[\alpha]_D^{25}$= 45.6° (*c* = 1.0, DCM).

HPLC analysis: Chiralcel IA-H (Hexane/*i*-PrOH = 95:5, flow rate = 1.0 mL/min, wave length = 254 nm), t_R = 10.927 min (major), t_R = 13.483 min (minor).

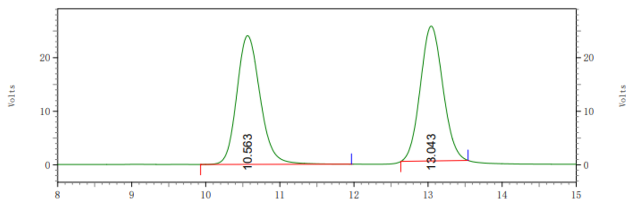

UV-WL1 254 nm

RetTime (min)	Width (min)	Height (Volts)	Area	Area (%)
10.563	2.04	96115	2112561	49.760
13.043	0.91	100654	2132982	50.240

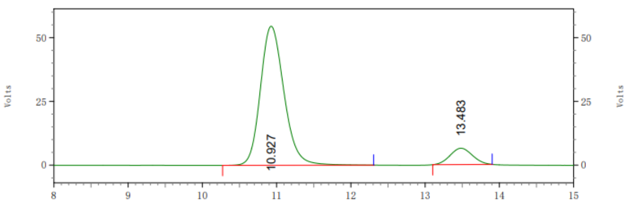

UV-WL1 254 nm

RetTime (min)	Width (min)	Height (Volts)	Area	Area (%)
10.927	2.03	218078	4818416	90.008
13.483	0.80	25546	534915	9.992

(*S*)-*N*-(3-ethyl-1-(2-hydroxynaphthalen-1-yl)pent-1-yn-3-yl)-2-methylpropane-2-sulfinamide(1w)

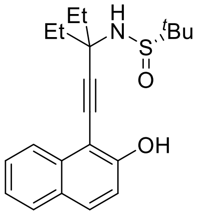

1w

Appearance: white solid.

Yield: 60%, 64 mg. **ee** = 9%.

^{1}H NMR (400 MHz, $CDCl_3$) δ 9.39 (s, 1H), 8.00 (d, *J* = 8.3 Hz, 1H), 7.72 (d, *J* = 8.1 Hz, 1H), 7.68 (d, *J* = 9.0 Hz, 1H), 7.47 (t, *J* = 7.6 Hz, 1H), 7.30 (t, *J* = 7.5 Hz, 1H), 7.15 (d, *J* = 8.9 Hz, 1H), 3.42 (s, 1H), 2.07 (dt, *J* = 14.5, 7.4 Hz, 1H), 1.94 (dp, *J* = 22.2, 7.7 Hz, 2H), 1.79 (dt, *J* = 13.9, 7.2 Hz, 1H), 1.28 (s, 9H), 1.15 (t, *J* = 7.4 Hz, 3H), 1.07 (t, *J* = 7.4 Hz, 3H).

^{13}C NMR (100 MHz, $CDCl_3$) δ 158.96, 133.75, 130.13, 128.09, 127.94, 126.82, 124.45, 123.28, 118.55, 101.78, 80.84, 57.58, 56.14, 32.14, 31.91, 22.58, 8.48, 8.06.

HRMS (ESI) m/z Calcd for $[C_{21}H_{27}NNaO_2S$, M + Na$]^+$: 380.1660, Found: 380.1665.

Optical Rotation: $[\alpha]_D^{25}$= 5.05° (*c* = 1.0, DCM).

HPLC analysis: Chiralcel OD-H (Hexane/*i*-PrOH = 85:15, flow rate = 1.0 mL/min, wave length = 254 nm), t_R = 6.207 min (minor), t_R = 10.893 min (major).

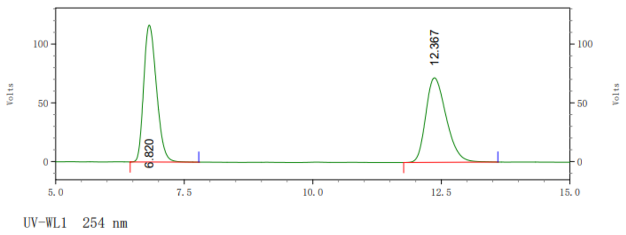

RetTime (min)	Width (min)	Height (Volts)	Area	Area (%)
6.820	1.33	466354	8082580	50.376
12.367	1.83	288051	7962009	49.624

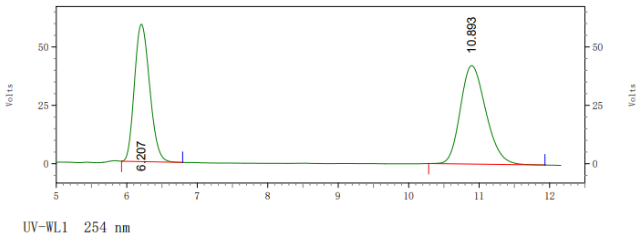

RetTime (min)	Width (min)	Height (Volts)	Area	Area (%)
6.207	0.87	235391	3537805	45.443
10.893	1.65	168689	4247384	54.557

(*aS,S*)-4-bromo-1-(tert-butyl)-3,3-diethyl-5-(2-hydroxynaphthalen-1-yl)-3H-1λ^{6}-isothiazole 1-oxide(2w)

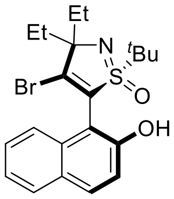

2w

Appearance: white solid.

Yield: 25%, 33 mg. **ee** = 27%.

^{1}H NMR (400 MHz, $CDCl_3$) δ 9.04 (s, 1H), 7.89 – 7.80 (m, 2H), 7.50 (d, *J* = 4.0 Hz, 2H), 7.38 (dt, *J* = 8.1, 4.0 Hz, 1H), 7.25 (d, *J* = 9.2 Hz, 1H), 2.12 (dq, *J* = 12.7, 6.5, 5.6 Hz, 1H), 2.06 – 1.99 (m, 1H), 1.86 (dt, *J* = 14.0, 7.2 Hz, 1H), 1.74 (dt, *J* = 14.6, 7.5 Hz, 1H), 1.37 (s, 9H), 1.20 (t, *J* = 7.4 Hz, 3H), 0.99 (t, *J* = 7.3 Hz, 3H).

^{13}C NMR (100 MHz, $CDCl_3$) δ 155.39, 154.29, 132.13, 131.61, 130.68, 129.05, 127.24, 123.63, 123.49, 120.25, 108.73, 83.83, 63.91, 34.31, 30.64, 24.70, 9.08, 8.11.

HRMS (ESI) m/z Calcd for $[C_{21}H_{26}BrNNaO_2S$, M + Na$]^+$: 458.0765, Found: 458.0760.

Optical Rotation: $[\alpha]_D^{25}$= 17.25° (*c* = 1.0, DCM).

HPLC analysis: Chiralcel IC-H (Hexane/*i*-PrOH = 98.5:1.5, flow rate = 1.0 mL/min, wave length = 254 nm), t_R = 13.870 min (major), t_R = 16.013 min (minor).

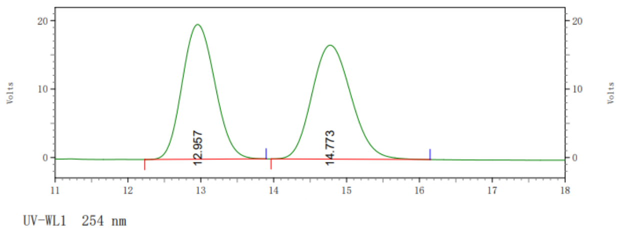

RetTime (min)	Width (min)	Height (Volts)	Area	Area (%)
12.957	1.67	78681	2459979	49.616
14.773	2.18	66526	2498095	50.384

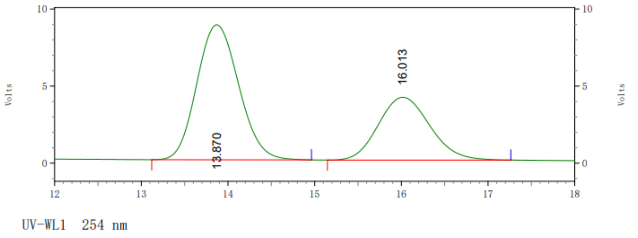

RetTime (min)	Width (min)	Height (Volts)	Area	Area (%)
13.870	1.84	35009	1177467	63.656
16.013	2.12	16289	672272	36.344

(S)-*N*-(1-((2-hydroxynaphthalen-1-yl)ethynyl)cyclohexyl)-2-methylpropane-2-sulfinamide(1x)

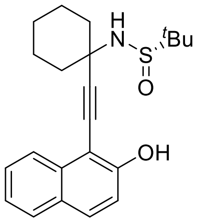

1x

Appearance: white solid.

Yield: 65%, 72 mg. **ee** = 7%.

^{1}H NMR (400 MHz, $CDCl_3$) δ 9.41 (s, 1H), 8.03 (d, *J* = 8.3 Hz, 1H), 7.73 (d, *J* = 8.1 Hz, 1H), 7.68 (d, *J* = 8.9 Hz, 1H), 7.54 – 7.46 (m, 1H), 7.36 – 7.28 (m, 1H), 7.15 (d, *J* = 9.0 Hz, 1H), 3.48 (s, 1H), 2.33 (d, *J* = 12.7 Hz, 1H), 2.02 (d, *J* = 13.3 Hz, 1H), 1.90 (td, *J* = 11.9, 11.3, 3.8 Hz, 1H), 1.80 (dt, *J* = 9.9, 5.3 Hz, 4H), 1.67 (ddd, *J* = 23.7, 12.1, 5.9 Hz, 2H), 1.40 – 1.32 (m, 1H), 1.28 (s, 9H).

^{13}C NMR (100 MHz, $CDCl_3$) δ 158.89, 133.84, 130.14, 128.11, 127.94, 126.87, 124.46, 123.26, 118.54, 102.28, 101.74, 80.65, 55.89, 54.44, 39.64, 38.52, 25.36, 23.02, 22.63, 22.46.

HRMS (ESI) m/z Calcd for $[C_{22}H_{27}NNaO_2S, M + Na]^+$: 392.5128, Found: 392.5135.

Optical Rotation: $[\alpha]_D^{25}$= 3.4° (*c* = 1.0, DCM).

HPLC analysis: Chiralcel OD-H (Hexane/*i*-PrOH = 90:10, flow rate = 1.0 mL/min, wave length = 254 nm), t_R = 5.847 min (minor), t_R = 10.987 min (major).

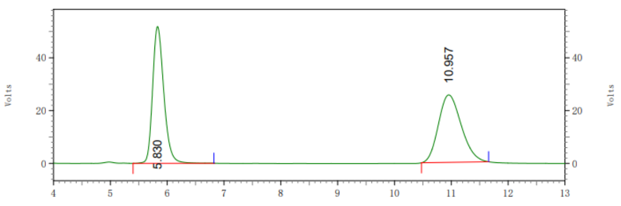

UV-WL1 254 nm

RetTime (min)	Width (min)	Height (Volts)	Area	Area (%)
5.830	1.42	207403	2843825	49.883
10.957	1.18	102249	2857150	50.117

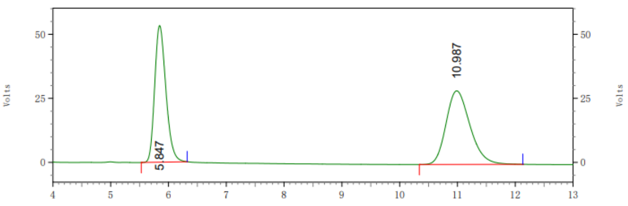

UV-WL1 254 nm

RetTime (min)	Width (min)	Height (Volts)	Area	Area (%)
5.847	0.79	213305	2880399	46.526
10.987	1.79	114933	3310488	53.474

(*aS,S*)-4-bromo-2-(*tert*-butyl)-3-(2-hydroxynaphthalen-1-yl)-2λ^{6}-thia-1-azaspiro[4.5]deca-1,3-diene 2-oxide(2x)

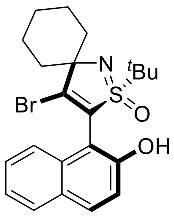

2x

Appearance: white solid.

Yield: 25%, 33.5 mg. **ee** = 17%.

^{1}H NMR (400 MHz, $CDCl_3$) δ 9.10 (s, 1H), 7.88 – 7.78 (m, 2H), 7.52 – 7.42 (m, 2H), 7.37 (t, *J* = 7.0 Hz, 1H), 7.24 (d, *J* = 8.9 Hz, 1H), 2.14 – 1.96 (m, 4H), 1.85 – 1.72 (m, 3H), 1.69 – 1.63 (m, 1H), 1.54 (d, *J* = 8.4 Hz, 1H), 1.32 (s, 9H), 1.25 (dd, *J* = 9.8, 6.0 Hz, 1H).

^{13}C NMR (100 MHz, $CDCl_3$) δ 157.22, 154.26, 132.05, 130.54, 129.42, 129.00, 127.17, 123.59, 123.57, 120.17, 108.53, 80.07, 62.88, 38.74, 37.12, 25.28, 24.33, 22.93, 22.11.

HRMS (ESI) m/z Calcd for $[C_{22}H_{26}BrNNaO_2S, M + Na]^+$: 470.0765, Found: 470.0770.

Optical Rotation: $[\alpha]_D^{25}$= 42.1° (*c* = 1.0, DCM).

HPLC analysis: Chiralcel OD-H (Hexane/*i*-PrOH = 98:2, flow rate = 1.0 mL/min, wave length = 254 nm), t_R = 4.580 min (major), t_R = 5.593 min (minor).

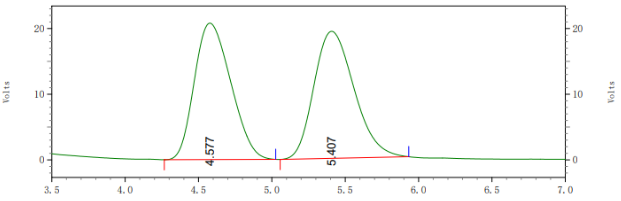

UV-WL1 254 nm

RetTime (min)	Width (min)	Height (Volts)	Area	Area (%)
4.577	0.76	83124	1433598	49.293
5.407	0.88	77387	1474750	50.707

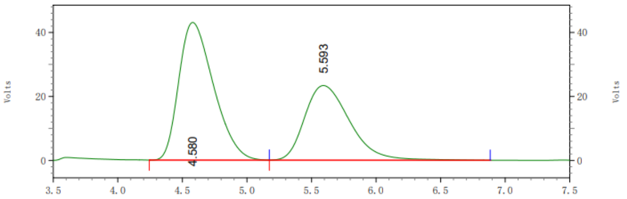

UV-WL1 254 nm

RetTime (min)	Width (min)	Height (Volts)	Area	Area (%)
4.580	0.93	172204	3224640	58.790
5.593	1.71	93328	2260364	41.210

Preparative gram-scale reaction for 1k

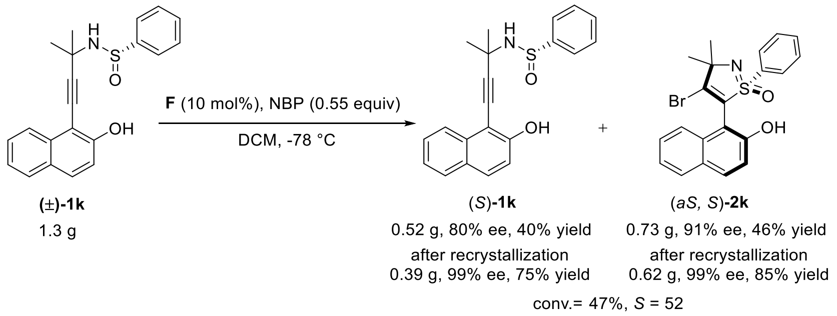

A flame-dried Schlenk tube equipped with a magnetic stirring bar, was charged with (±)-**1k** (1.3 g, 3.72 mmol, 1.0 equiv), and catalyst **F** (10 mol%), DCM (150.0 mL) was injected into the tube at -78 ºC. After stirring for 10min, the NBP (464 mg, 2.05 mmol, 0.55 equiv) was added into the mixture, after the reaction was completed, monitored by TLC. The reaction was quenched with saturated ammonium chloride solution, and extracted with DCM (3×50 mL), all organic layer was remove under reduced pressure, and the residue was purified by column chromatography (PE:EA= 4:1) to afford (*S*)-**1k** (0.52g, 80% ee, 40% yield) and the product (*aS, S*)-**2k** (0.73g, 91% ee, 46% yield); after recrystallization (Both (*aS, S*)-**2k** and (*S*)-**1k** recrystallization conditions were DCM/hexane= 1:1 under room temperature) to afford the desired (*S*)-**1k** (0.39 g, 99% ee, 75% yield) and (*aS, S*)-**2k** (0.62 g, 99% ee, 85% yield) all as white solid.

Transformation experiments of (*S*)-1k

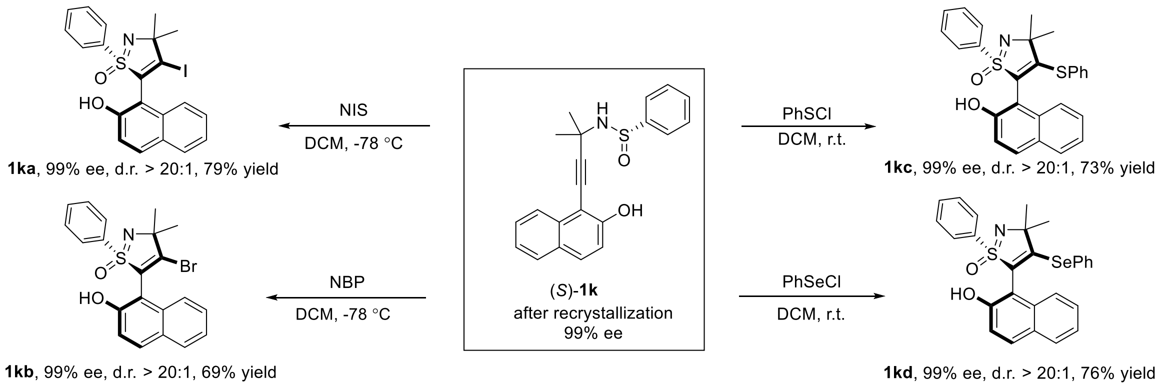

A flame-dried Schlenk tube equipped with a magnetic stirring bar, was charged with (*S*)-**1k** (0.1 mmol, 1 equiv), DCM (4.0 mL) was injected into the tube at corresponding temperature. After stirring for 5min, the corresponding electrophile reagents (NIS, NBP, PhSCl, PhSeCl, 1.1 equiv) was added into the mixture, after the reaction was completed, monitored by TLC. The reaction was quenched with saturated ammonium chloride solution, and extracted with DCM (3×5.0 mL), all organic layer was remove under reduced pressure, and the residue was purified by column chromatography (PE: EA = 5:1) to afford the desired product **1ka-1kd.**.

(*aR, R*)-5-(2-hydroxynaphthalen-1-yl)-4-iodo-3,3-dimethyl-1-phenyl-3H-isothiazole 1-oxide (1ka)

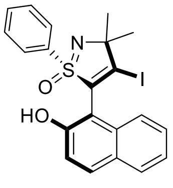

1ka

Appearance: white solid.

Yield: 79%, 37.5 mg. **ee** = 99%.

^{1}H NMR (400 MHz, $CDCl_3$) δ 8.19 (s, 1H), 7.83 (d, *J* = 7.8 Hz, 2H), 7.76 (d, *J* = 8.9 Hz, 1H), 7.67 (d, *J* = 7.8 Hz, 1H), 7.43 (t, *J* = 7.5 Hz, 1H), 7.32 (t, *J* = 7.7 Hz, 2H), 7.27 (d, *J* = 8.1 Hz, 1H), 7.18 (p, *J* = 7.1 Hz, 2H), 6.86 (d, *J* = 8.2 Hz, 1H), 1.80 (s, 6H).

^{13}C NMR (100 MHz, $CDCl_3$) δ 153.70, 136.40, 136.18, 134.08, 132.01, 131.41, 129.96, 129.09, 128.66, 128.35, 126.97, 123.55, 122.35, 119.62, 108.56, 78.86, 31.32, 30.37.

HRMS (ESI) m/z Calcd for $[C_{21}H_{18}INNaO_2S, M + Na]^+$: 497.9995, Found: 497.9991.

Optical Rotation: $[\alpha]_D^{25}$= -190.0° (*c* =1.0, DCM).

HPLC analysis: Chiralcel OD-H (Hexane/*i*-PrOH = 85:15, flow rate = 1.0 mL/min, wave length = 254 nm), t_R = 5.233 min (major), t_R = 7.997 min (minor).

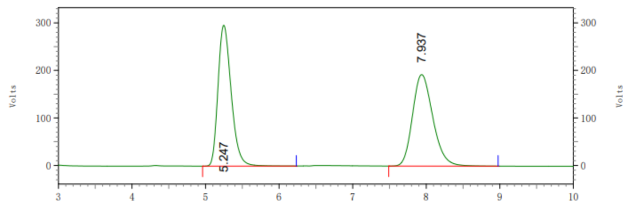

UV-WL1 254 nm

RetTime (min)	Width (min)	Height (Volts)	Area	Area (%)
5.247	1.27	1185472	14791310	49.982
7.937	1.49	770268	14801957	50.018

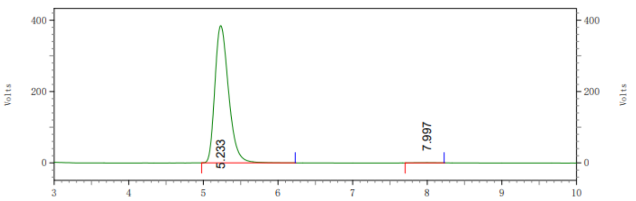

UV-WL1 254 nm

RetTime (min)	Width (min)	Height (Volts)	Area	Area (%)
5.233	1.25	1537876	19639489	99.847
7.997	0.52	1854	30025	0.153

(*aR, R*)-4-bromo-5-(2-hydroxynaphthalen-1-yl)-3, 3-dimethyl-1-phenyl-3H-isothiazole 1-oxide (1kb)

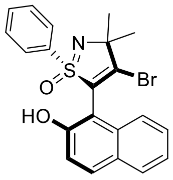

1kb

Appearance: white solid.

Yield: 69%, 29.5 mg. **ee** = 99%.

^{1}H NMR (400 MHz, DMSO-d_6) δ 10.14 (s, 1H), 7.82 (d, *J* = 9.0 Hz, 1H), 7.68 (d, *J* = 8.1 Hz, 1H), 7.60 (d, *J* = 7.4 Hz, 2H), 7.54 (t, *J* = 7.3 Hz, 1H), 7.39 (t, *J* = 7.7 Hz, 2H), 7.25 (d, *J* = 8.9 Hz, 1H), 7.07 (t, *J* = 7.4 Hz, 1H), 6.82 (t, *J* = 7.6 Hz, 1H), 6.45 (d, *J* = 8.4 Hz, 1H), 1.71 (s, 3H), 1.66 (s, 3H).

^{13}C NMR (100 MHz, DMSO-d_6) δ 154.95, 153.42, 139.35, 134.36, 133.56, 132.09, 131.40, 129.56, 129.21, 128.00, 127.25, 126.09, 122.59, 122.00, 118.26, 105.95, 74.37, 30.81, 28.76.

HRMS (ESI) m/z Calcd for $[C_{21}H_{18}BrNNaO_2S, M + Na]^+$: 450.0134, Found: 450.0139.

Optical Rotation: $[\alpha]_D^{25}$= -167.0° (*c* =1.0, DCM).

HPLC analysis: Chiralcel OD-H (Hexane/*i*-PrOH = 85:15, flow rate = 1.0 mL/min, wave length = 254 nm), t_R = 5.477 min (major), t_R = 8.367 min (minor).

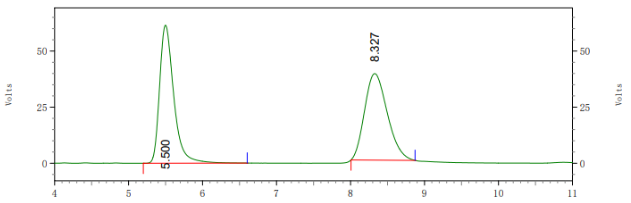

UV-WL1 254 nm

RetTime (min)	Width (min)	Height (Volts)	Area	Area (%)
5.500	1.40	246119	3271941	49.831
8.327	0.87	154321	3294195	50.169

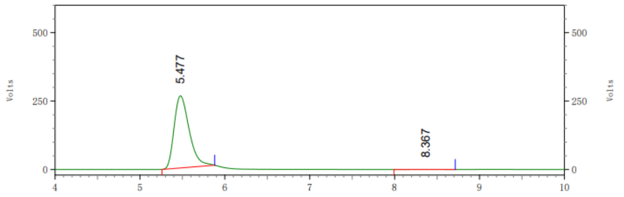

UV-WL1 254 nm

RetTime (min)	Width (min)	Height (Volts)	Area	Area (%)
5.477	0.62	1053465	12978824	99.828
8.367	0.72	1082	22368	0.172

Physical property comparison of 1kb and 2k by both chiral HPLC and normal HPLC

As shown in Supplementary Figure 1, **1kb**, obtained from **1k**, and **2k** were demonstrated to be enantiomers of each other by mixing either one with rac-**2k** and running chiral HPLC analysis.

Chiral HPLC analysis of 2k and 1kb:
Chiralcel OD-H, Hexane/*i*-PrOH = 85:15, flow rate = 1.0 mL/min, wave length = 254 nm

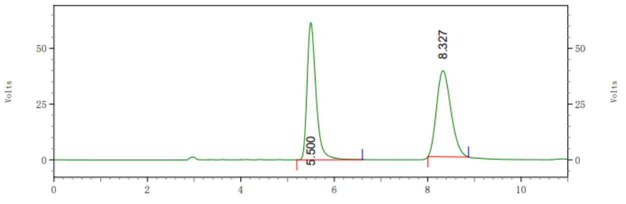

UV-WL1 254 nm

RetTime (min)	Width (min)	Height (Volts)	Area	Area (%)
5.500	1.40	246119	3271941	49.831
8.327	0.87	154321	3294195	50.169

rac-2k

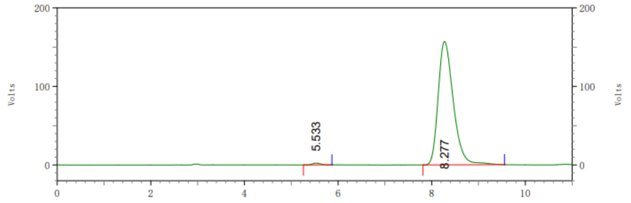

UV-WL1 254 nm

RetTime (min)	Width (min)	Height (Volts)	Area	Area (%)
5.533	0.61	9182	116011	0.812
8.277	1.74	628511	14162279	99.188

2k

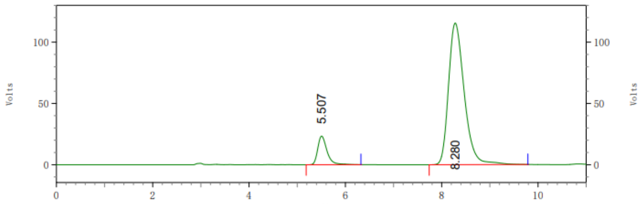

UV-WL1 254 nm

RetTime (min)	Width (min)	Height (Volts)	Area	Area (%)
5.507	1.14	93514	1253439	10.681
8.280	2.05	462169	10481441	89.319

2k + rac-2k

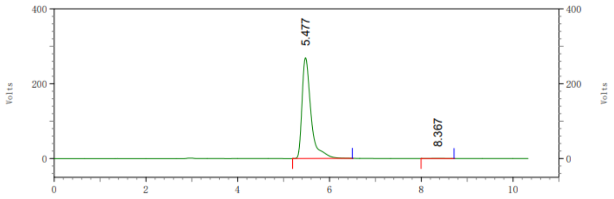

UV-WL1 254 nm

RetTime (min)	Width (min)	Height (Volts)	Area	Area (%)
5.477	1.30	1076082	14643978	99.847
8.367	0.72	1082	22368	0.153

1kb

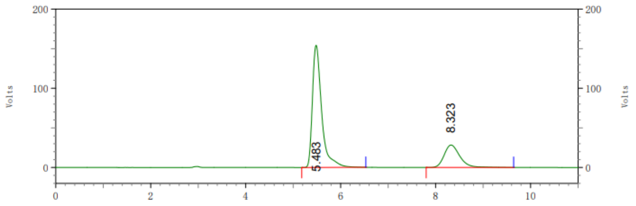

UV-WL1 254 nm

RetTime (min)	Width (min)	Height (Volts)	Area	Area (%)
5.483	1.35	617032	8358455	76.351
8.323	1.84	113261	2588999	23.649

1kb + rac-2k

Supplementary Figure 1. The chiral HPLC analysis of 1kb and 2k.

Retention times of **1kb**, **2k** and rac-**2k** were identical in normal HPLC chromatograms, suggesting that they are not diastereomers.

**Regular HPLC analysis of 2k and 1kb:** SHIMADZU(LC-20AD), C18(4.6*250 mm, 5 μm), Methanol/H_2O = 75:25, flow rate = 1.0 mL/min, wave length = 254 nm.	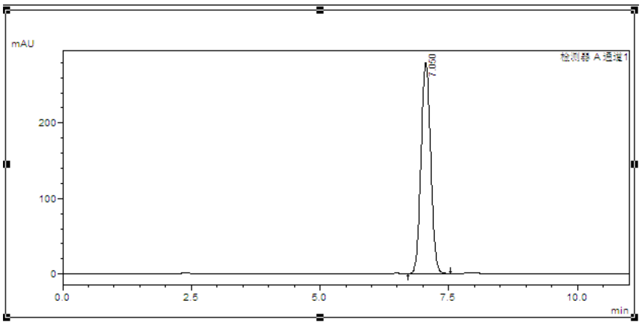
	rac-2k, t_R = 7.050 min
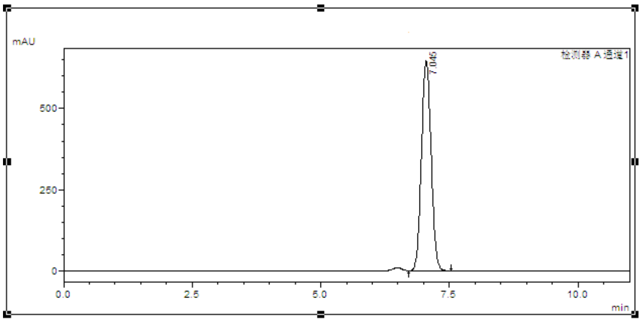	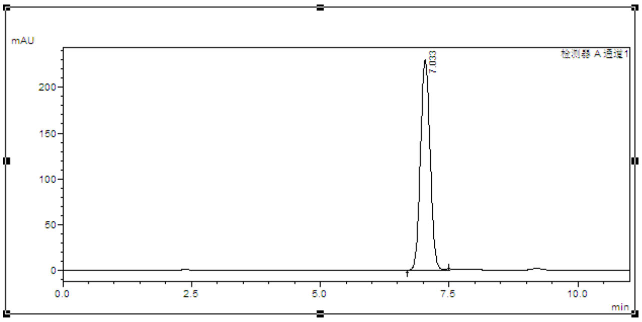
2k, t_R = 7.045 min	1kb, t_R = 7.033 min
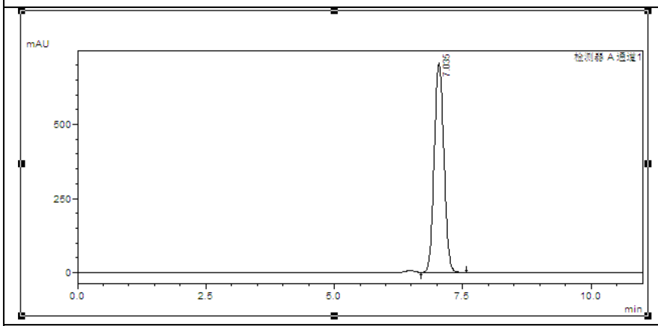	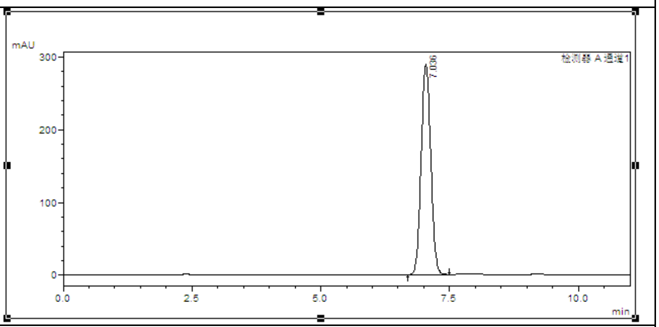
2k + rac-2k, t_R = 7.035 min	1kb + rac-2k, t_R = 7.036 min

Supplementary Figure 2. The regular HPLC analysis of 1kb and 2k.

(*aR, R*)-5-(2-hydroxynaphthalen-1-yl)-3,3-dimethyl-1-phenyl-4-(phenylthio)-3H-isothiazole 1-oxide (1kc)

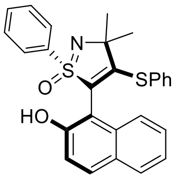

1kc

Appearance: white solid.

Yield: 76%, 33.4 mg. **ee** = 99%.

^{1}H NMR (400 MHz, $CDCl_3$) δ 8.44 (s, 1H), 7.79 – 7.72 (m, 2H), 7.40 (d, *J* = 7.9 Hz, 1H), 7.29 (dd, *J* = 13.6, 8.1 Hz, 2H), 7.25 – 7.17 (m, 3H), 7.15 – 7.09 (m, 1H), 6.95 (d, *J* = 8.4 Hz, 1H), 6.77 (dd, *J* = 6.0, 3.3 Hz, 1H), 6.75 (d, *J* = 8.9 Hz, 1H), 6.62 (q, *J* = 4.6, 3.5 Hz, 4H), 2.03 (s, 3H), 1.86 (s, 3H).

^{13}C NMR (100 MHz, $CDCl_3$) δ 165.70, 153.64, 136.89, 133.47, 132.69, 132.07, 131.24, 129.68, 128.71, 128.53, 128.31, 127.92, 127.72, 126.49, 125.48, 125.28, 123.18, 122.99, 122.76, 119.08, 106.72, 77.90, 33.10, 29.97.

HRMS (ESI) m/z Calcd for $[C_{27}H_{23}NNaO_2S_2, M + Na]^+$: 480.1062, Found: 480.1068.

Optical Rotation: $[\alpha]_D^{25}$= -351.0° (*c* =1.0, DCM).

HPLC analysis: Chiralcel OD-H (Hexane/*i*-PrOH = 85:15, flow rate = 1.0 mL/min, wave length = 254 nm), t_R = 4.787 min (major), t_R = 6.457 min (minor).

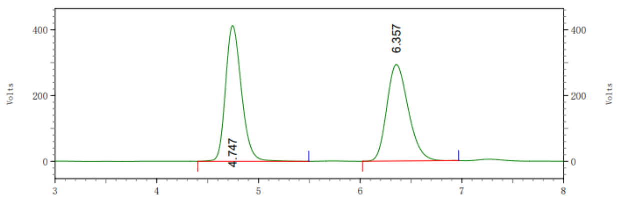

UV-WL1 254 nm

RetTime (min)	Width (min)	Height (Volts)	Area	Area (%)
4.747	1.09	1650183	17916117	50.923
6.357	0.94	1172892	17266930	49.077

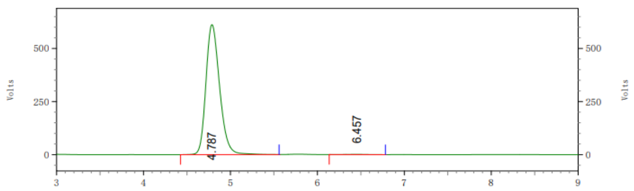

UV-WL1 254 nm

RetTime (min)	Width (min)	Height (Volts)	Area	Area (%)
4.787	1.13	2447679	26756096	99.803
6.457	0.65	3389	52763	0.197

(*aR, R*)-5-(2-hydroxynaphthalen-1-yl)-3,3-dimethyl-1-phenyl-4-(phenylselanyl)-3H-isothiazole 1-oxide (1kd)

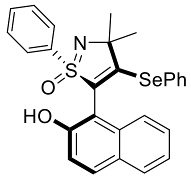

1kd

Appearance: white solid.

Yield: 73%, 38.4 mg. **ee** = 99%.

^{1}H NMR (400 MHz, $CDCl_3$) δ 8.29 (s, 1H), 7.76 (d, *J* = 7.7 Hz, 2H), 7.45 (d, *J* = 7.8 Hz, 1H), 7.36 – 7.28 (m, 2H), 7.23 (t, *J* = 7.6 Hz, 2H), 7.18 (d, *J* = 7.3 Hz, 1H), 7.14 (t, *J* = 7.2 Hz, 1H), 7.00 (d, *J* = 8.2 Hz, 1H), 6.87 (t, *J* = 6.8 Hz, 1H), 6.78 – 6.61 (m, 5H), 2.01 (s, 3H), 1.83 (s, 3H).

^{13}C NMR (100 MHz, $CDCl_3$) δ 164.59, 153.69, 136.74, 134.42, 133.54, 131.72, 131.25, 129.79, 129.59, 128.76, 128.43, 128.18, 127.93, 126.43, 123.03, 122.83, 122.68, 119.19, 106.96, 78.65, 33.30, 30.26.

HRMS (ESI) m/z Calcd for $[C_{27}H_{23}NNaO_2SSe, M + Na]^+$: 528.0507, Found: 528.0501.

Optical Rotation: $[\alpha]_D^{25}$= -677.0° (*c* =1.0, DCM).

HPLC analysis: Chiralcel AD-H (Hexane/*i*-PrOH = 85:15, flow rate = 1.0 mL/min, wave length = 254 nm), t_R = 6.107 min (major), t_R = 20.987 min (minor).

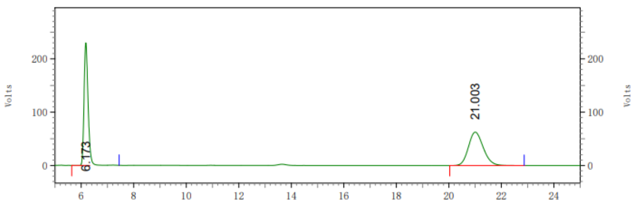

UV-WL1 254 nm

RetTime (min)	Width (min)	Height (Volts)	Area	Area (%)
6.173	1.80	919703	9887878	50.128
21.003	2.83	250411	9837330	49.872

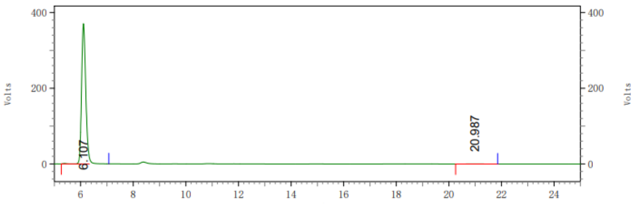

UV-WL1 254 nm

RetTime (min)	Width (min)	Height (Volts)	Area	Area (%)
6.107	1.80	1483449	16494792	99.858
20.987	1.60	632	23447	0.142

Transformation experiments of (*aS, S*)-2k

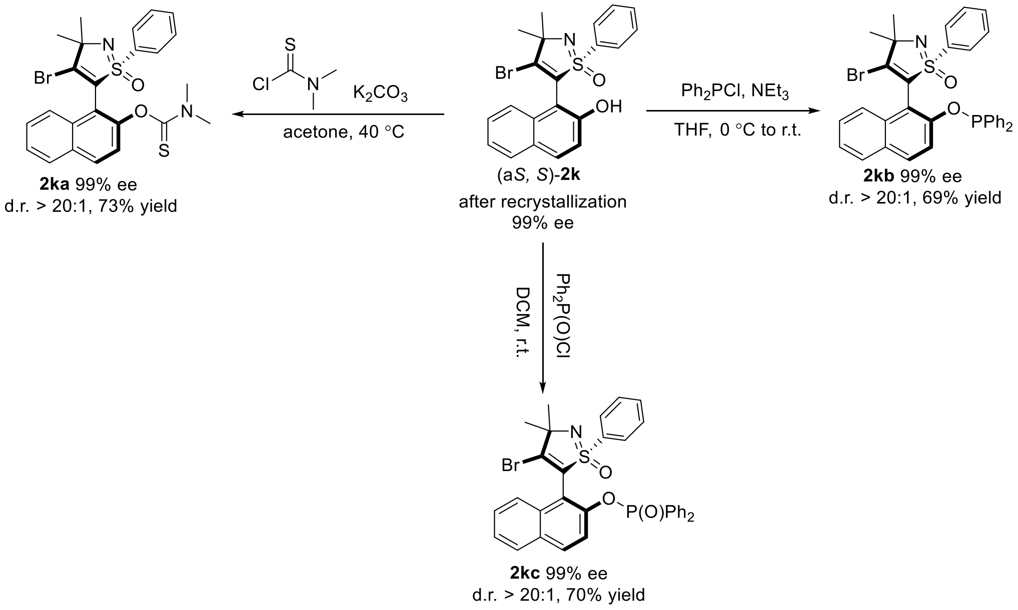

Synthesis of 2ka: A solution of (*aS, S*)-**2k** (0.1 mmol, 1 equiv) in 1 mL of acetone containing of potassium carbonate (0.5 mmol, 69 mg) was stirred at 40 °C for 30 minutes then dimethylthiocarbamoyl chloride (0.2 mmol, 24.7 mg) was added. The reaction was refluxed overnight. After total disappearance of starting material (monitored by TLC), the solvent was evaporated under reduced pressure, the residue was treated with dichloromethane and washed with water. The organic layer was dried over anhydrous Na_2SO_4. The crude white solid obtained after evaporation of the solvent was purified by flash chromatography on silica gel (PE: EA = 5:1) to afford white solid **2ka**.

(*aS,S*)-O-(1-(4-bromo-3,3-dimethyl-1-oxido-1-phenyl-3H-1l6-isothiazol-5-yl)naphthalen-2-yl) dimethylcarbamothioate(2ka)

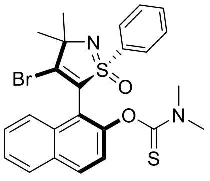

2ka

Appearance: white solid.

Yield: 73%, 38 mg. **ee** = 99%.

^{1}H NMR (400 MHz, $CDCl_3$)δ 7.88 (d, *J* = 8.9 Hz, 1H), 7.74 (d, *J* = 8.1 Hz, 1H), 7.70 (d, *J* = 7.7 Hz, 2H), 7.55 (d, *J* = 8.9 Hz, 1H), 7.35 (t, *J* = 7.2 Hz, 1H), 7.29 (s, 1H), 7.22 (t, *J* = 7.6 Hz, 2H), 7.00 (t, *J* = 7.5 Hz, 1H), 6.70 (d, *J* = 8.4 Hz, 1H), 3.49 (s, 6H), 1.81 (s, 3H), 1.76 (s, 3H).

^{13}C NMR (100 MHz, $CDCl_3$) δ 186.37, 154.09, 150.49, 138.67, 133.24, 133.16, 131.20, 130.89, 129.96, 129.39, 128.65, 128.16, 126.46, 125.44, 123.75, 123.14, 115.80, 75.11, 43.11, 38.99, 30.85, 29.02.

HRMS (ESI) m/z Calcd for $[C_{24}H_{23}BrN_2NaO_2S_2, M + Na]^+$: 537.0277, Found: 537.0273.

Optical Rotation: $[\alpha]_D^{25}$= 21.1° (*c* =1.0, DCM).

HPLC analysis: Chiralcel OD-H (Hexane/*i*-PrOH = 85:15, flow rate = 1.0 mL/min, wave length = 254 nm), t_R = 10.063 min (minor), t_R = 11.997 min (major).

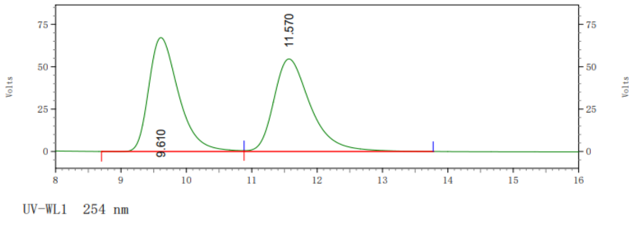

UV-WL1 254 nm

RetTime (min)	Width (min)	Height (Volts)	Area	Area (%)
9.610	2.18	268882	8537871	49.985
11.570	2.89	218456	8543010	50.015

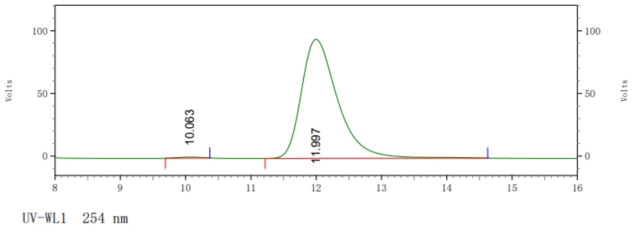

UV-WL1 254 nm

RetTime (min)	Width (min)	Height (Volts)	Area	Area (%)
10.063	0.68	3155	72651	0.493
11.997	3.41	379812	14657012	99.507

Synthesis of 2kb:

The (*aS*, *S*)-**2k** (0.1 mmol, 1.0 equiv) and DMAP (0.15 mmol, 1.5 equiv) were dissolved into 2.0 mL of THF under an argon atmosphere and cooled to 0 ºC with an ice bath. A solution of chlorodiphenylphosphine (0.45 mmol, 4.5 equiv) in THF (1 mL) was added dropwise and followed by the addition of triethylamine (1.8 mmol, 18.0 equiv). The reaction mixture was allowed to warm to ambient temperature and stirred for 3.0 h. Filtration of triethylammonium chloride followed by flash column chromatography (PE: EA = 5: 1) on basic silica gel to give desired product **2kb**.

(*aS*,*S*)-4-bromo-5-(2-((diphenylphosphaneyl)oxy)naphthalen-1-yl)-3,3-dimethyl-1-phenyl-3H-isothiazole 1-oxide-(2kb)

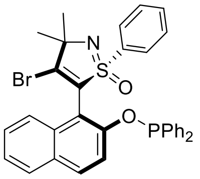

2kb

Appearance: white solid.

Yield: 69%, 42 mg. **ee** = 99%.

^{1}H NMR (400 MHz, $CDCl_3$) δ 8.28 (dd, *J* = 12.7, 7.3 Hz, 2H), 8.12 (dd, *J* = 13.0, 7.4 Hz, 2H), 7.84 (d, *J* = 9.1 Hz, 1H), 7.67 (td, *J* = 15.6, 14.3, 8.7 Hz, 4H), 7.50 (dq, *J* = 12.8, 7.6, 6.9 Hz, 6H), 7.41 (d, *J* = 7.4 Hz, 1H), 7.26 (d, *J* = 4.8 Hz, 2H), 7.16 (t, *J* = 7.6 Hz, 1H), 6.86 (t, *J* = 7.8 Hz, 1H), 6.53 (d, *J* = 8.5 Hz, 1H), 1.78 (s, 3H), 1.51 (s, 3H).

^{13}C NMR (100 MHz, $CDCl_3$) δ 154.27, 149.07, 149.00, 138.90, 133.40, 132.57, 132.47, 131.87, 131.76, 131.67, 131.15, 130.41, 129.82, 129.70, 129.53, 128.85, 128.79, 128.66, 128.59, 128.45, 127.98, 126.51, 124.72, 123.02, 119.01, 112.39, 75.30, 30.10, 29.12.

HRMS (ESI) m/z Calcd for $[C_{33}H_{27}BrNNaO_2PS, M + Na]^+$: 634.0576, Found: 634.0574.

Optical Rotation: $[\alpha]_D^{25}$= -90° (*c* =1.0, DCM).

HPLC analysis: Chiralcel IA-H (Hexane/*i*-PrOH = 80:20, flow rate = 1.0 mL/min, wave length = 254 nm), t_R = 20.280 min (minor), t_R = 23.493 min (major).

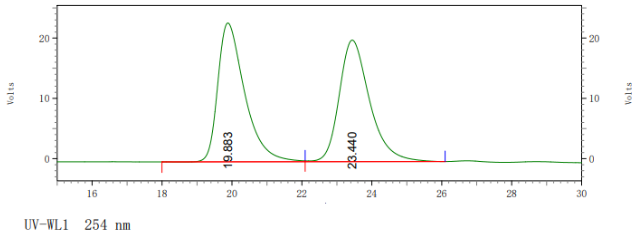

RetTime (min)	Width (min)	Height (Volts)	Area	Area (%)
19.883	4.10	92093	4923850	50.243
23.440	4.00	80637	4876193	49.757

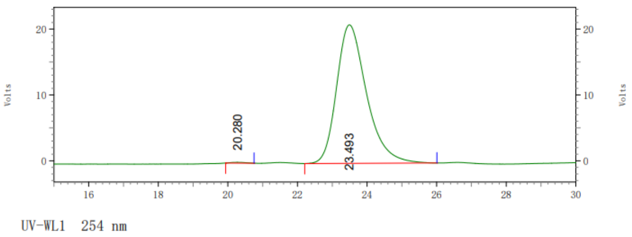

RetTime (min)	Width (min)	Height (Volts)	Area	Area (%)
20.280	0.82	452	13276	0.264
23.493	3.80	84075	5024250	99.736

Synthesis of 2kc:

To a solution of (*aS*, *S*)-**2k** (0.1 mmol, 1.0 equiv) in DCM (2.0 mL) was added triethylamine (0.13 mmol, 1.3 equiv), followed by catalytic DMAP. Diphenylphosphinic chloride (0.11 mmol, 1.1 equiv) was added dropwise, and the resulting mixture was allowed to stir at r.t. for 3h. The mixture was added to saturated NH_4Cl solution (5 mL). The layers were separated, and the aqueous layer was extracted with DCM. The combined organic layers were washed with saturated $NaHCO_3$ and water, then dried over Na_2SO_4, filtered, and concentrated in vacuo to afford the crude product, which was purified by flash chromatography on silica gel using PE/EA (5: 1) as the eluent to afford the corresponding **2kc**.

(*aS*, *S*)-1-(4-bromo-3,3-dimethyl-1-oxido-1-phenyl-3H-1l6-isothiazol-5-yl)naphthalen-2-yl diphenylphosphinate (2kc)

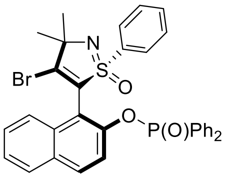

2kc

Appearance: white solid.

Yield: 70%, 42.8 mg. **ee** = 99%.

^{1}H NMR (400 MHz, $CDCl_3$) δ 8.28 (dd, *J* = 12.6, 7.2 Hz, 2H), 8.12 (dd, *J* = 13.0, 7.4 Hz, 2H), 7.83 (d, *J* = 9.1 Hz, 1H), 7.67 (td, *J* = 15.9, 15.0, 8.6 Hz, 4H), 7.50 (dq, *J* = 14.5, 7.8 Hz, 6H), 7.41 (t, *J* = 7.4 Hz, 1H), 7.30 – 7.20 (m, 2H), 7.16 (t, *J* = 7.6 Hz, 1H), 6.86 (t, *J* = 7.7 Hz, 1H), 6.53 (d, *J* = 8.5 Hz, 1H), 1.78 (s, 3H), 1.51 (s, 3H).

^{13}C NMR (100 MHz, $CDCl_3$) δ 154.28, 149.07, 138.95, 133.39, 132.58, 132.48, 131.88, 131.77, 131.67, 131.16, 129.71, 129.54, 128.85, 128.67, 128.60, 128.46, 127.98, 126.52, 124.72, 123.03, 119.04, 77.00, 75.32, 30.12, 29.14.

HRMS (ESI) m/z Calcd for $[C_{33}H_{27}BrNNaO_3PS, M + Na]^+$: 650.0525, Found: 650.0529.

Optical Rotation: $[\alpha]_D^{25}$= -120.5° (*c* = 1.0, DCM).

HPLC analysis: Chiralcel OD-H (Hexane/*i*-PrOH = 85:15, flow rate = 1.0 mL/min, wave length = 254 nm), t_R = 9.283 min (minor), t_R = 10.790 min (major).

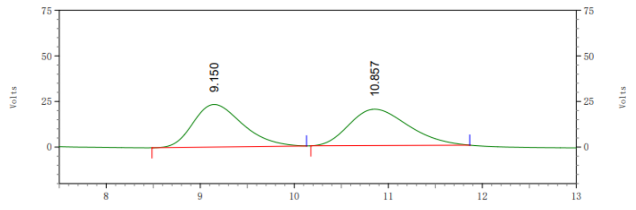

UV-WL1 254 nm

RetTime (min)	Width (min)	Height (Volts)	Area	Area (%)
9.150	1.64	93400	3411353	49.428
10.857	1.69	79726	3490264	50.572

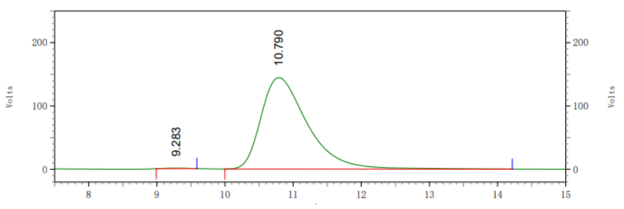

UV-WL1 254 nm

RetTime (min)	Width (min)	Height (Volts)	Area	Area (%)
9.283	0.60	3127	69248	0.253
10.790	4.22	577259	27295161	99.747

General procedure for the asymmetric synthesis of compounds (*R*)-4a-4p

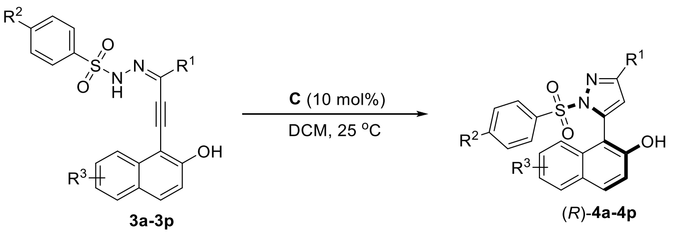

A flame-dried Schlenk tube equipped with a magnetic stirring bar, was charged with **3a-3p** (0.1 mmol, 1 equiv), and catalyst **C** (10 mol%), DCM (4.0 mL) was injected into the tube at 25 °C. After stirring for 12 h, the mixture was filtered and purified by silica gel chromatography (PE/EA = 4:1) to afford the product (*R*)-**4a-4p**. Racemic samples were prepared without the addition of the catalyst.

Analytical dates of (*S*)-1a-1t and (*R*)-4a-4p

(*R*)-1-(3-(*tert*-butyl)-1-tosyl-1*H*-pyrazol-5-yl) naphthalen-2-ol (4a)

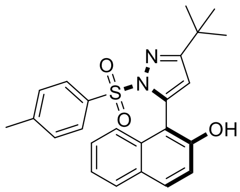

4a

Appearance: white solid.

Yield: 83%, 34.8 mg. **ee** = 91%.

^{1}H NMR (400 MHz, $CDCl_3$) δ 7.84 (d, *J* = 8.8 Hz, 1H), 7.76 (d, *J* = 8.0 Hz, 1H), 7.43 (d, *J* = 7.9 Hz, 2H), 7.26 (dd, *J* = 20.0, 8.1 Hz, 2H), 7.15 (t, *J* = 7.4 Hz, 1H), 7.00 (d, *J* = 7.8 Hz, 2H), 6.86 (d, *J* = 8.3 Hz, 1H), 6.37 (s, 1H), 5.63 (s, 1H), 2.30 (s, 3H), 1.37 (s, 9H).

^{13}C NMR (100 MHz, $CDCl_3$) δ 166.39, 152.37, 145.05, 139.74, 134.07, 133.52, 131.59, 129.24, 128.41, 128.10, 127.92, 126.68, 123.85, 123.33, 117.77, 111.23, 109.64, 32.80, 29.92, 21.52.

HRMS (ESI) m/z Calcd for $[C_{24}H_{24}N_2NaO_3S, M + Na]^+$: 443.1322, Found: 443.1325.

Optical Rotation: $[\alpha]_D^{25}$= 49° (*c* = 1.0, DCM).

HPLC analysis: Chiralcel IB-H (Hexane/*i*-PrOH = 80:20, flow rate = 1.0 mL/min, wave length = 254 nm), t_R = 8.608 min (major), t_R = 18.714 min (minor).

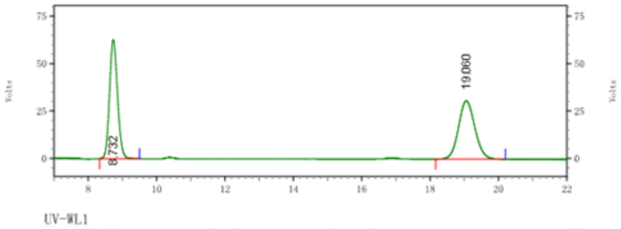

UV-WL1

RetTime (min)	Width (min)	Height (Volts)	Area	Area (%)
8.732	1.17	250992	4077993	50.035
19.060	2.04	122678	4072343	49.965

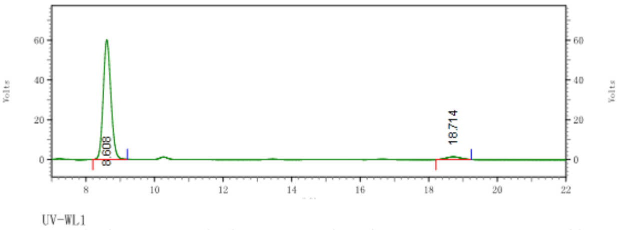

UV-WL1

RetTime (min)	Width (min)	Height (Volts)	Area	Area (%)
8.608	1.01	240563	3739384	95.833
18.714	1.03	5518	162598	4.167

(*R*)-1-(3-methyl-1-tosyl-1*H*-pyrazol-5-yl) naphthalen-2-ol (4b)

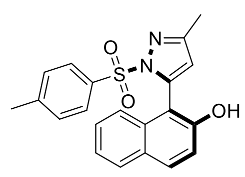

4b

Appearance: white solid.

Yield: 72%, 27.2 mg. **ee** = 88%.

^{1}H NMR (400 MHz, $CDCl_3$) δ 7.82 (d, J = 8.8 Hz, 1H), 7.71 (d, J = 8.0 Hz, 1H), 7.33 (d, J = 7.8 Hz, 2H), 7.21 (p, J = 9.6, 8.4 Hz, 3H), 7.08 (t, J = 7.4 Hz, 1H), 6.93 (d, J = 7.7 Hz, 2H), 6.80 (d, J = 8.3 Hz, 1H), 6.23 (s, 1H), 2.36 (s, 3H), 2.24 (s, 3H).

^{13}C NMR (100 MHz, $CDCl_3$) δ 153.63, 152.41, 145.31, 139.64, 133.92, 133.54, 131.81, 129.42, 128.46, 128.18, 127.94, 126.76, 123.93, 123.46, 117.83, 114.30, 109.39, 21.57, 14.09.

HRMS (ESI) m/z Calcd for $[C_{21}H_{18}N_2NaO_3S, M+Na]^+$: 401.0852, Found: 401.0857.

Optical Rotation: $[\alpha]_D^{25}$= 55° (*c* = 1.0, DCM).

HPLC analysis: Chiralcel OD-H (Hexane/*i*-PrOH = 85:15, flow rate = 1.0 mL/min, wave length = 254 nm), t_R = 9.094 min (minor), t_R = 11.054 min (major).

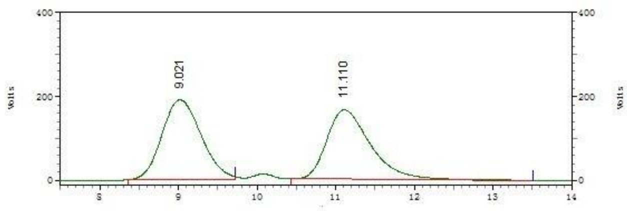

RetTime (min)	Width (min)	Height (Volts)	Area	Area (%)
9.021	1.36	758640	26419454	50.819
11.110	3.07	660647	25567676	49.181

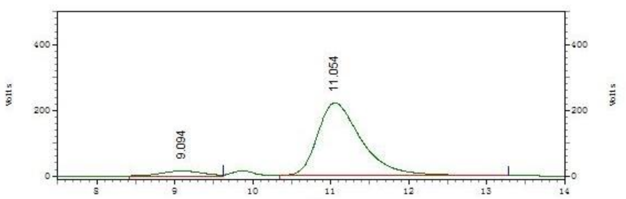

RetTime (min)	Width (min)	Height (Volts)	Area	Area (%)
9.094	1.21	59141	2116509	5.792
11.054	2.94	882394	34422362	94.208

(*R*)-1-(3-ethyl-1-tosyl-1*H*-pyrazol-5-yl) naphthalen-2-ol (4c)

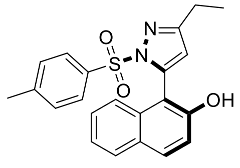

4c

Appearance: white solid.

Yield: 75%, 29.4 mg. **ee** = 99%.

^{1}H NMR (400 MHz, $CDCl_3$) δ 7.81 (d, *J* = 8.9 Hz, 1H), 7.70 (d, *J* = 8.1 Hz, 1H), 7.32 (d, *J* = 8.0 Hz, 2H), 7.20 (q, *J* = 12.1, 9.8 Hz, 3H), 7.06 (t, *J* = 7.6 Hz, 1H), 6.92 (d, *J* = 8.0 Hz, 2H), 6.77 (d, *J* = 8.4 Hz, 1H), 6.26 (s, 1H), 5.16 (s, 1H), 2.73 (q, *J* = 7.6 Hz, 2H), 2.23 (s, 3H), 1.25 (t, *J* = 7.6 Hz, 3H).

^{13}C NMR (100 MHz, $CDCl_3$) δ 159.25, 152.40, 145.27, 139.57, 133.93, 133.55, 131.79, 129.39, 128.47, 128.17, 127.94, 126.75, 123.92, 123.45, 117.84, 112.90, 109.51, 21.83, 21.56, 13.10.

HRMS (ESI) m/z Calcd for $[C_{22}H_{20}N_2NaO_3S, M + Na]^+$: 415.1009, Found: 415.1003.

Optical Rotation: $[\alpha]_D^{25}$= 33° (*c* = 1.0, DCM).

HPLC analysis: Chiralcel OD-H (Hexane/*i*-PrOH = 85:15, flow rate = 1.0 mL/min, wave length = 254 nm), t_R = 6.375 min (major), t_R = 7.061 min (minor).

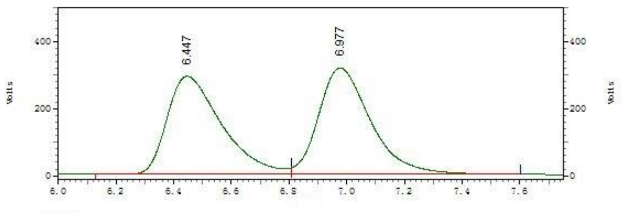

RetTime (min)	Width (min)	Height (Volts)	Area	Area (%)
6.447	0.68	1167956	15371928	49.173
6.977	0.79	1267379	15888899	50.827

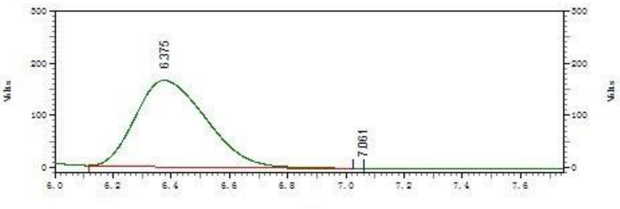

RetTime (min)	Width (min)	Height (Volts)	Area	Area (%)
6.375	0.91	656462	11034143	100.000
7.061	0.00	0	0	0.000

(*R*)-1-(3-ethyl-1-tosyl-1*H*-pyrazol-5-yl) naphthalen-2-ol (4d)

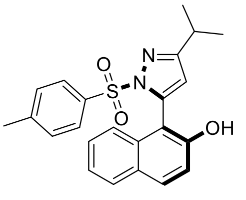

4d

Appearance: white solid.

Yield: 77%, 31.2 mg. **ee** = 89%.

^{1}H NMR (400 MHz, $CDCl_3$) δ 7.79 (d, *J* = 8.9 Hz, 1H), 7.69 (d, *J* = 8.1 Hz, 1H), 7.32 (d, *J* = 8.0 Hz, 2H), 7.25 – 7.14 (m, 2H), 7.06 (t, *J* = 7.6 Hz, 1H), 6.91 (d, *J* = 7.9 Hz, 2H), 6.75 (d, *J* = 8.4 Hz, 1H), 6.27 (s, 1H), 3.07 (dt, *J* = 13.9, 6.9 Hz, 1H), 2.22 (s, 3H), 1.25 (d, *J* = 6.9 Hz, 6H).

^{13}C NMR (100 MHz, $CDCl_3$) δ 163.56, 152.39, 145.19, 139.58, 133.94, 133.53, 131.74, 129.33, 128.45, 128.13, 127.93, 126.73, 123.86, 123.40, 117.83, 111.55, 109.58, 28.23, 22.23, 21.55.

HRMS (ESI) m/z Calcd for $[C_{23}H_{22}N_2NaO_3S, M + Na]^+$: 429.1165, Found: 429.1168.

Optical Rotation: $[\alpha]_D^{25}$= 32° (*c* = 1.0, DCM).

HPLC analysis: Chiralcel OD-H (Hexane/*i*-PrOH = 90:10, flow rate = 1.0 mL/min, wave length = 254 nm), t_R = 8.362 min (minor), t_R = 14.429 min (major).

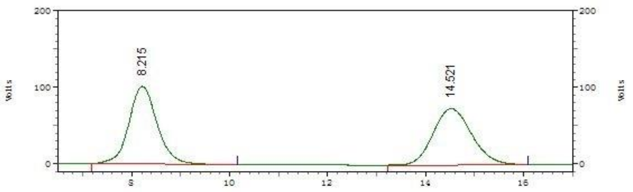

RetTime (min)	Width (min)	Height (Volts)	Area	Area (%)
8.215	3.00	405644	16277248	50.288
14.521	2.87	293686	16090973	49.712

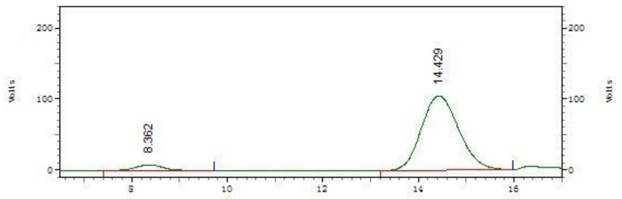

RetTime (min)	Width (min)	Height (Volts)	Area	Area (%)
8.362	2.33	33347	1378223	5.699
14.429	2.77	419702	22805301	94.301

(*R*)-1-(3-(pentan-3-yl)-1-tosyl-1*H*-pyrazol-5-yl) naphthalen-2-ol (4e)

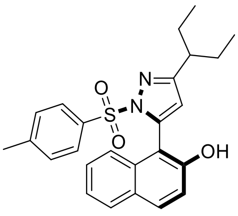

4e

Appearance: white solid.

Yield: 80%, 34.7 mg. **ee** = 93%.

^{1}H NMR (400 MHz, $CDCl_3$) δ 7.87 (d, *J* = 8.4 Hz, 1H), 7.77 (d, *J* = 7.4 Hz, 1H), 7.37 (d, *J* = 7.2 Hz, 2H), 7.30 (s, 1H), 7.17 – 7.08 (m, 1H), 6.97 (d, *J* = 7.1 Hz, 2H), 6.80 (d, *J* = 7.6 Hz, 1H), 6.30 (s, 1H), 5.43 (s, 1H), 2.74 (s, 1H), 2.29 (s, 3H), 1.81 – 1.67 (m, 2H), 1.65 – 1.50 (m, 2H), 0.84 (s, 6H).

^{13}C NMR (100 MHz, a $CDCl_3$) δ 161.82, 152.27, 145.16, 139.92, 133.86, 133.42, 131.77, 129.29, 128.45, 127.94, 126.78, 123.67, 123.39, 117.81, 112.15, 109.97, 109.53, 42.54, 27.62, 21.54, 11.92.

HRMS (ESI) m/z Calcd for $[C_{25}H_{26}N_2NaO_3S, M + Na]^+$: 457.1556, Found: 457.1550.

Optical Rotation: $[\alpha]_D^{25}$= 40° (*c* = 1.0, DCM).

HPLC analysis: Chiralcel AD-H (Hexane/*i*-PrOH = 90:10, flow rate = 1.0 mL/min, wave length = 254 nm), t_R = 5.477 min (minor), t_R = 7.242 min (major).

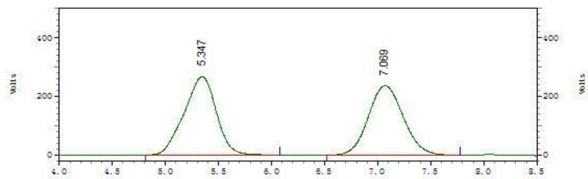

RetTime (min)	Width (min)	Height (Volts)	Area	Area (%)
5.347	1.27	1068107	22477780	50.884
7.069	1.26	947250	21696363	49.116

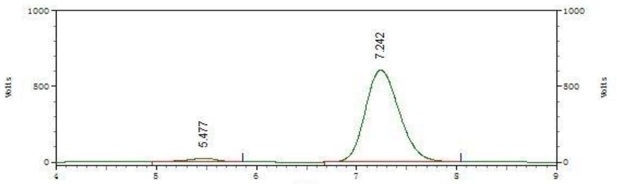

RetTime (min)	Width (min)	Height (Volts)	Area	Area (%)
5.477	0.90	97367	2044851	3.463
7.242	1.36	2423503	57010192	96.537

(*R*)-1-(3-cyclopentyl-1-tosyl-1*H*-pyrazol-5-yl) naphthalen-2-ol (4f)

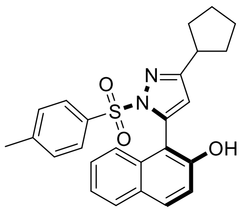

4f

Appearance: white solid.

Yield: 84%, 36.3 mg. **ee** = 95%.

^{1}H NMR (400 MHz, $CDCl_3$) δ 7.79 (d, *J* = 8.9 Hz, 1H), 7.69 (d, *J* = 8.1 Hz, 1H), 7.32 (d, *J* = 7.9 Hz, 2H), 7.26 – 7.13 (m, 2H), 7.06 (t, *J* = 7.7 Hz, 1H), 6.91 (d, *J* = 8.0 Hz, 2H), 6.76 (d, *J* = 8.5 Hz, 1H), 6.25 (s, 1H), 5.42 (s, 1H), 3.17 (dd, *J* = 10.3, 5.5 Hz, 1H), 2.23 (s, 3H), 2.11 – 1.98 (m, 2H), 1.76 – 1.53 (m, 7H).

^{13}C NMR (100 MHz, $CDCl_3$) δ 162.18, 152.33, 145.19, 139.54, 133.86, 133.49, 131.74, 129.33, 128.41, 128.11, 127.91, 126.72, 123.86, 123.40, 117.79, 112.07, 39.08, 33.02, 25.45, 21.56.

HRMS (ESI) m/z Calcd for $[C_{25}H_{24}N_2NaO_3S, M + Na]^+$: 455.1400, Found: 455.1405.

Optical Rotation: $[\alpha]_D^{25}$= 25° (*c* = 1.0, acetone).

HPLC analysis: Chiralcel OD-H (Hexane/*i*-PrOH = 90:10, flow rate = 1.0 mL/min, wave length = 254 nm), t_R = 8.172 min (minor), t_R = 11.167 min (major).

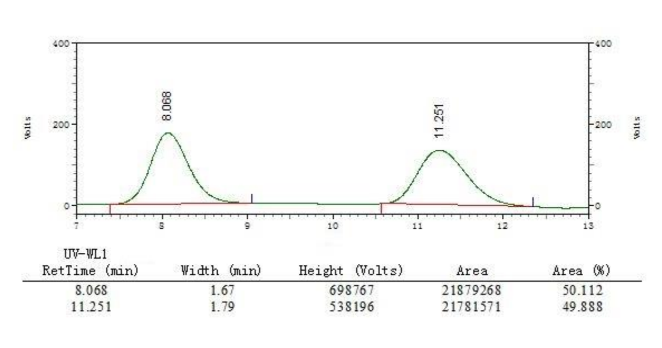

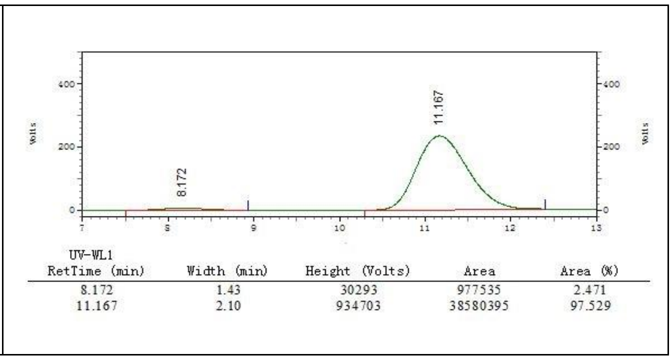

(*R*)-1-(3-cyclohexyl-1-tosyl-1*H*-pyrazol-5-yl) naphthalen-2-ol (4g)

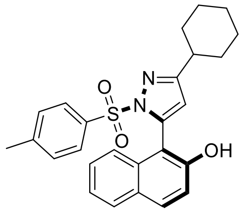

4g

Appearance: yellow solid.

Yield: 82%, 36.5 mg. **ee** = 96%.

^{1}H NMR (400 MHz, $CDCl_3$) δ 7.86 (d, *J* = 8.9 Hz, 1H), 7.76 (d, *J* = 8.1 Hz, 1H), 7.38 (d, *J* = 8.1 Hz, 2H), 7.32 – 7.22 (m, 2H), 7.12 (t, *J* = 7.6 Hz, 1H), 6.98 (d, *J* = 8.0 Hz, 2H), 6.81 (d, *J* = 8.4 Hz, 1H), 6.32 (s, 1H), 5.29 (s, 1H), 2.90 – 2.77 (m, 1H), 2.29 (s, 3H), 2.05 (d, *J* = 11.4 Hz, 2H), 1.82 (d, *J* = 12.0 Hz, 2H), 1.73 (d, *J* = 12.7 Hz, 1H), 1.43 (dq, *J* = 31.2, 11.4, 10.6 Hz, 4H), 1.30 (s, 1H).

^{13}C NMR (100 MHz, $CDCl_3$) δ 162.68, 152.31, 145.18, 139.28, 133.89, 133.50, 131.75, 129.33, 128.45, 128.09, 127.92, 126.73, 123.86, 123.41, 117.79, 111.85, 109.57, 37.72, 32.55, 32.49, 26.07, 25.90, 21.56.

HRMS (ESI) m/z Calcd for $[C_{26}H_{26}N_2NaO_3S, M + Na]^+$: 469.1556, Found: 469.1551.

Optical Rotation: $[\alpha]_D^{25}$= 26° (*c* = 1.0, DCM).

HPLC analysis: Chiralcel OD-H (Hexane/*i*-PrOH = 90:10, flow rate = 1.0 mL/min, wave length = 254 nm), t_R = 11.179 min (minor), t_R = 17.919 min (major).

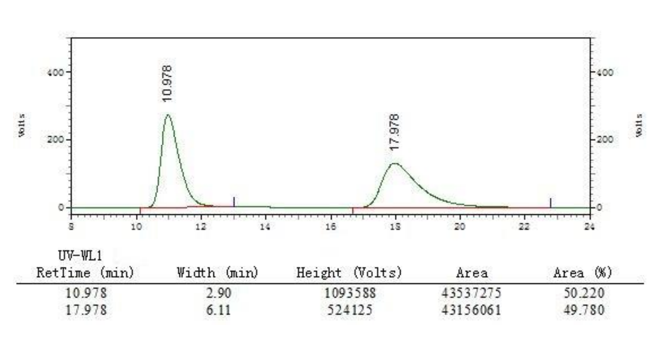

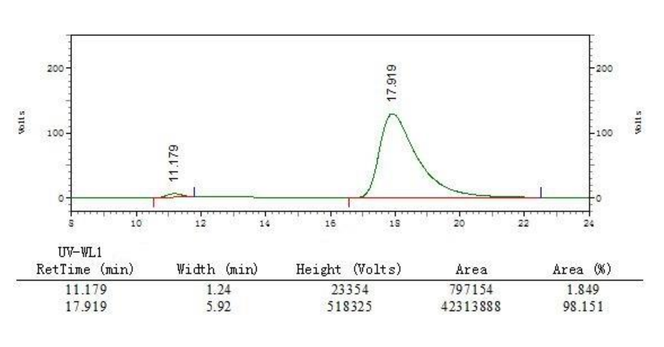

(*R*)-1-(3-((3*R*,5*R*,7*R*)-adamantan-1-yl)-1-tosyl-1*H*-pyrazol-5-yl) naphthalen-2-ol (4h)

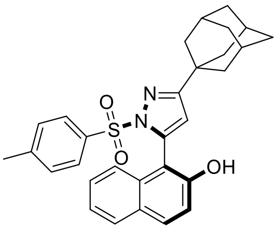

4h

Appearance: white solid.

Yield: 80%, 39.8 mg. **ee** = 96%.

^{1}H NMR (400 MHz, $CDCl_3$) δ 7.85 (d, *J* = 8.9 Hz, 1H), 7.76 (d, *J* = 8.0 Hz, 1H), 7.40 (d, *J* = 8.2 Hz, 2H), 7.33 – 7.20 (m, 2H), 7.18 – 7.10 (m, 1H), 6.99 (d, *J* = 8.1 Hz, 2H), 6.82 (d, *J* = 8.4 Hz, 1H), 6.36 (s, 1H), 5.39 (s, 1H), 2.30 (s, 3H), 2.09 (m, 3H), 2.02 (m, 6H), 1.78 (m, 6H).

^{13}C NMR (100 MHz, $CDCl_3$) δ 166.42, 152.25, 145.04, 139.33, 133.97, 133.48, 131.62, 129.25, 128.41, 128.04, 127.90, 126.67, 123.88, 123.35, 117.73, 110.87, 109.67, 41.84, 36.59, 34.69, 28.36, 21.55.

HRMS (ESI) m/z Calcd for $[C_{30}H_{30}N_2NaO_3S, M + Na]^+$: 521.1869, Found: 521.1860.

Optical Rotation: $[\alpha]_D^{25}$= 32° (*c* = 1.0, DCM).

HPLC analysis: Chiralcel OD-H (Hexane/*i*-PrOH = 90:10, flow rate = 1.0 mL/min, wave length = 254 nm), t_R = 10.483min (minor), t_R = 20.308 min (major).

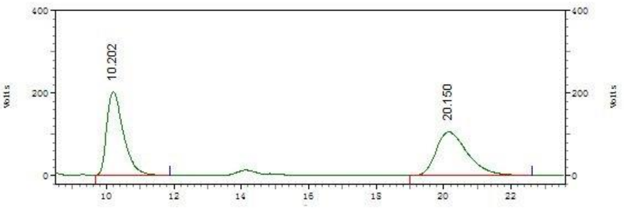

RetTime (min)	Width (min)	Height (Volts)	Area	Area (%)
10.202	2.21	805175	27587092	50.172
20.150	3.65	422075	27398300	49.828

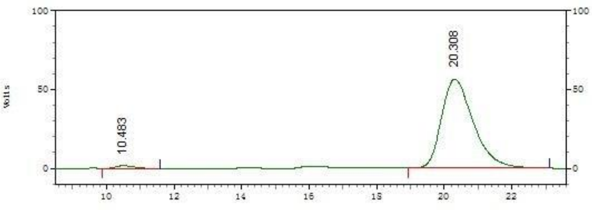

RetTime (min)	Width (min)	Height (Volts)	Area	Area (%)
10.483	1.71	7335	292604	1.947
20.308	4.18	225011	14739402	98.053

(*R*)-1-(3-phenyl-1-tosyl-1*H*-pyrazol-5-yl) naphthalen-2-ol (4i)

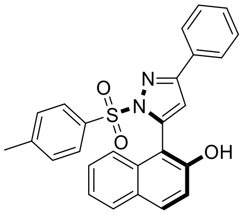

4i

Appearance: white solid.

Yield: 83%, 37.4mg. **ee** = 83%.

^{1}H NMR (400 MHz, acetone-*d*$_6$) δ 8.94 (s, 1H), 8.02 – 7.92 (m, 3H), 7.87 (d, J = 7.5 Hz, 1H), 7.68 (d, J = 8.2 Hz, 2H), 7.44 (dt, J = 23.8, 7.2 Hz, 3H), 7.36 – 7.23 (m, 5H), 7.15 (d, J = 8.0 Hz, 1H), 6.96 (s, 1H), 2.35 (s, 3H).

^{13}C NMR (100 MHz, acetone-*d*$_6$) δ 155.26, 154.80, 146.39, 143.44, 135.90, 135.09, 132.78, 132.05, 130.38, 129.91, 129.64, 129.16, 129.06, 128.84, 127.59, 126.92, 124.69, 123.82, 118.74, 110.82, 110.45, 21.53.

HRMS (ESI) m/z Calcd for $[C_{26}H_{20}N_2NaO_3S, M + Na]^+$: 463.1087, Found: 463.1084.

Optical Rotation: $[\alpha]_D^{25}$= 26° (*c* = 1.0, DCM).

HPLC analysis: Chiralcel IB-H (Hexane/*i*-PrOH = 70:30, flow rate = 1.0 mL/min, wave length = 254 nm), t_R = 6.626 min (minor), t_R = 8.053 min (major).

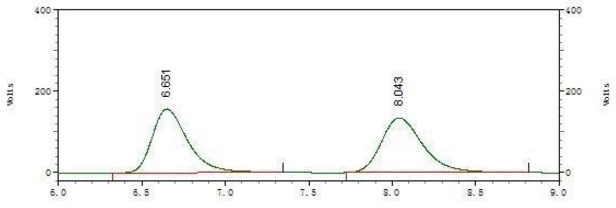

UV-WL1

RetTime (min)	Width (min)	Height (Volts)	Area	Area (%)
6.651	1.02	628089	9331719	50.038
8.043	1.09	537274	9317364	49.962

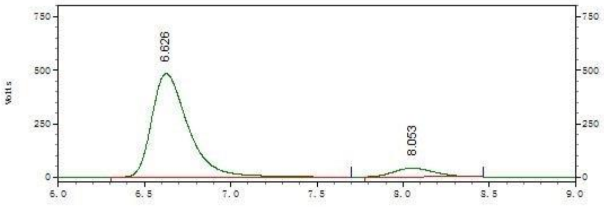

UV-WL1

RetTime (min)	Width (min)	Height (Volts)	Area	Area (%)
6.626	1.40	1934417	28504662	91.339
8.053	0.69	162668	2702839	8.661

(*R*)-1-(3-(tert-butyl)-1-(phenylsulfonyl)-1*H*-pyrazol-5-yl) naphthalen-2-ol (4j)

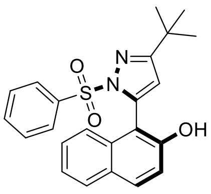

4j

Appearance: white solid.

Yield: 81%, 32.9 mg. **ee** = 91%.

^{1}H NMR (400 MHz, $CDCl_3$) δ 7.75 (d, *J* = 8.5 Hz, 1H), 7.67 (d, *J* = 7.5 Hz, 1H), 7.48 (d, *J* = 7.0 Hz, 2H), 7.40 – 7.31 (m, 1H), 7.27 – 7.10 (m, 4H), 7.07 (d, *J* = 7.2 Hz, 1H), 6.79 (d, *J* = 7.8 Hz, 1H), 6.29 (s, 1H), 1.28 (s, 9H).

^{13}C NMR (100 MHz, $CDCl_3$) δ 166.67, 152.52, 140.26, 137.08, 133.85, 133.49, 131.60, 128.61, 128.34, 128.02, 127.95, 126.77, 123.72, 123.36, 117.79, 111.28, 109.53, 32.80, 29.88.

HRMS (ESI) m/z Calcd for $[C_{23}H_{22}N_2NaO_3S, M + Na]^+$: 429.1243, Found: 429.1238.

Optical Rotation: $[\alpha]_D^{25}$= 31° (*c* = 1.0, DCM).

HPLC analysis: Chiralcel OD-H (Hexane/*i*-PrOH = 90:10, flow rate = 1.0 mL/min, wave length = 254 nm), t_R = 9.923 min (minor), t_R = 12.674 min (major).

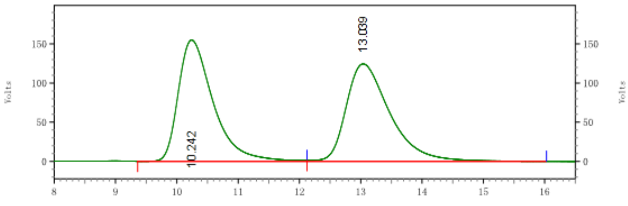

UV-WL1

RetTime (min)	Width (min)	Height (Volts)	Area	Area (%)
10.242	2.76	618525	24434691	49.849
13.039	3.90	496995	24582643	50.151

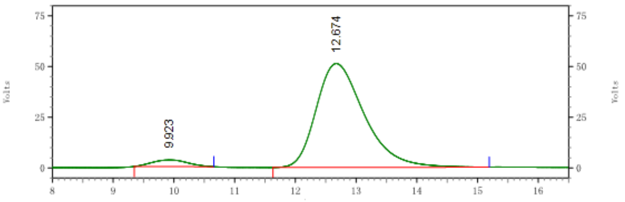

UV-WL1

RetTime (min)	Width (min)	Height (Volts)	Area	Area (%)
9.923	1.31	13026	532905	4.508
12.674	3.56	204845	11287833	95.492

(*R*)-1-(1-(phenylsulfonyl)-3-(p-tolyl)-1*H*-pyrazol-5-yl) naphthalen-2-ol (4k)

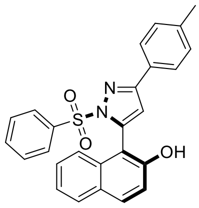

4k

Appearance: white solid.

Yield: 81%, 35.7 mg. **ee** = 84%.

^{1}H NMR (400 MHz, acetone-d_6) δ 8.99 (s, 1H), 7.95 (d, J = 8.9 Hz, 1H), 7.86 (dt, J = 8.1, 2.4 Hz, 3H), 7.82 (d, J = 8.1 Hz, 2H), 7.65 (t, J = 7.4 Hz, 1H), 7.48 (t, J = 7.4 Hz, 2H), 7.37 – 7.25 (m, 5H), 7.17 (d, J = 8.0 Hz, 1H), 6.94 (s, 1H), 2.36 (s, 3H).

^{13}C NMR (100 MHz, acetone-d_6) 155.58, 154.76, 143.57, 139.88, 138.89, 135.10, 132.05, 130.25, 129.93, 129.86, 129.06, 128.85, 127.63, 126.91, 124.66, 123.86, 118.71, 110.87, 110.45, 29.85, 21.32.

HRMS (ESI) m/z Calcd for $[C_{26}H_{20}N_2NaO_3S, M + Na]^+$: 463.1087, Found: 463.1080.

Optical Rotation: $[\alpha]_D^{25}$= 27° (*c* = 1.0, DCM).

HPLC analysis: Chiralcel IB-H (Hexane/*i*-PrOH = 90:10, flow rate = 1.0 mL/min, wave length = 254 nm), t_R = 26.136 min (major), t_R =32.041 min (minor).

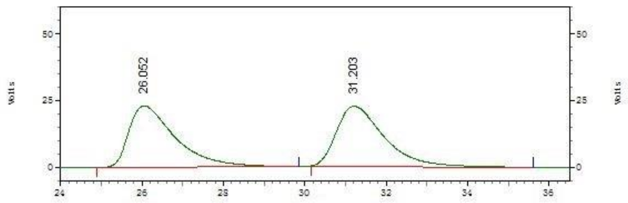

UV-WL1

RetTime (min)	Width (min)	Height (Volts)	Area	Area (%)
26.052	4.95	92615	7389757	50.317
31.203	5.47	89821	7296731	49.683

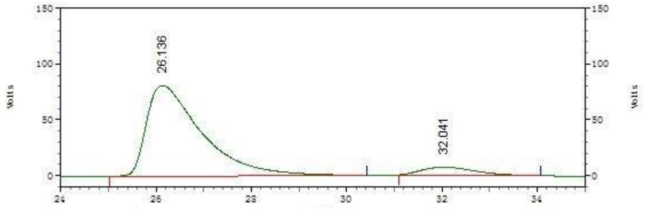

UV-WL1

RetTime (min)	Width (min)	Height (Volts)	Area	Area (%)
26.136	5.42	325757	26232507	92.194
32.041	2.97	28809	2220994	7.806

(*R*)-1-(1-(mesitylsulfonyl)-3-(p-tolyl)-1*H*-pyrazol-5-yl) naphthalen-2-ol (4l)

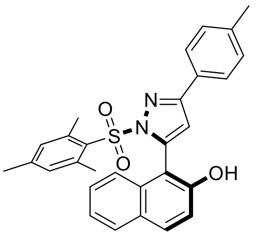

4l

Appearance: white solid.

Yield: 84%, 40.8 mg. **ee** = 94%.

^{1}H NMR (400 MHz, acetone-d_6) δ 8.90 (s, 1H), 7.91 – 7.83 (m, 3H), 7.78 (d, *J* = 8.0 Hz, 1H), 7.28 (d, *J* = 7.8 Hz, 2H), 7.24 (d, *J* = 6.8 Hz, 1H), 7.21 (s, 1H), 7.18 (d, *J* = 6.9 Hz, 1H), 7.12 (d, *J* = 8.3 Hz, 1H), 6.91 (s, 1H), 6.74 (s, 2H), 2.36 (s, 3H), 2.26 (s, 6H), 2.16 (s, 3H).

^{13}C NMR (100 MHz, acetone-d_6) 155.00, 153.29, 145.04, 142.14, 141.69, 139.47, 134.83, 133.07, 132.51, 132.18, 130.20, 128.85, 128.53, 127.44, 126.74, 124.63, 123.55, 118.63, 110.39, 109.49, 22.62, 21.31, 20.97.

HRMS (ESI) m/z Calcd for $[C_{29}H_{26}N_2NaO_3S, M + Na]^+$: 505.1556, Found: 505.1559.

Optical Rotation: $[\alpha]_D^{25}$= 29° (*c* = 1.0, DCM).

HPLC analysis: Chiralcel OD-H (Hexane/*i*-PrOH = 90:10, flow rate = 1.0 mL/min, wave length = 254 nm), t_R = 7.874 min (major), t_R = 13.292 min (minor).

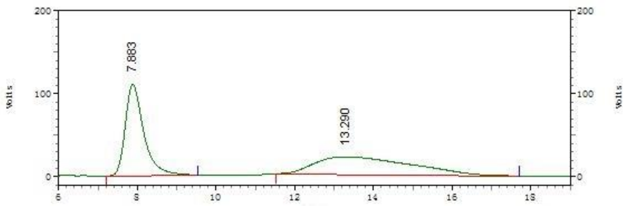

UV-WL1

RetTime (min)	Width (min)	Height (Volts)	Area	Area (%)
7.883	2.31	442395	14455883	50.303
13.290	6.17	84368	14281766	49.697

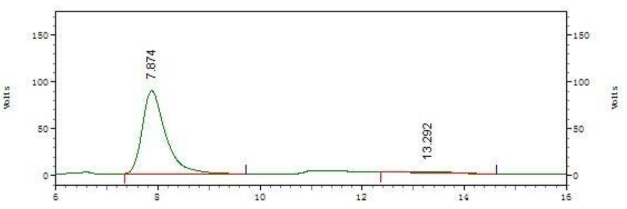

UV-WL1

RetTime (min)	Width (min)	Height (Volts)	Area	Area (%)
7.874	2.38	354843	11370220	96.974
13.292	2.29	5357	354849	3.026

(*R*)-1-(3-(tert-butyl)-1-((4-(trifluoromethyl) phenyl) sulfonyl)-1*H*-pyrazol-5-yl) naphthalen-2-ol (4m)

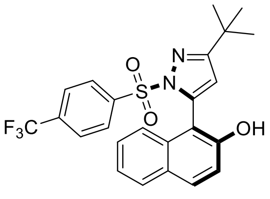

4m

Appearance: white solid.

Yield: 80%, 37.9 mg. **ee** = 89%.

^{1}H NMR (400 MHz, $CDCl_3$) δ 7.79 (d, *J* = 8.9 Hz, 1H), 7.69 (d, *J* = 8.1 Hz, 1H), 7.57 (d, *J* = 8.1 Hz, 2H), 7.39 (d, *J* = 8.2 Hz, 2H), 7.24 – 7.13 (m, 2H), 7.03 (t, *J* = 7.6 Hz, 1H), 6.65 (d, *J* = 8.4 Hz, 1H), 6.35 (s, 1H), 1.31 (s, 9H).

^{13}C NMR (100 MHz, $CDCl_3$) δ 167.15, 152.55, 140.42, 140.20, 135.32 (q, *J* = 32.6 Hz), 133.33, 131.92, 128.62, 128.39, 128.11, 126.99, 125.75 (q, *J* = 3.0 Hz) 123.61, 123.26, 122.88 (q, *J* = 272.0 Hz), 117.80, 111.78, 109.04, 32.93, 29.87, 29.68.

HRMS (ESI) m/z Calcd for $[C_{24}H_{21}F_3N_2NaO_3S, M + Na]^+$: 497.1117, Found: 497.1110.

Optical Rotation: $[\alpha]_D^{25}$= 34° (*c* = 1.0, DCM).

HPLC analysis: Chiralcel OD-H (Hexane/*i*-PrOH = 85:15, flow rate = 1.0 mL/min, wave length = 254 nm), t_R = 7.457 min (major), t_R = 12.811 min (minor).

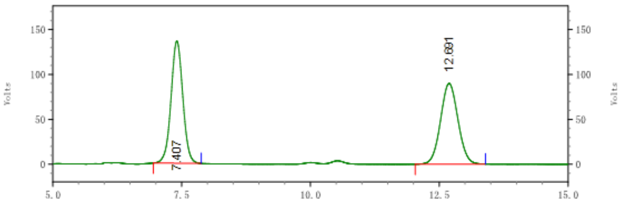

UV-WL1

RetTime (min)	Width (min)	Height (Volts)	Area	Area (%)
7.407	0.93	544602	8680125	50.655
12.691	1.37	359435	8455791	49.345

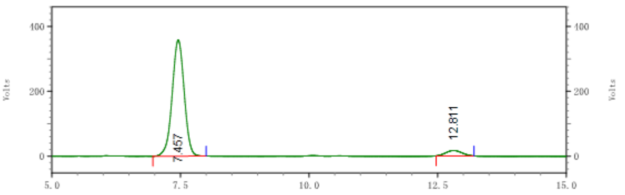

UV-WL1

RetTime (min)	Width (min)	Height (Volts)	Area	Area (%)
7.457	1.04	1432692	23316875	94.230
12.811	0.74	66254	1427783	5.770

(*R*)-1-(3-(tert-butyl)-1-((4-chlorophenyl) sulfonyl)-1*H*-pyrazol-5-yl) naphthalen-2-ol (4n)

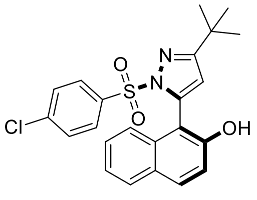

4n

Appearance: white solid.

Yield: 77%, 33.9 mg. **ee** = 94%.

^{1}H NMR (400 MHz, $CDCl_3$) δ 7.87 (d, *J* = 8.9 Hz, 1H), 7.78 (d, *J* = 8.1 Hz, 1H), 7.47 (d, *J* = 8.5 Hz, 2H), 7.31 (t, *J* = 7.4 Hz, 1H), 7.23 – 7.13 (m, 3H), 6.82 (d, *J* = 8.4 Hz, 1H), 6.41 (s, 1H), 5.39 (s, 1H), 1.38 (s, 9H).

^{13}C NMR (100 MHz, $CDCl_3$) δ 166.93, 152.29, 140.76, 139.76, 135.38, 133.37, 131.87, 129.54, 129.00, 128.46, 128.10, 126.98, 123.59, 117.71, 111.64, 109.27, 32.91, 29.91.

HRMS (ESI) m/z Calcd for $[C_{23}H_{21}ClN_2NaO_3S, M + Na]^+$: 463.0854, Found: 463.0855.

Optical Rotation: $[\alpha]_D^{25}$= 40° (*c* = 1.0, DCM).

HPLC analysis: Chiralcel OD-H (Hexane/*i*-PrOH = 90:10, flow rate = 1.0 mL/min, wave length = 254 nm), t_R = 6.713 min (minor), t_R = 10.699 min (major).

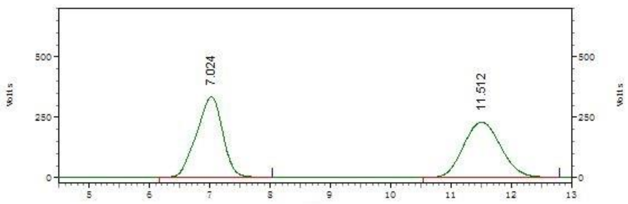

RetTime (min)	Width (min)	Height (Volts)	Area	Area (%)
7.024	1.88	1331454	39933085	50.356
11.512	2.27	916432	39368410	49.644

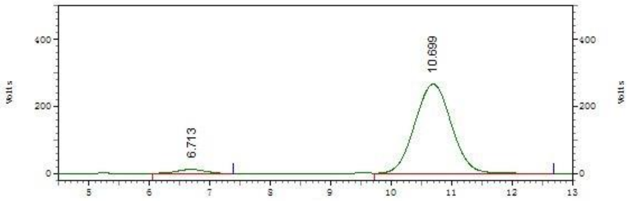

RetTime (min)	Width (min)	Height (Volts)	Area	Area (%)
6.713	1.33	44764	1479288	3.205
10.699	2.96	1063319	44680849	96.795

(*R*)-7-bromo-1-(3-(*tert*-butyl)-1-((4-chlorophenyl) sulfonyl)-1*H*-pyrazol-5-yl) naphthalen-2-ol (4o)

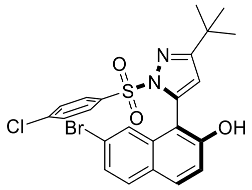

4o

Appearance: yellow solid.

Yield: 80%, 41.5 mg. **ee** = 87%.

^{1}H NMR (400 MHz, $CDCl_3$) δ 7.84 (d, *J* = 8.9 Hz, 1H), 7.64 (d, *J* = 8.6 Hz, 1H), 7.44 (d, *J* = 8.4 Hz, 2H), 7.38 (d, *J* = 8.4 Hz, 1H), 7.25 (d, *J* = 10.7 Hz, 4H), 6.76 (s, 1H), 6.42 (s, 1H), 5.42 (s, 1H), 1.40 (s, 9H).

^{13}C NMR (100 MHz, $CDCl_3$) δ 166.98, 153.28, 141.10, 138.76, 135.07, 134.48, 131.79, 129.72, 129.30, 129.20, 127.03, 126.79, 125.75, 121.67, 118.24, 111.89, 108.51, 32.95, 29.90.

HRMS (ESI) m/z Calcd for $[C_{23}H_{20}BrClN_2NaO_3S, M + Na]^+$: 540.9959, Found: 540.9952.

Optical Rotation: $[\alpha]_D^{25}$= 26° (*c* = 1.0, DCM).

HPLC analysis: Chiralcel OD-H (Hexane/*i*-PrOH = 85:15, flow rate = 1.0 mL/min, wave length = 254 nm), t_R = 4.807 min (minor), t_R = 5.703 min (major).

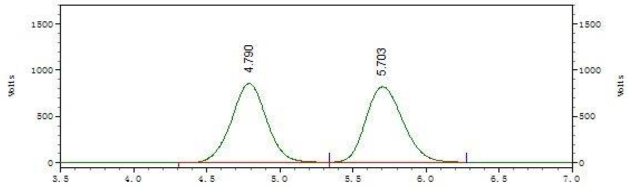

UV-WL1

RetTime (min)	Width (min)	Height (Volts)	Area	Area (%)
4.790	1.03	3423005	57957746	50.307
5.703	0.93	3254423	57251486	49.693

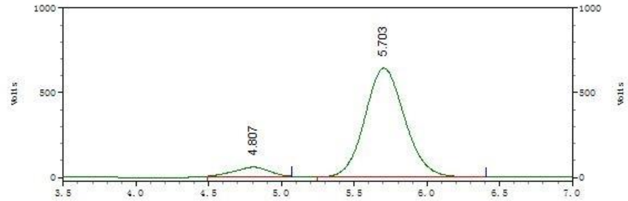

UV-WL1

RetTime (min)	Width (min)	Height (Volts)	Area	Area (%)
4.807	0.58	217431	3622882	6.886
5.703	1.16	2570052	48987645	93.114

(*R*)-5-(4-iodo-1,1-diphenyl-1*H*-benzo[c] [1,2] oxasilin-3-yl) quinolin-6-ol (4p)

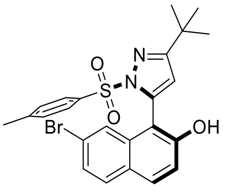

4p

Appearance: white solid.

Yield: 83%, 41.4 mg. **ee** = 90%.

^{1}H NMR (400 MHz, $CDCl_3$) δ 7.81 (d, *J* = 8.8 Hz, 1H), 7.61 (d, *J* = 8.5 Hz, 1H), 7.33 (t, *J* = 7.0 Hz, 3H), 7.27 (d, *J* = 5.9 Hz, 1H), 7.01 (d, *J* = 7.7 Hz, 2H), 6.70 (s, 1H), 6.38 (s, 1H), 5.65 (s, 1H), 2.33 (s, 3H), 1.40 (s, 9H).

^{13}C NMR (100 MHz, $CDCl_3$) δ 166.42, 153.41, 145.46, 138.60, 134.52, 133.58, 131.60, 129.55, 129.40, 127.78, 126.66, 125.82, 121.35, 118.36, 111.61, 108.80, 32.86, 29.92, 21.68.

HRMS (ESI) m/z Calcd for $[C_{24}H_{23}BrN_2NaO_3S, M + Na]^+$: 521.0505, Found: 521.0502.

Optical Rotation: $[\alpha]_D^{25}$= 43° (*c* = 1.0, acetone).

HPLC analysis: Chiralcel OD-H (Hexane/*i*-PrOH = 85:15, flow rate = 1.0 mL/min, wave length = 254 nm), t_R = 5.151 min (minor), t_R =7.005 min (major).

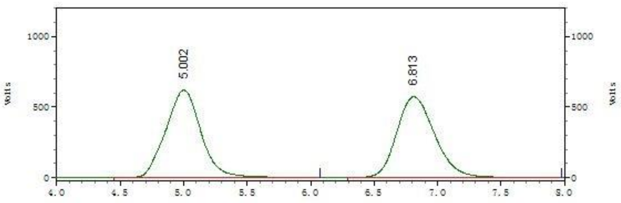

RetTime (min)	Width (min)	Height (Volts)	Area	Area (%)
5.002	1.62	2472487	47644441	50.148
6.813	1.69	2284157	47363563	49.852

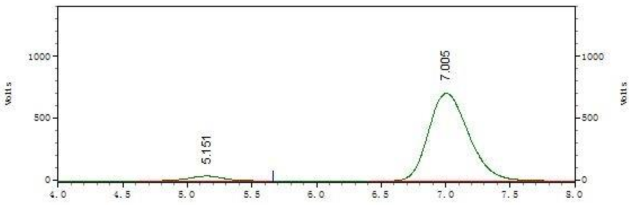

RetTime (min)	Width (min)	Height (Volts)	Area	Area (%)
5.151	1.05	158182	3080876	4.765
7.005	1.89	2841143	61579620	95.235

General procedure for the asymmetric synthesis of compounds (*S*)-4q-4aa

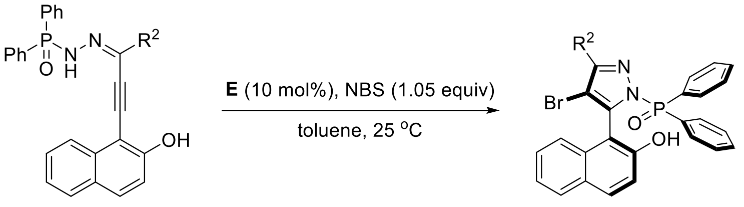

3q-3aa (*S*)-**4q-4aa**

A flame-dried Schlenk tube equipped with a magnetic stirring bar, was charged with **3q-3aa** (0.1 mmol, 1.0 equiv), NBS (0.105 mmol, 1.05 equiv), and catalyst **E** (20 mol%), toluene (2.0 mL) was injected into the tube at 25 ºC. After stirring for 1 h, the mixture was filtered and purified by silica gel chromatography (PE/EA = 3:1) to afford the product (*S*)-**4q-4aa**. Racemic samples were prepared without the addition of the catalyst.

Analytical dates of (*S*)-4q-4aa

(*S*)-(4-bromo-3-(*tert*-butyl)-5-(2-hydroxynaphthalen-1-yl)-1*H*-pyrazol-1-yl) diphenylphosphine oxide (4q)

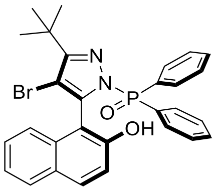

4q

Appearance: white solid.

Yield: 74%, 40.2 mg. **ee** = 94%.

^{1}H NMR (400 MHz, $CDCl_3$) δ 7.83 (dd, *J* = 13.0, 8.1 Hz, 2H), 7.47 (dd, *J* = 8.1, 4.2 Hz, 3H), 7.42 – 7.32 (m, 4H), 7.14 – 7.07 (m, 2H), 6.97 (dd, *J* = 12.7, 8.2 Hz, 2H), 6.92 – 6.86 (m, 1H), 6.81 (td, *J* = 7.5, 3.8 Hz, 2H), 1.36 (s, 9H).

^{13}C NMR (100 MHz, $CDCl_3$) δ154.26, 144.02, 143.95, 133.01, 132.90, 132.77, 132.74, 132.20, 131.93, 131.90, 131.57, 131.50, 131.46, 129.21, 128.46, 128.02, 127.88, 127.84, 127.71, 127.57, 126.54, 126.24, 124.04, 123.11, 120.48, 110.88, 109.98, 100.20, 33.81, 28.54.

HRMS (ESI) m/z Calcd for $[C_{29}H_{26}BrN_2NaO_2P, M + Na]^+$: 567.0807, Found: 567.0802.

Optical Rotation: $[\alpha]_D^{25}$= -40.4° (*c* = 1.0, DCM).

HPLC analysis: Chiralcel AD-H (Hexane/*i*-PrOH = 90:10, flow rate = 1.0 mL/min, wave length = 254 nm), t_R = 7.138 min (minor), t_R = 12.005 min (major).

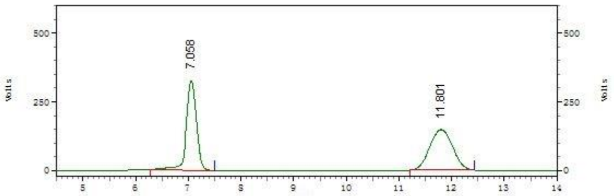

UV-WL1

RetTime (min)	Width (min)	Height (Volts)	Area	Area (%)
7.058	1.22	1306921	17249308	49.138
11.801	1.21	591839	17854169	50.862

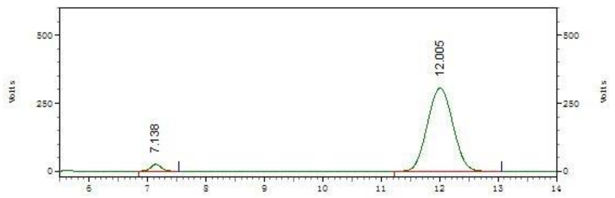

UV-WL1

RetTime (min)	Width (min)	Height (Volts)	Area	Area (%)
7.138	0.68	98918	1215430	3.189
12.005	1.83	1223439	36899078	96.811

(*S*)-(4-bromo-5-(2-hydroxynaphthalen-1-yl)-3-(pentan-3-yl)-1*H*-pyrazol-1-yl) diphenylphosphine oxide (4r)

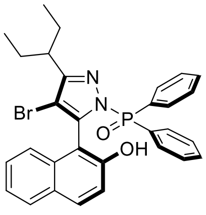

4r

Appearance: white solid.

Yield: 72%, 40.17 mg. **ee** = 97%.

^{1}H NMR (400 MHz, $CDCl_3$) δ 7.80 (dd, *J* = 12.9, 7.8 Hz, 2H), 7.50 (d, *J* = 8.2 Hz, 2H), 7.37 (dd, *J* = 12.8, 7.0 Hz, 4H), 7.23 – 7.17 (m, 1H), 7.12 (s, 2H), 7.01 (s, 2H), 6.90 (d, *J* = 23.8 Hz, 3H), 2.71 (t, *J* = 6.3 Hz, 1H), 1.69 (dq, *J* = 20.7, 6.4 Hz, 4H), 0.84 (q, *J* = 7.2 Hz, 6H).

^{13}C NMR (100 MHz, $CDCl_3$) δ 158.34, 154.08, 142.57, 132.88, 132.84, 132.82, 132.78, 132.21, 132.05, 132.02, 131.57, 131.46, 128.61, 128.13, 127.99, 127.90, 127.80, 127.66, 126.33, 123.94, 123.22, 120.53, 111.01, 102.94, 41.19, 26.34, 11.89.

HRMS (ESI) m/z Calcd for $[C_{30}H_{28}BrN_2NaO_2P, M + Na]^+$: 581.0964, Found: 581.0969.

Optical Rotation: $[\alpha]_D^{25}$= 44° (*c* = 1.0, DCM).

HPLC analysis: Chiralcel AD-H (Hexane/*i*-PrOH = 90:10, flow rate = 1.0 mL/min, wave length = 254 nm), t_R = 21.543 (minor), t_R =30.158 min (major).

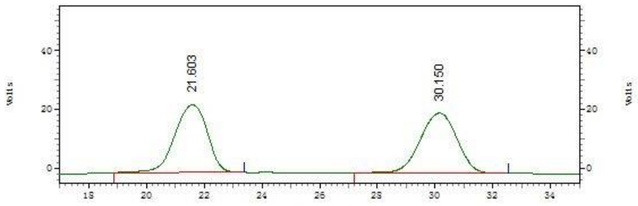

UV-WL1

RetTime (min)	Width (min)	Height (Volts)	Area	Area (%)
21.603	4.49	91818	7206137	50.136
30.150	5.33	81420	7167133	49.864

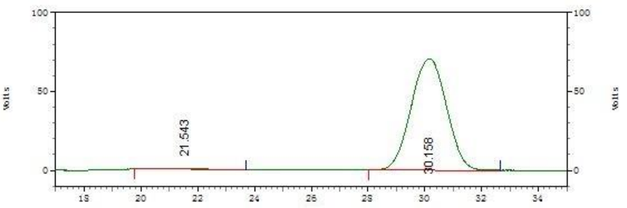

UV-WL1

RetTime (min)	Width (min)	Height (Volts)	Area	Area (%)
21.543	3.93	3486	292291	1.182
30.158	4.64	282039	24432957	98.818

(*S*)-(4-bromo-3-cyclopropyl-5-(2-hydroxynaphthalen-1-yl)-1*H*-pyrazol-1-yl) diphenylphosphine oxide (4s)

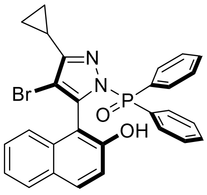

4s

Appearance: yellow solid.

Yield: 75%, 39.6 mg. **ee** = 94%.

^{1}H NMR (400 MHz, $CDCl_3$) δ 7.77 (dd, *J* = 8.1, 4.2 Hz, 1H), 7.62 (dd, *J* = 12.0, 7.8 Hz, 4H), 7.41 – 7.37 (m, 4H), 7.37 – 7.32 (m, 3H), 7.23 (dd, *J* = 7.9, 3.2 Hz, 3H), 7.19 (d, *J* = 9.0 Hz, 1H), 1.92 (p, *J* = 7.1 Hz, 1H), 0.99 (d, *J* = 6.8 Hz, 4H).

^{13}C NMR (100 MHz, $CDCl_3$) δ 153.28, 148.79, 141.60, 134.24, 132.85, 132.48, 131.55, 131.41, 131.39, 131.14, 131.04, 128.64, 128.23, 128.14, 128.10, 126.77, 124.66, 123.48, 118.39, 108.56, 96.79, 7.32, 7.14.

HRMS (ESI) m/z Calcd for $[C_{28}H_{22}BrN_2NaO_2P, M + Na]^+$: 551.0494, Found: 551.0490

Optical Rotation: $[\alpha]_D^{25}$= 36.2° (*c* = 1.0, DCM).

HPLC analysis: Chiralcel AD-H (Hexane/*i*-PrOH = 90:10, flow rate = 1.0 mL/min, wave length = 254 nm), t_R = 14.733 min (minor)., t_R = 24.682 min (major).

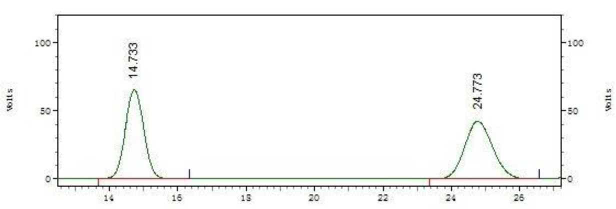

UV-WL1

RetTime (min)	Width (min)	Height (Volts)	Area	Area (%)
14.733	2.69	259686	9739575	50.136
24.773	3.19	168972	9686589	49.864

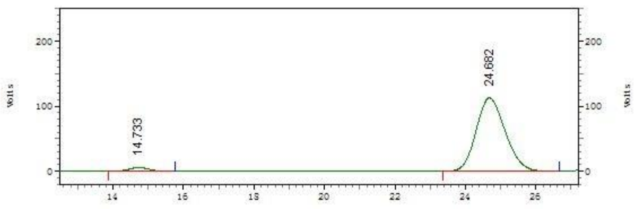

UV-WL1

RetTime (min)	Width (min)	Height (Volts)	Area	Area (%)
14.733	1.88	21356	810248	3.042
24.682	3.32	450333	25821826	96.958

(*S*)-(4-bromo-3-cyclobutyl-5-(2-hydroxynaphthalen-1-yl)-1*H*-pyrazol-1-yl) diphenylphosphine oxide (4t)

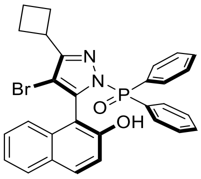

4t

Appearance: white solid.

Yield: 75%, 40.6 mg. **ee** = 98%.

^{1}H NMR (400 MHz, DMSO-*d*$_6$) δ 9.91 (s, 1H), 7.73 (t, *J* = 8.7 Hz, 2H), 7.65 – 7.47 (m, 6H), 7.46 – 7.34 (m, 4H), 7.25 (p, *J* = 5.8 Hz, 2H), 7.05 (d, *J* = 8.9 Hz, 1H), 6.97 (d, *J* = 7.8 Hz, 1H), 3.61 (p, *J* = 8.4 Hz, 1H), 2.37 – 2.20 (m, 4H), 2.10 – 1.96 (m, 1H), 1.85 (dh, *J* = 11.7, 4.0 Hz, 1H).

^{13}C NMR (100 MHz, DMSO-*d*$_6$) δ 153.94, 135.64, 134.30, 133.60, 131.36, 130.89, 130.79, 130.39, 128.42, 128.29, 127.86, 127.49, 126.60, 123.77, 122.68, 118.10, 93.34, 31.74, 27.83, 18.14.

HRMS (ESI) m/z Calcd for $[C_{29}H_{24}BrN_2NaO_2P, M + Na]^+$: 565.0651, Found: 565.0657.

Optical Rotation: $[\alpha]_D^{25}$= 102.0° (*c* = 0.15, DCM).

HPLC analysis: Chiralcel AD-H (Hexane/*i*-PrOH = 80:20, flow rate = 1.0 mL/min, wave length = 254 nm), t_R = 6.074 min (minor), t_R = 8.322 min (major).

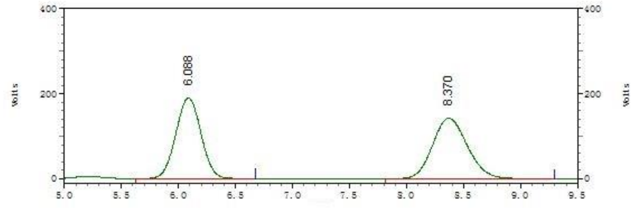

UV-WL1

RetTime (min)	Width (min)	Height (Volts)	Area	Area (%)
6.088	1.05	758299	11639196	49.160
8.370	1.48	567687	12037115	50.840

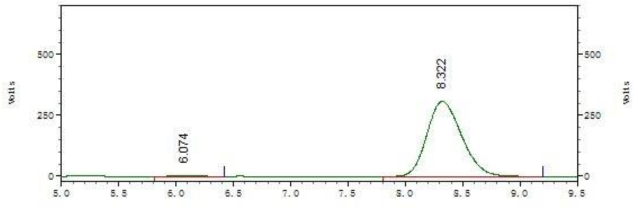

UV-WL1

RetTime (min)	Width (min)	Height (Volts)	Area	Area (%)
6.074	0.61	11333	173067	0.660
8.322	1.40	1235121	26048076	99.340

(*S*)-(4-bromo-3-cyclohexyl-5-(2-hydroxynaphthalen-1-yl)-1*H*-pyrazol-1-yl) diphenylphosphine oxide (4u)

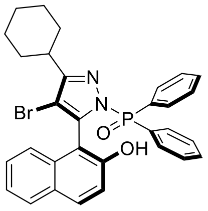

4u

Appearance: white solid.

Yield: 75%, 42.8 mg. **ee** = 86%.

^{1}H NMR (400 MHz, $CDCl_3$) δ 7.91 (dd, *J* = 12.9, 7.7 Hz, 2H), 7.61 (dd, *J* = 21.5, 8.3 Hz, 3H), 7.52 – 7.34 (m, 4H), 7.22 – 7.16 (m, 2H), 7.03 (t, *J* = 7.4 Hz, 1H), 6.98 – 6.80 (m, 3H), 2.79 (t, *J* = 11.1 Hz, 1H), 2.00 (s, 2H), 1.81 (d, *J* = 11.4 Hz, 2H), 1.70 (d, *J* = 10.4 Hz, 1H), 1.50 (d, *J* = 13.9 Hz, 1H), 1.46 – 1.19 (m, 4H).

^{13}C NMR (100 MHz, $CDCl_3$) δ 159.59, 153.93, 142.27, 133.11, 133.00, 132.93, 132.12, 131.96, 131.75, 131.45, 131.35, 128.85, 128.13, 127.99, 127.85, 127.72, 126.25, 124.29, 123.45, 121.18, 111.91, 36.90, 31.06, 26.20, 26.02.

HRMS (ESI) m/z Calcd for $[C_{31}H_{28}BrN_2NaO_2P, M + Na]^+$: 593.0964, Found: 593.0968.

Optical Rotation: $[\alpha]_D^{25}$= 43.2° (*c* = 1.0, acetone).

HPLC analysis: Chiralcel AD-H (Hexane/*i*-PrOH = 90:10, flow rate = 1.0 mL/min, wave length = 254 nm), t_R = 11.661 min (minor), t_R =14.895 min (major).

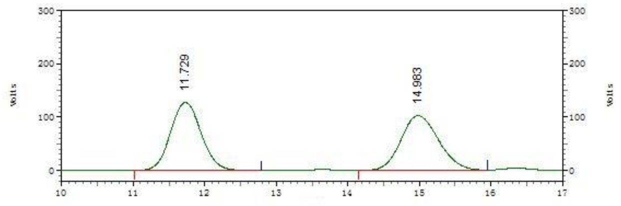

UV-WL1

RetTime (min)	Width (min)	Height (Volts)	Area	Area (%)
11.729	1.76	509707	15084740	50.207
14.983	1.80	410200	14960537	49.793

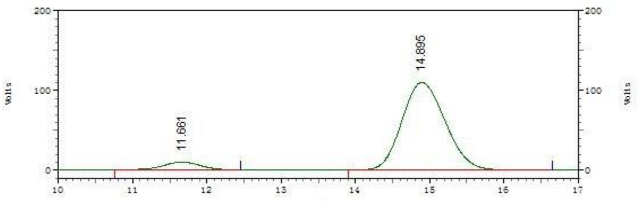

UV-WL1

RetTime (min)	Width (min)	Height (Volts)	Area	Area (%)
11.661	1.70	37532	1258857	6.641
14.895	2.76	439380	17697362	93.359

(*S*)-(3-((3*R*,5*R*,7*R*)-adamantan-1-yl)-4-bromo-5-(2-hydroxynaphthalen-1-yl)-1*H*-pyrazol-1-yl) diphenylphosphine oxide (4v)

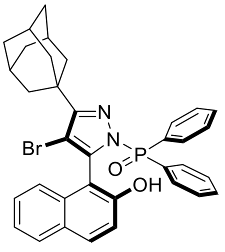

4v

Appearance: white solid.

Yield: 77%, 44.0 mg. **ee** = 88%.

^{1}H NMR (400 MHz, $CDCl_3$) δ 7.94 (dd, *J* = 13.1, 7.7 Hz, 2H), 7.69 – 7.53 (m, 3H), 7.49 (td, *J* = 7.5, 3.7 Hz, 2H), 7.42 (dd, *J* = 13.0, 7.6 Hz, 2H), 7.20 (dd, *J* = 9.2, 5.8 Hz, 3H), 7.00 (t, *J* = 7.5 Hz, 1H), 6.96 – 6.91 (m, 1H), 6.88 (dt, *J* = 11.5, 5.6 Hz, 2H), 2.14 (m, *J* = 2.8 Hz, 5H), 2.06 (m, 3H), 1.76 (m, *J* = 3.1 Hz, 5H), 1.56 (m, 2H).

^{13}C NMR (100 MHz, $CDCl_3$) δ 153.84, 133.20, 133.09, 132.96, 132.94, 132.08, 131.87, 131.84, 131.80, 131.40, 131.29, 128.91, 128.08, 127.94, 127.80, 127.66, 126.26, 124.36, 123.54, 121.42, 112.38, 39.61, 36.76, 36.06, 28.41.

HRMS (ESI) m/z Calcd for $[C_{35}H_{32}BrN_2NaO_2P, M + Na]^+$: 645.1277, Found: 645.1272.

Optical Rotation: $[\alpha]_D^{25}$= 38.0° (*c* = 1.0, acetone).

HPLC analysis: Chiralcel AD-H (Hexane/*i*-PrOH = 90:10, flow rate = 1.0 mL/min, wave length = 254 nm), t_R = 8.804 min (minor), t_R = 11.583 min (major).

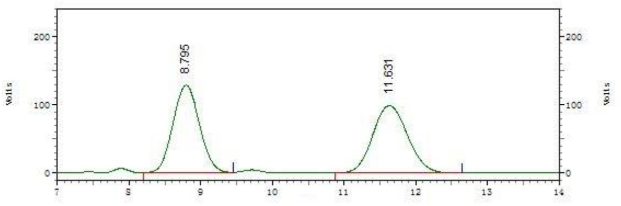

UV-WL1

RetTime (min)	Width (min)	Height (Volts)	Area	Area (%)
8.795	1.24	511704	13070381	49.561
11.631	1.77	393493	13302083	50.439

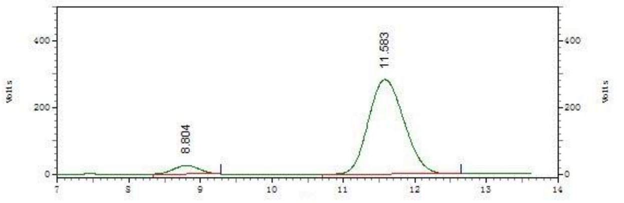

UV-WL1

RetTime (min)	Width (min)	Height (Volts)	Area	Area (%)
8.804	0.93	101447	2527771	6.076
11.583	1.94	1132810	39076021	93.924

(*S*)-(4-bromo-5-(2-hydroxynaphthalen-1-yl)-3-(p-tolyl)-1*H*-pyrazol-1-yl) diphenylphosphine oxide (4w)

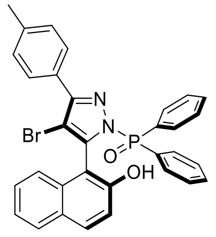

4w

Appearance: white solid.

Yield: 78%, 45.0 mg. **ee** = 89%.

^{1}H NMR (400 MHz, acetone-*d*$_6$) δ 8.78 (s, 1H), 7.88 – 7.69 (m, 8H), 7.57 (t, *J* = 7.4 Hz, 1H), 7.50 – 7.41 (m, 3H), 7.34 (td, *J* = 7.5, 3.6 Hz, 2H), 7.28 (dd, *J* = 11.0, 5.4 Hz, 4H), 7.21 – 7.13 (m, 2H), 2.38 (s, 3H).

^{13}C NMR (100 MHz, acetone-*d*$_6$), 155.12, 139.66, 133.62, 133.60, 133.42, 133.40, 133.30, 133.19, 132.98, 132.87, 132.40, 129.89, 129.33, 129.12, 129.03, 128.99, 128.90, 128.81, 128.61, 127.43, 124.87, 123.87, 119.49, 119.32, 21.31.

HRMS (ESI) m/z Calcd for [$C_{32}H_{24}BrN_2NaO_2P$, M + Na]$^+$: 601.0651, Found: 601.0657.

Optical Rotation: $[\alpha]_D^{25}$= 55.2° (*c* = 1.0, acetone).

HPLC analysis: Chiralcel AD-H (Hexane/*i*-PrOH = 85:15, flow rate = 1.0 mL/min, wave length = 254 nm), t_R = 11.456 min (major), t_R = 13.226 min (minor).

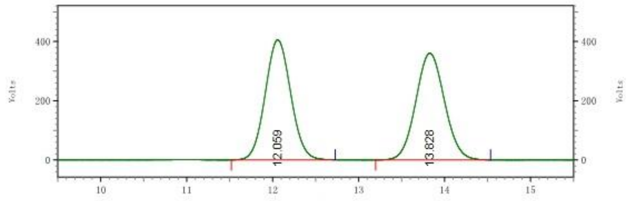

UV-WL1

RetTime (min)	Width (min)	Height (Volts)	Area	Area (%)
12.059	1.21	1620607	33507382	50.053
13.828	1.34	1440536	33436579	49.947

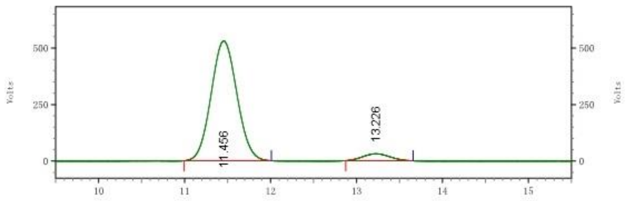

UV-WL1

RetTime (min)	Width (min)	Height (Volts)	Area	Area (%)
11.456	1.01	2120914	44375529	94.287
13.226	0.78	122962	2688932	5.713

(*S*)-(4-bromo-3-cyclopropyl-5-(2-hydroxy-7-methoxynaphthalen-1-yl)-1H-pyrazol-1-yl) diphenylphosphine oxide (4x)

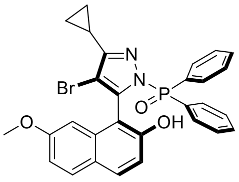

4x

Appearance: white solid.

Yield: 70%, 39 mg. **ee** = 96%.

^{1}H NMR (400 MHz, acetone-*d*$_6$) δ 7.75 (dt, *J* = 23.6, 11.9 Hz, 5H), 7.60 (s, 4H), 7.51 – 7.44 (m, 1H), 7.38 (s, 2H), 7.12 (d, *J* = 8.7 Hz, 1H), 6.98 (d, *J* = 8.4 Hz, 1H), 6.81 (s, 1H), 3.74 (s, 3H), 1.00 (d, *J* = 6.6 Hz, 4H).

^{13}C NMR (100 MHz, acetone-*d*$_6$) 159.55, 155.47, 149.95, 140.67, 135.91, 134.45, 132.44, 132.42, 131.98, 131.88, 131.52, 130.47, 129.26, 129.13, 124.71, 116.30, 116.00, 109.48, 104.56, 96.23, 55.39, 8.13, 7.68.

HRMS (ESI) m/z Calcd for [$C_{29}H_{24}BrN_2NaO_3P$, M + Na]$^+$: 581.0600, Found: 581.0605.

Optical Rotation: $[\alpha]_D^{25}$= 45.4° (*c* = 1.0, acetone).

HPLC analysis: Chiralcel AD-H (Hexane/*i*-PrOH = 85:15, flow rate = 1.0 mL/min, wave length = 254 nm), t_R = 9.827 min (minor), t_R = 20.249 min (major).

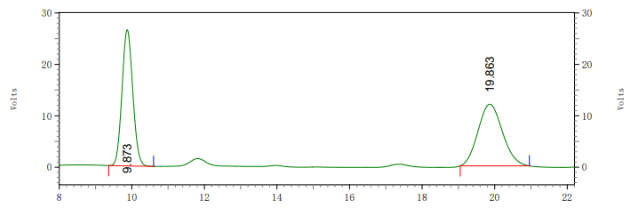

UV-WL1 254 nm

RetTime (min)	Width (min)	Height (Volts)	Area	Area (%)
9.873	1.24	105882	2196393	49.442
19.863	1.91	47921	2245945	50.558

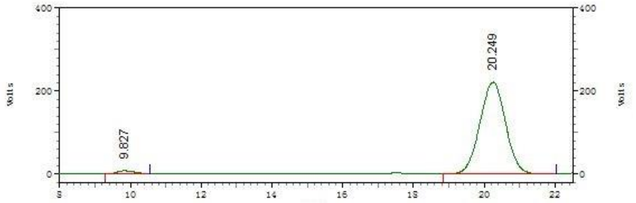

UV-WL1

RetTime (min)	Width (min)	Height (Volts)	Area	Area (%)
9.827	1.27	30721	1045589	2.294
20.249	3.19	883868	44539516	97.706

(*S*)-(4-bromo-5-(7-bromo-2-hydroxynaphthalen-1-yl)-3-(tert-butyl)-1*H*-pyrazol-1-yl) diphenylphosphine oxide (4y)

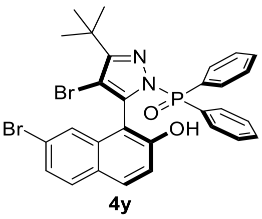

4y

Appearance: white solid.

Yield: 98%, 50.7 mg. **ee** = 86%.

^{1}H NMR (400 MHz, acetone-d_6) δ 9.17 (s, 1H), 7.89 – 7.74 (m, 3H), 7.73 – 7.57 (m, 4H), 7.54 – 7.42 (m, 3H), 7.40 – 7.29 (m, 3H), 7.16 (d, *J* = 8.4 Hz, 2H), 1.44 (s, 9H).

^{13}C NMR (100 MHz, $CDCl_3$) δ 154.57, 143.24, 143.16, 133.03, 132.93, 132.23, 131.59, 131.48, 130.79, 130.60, 129.47, 128.11, 127.97, 127.83, 125.91, 121.93, 116.92, 111.49, 100.42, 33.84, 28.51.

HRMS (ESI) m/z Calcd for $[C_{29}H_{25}Br_2N_2NaO_2P, M + Na]^+$: 644.9913, Found: 644.9918.

Optical Rotation: $[\alpha]_D^{25}$= 35.0° (*c* = 0.5, acetone).

HPLC analysis: Chiralcel AD-H (Hexane/*i*-PrOH = 90:10, flow rate = 1.0 mL/min, wave length = 254 nm), t_R = 6.313 min (minor), t_R = 18.938 min (major).

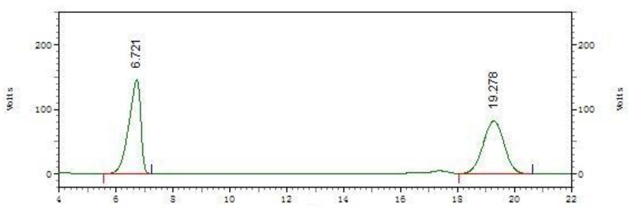

UV-WL1

RetTime (min)	Width (min)	Height (Volts)	Area	Area (%)
6.721	1.71	580451	16795060	50.082
19.278	2.56	325201	16740355	49.918

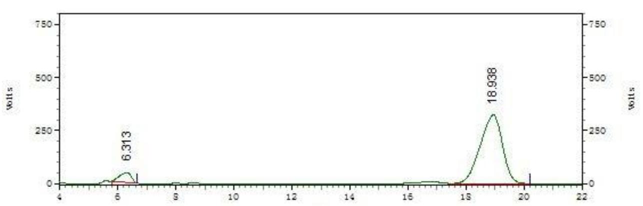

UV-WL1

RetTime (min)	Width (min)	Height (Volts)	Area	Area (%)
6.313	0.87	187109	5196775	6.994
18.938	2.74	1292798	69102734	93.006

(*S*)-(4-bromo-5-(6-bromo-2-hydroxynaphthalen-1-yl)-3-(tert-butyl)-1*H*-pyrazol-1-yl) diphenylphosphine oxide (4z)

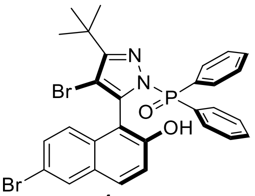

4z

Appearance: white solid.

Yield: 97%, 51.5 mg. **ee** = 87%.

^{1}H NMR (400 MHz, $CDCl_3$) δ 8.74 (s, 1H), 7.92 (dd, *J* = 12.9, 7.8 Hz, 2H), 7.72 (s, 1H), 7.63 – 7.56 (m, 1H), 7.52 – 7.40 (m, 5H), 7.24 (s, 1H), 7.13 (t, *J* = 7.3 Hz, 1H), 7.06 (s, 1H), 6.99 – 6.89 (m, 2H), 6.81 (d, *J* = 8.9 Hz, 1H), 1.43 (s, 9H).

^{13}C NMR (100 MHz, $CDCl_3$) δ 154.57, 143.16, 133.03, 132.96, 132.93, 132.23, 132.20, 131.59, 131.48, 130.79, 130.60, 129.77, 129.70, 129.47, 128.11, 127.97, 127.83, 125.91, 100.39, 33.84, 28.51.

HRMS (ESI) m/z Calcd for $[C_{29}H_{25}Br_2N_2NaO_2P, M + Na]^+$: 644.9913, Found: 644.9917.

Optical Rotation: $[\alpha]_D^{25}$= 31.6° (*c* = 0.5, acetone).

HPLC analysis: Chiralcel AD-H (Hexane/*i*-PrOH = 85:15, flow rate = 1.0 mL/min, wave length = 254 nm), t_R = 6.879 min (major), t_R = 7.638 min (minor).

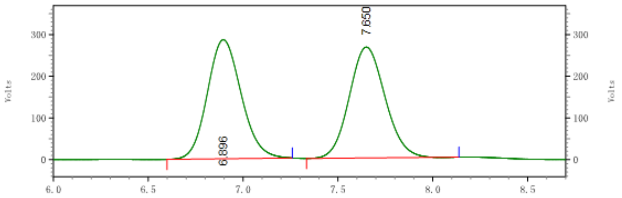

UV-WL1

RetTime (min)	Width (min)	Height (Volts)	Area	Area (%)
6.896	0.66	1142093	14194295	50.404
7.650	0.80	1064000	13966888	49.596

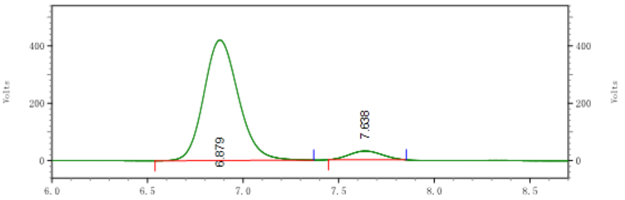

UV-WL1

RetTime (min)	Width (min)	Height (Volts)	Area	Area (%)
6.879	0.83	1680587	21118621	93.575
7.638	0.41	121373	1450041	6.425

(*S*)-(4-bromo-5-(6-bromo-2-hydroxynaphthalen-1-yl)-3-(pentan-3-yl)-1*H*-pyrazol-1-yl) diphenylphosphine oxide (4aa)

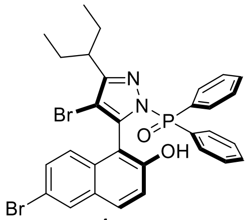

4aa

Appearance: white solid.

Yield: 72%, 52.2 mg. **ee** = 92%.

^{1}H NMR (400 MHz, $CDCl_3$) δ 8.88 (s, 1H), 7.90 (s, 1H), 7.75 – 7.64 (m, 3H), 7.47 (d, *J* = 29.0 Hz, 4H), 7.37 (dd, *J* = 13.8, 8.3 Hz, 3H), 7.23 (d, *J* = 7.3 Hz, 2H), 7.16 (d, *J* = 6.9 Hz, 3H), 2.89 – 2.80 (m, 1H), 1.87 (dq, *J* = 14.3, 7.1, 6.7 Hz, 4H), 0.93 (dt, *J* = 15.4, 7.3 Hz, 6H).

^{13}C NMR (100 MHz, acetone-d_6) δ 206.23, 149.37, 149.29, 133.45, 132.98, 132.76, 132.65, 132.57, 132.50, 132.47, 131.15, 130.93, 130.82, 129.72, 129.58, 129.44, 128.47, 128.36, 121.84, 121.81, 119.67, 41.60, 29.85, 28.14, 12.66.

HRMS (ESI) m/z Calcd for $[C_{30}H_{27}Br_2N_2NaO_2P, M + Na]^+$: 659.0069, Found: 659.0063.

Optical Rotation: $[\alpha]_D^{25}$= 26.5° (*c* = 0.3, acetone).

HPLC analysis: Chiralcel AD-H (Hexane/*i*-PrOH = 90:10, flow rate = 1.0 mL/min, wave length = 254 nm), t_R = 9.869min (minor), t_R = 13.825 min (major).

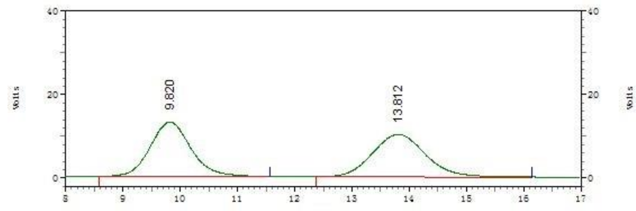

UV-WL1

RetTime (min)	Width (min)	Height (Volts)	Area	Area (%)
9.820	2.98	52133	2588116	49.612
13.812	3.76	40775	2628616	50.388

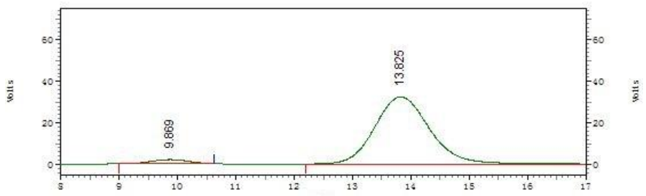

UV-WL1

RetTime (min)	Width (min)	Height (Volts)	Area	Area (%)
9.869	1.63	7868	340120	3.872
13.825	4.85	129409	8444057	96.128

V. Racemization studies

Racemization studies of 2k

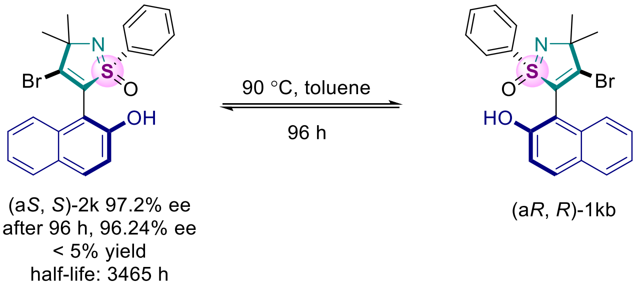

Thermal Racemization of **2k**: A solution of **2k** (17.5 mg, 97% ee) in toluene (3 mL) was heated at 90 °C. At intervals, small samples were taken and the solvent was removed by evaporation. The enantiomeric excess was determined by using HPLC (HPLC conditions: Chiralcel OD-H, Hexane/i-PrOH = 85:15, flow rate = 1.0 mL/min, wave length = 254 nm).

Supplementary Table 4. The change of the ee value of 2k at 90 °C.

Time/h	% second eluted enantiomer (%t)	ln ((%t-50)/(%0-50))
0	97.20	0
0.166	97.18	-0.00042
0.25	97.16	-0.00085
0.42	97.14	-0.00127
0.75	97.14	-0.00127
1	97.12	-0.0017
1.916	97.11	-0.00191
3.916	97.10	-0.00212
5.916	97.05	-0.00318
10.416	96.98	-0.00467
16.416	96.95	-0.00531
21.42	96.91	-0.00616
25.42	96.85	-0.00744
29.42	96.81	-0.0083
33.42	96.80	-0.00851
37.42	96.74	-0.00979
41.42	96.69	-0.01086
45.42	96.67	-0.01138
49.42	96.64	-0.01194
54.42	96.58	-0.01322
58.42	96.55	-0.01387
62.42	96.52	-0.01451
69	96.48	-0.01537
73	96.46	-0.0158
77	96.42	-0.01666
81	96.34	-0.01839
85	96.30	-0.01925
93	96.27	-0.0199
97	96.24	-0.02055

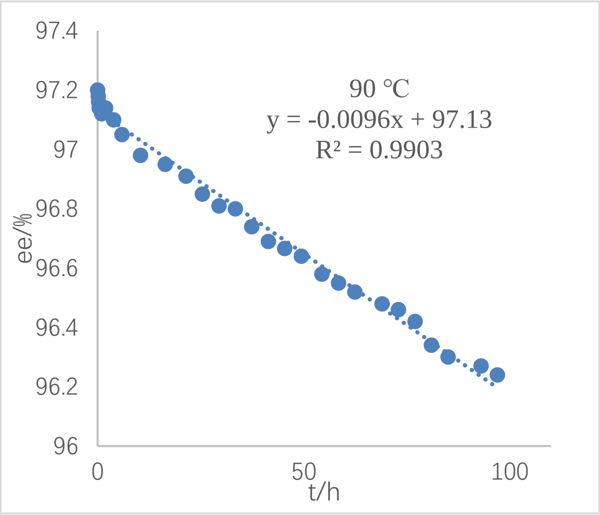

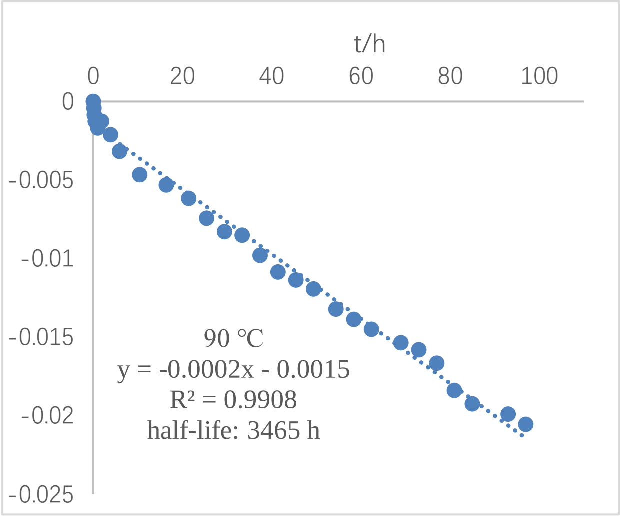

Supplementary Figure 3. Plot of the racemization of compound **2k** at 90 °C.

Racemization studies of 4f

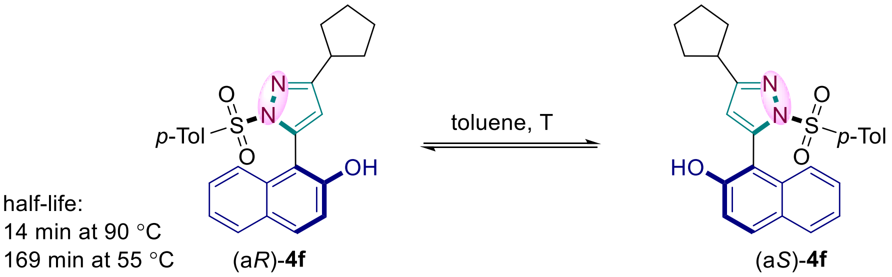

Thermal Racemization of **4f**: A solution of **4f** (20 mg, 95% ee) in toluene (3 mL) was heated at given temperature. At intervals, small samples were taken and the solvent was removed by evaporation. The enantiomeric excess was determined by using HPLC (HPLC conditions: Chiralcel OD-H, Hexane/i-PrOH = 90:10, flow rate = 1.0 mL/min, wave length = 254 nm).

Supplementary Table 5. The change of the ee value of 4f at 90 °C.

Time/min	% second eluted enantiomer (%t)	ln ((%t-50)/(%0-50))
0	95	0
2	92.52	-0.05802
7	86.02	-0.22392
12	79.5	-0.4236
17	73.74	-0.64083
22	63.95	-1.17252
27	58.18	-1.70630
32	51.68	-3.28920

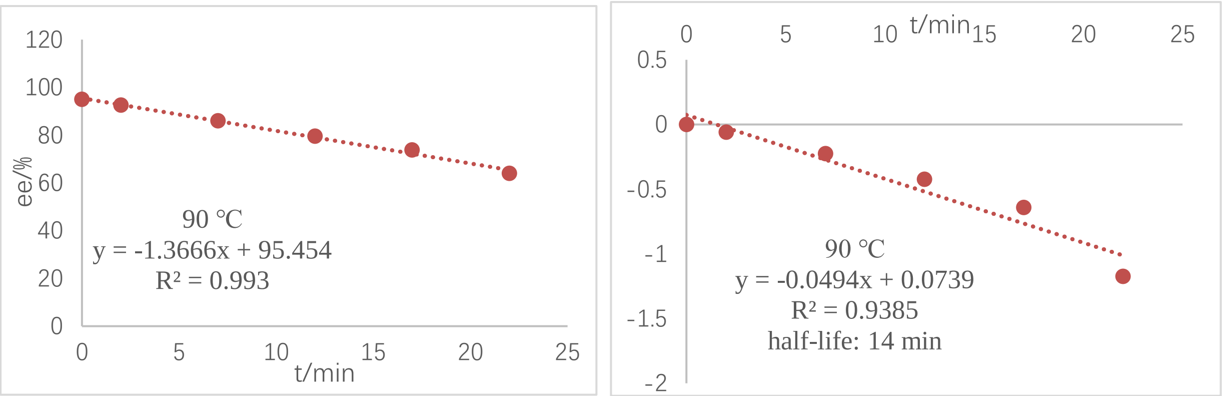

Supplementary Figure 4. Plot of the racemization of compound **4f** at 90 °C.

Supplementary Table 6. The change of the ee value of 4f at 55 °C.

Time/min	% second eluted enantiomer (%t)	ln ((%t-50)/(%0-50))
0	95	0
10	94.1	-0.0202
20	92.35	-0.06069
30	91.1	-0.09065
40	90.4	-0.10783
50	88.55	-0.15471
60	87.85	-0.17303
70	86.34	-0.21374
80	84.62	-0.26223
90	83.98	-0.28089
100	82.82	-0.31562
110	81.48	-0.35731
120	80.3	-0.39551
130	78.9	-0.44282
140	77.56	-0.49030
150	76.48	-0.53027
160	74.84	-0.59421
170	73.94	-0.63111
185	72.56	-0.69048
200	71.1	-0.75739
220	69.08	-0.85802
240	67.38	-0.95134
270	64.76	-1.11474

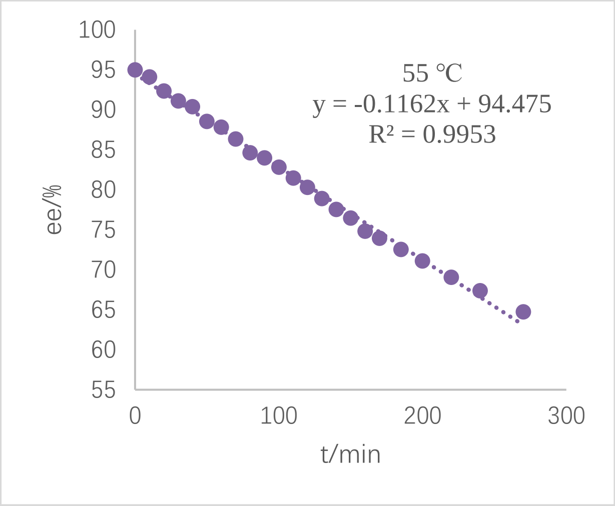

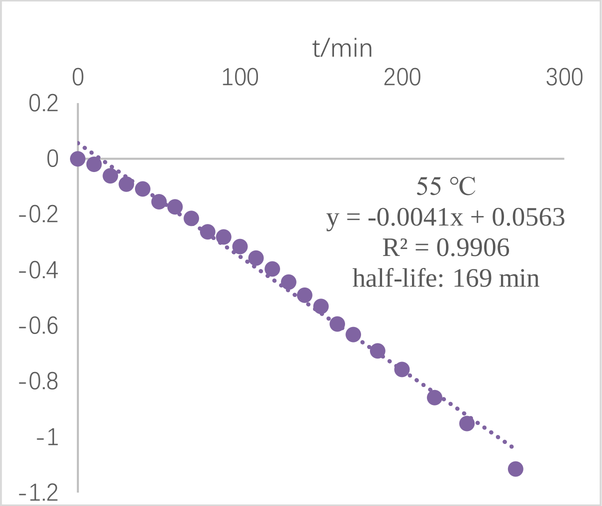

Supplementary Figure 5. Plot of the racemization of compound **4f** at 55 ºC.

Racemization studies of *N*-diphenylphosphine oxide pyrazoles (**4q-4aa**): The *N*-diphenylphosphine oxide pyrazole compounds were unstable and easily decomposes under heating conditions.

VI. Control experiments and plausible catalytic cycle

Compound (±)-**1l’** was prepared according to the general procedure as described for **S3**. The control experiment was carried out following the standard conditions: 0.05 mmol (±)-**1l’** and 10 mol% catalyst **F** in DCM (2.0 mL) at -78 ºC for 0.5 h.

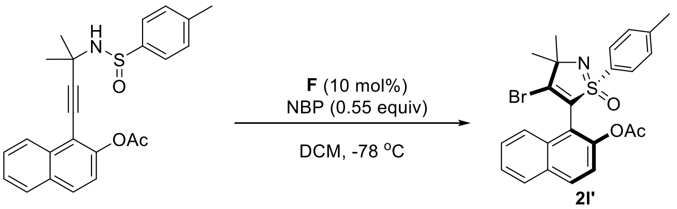

(±)-**1l'** **2l'** No reaction

Analytical dates of 1l’

1-(3-methyl-3-((p-tolylsulfinyl)amino)but-1-yn-1-yl)naphthalen-2-yl acetate (1l’)

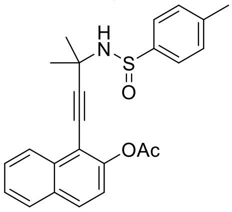

1l'

Appearance: yellow oil.

^{1}H NMR (400 MHz, $CDCl_3$) δ 8.33 (d, *J* = 8.3 Hz, 1H), 7.82 (d, *J* = 8.7 Hz, 2H), 7.63 (d, *J* = 8.2 Hz, 2H), 7.58 (d, *J* = 7.2 Hz, 1H), 7.49 (t, *J* = 7.5 Hz, 1H), 7.27 (d, *J* = 8.7 Hz, 2H), 7.23 (d, *J* = 8.9 Hz, 1H), 4.32 (s, 1H), 2.41 (s, 3H), 2.38 (s, 3H), 1.88 (s, 3H), 1.80 (s, 3H).

^{13}C NMR (100 MHz, $CDCl_3$) δ 168.93, 150.39, 142.95, 141.21, 133.84, 131.12, 129.77, 129.46, 128.06, 127.42, 126.12, 126.04, 125.36, 120.95, 112.56, 102.19, 77.08, 52.02, 32.05, 31.77, 21.25, 20.93.

HRMS (ESI) m/z Calcd for $[C_{24}H_{23}NNaO_3S, M + Na]^+$: 428.1291, Found: 428.1295.

Compound (±)-**1l”** was prepared according to the general procedure as following. (Using the synthesis method of **S3** to get **S3’**). To a solution of the **S3’** (1.0 equiv) in methanol (0.5 M) was added $NaBH_4$ (1.1 equiv) at 0 ºC and move to room temperature, After the reaction was completed by TLC monitored. The reaction was quenched by pouring into cooled saturated NH_4Cl aqueous and the mixture was extracted with EA. The organic phases were combined, washed with brine, dried over Na_2SO_4, and concentrated to afford the crude product. The crude product was purified by column chromatography on silica gel (PE/EA = 5:1) to afford (±)-**1l”**.

The control experiment was carried out following the standard conditions: 0.05 mmol (±)-**1l”** and 10 mol% catalyst **F** in DCM (2.0 mL) at -78 ºC for 0.5 h.

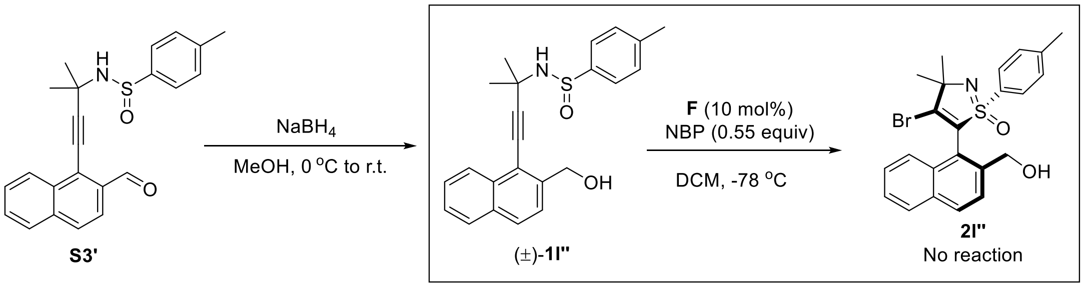

Analytical dates of 1l’’

***N*-(4-(2-(hydroxymethyl)naphthalen-1-yl)-2-methylbut-3-yn-2-yl)-4-methylbenzenesulfinamide (1l’’)**

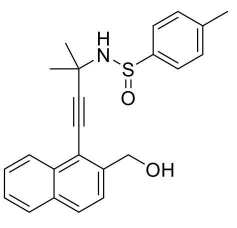

1l''

Appearance: white solid.

^{1}H NMR (400 MHz, $CDCl_3$) δ 8.29 (d, *J* = 8.3 Hz, 1H), 7.84 – 7.74 (m, 2H), 7.59 (d, *J* = 7.5 Hz, 2H), 7.54 (t, *J* = 7.5 Hz, 2H), 7.50 – 7.44 (m, 1H), 7.25 (d, *J* = 5.7 Hz, 2H), 4.96 (q, *J* = 12.4 Hz, 2H), 4.64 (s, 1H), 4.47 (s, 1H), 2.37 (s, 3H), 1.88 (s, 3H), 1.78 (s, 3H).

^{13}C NMR (100 MHz, $CDCl_3$) δ 142.41, 142.05, 141.41, 133.44, 132.45, 129.52, 128.61, 128.07, 126.72, 126.59, 126.02, 125.92, 125.45, 125.06, 118.57, 102.22, 79.92, 64.01, 51.02, 32.31, 30.46, 21.28.

HRMS (ESI) m/z Calcd for [$C_{23}H_{23}NNaO_2S$, M + Na]$^+$: 400.1342, Found: 400.1349.

Compound (±)-**3i’** was prepared according to the general procedure as described for **S7**. The control experiment was carried out following the standard conditions: 0.05 mmol (±)-**3a’** and 10 mol% catalyst **C** in DCM (2.0 mL) at 25 ºC for 12 h.

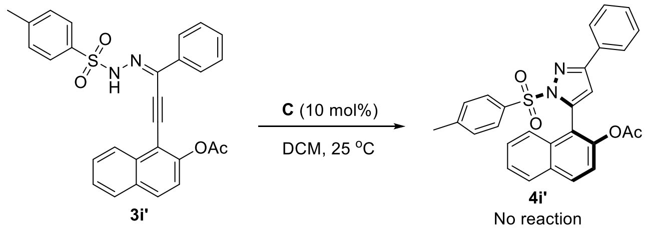

Analytical dates of 3i’

(*Z*)-1-(3-phenyl-3-(2-tosylhydrazineylidene)prop-1-yn-1-yl)naphthalen-2-yl acetate(3i’)

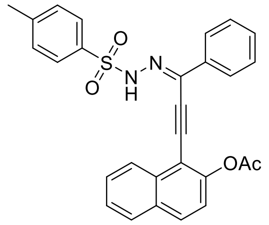

3i'

Appearance: white solid.

^{1}H NMR (400 MHz, $CDCl_3$) δ 8.83 (s, 1H), 8.22 (d, *J* = 8.3 Hz, 1H), 8.01 – 7.95 (m, 3H), 7.92 (t, *J* = 7.6 Hz, 3H), 7.67 (d, *J* = 7.1 Hz, 1H), 7.57 (t, *J* = 7.2 Hz, 1H), 7.47 – 7.40 (m, 3H), 7.35 (d, *J* = 9.0 Hz, 1H), 7.32 (d, *J* = 8.2 Hz, 2H), 2.49 (s, 3H), 2.41 (s, 3H).

^{13}C NMR (100 MHz, $CDCl_3$) δ 169.12, 151.59, 144.37, 135.62, 135.44, 133.87, 133.25, 132.20, 131.08, 130.26, 129.73, 128.57, 128.42, 127.94, 126.72, 126.60, 125.31, 121.19, 110.39, 98.11, 86.71, 21.61, 21.24.

HRMS (ESI) m/z Calcd for [$C_{28}H_{22}N_2NaO_4S$, M + Na]$^+$: 505.1192, Found: 505.1187.

Compound (±)-**3q’** was prepared according to the general procedure as described for **S8**. The control experiment was carried out following the standard conditions: 0.05 mmol (±)- **3q’** and 20 mol% catalyst **E** in toluene (1.0 mL) at 25 ºC for 1.0 h.

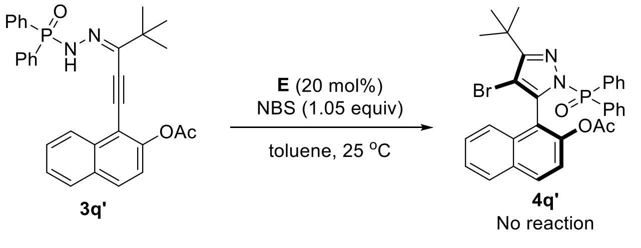

Analytical dates of 3q’

(Z)-1-(3-(2-(diphenylphosphoryl)hydrazineylidene)-4,4-dimethylpent-1-yn-1-yl)naphthalen-2-yl acetate (3q’)

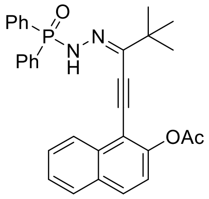

3q'

Appearance: white solid.

^{1}H NMR (400 MHz, $CDCl_3$) δ 8.12 (d, *J* = 7.6 Hz, 1H), 7.92 (d, *J* = 7.3 Hz, 2H), 7.89 (d, *J* = 8.3 Hz, 3H), 7.87 – 7.84 (m, 1H), 7.80 (d, *J* = 20.9 Hz, 1H), 7.58 – 7.52 (m, 2H), 7.47 (td, *J* = 7.5, 3.4 Hz, 5H), 7.28 (s, 1H), 2.28 (s, 3H), 1.81 (s, 1H), 1.25 (s, 9H).

^{13}C NMR (100 MHz, $CDCl_3$) δ 158.34, 158.24, 154.08, 142.65, 132.89, 132.82, 132.21, 132.02, 131.46, 128.61, 128.50, 128.43, 128.13, 127.99, 127.90, 127.80, 127.66, 126.33, 123.94, 123.22, 120.53, 117.80, 111.01, 102.98, 41.19, 26.34, 26.14, 11.89.

HRMS (ESI) m/z Calcd for $[C_{31}H_{29}N_2NaO_3P, M + Na]^+$: 531.1808, Found: 531.1802.

Plausible catalytic cycle

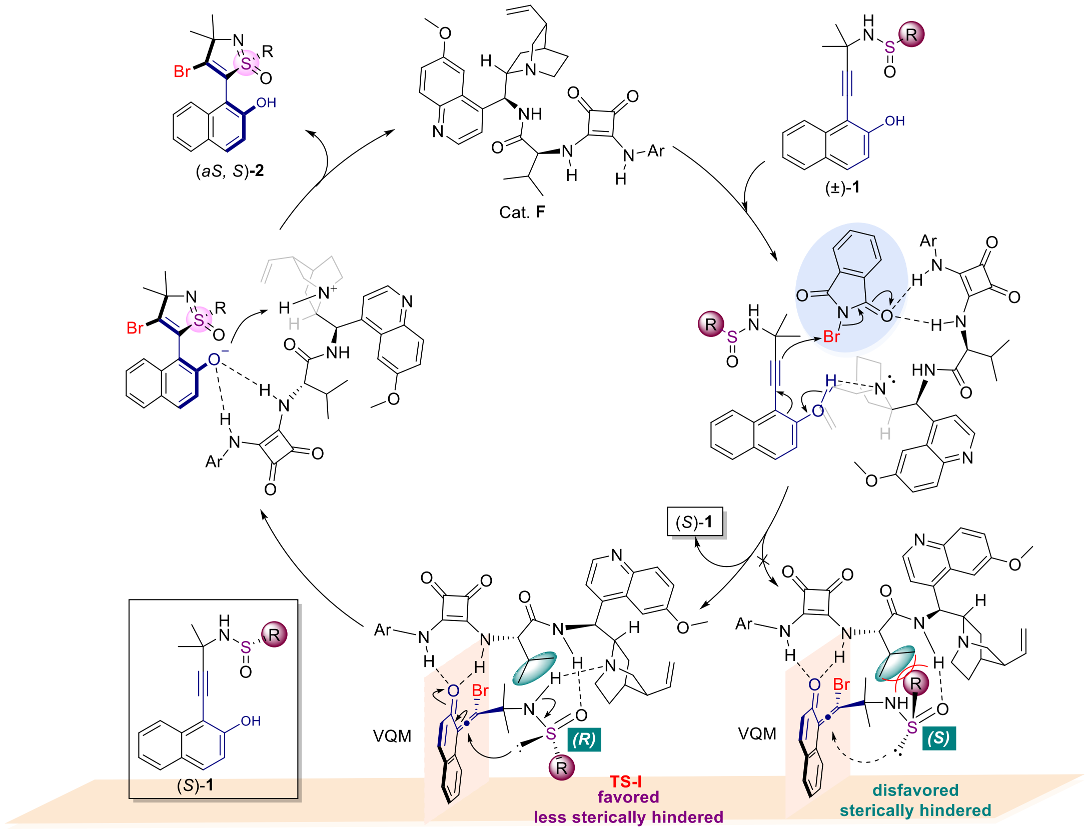

Supplementary Figure 6. Plausible catalytic cycle for (±)-1.

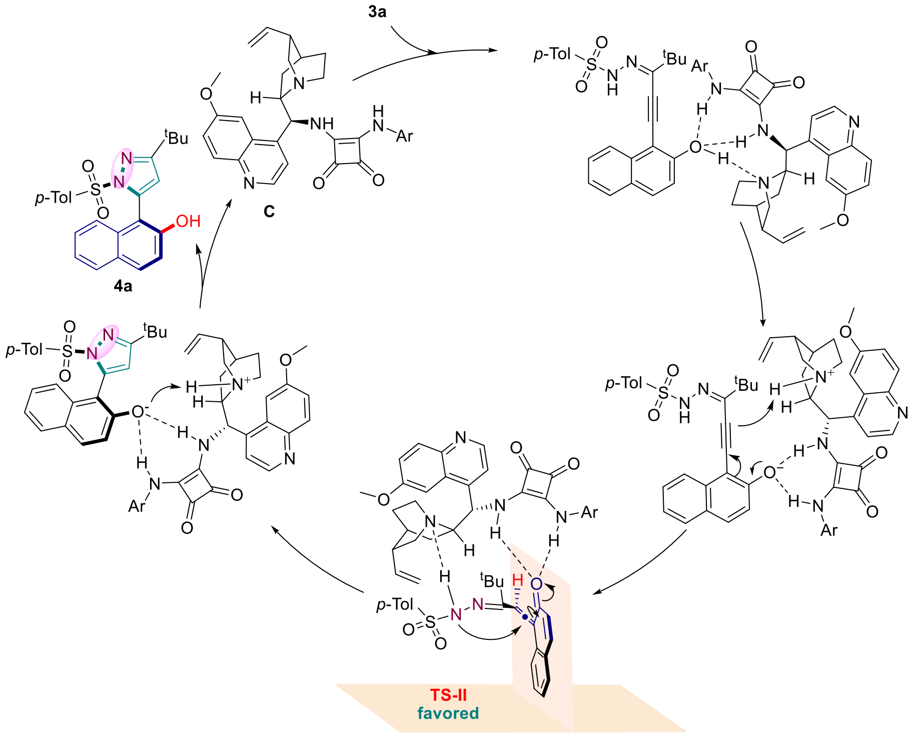

Supplementary Figure 7. Plausible catalytic cycle for 3a.

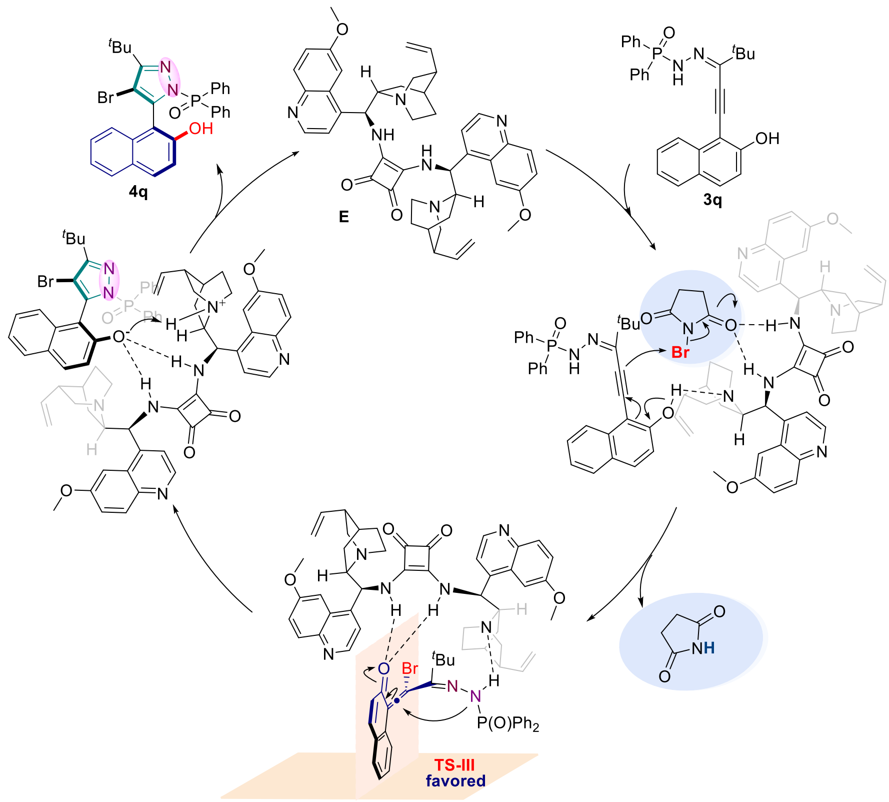

Supplementary Figure 8. Plausible catalytic cycle for 3q.

VII. ^{1}H and ^{13}C NMR spectra

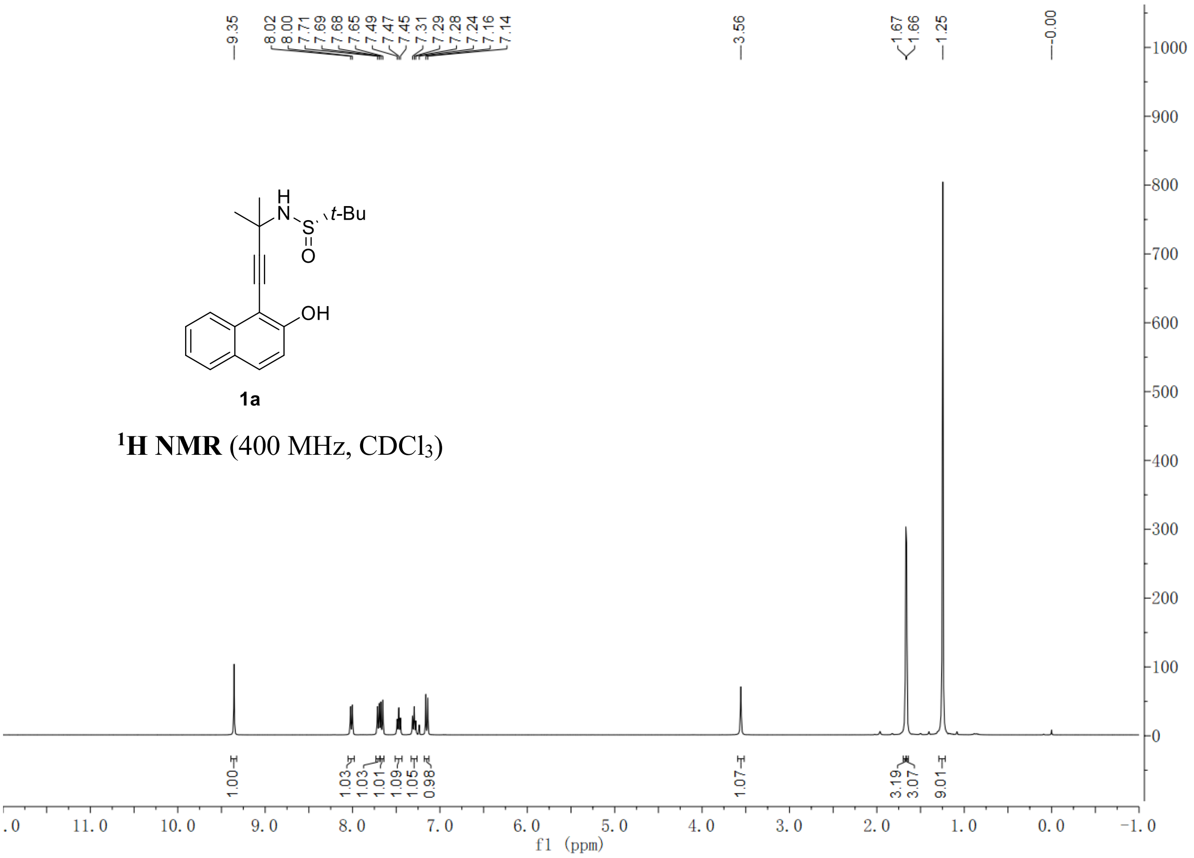

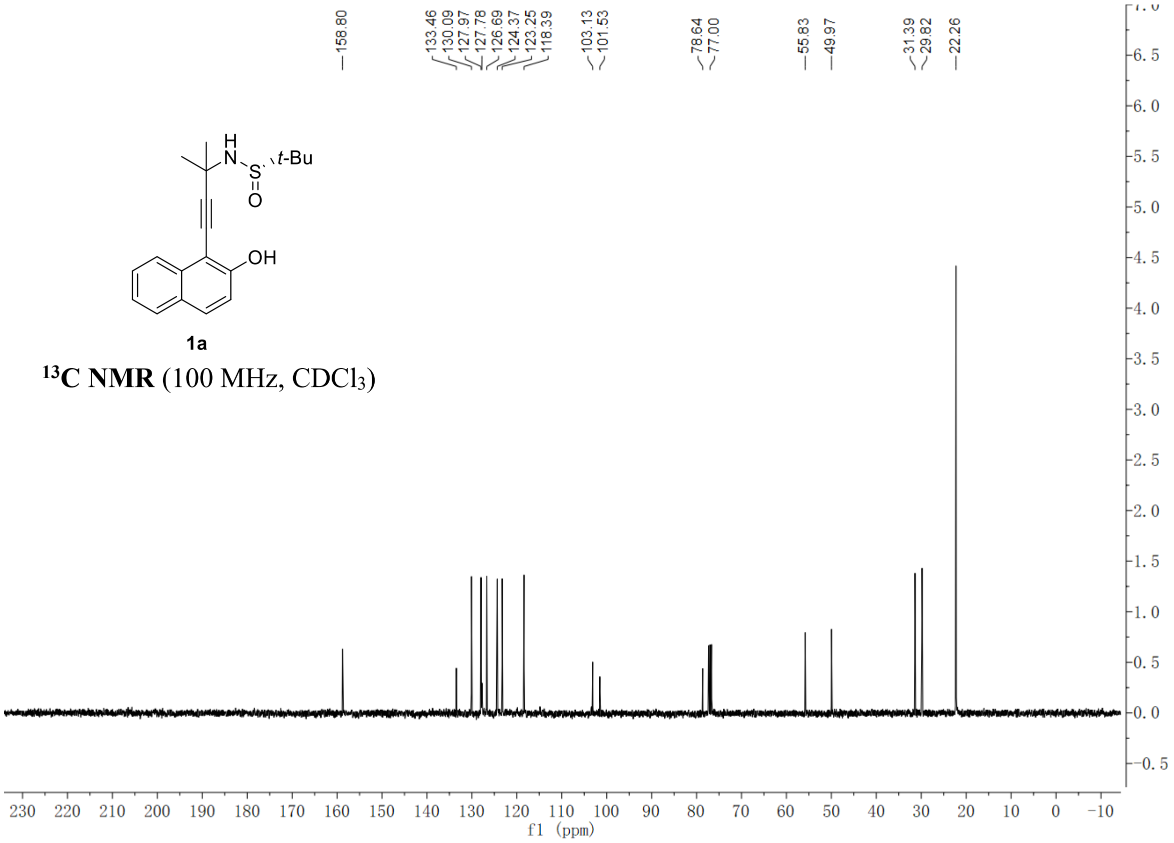

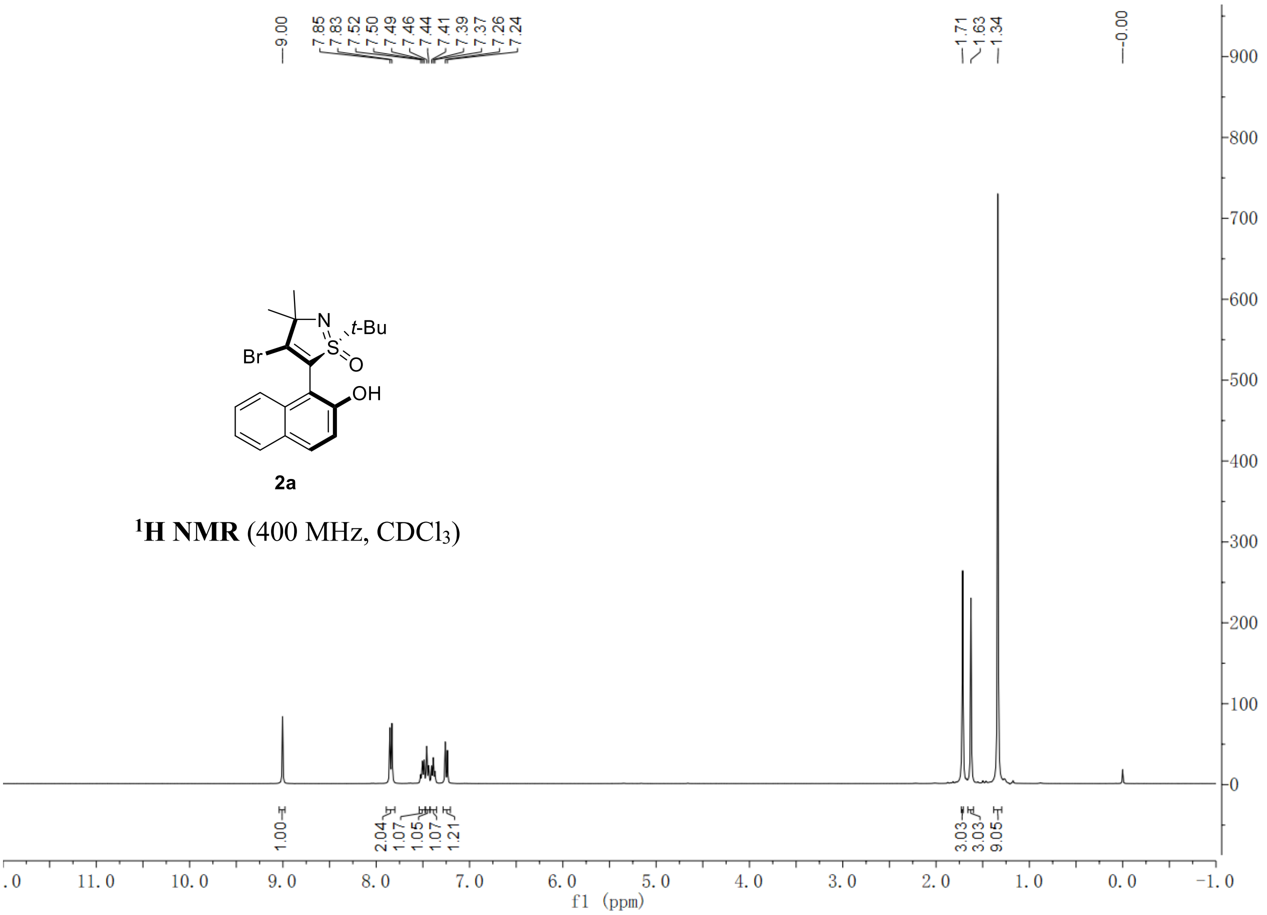

2a

^{1}**H NMR** (400 MHz, $CDCl_3$)

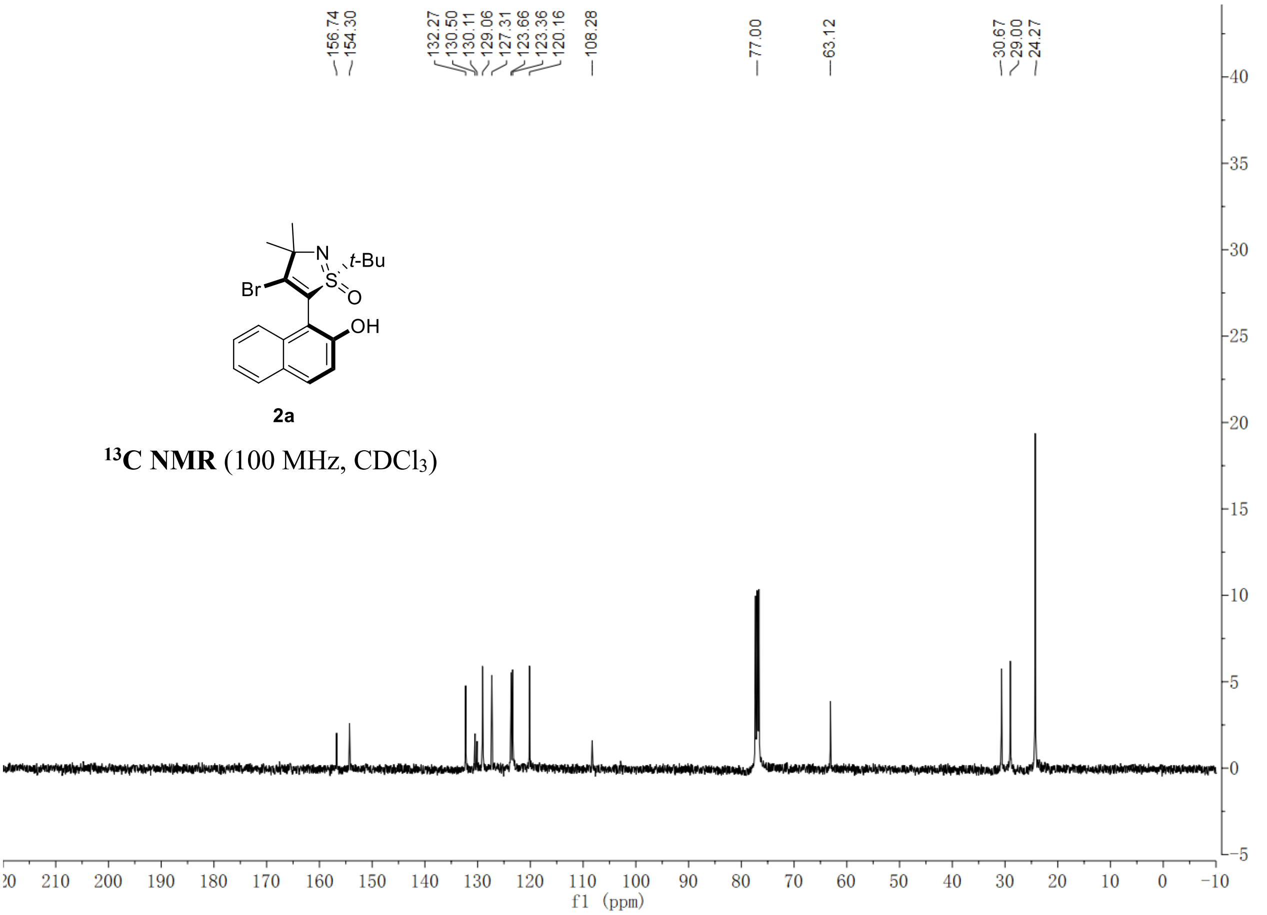

2a

^{13}**C NMR** (100 MHz, $CDCl_3$)

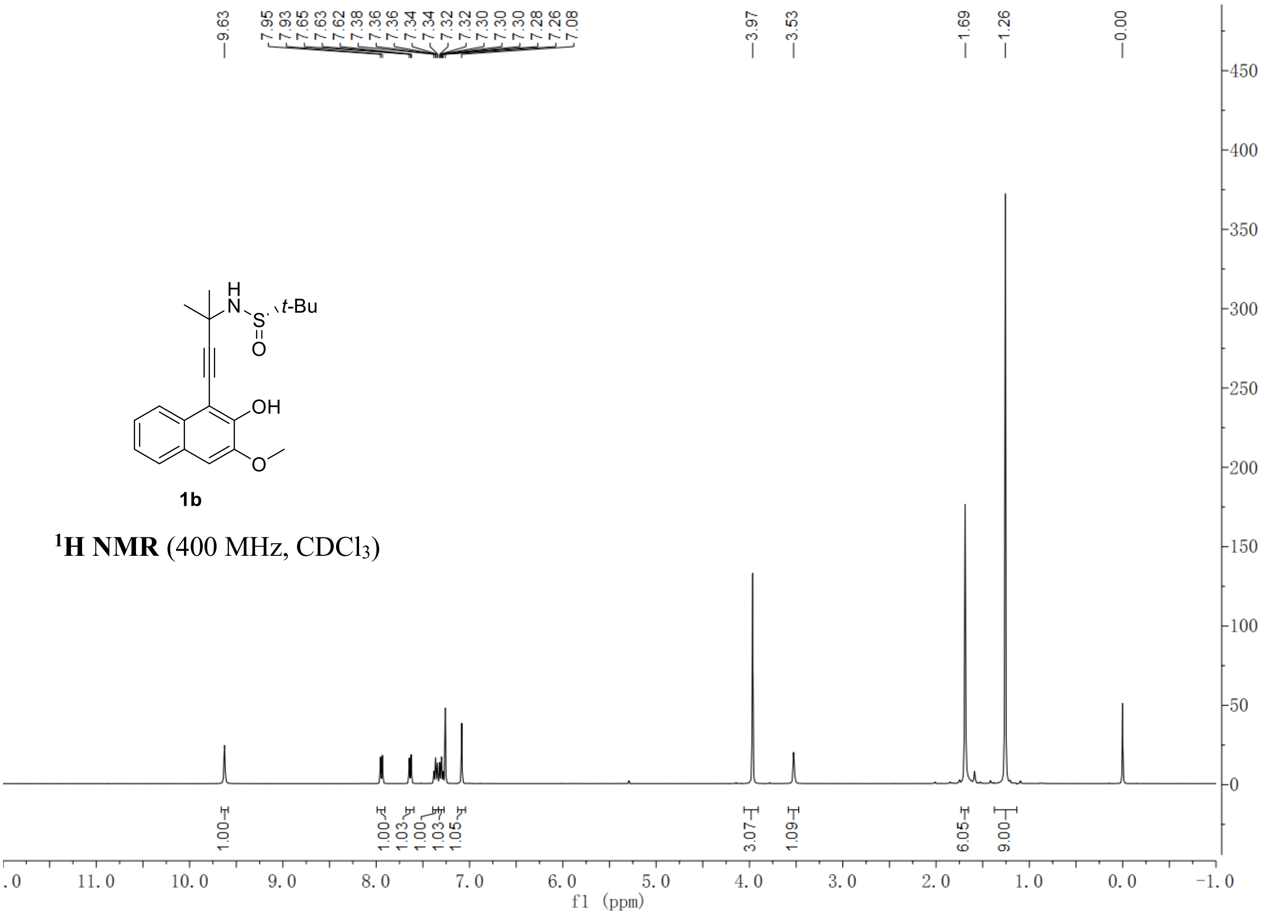
9.63
7.95 7.93 7.65 7.63 7.62 7.38 7.36 7.36 7.34 7.34 7.32 7.32 7.30 7.30 7.30 7.28 7.26 7.08
3.97
3.53
1.69
1.26
0.00
H
N
S
t-Bu
O
OH
O
1b
^{1}H NMR (400 MHz, CDCl$_3$)
1.00
1.00
1.03
1.00
1.03
1.05
3.07
1.09
6.05
9.00
f1 (ppm)

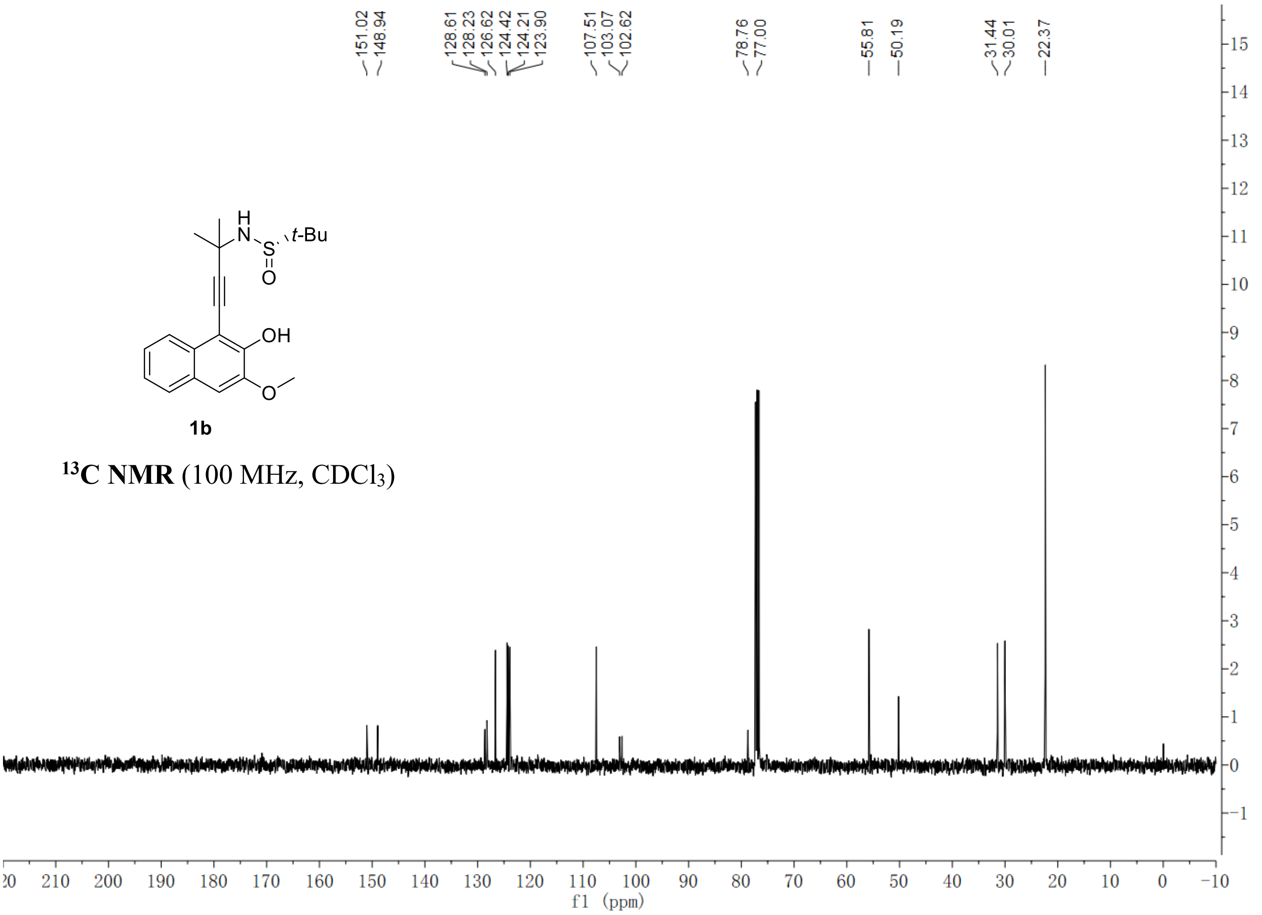
151.02
148.94
128.61 128.23 126.62 124.42 124.21 123.90
107.51 103.07 102.62
78.76 77.00
55.81
50.19
31.44 30.01
22.37
H
N
S
t-Bu
O
OH
O
1b
^{13}C NMR (100 MHz, CDCl$_3$)
f1 (ppm)

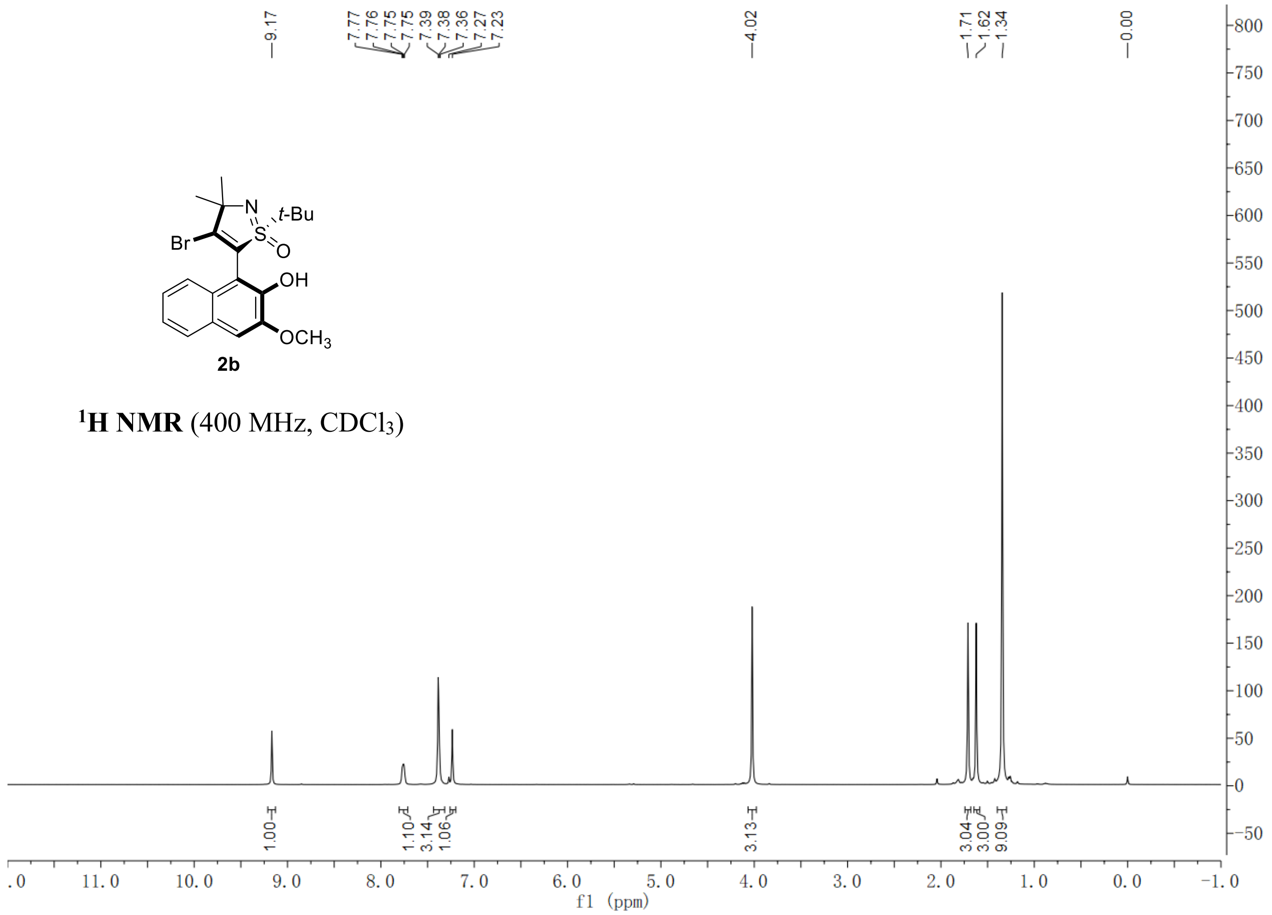

¹H NMR (400 MHz, $CDCl_3$)

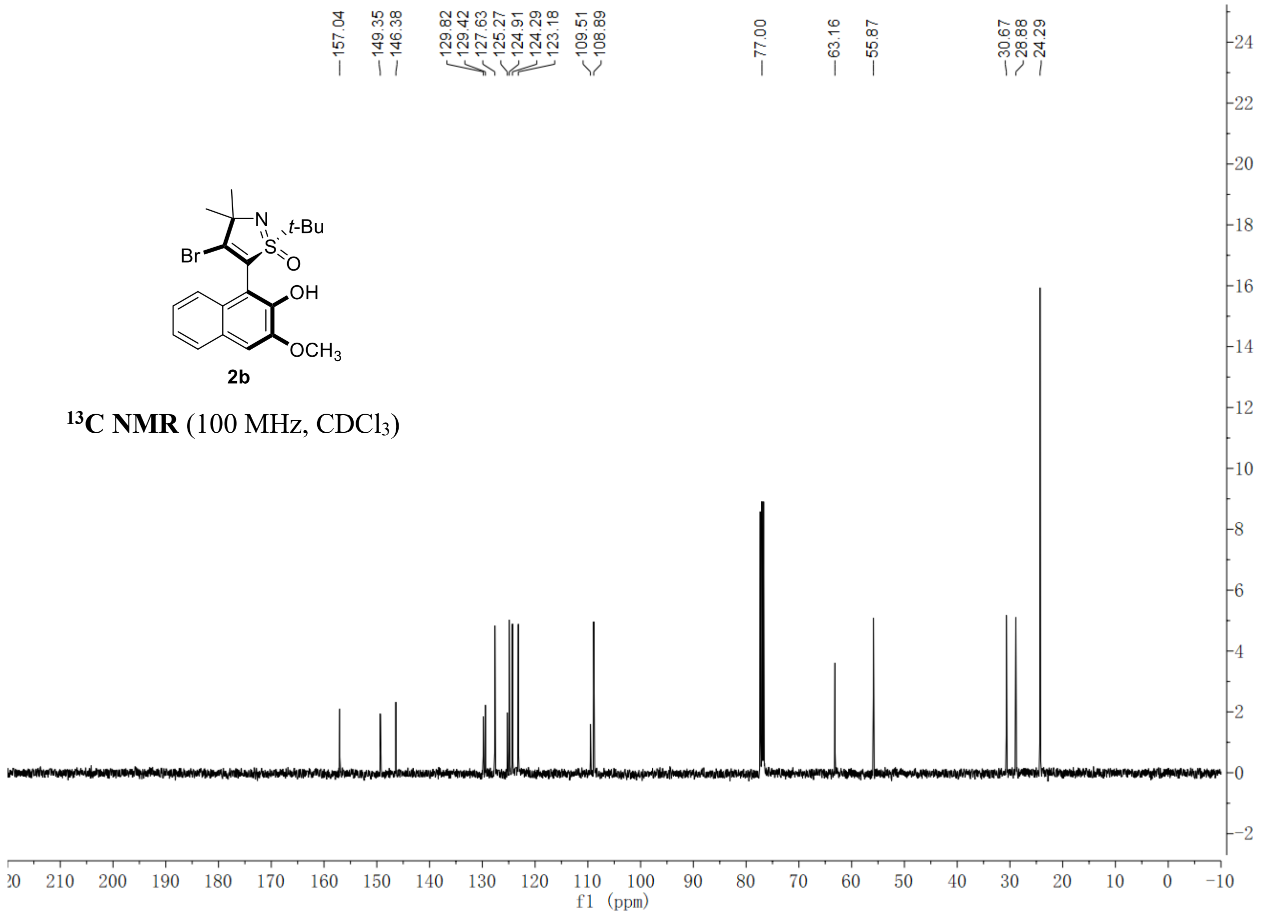

¹³C NMR (100 MHz, $CDCl_3$)

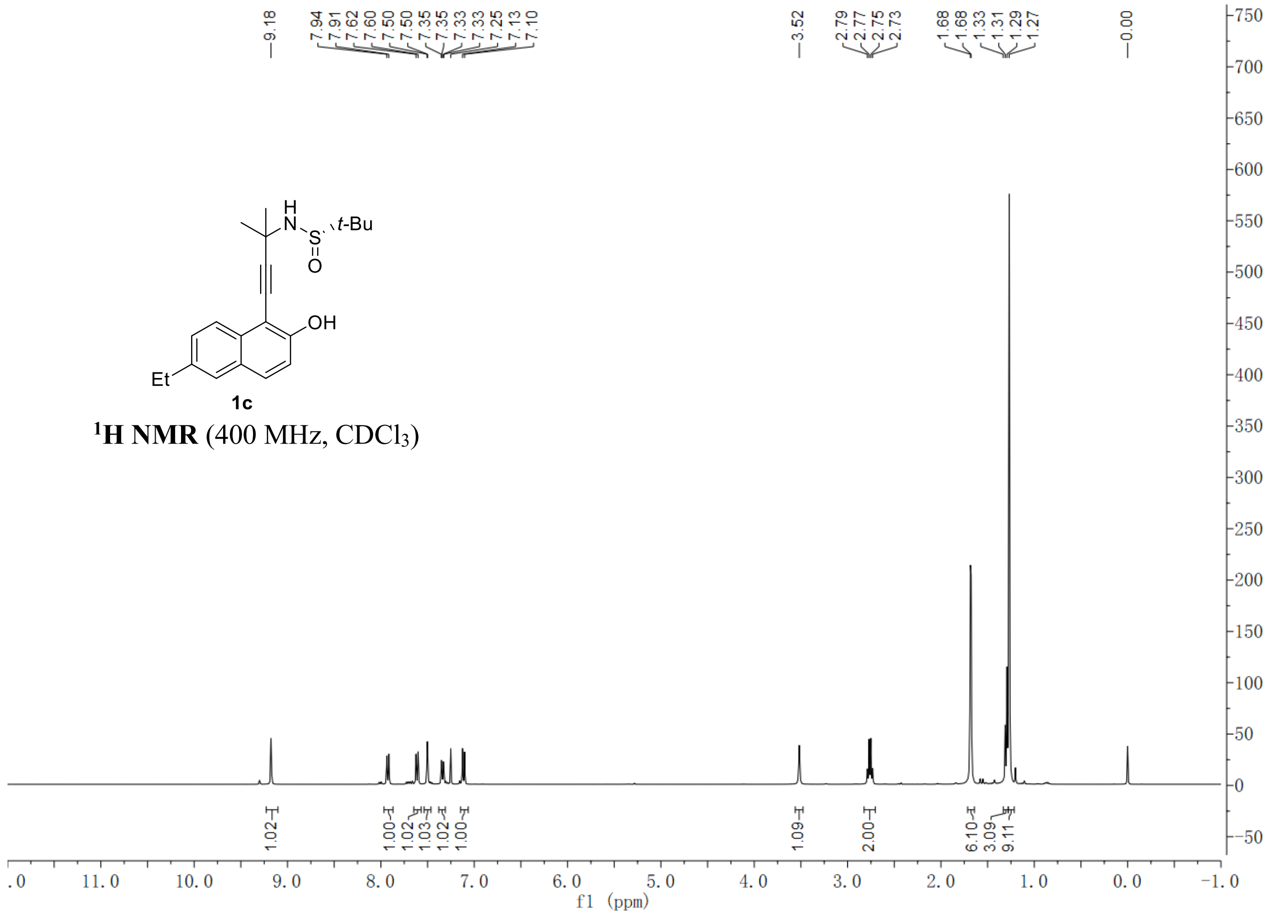
9.18
7.94 7.91 7.62 7.60 7.50 7.50 7.35 7.35 7.33 7.33 7.25 7.13 7.10
3.52
2.79 2.77 2.75 2.73
1.68 1.68 1.33 1.31 1.29 1.27
0.00
H
N
S
t-Bu
O
OH
Et
1c
^{1}H NMR (400 MHz, CDCl$_3$)
1.02 1.00 1.02 1.03 1.02 1.00 1.09 2.00 6.10 3.09 9.11
f1 (ppm)

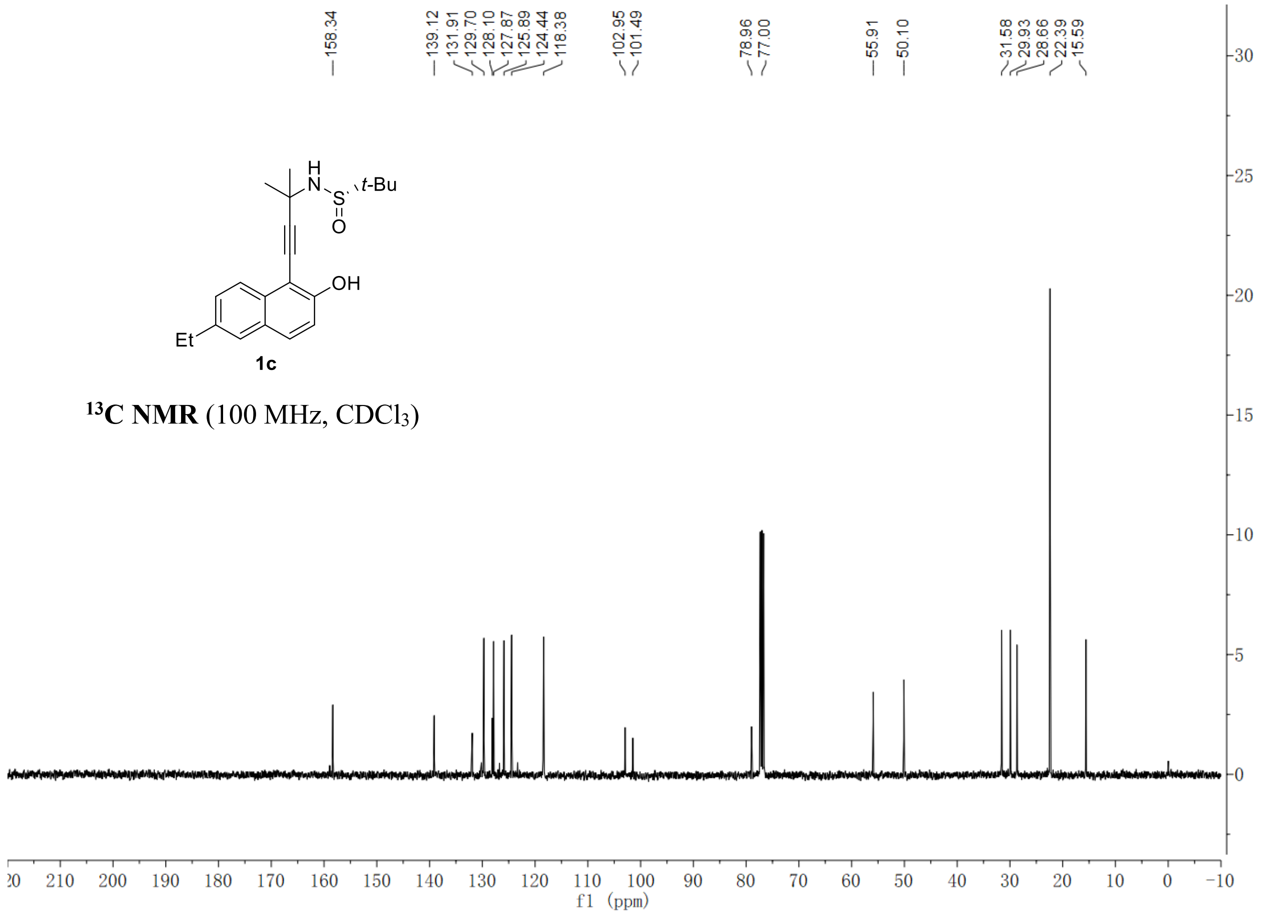
158.34
139.12 131.91 129.70 128.10 127.87 125.89 124.44 118.38
102.95 101.49
78.96 77.00
55.91
50.10
31.58 29.93 28.66 22.39 15.59
H
N
S
t-Bu
O
OH
Et
1c
^{13}C NMR (100 MHz, CDCl$_3$)
f1 (ppm)

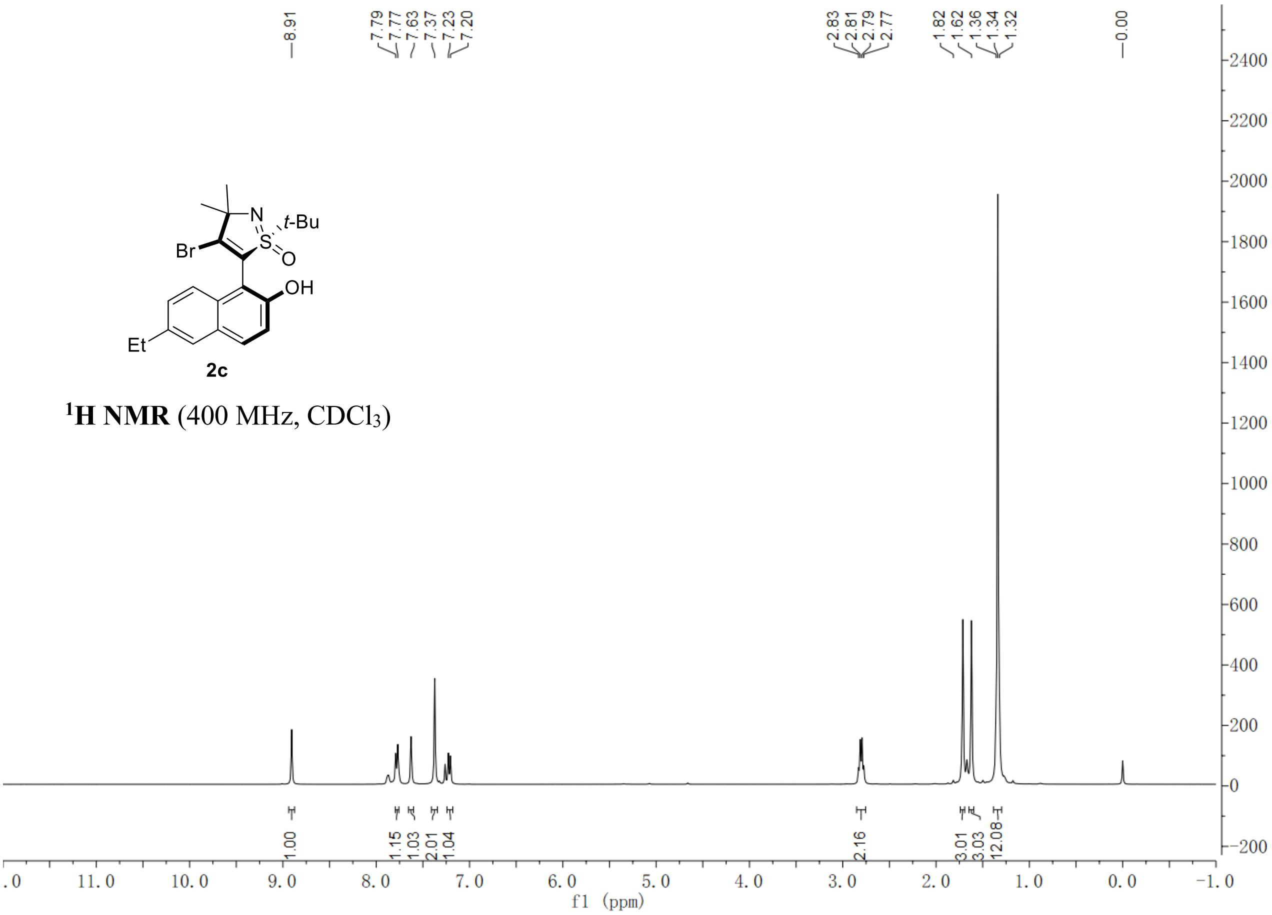

2c

^{1}H NMR (400 MHz, $CDCl_3$)

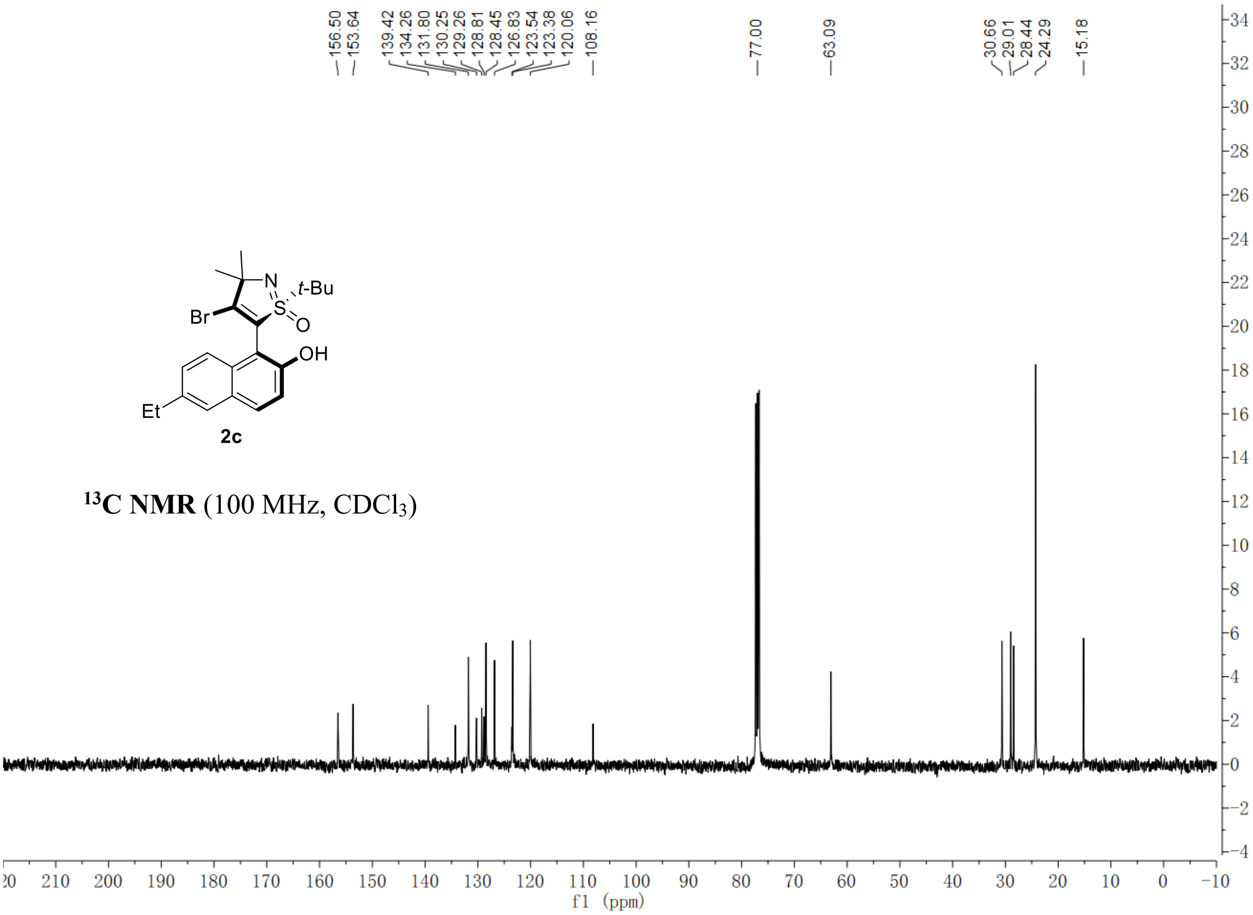

2c

^{13}C NMR (100 MHz, $CDCl_3$)

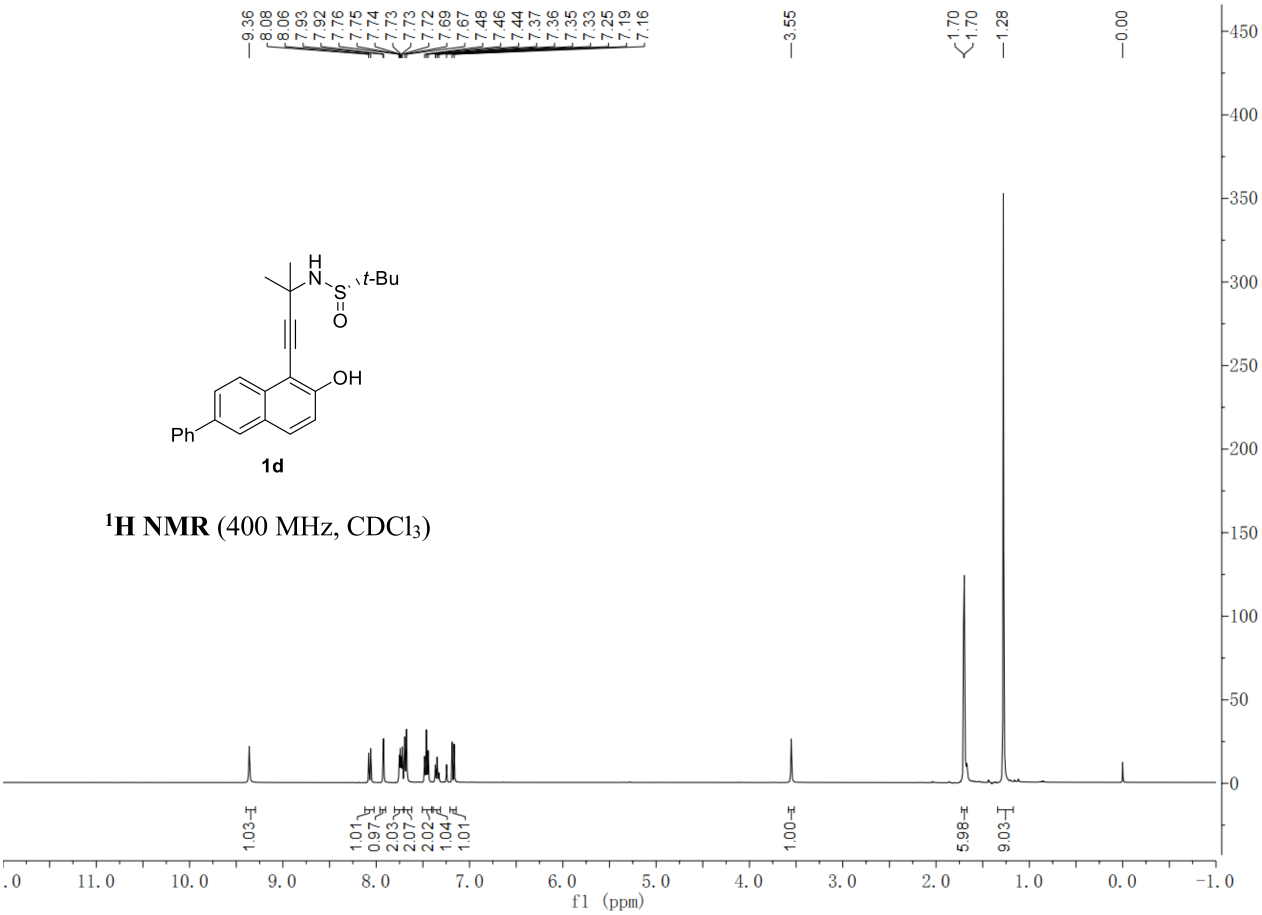

1d
1H NMR (400 MHz, CDCl3)
f1 (ppm)

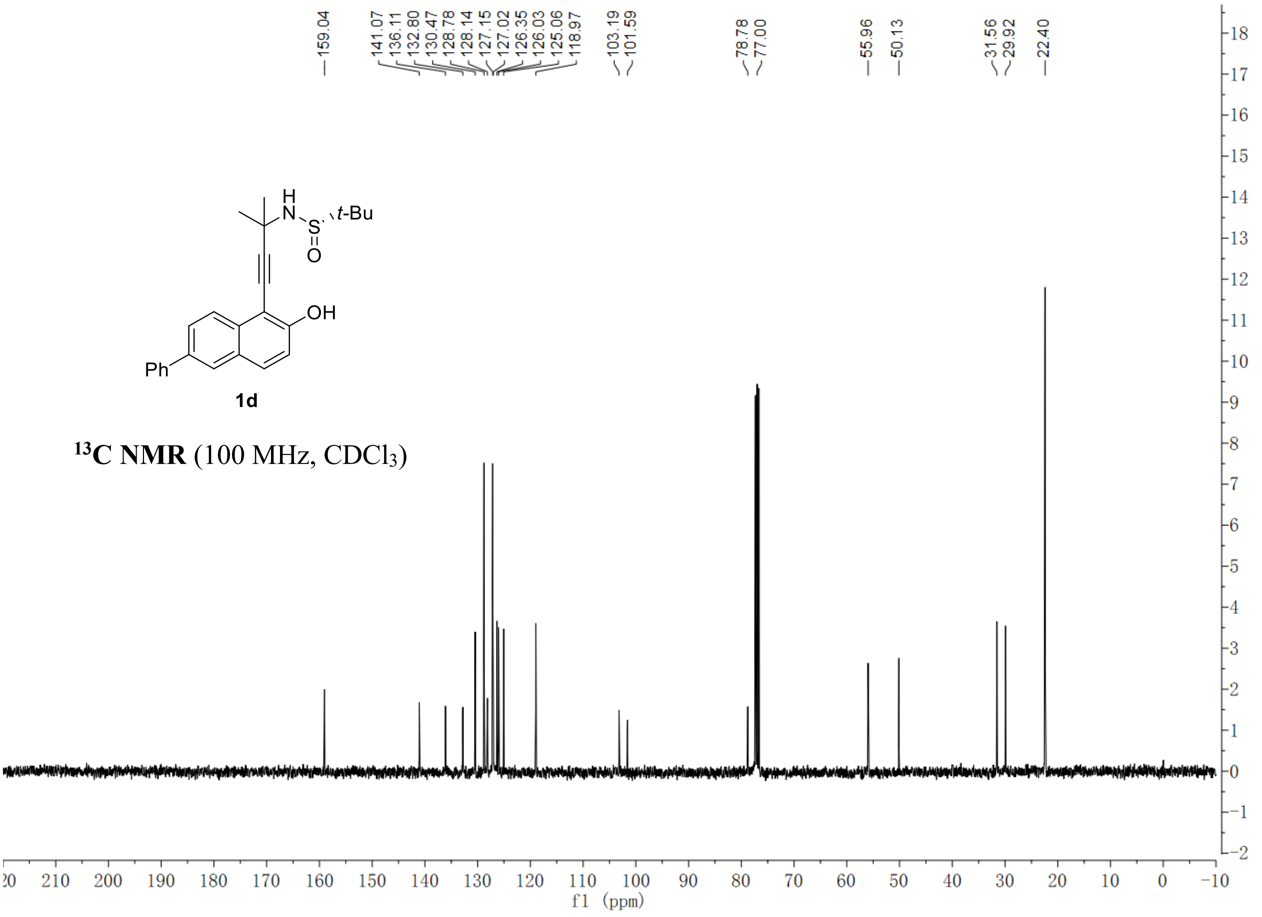

1d
13C NMR (100 MHz, CDCl3)
f1 (ppm)

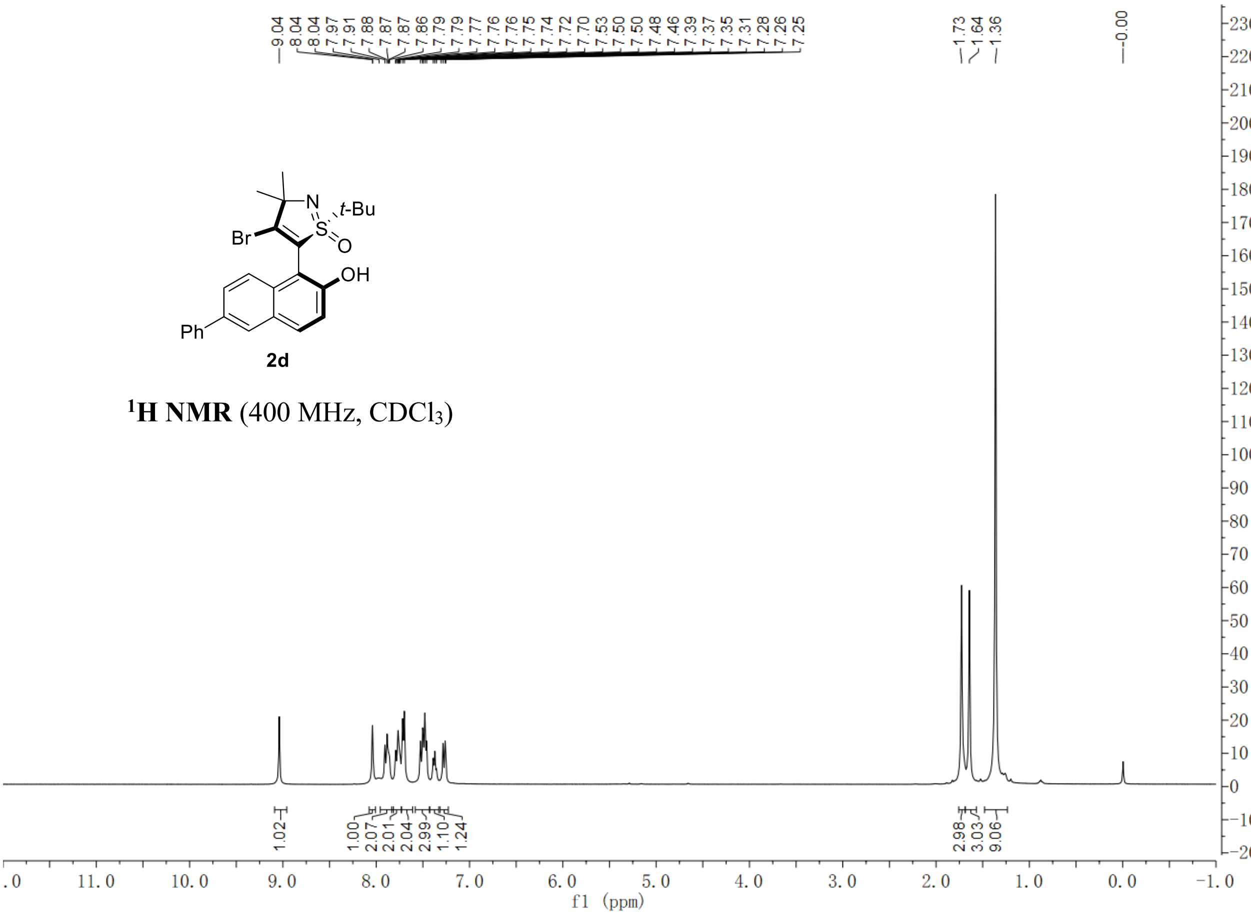

2d

^{1}H NMR (400 MHz, $CDCl_3$)

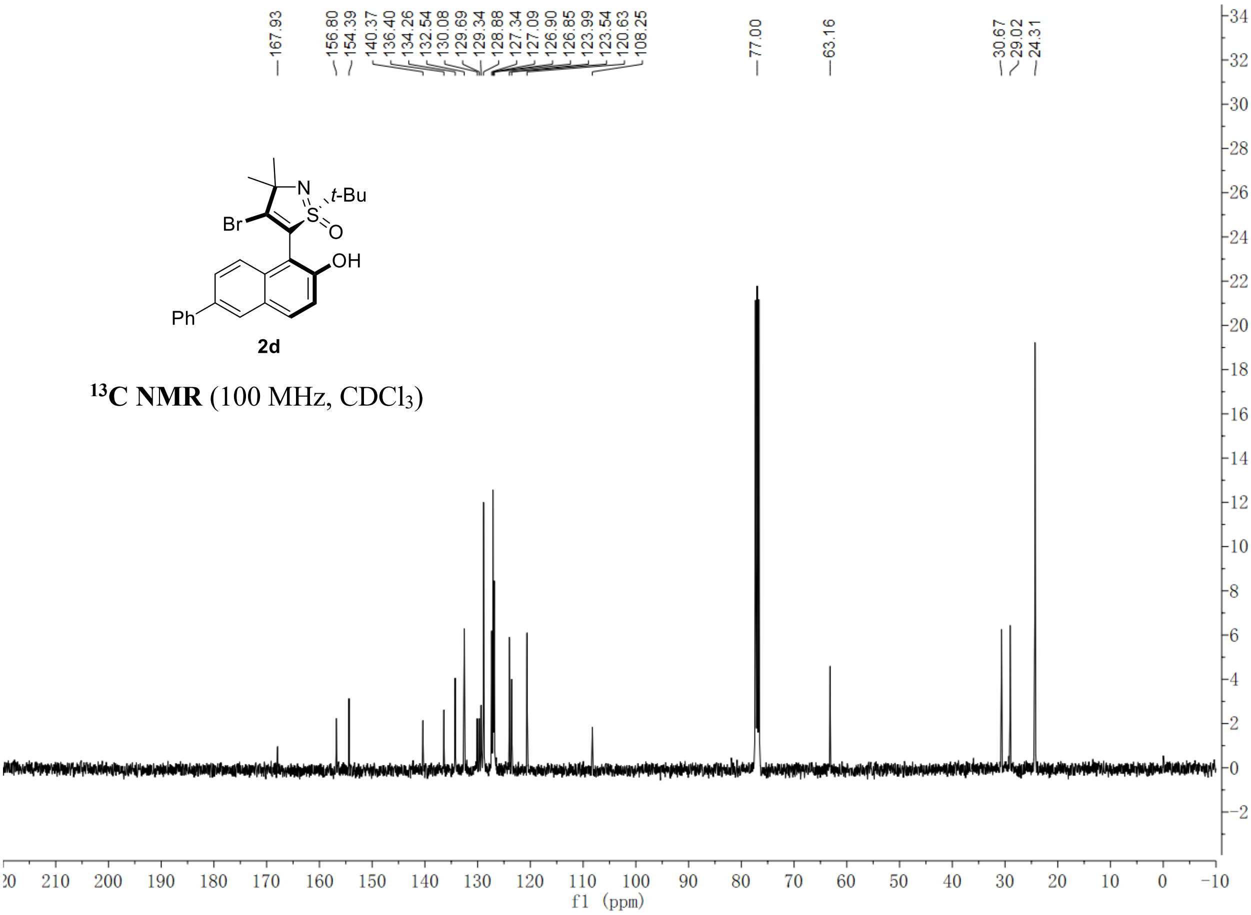

2d

^{13}C NMR (100 MHz, $CDCl_3$)

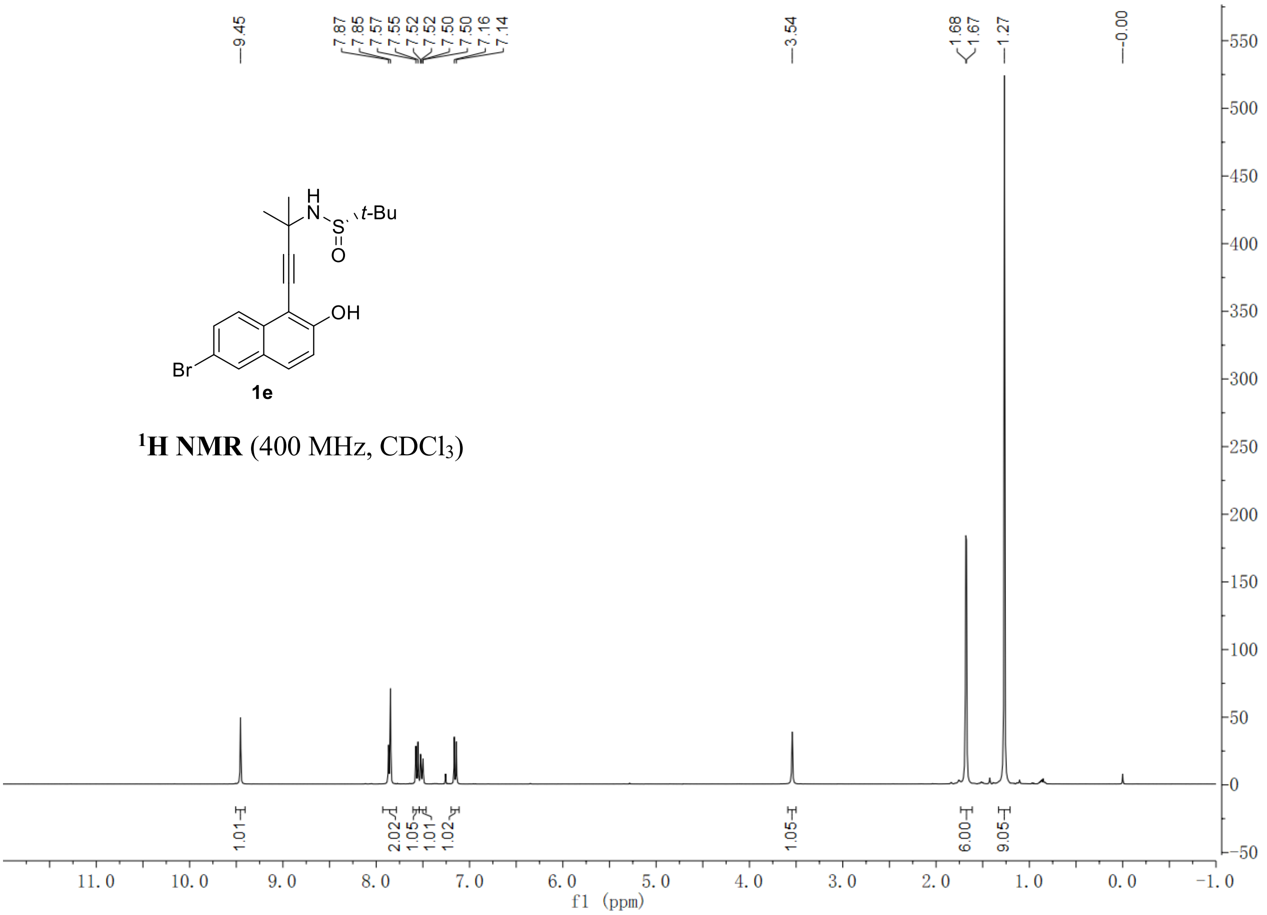

^{1}H NMR (400 MHz, $CDCl_3$)

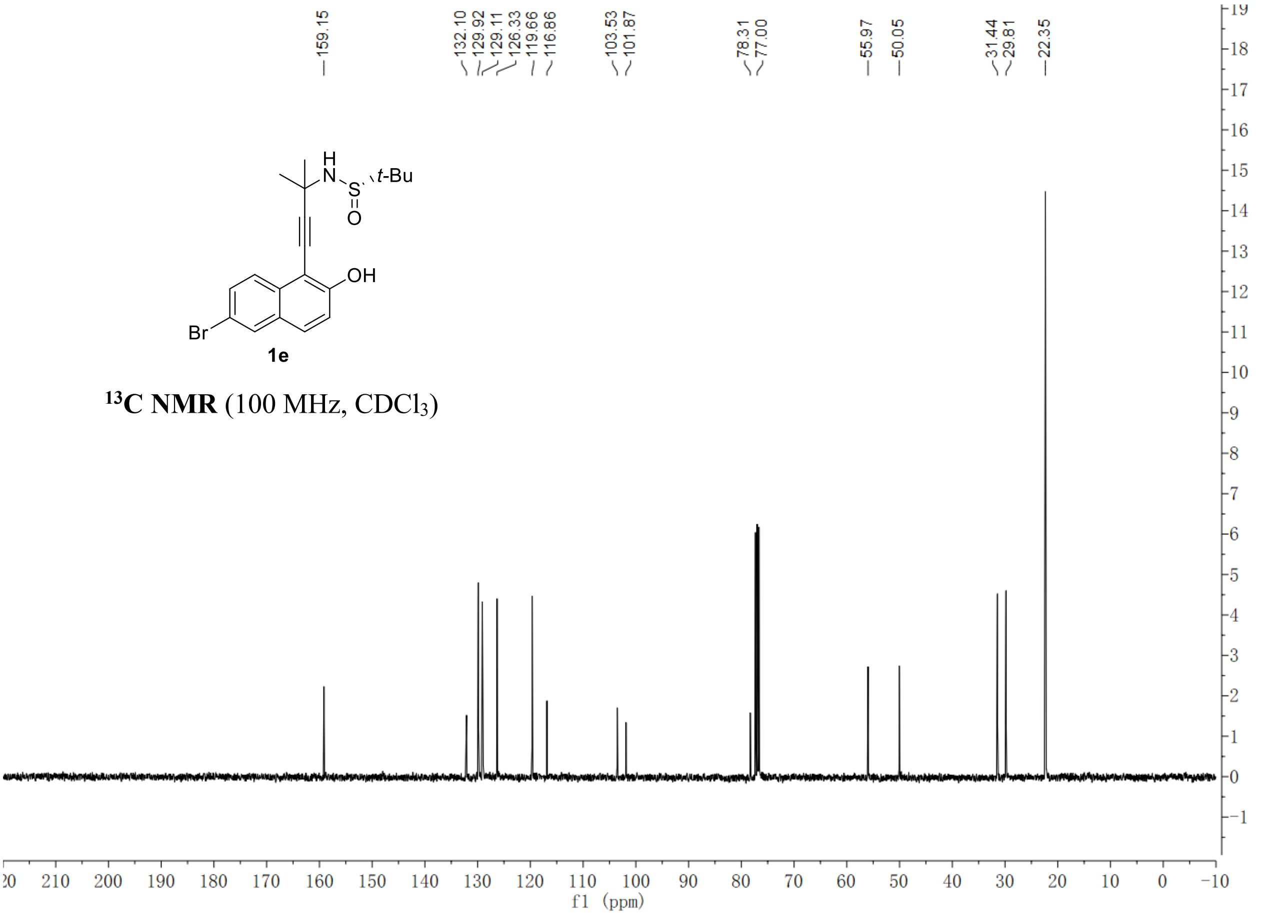

^{13}C NMR (100 MHz, $CDCl_3$)

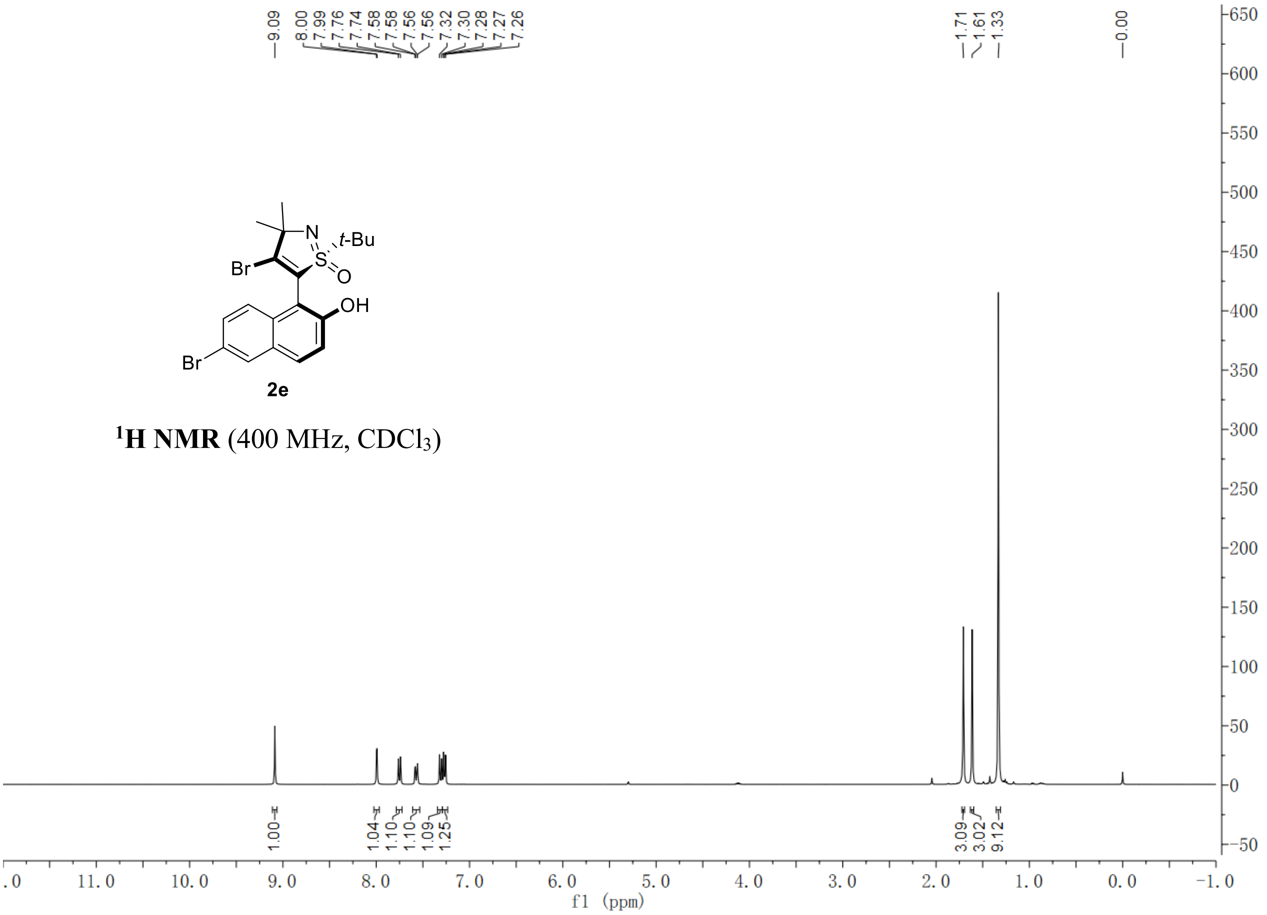

2e

^{1}H NMR (400 MHz, $CDCl_3$)

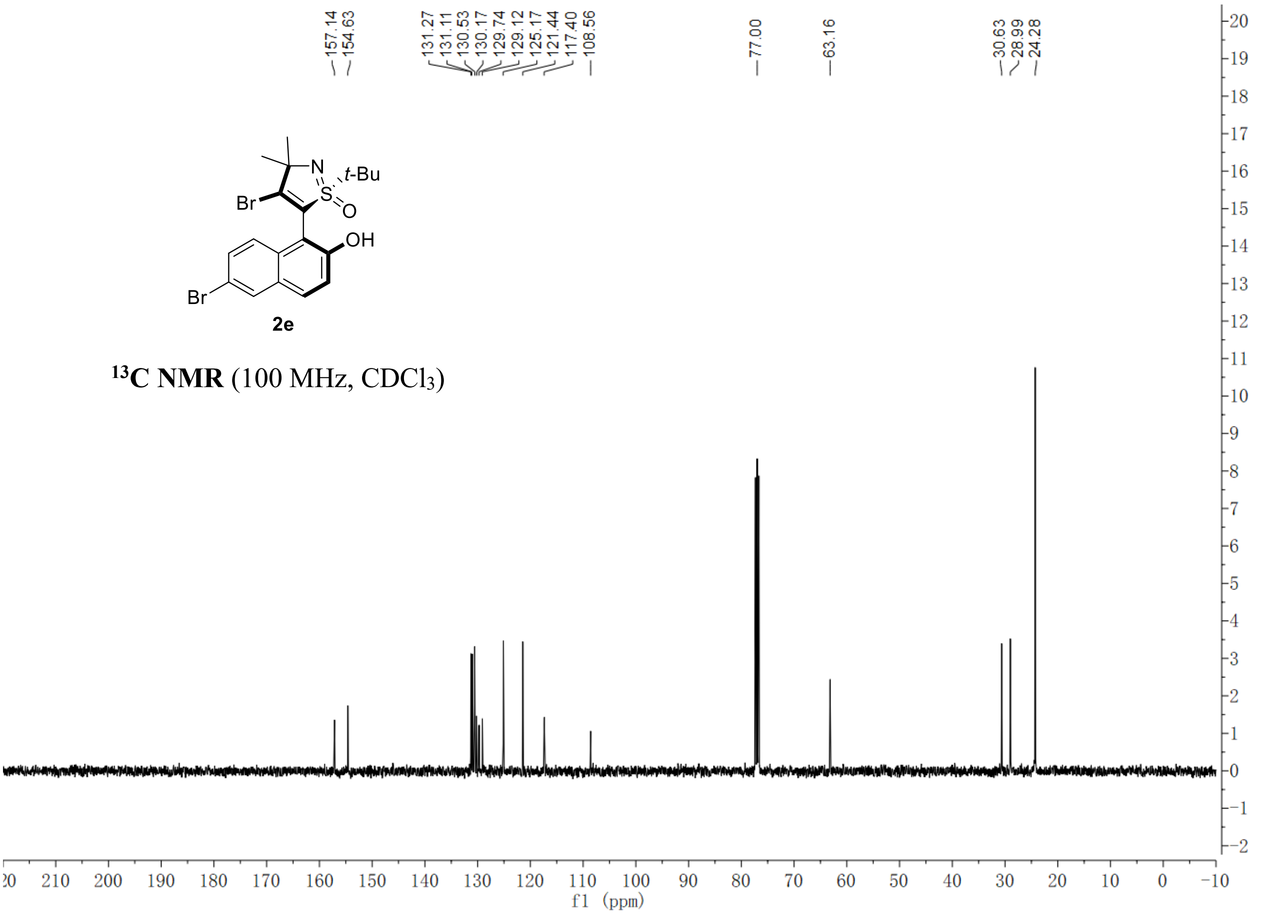

2e

^{13}C NMR (100 MHz, $CDCl_3$)

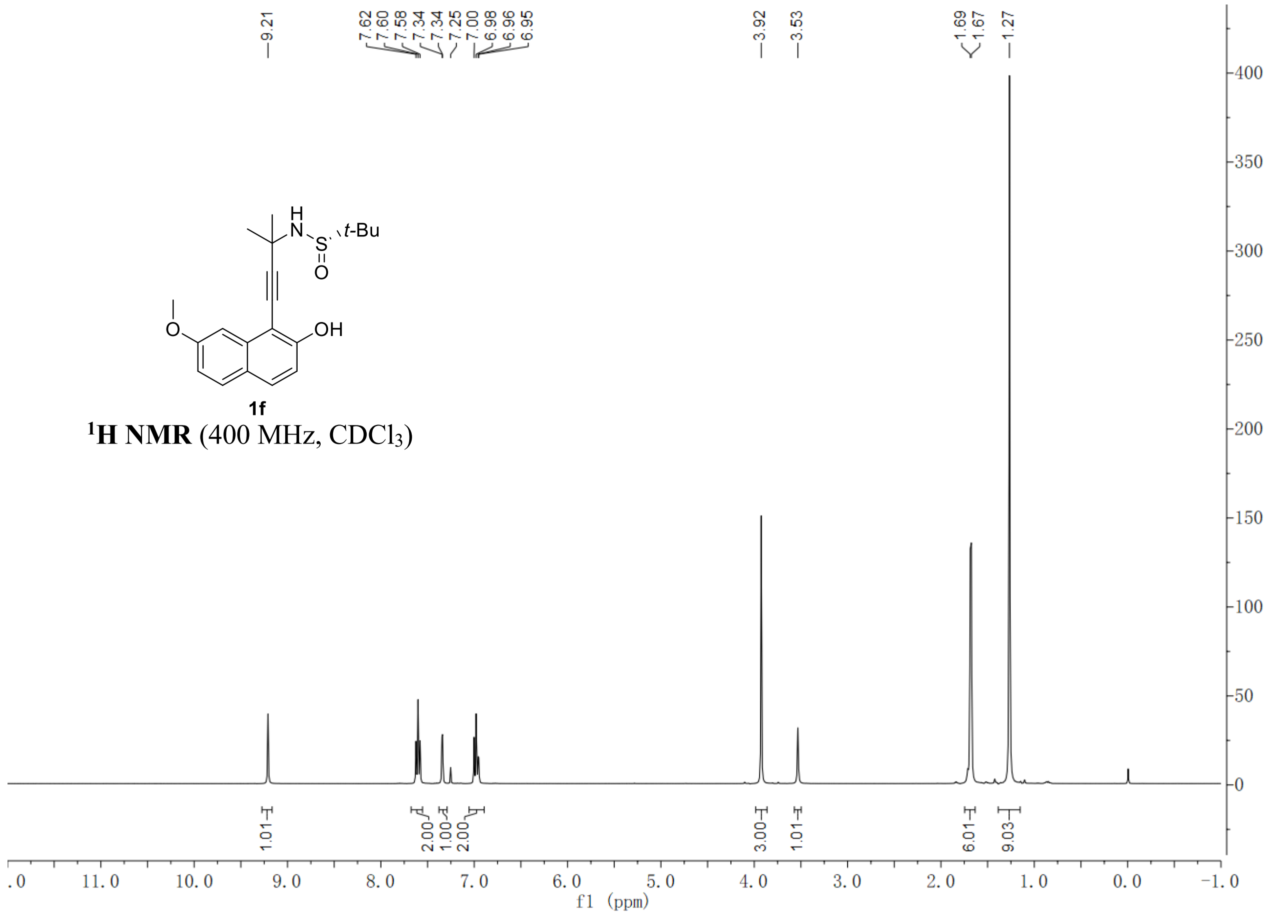

1f

^{1}H NMR (400 MHz, $CDCl_3$)

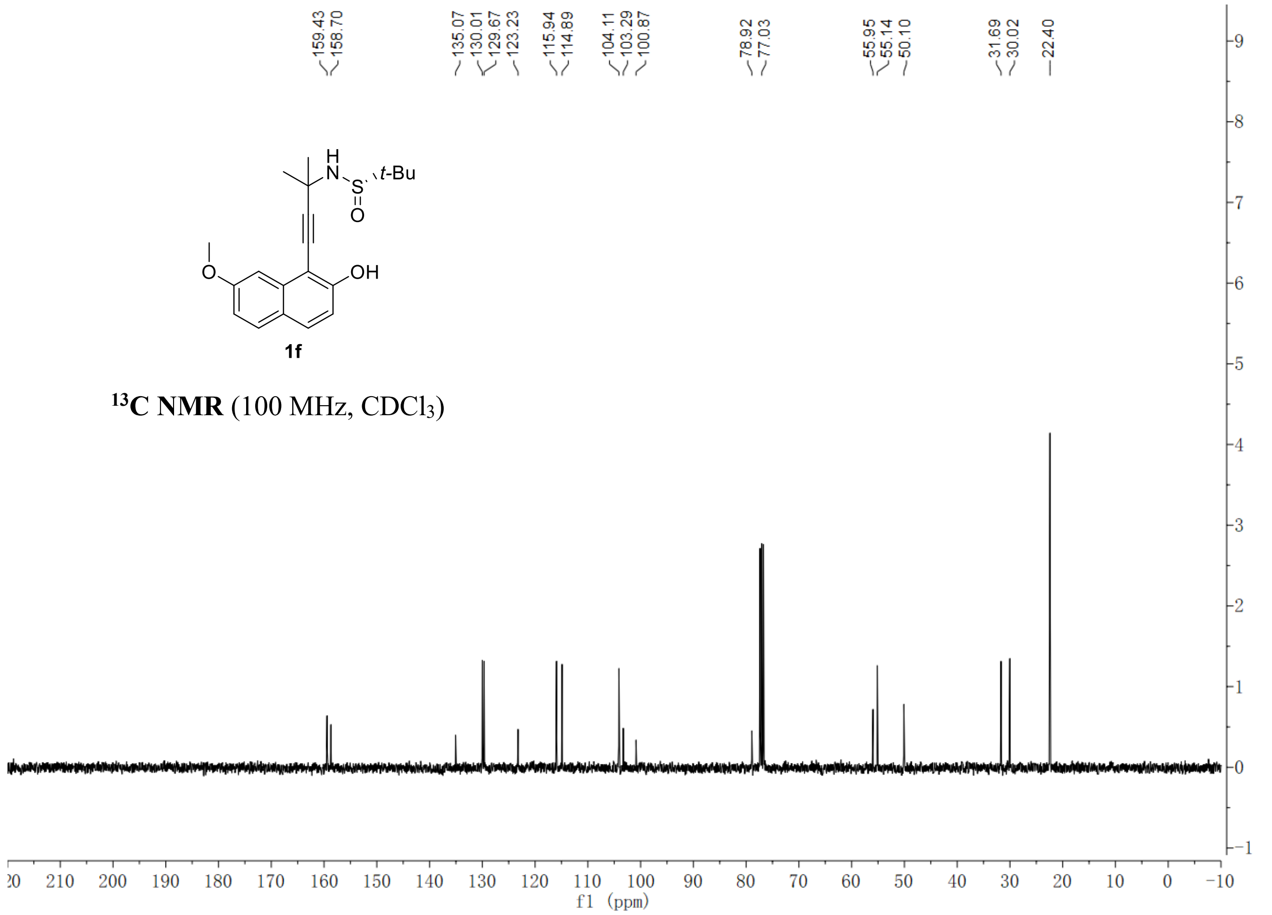

1f

^{13}C NMR (100 MHz, $CDCl_3$)

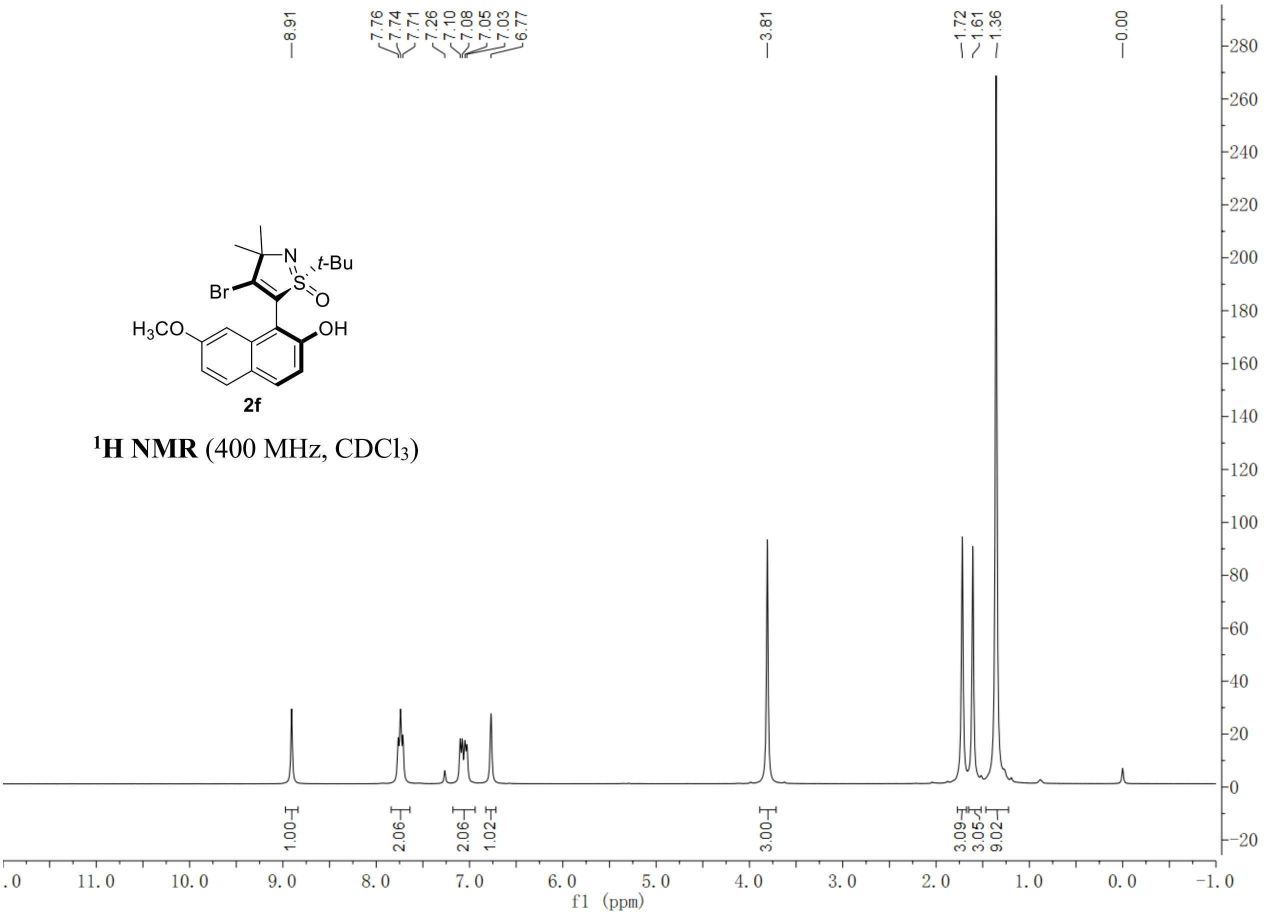
8.91
7.76
7.74
7.71
7.26
7.10
7.08
7.05
7.03
6.77
3.81
1.72
1.61
1.36
0.00
t-Bu
Br
H3CO
OH
2f
1H NMR (400 MHz, CDCl3)
1.00
2.06
2.06
1.02
3.00
3.09
3.05
9.02
f1 (ppm)

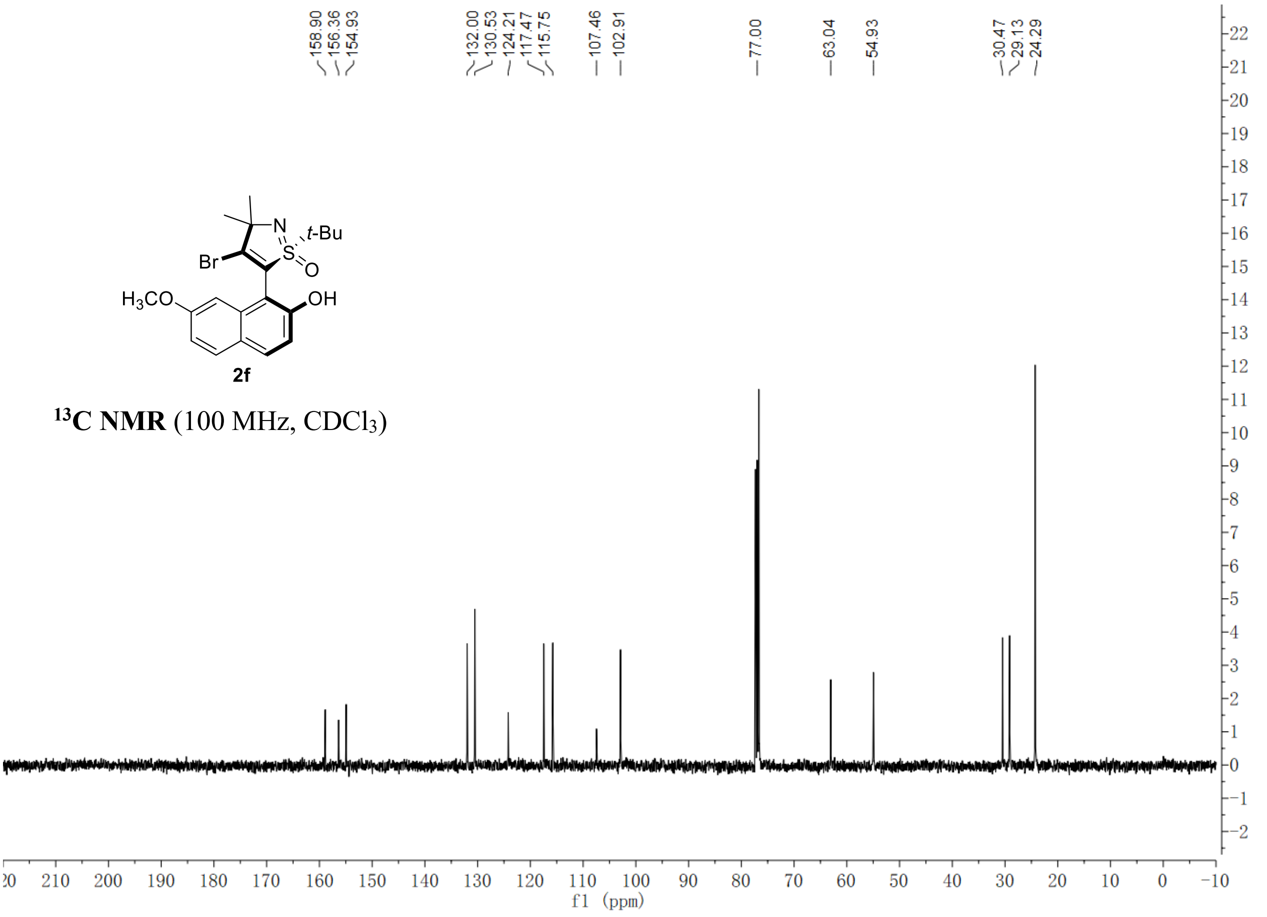
158.90
156.36
154.93
132.00
130.53
124.21
117.47
115.75
107.46
102.91
77.00
63.04
54.93
30.47
29.13
24.29
t-Bu
Br
H3CO
OH
2f
13C NMR (100 MHz, CDCl3)
f1 (ppm)

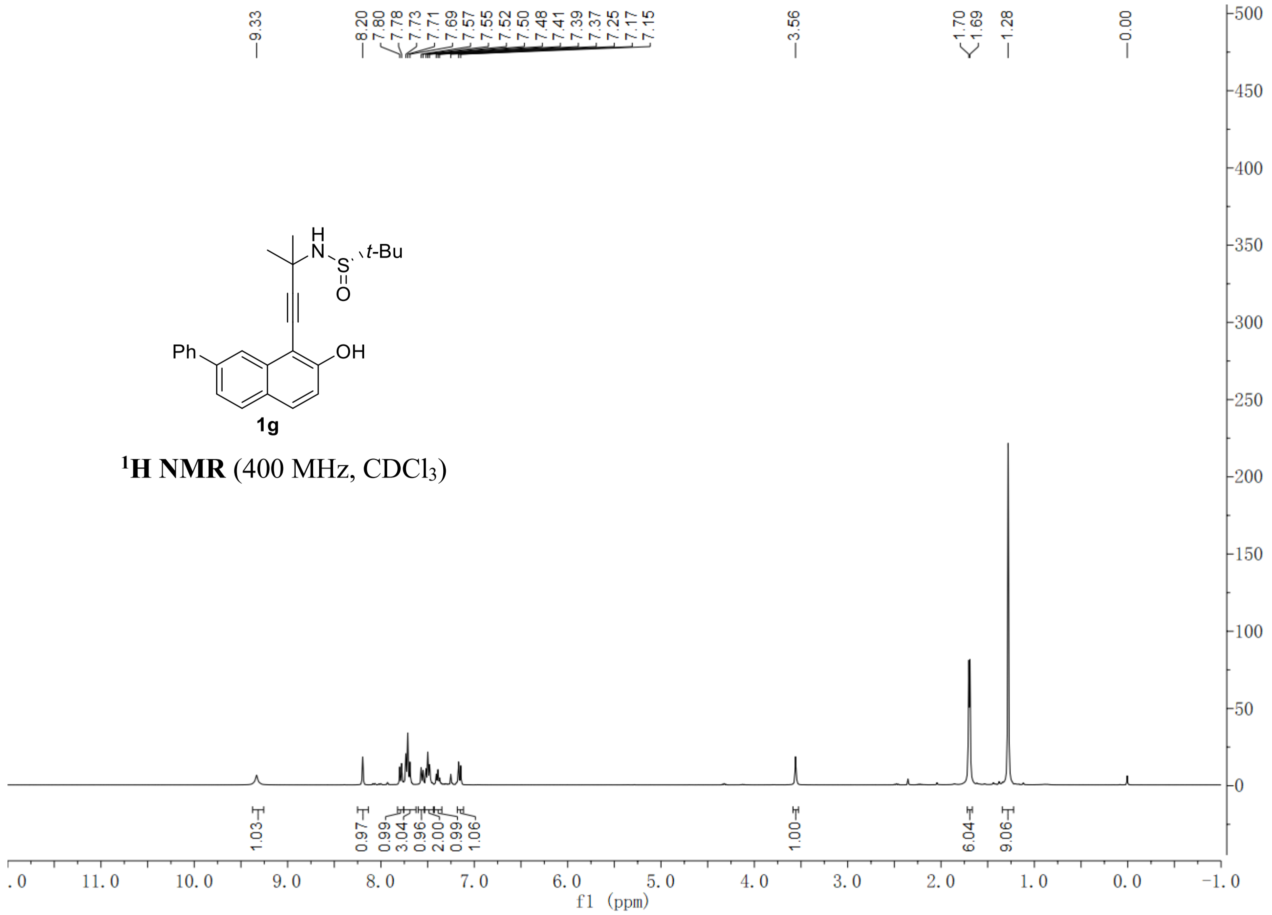

^{1}**H NMR** (400 MHz, $CDCl_3$)

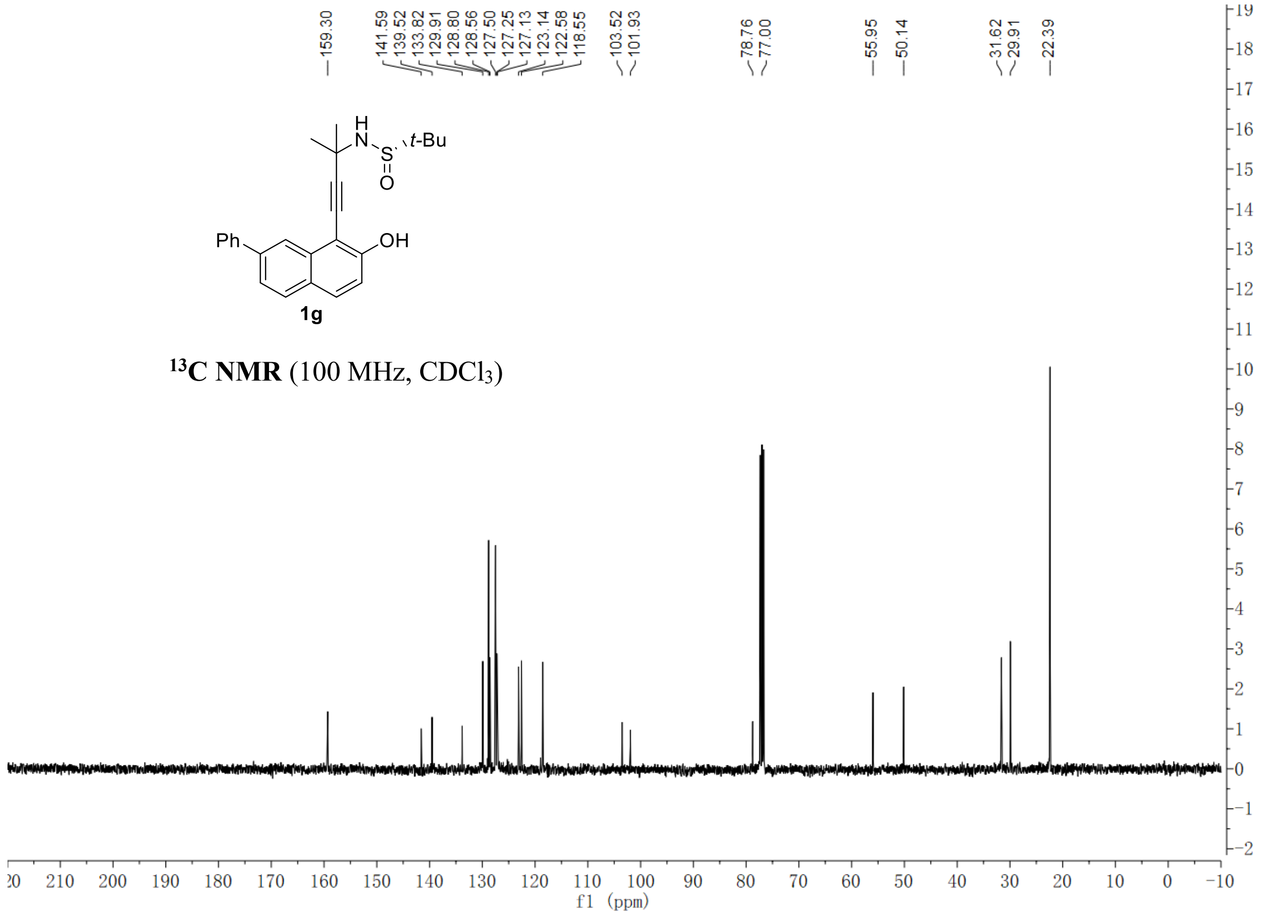

^{13}**C NMR** (100 MHz, $CDCl_3$)

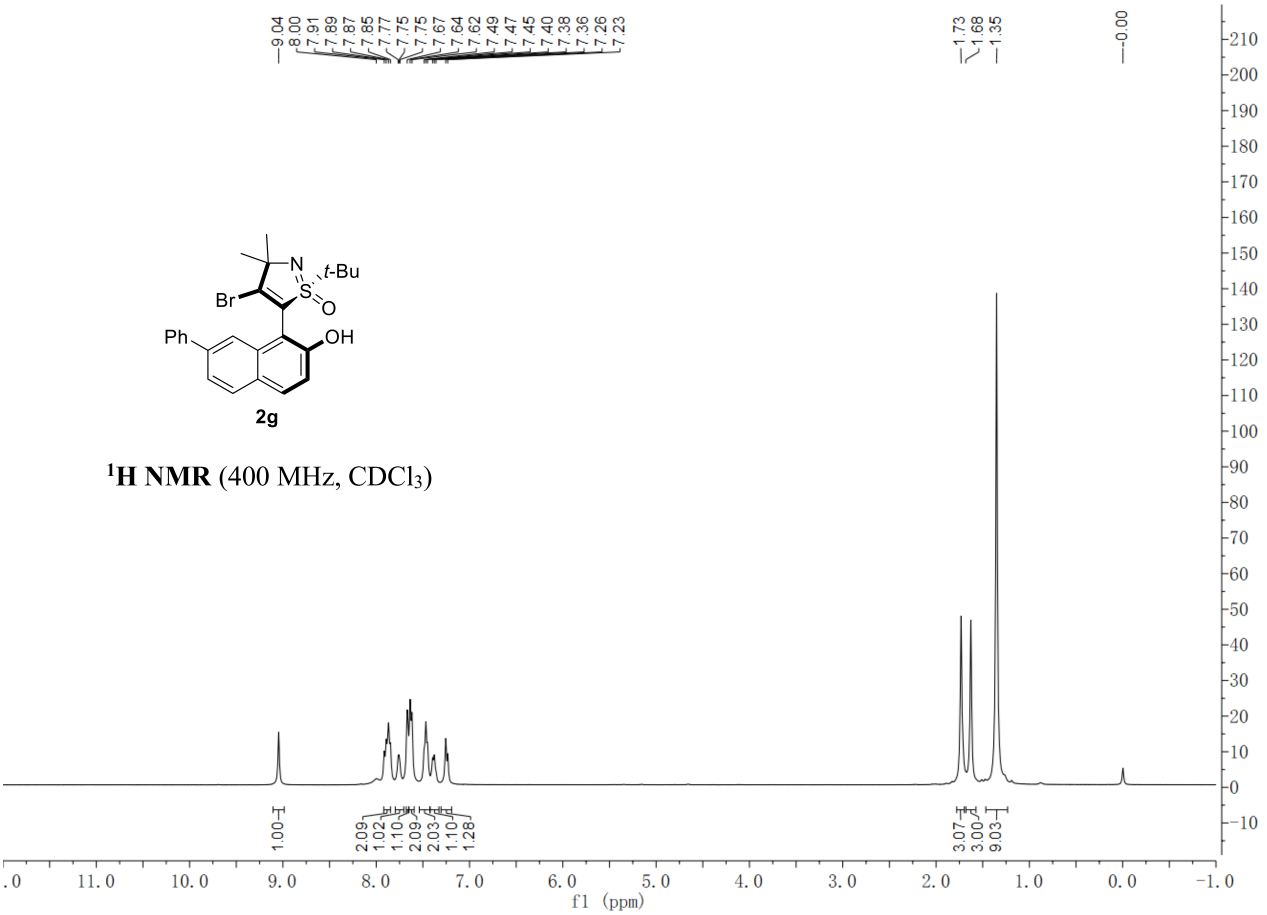

^{1}H NMR (400 MHz, $CDCl_3$)

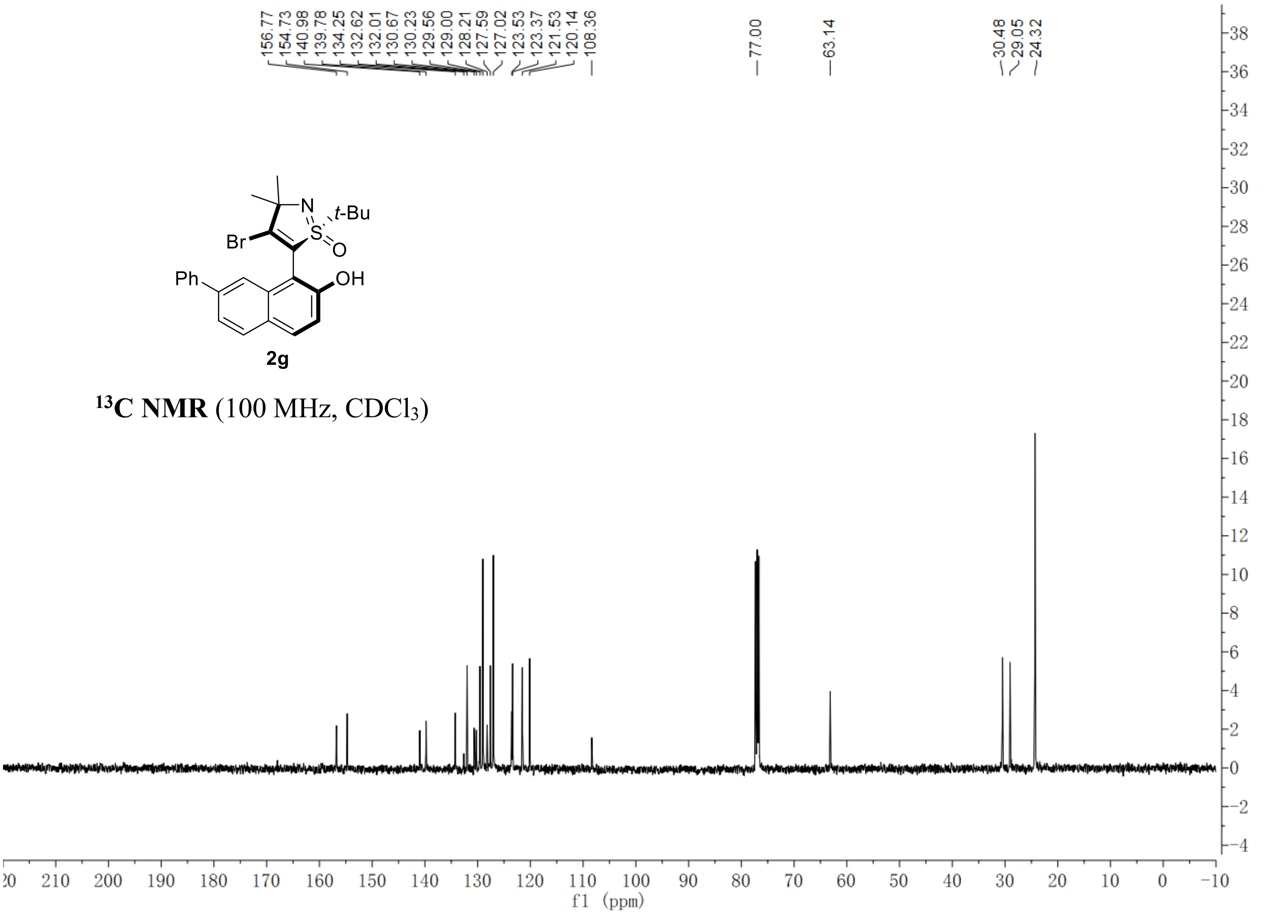

^{13}C NMR (100 MHz, $CDCl_3$)

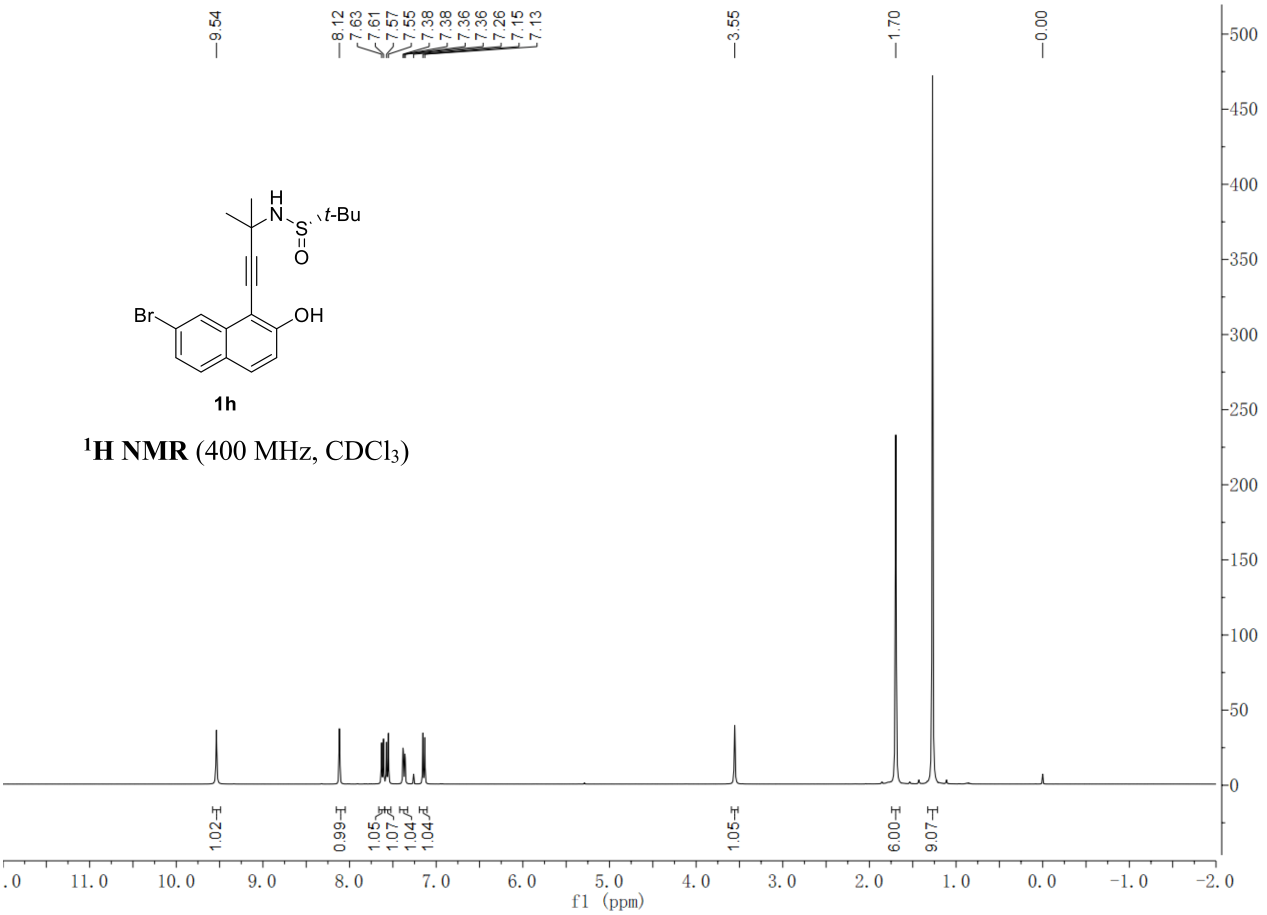

1h

^{1}H NMR (400 MHz, $CDCl_3$)

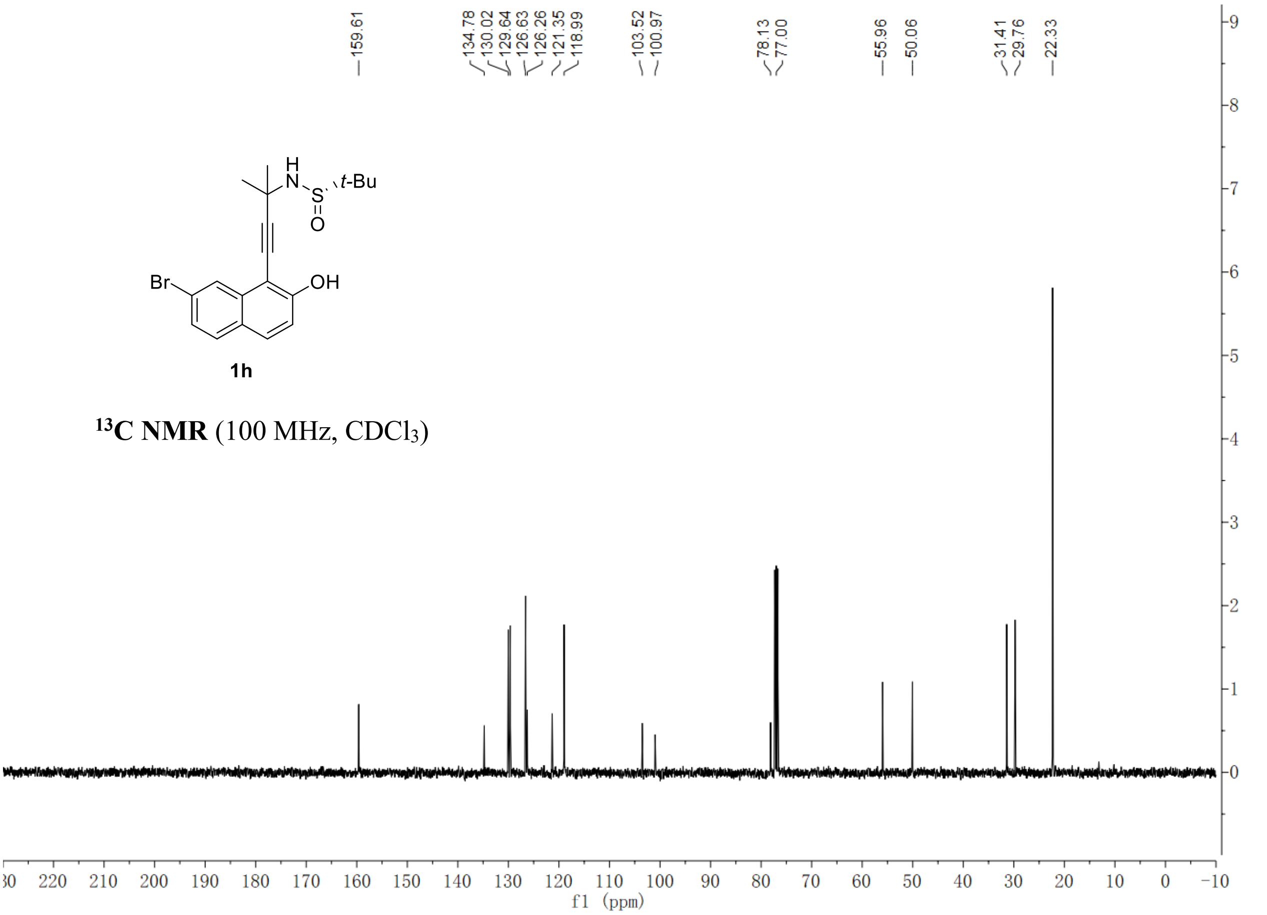

1h

^{13}C NMR (100 MHz, $CDCl_3$)

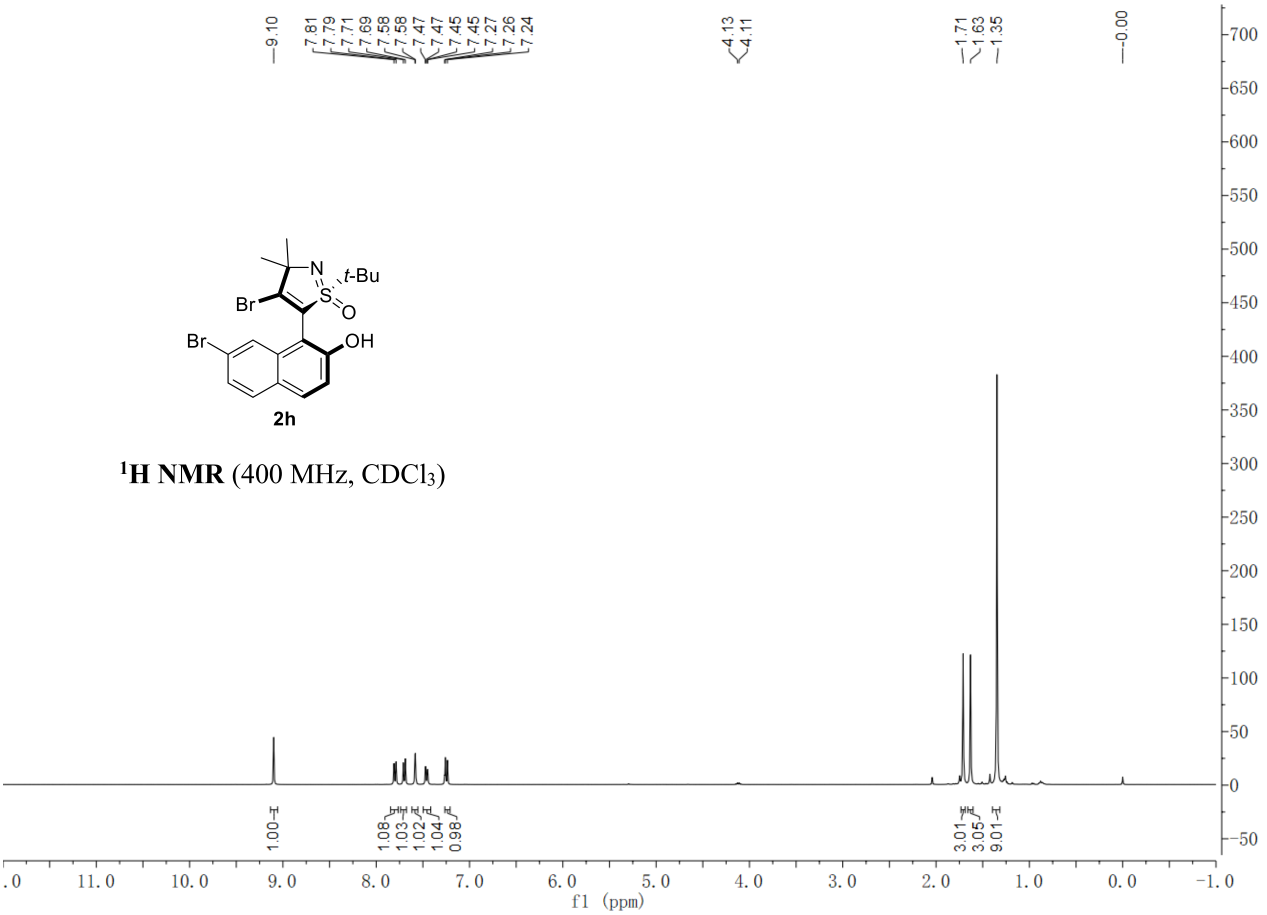

^{1}**H NMR** (400 MHz, $CDCl_3$)

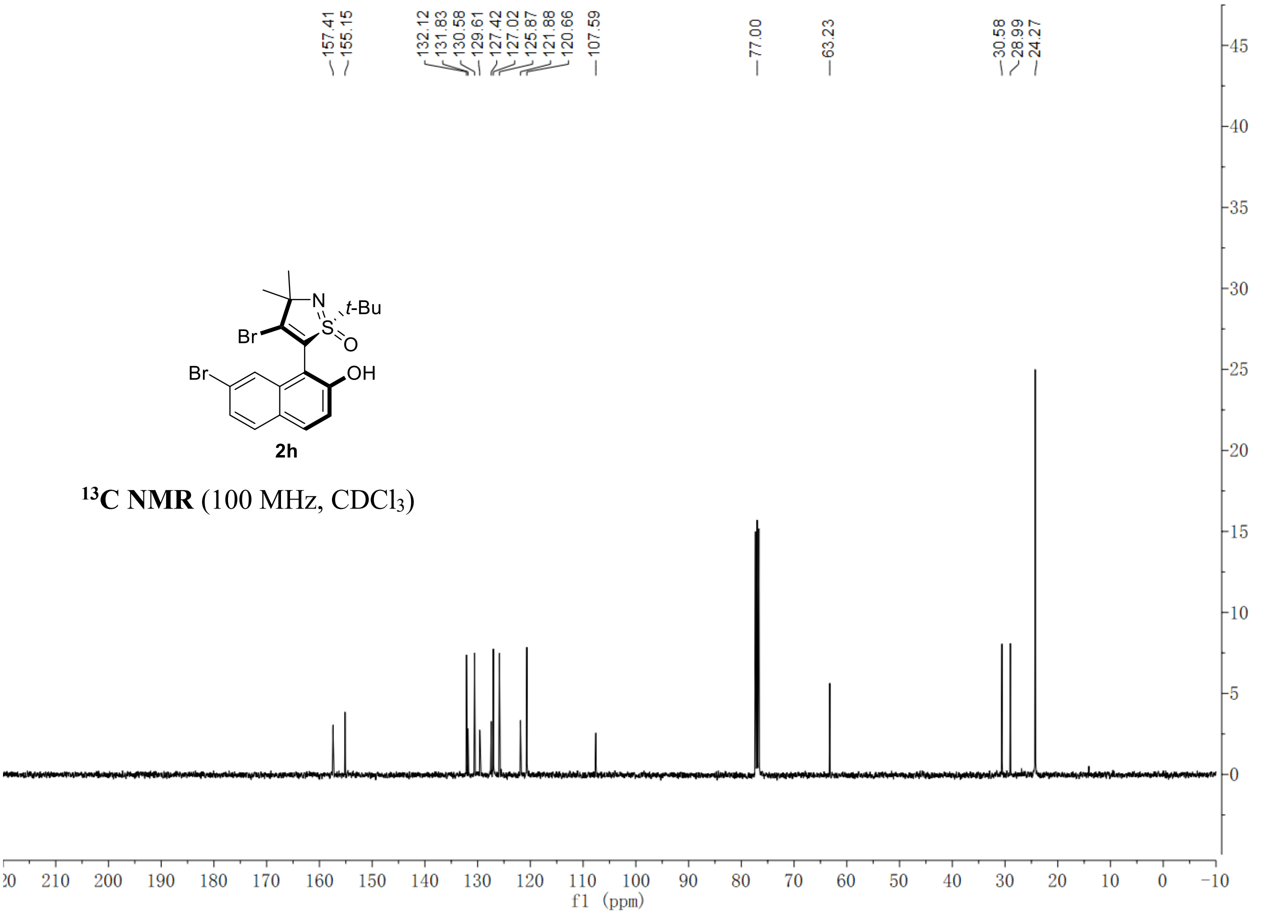

^{13}**C NMR** (100 MHz, $CDCl_3$)

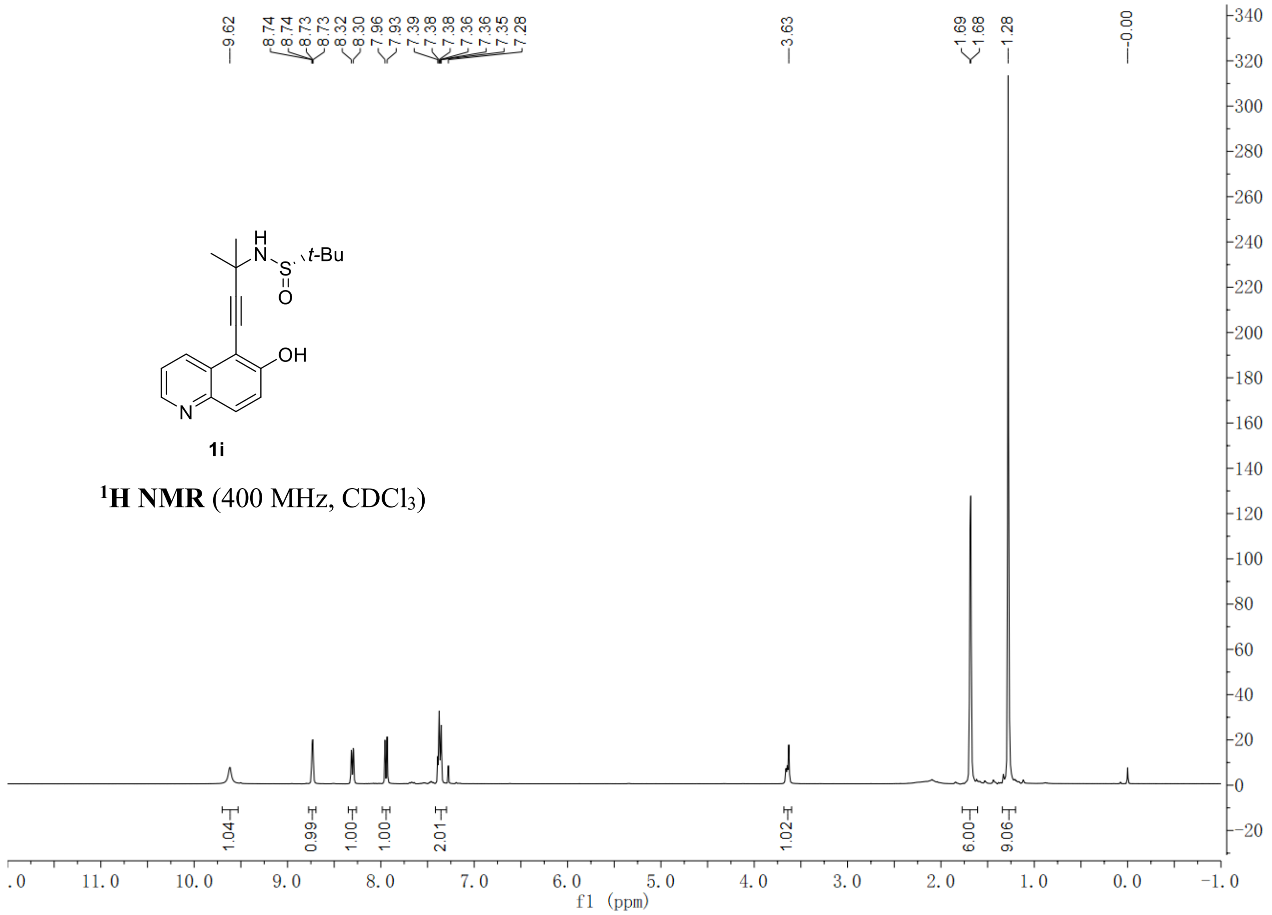

^{1}H NMR (400 MHz, $CDCl_3$)

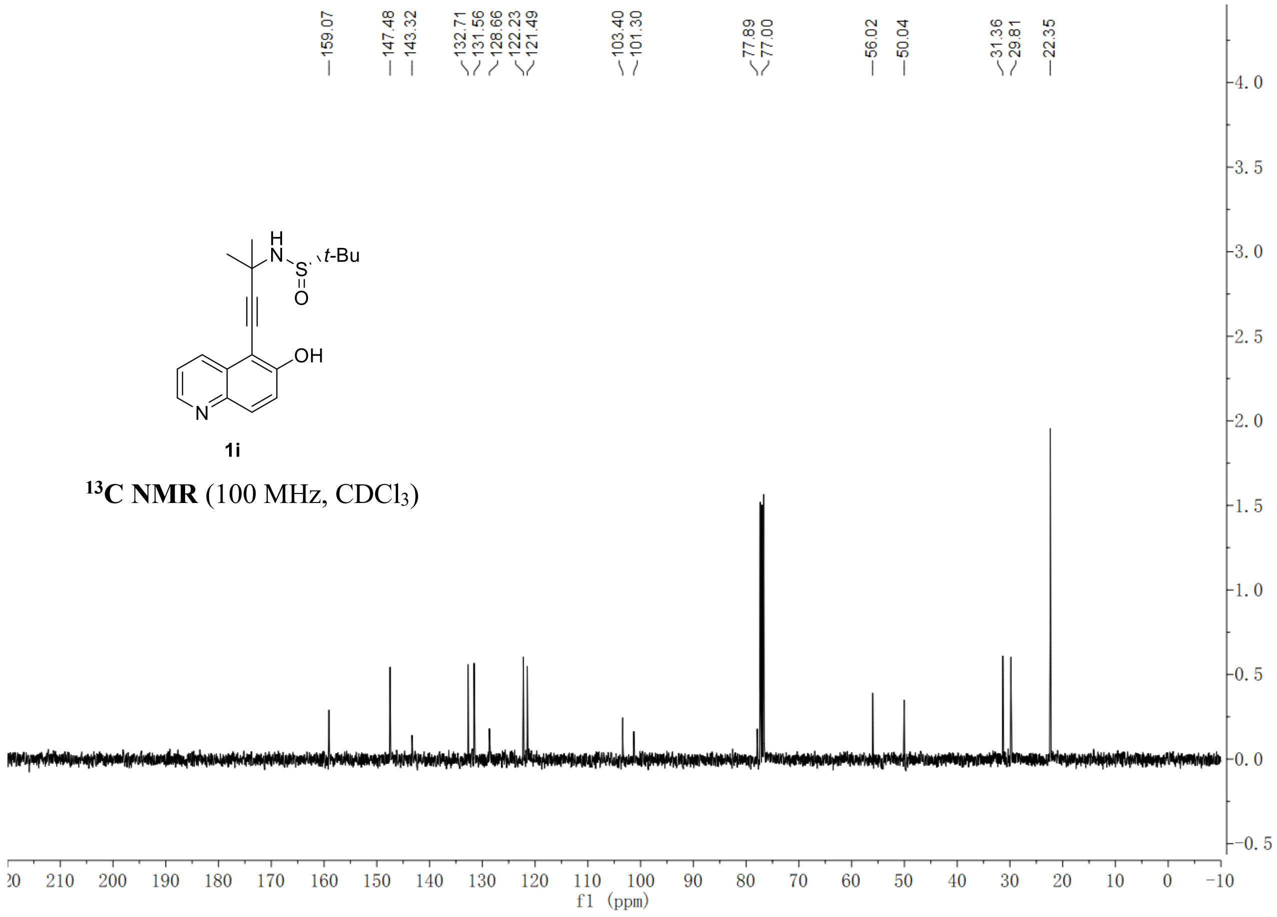

^{13}C NMR (100 MHz, $CDCl_3$)

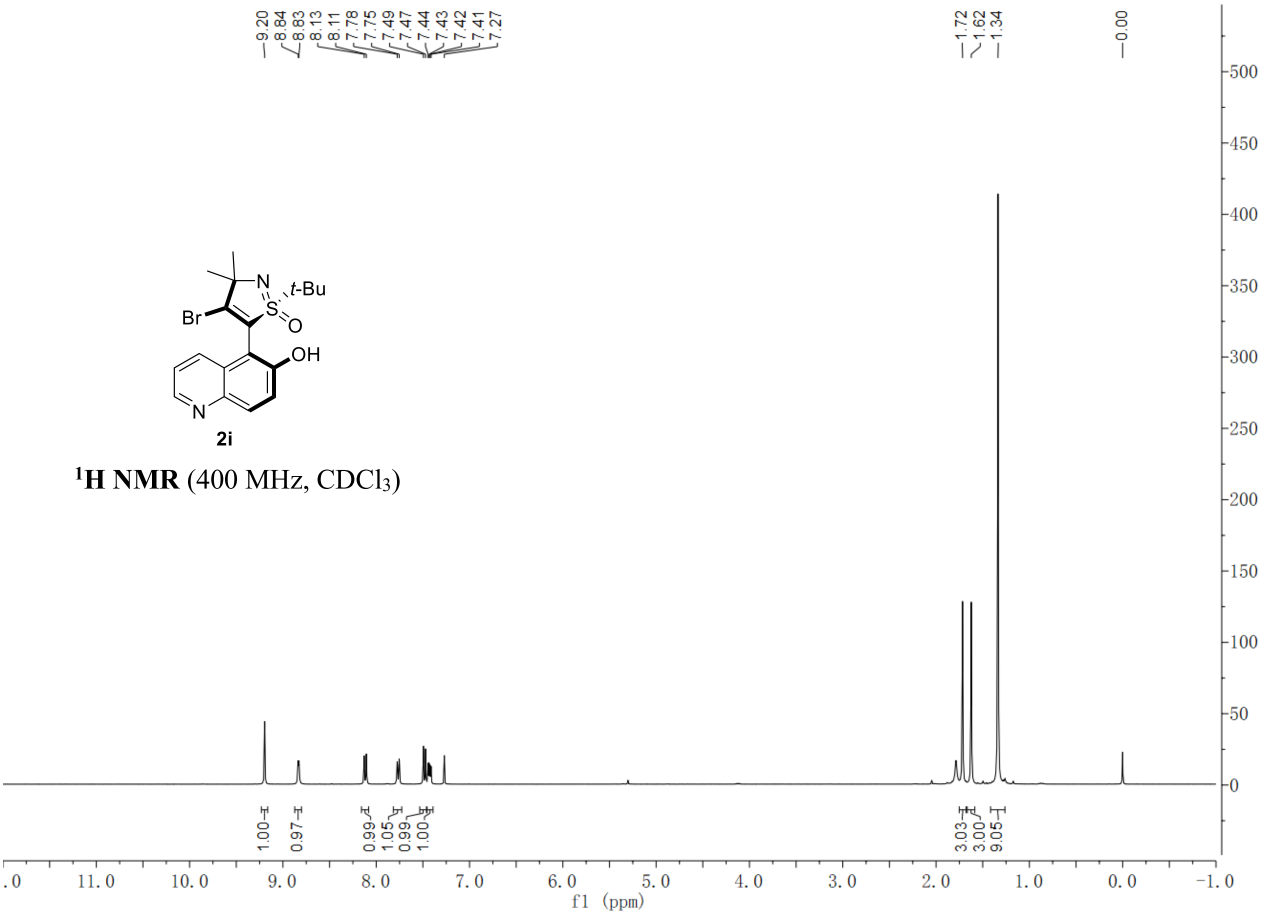

2i

¹H NMR (400 MHz, $CDCl_3$)

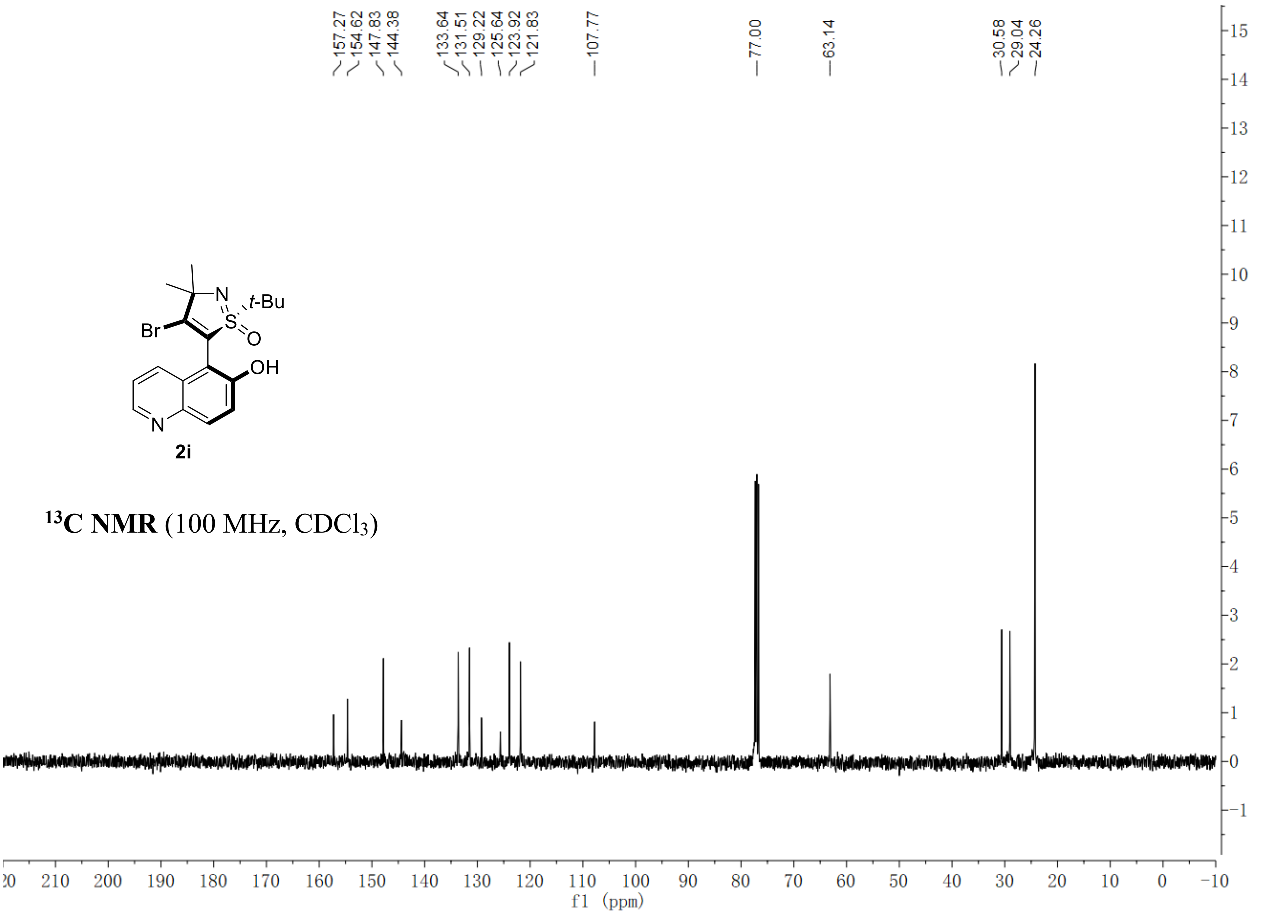

2i

¹³C NMR (100 MHz, $CDCl_3$)

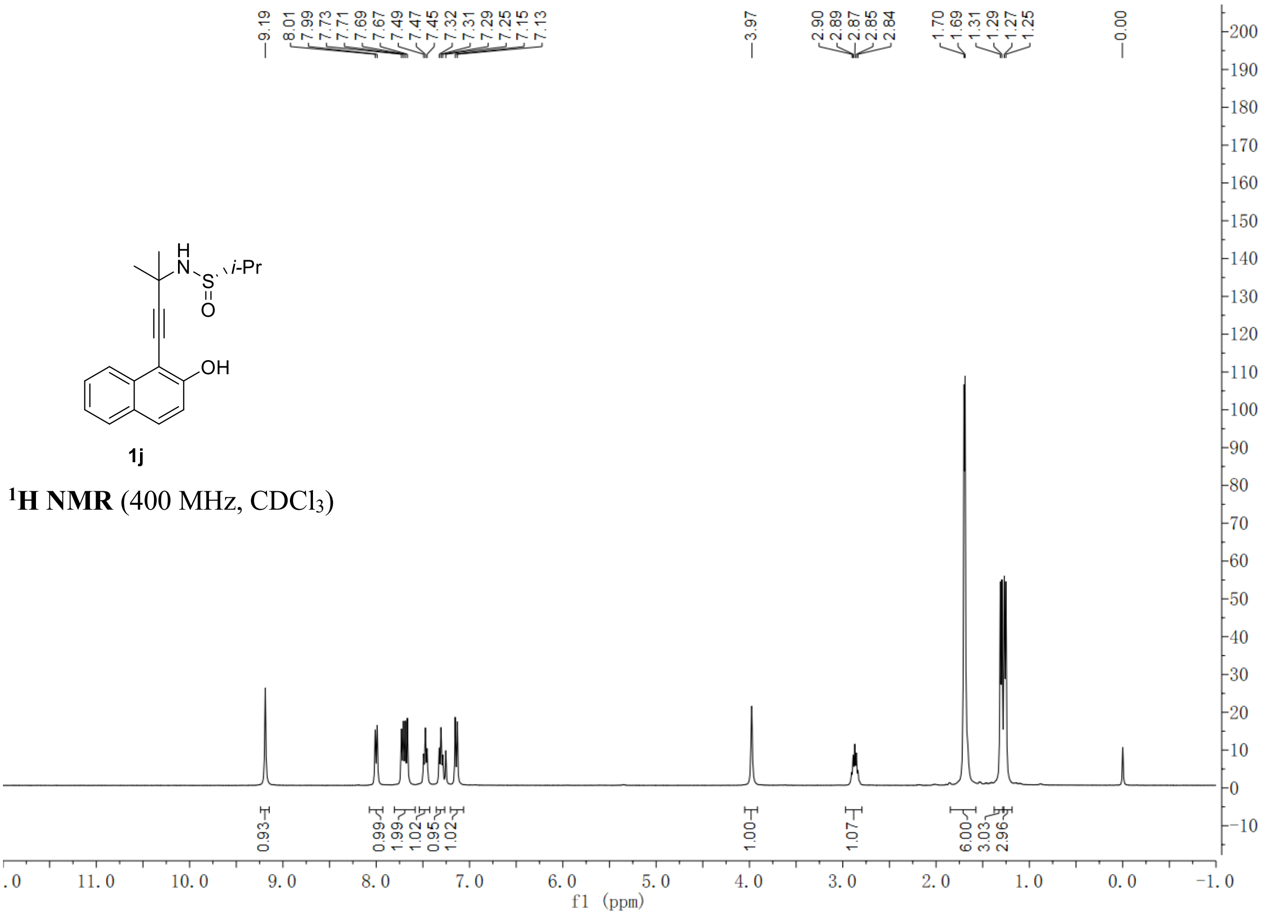

1j

[1]**H NMR** (400 MHz, $CDCl_3$)

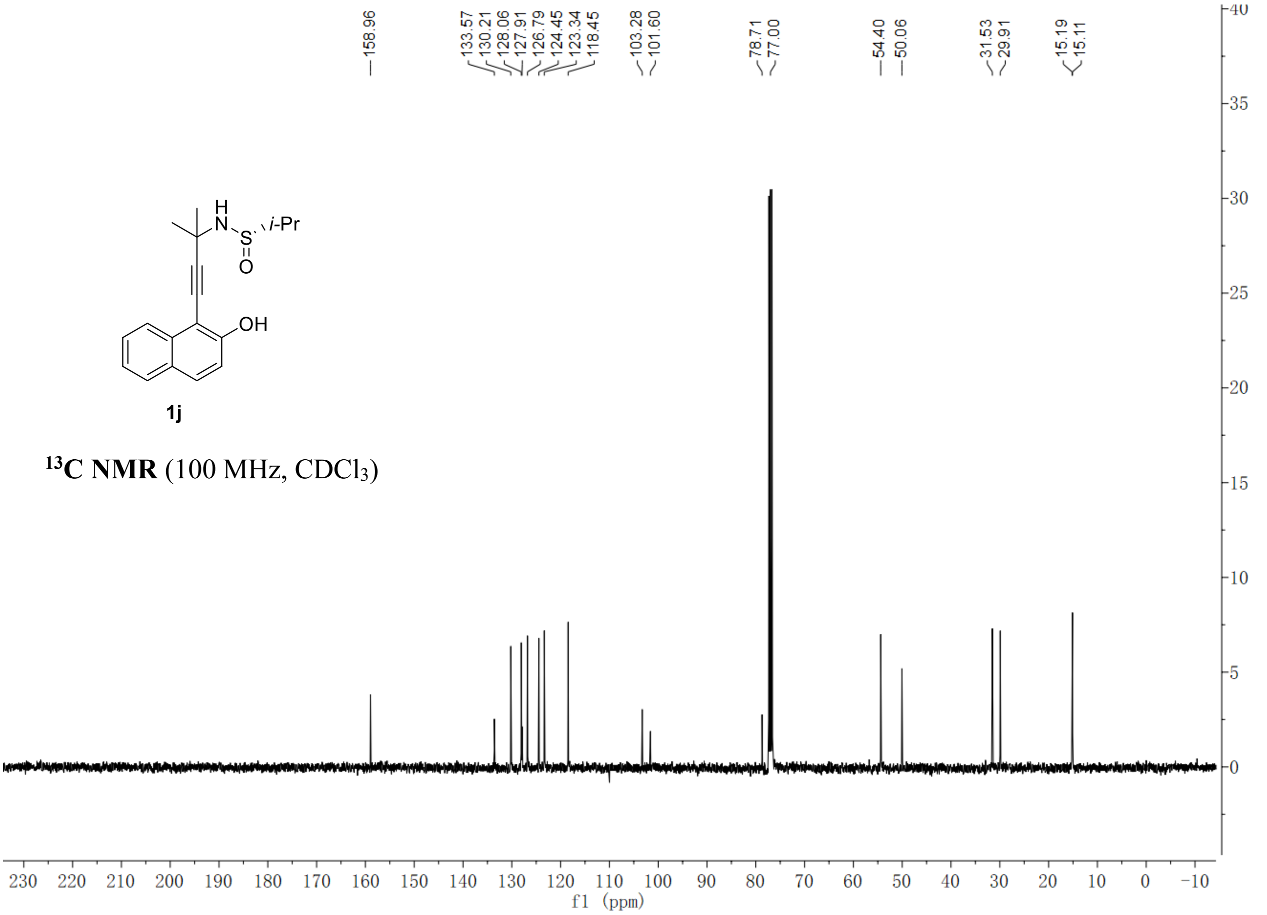

1j

[13]**C NMR** (100 MHz, $CDCl_3$)

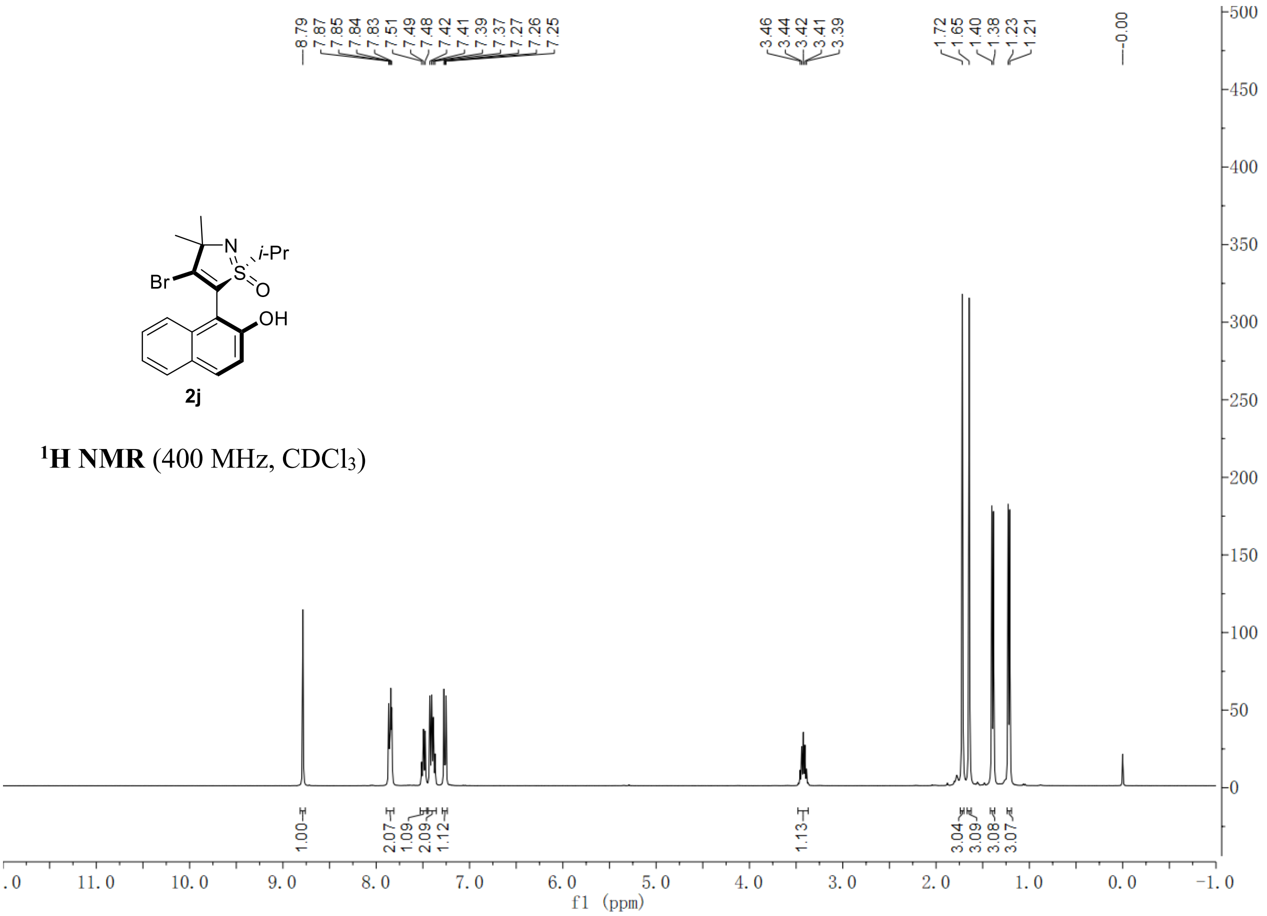

^{1}H NMR (400 MHz, $CDCl_3$)

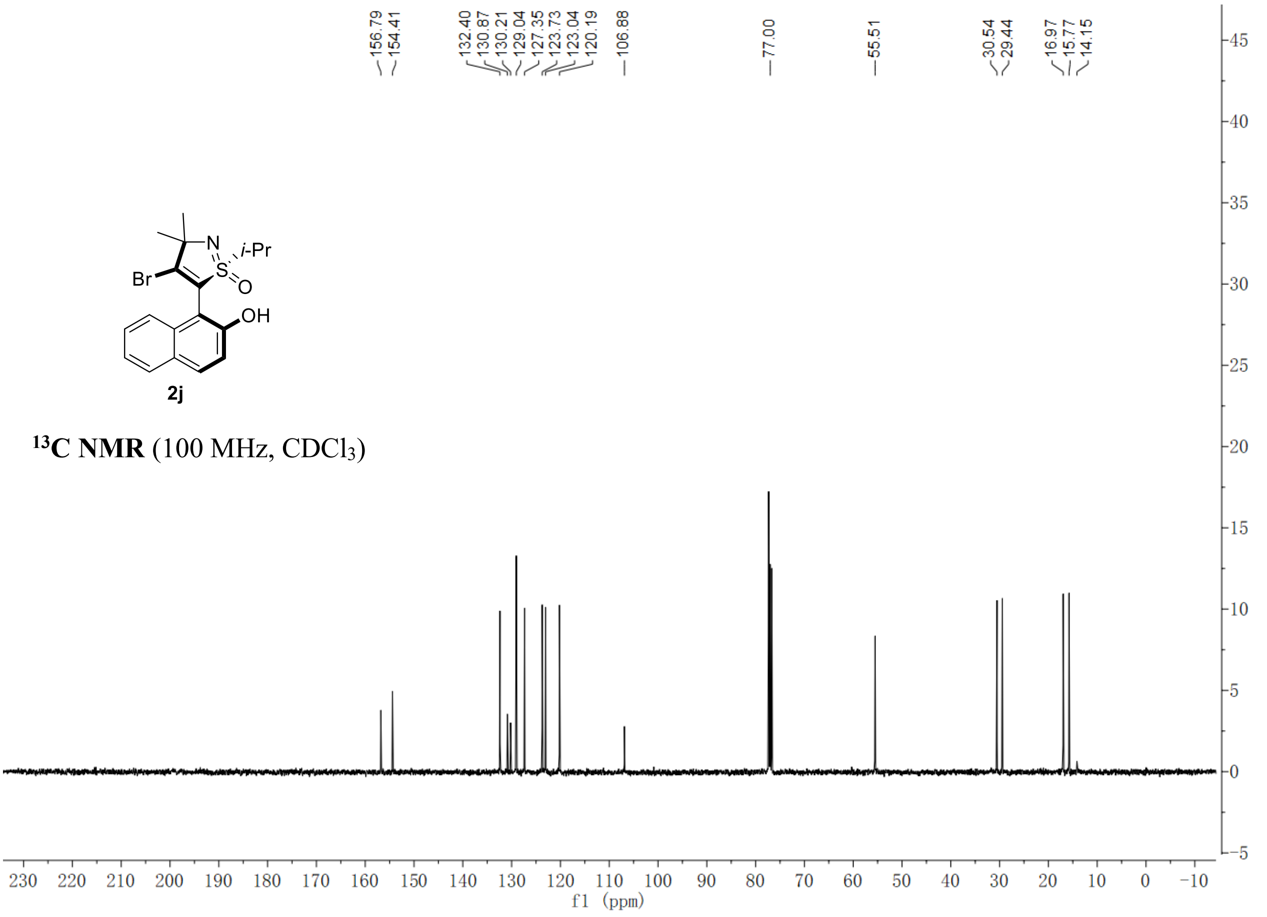

^{13}C NMR (100 MHz, $CDCl_3$)

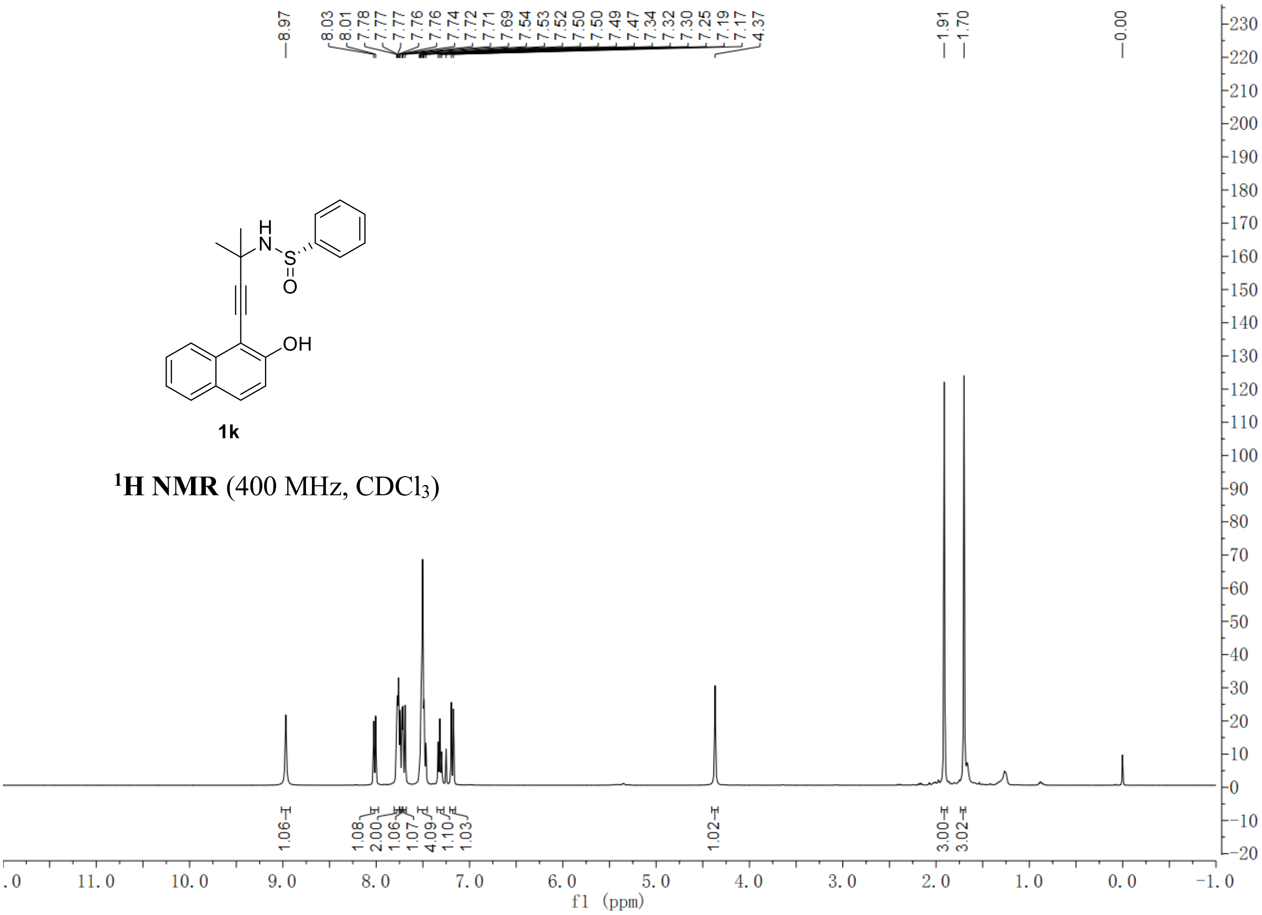
8.97
8.03
8.01
7.78
7.77
7.77
7.76
7.76
7.74
7.72
7.71
7.69
7.54
7.53
7.52
7.50
7.50
7.49
7.47
7.34
7.32
7.30
7.25
7.19
7.17
4.37
1.91
1.70
0.00
H
N
S
O
OH
1k
^{1}H NMR (400 MHz, CDCl$_3$)
1.06
1.08
2.00
1.06
1.07
4.09
1.10
1.03
1.02
3.00
3.02
f1 (ppm)

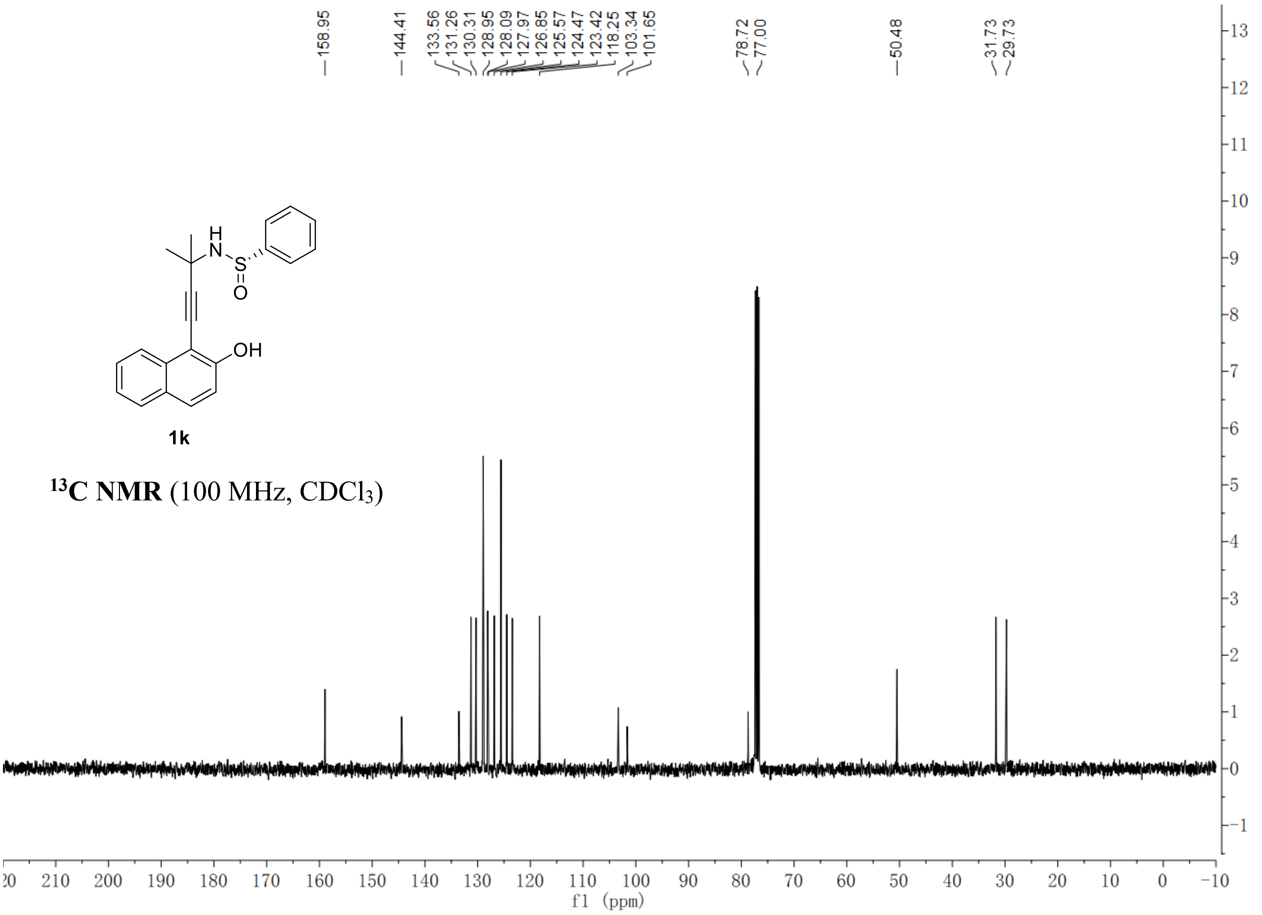
158.95
144.41
133.56
131.26
130.31
128.95
128.09
127.97
126.85
125.57
124.47
123.42
118.25
103.34
101.65
78.72
77.00
50.48
31.73
29.73
H
N
S
O
OH
1k
^{13}C NMR (100 MHz, CDCl$_3$)
f1 (ppm)

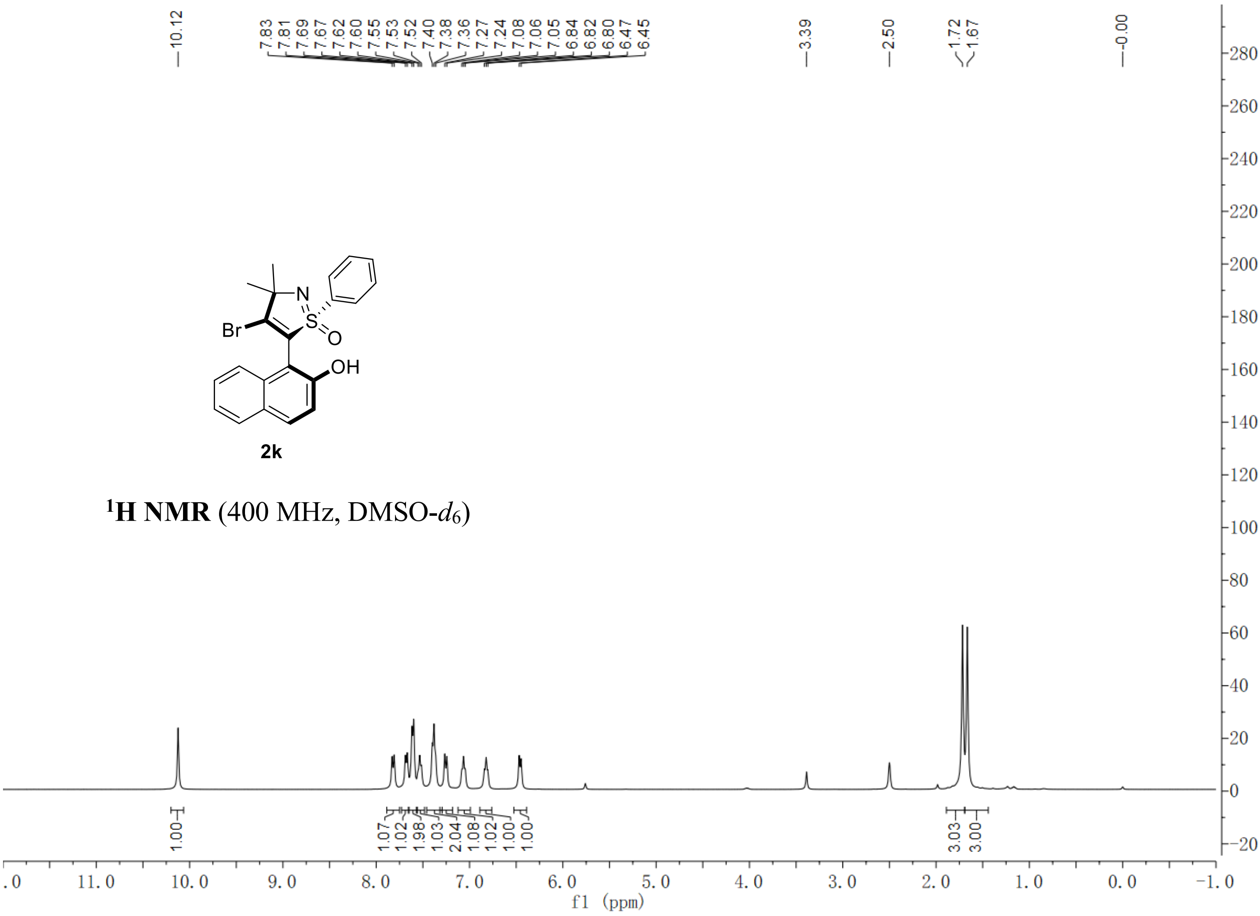

¹H NMR (400 MHz, DMSO-d_6)

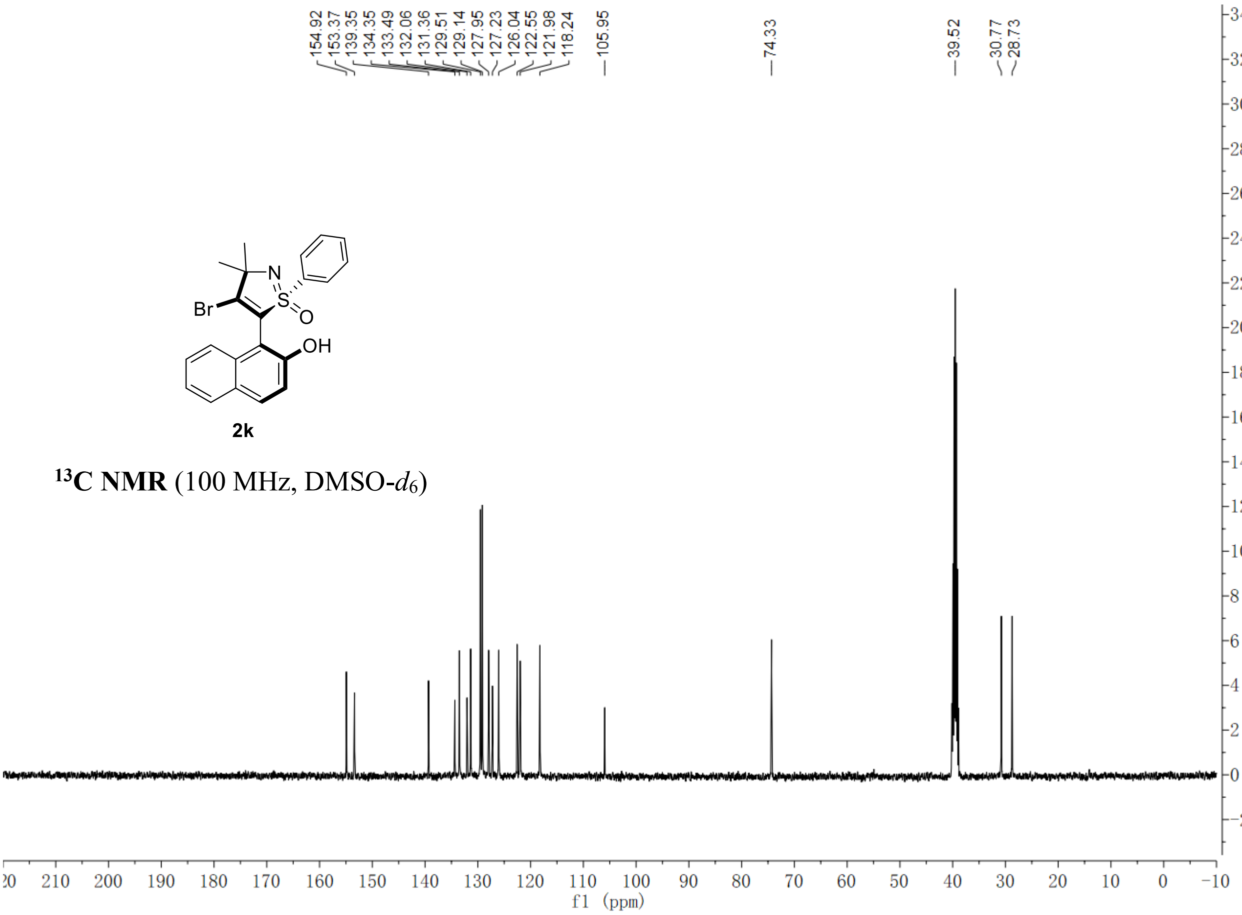

¹³C NMR (100 MHz, DMSO-d_6)

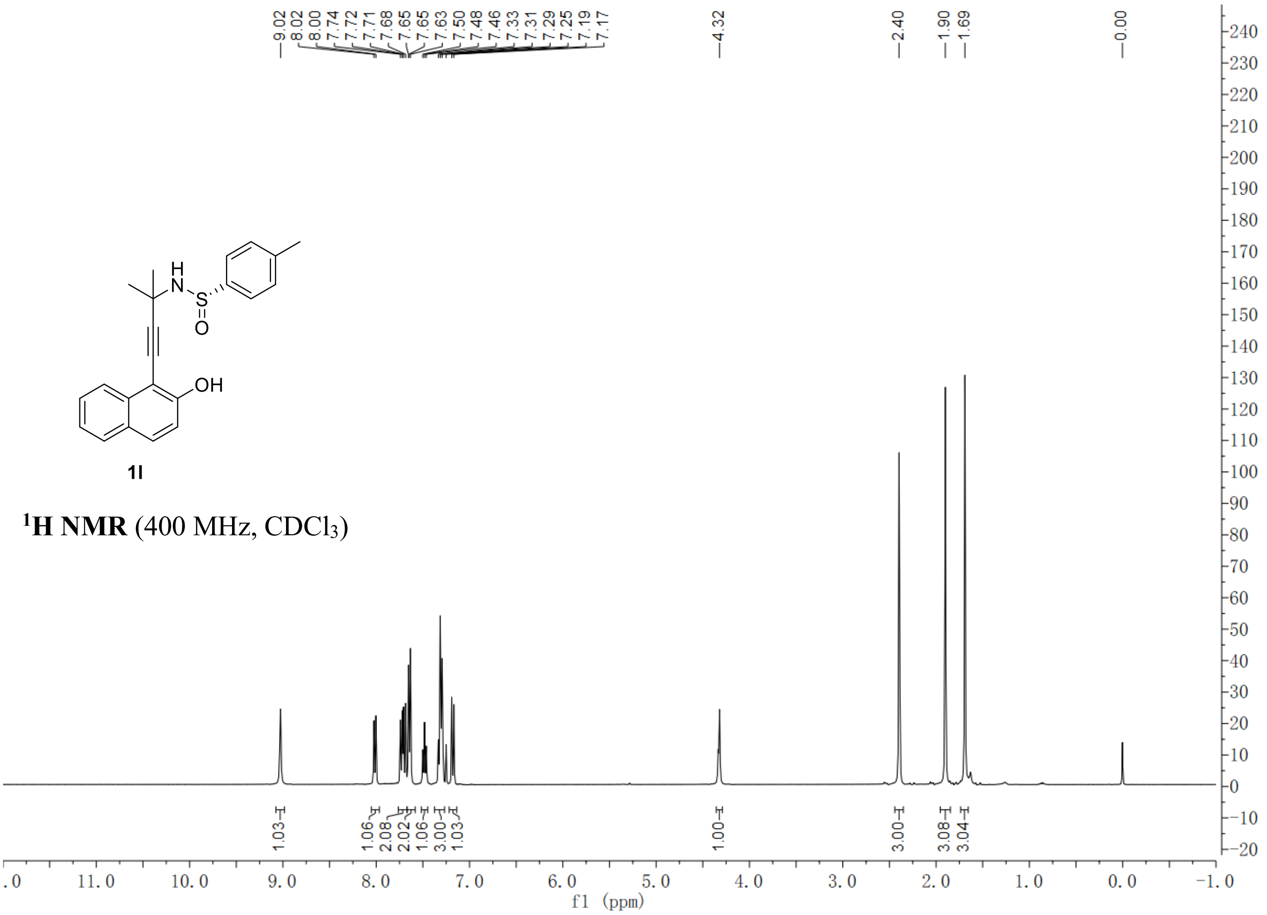

1l
^{1}H NMR (400 MHz, CDCl$_3$)

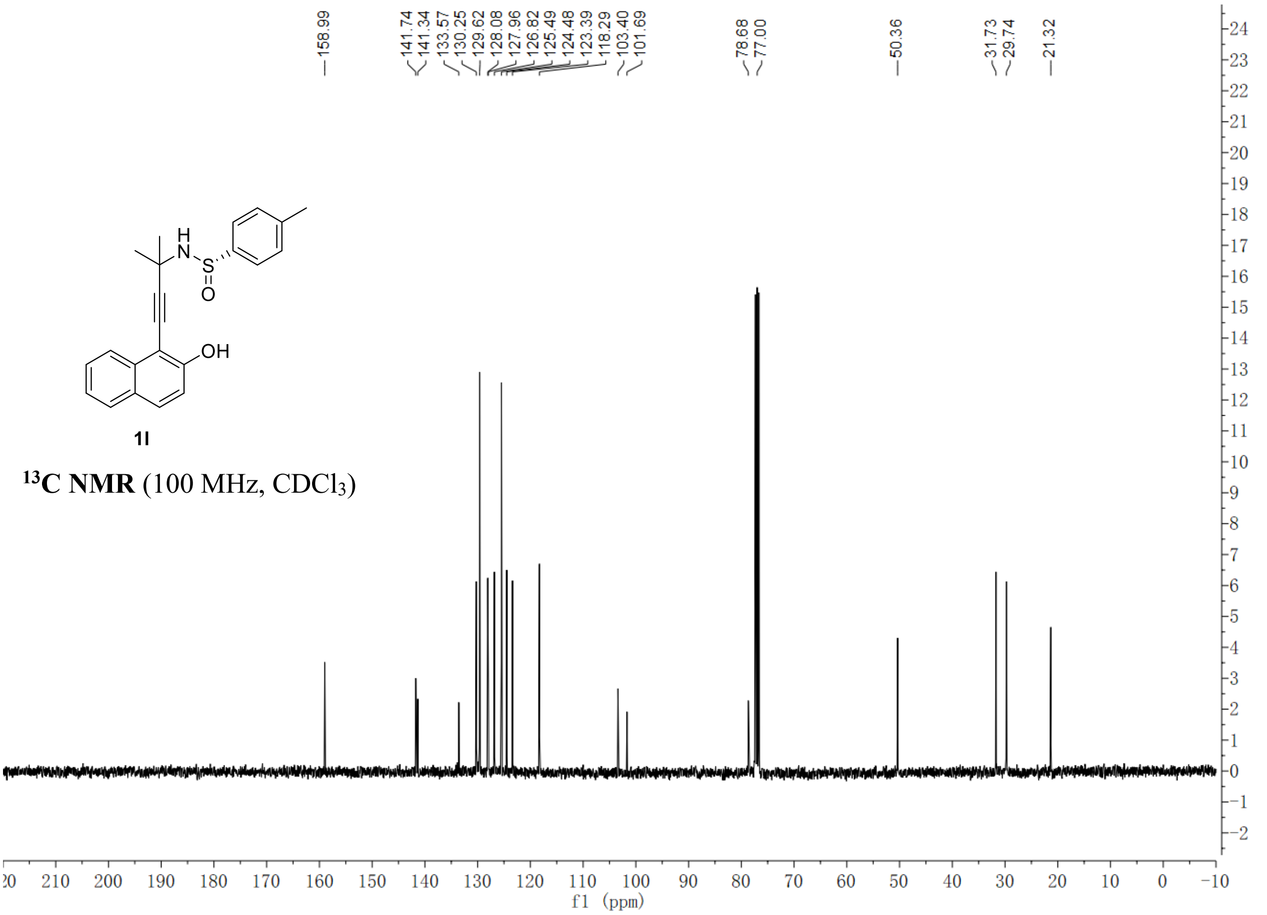

1l
^{13}C NMR (100 MHz, CDCl$_3$)

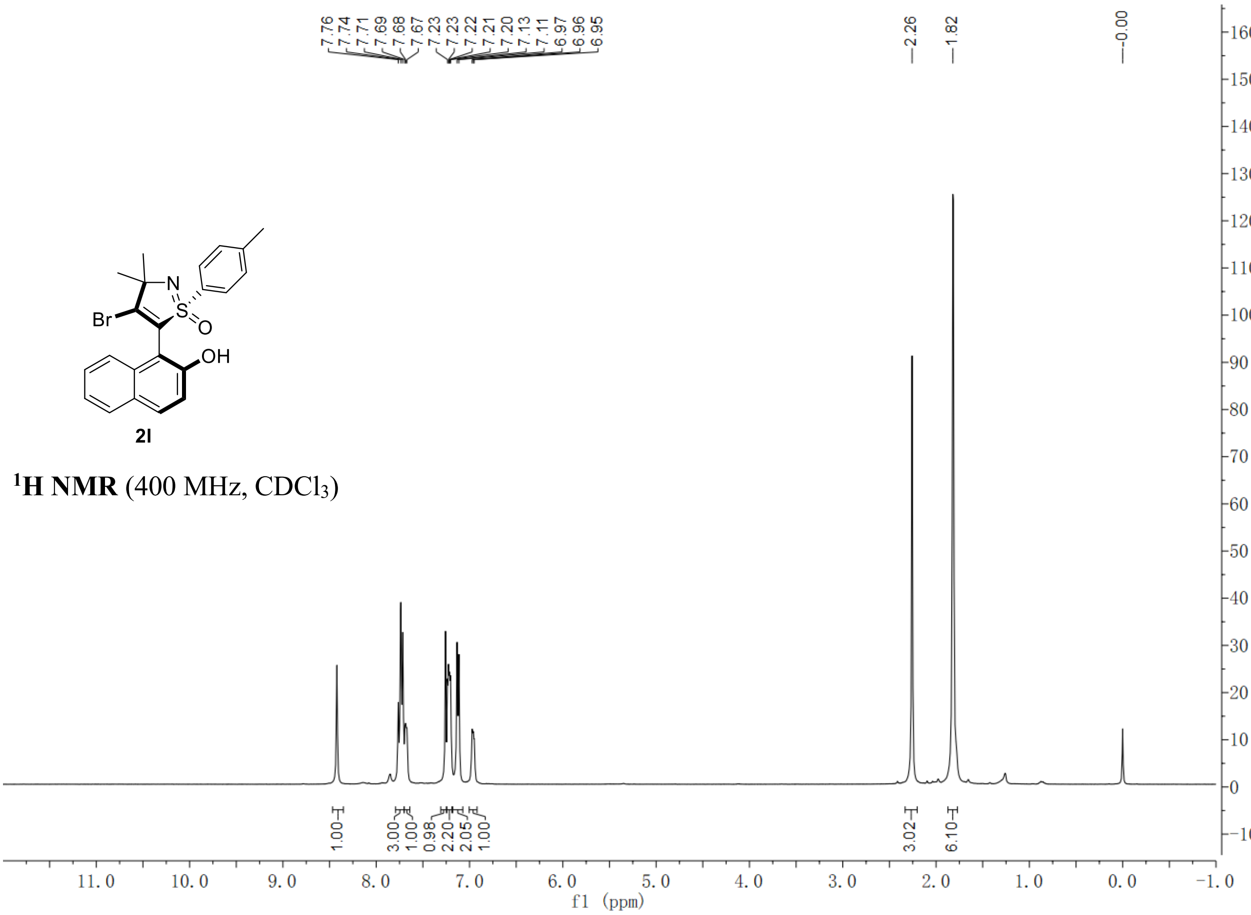

2l

^{1}**H NMR** (400 MHz, $CDCl_3$)

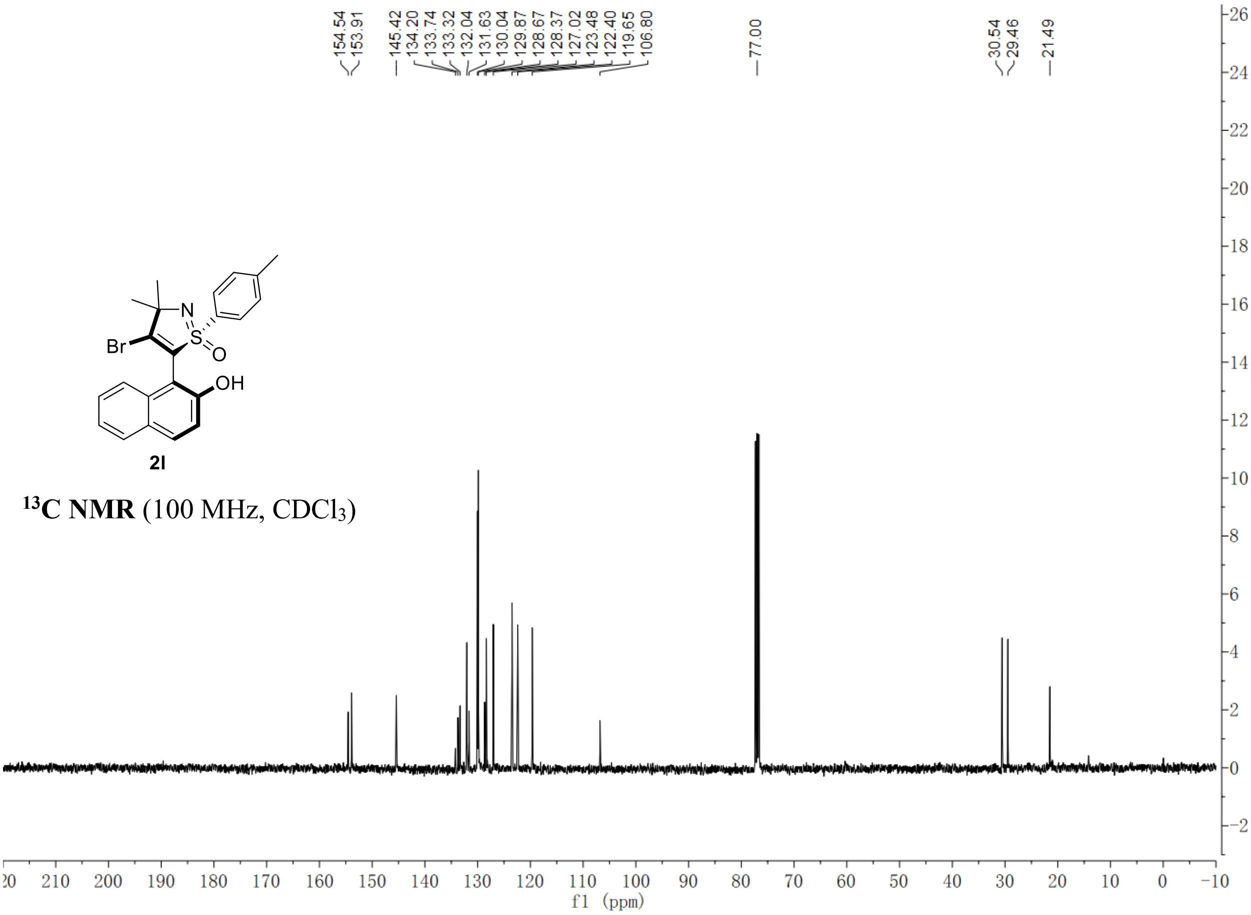

2l

^{13}**C NMR** (100 MHz, $CDCl_3$)

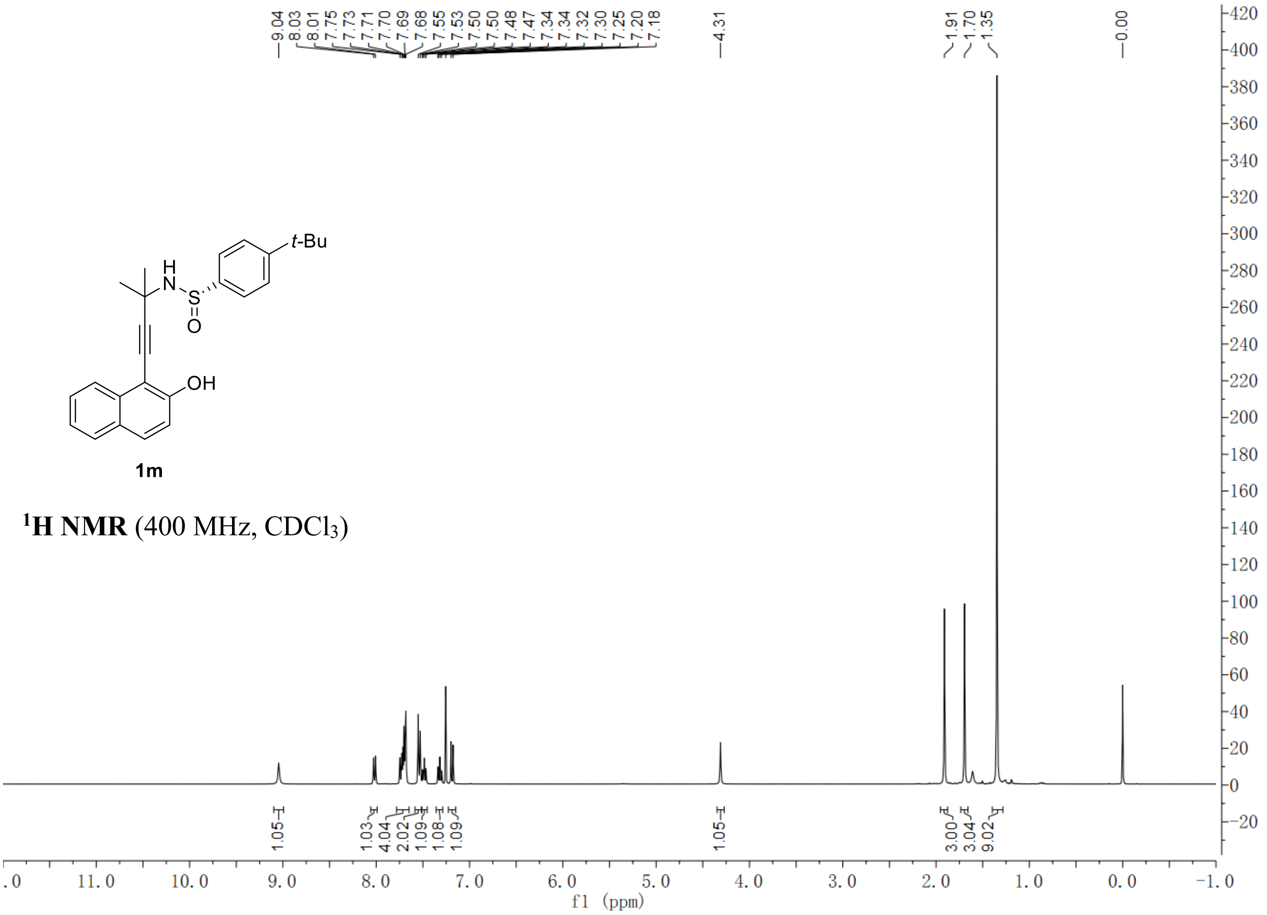
t-Bu
H
N
S
O
OH
1m
1H NMR (400 MHz, CDCl3)
9.04
8.03
8.01
7.75
7.73
7.71
7.70
7.69
7.68
7.55
7.53
7.50
7.50
7.48
7.47
7.34
7.34
7.32
7.30
7.25
7.20
7.18
4.31
1.91
1.70
1.35
0.00
1.05
1.03
4.04
2.02
1.09
1.08
1.09
1.05
3.00
3.04
9.02
f1 (ppm)

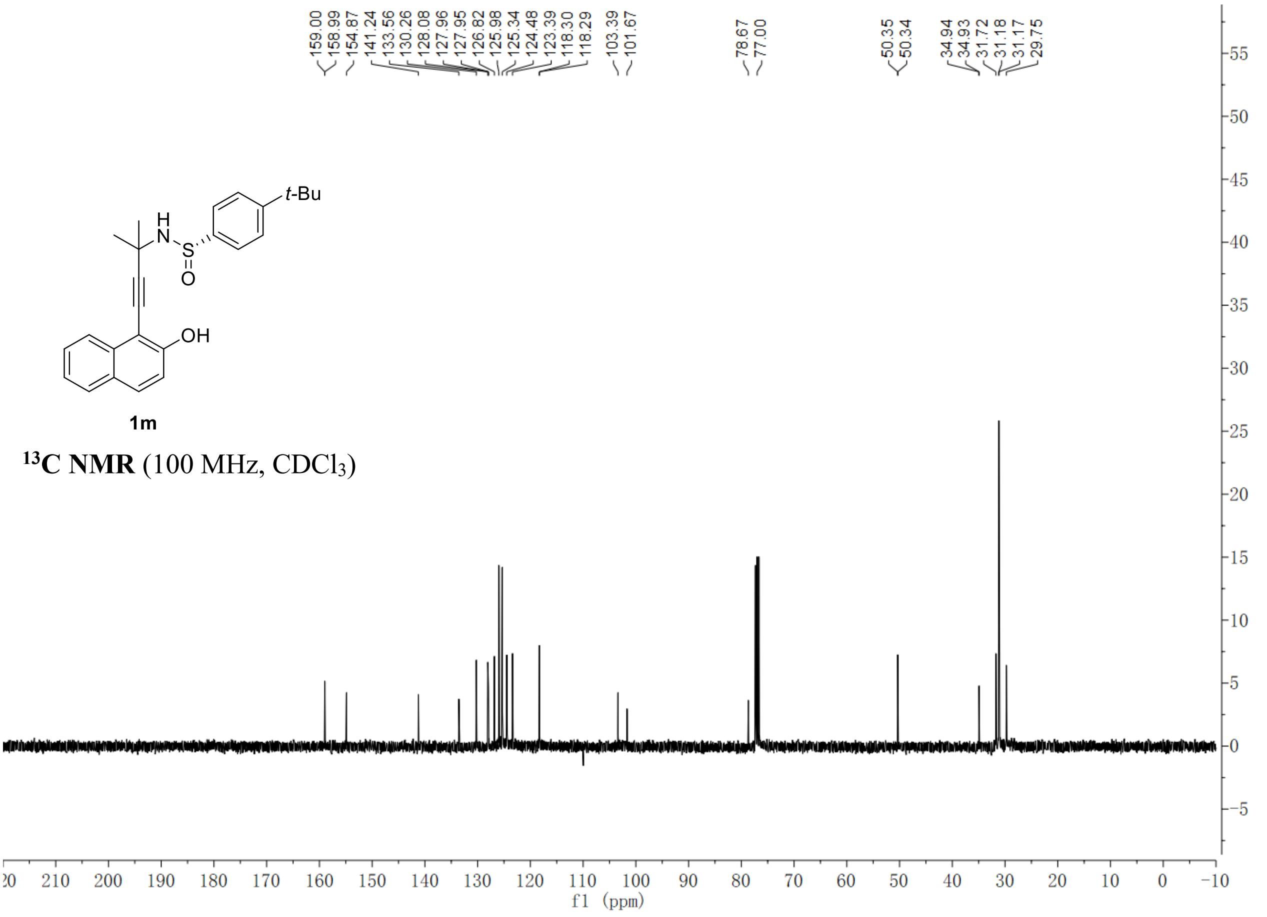
t-Bu
H
N
S
O
OH
1m
13C NMR (100 MHz, CDCl3)
159.00
158.99
154.87
141.24
133.56
130.26
128.08
127.96
127.95
126.82
125.98
125.34
124.48
123.39
118.30
118.29
103.39
101.67
78.67
77.00
50.35
50.34
34.94
34.93
31.72
31.18
31.17
29.75
f1 (ppm)

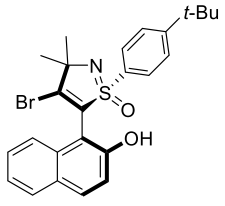

2m

^{1}H NMR (400 MHz, $CDCl_3$)

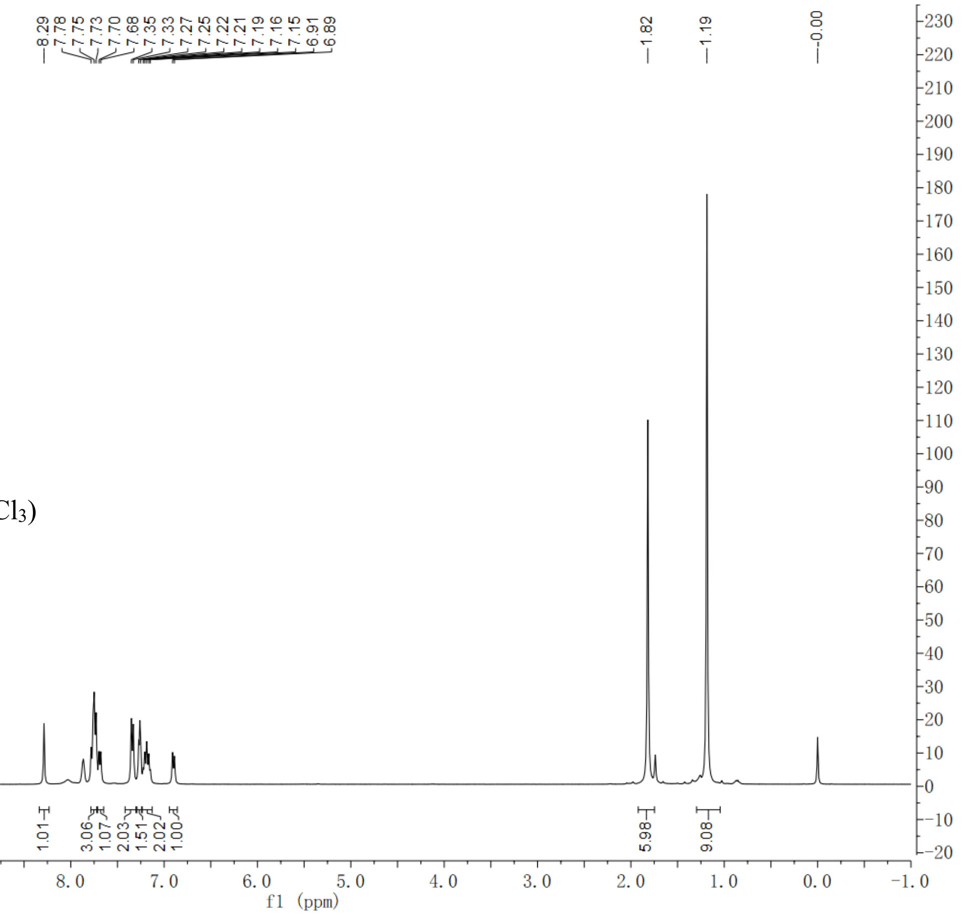

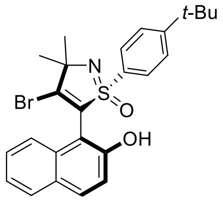

2m

^{13}C NMR (100 MHz, $CDCl_3$)

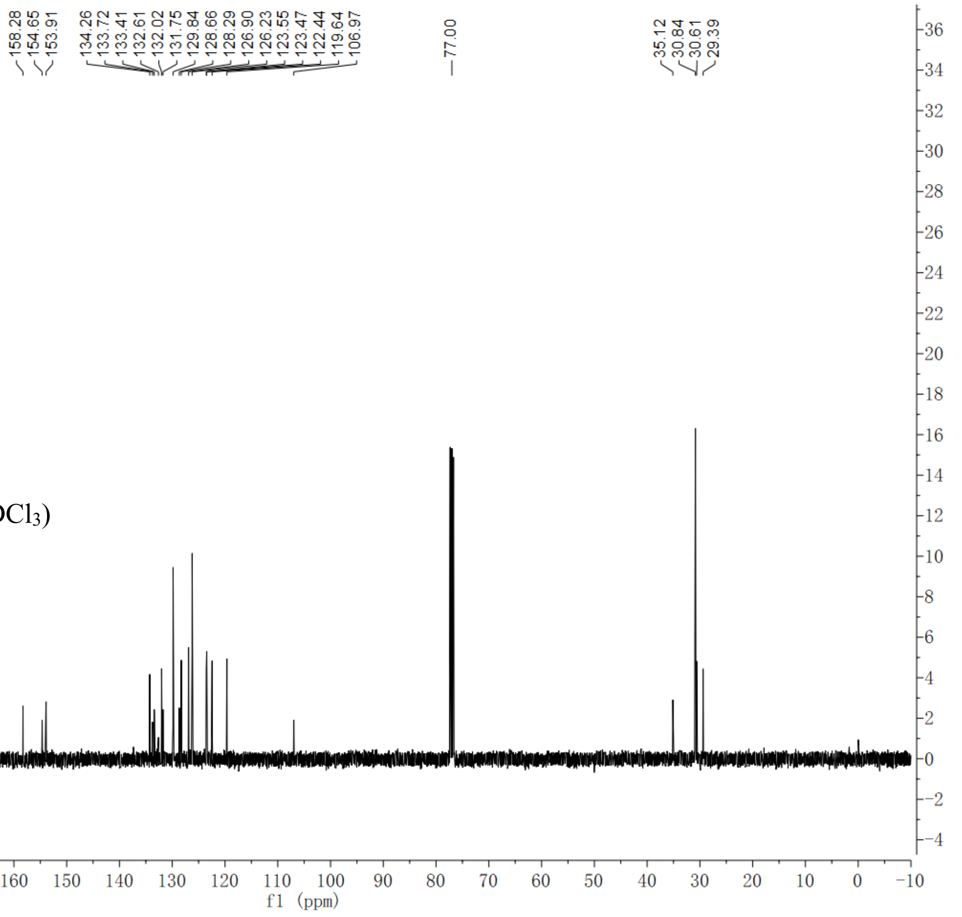

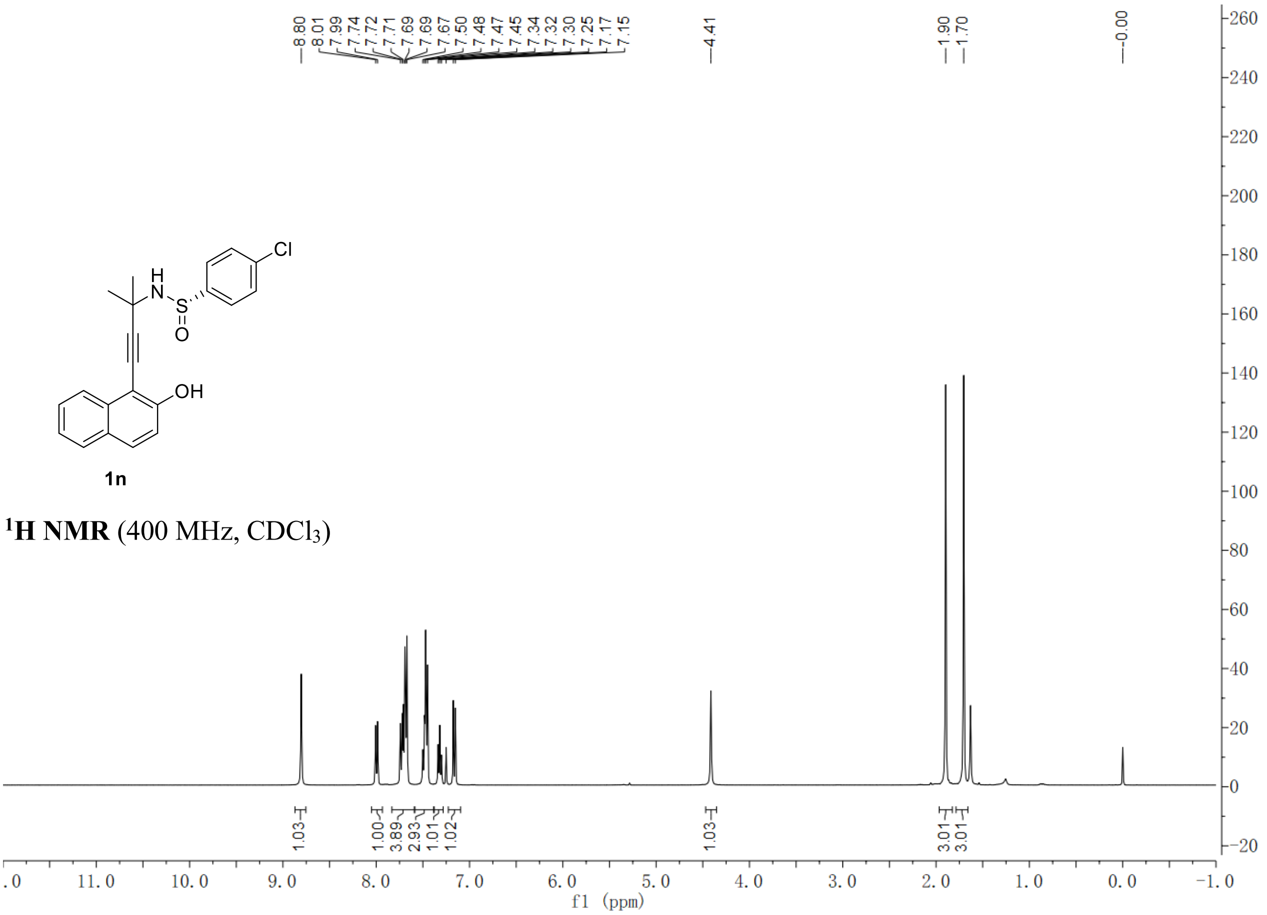

1n

^{1}H NMR (400 MHz, $CDCl_3$)

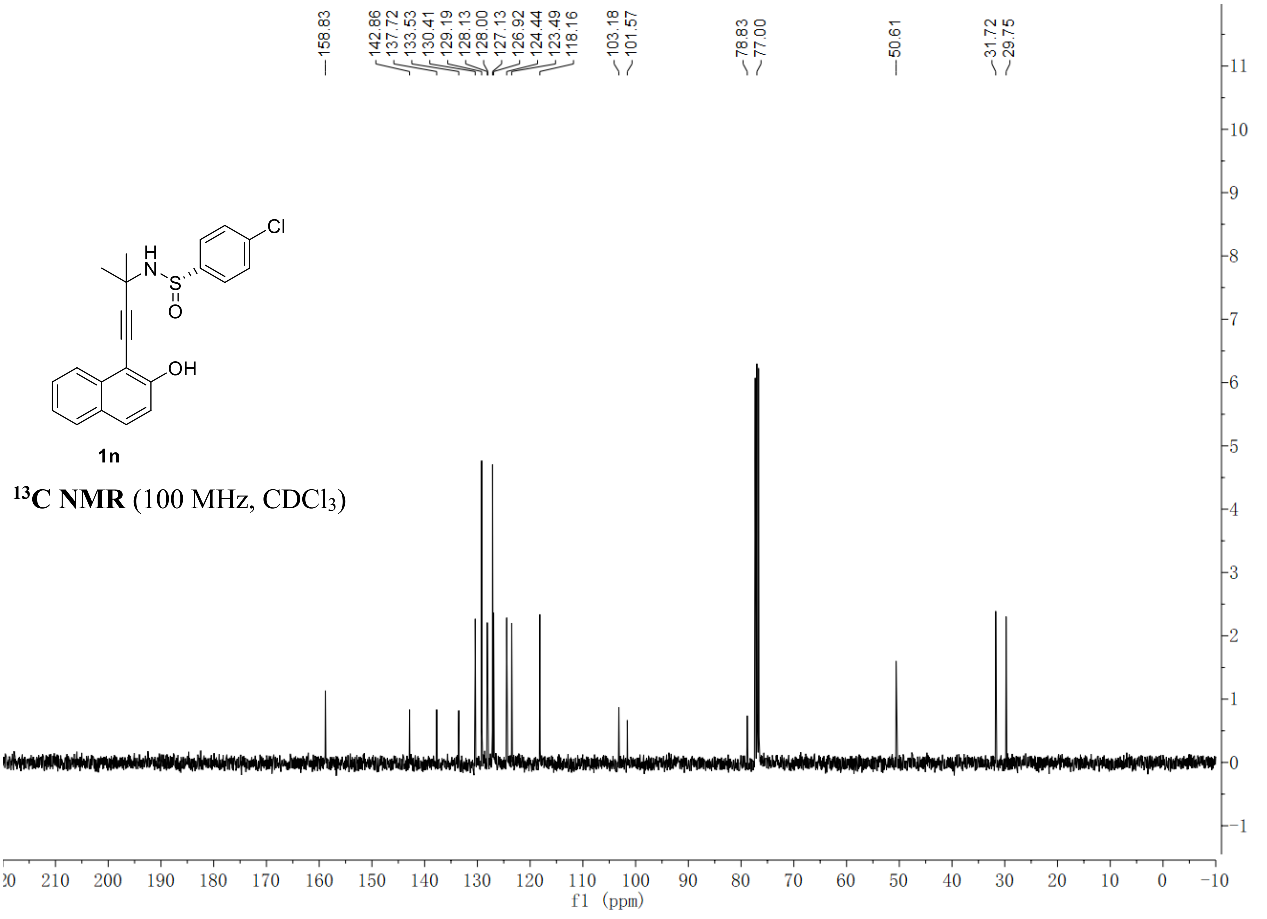

1n

^{13}C NMR (100 MHz, $CDCl_3$)

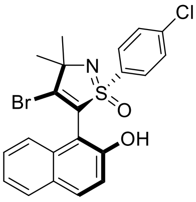

2n

[1]**H NMR** (400 MHz, $CDCl_3$)

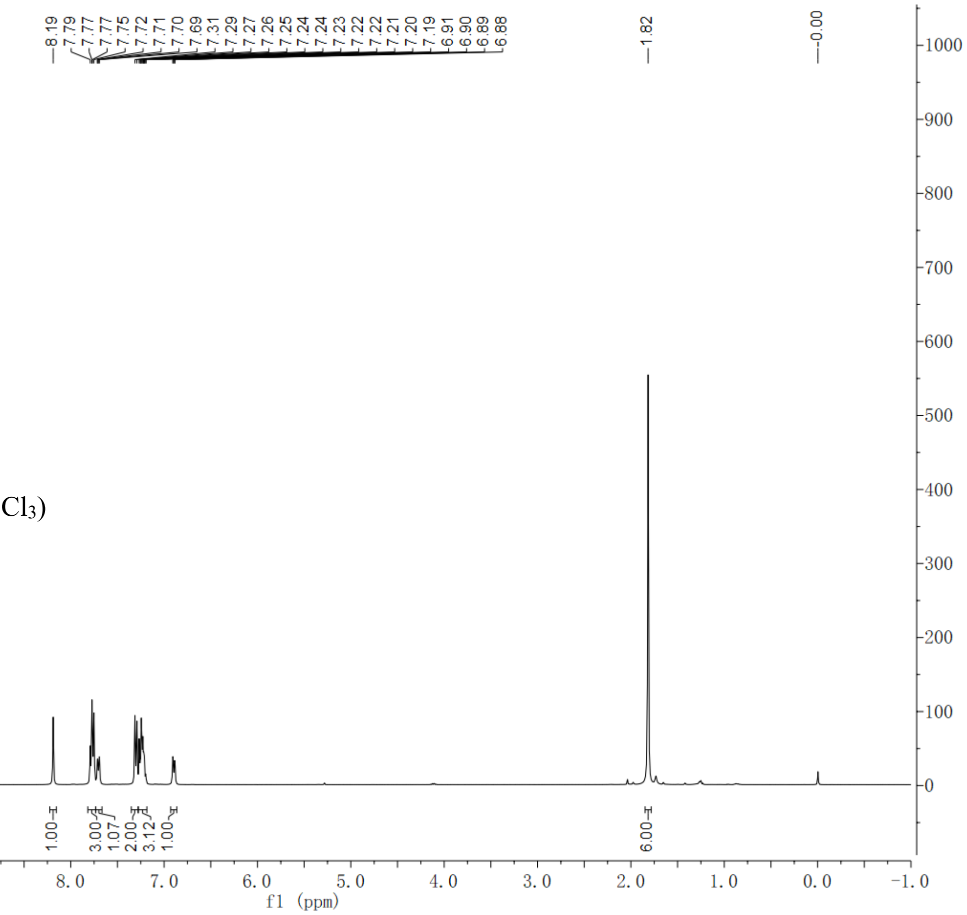

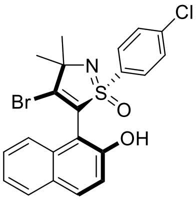

2n

[13]**C NMR** (100 MHz, $CDCl_3$)

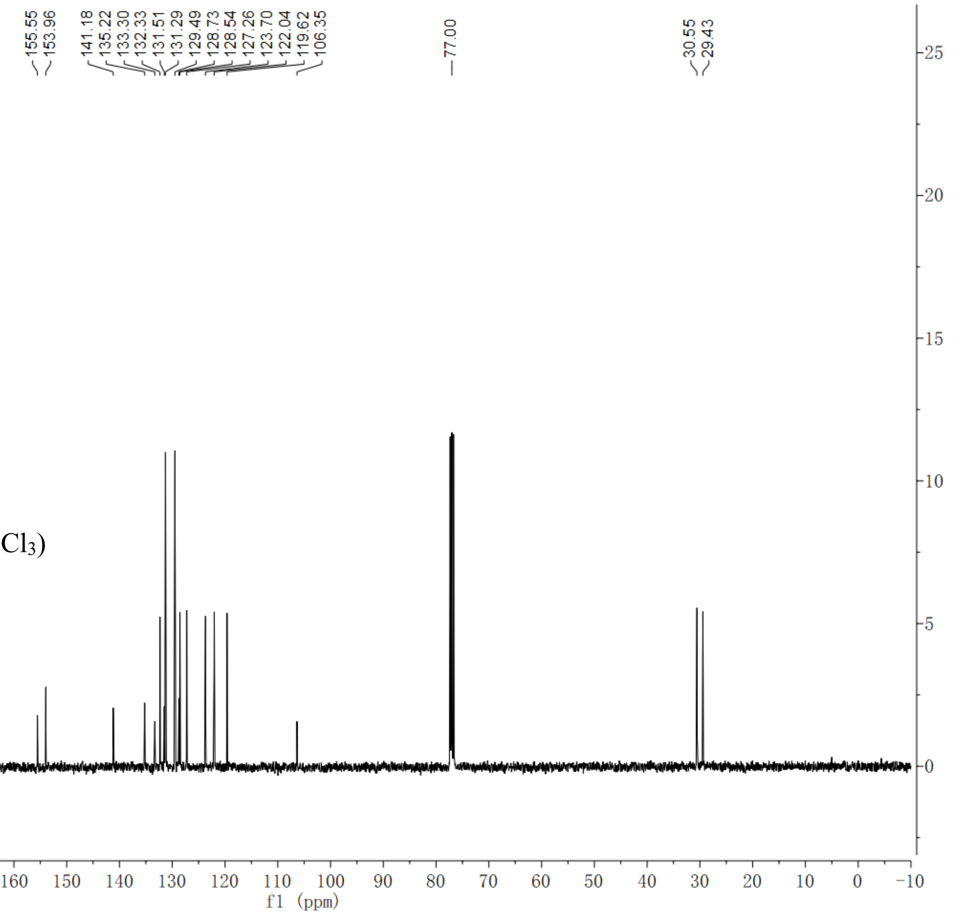

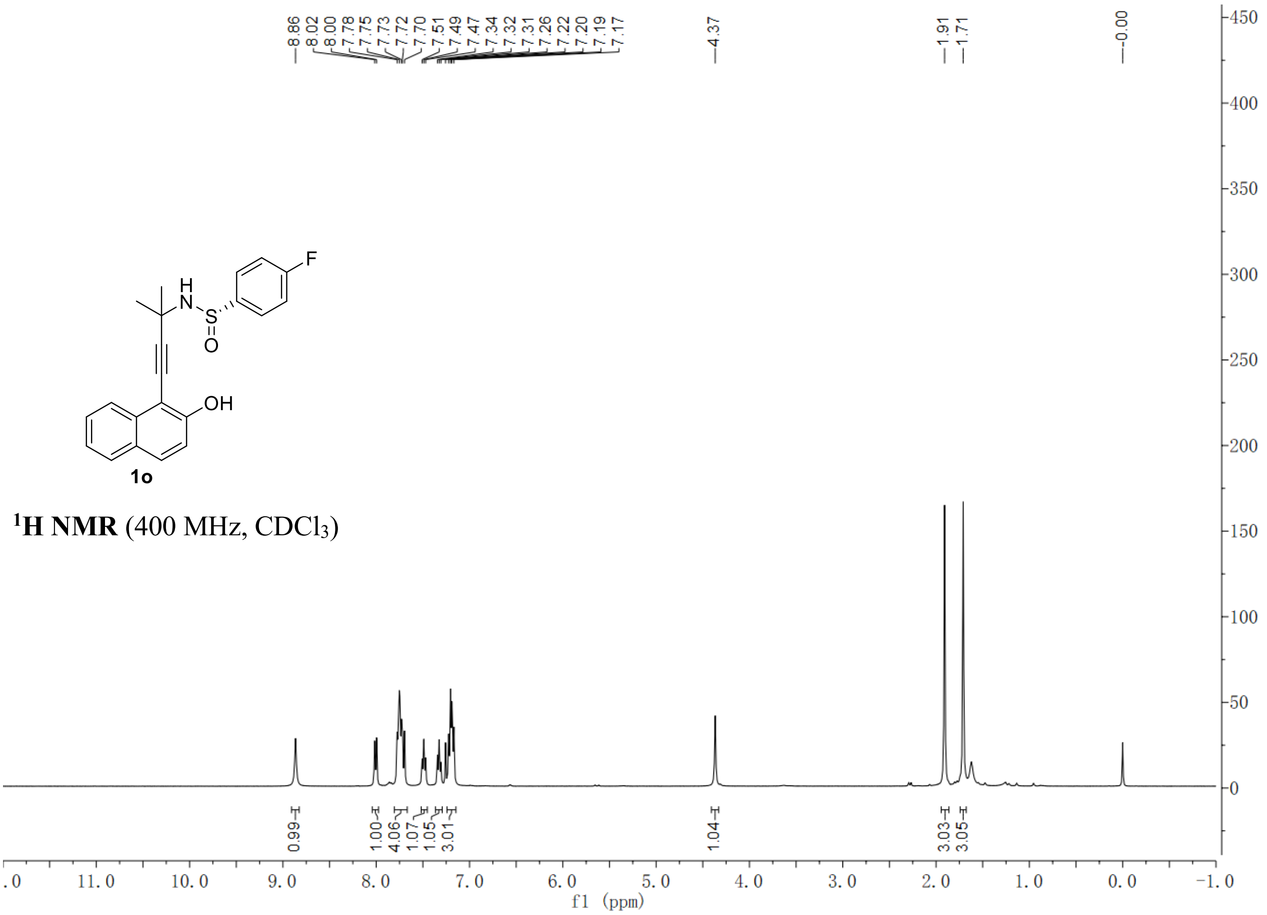

^{1}H NMR (400 MHz, $CDCl_3$)

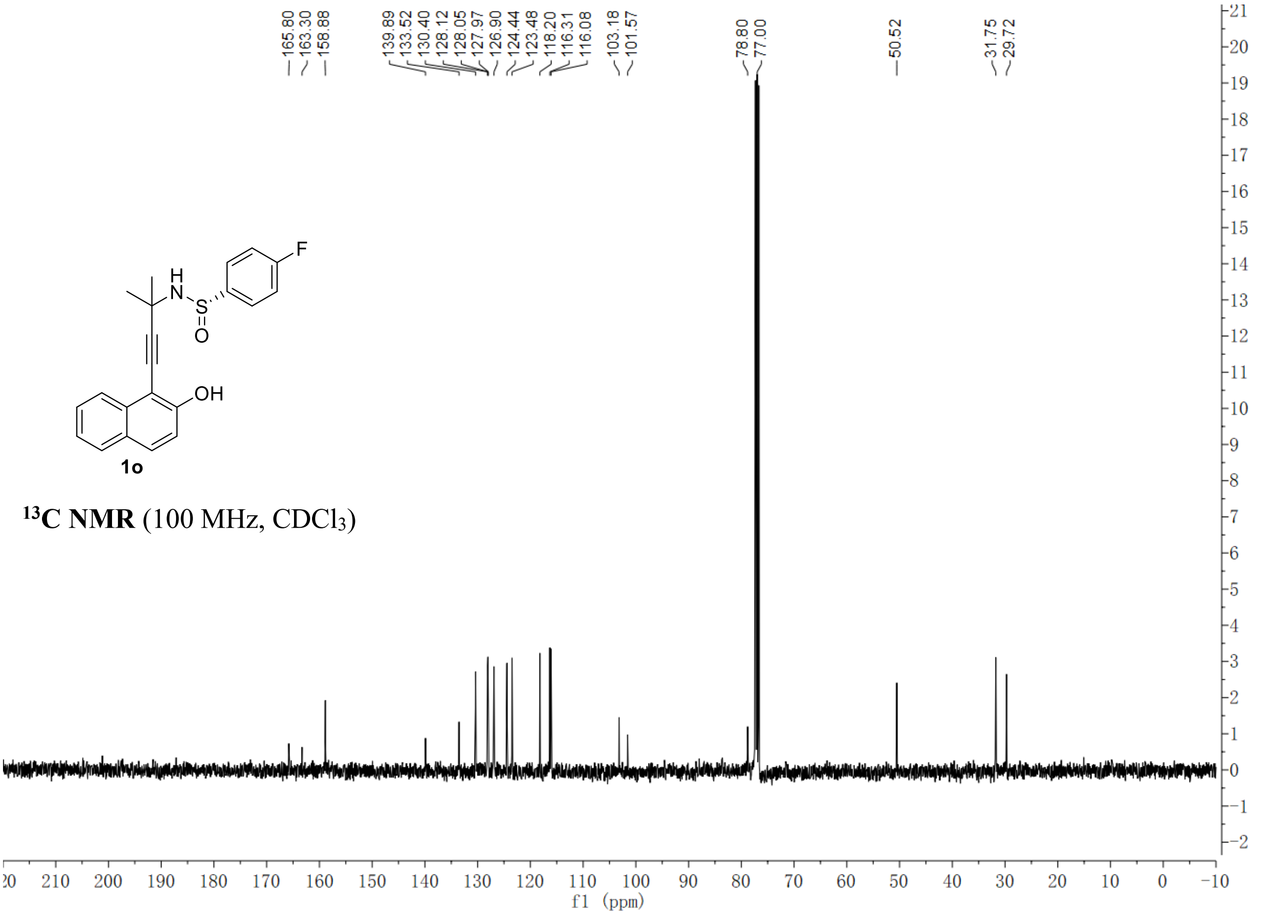

^{13}C NMR (100 MHz, $CDCl_3$)

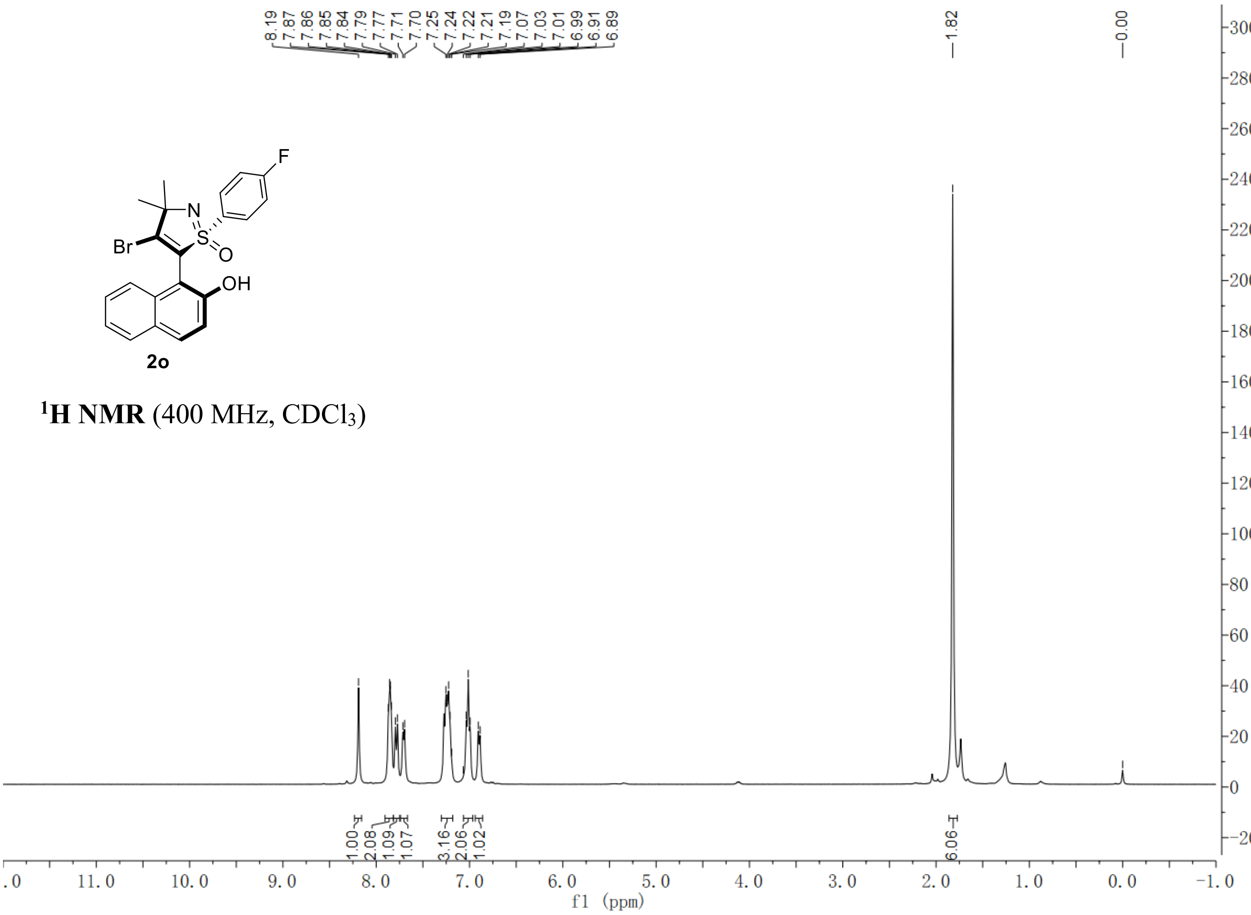
8.19 7.87 7.86 7.85 7.84 7.79 7.77 7.71 7.70 7.25 7.24 7.22 7.21 7.19 7.07 7.03 7.01 6.99 6.91 6.89
1.82
0.00
F
N
S
O
Br
OH
2o
^{1}H NMR (400 MHz, CDCl$_3$)
1.00 2.08 1.09 1.07 3.16 2.06 1.02
6.06
f1 (ppm)

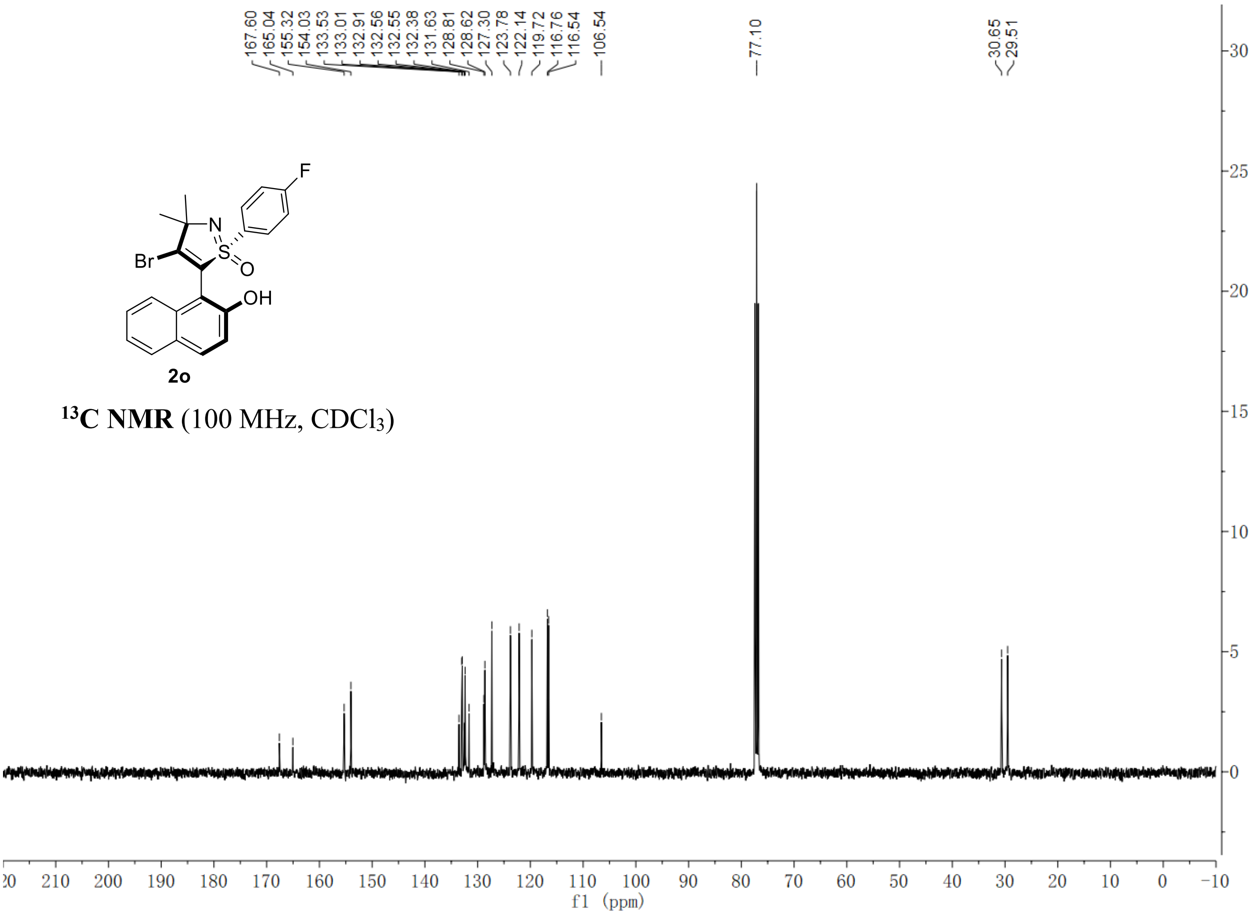
167.60 165.04 155.32 154.03 133.53 133.01 132.91 132.56 132.55 132.38 131.63 128.81 128.62 127.30 123.78 122.14 119.72 116.76 116.54 106.54
77.10
30.65 29.51
F
N
S
O
Br
OH
2o
^{13}C NMR (100 MHz, CDCl$_3$)
f1 (ppm)

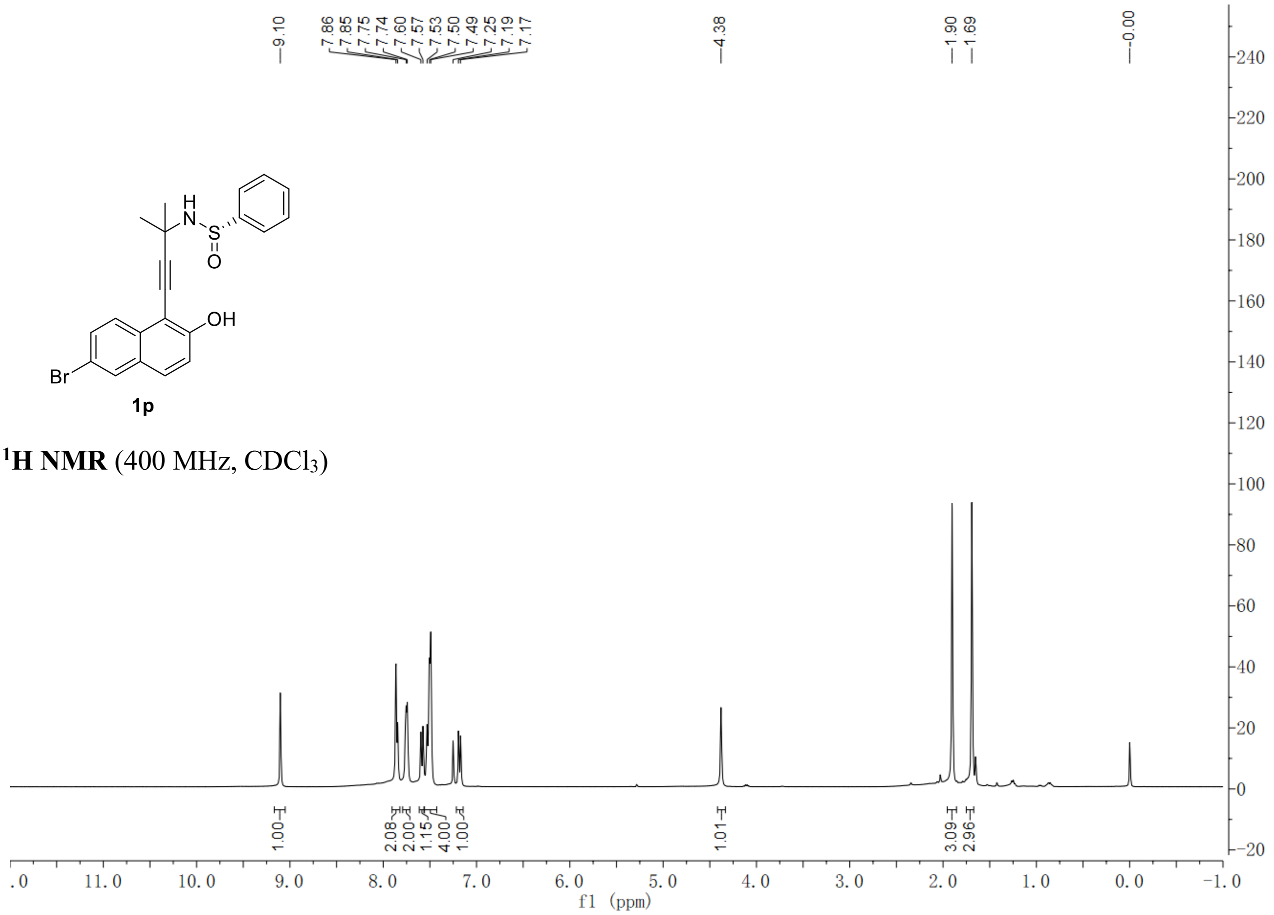

1p

^{1}H NMR (400 MHz, $CDCl_3$)

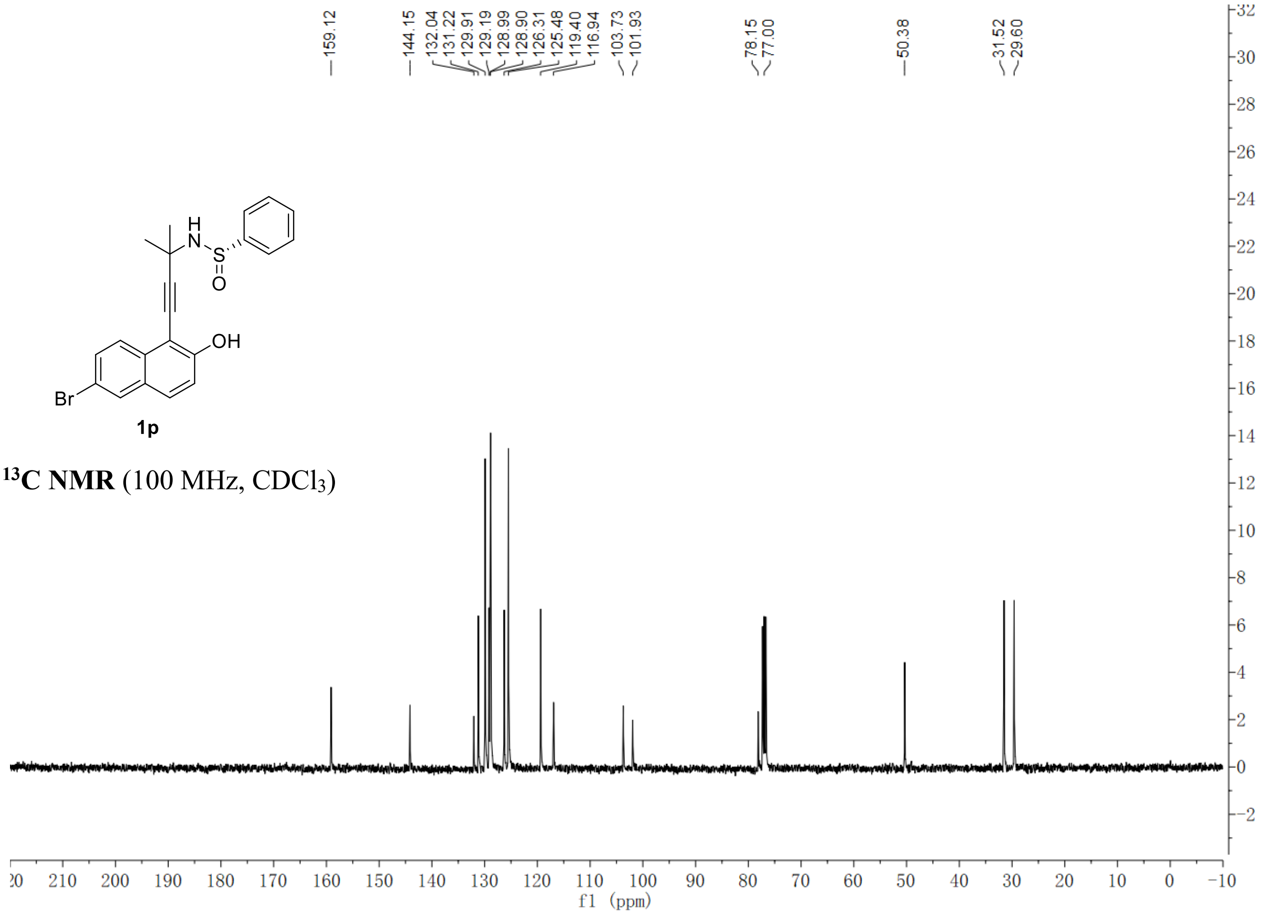

1p

^{13}C NMR (100 MHz, $CDCl_3$)

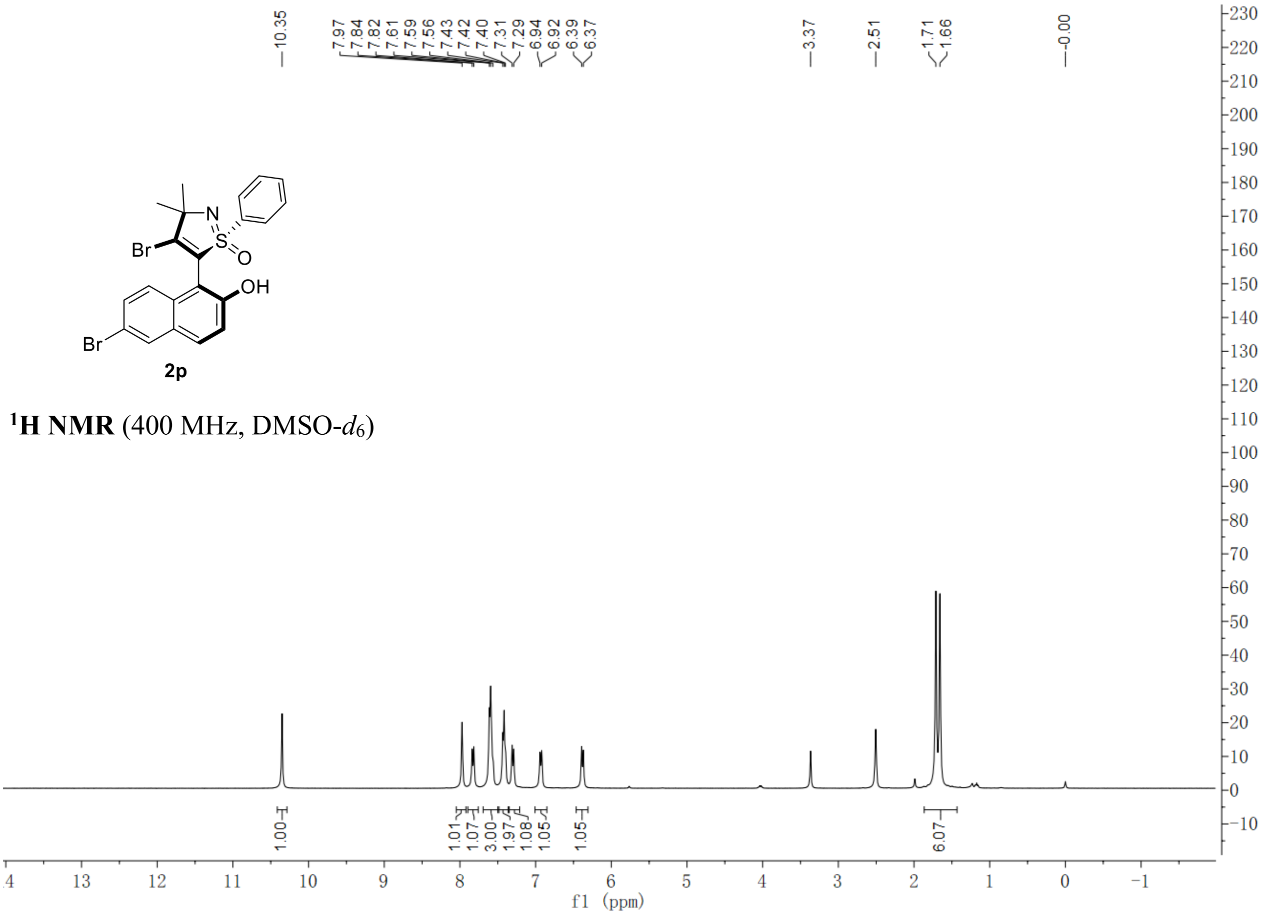

2p

^{1}H NMR (400 MHz, DMSO-d_6)

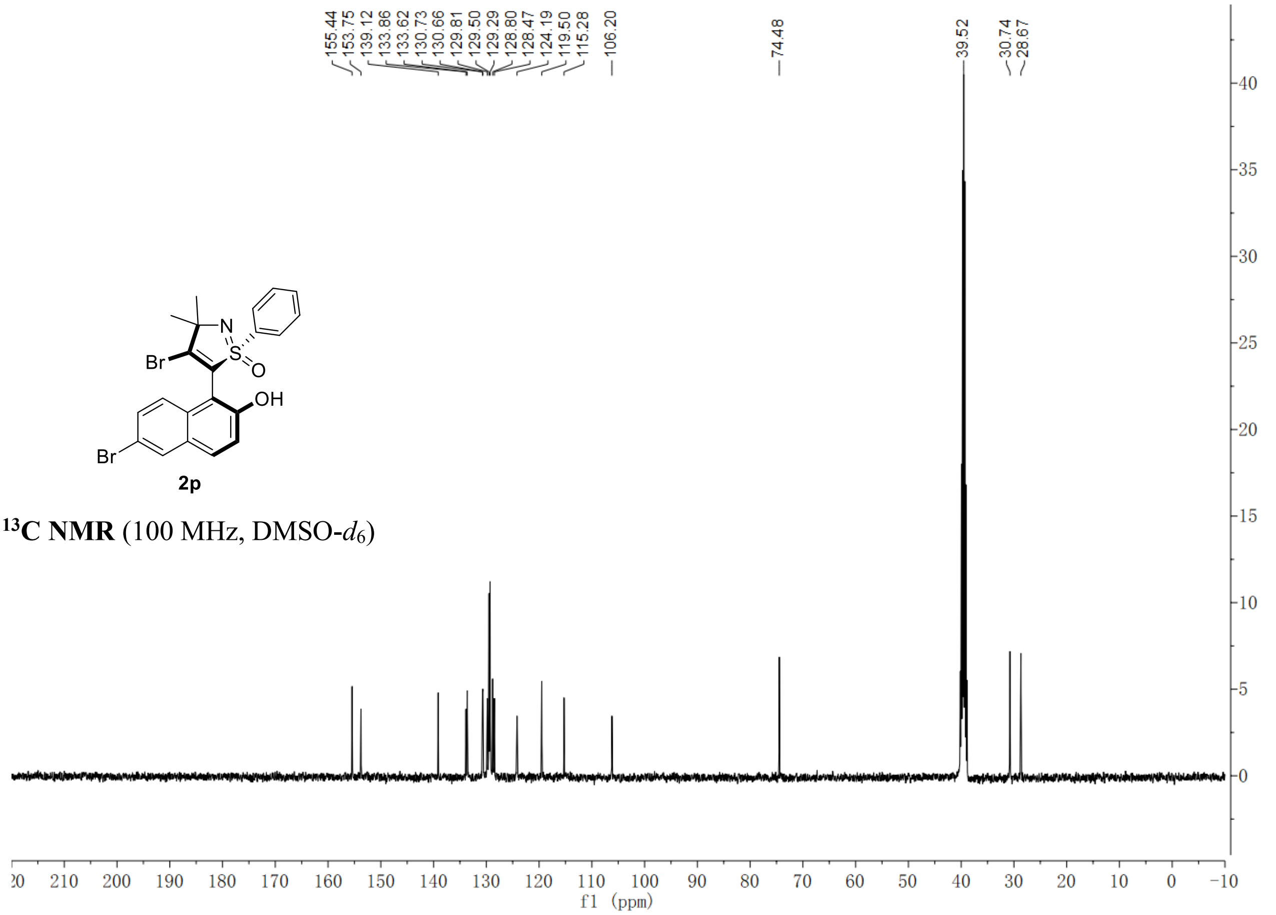

2p

^{13}C NMR (100 MHz, DMSO-d_6)

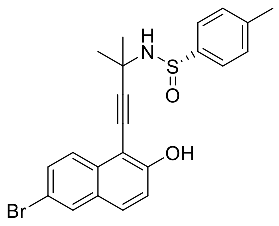

1q

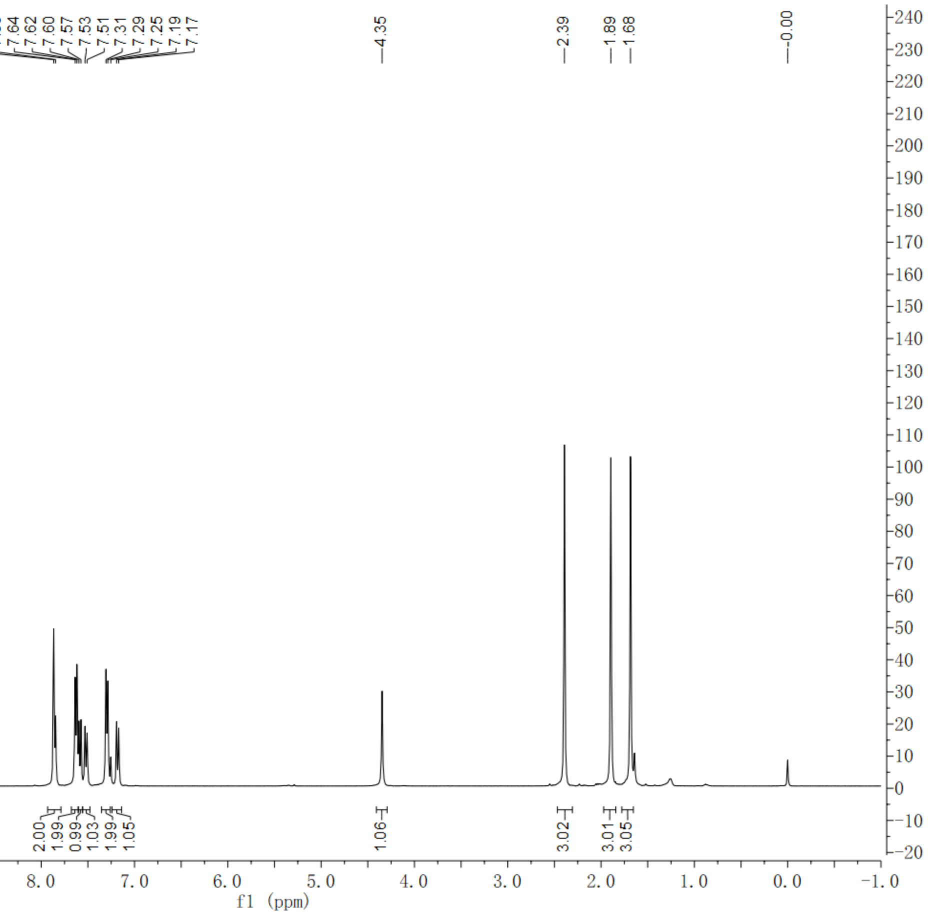

^{1}H NMR (400 MHz, $CDCl_3$)

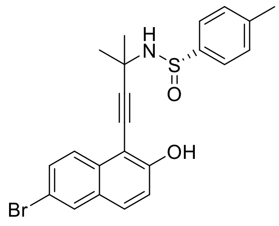

1q

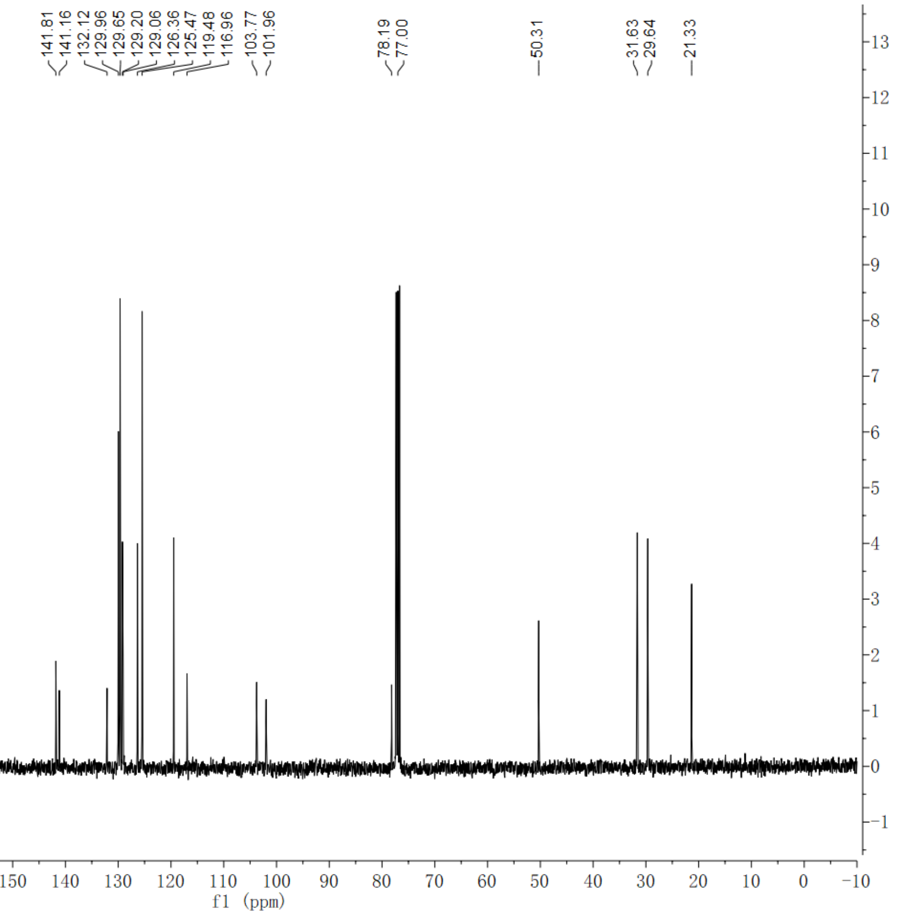

^{13}C NMR (100 MHz, $CDCl_3$)

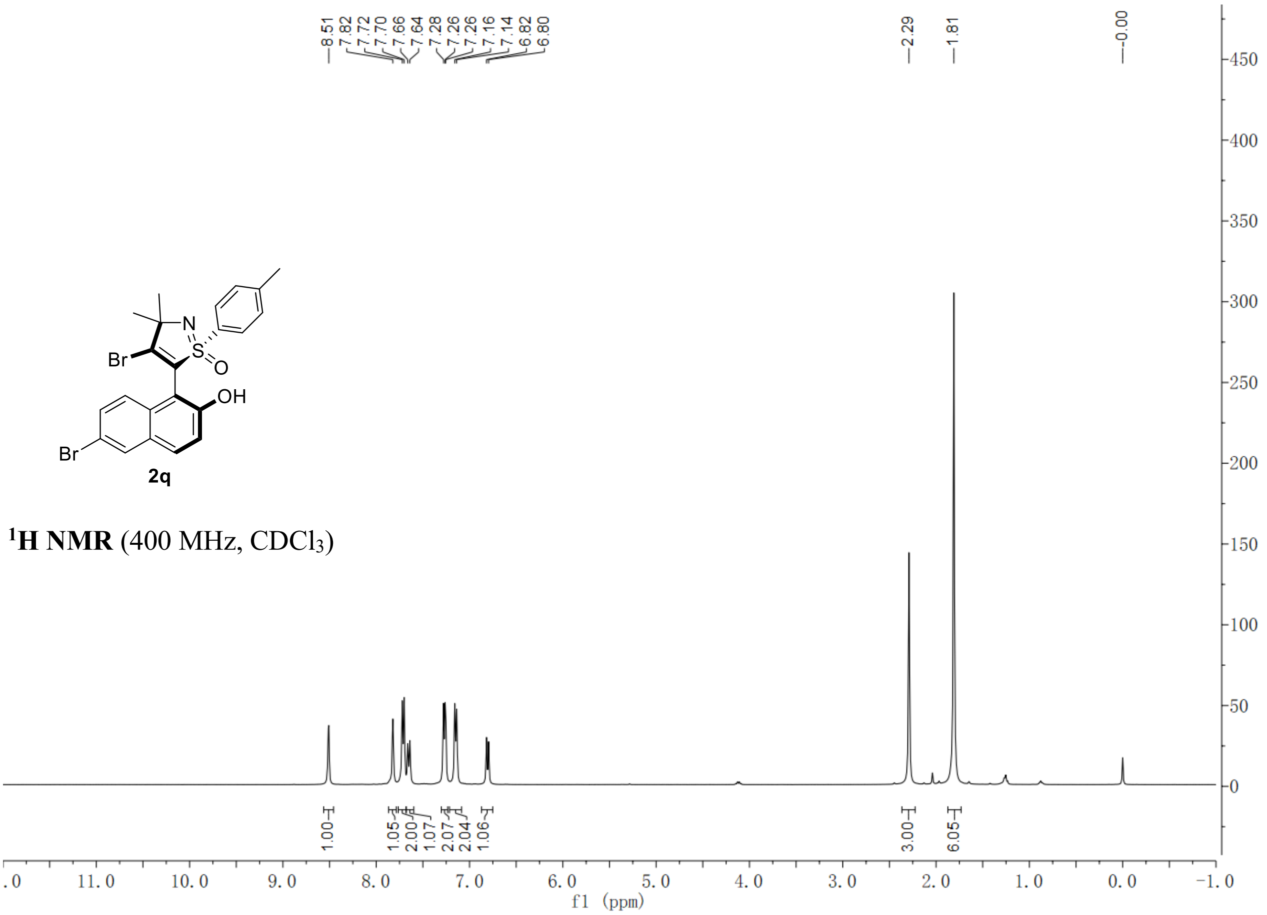

^{1}H NMR (400 MHz, $CDCl_3$)

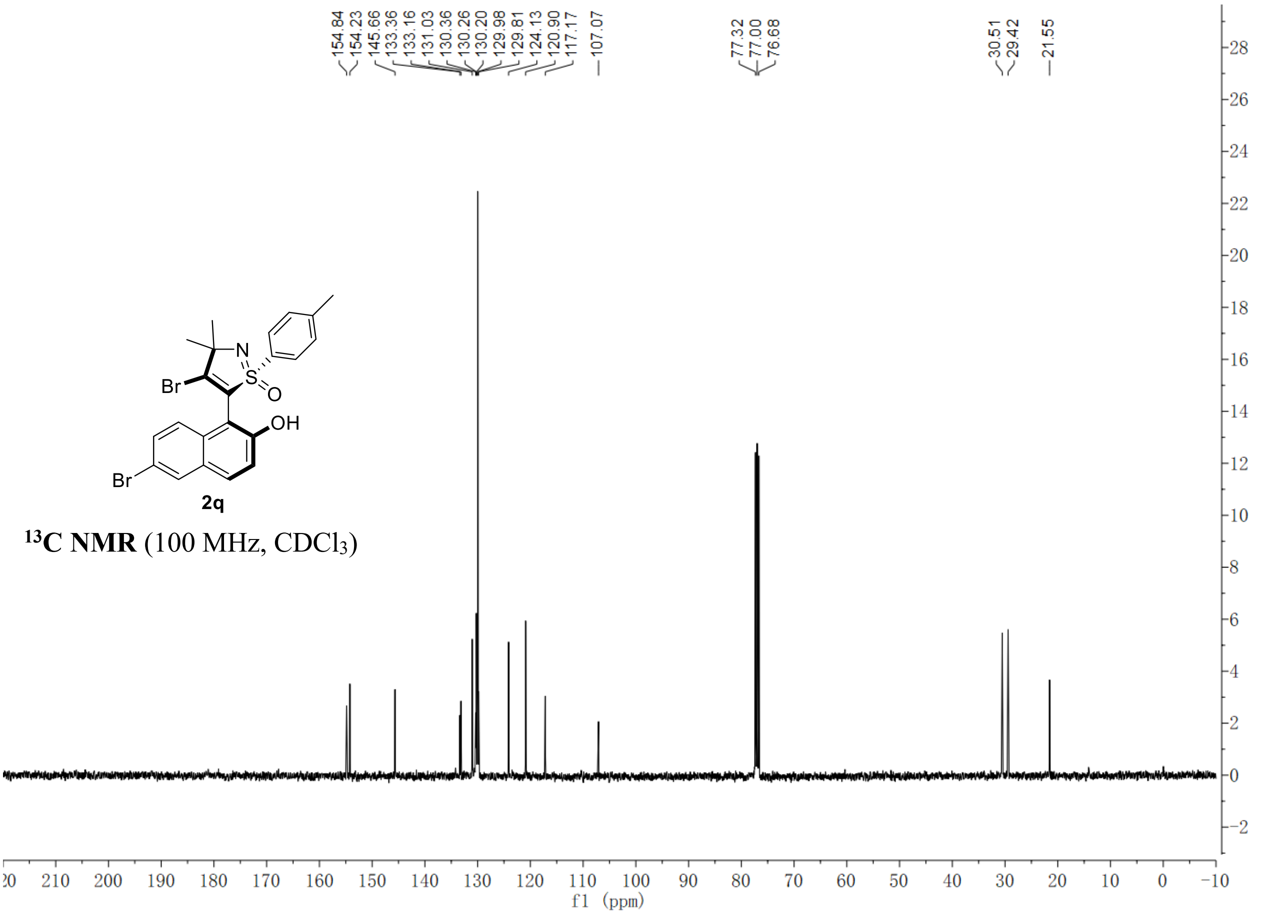

^{13}C NMR (100 MHz, $CDCl_3$)

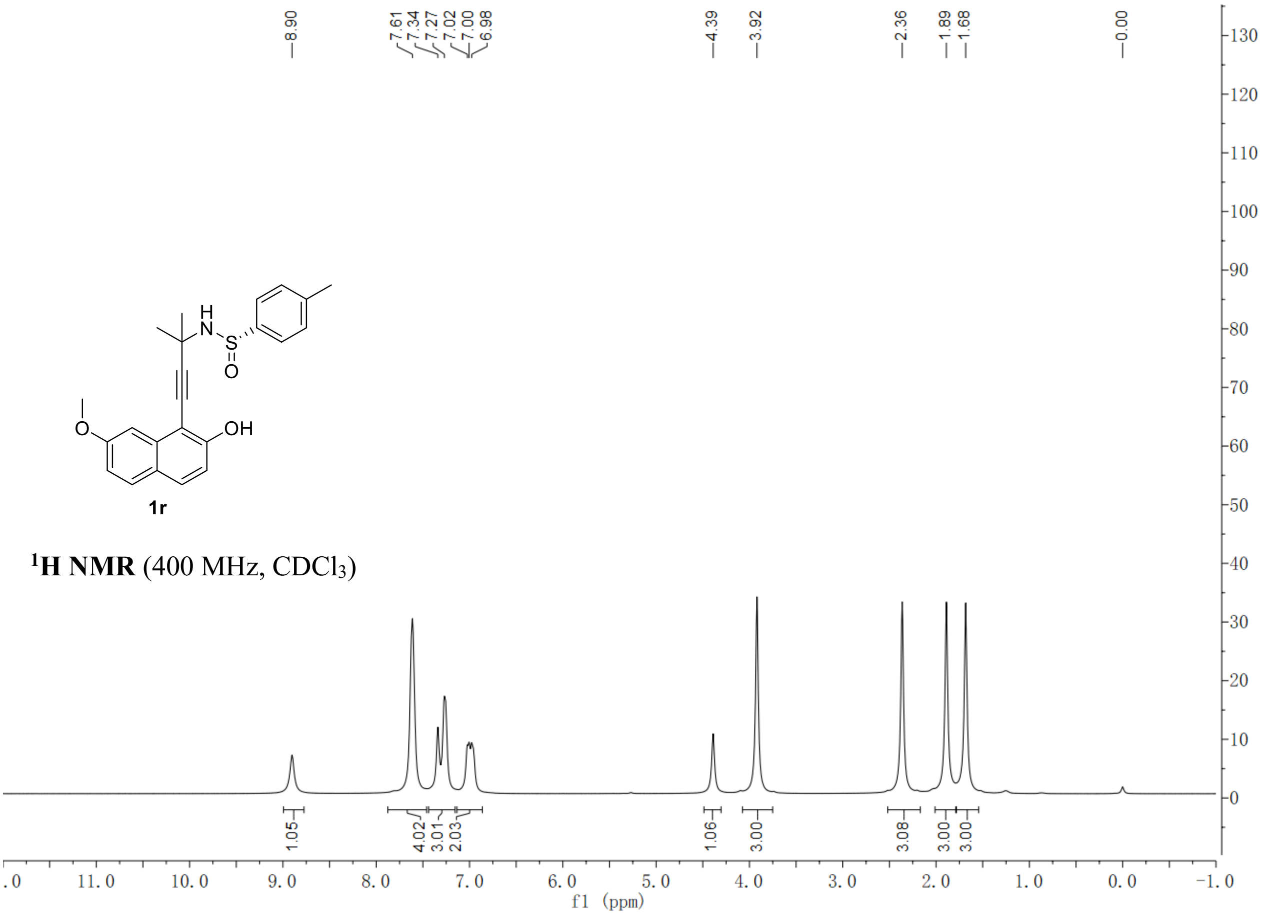

1r

^{1}H NMR (400 MHz, $CDCl_3$)

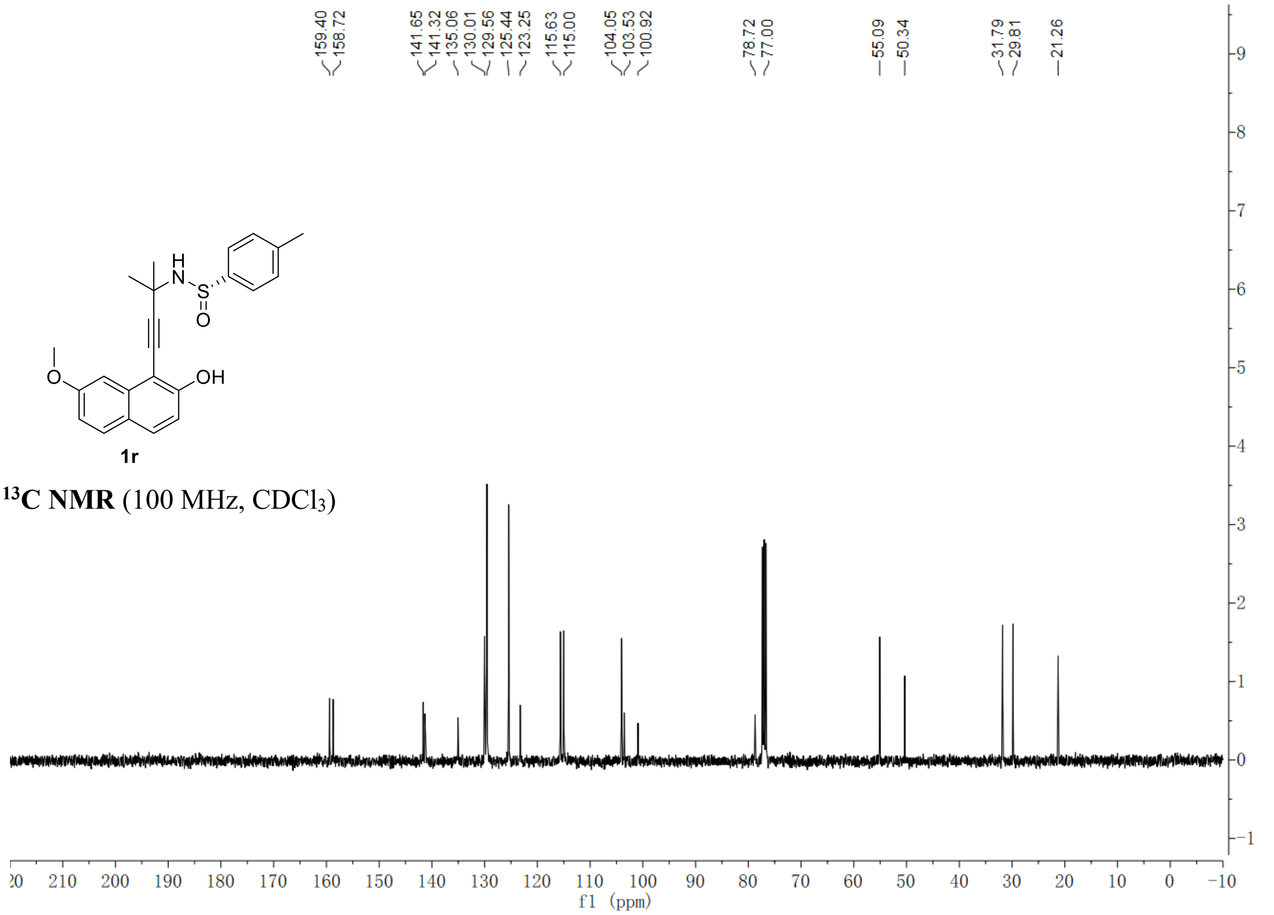

1r

^{13}C NMR (100 MHz, $CDCl_3$)

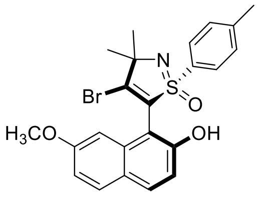

2r

^{1}H NMR (400 MHz, $CDCl_3$)

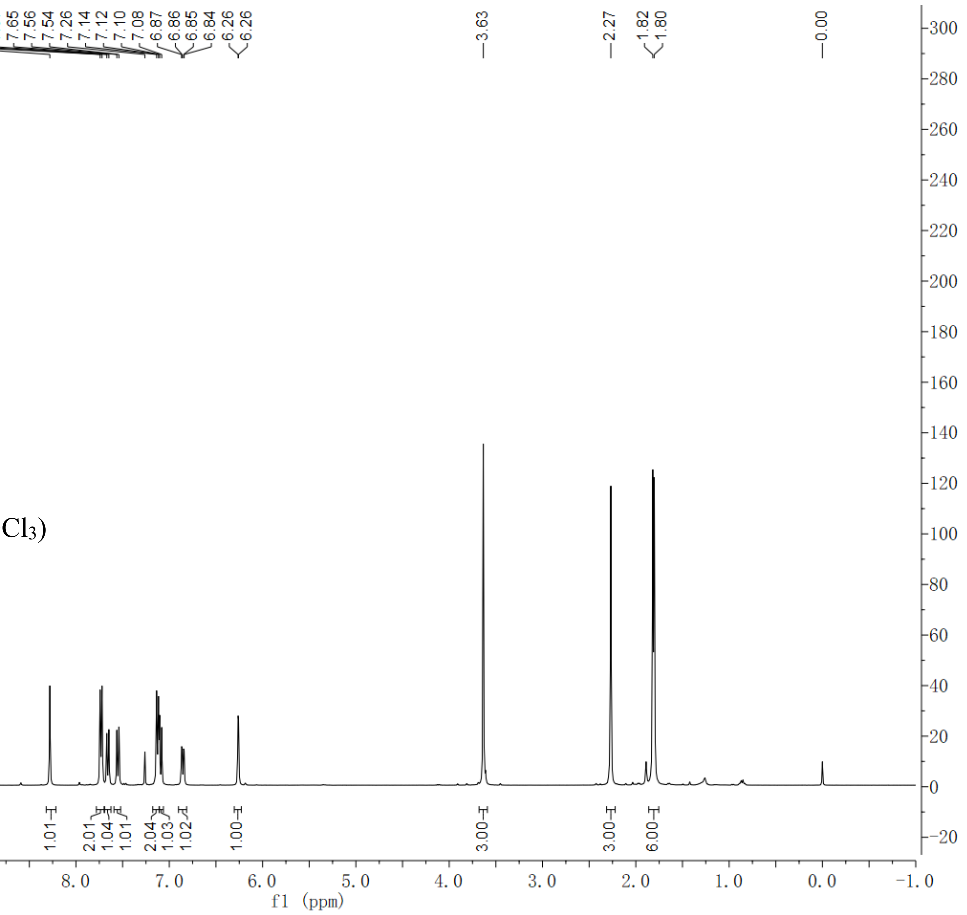

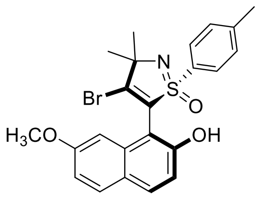

2r

^{13}C NMR (100 MHz, $CDCl_3$)

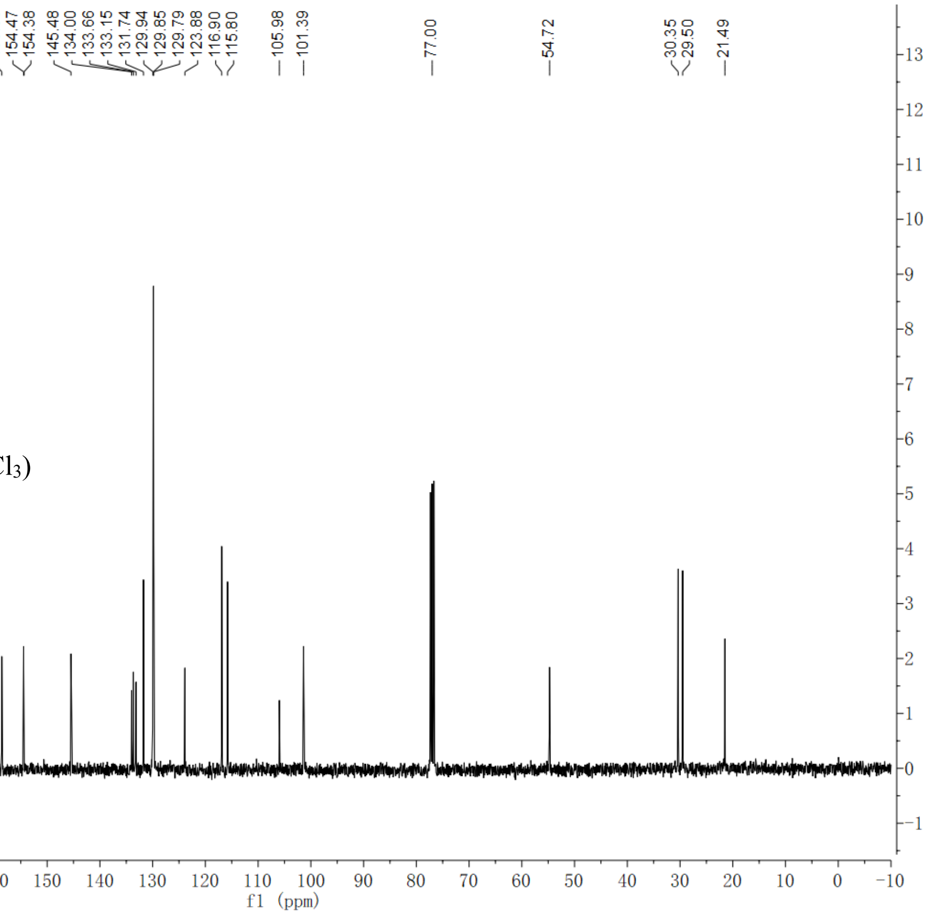

1s

^{1}H NMR (400 MHz, CDCl₃)

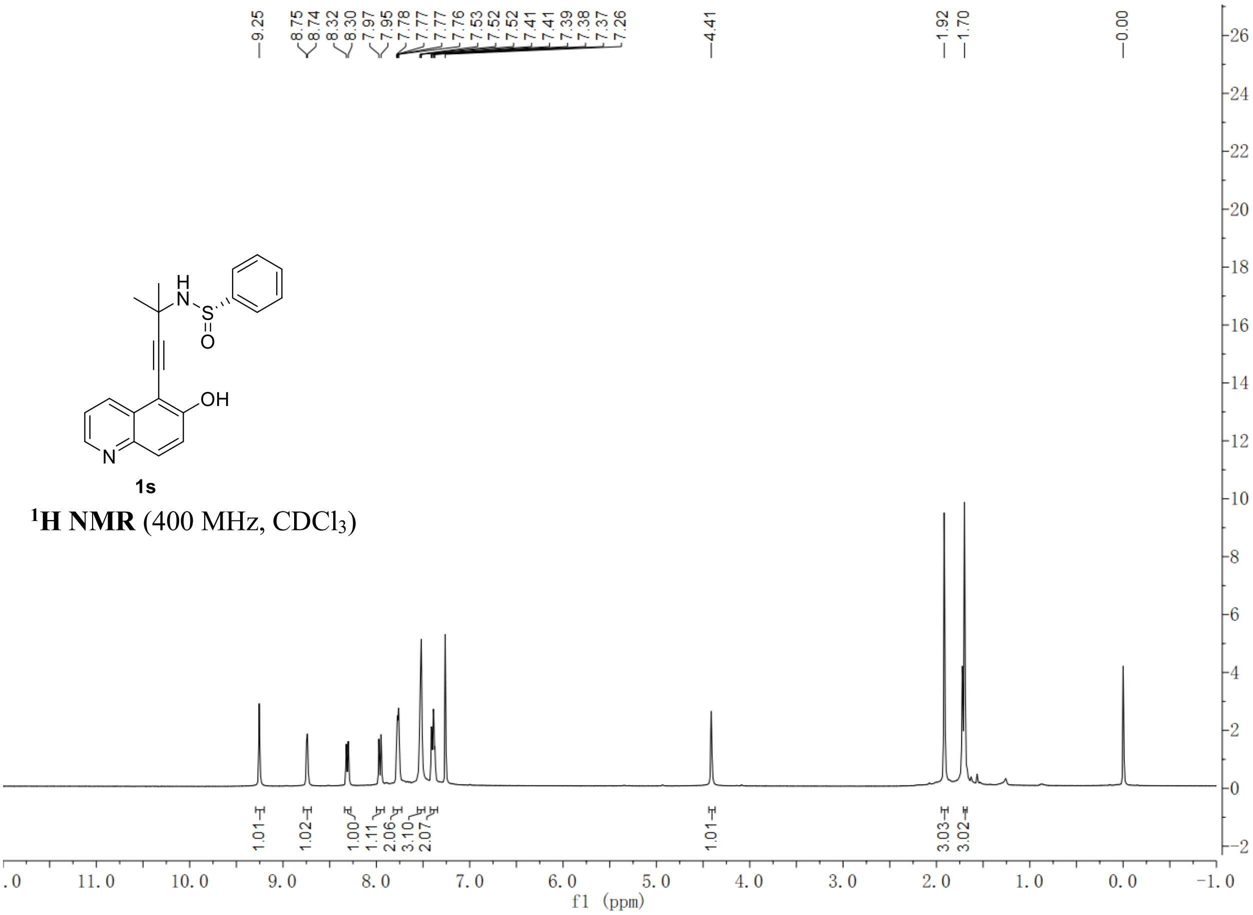

1s

^{13}C NMR (100 MHz, CDCl₃)

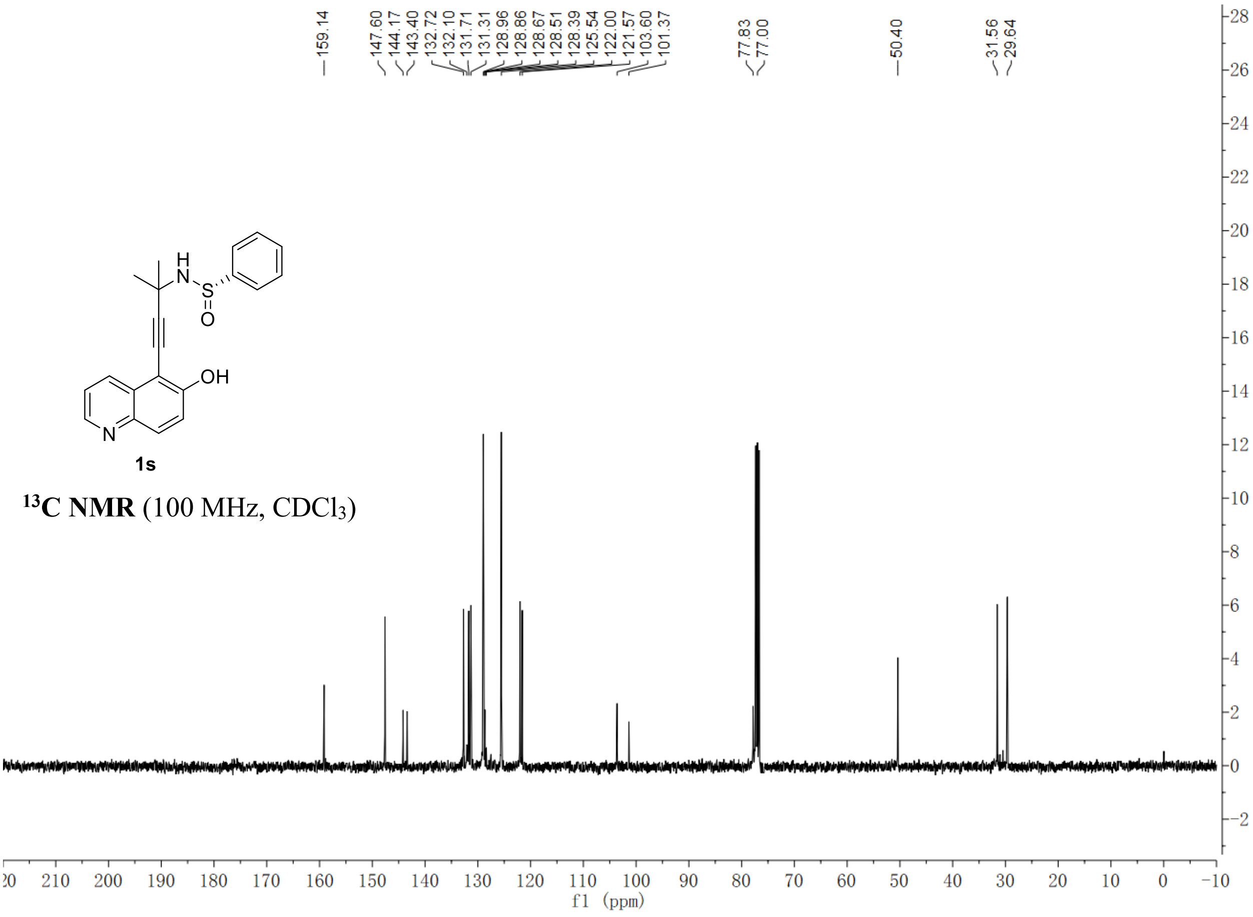

2s

^{1}H NMR (400 MHz, $CDCl_3$)

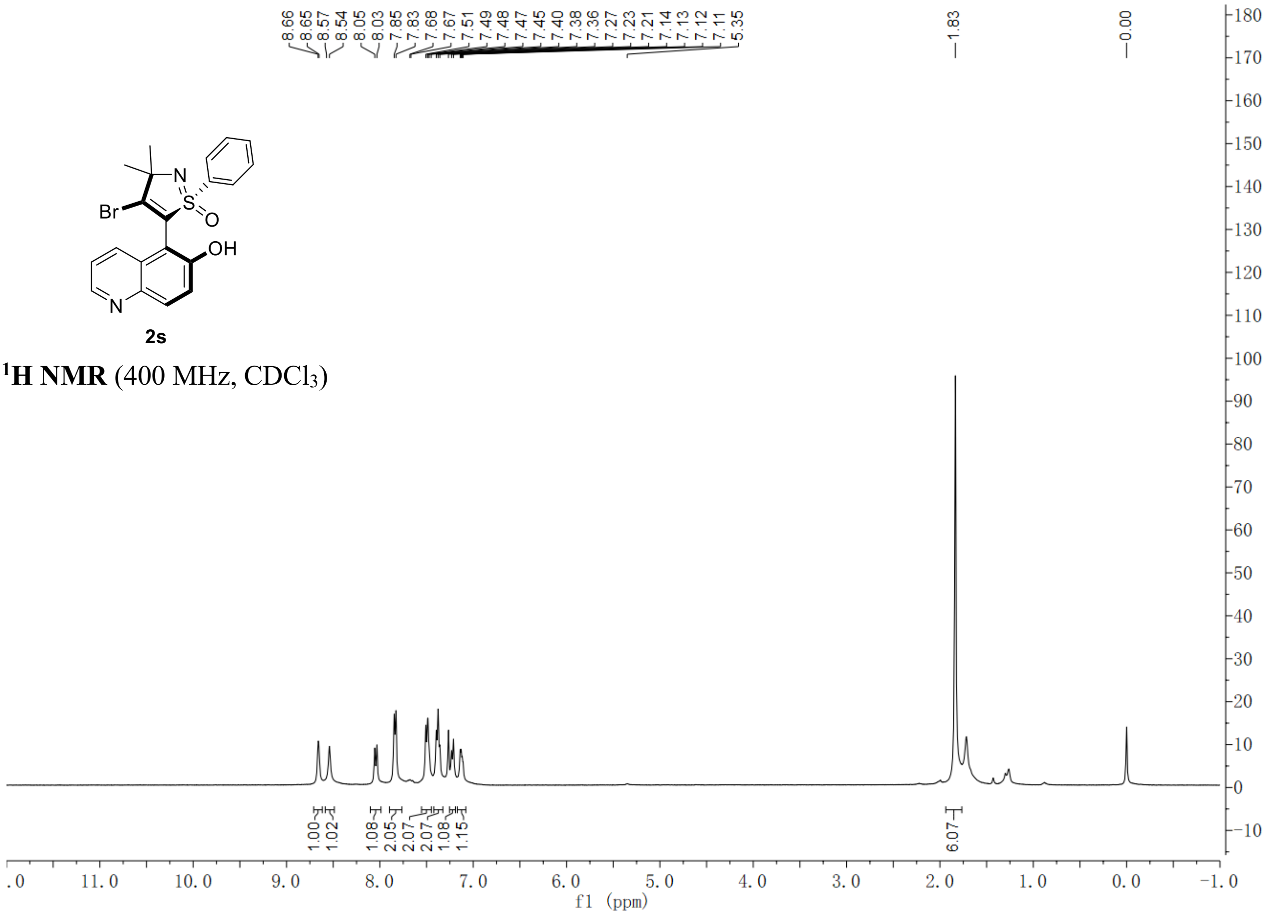

2s

^{13}C NMR (100 MHz, $CDCl_3$)

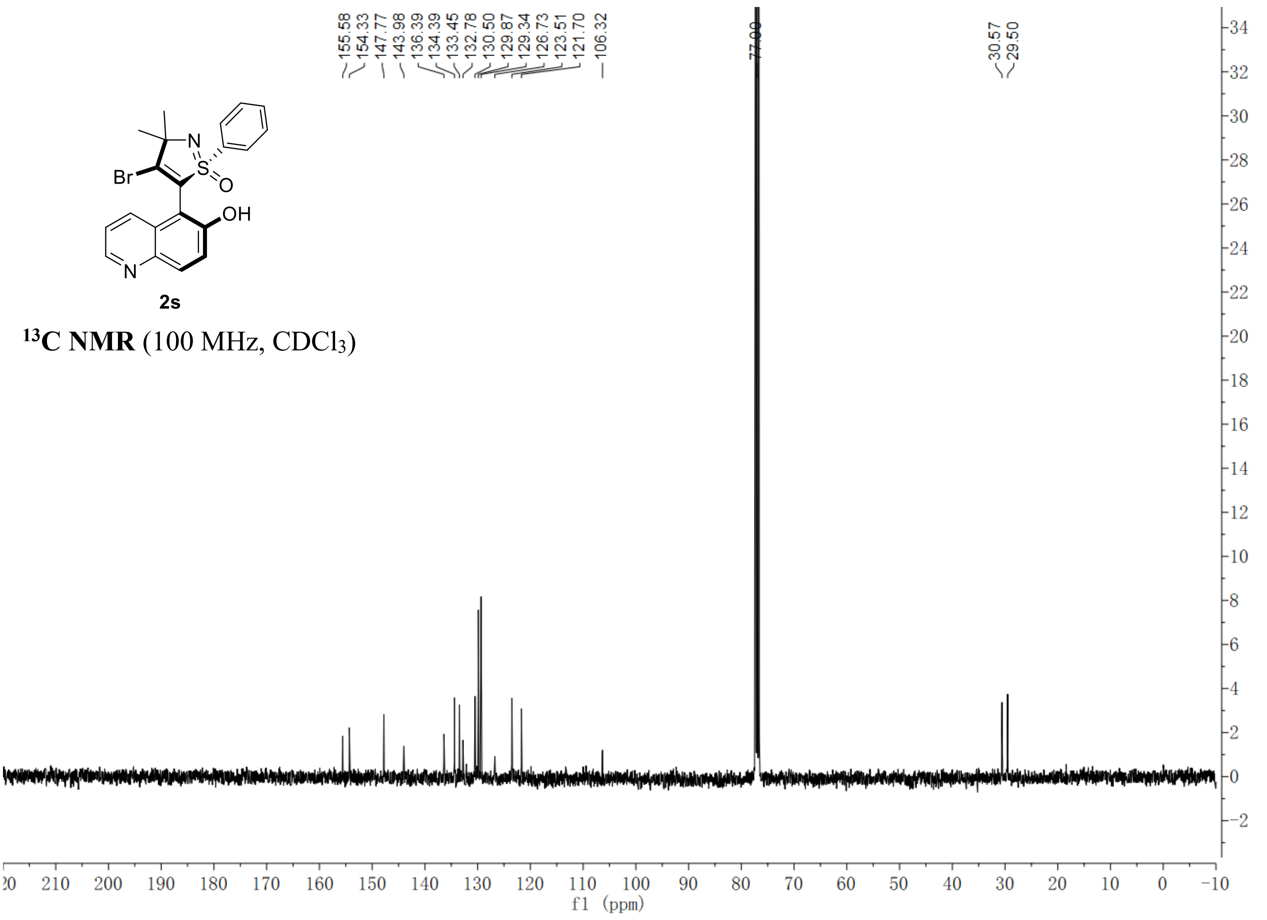

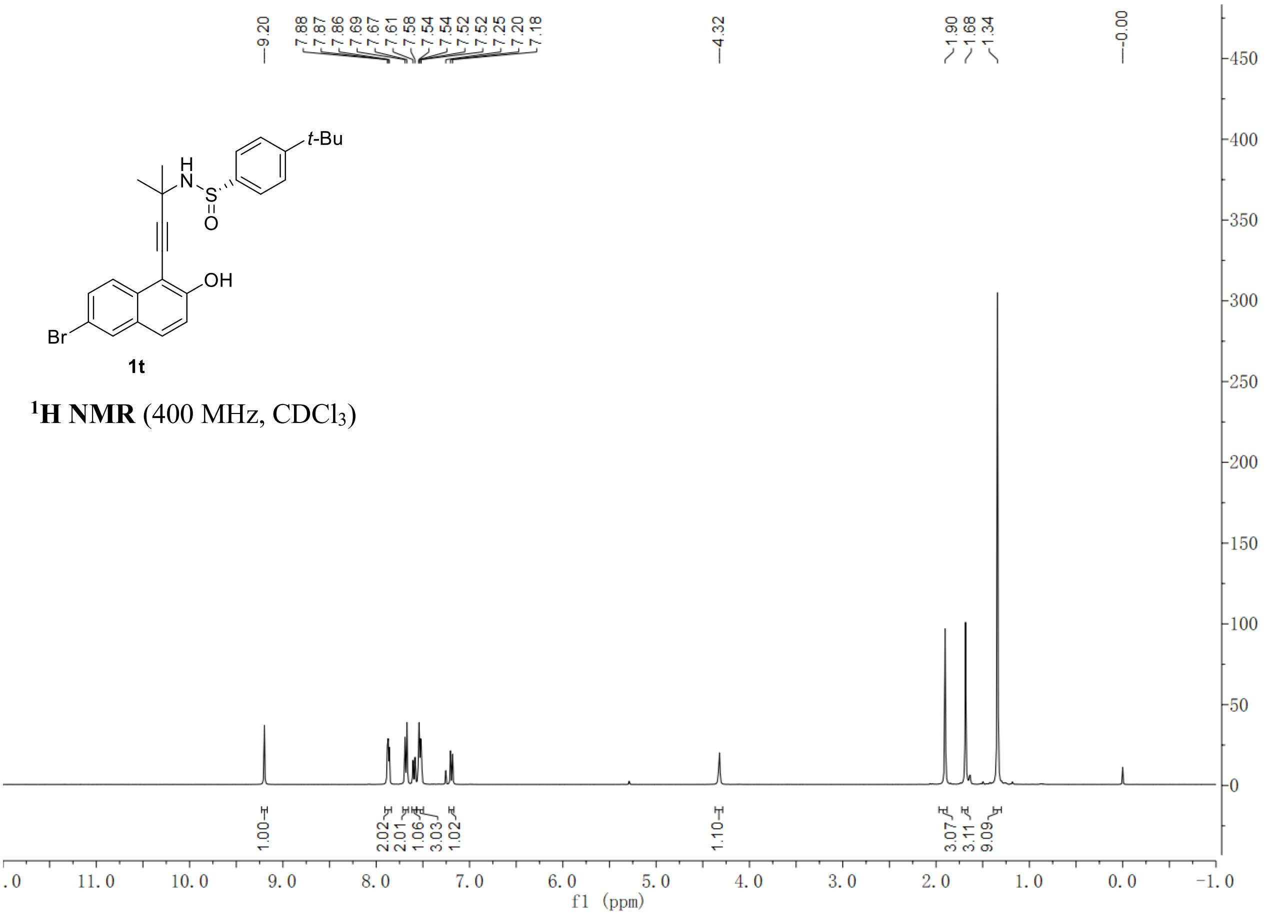

1t

^{1}H NMR (400 MHz, $CDCl_3$)

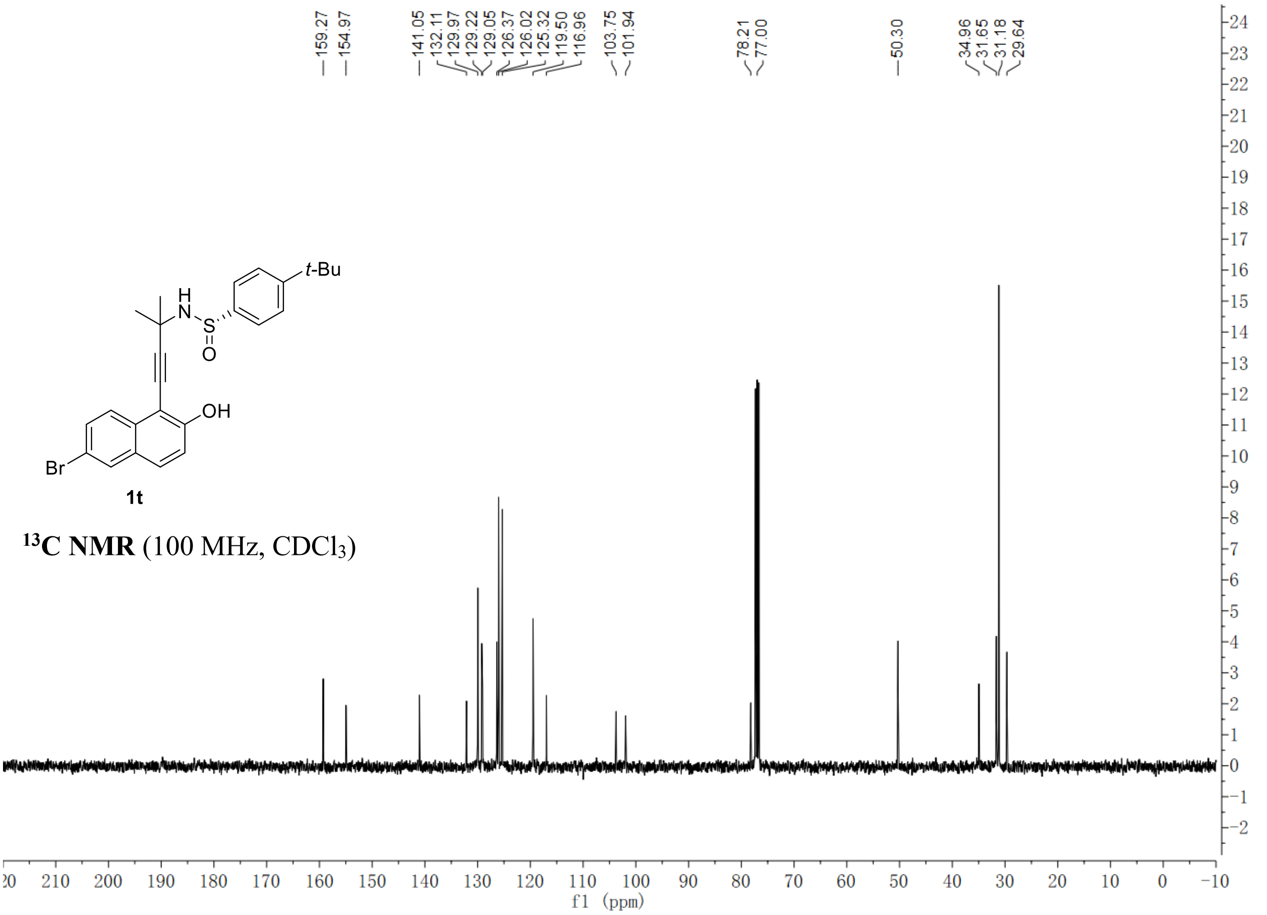

1t

^{13}C NMR (100 MHz, $CDCl_3$)

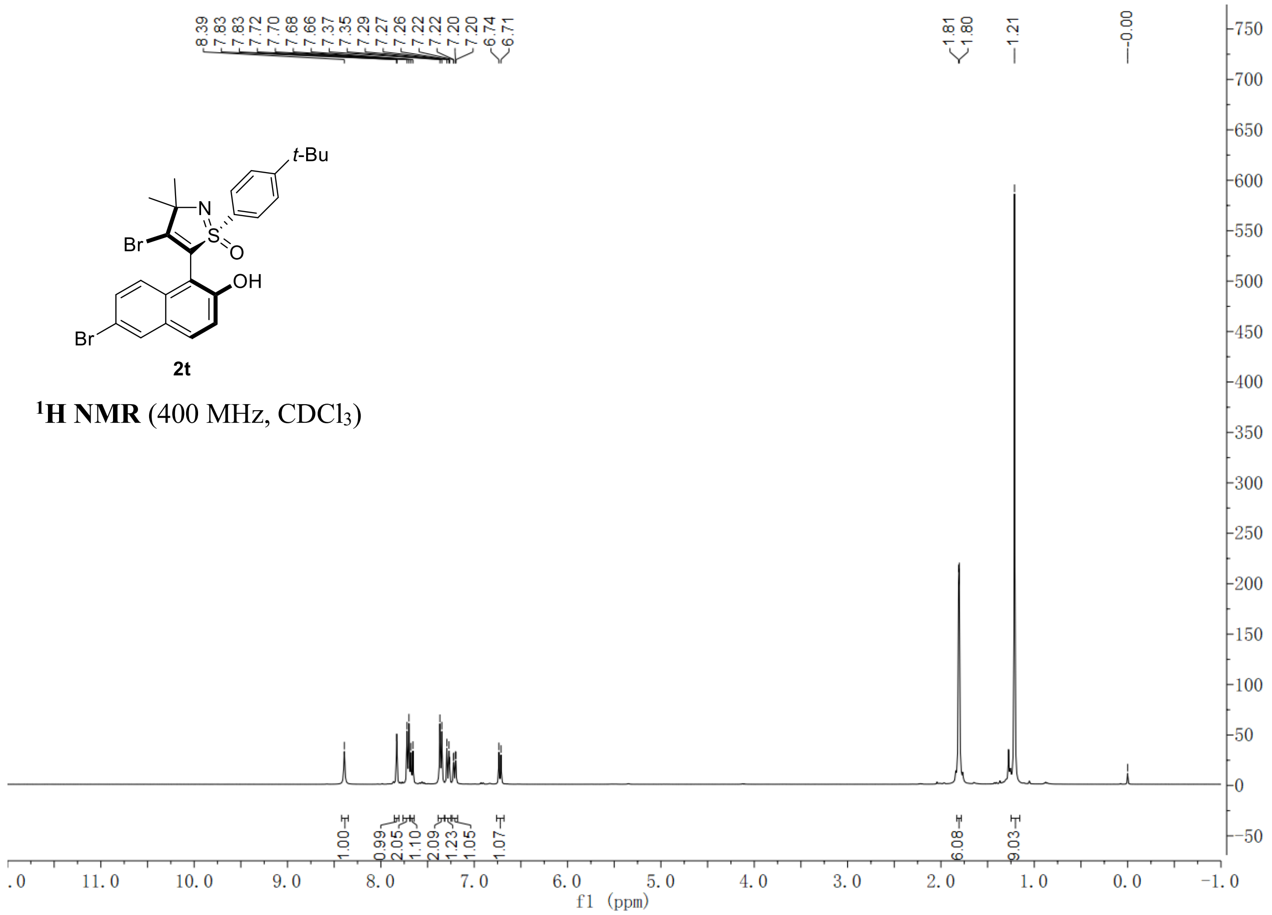

t-Bu
N
S
O
Br
OH
Br
2t
1H NMR (400 MHz, CDCl3)
8.39
7.83
7.83
7.72
7.70
7.68
7.66
7.37
7.35
7.29
7.27
7.26
7.22
7.22
7.20
7.20
6.74
6.71
1.81
1.80
1.21
0.00
1.00
0.99
2.05
1.10
2.09
1.23
1.05
1.07
6.08
9.03
f1 (ppm)

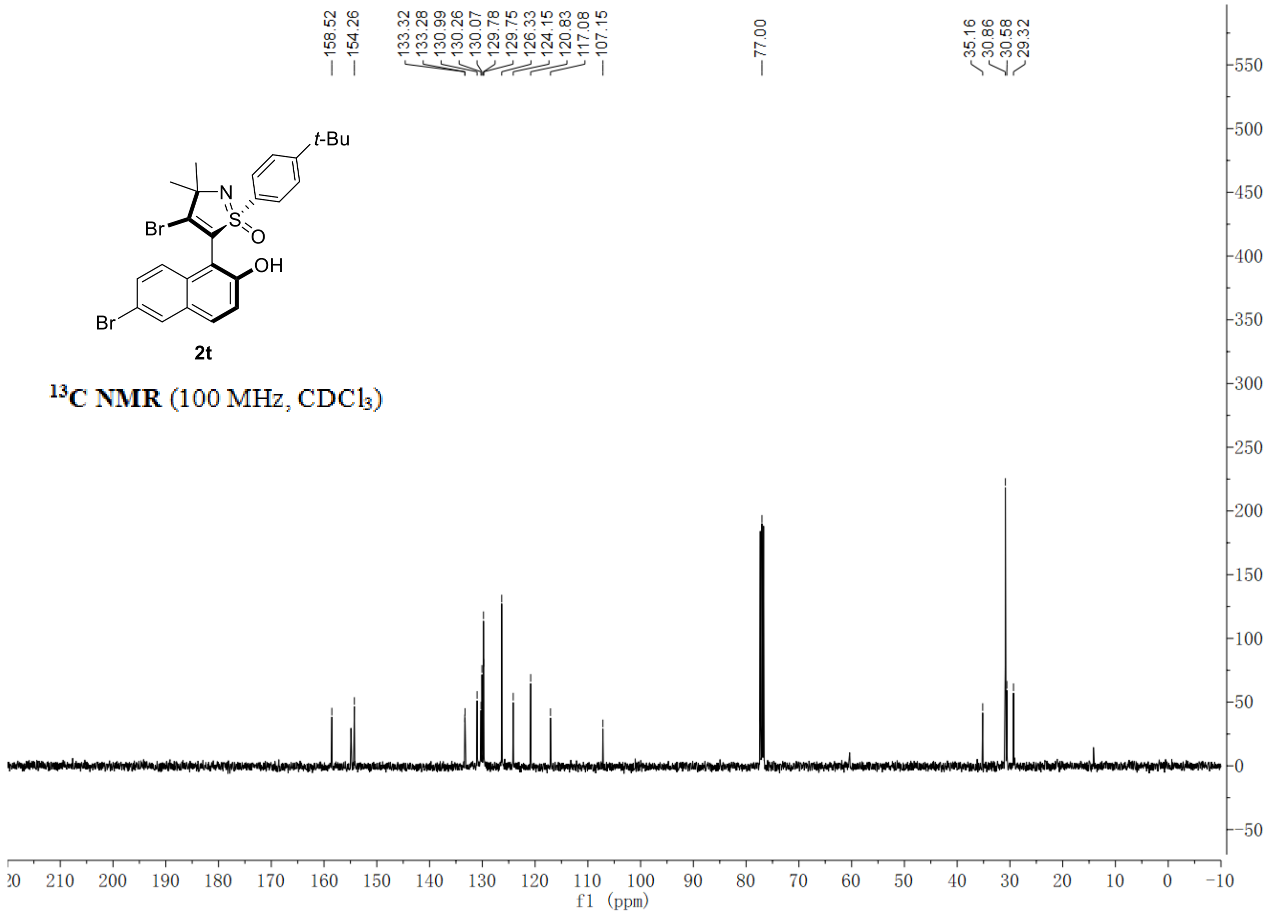

t-Bu
N
S
O
Br
OH
Br
2t
13C NMR (100 MHz, CDCl3)
158.52
154.26
133.32
133.28
130.99
130.26
130.07
129.78
129.75
126.33
124.15
120.83
117.08
107.15
77.00
35.16
30.86
30.58
29.32
f1 (ppm)

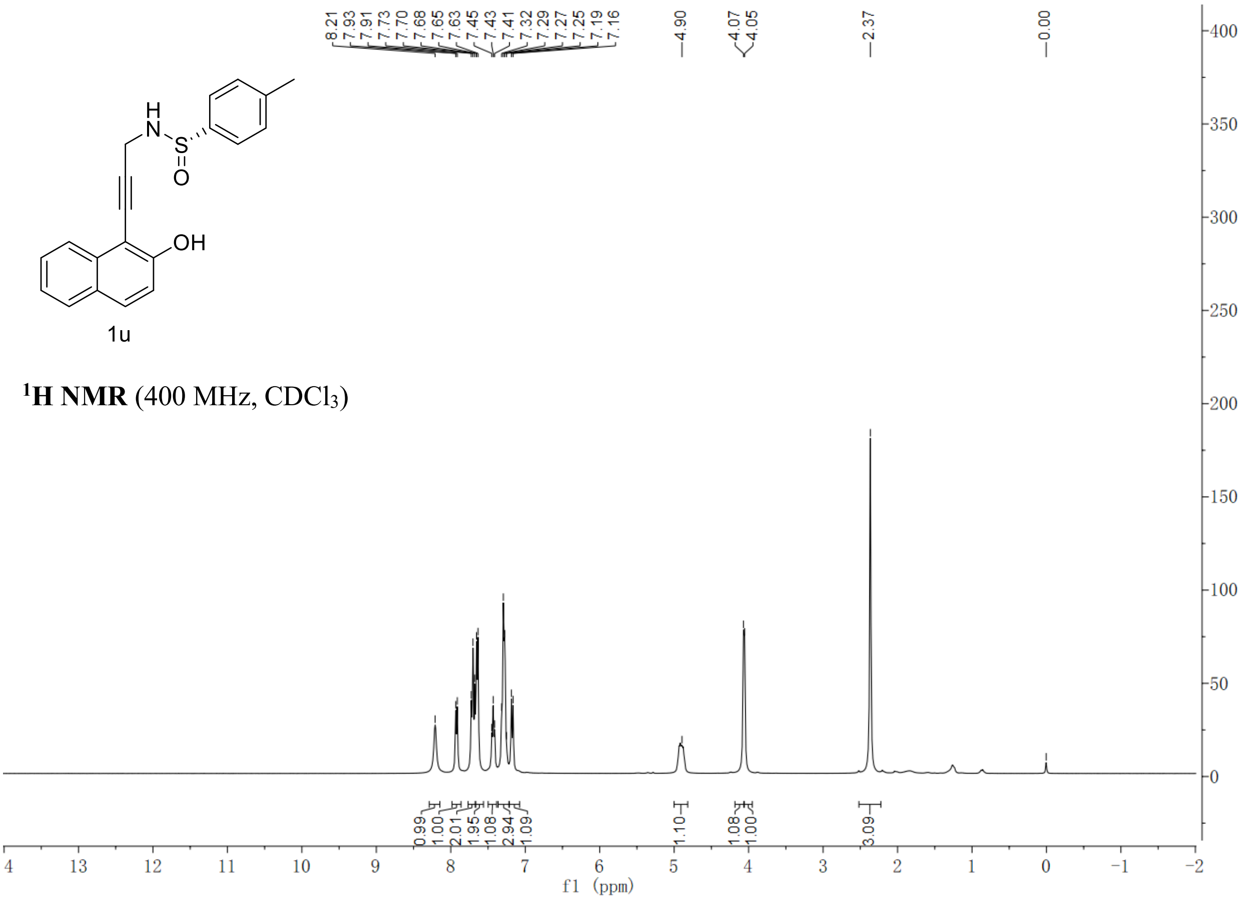

1u

^{1}H NMR (400 MHz, $CDCl_3$)

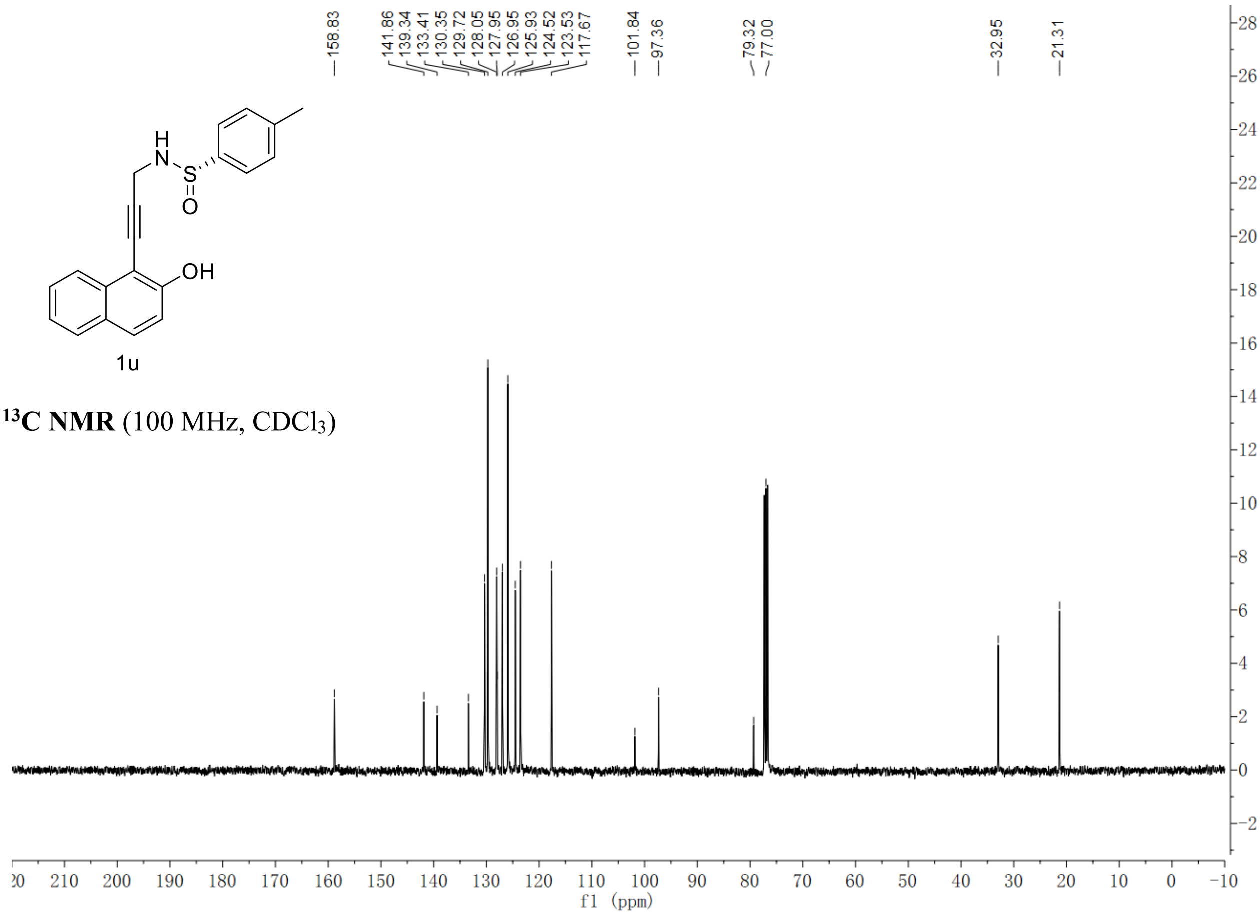

1u

^{13}C NMR (100 MHz, $CDCl_3$)

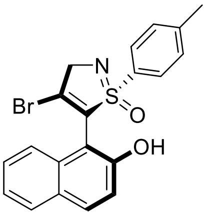

2u

^{1}H NMR (400 MHz, $CDCl_3$)

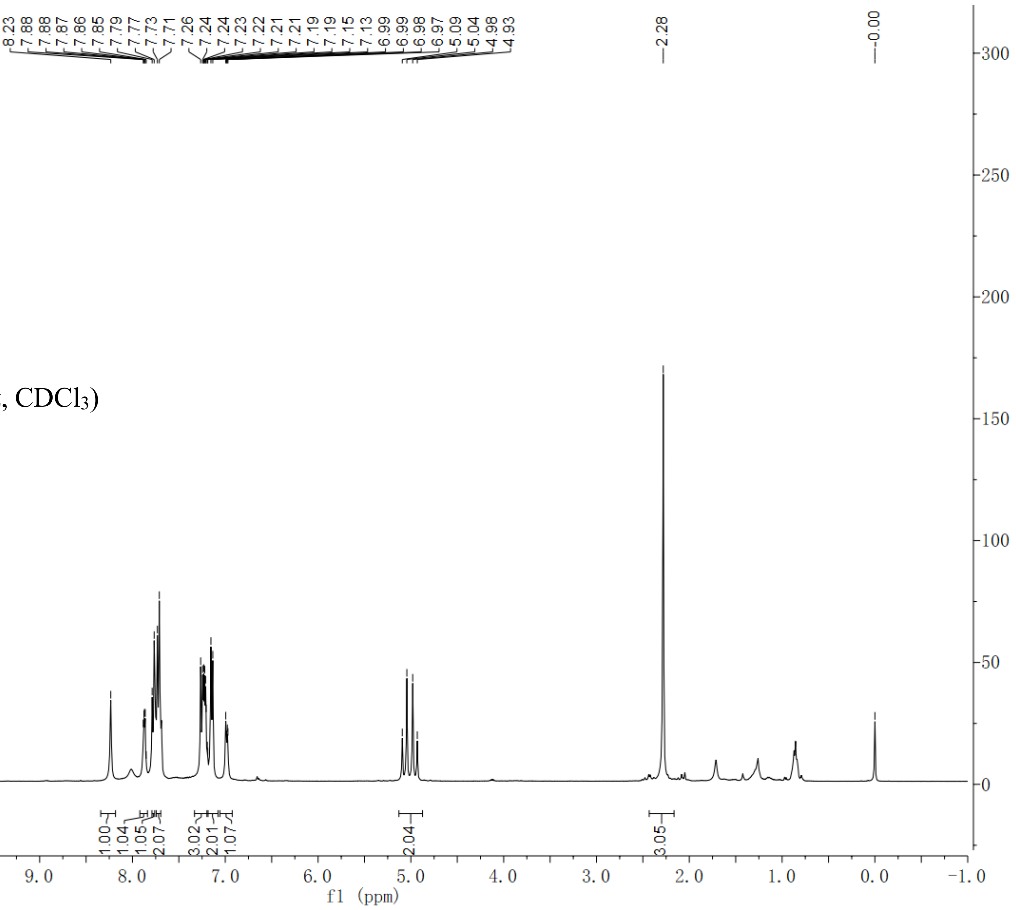

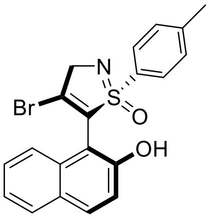

2u

^{13}C NMR (100 MHz, $CDCl_3$)

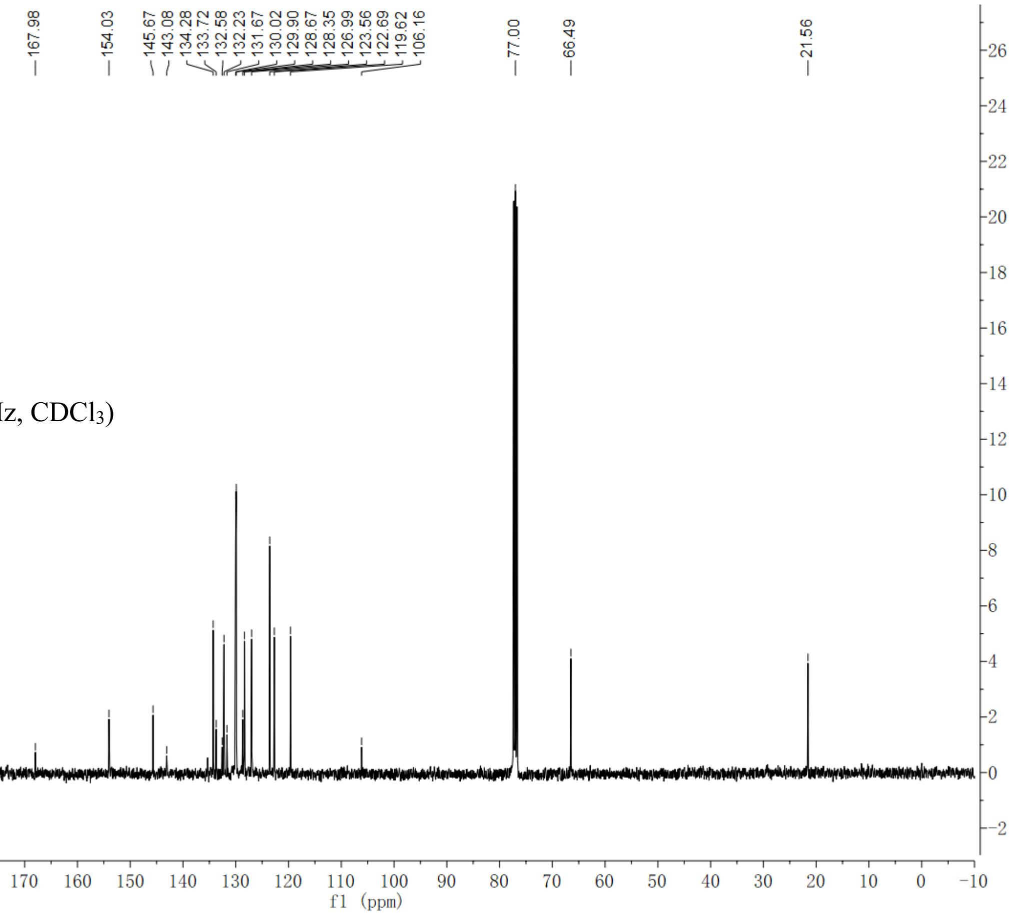

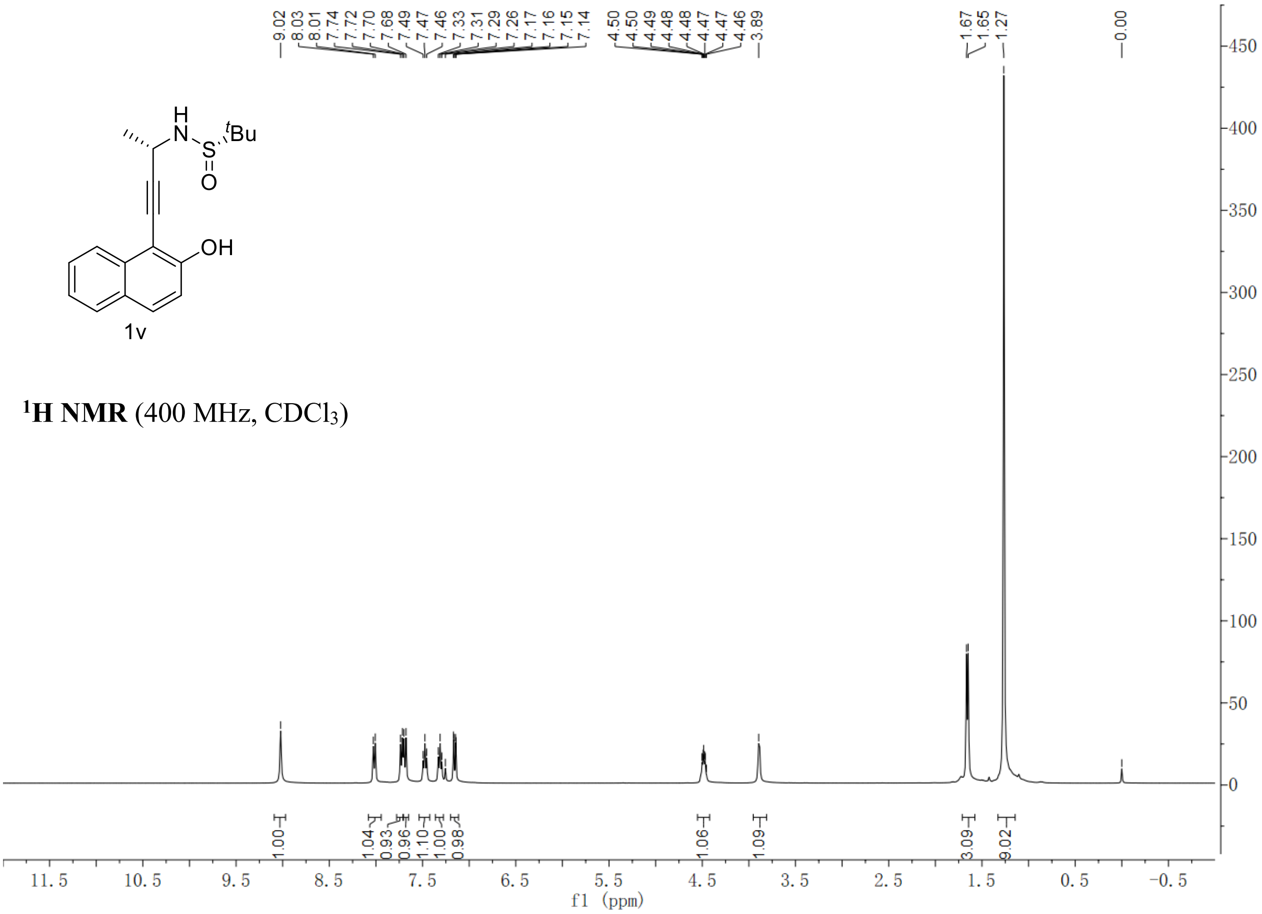

1v

^{1}H NMR (400 MHz, $CDCl_3$)

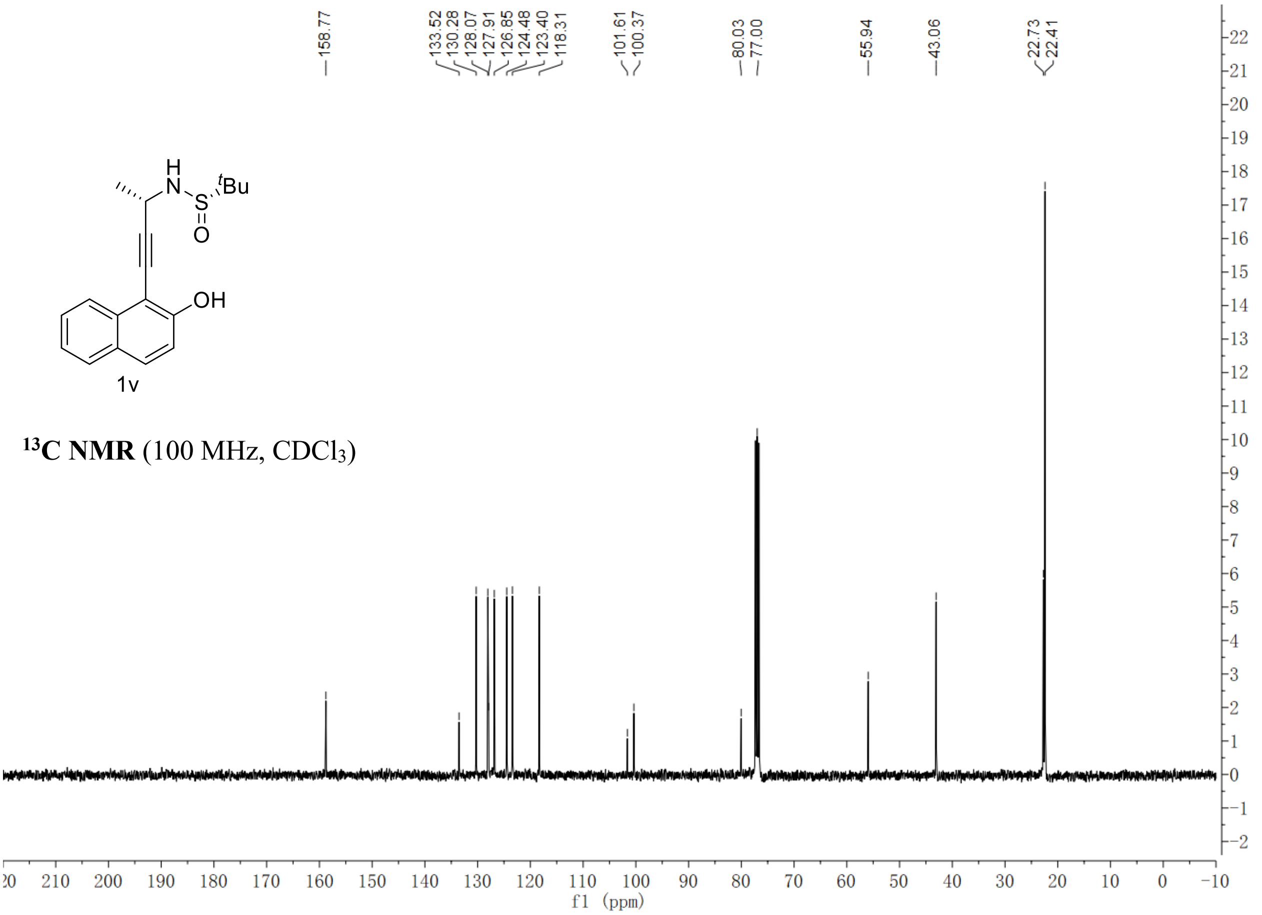

1v

^{13}C NMR (100 MHz, $CDCl_3$)

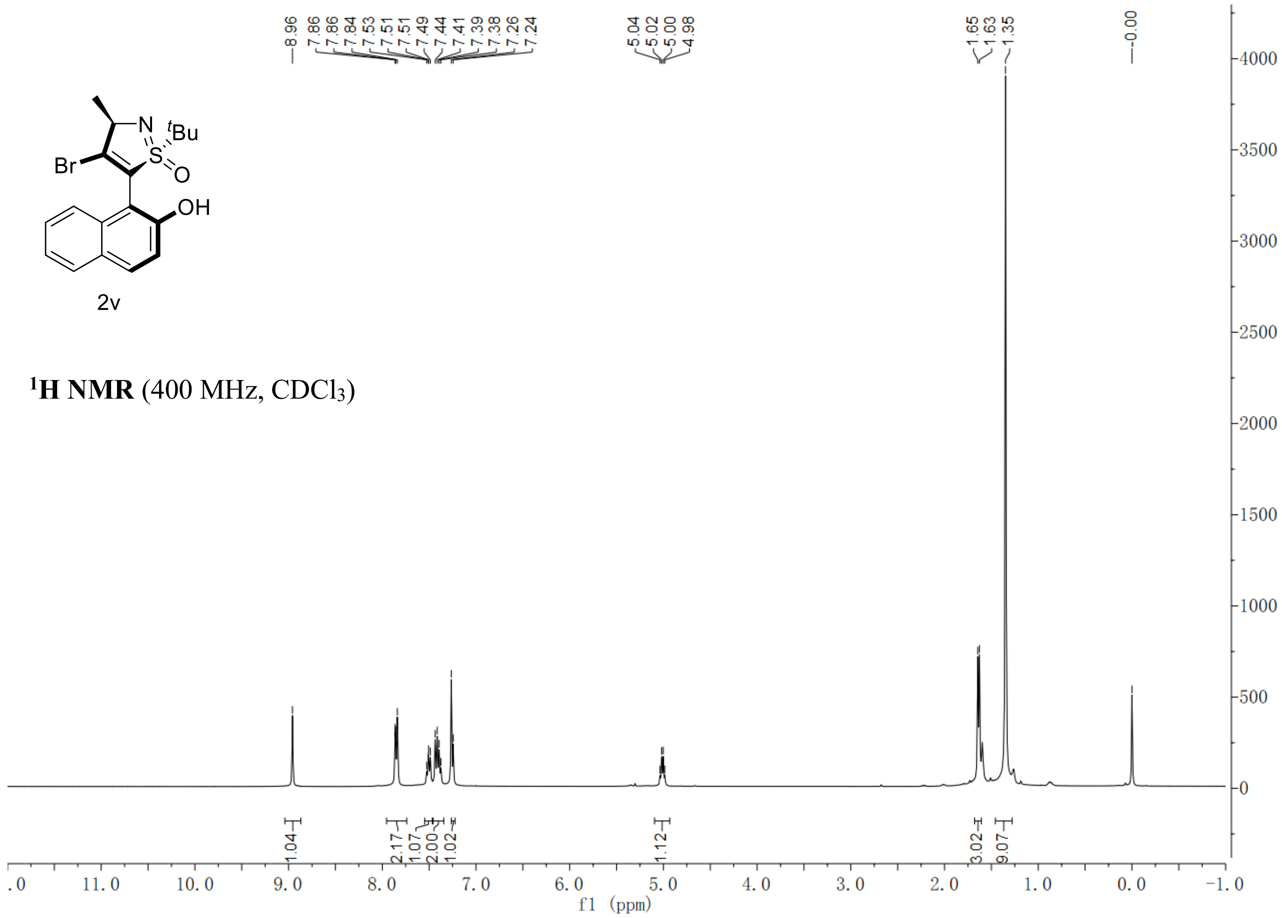

2v

¹H NMR (400 MHz, $CDCl_3$)

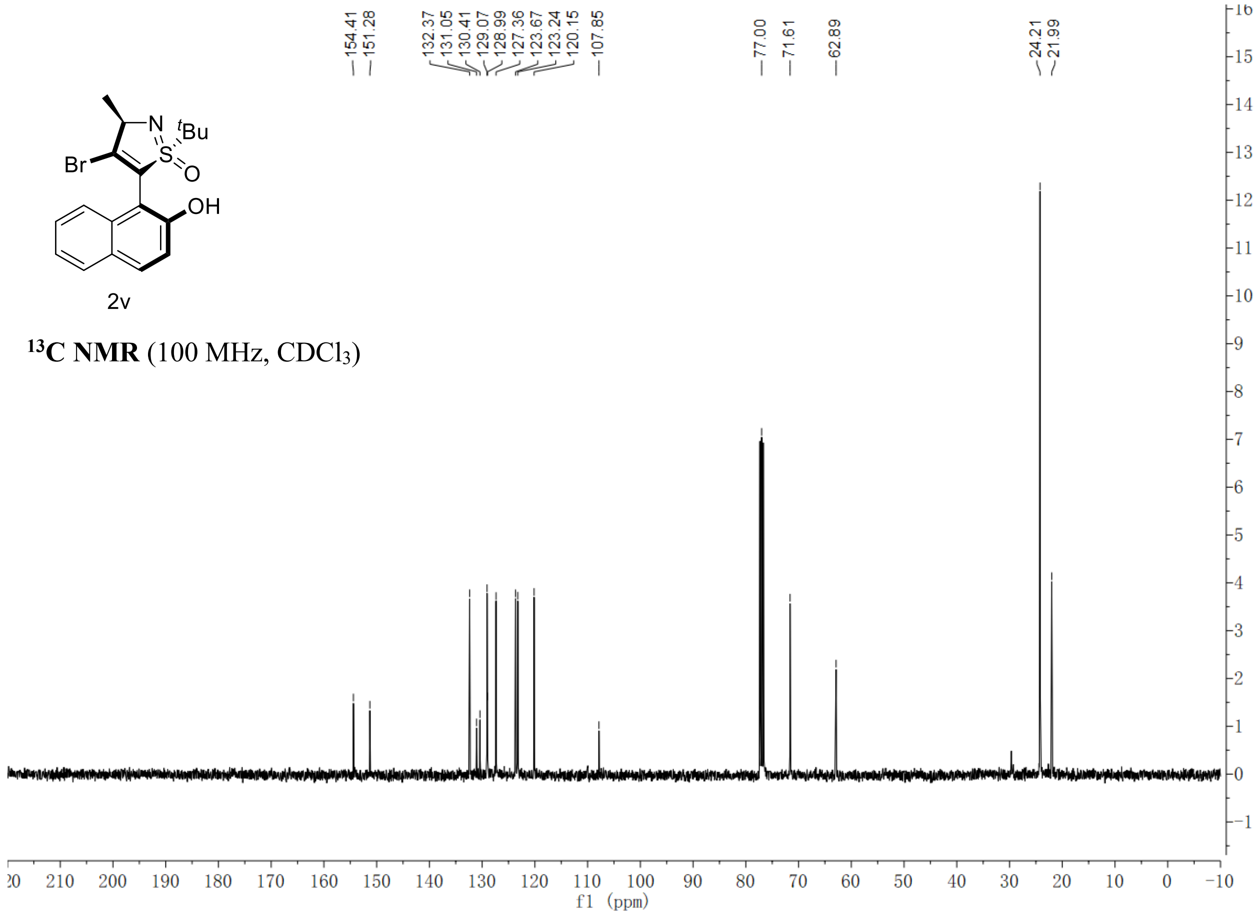

2v

¹³C NMR (100 MHz, $CDCl_3$)

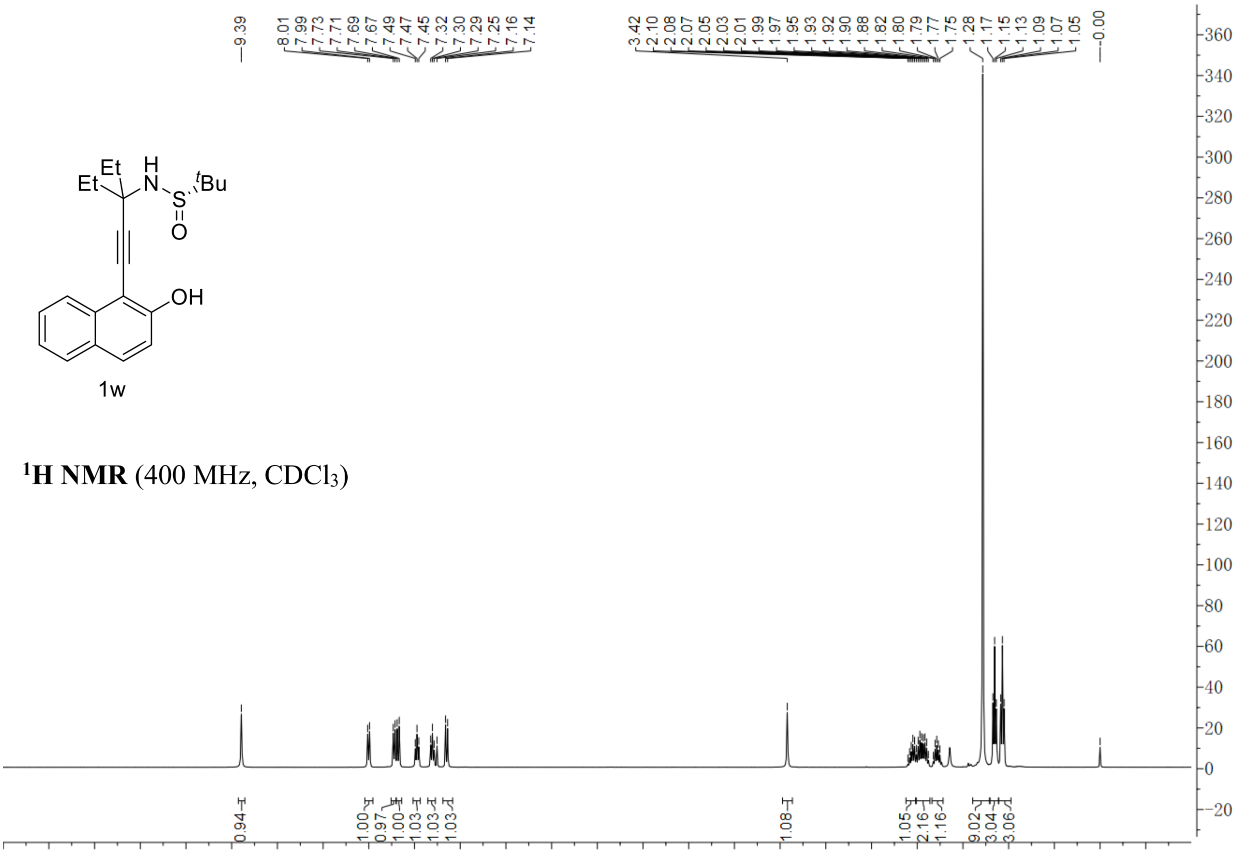

¹H NMR (400 MHz, $CDCl_3$)

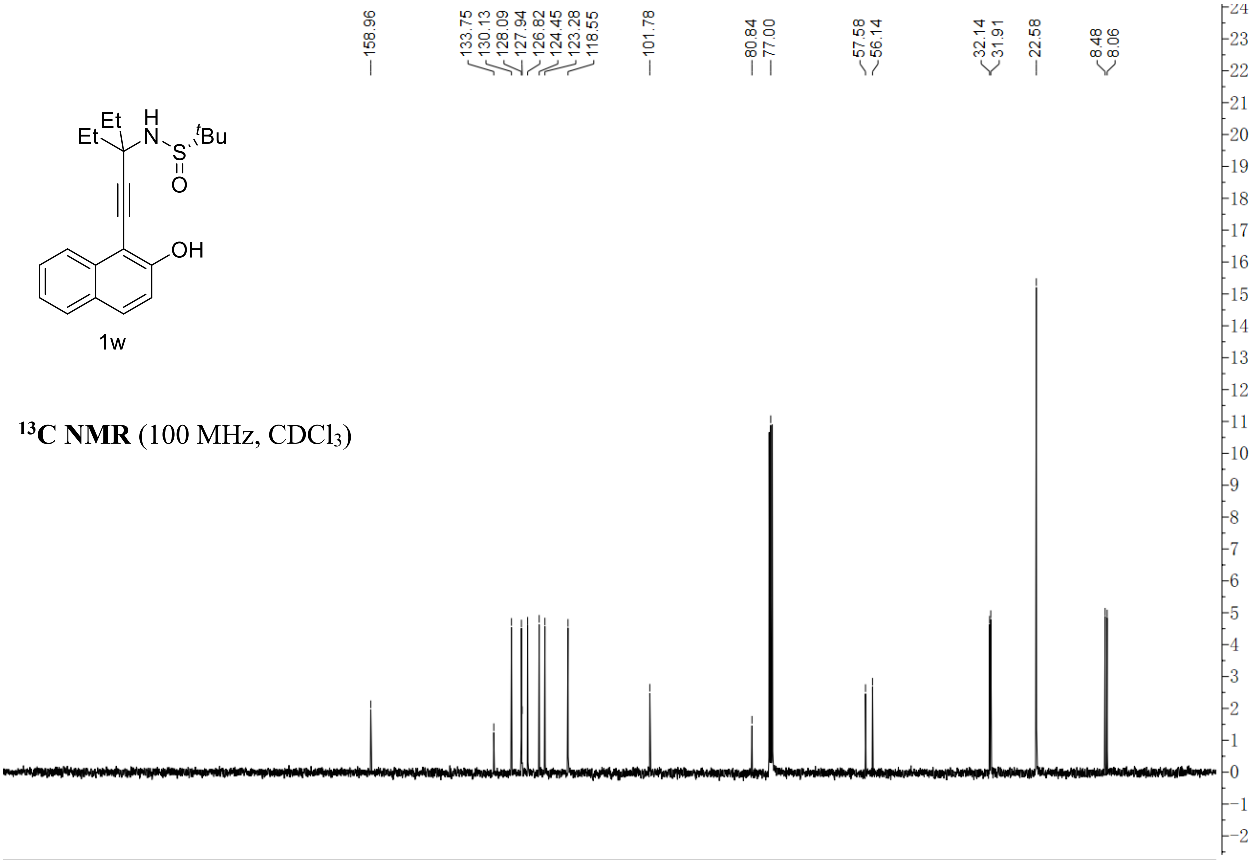

¹³C NMR (100 MHz, $CDCl_3$)

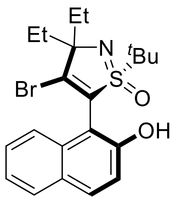

2w

^{1}H NMR (400 MHz, $CDCl_3$)

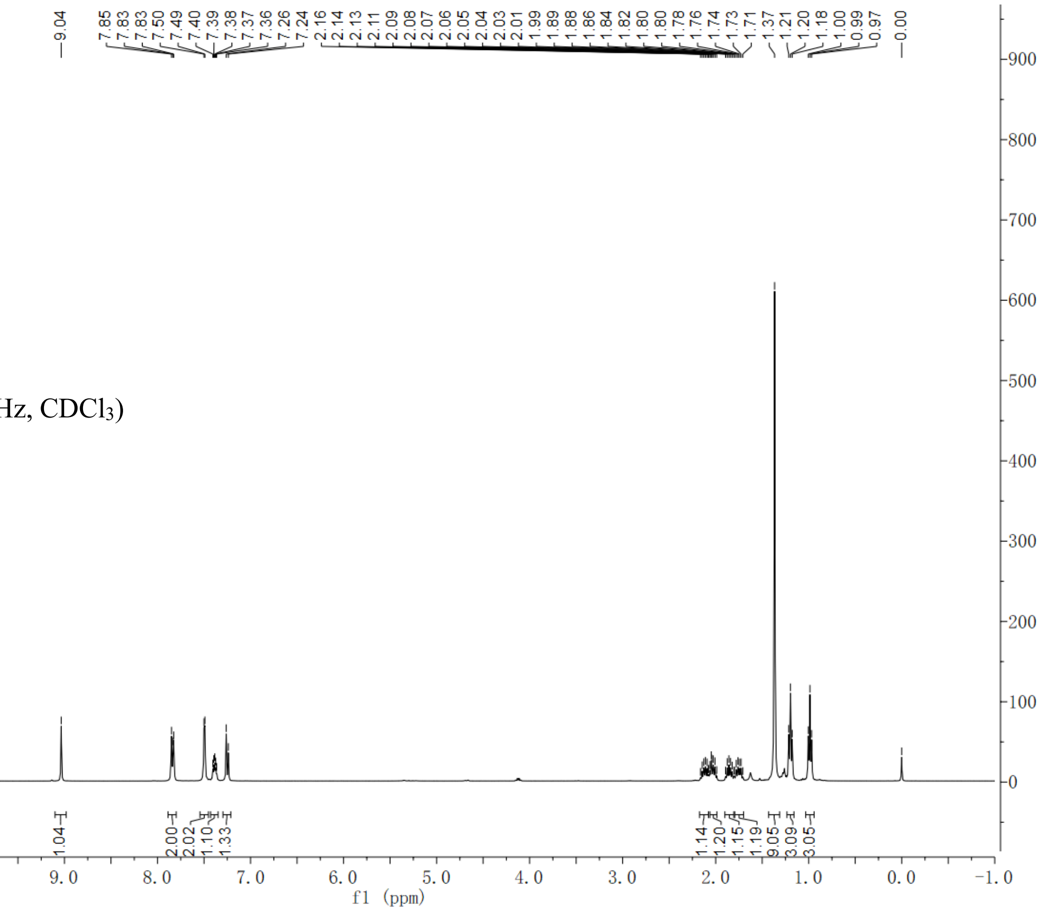

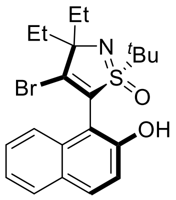

2w

^{13}C NMR (100 MHz, $CDCl_3$)

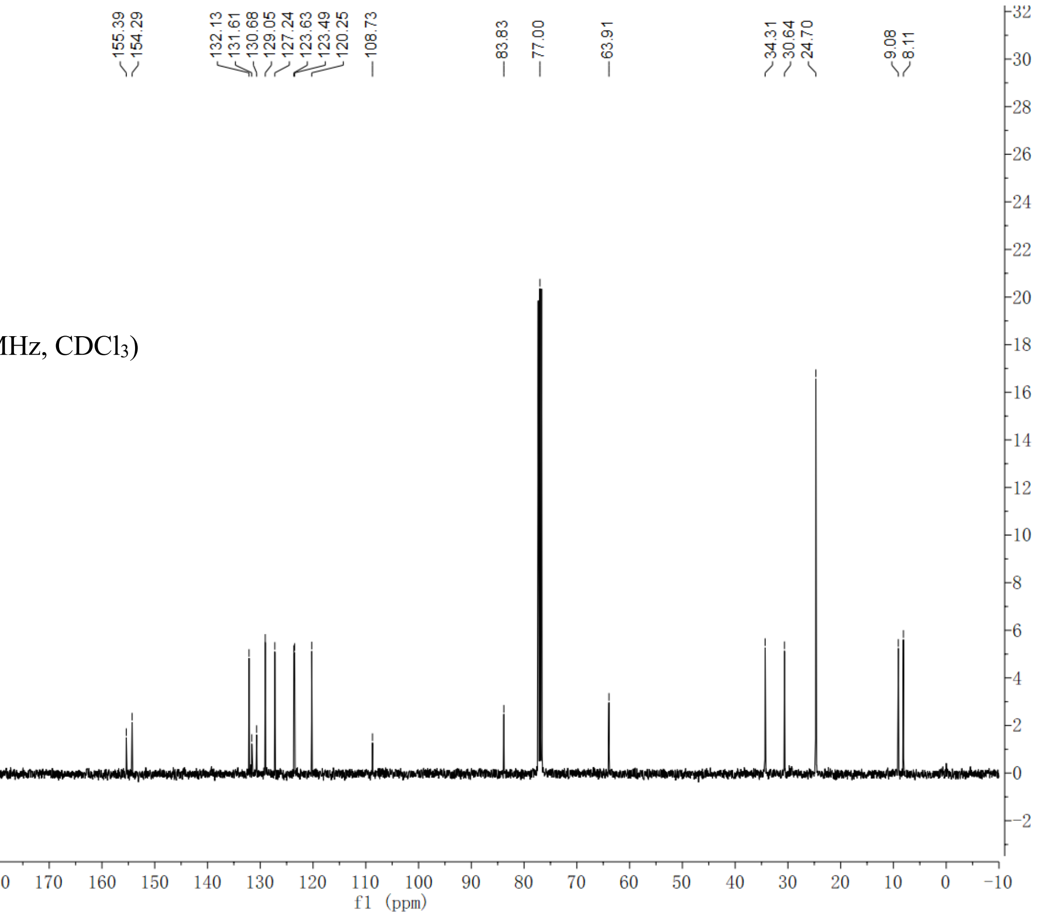

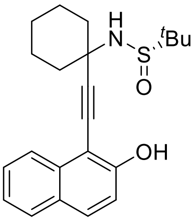

1x

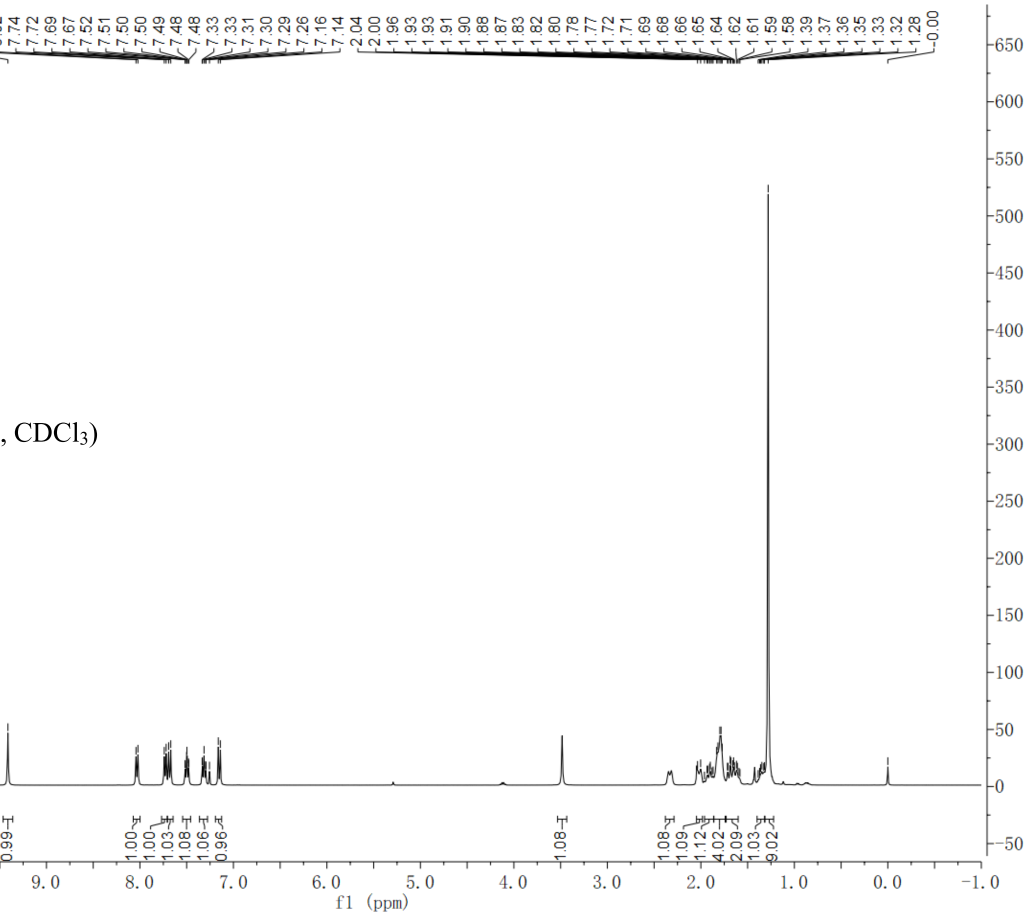

^{1}H NMR (400 MHz, $CDCl_3$)

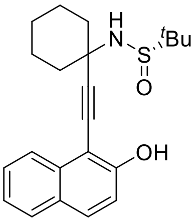

1x

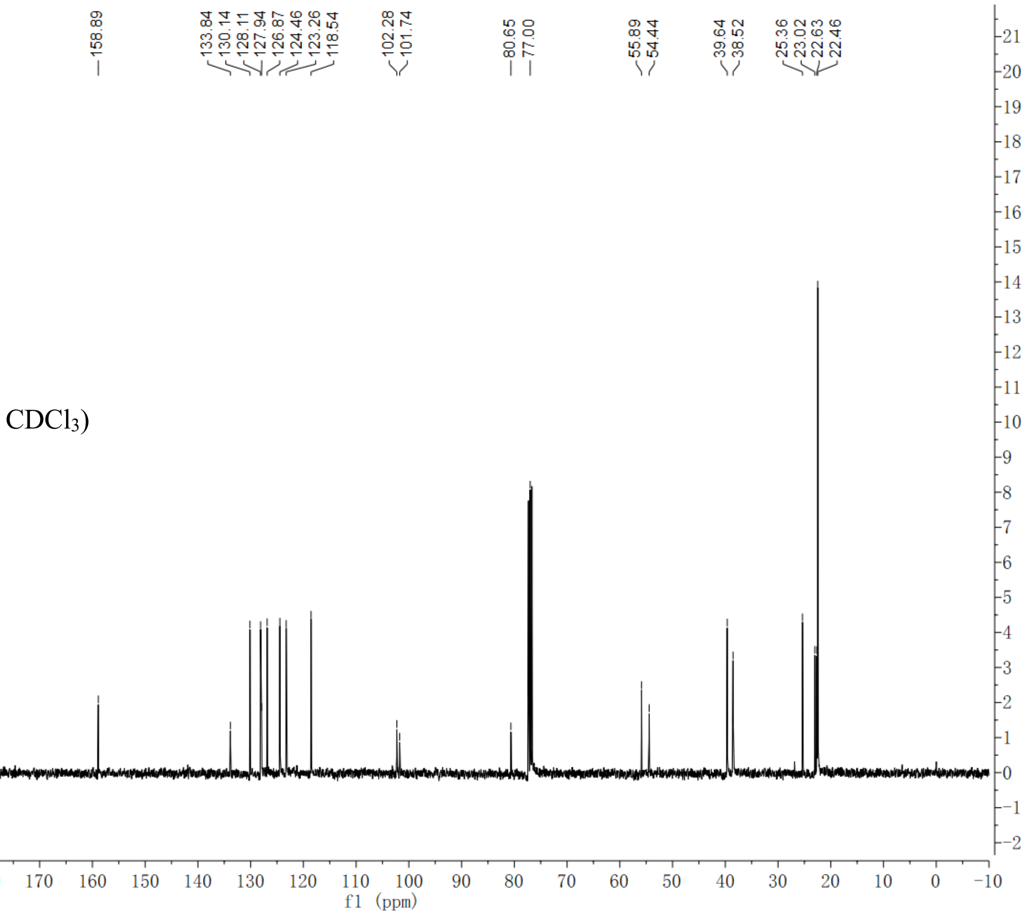

^{13}C NMR (100 MHz, $CDCl_3$)

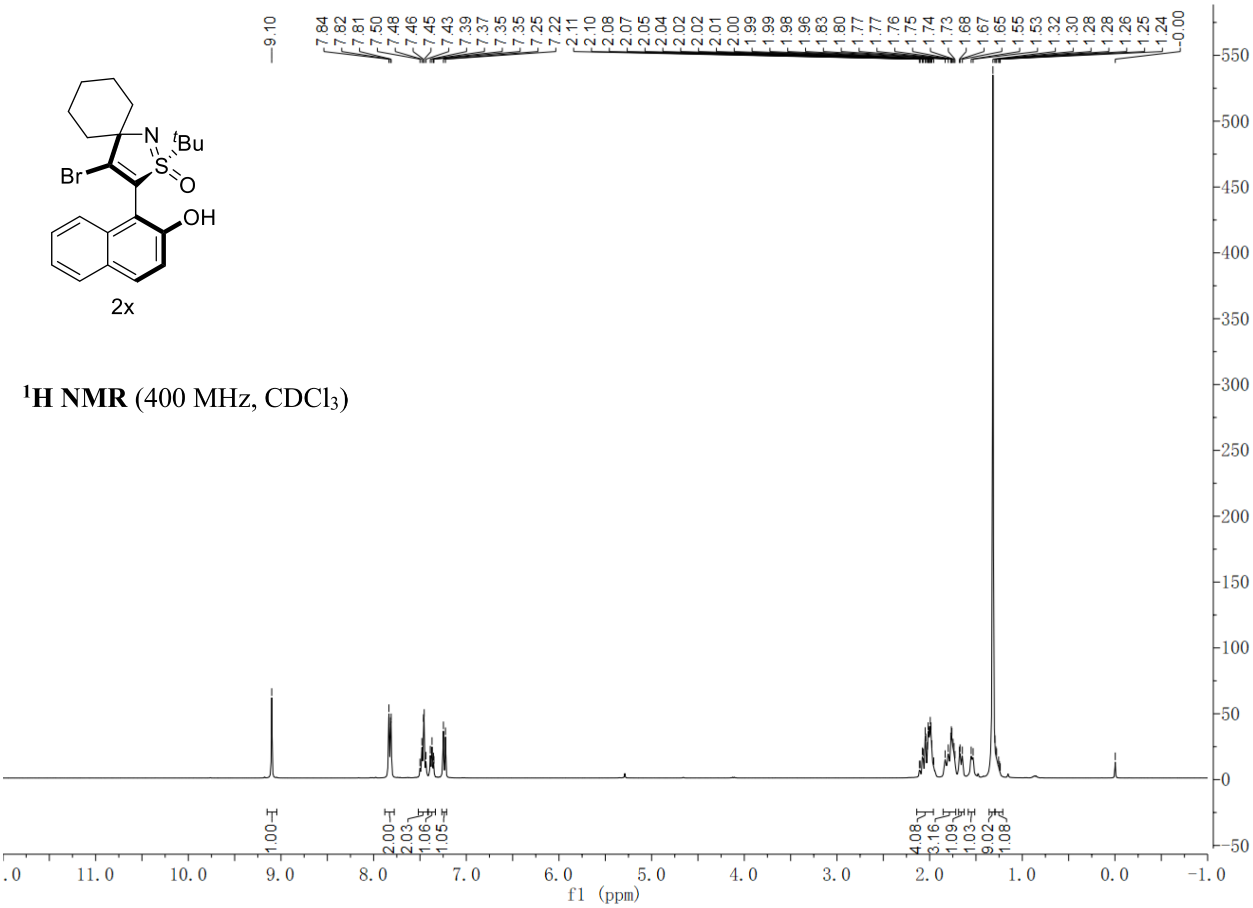

^{1}H NMR (400 MHz, $CDCl_3$)

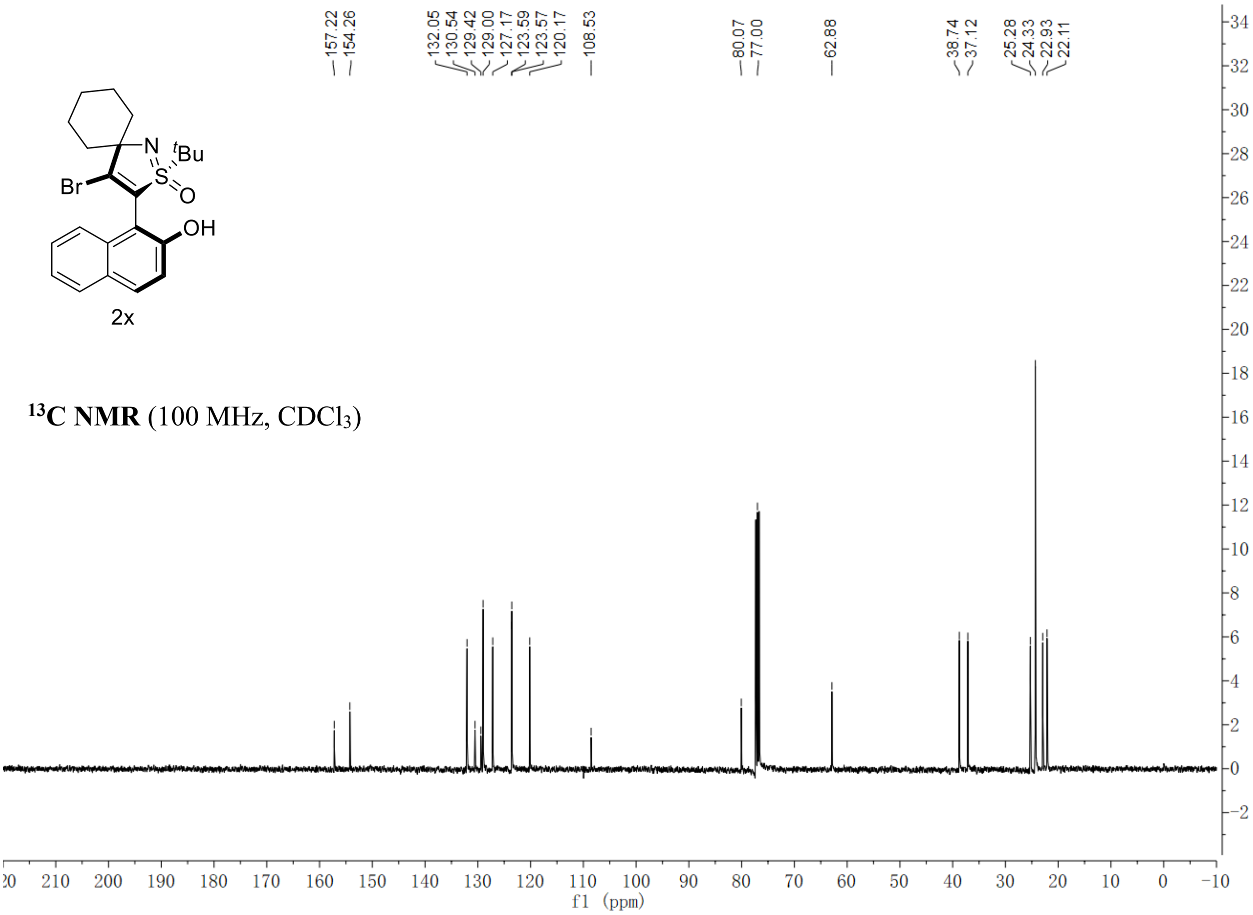

^{13}C NMR (100 MHz, $CDCl_3$)

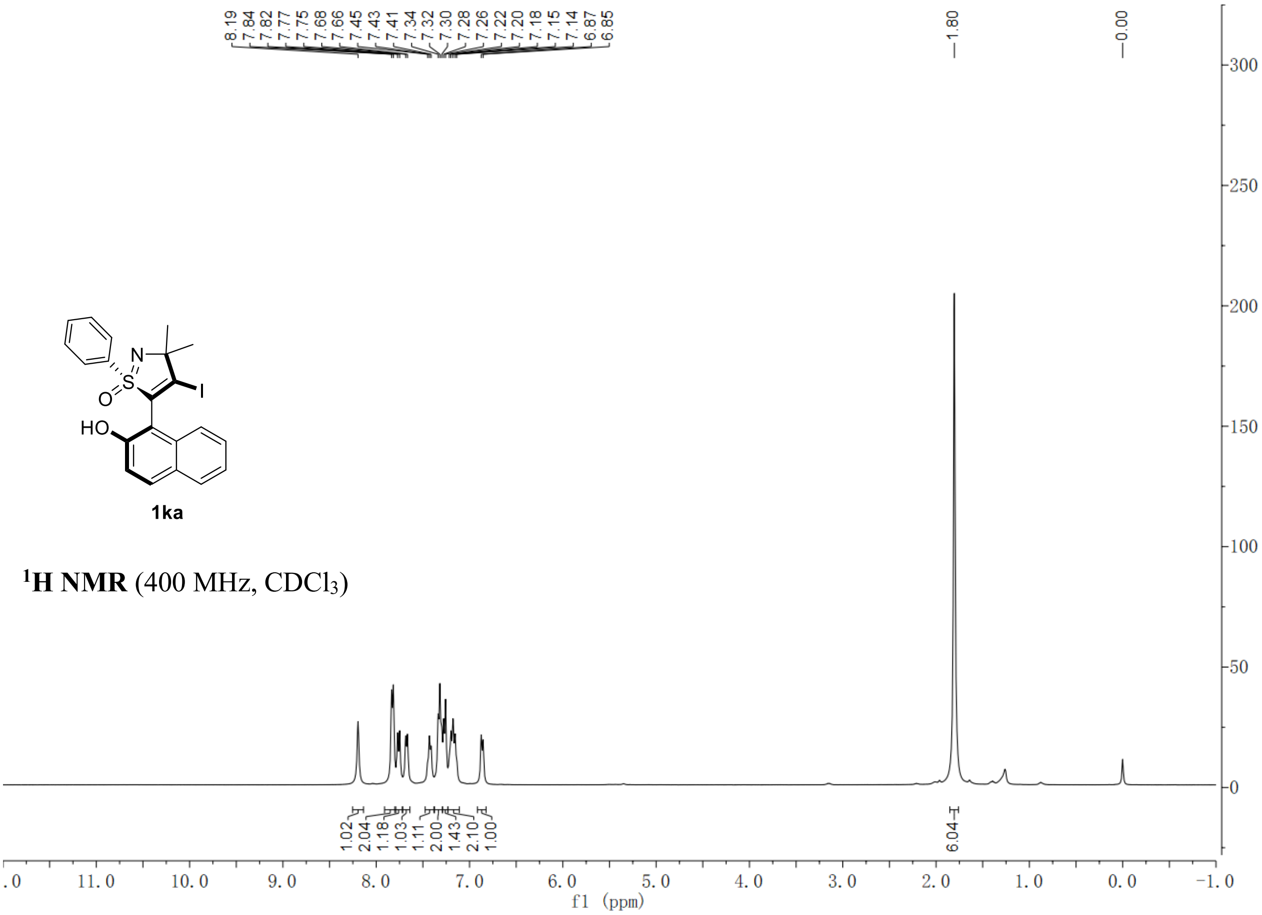

^{1}H NMR (400 MHz, $CDCl_3$)

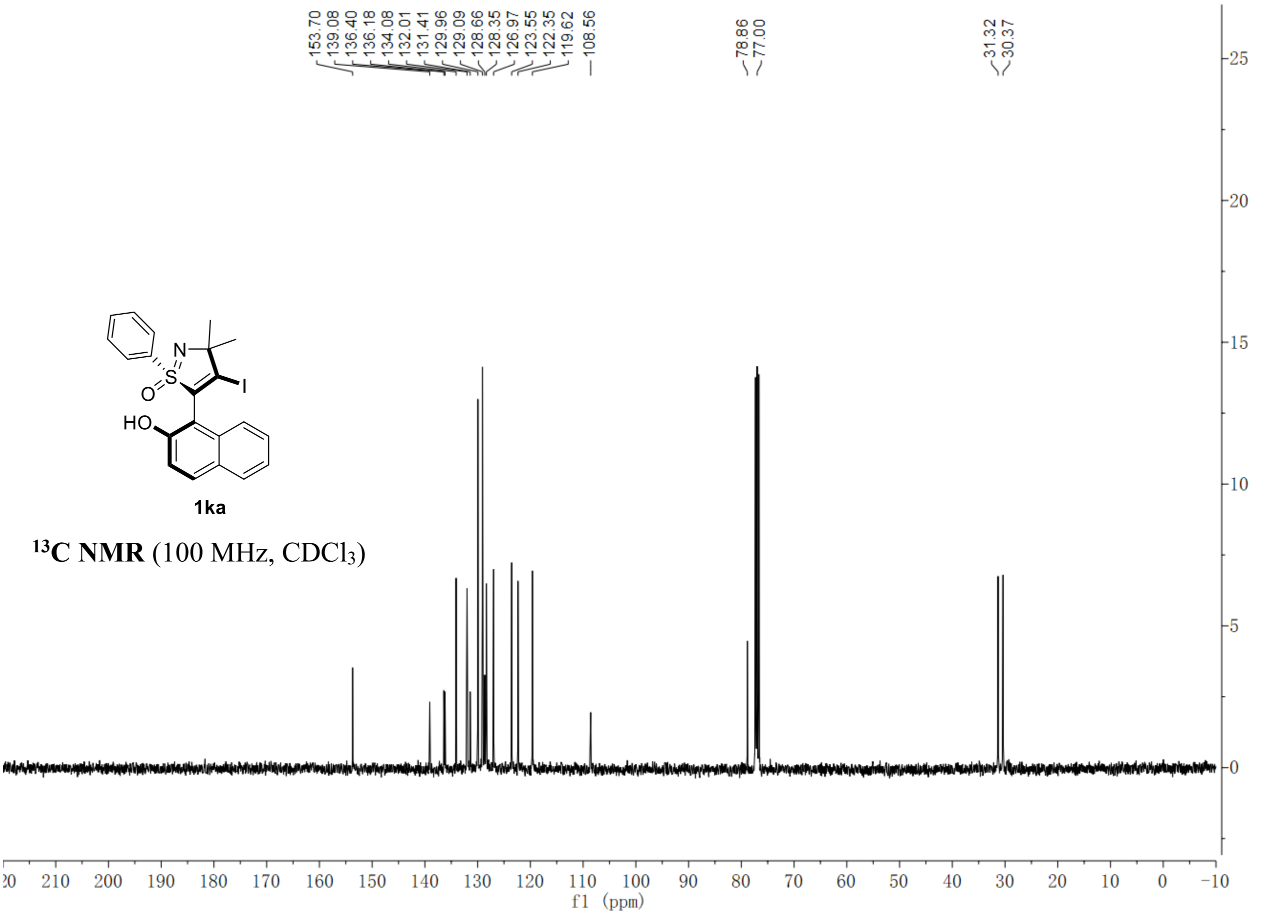

^{13}C NMR (100 MHz, $CDCl_3$)

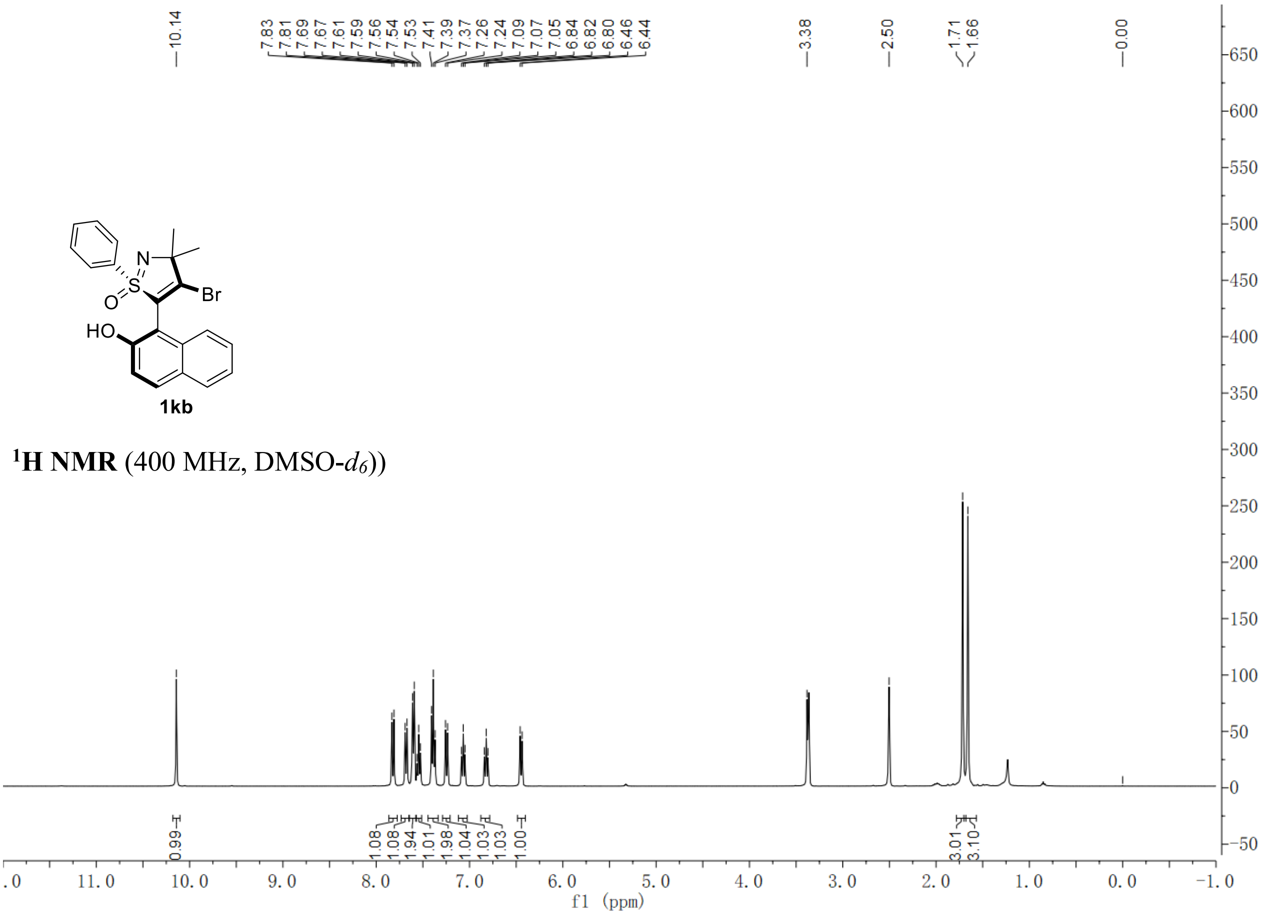

^{1}H NMR (400 MHz, DMSO-d_6))

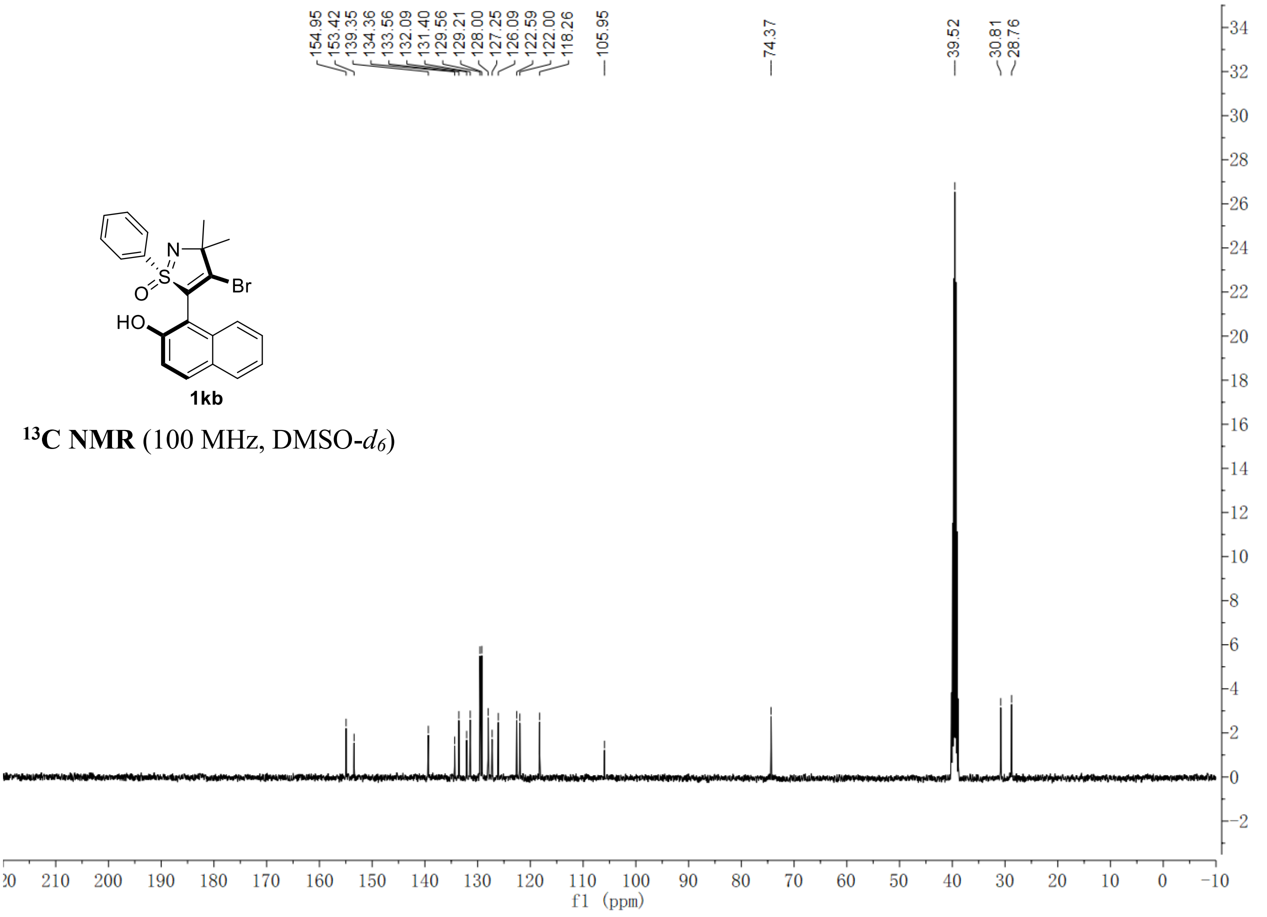

^{13}C NMR (100 MHz, DMSO-d_6)

1kc

^{1}H NMR (400 MHz, $CDCl_3$)

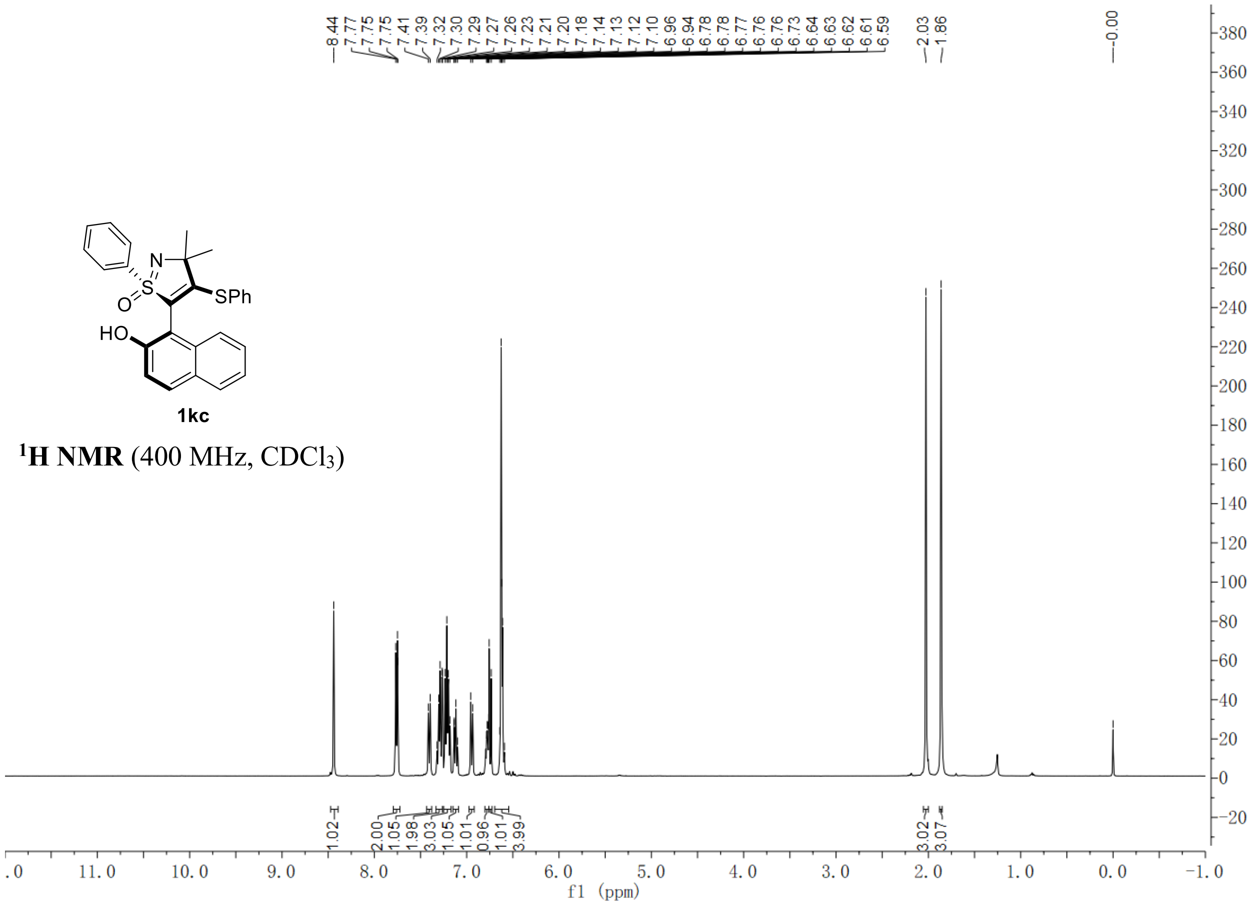

1kc

^{13}C NMR (100 MHz, $CDCl_3$)

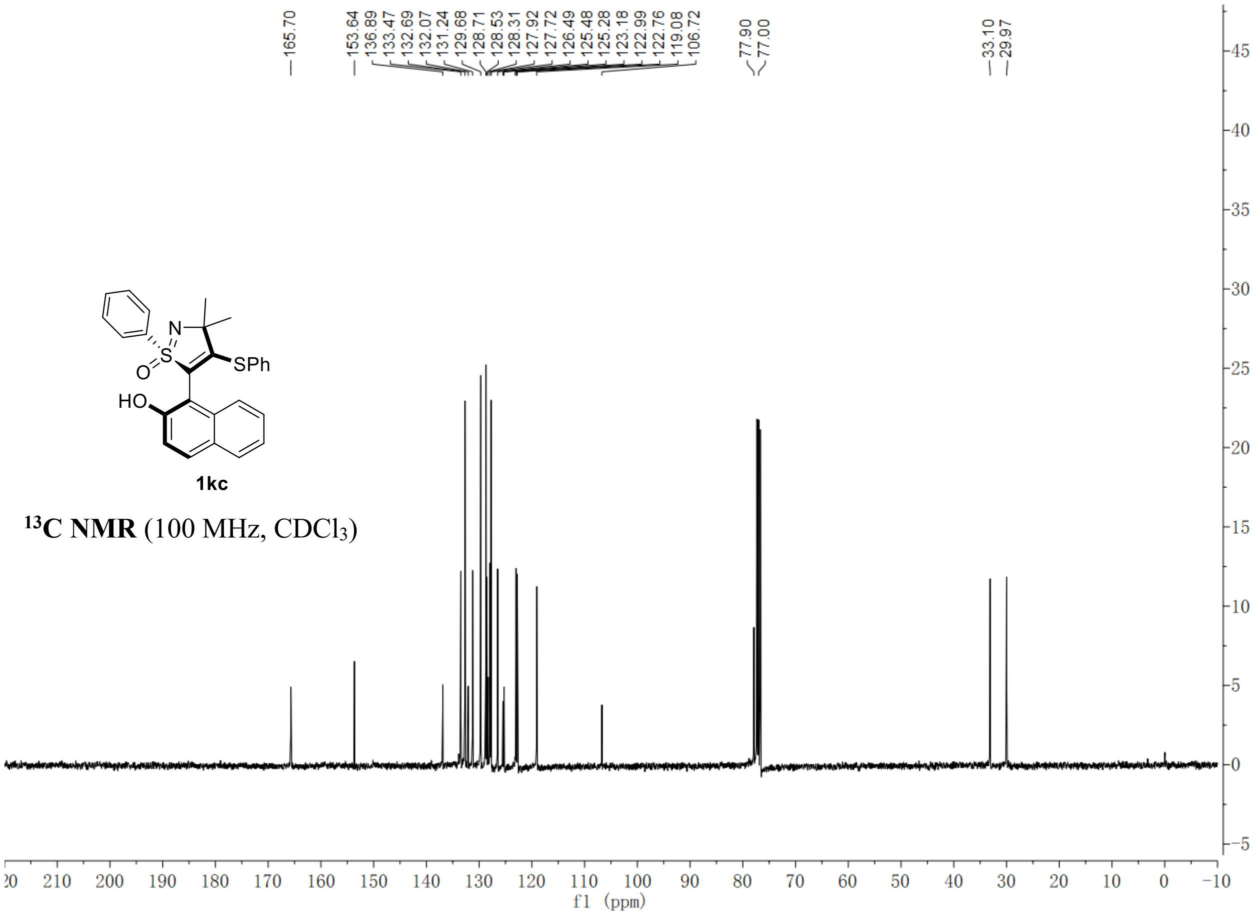

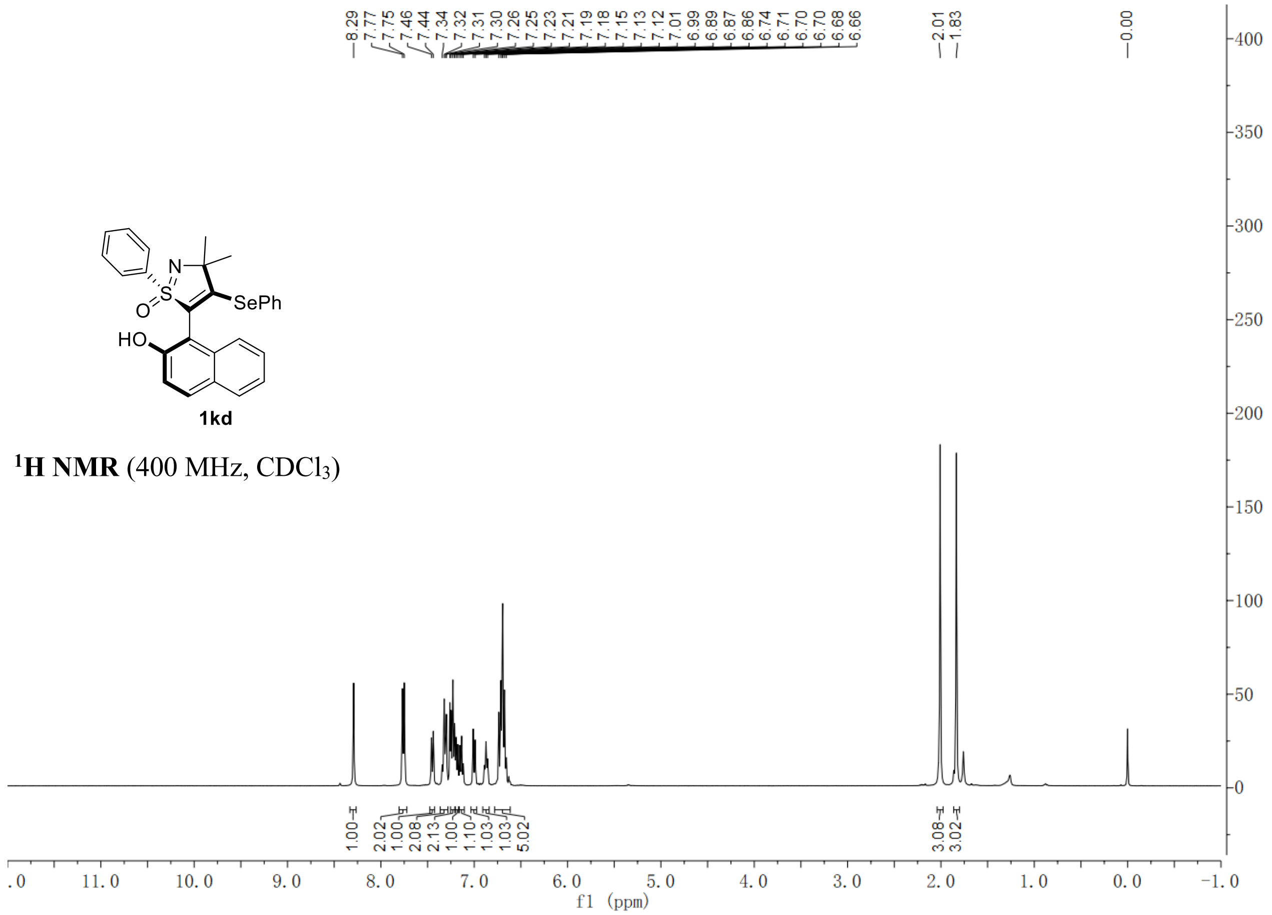
8.29 7.77 7.75 7.46 7.44 7.34 7.32 7.31 7.30 7.26 7.25 7.23 7.21 7.19 7.18 7.15 7.13 7.12 7.01 6.99 6.89 6.87 6.86 6.74 6.71 6.70 6.68 6.66
2.01 1.83
0.00
SePh
HO
N
S
O
1kd
^{1}H NMR (400 MHz, CDCl$_3$)
1.00 2.02 1.00 2.08 2.13 1.00 1.10 1.03 1.03 5.02
3.08 3.02
f1 (ppm)

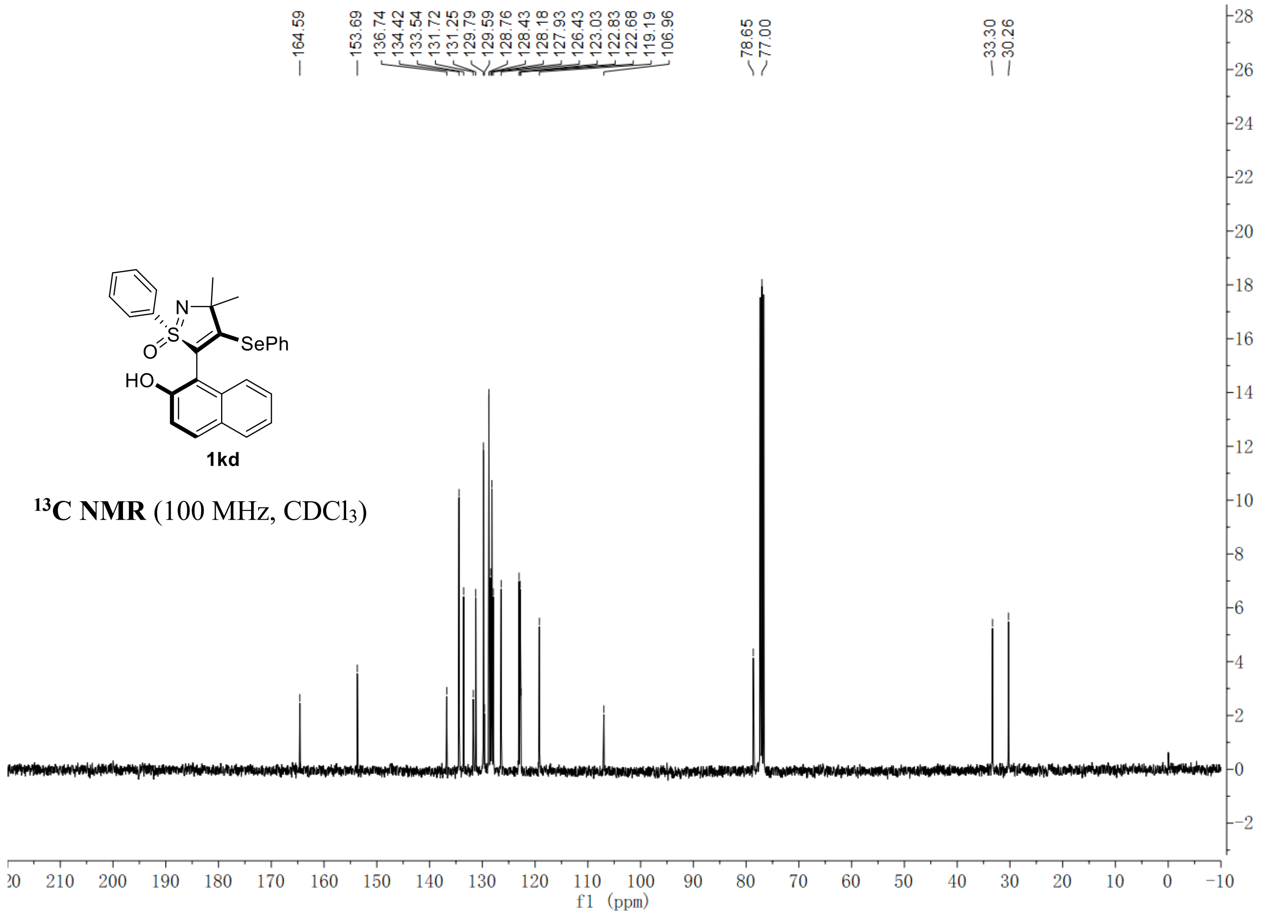
164.59
153.69 136.74 134.42 133.54 131.72 131.25 129.79 129.59 128.76 128.43 128.18 127.93 126.43 123.03 122.83 122.68 119.19 106.96
78.65 77.00
33.30 30.26
SePh
HO
N
S
O
1kd
^{13}C NMR (100 MHz, CDCl$_3$)
f1 (ppm)

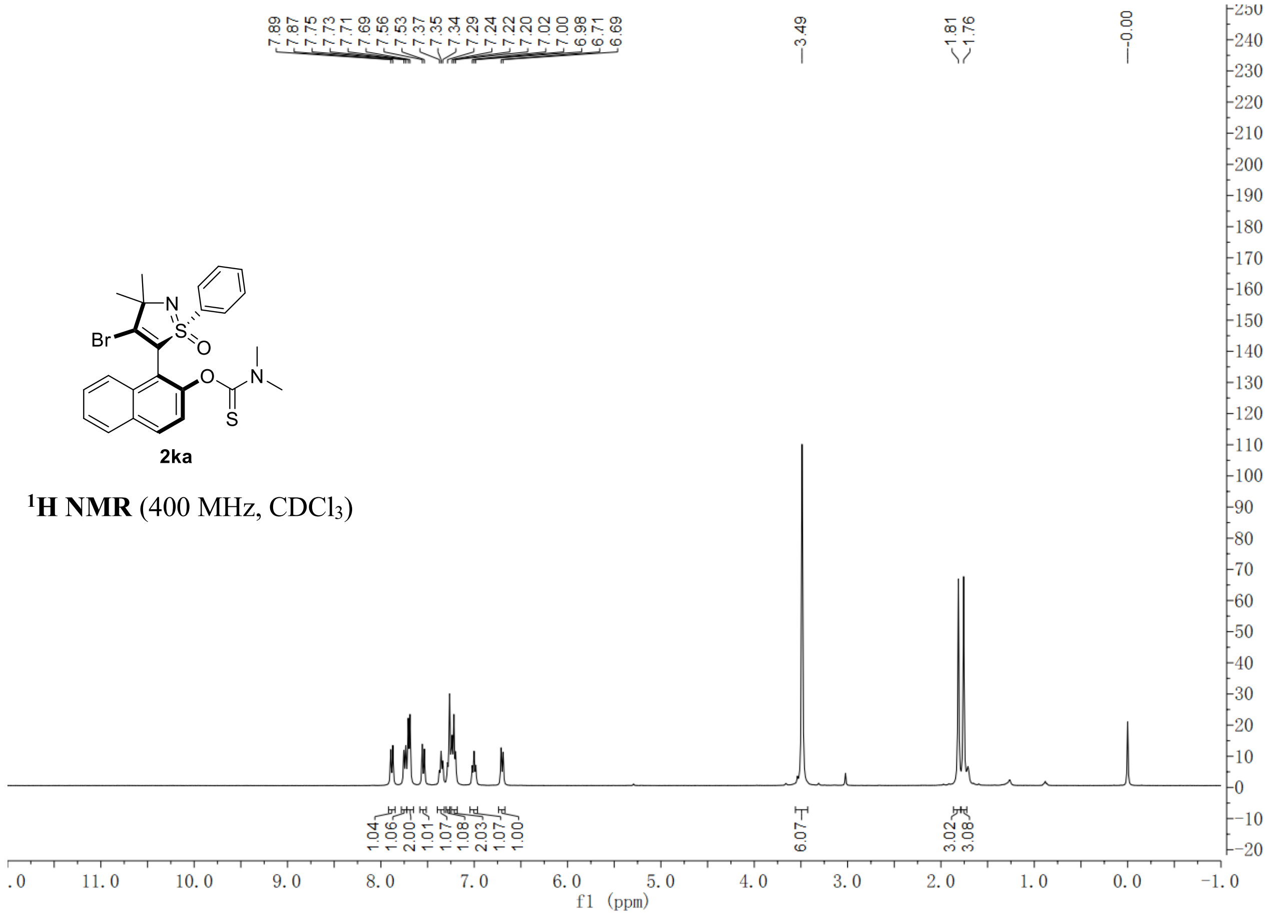
7.89
7.87
7.75
7.73
7.71
7.69
7.56
7.53
7.37
7.35
7.34
7.29
7.24
7.22
7.20
7.02
7.00
6.98
6.71
6.69
3.49
1.81
1.76
0.00
2ka
¹H NMR (400 MHz, CDCl3)
1.04
1.06
2.00
1.01
1.07
1.08
2.03
1.07
1.00
6.07
3.02
3.08
f1 (ppm)

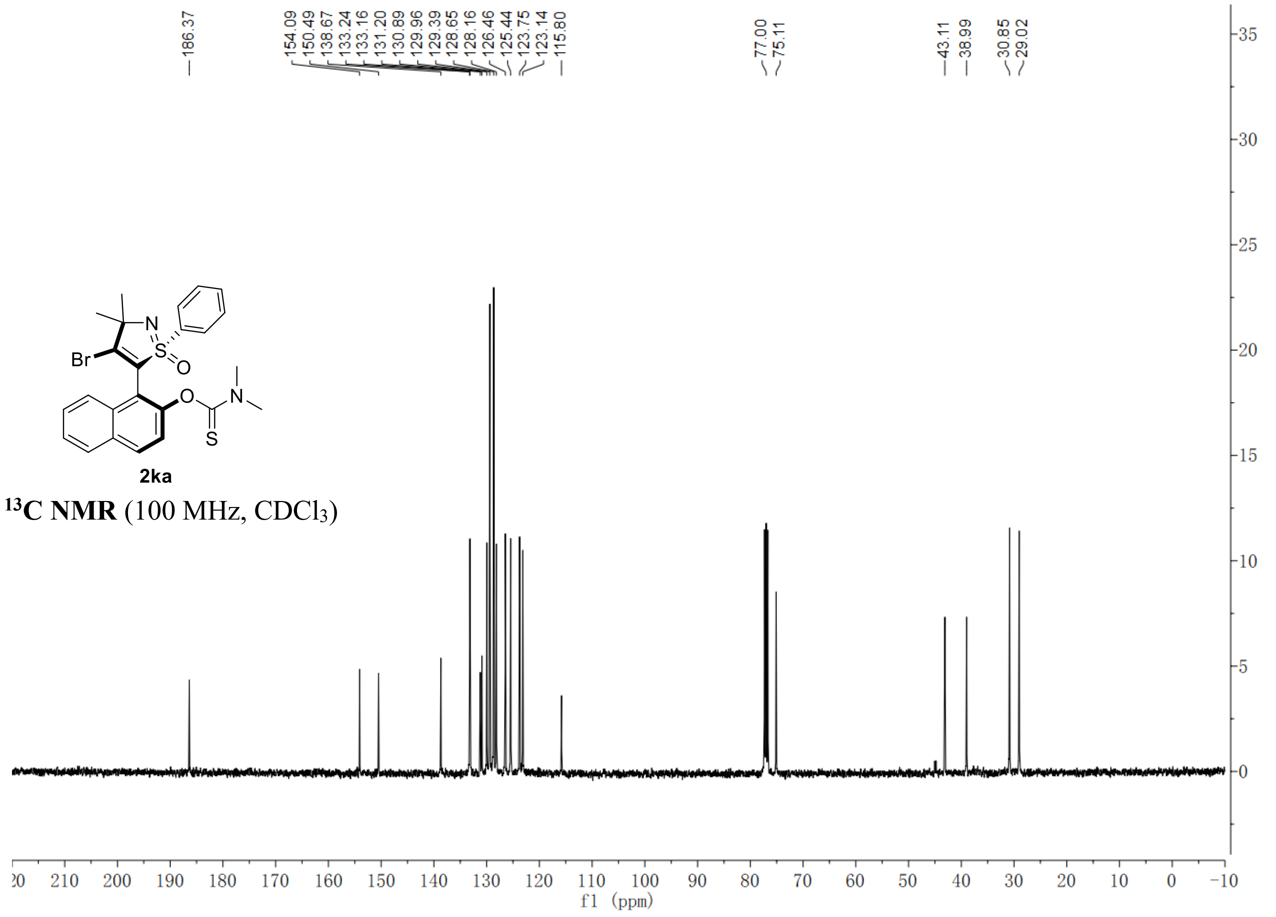
186.37
154.09
150.49
138.67
133.24
133.16
131.20
130.89
129.96
129.39
128.65
128.16
126.46
125.44
123.75
123.14
115.80
77.00
75.11
43.11
38.99
30.85
29.02
2ka
¹³C NMR (100 MHz, CDCl3)
f1 (ppm)

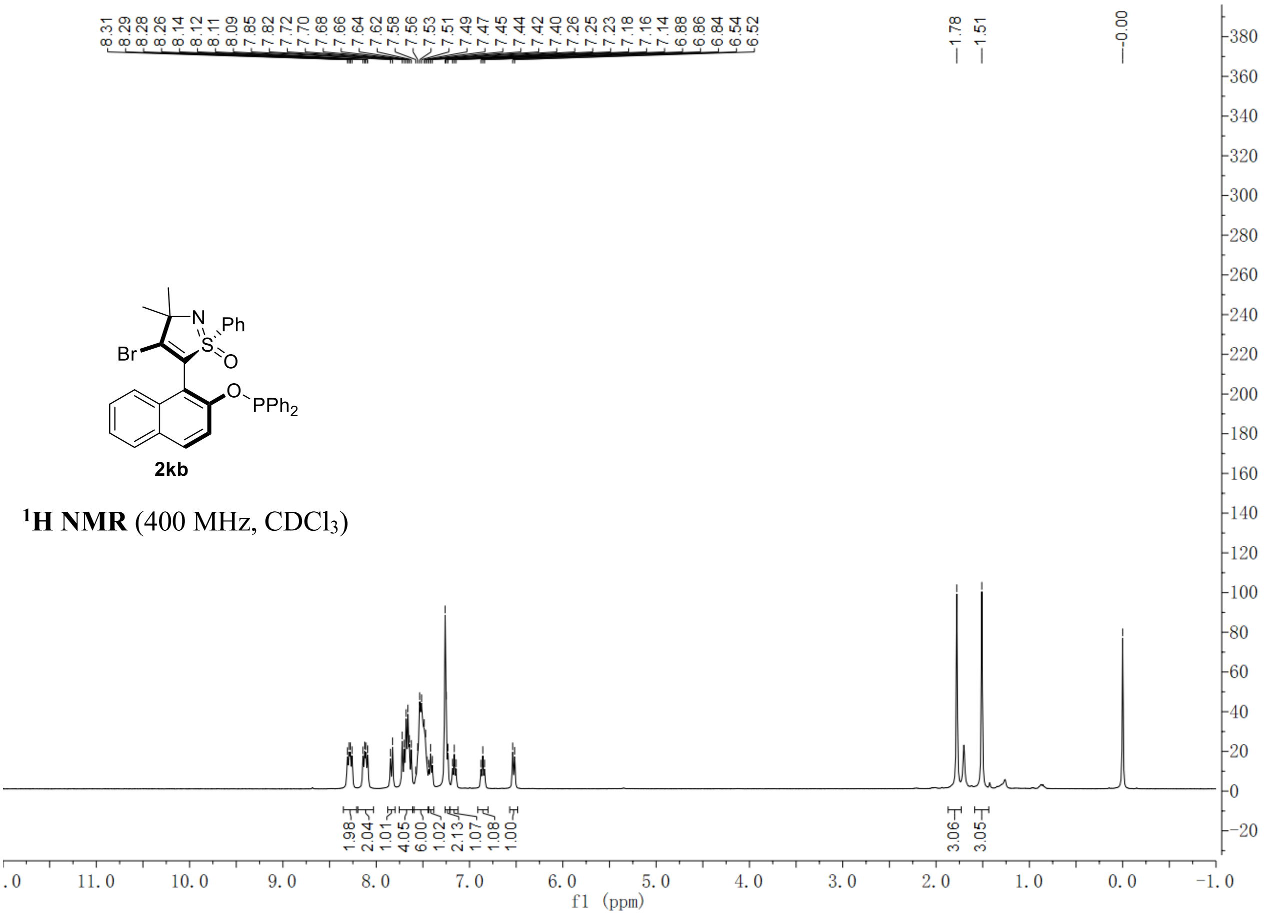

2kb

^{1}H NMR (400 MHz, $CDCl_3$)

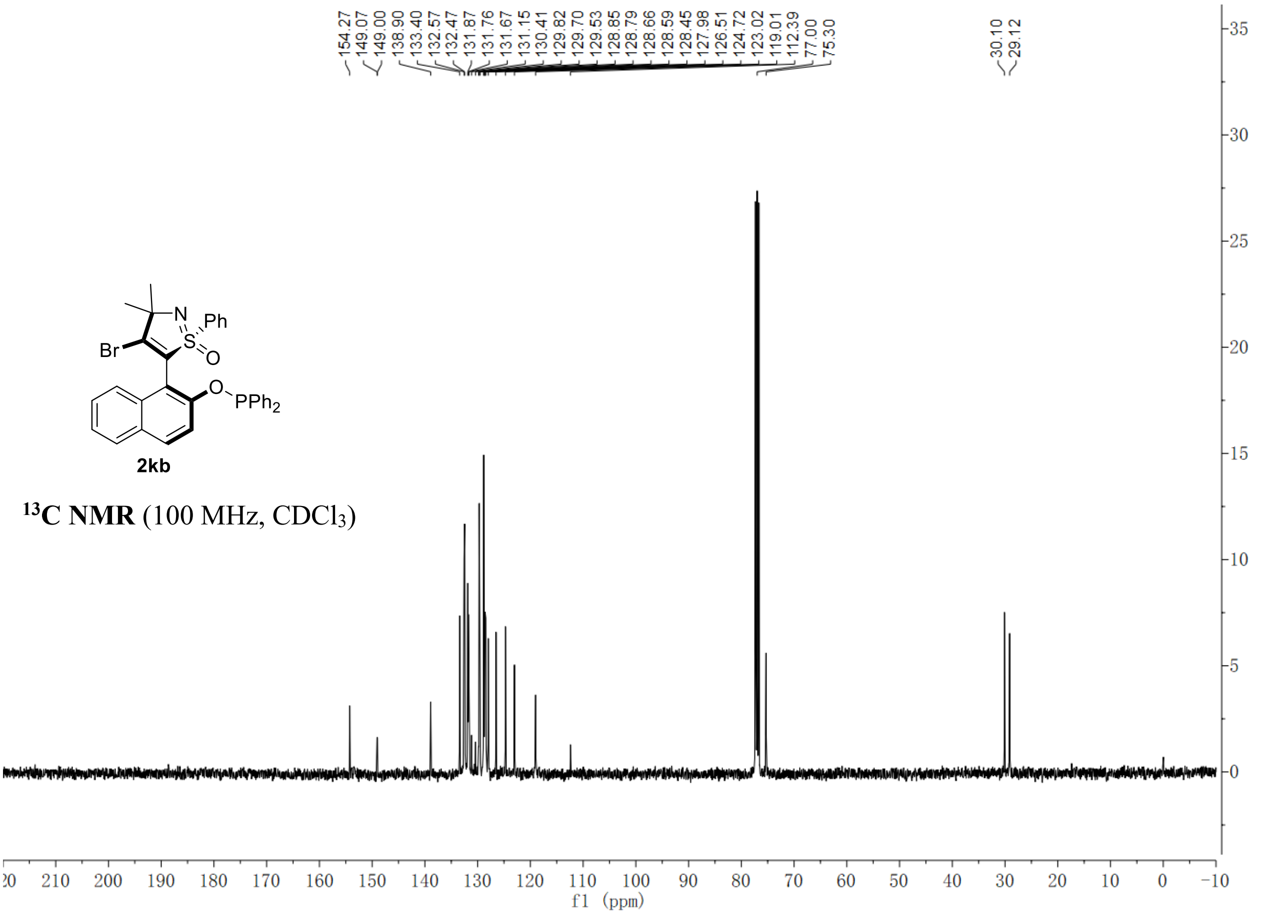

2kb

^{13}C NMR (100 MHz, $CDCl_3$)

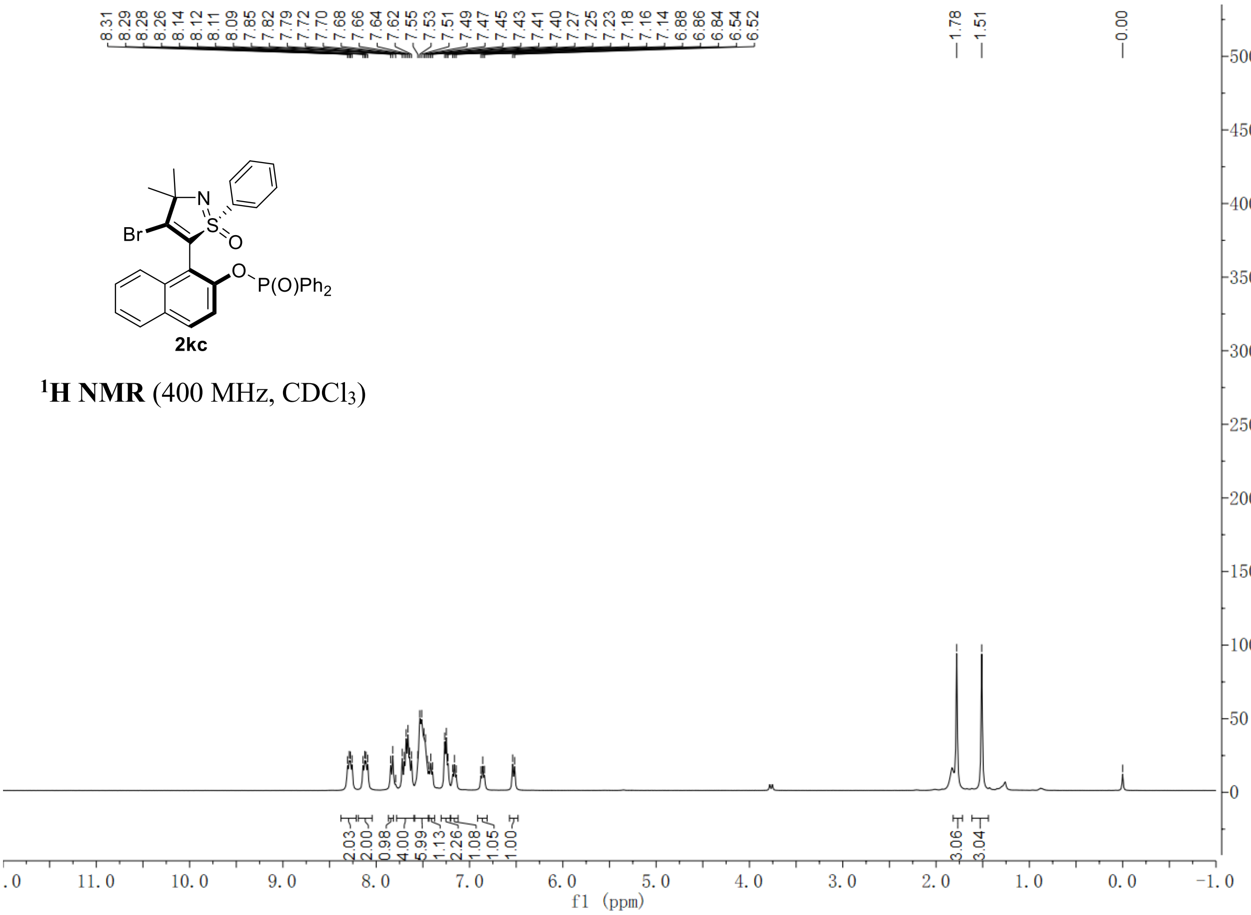
8.31 8.29 8.28 8.26 8.14 8.12 8.11 8.09 7.85 7.82 7.79 7.72 7.70 7.68 7.66 7.64 7.62 7.55 7.53 7.51 7.49 7.47 7.45 7.43 7.41 7.40 7.27 7.25 7.23 7.18 7.16 7.14 6.88 6.86 6.84 6.54 6.52
1.78
1.51
0.00
Br
N
S
O
O
P(O)Ph2
2kc
1H NMR (400 MHz, CDCl3)
2.03 2.00 0.98 4.00 5.99 1.13 2.26 1.08 1.05 1.00
3.06 3.04
f1 (ppm)

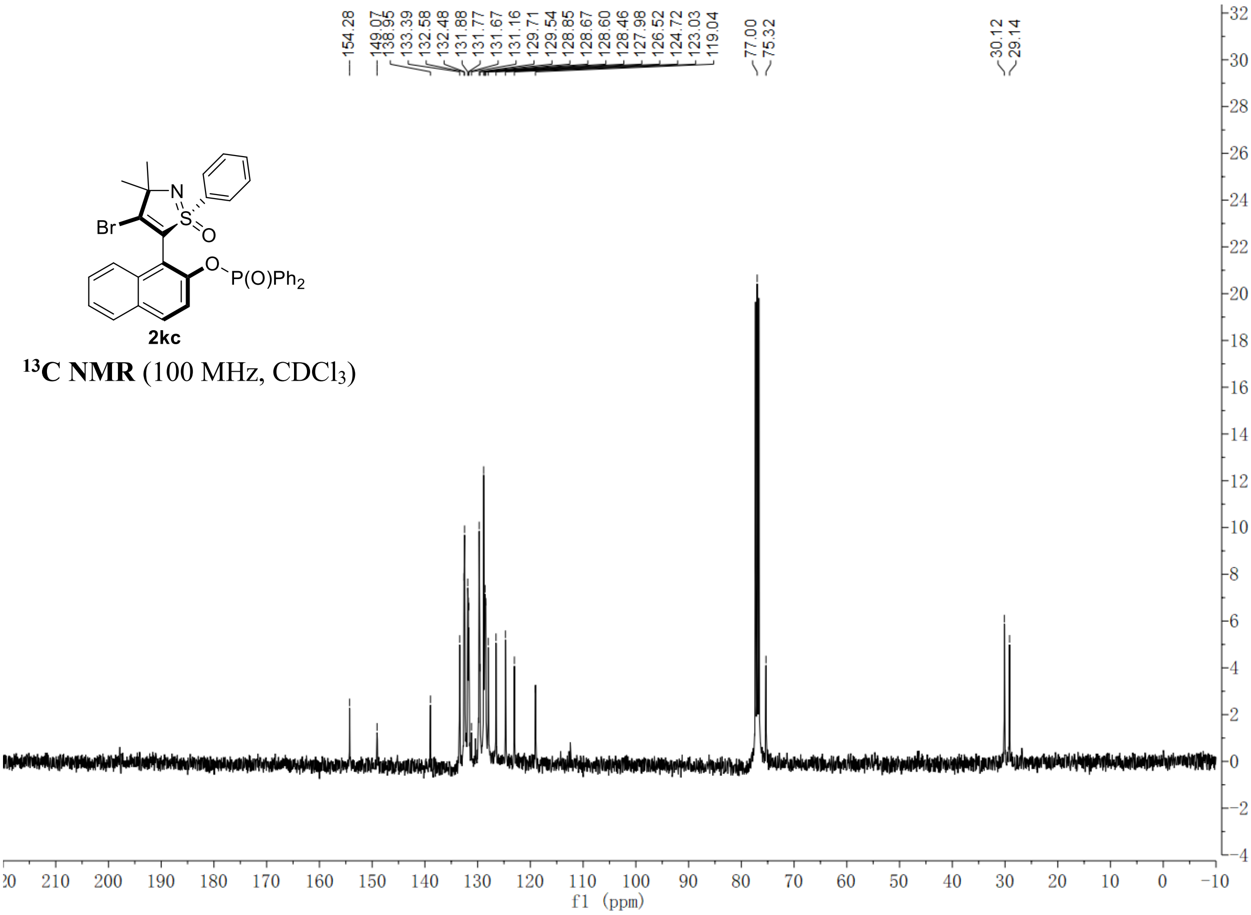
154.28
149.07 138.95 133.39 132.58 132.48 131.88 131.77 131.67 131.16 129.71 129.54 128.85 128.67 128.60 128.46 127.98 126.52 124.72 123.03 119.04
77.00 75.32
30.12 29.14
Br
N
S
O
O
P(O)Ph2
2kc
13C NMR (100 MHz, CDCl3)
f1 (ppm)

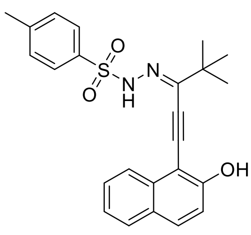

3a

^{1}H NMR (400 MHz, $CDCl_3$)

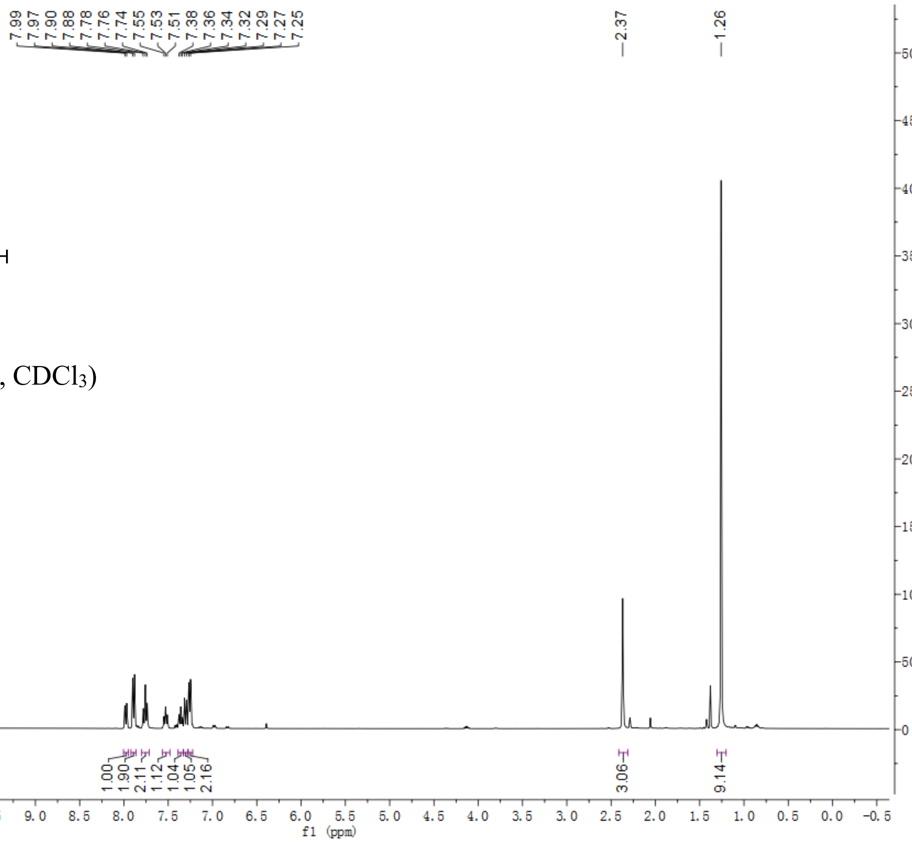

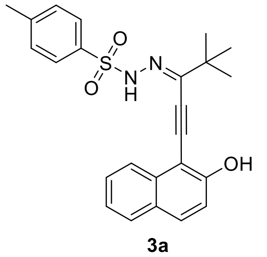

3a

^{13}C NMR (100 MHz, $CDCl_3$)

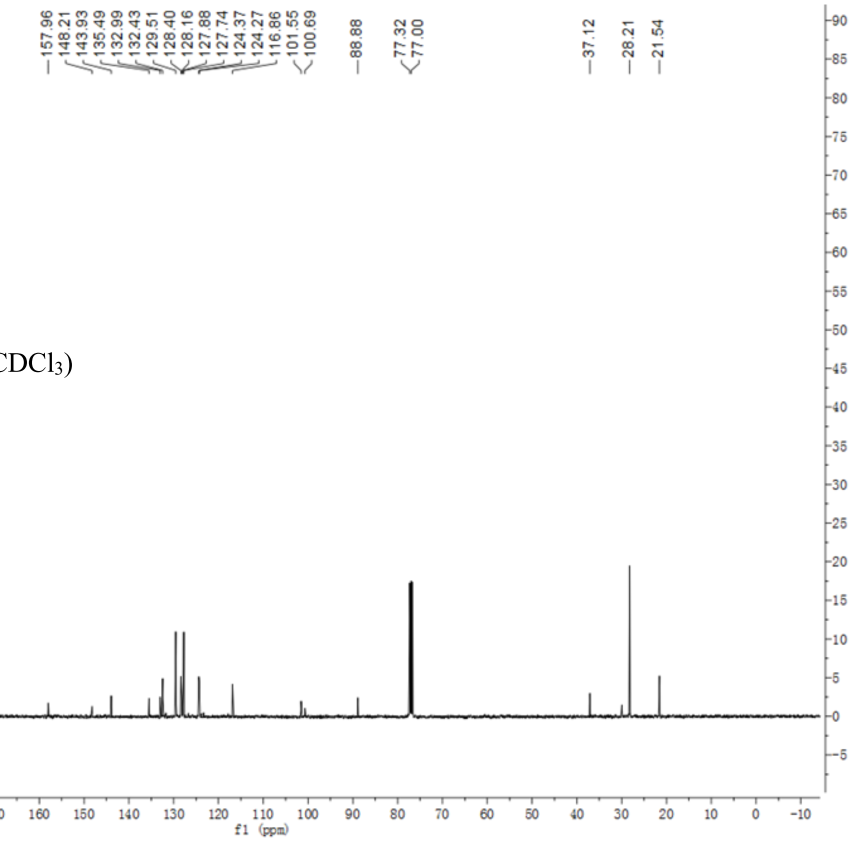

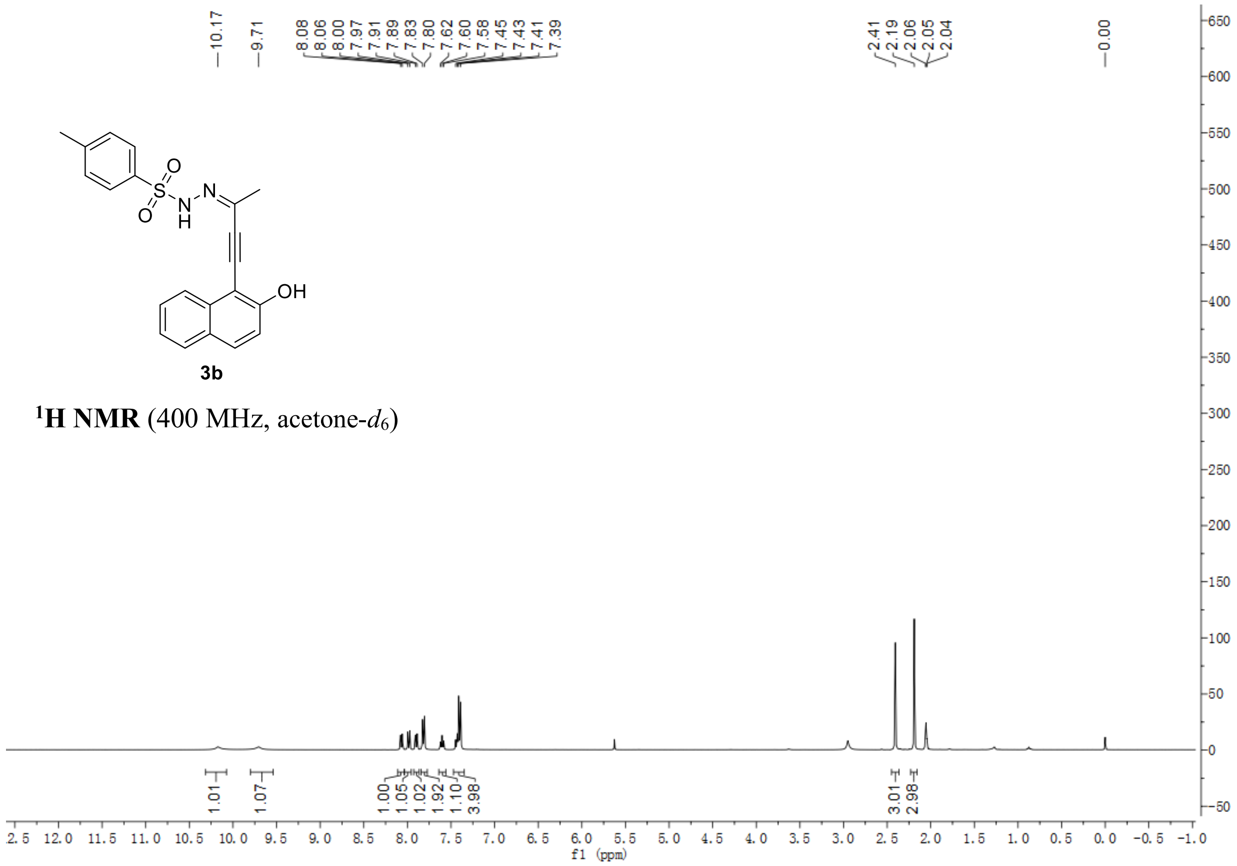
10.17
9.71
8.08 8.06 8.00 7.97 7.91 7.89 7.83 7.80 7.62 7.60 7.58 7.45 7.43 7.41 7.39
2.41 2.19 2.06 2.05 2.04
0.00
3b
^{1}H NMR (400 MHz, acetone-d_6)
1.01 1.07 1.00 1.05 1.02 1.92 1.10 3.98 3.01 2.98
f1 (ppm)

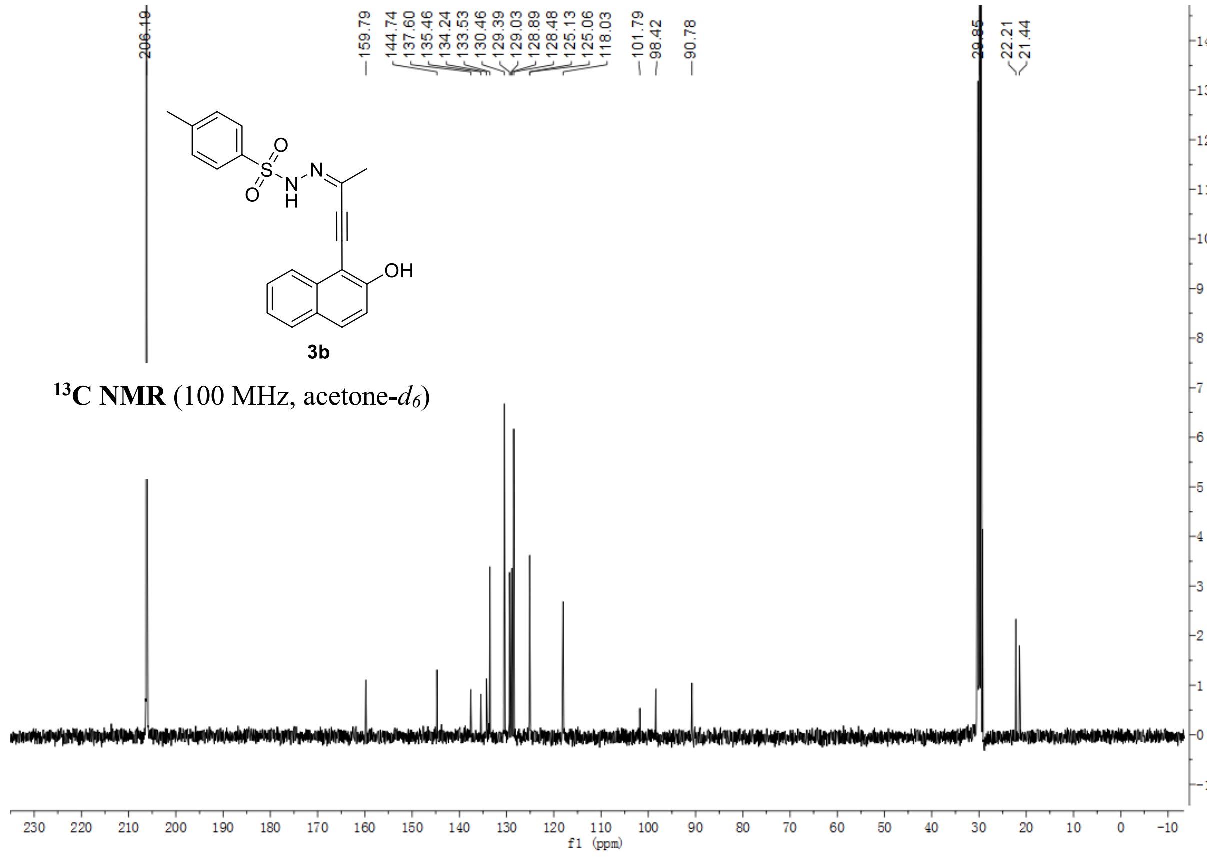
206.19
159.79 144.74 137.60 135.46 134.24 133.53 130.46 129.39 129.03 128.89 128.48 125.13 125.06 118.03 101.79 98.42 90.78
29.85 22.21 21.44
3b
^{13}C NMR (100 MHz, acetone-d_6)
f1 (ppm)

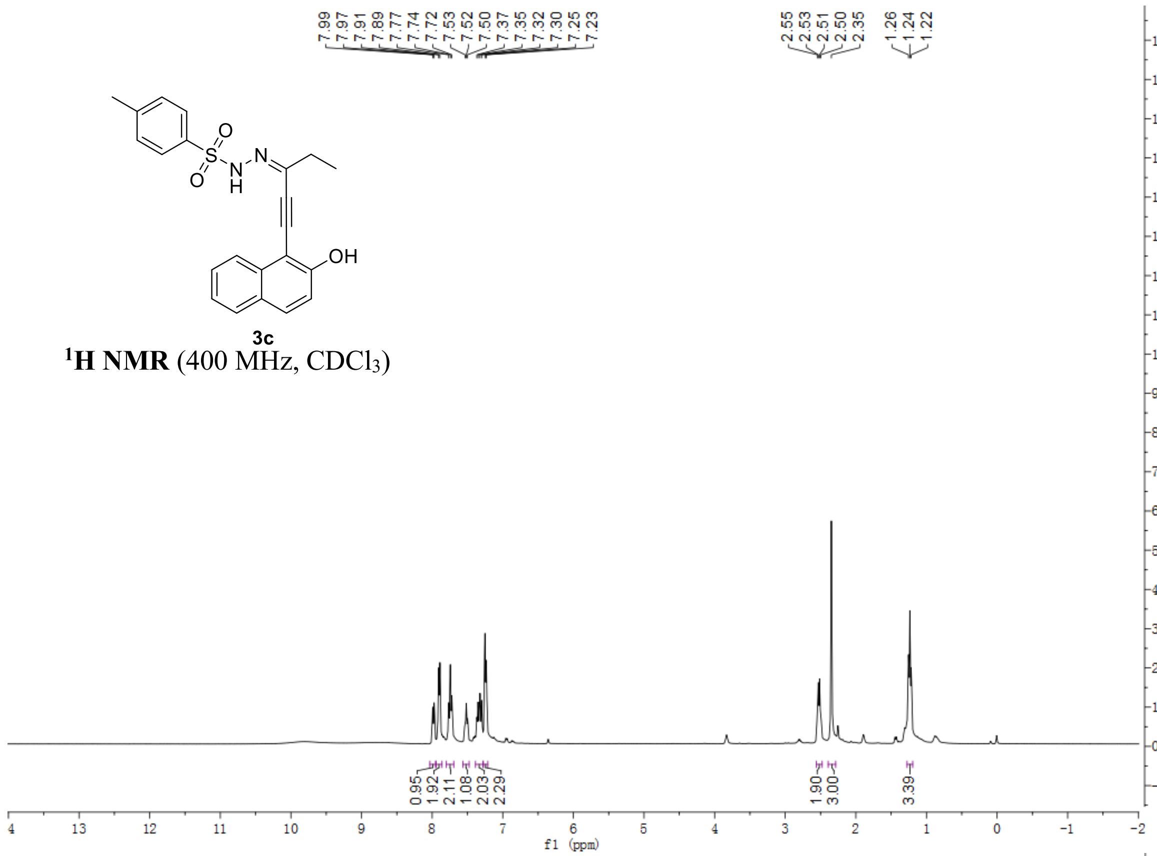

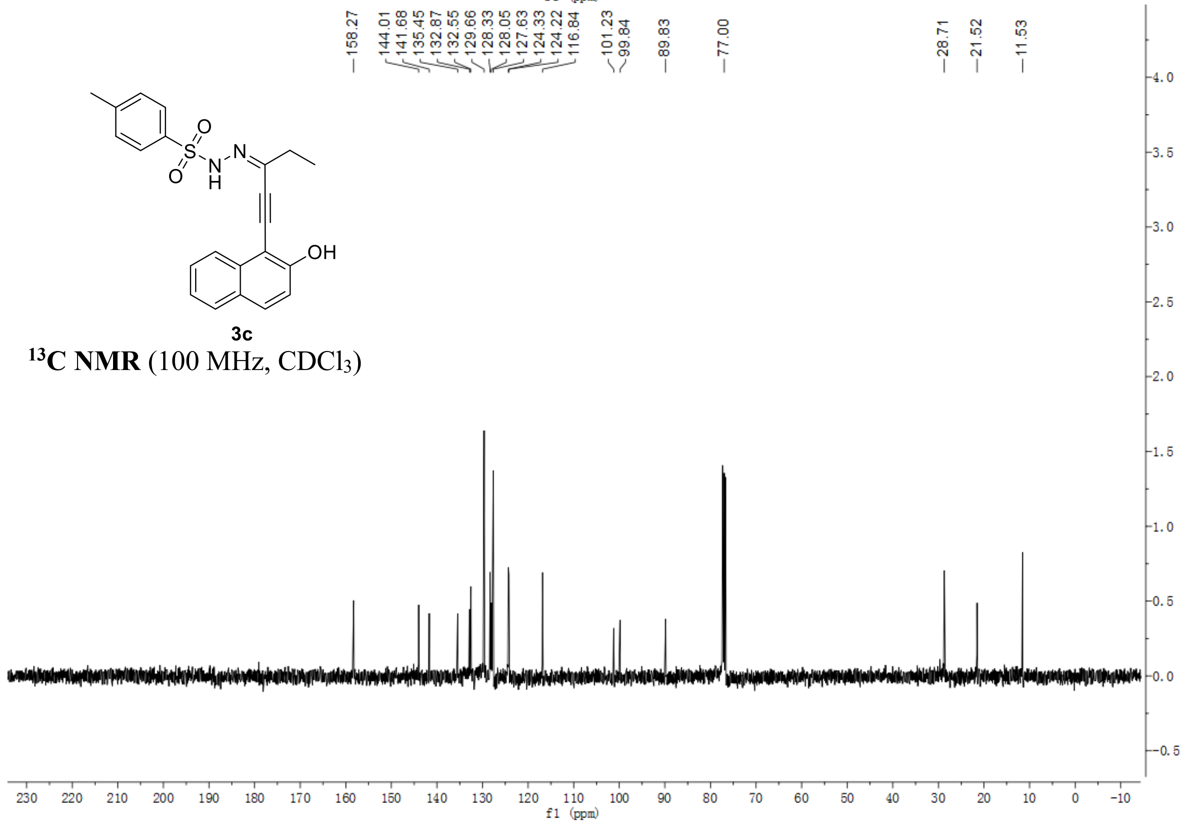

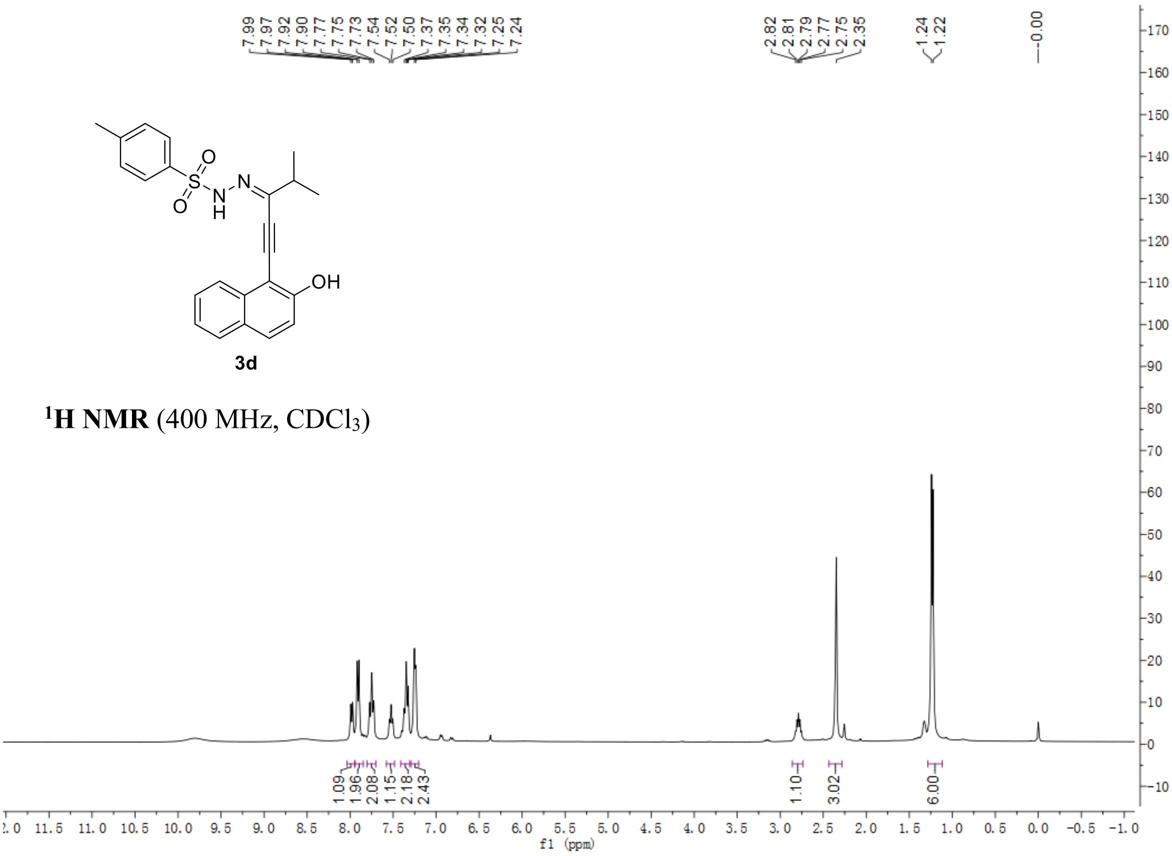

3d

^{1}H NMR (400 MHz, $CDCl_3$)

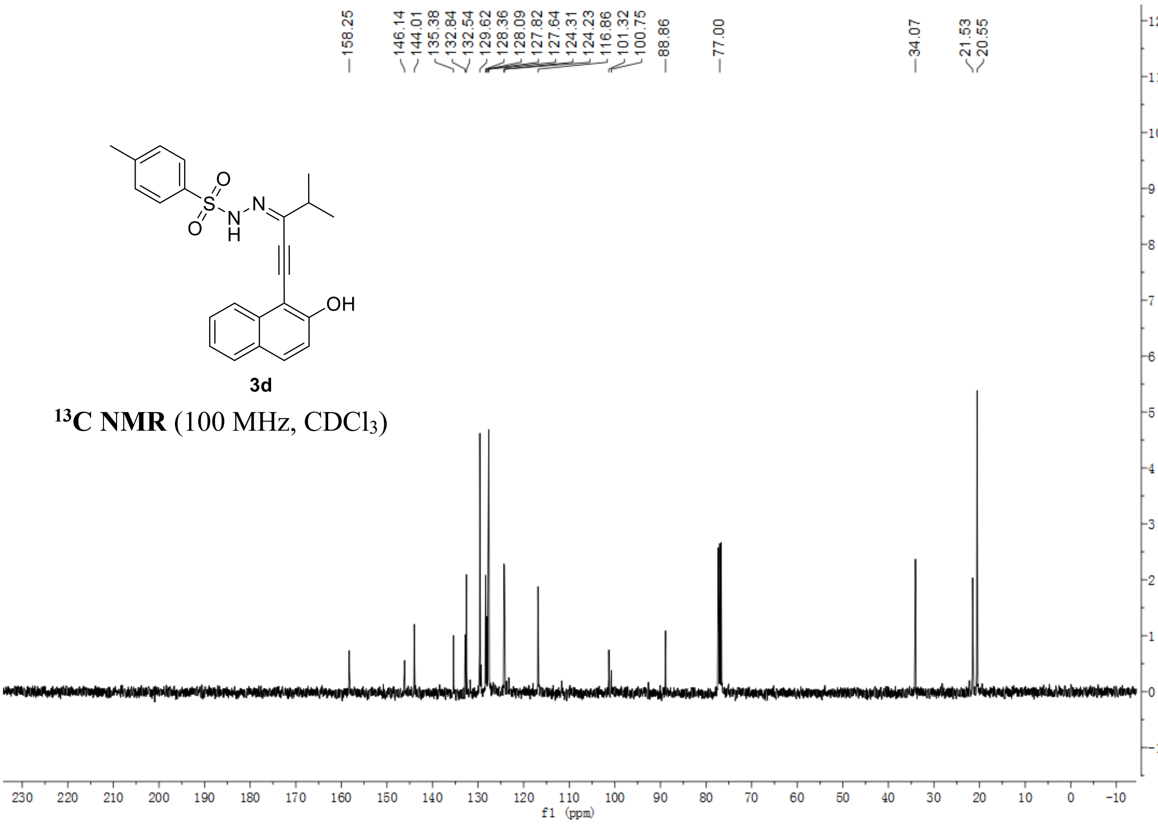

3d

^{13}C NMR (100 MHz, $CDCl_3$)

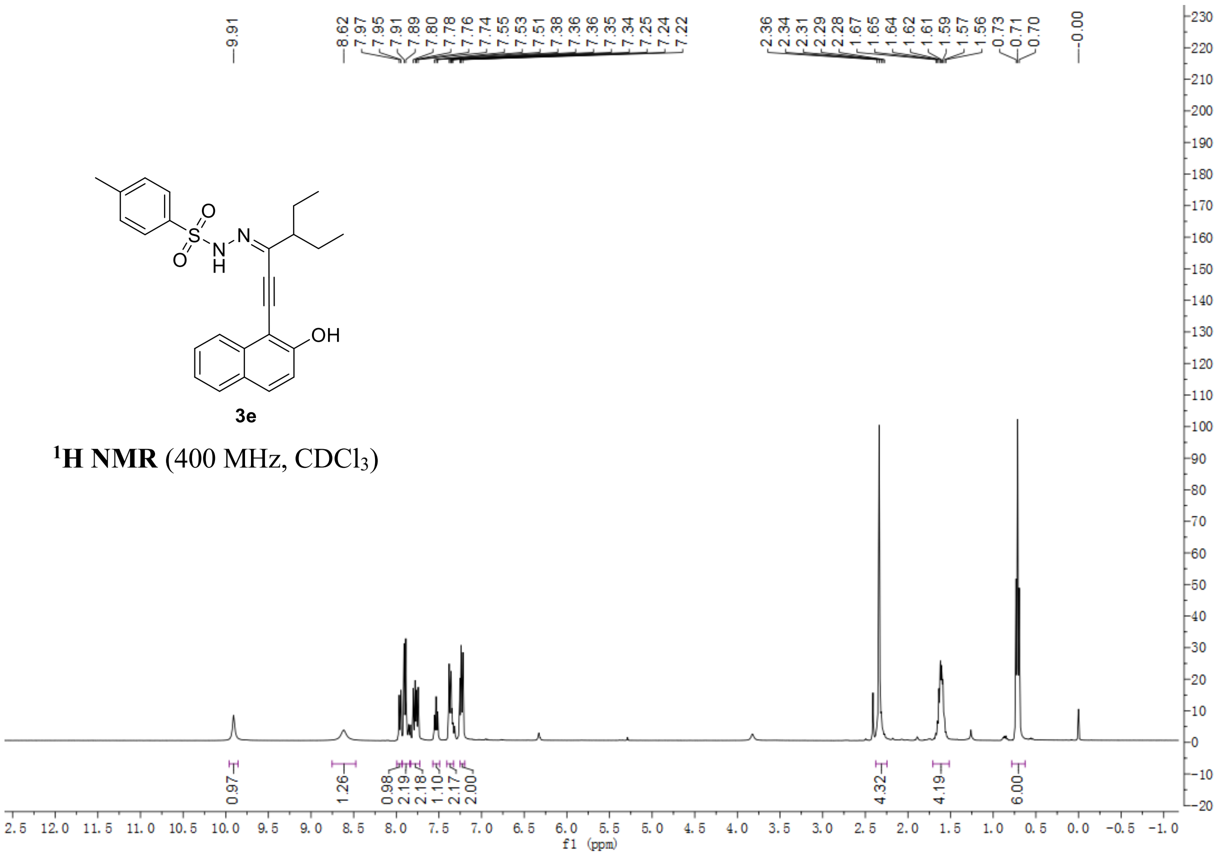

3e

^{1}H NMR (400 MHz, $CDCl_3$)

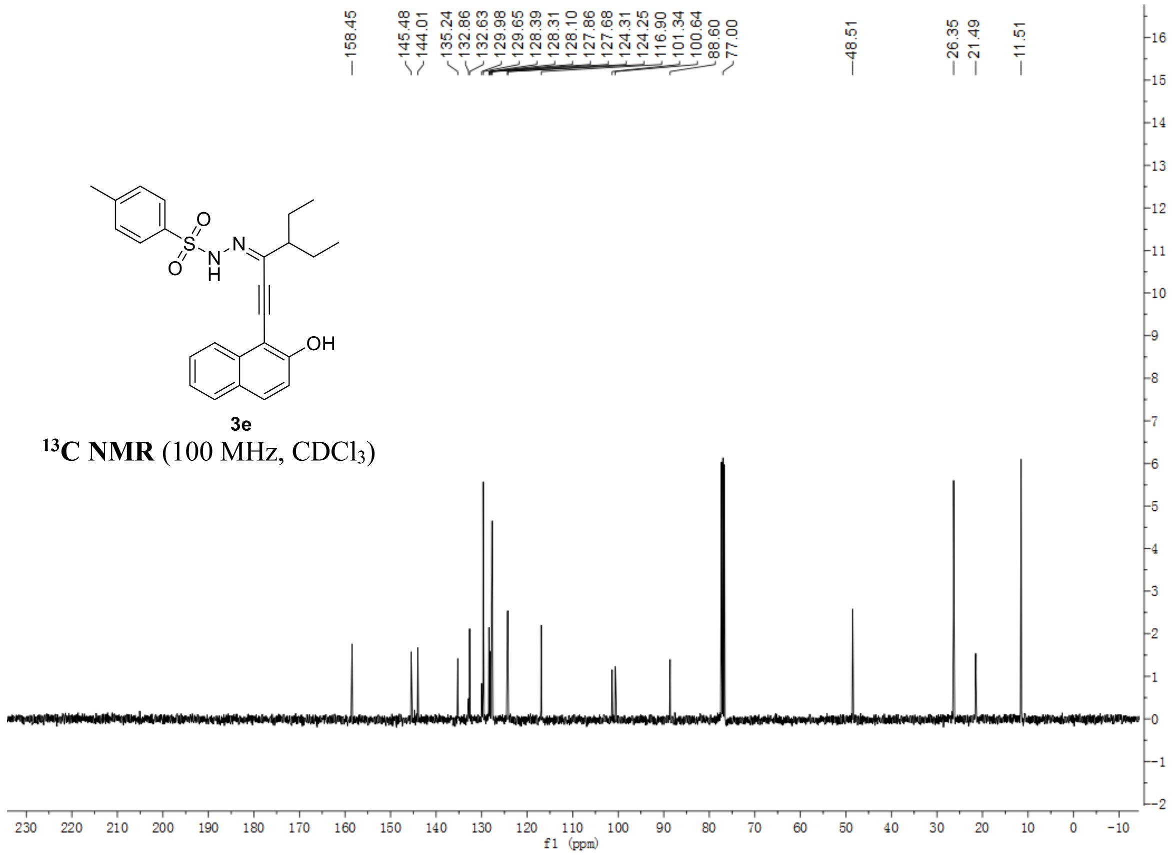

3e

^{13}C NMR (100 MHz, $CDCl_3$)

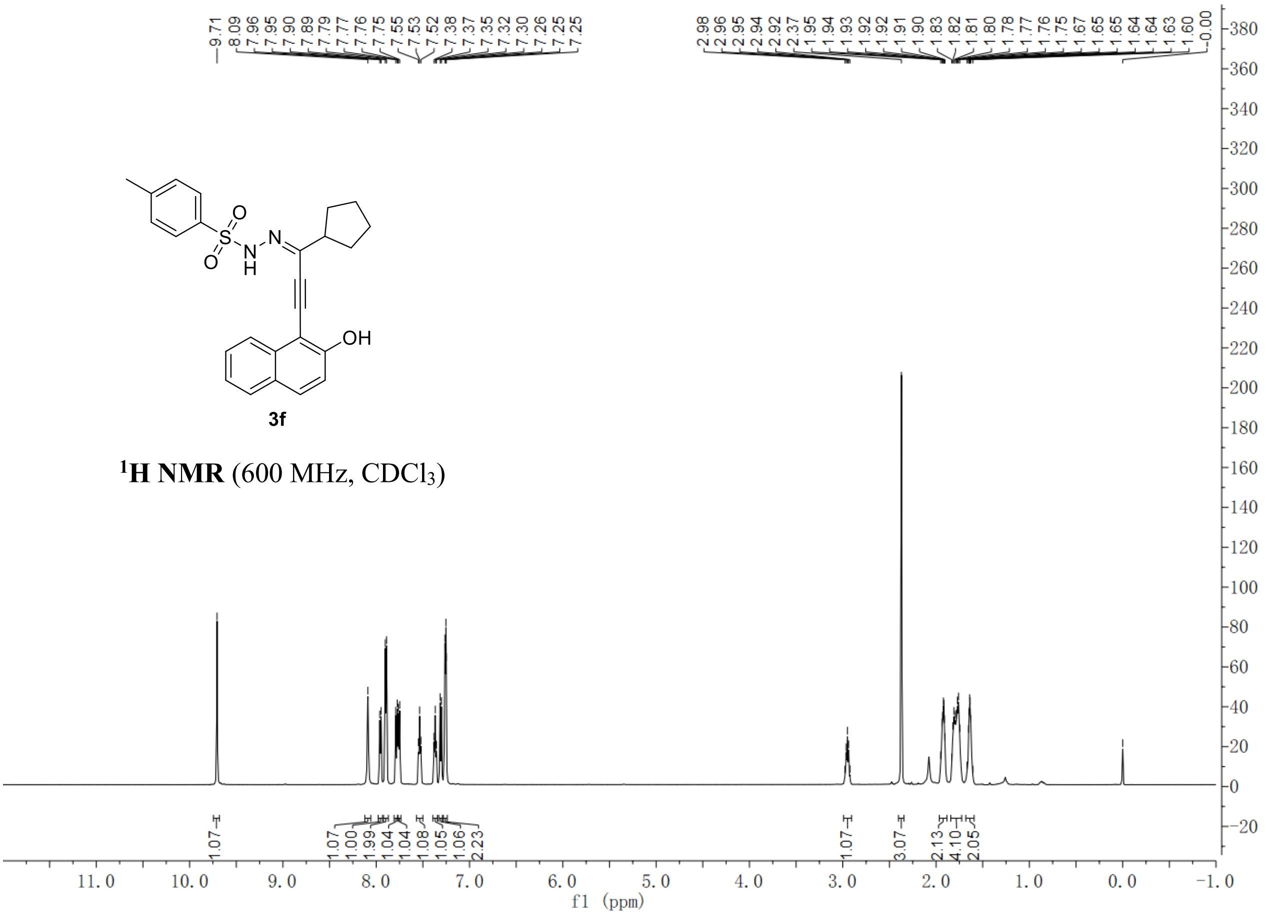

3f

¹H NMR (600 MHz, CDCl$_3$)

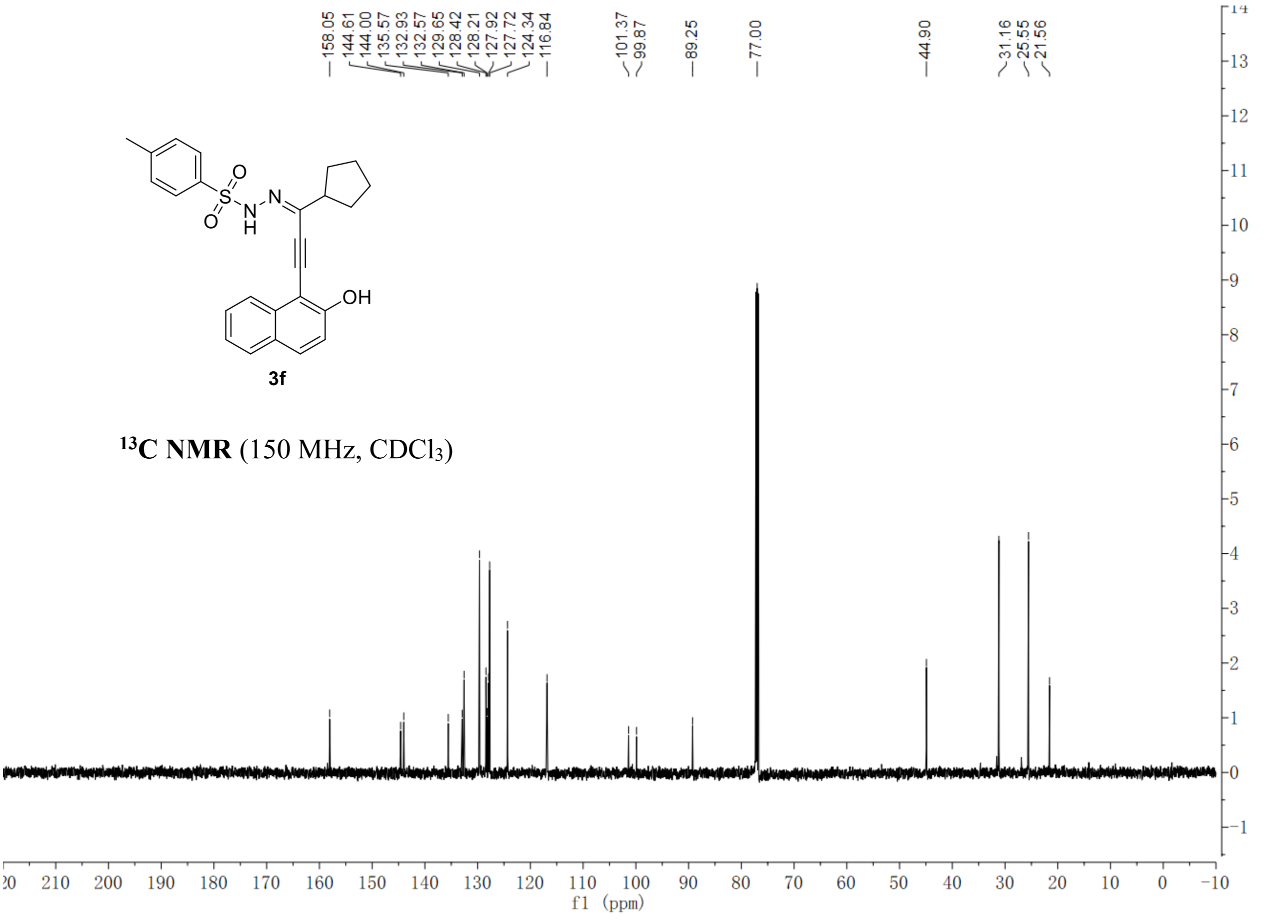

3f

¹³C NMR (150 MHz, CDCl$_3$)

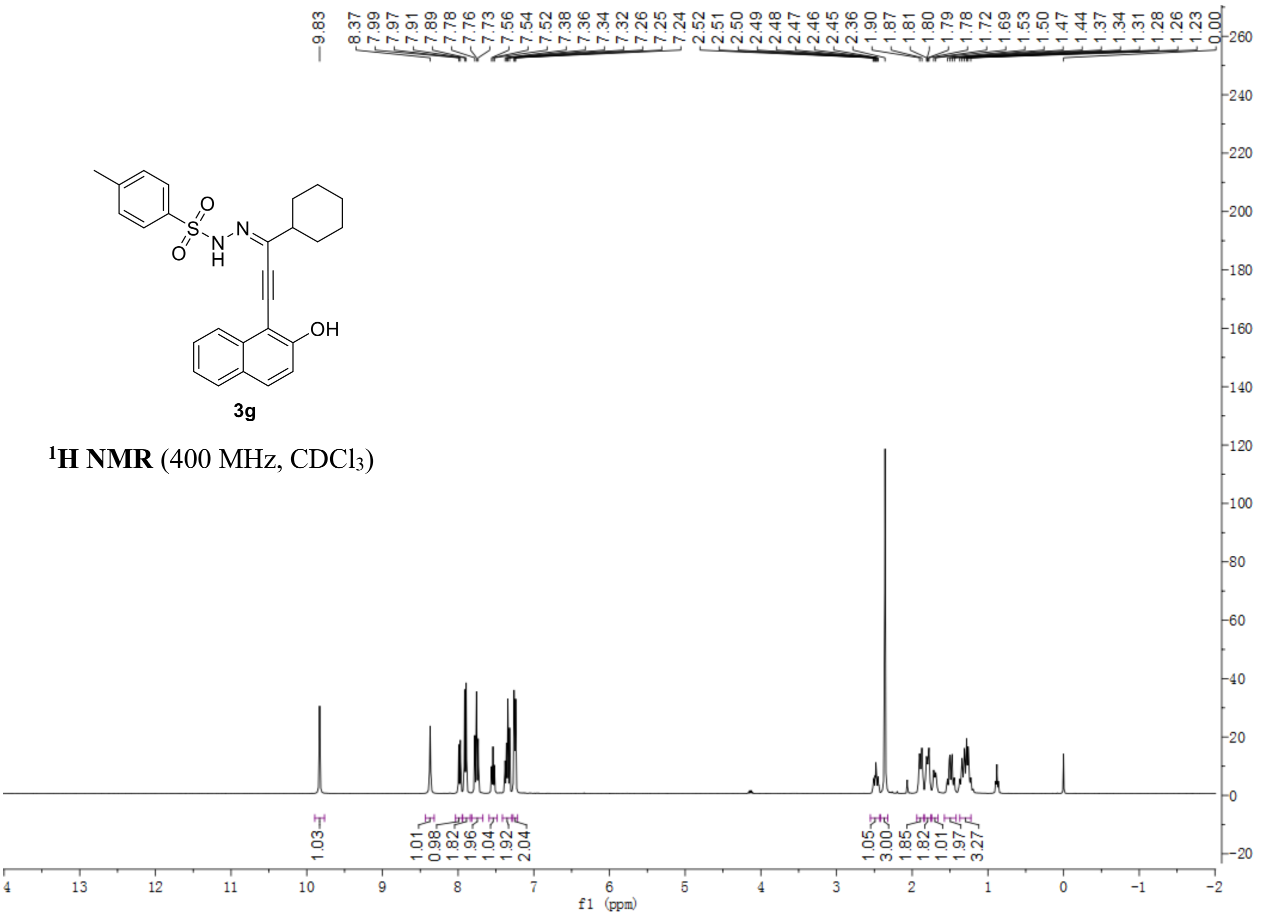
3g
¹H NMR (400 MHz, CDCl3)
f1 (ppm)

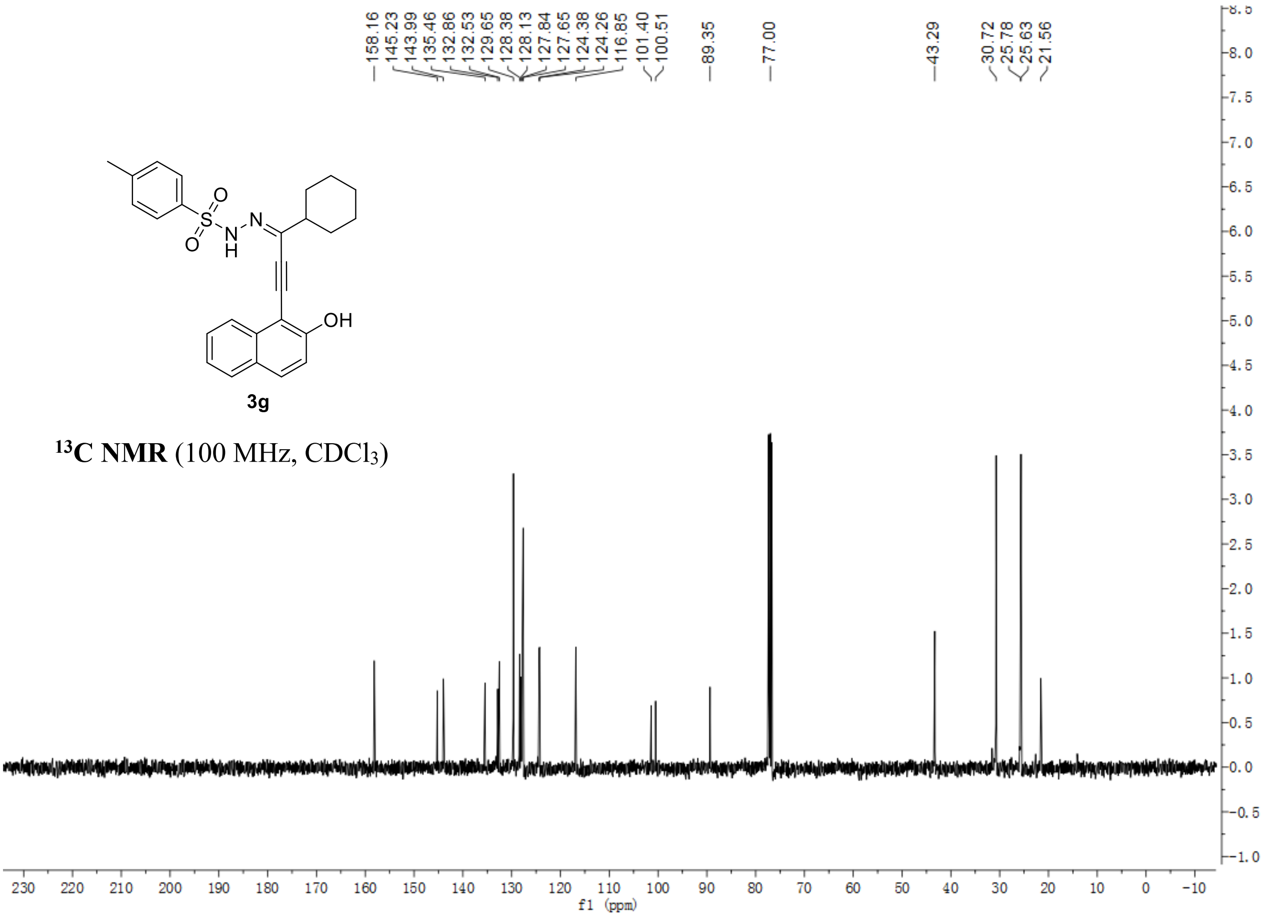
3g
¹³C NMR (100 MHz, CDCl3)
f1 (ppm)

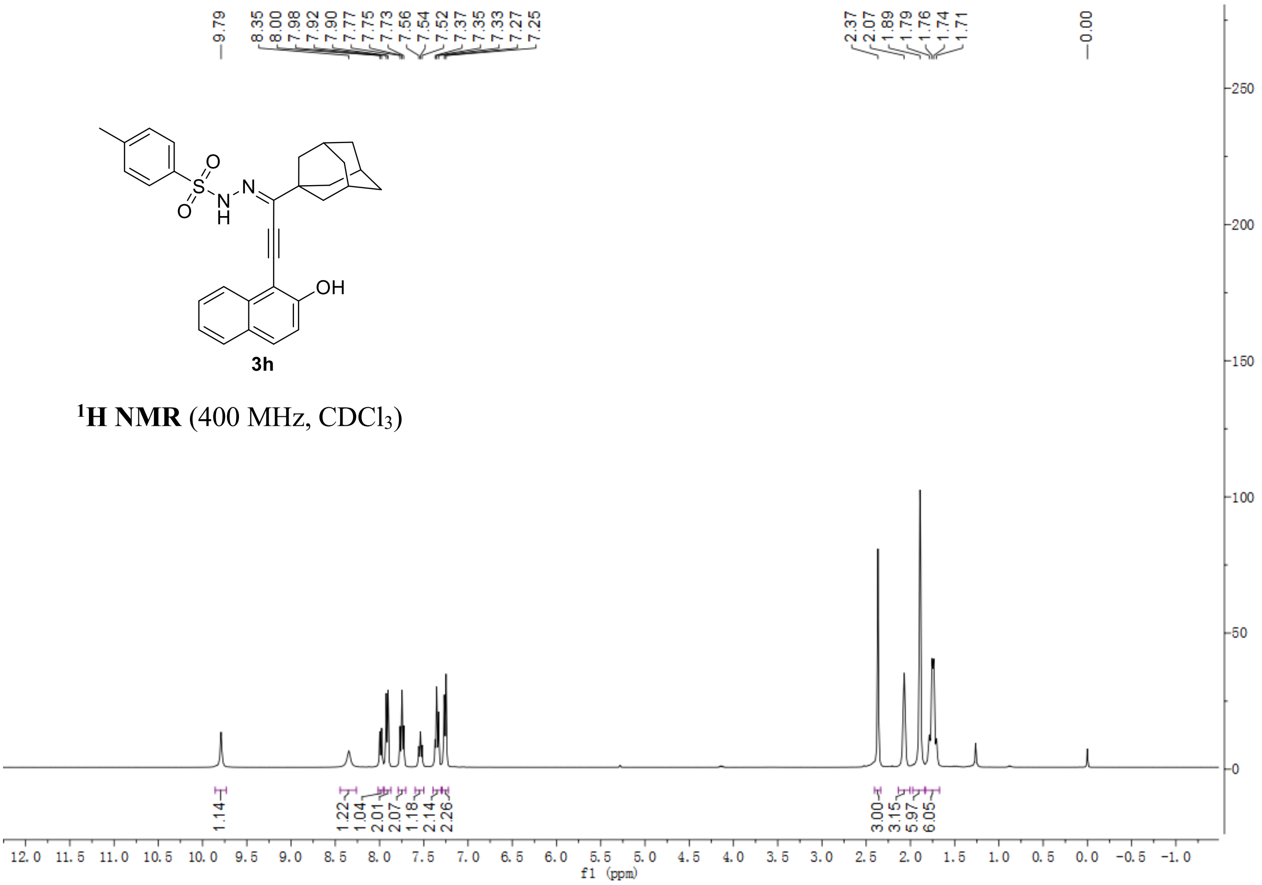

^{1}H NMR (400 MHz, $CDCl_3$)

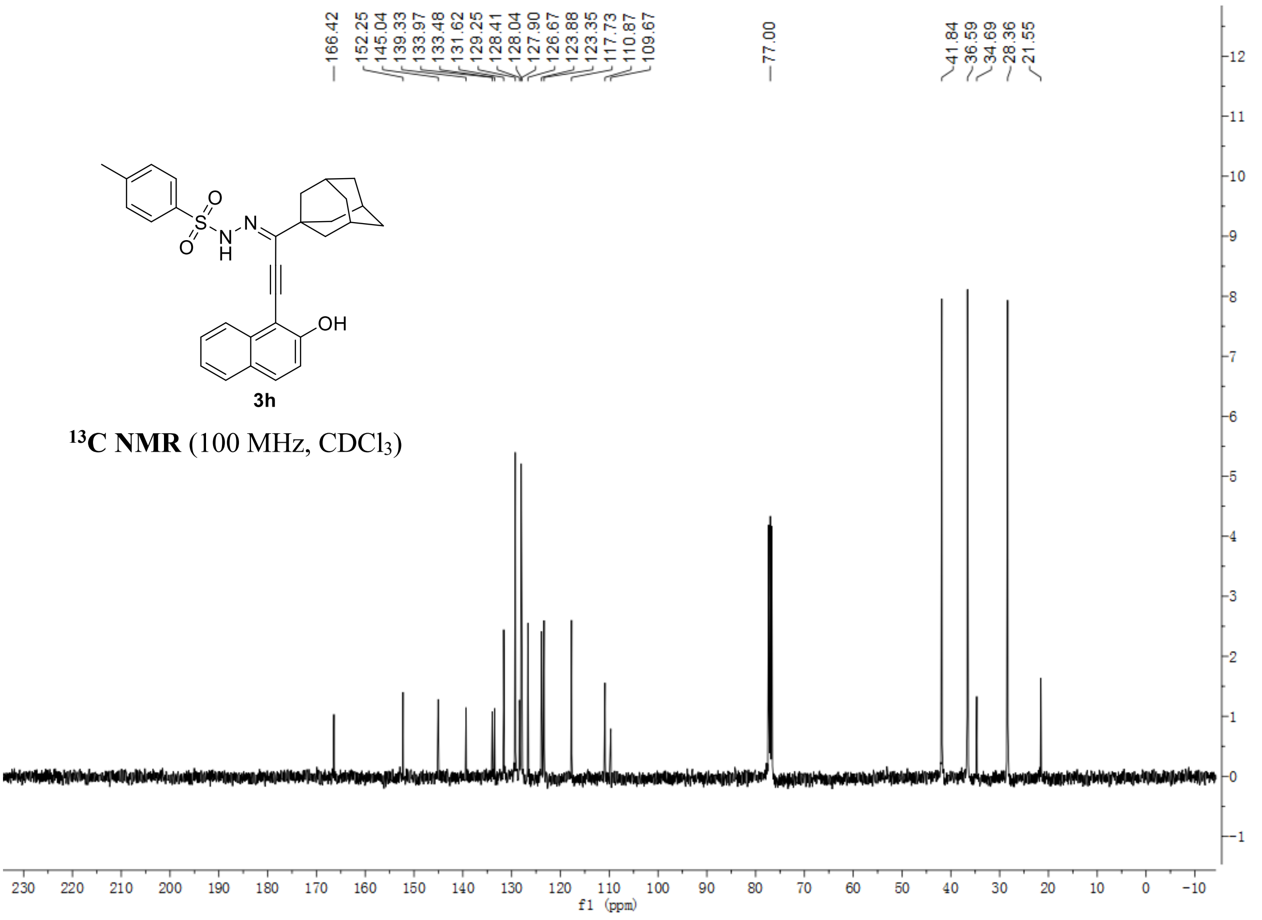

^{13}C NMR (100 MHz, $CDCl_3$)

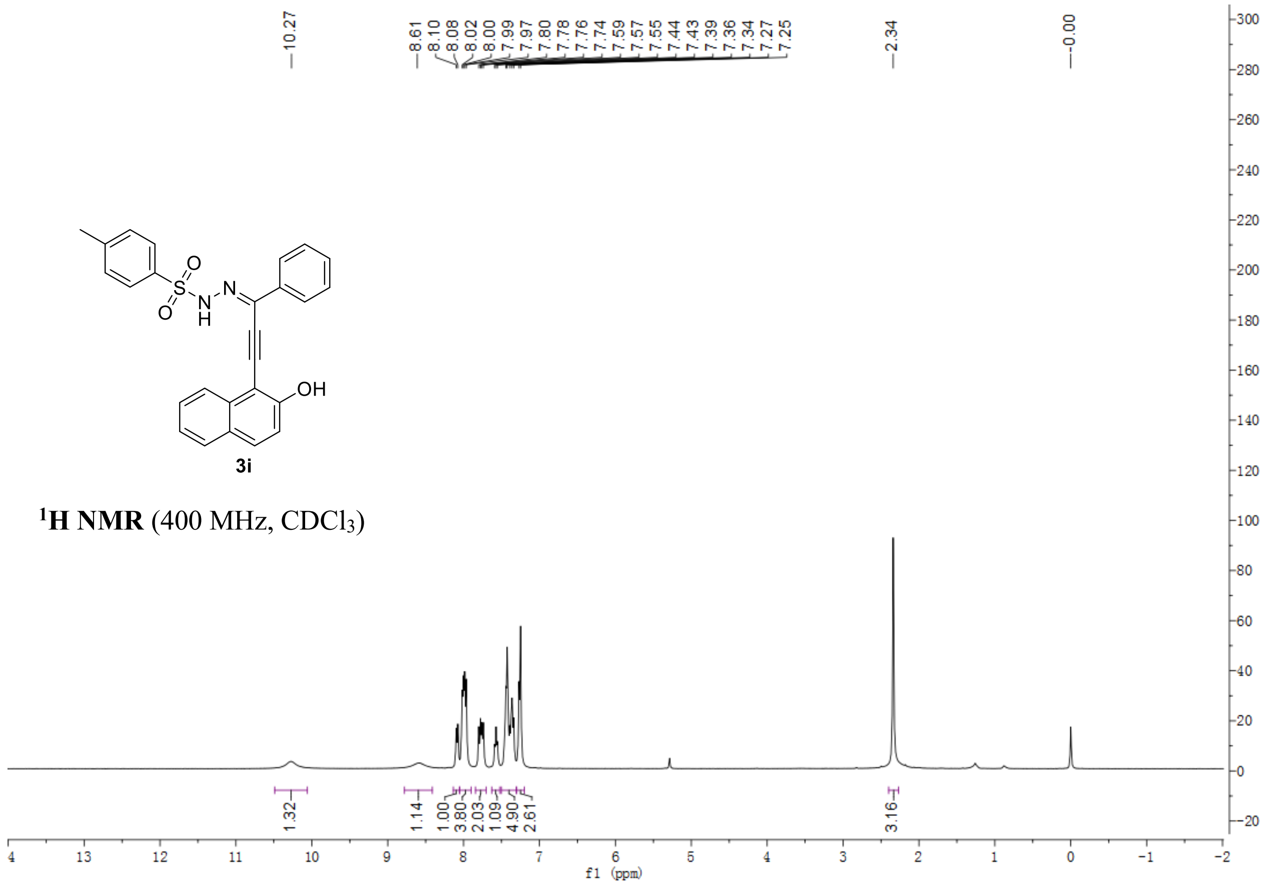

3i

^{1}H NMR (400 MHz, $CDCl_3$)

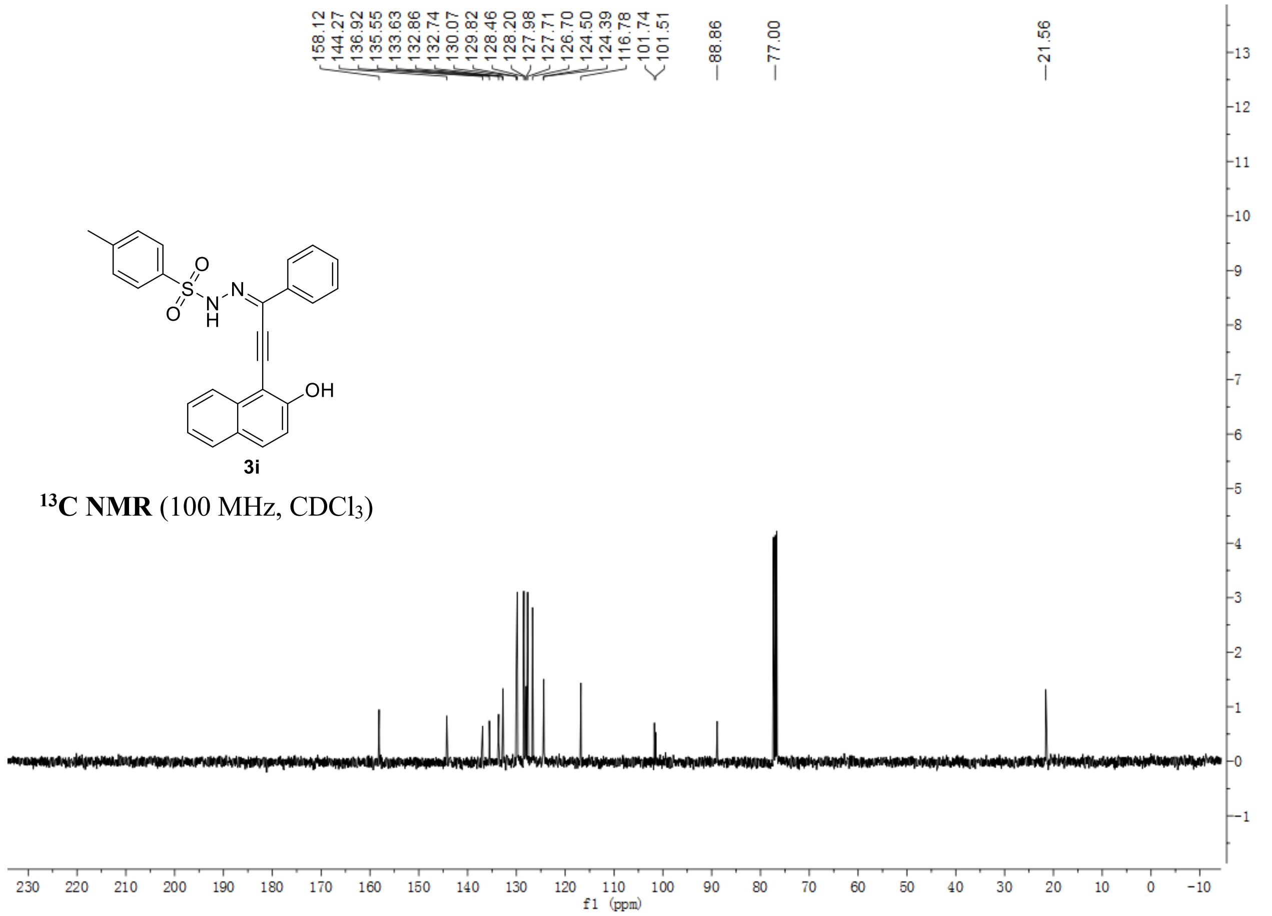

3i

^{13}C NMR (100 MHz, $CDCl_3$)

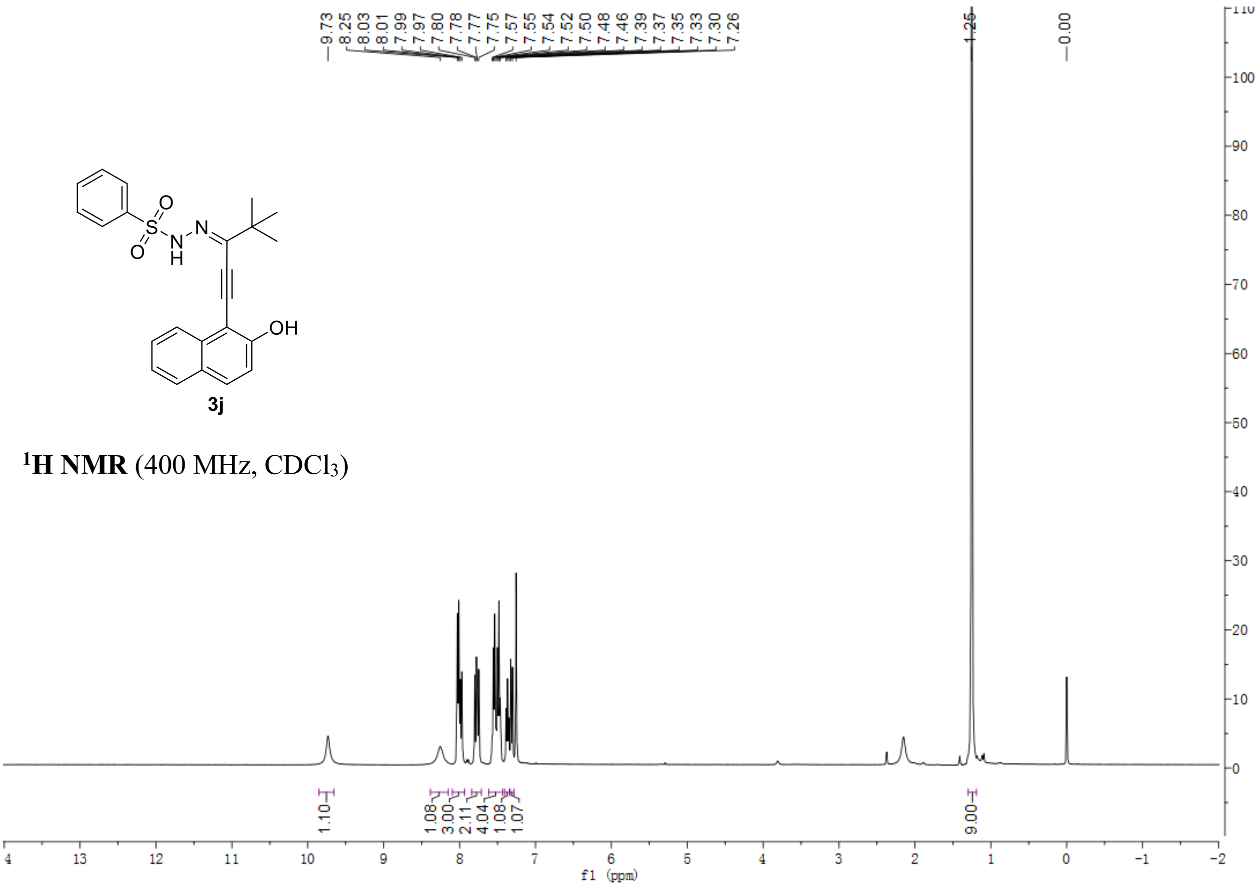

^{1}H NMR (400 MHz, $CDCl_3$)

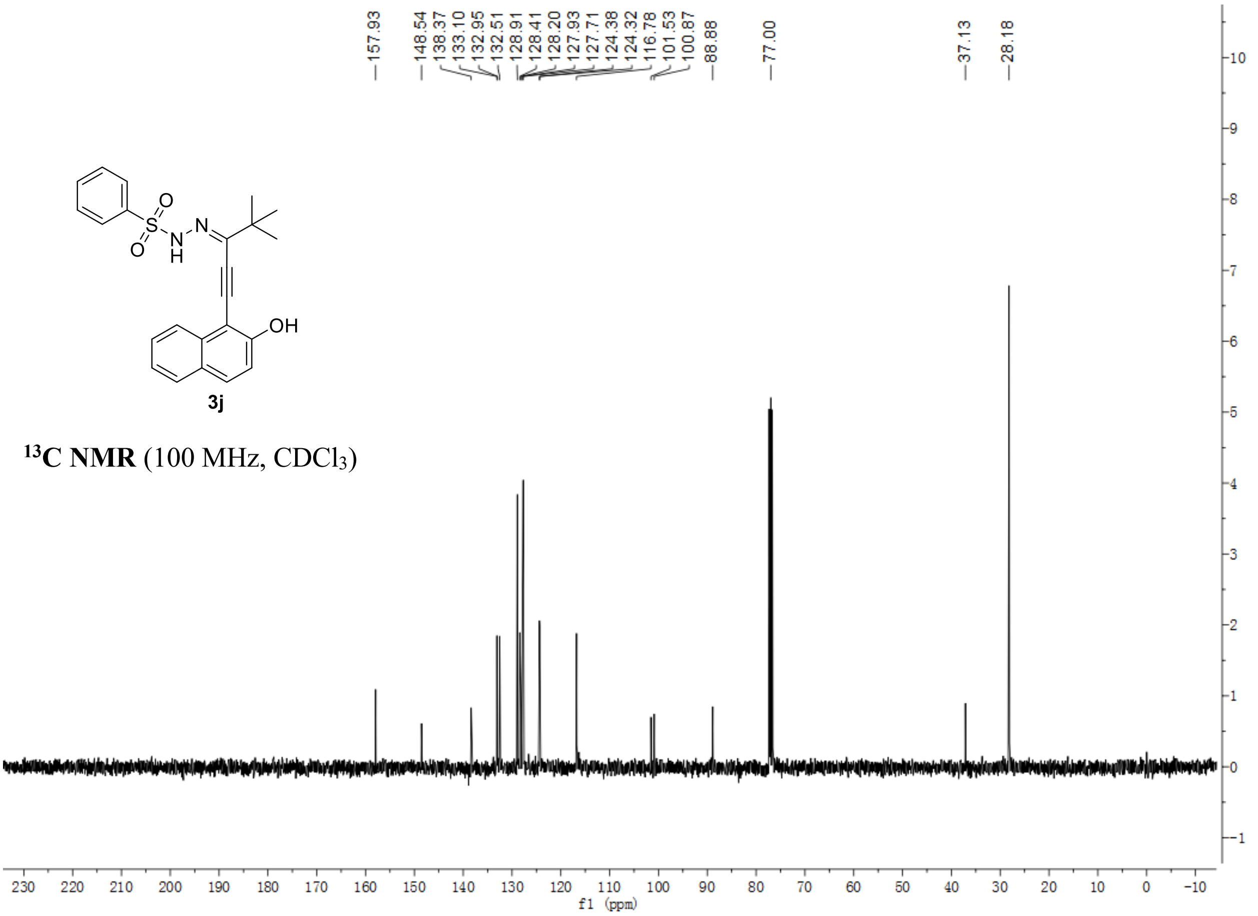

^{13}C NMR (100 MHz, $CDCl_3$)

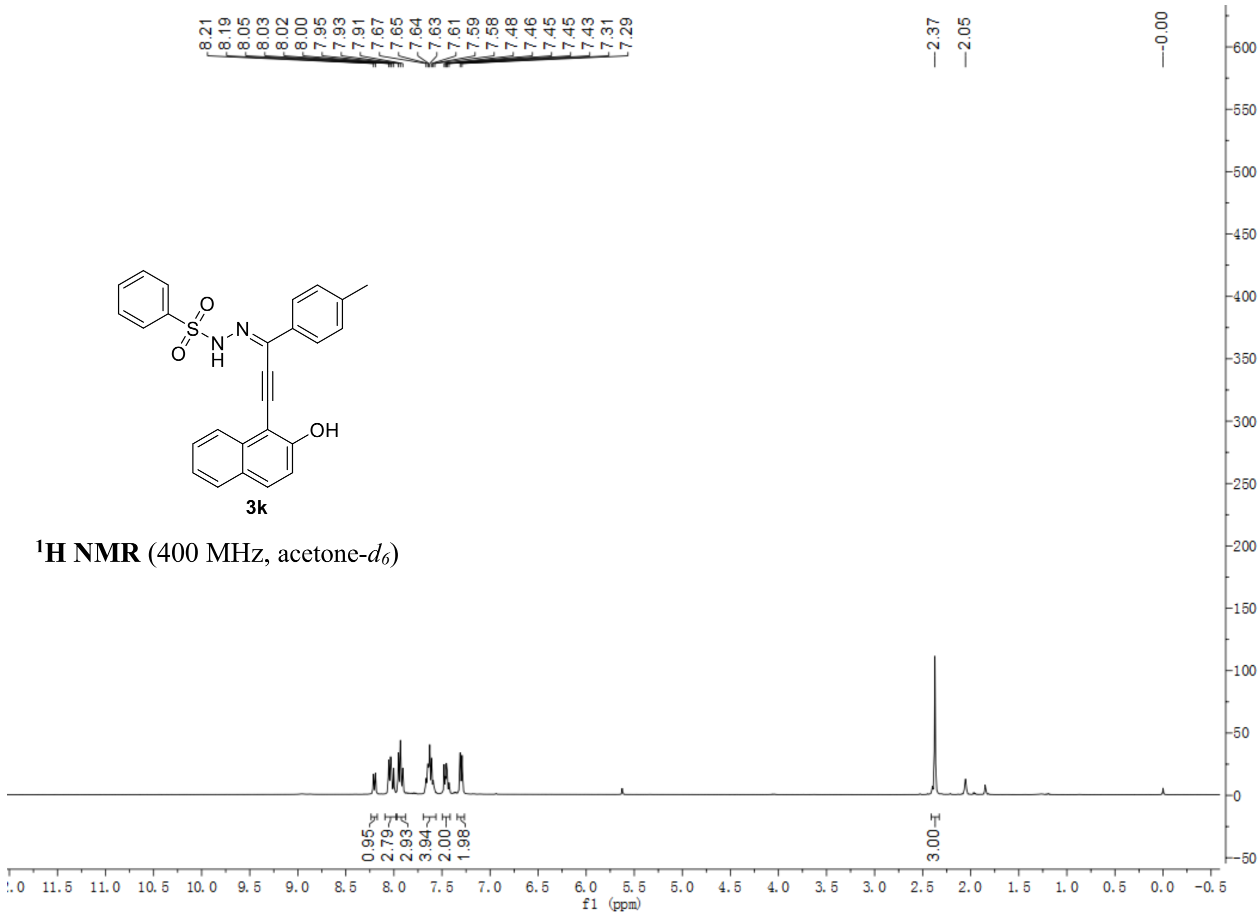

3k

^{1}H NMR (400 MHz, acetone-d_6)

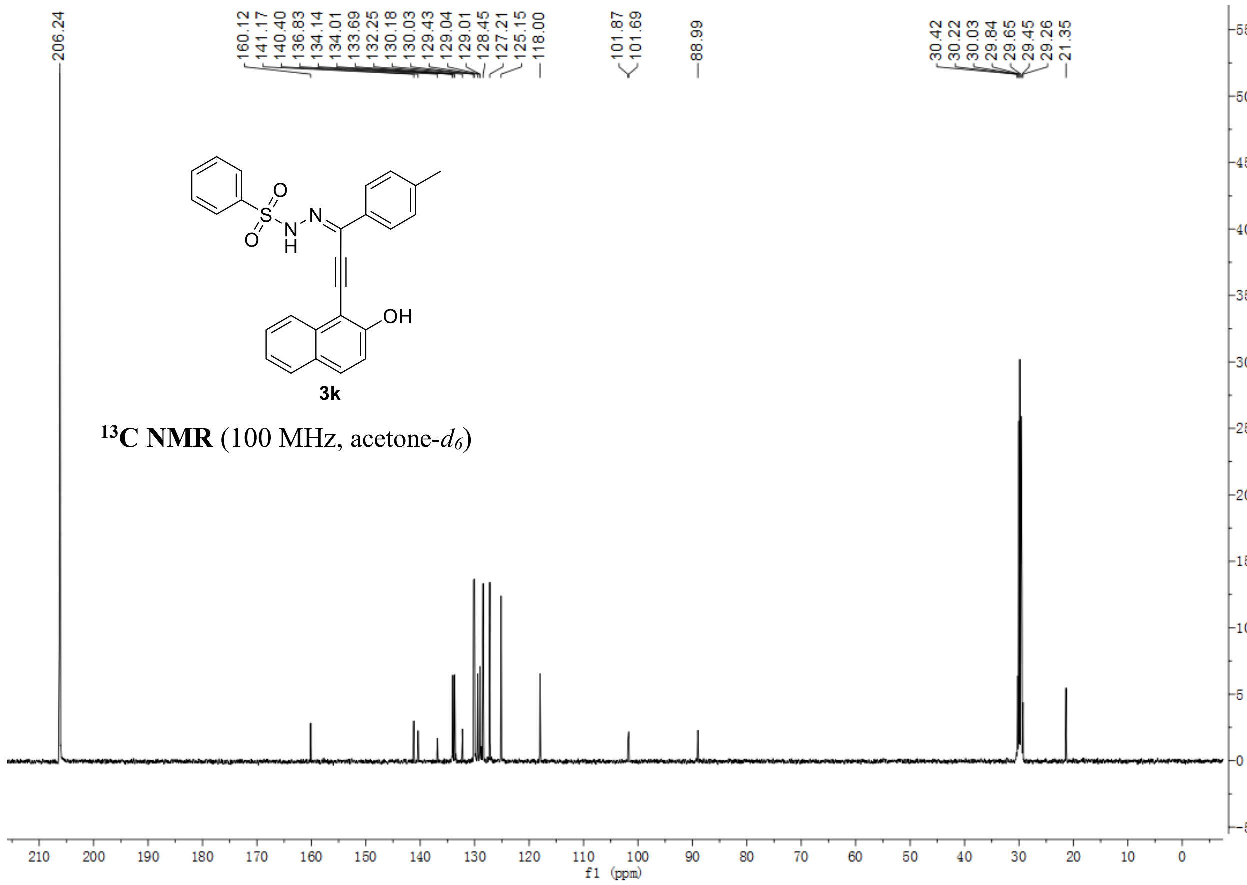

3k

^{13}C NMR (100 MHz, acetone-d_6)

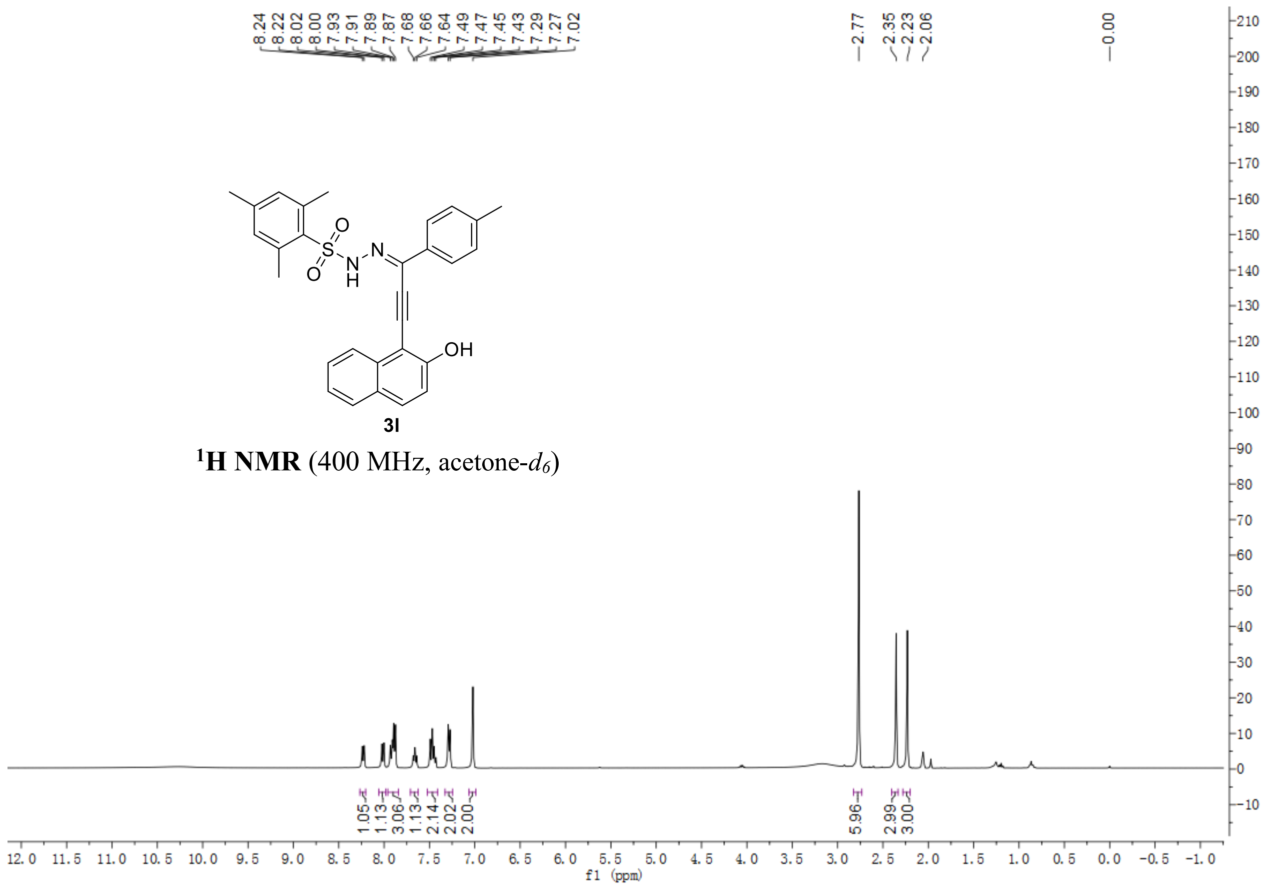

3l

^{1}H NMR (400 MHz, acetone-d_6)

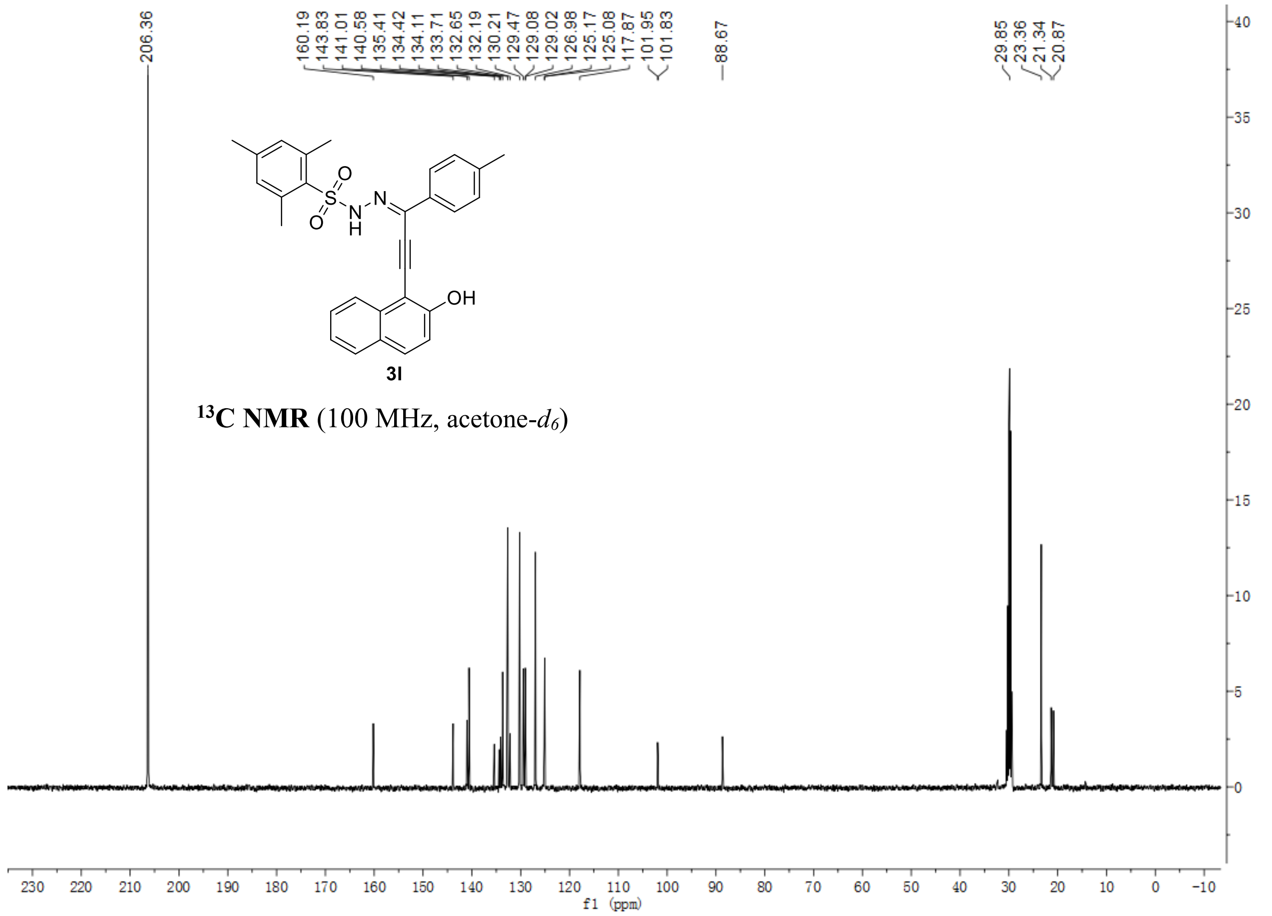

3l

^{13}C NMR (100 MHz, acetone-d_6)

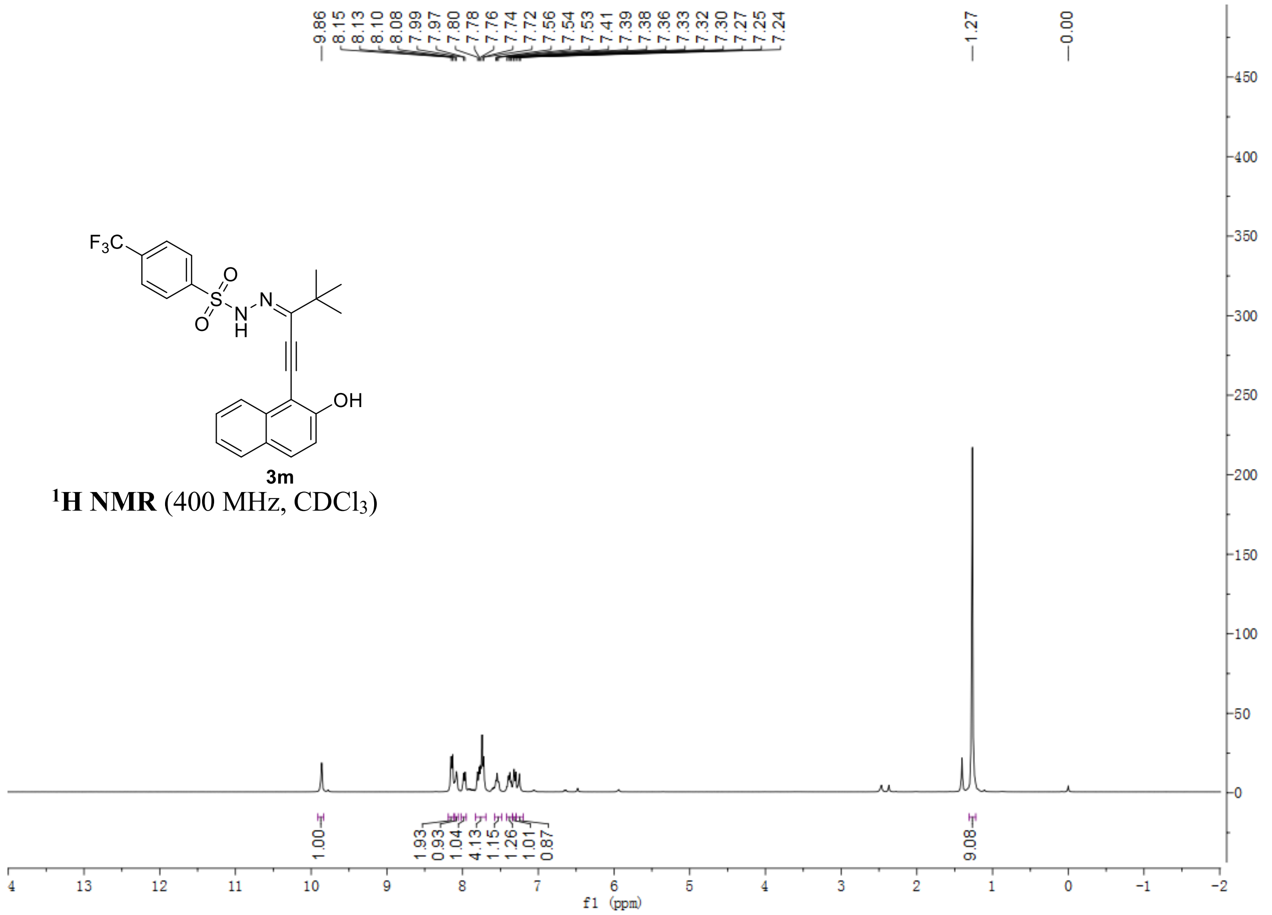

3m

^{1}H NMR (400 MHz, $CDCl_3$)

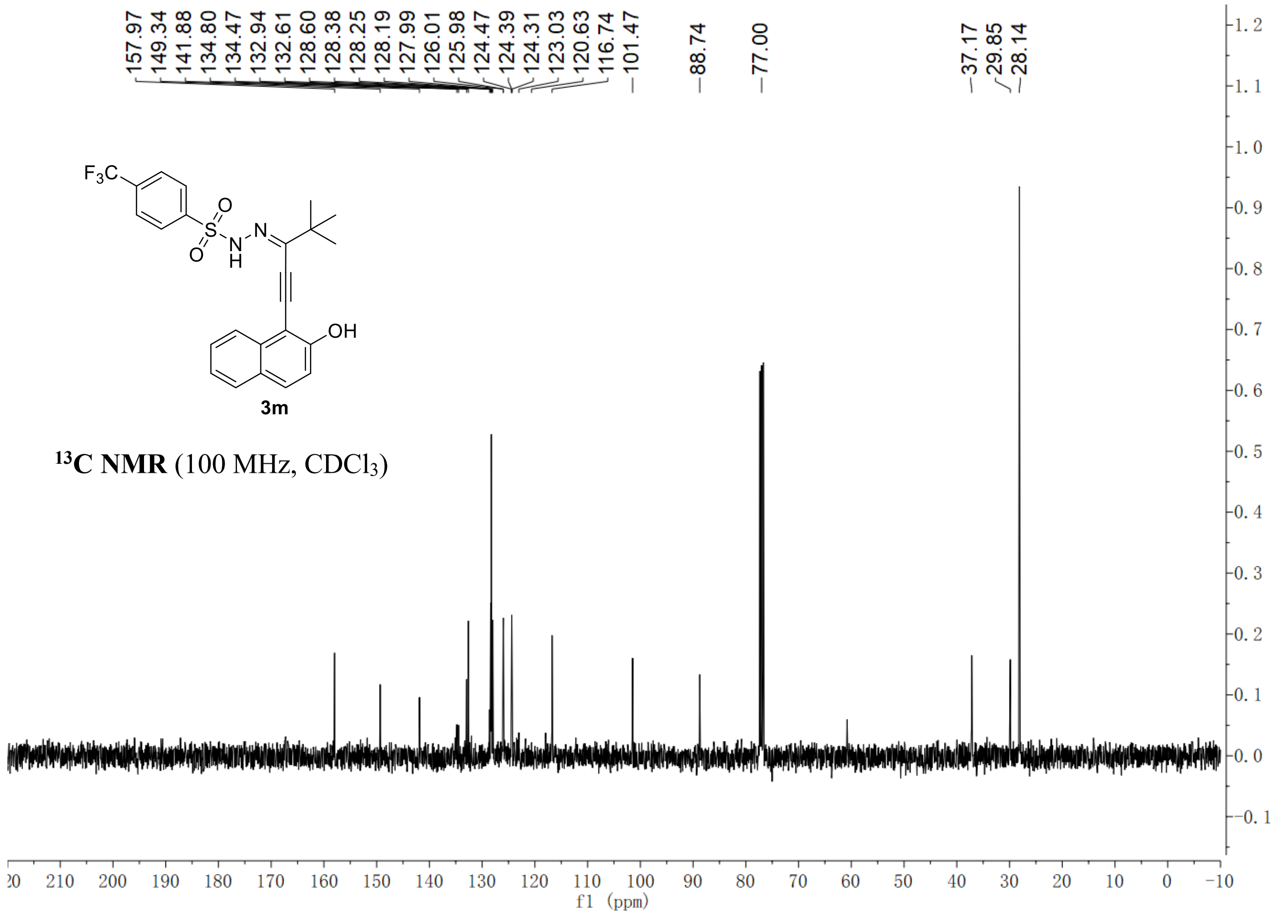

3m

^{13}C NMR (100 MHz, $CDCl_3$)

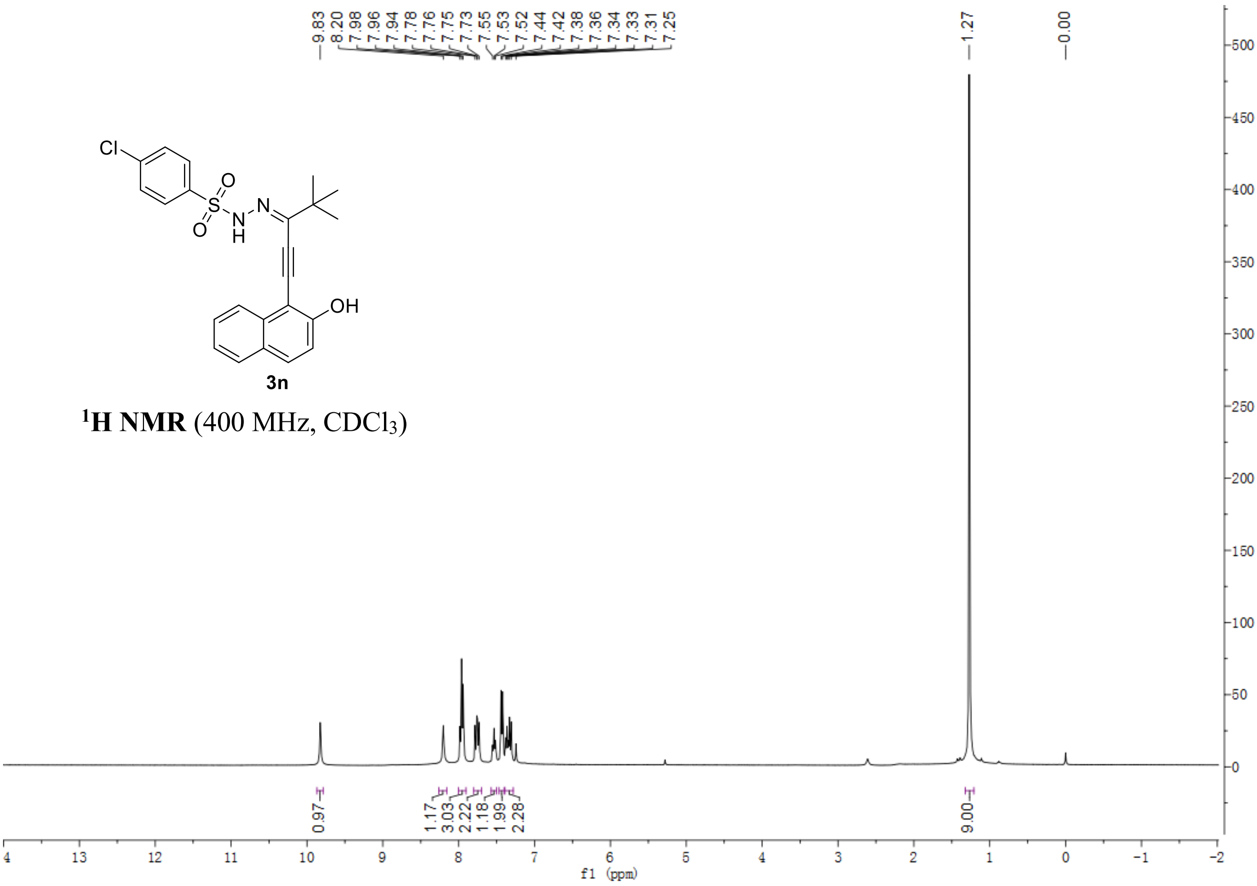
9.83
8.20
7.98
7.96
7.94
7.78
7.76
7.75
7.73
7.55
7.53
7.52
7.44
7.42
7.38
7.36
7.34
7.33
7.31
7.25
1.27
0.00
Cl
O
S
O
N
H
N
OH
3n
0.97
1.17
3.03
2.22
1.18
1.99
2.28
9.00
f1 (ppm)
¹H NMR (400 MHz, CDCl₃)

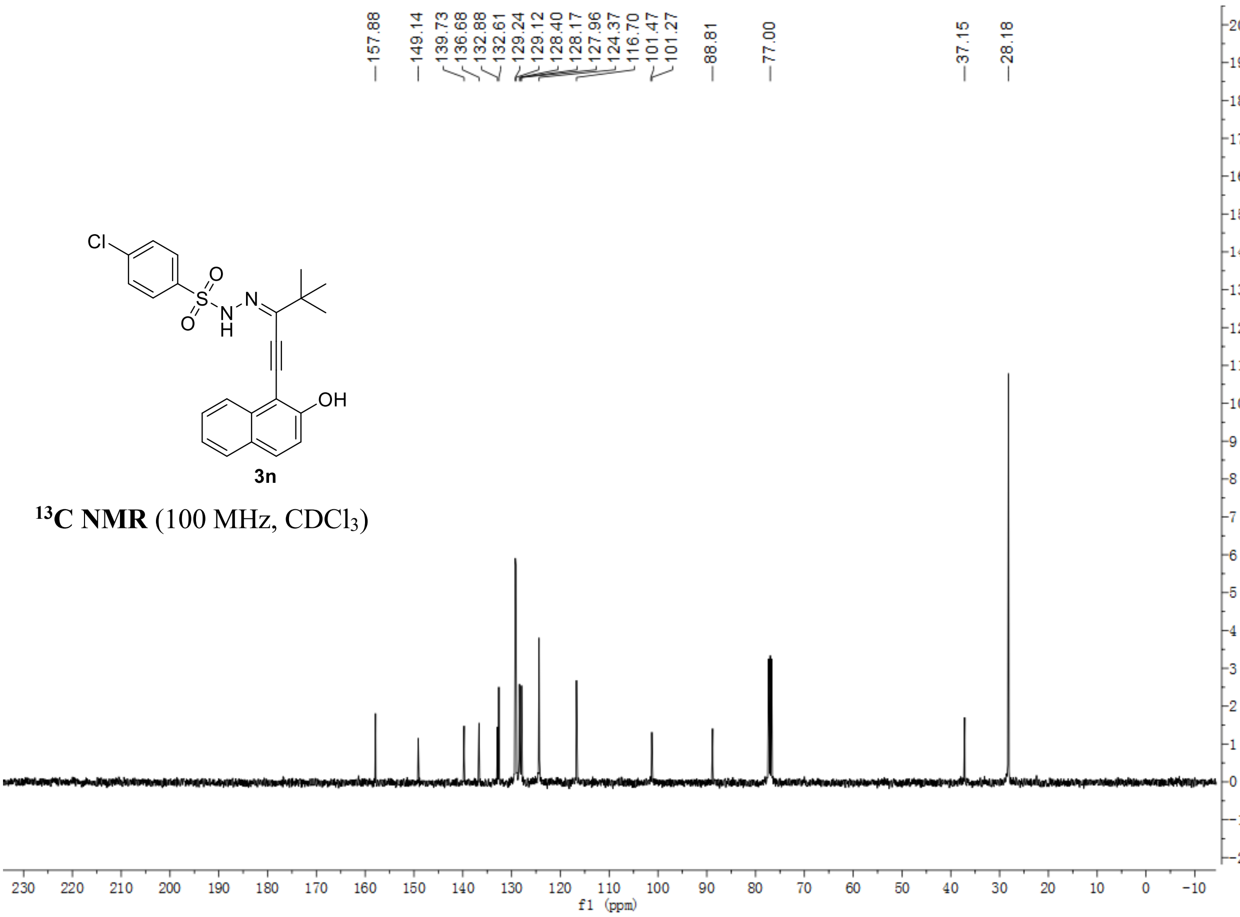
157.88
149.14
139.73
136.68
132.88
132.61
129.24
129.12
128.40
128.17
127.96
124.37
116.70
101.47
101.27
88.81
77.00
37.15
28.18
Cl
O
S
O
N
H
N
OH
3n
f1 (ppm)
¹³C NMR (100 MHz, CDCl₃)

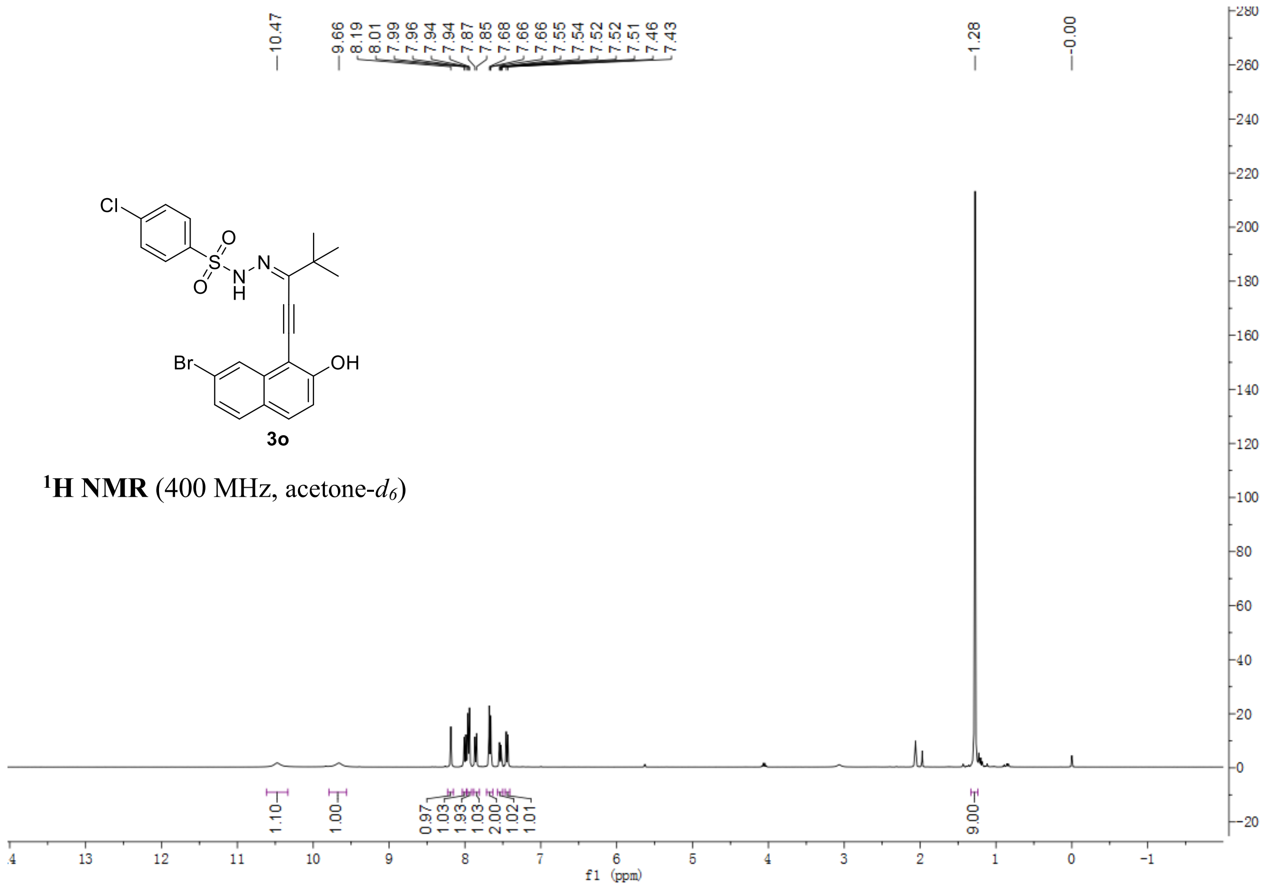
10.47
9.66
8.19
8.01
7.99
7.96
7.94
7.94
7.87
7.85
7.68
7.66
7.66
7.55
7.54
7.52
7.52
7.51
7.46
7.43
1.28
0.00
Cl
O
S
O
N
H
N
Br
OH
3o
1H NMR (400 MHz, acetone-d6)
1.10
1.00
0.97
1.03
1.93
1.03
2.00
1.02
1.01
9.00
f1 (ppm)

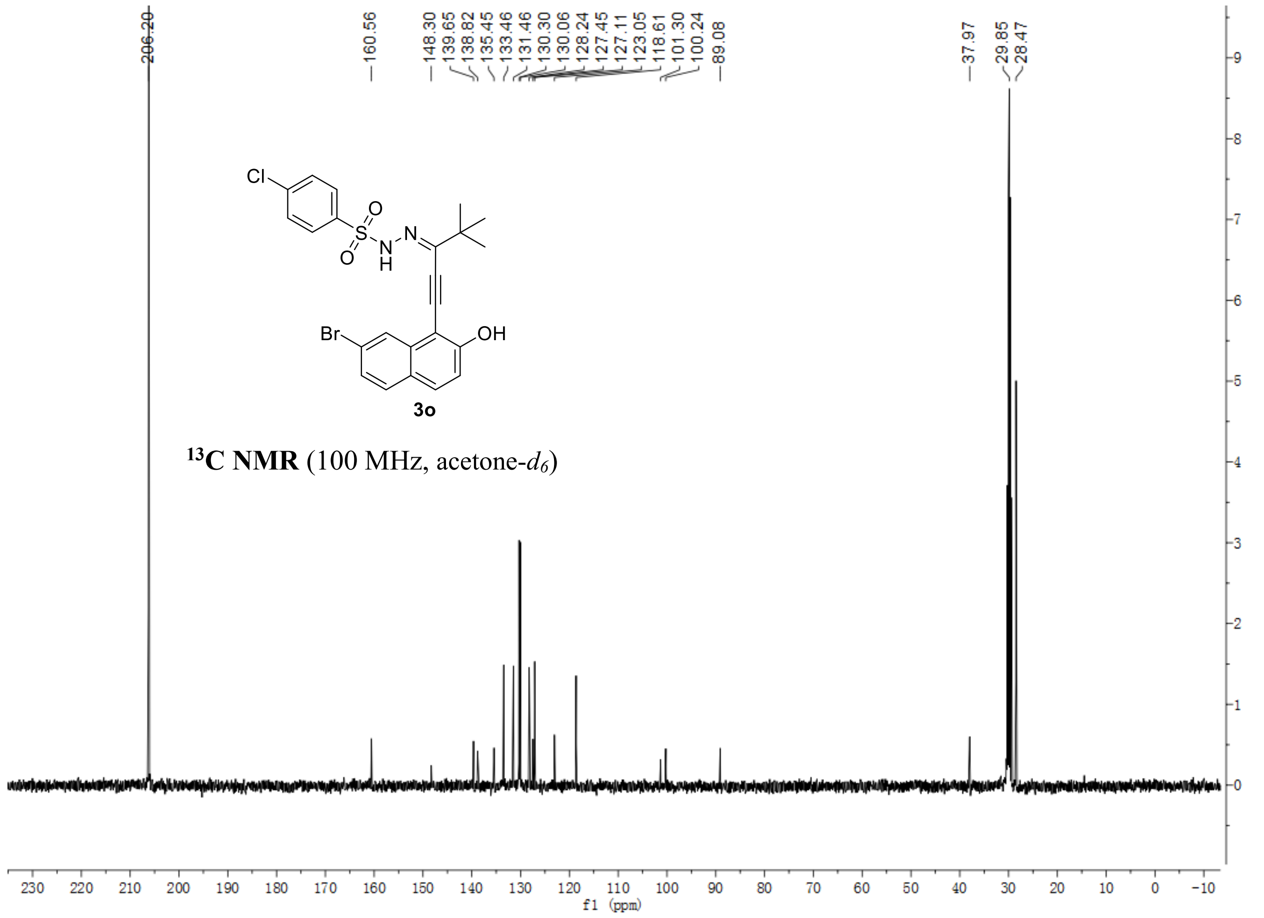
206.20
160.56
148.30
139.65
138.82
135.45
133.46
131.46
130.30
130.06
128.24
127.45
127.11
123.05
118.61
101.30
100.24
89.08
37.97
29.85
28.47
Cl
O
S
O
N
H
N
Br
OH
3o
13C NMR (100 MHz, acetone-d6)
f1 (ppm)

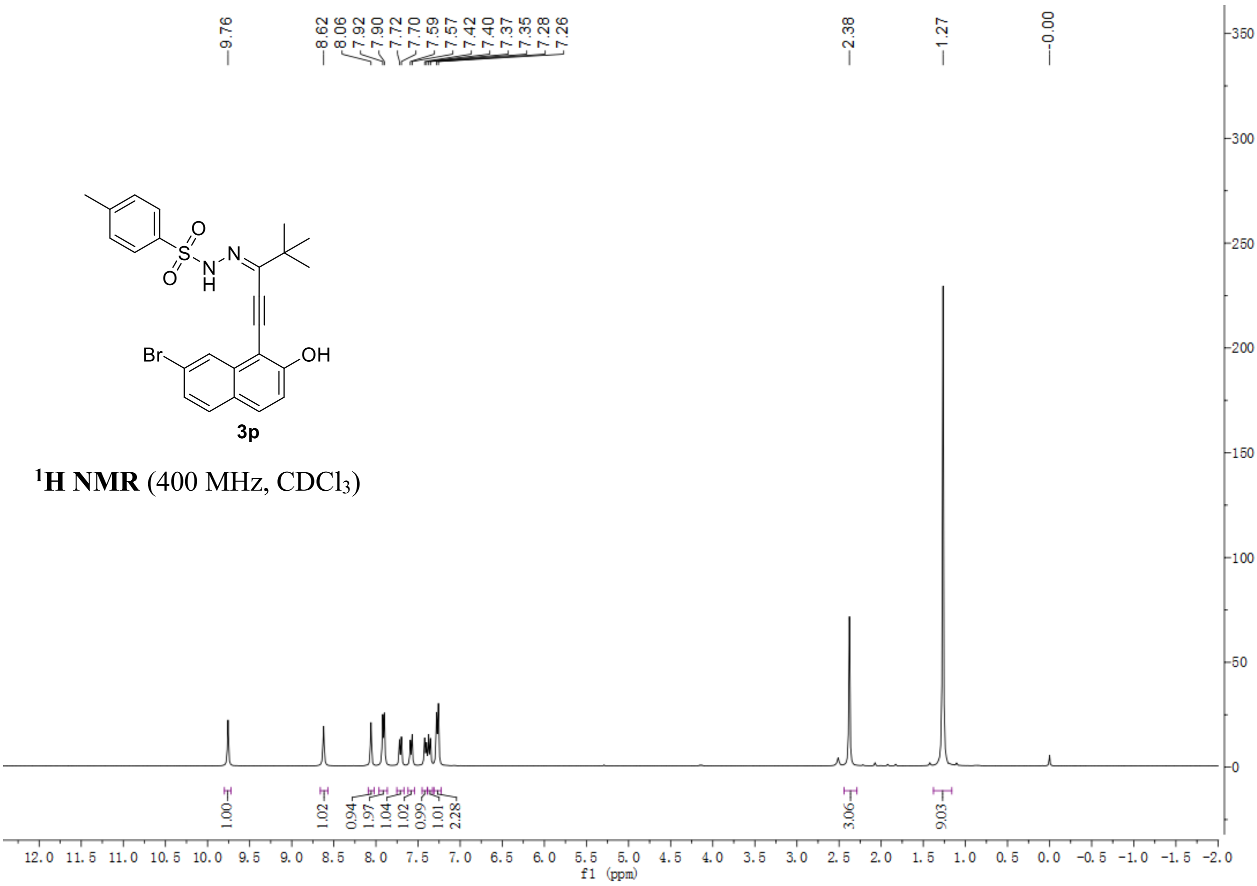
3p
¹H NMR (400 MHz, CDCl3)
9.76
8.62
8.06
7.92
7.90
7.72
7.70
7.59
7.57
7.42
7.40
7.37
7.35
7.28
7.26
2.38
1.27
0.00
1.00
1.02
0.94
1.97
1.04
1.02
0.99
1.01
2.28
3.06
9.03
f1 (ppm)

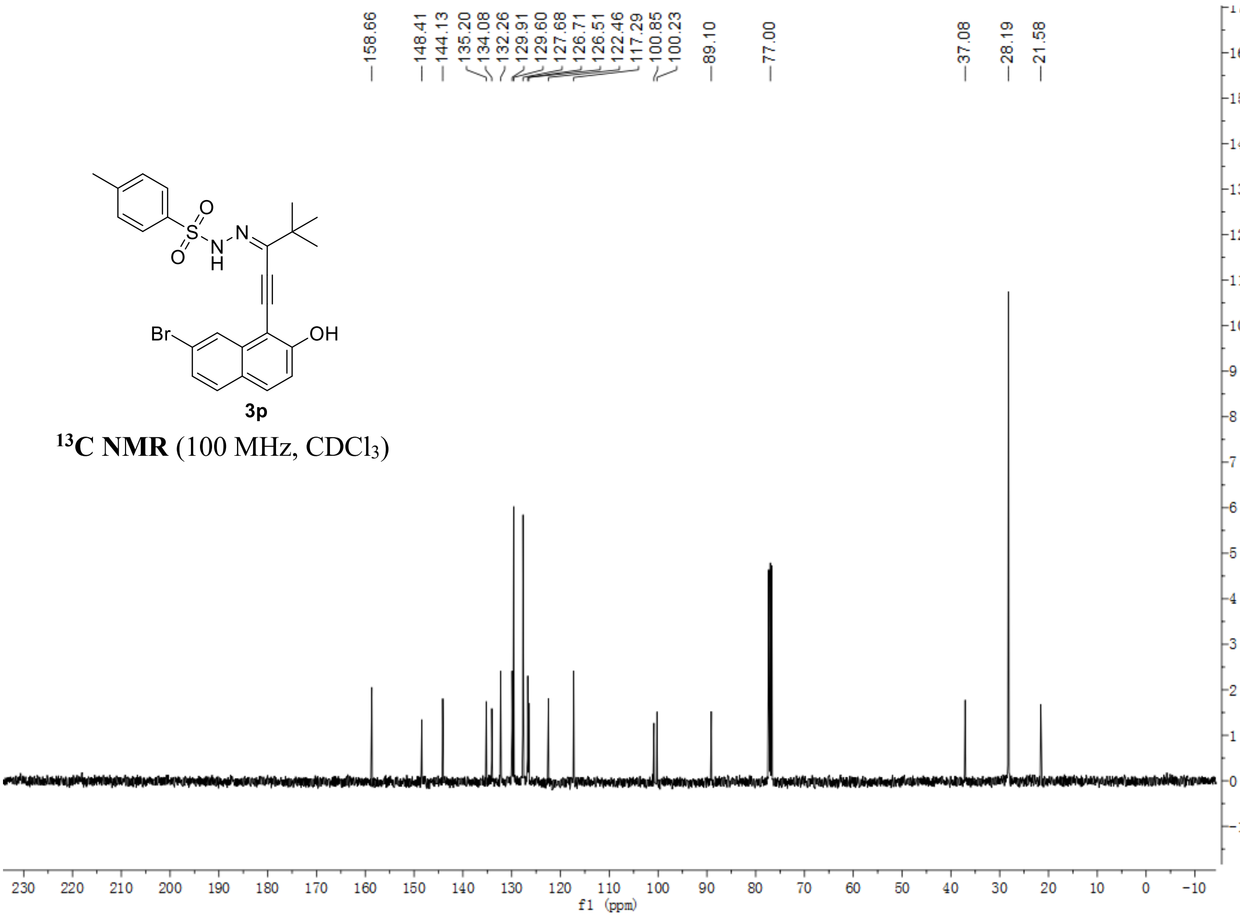
3p
¹³C NMR (100 MHz, CDCl3)
158.66
148.41
144.13
135.20
134.08
132.26
129.91
129.60
127.68
126.71
126.51
122.46
117.29
100.85
100.23
89.10
77.00
37.08
28.19
21.58
f1 (ppm)

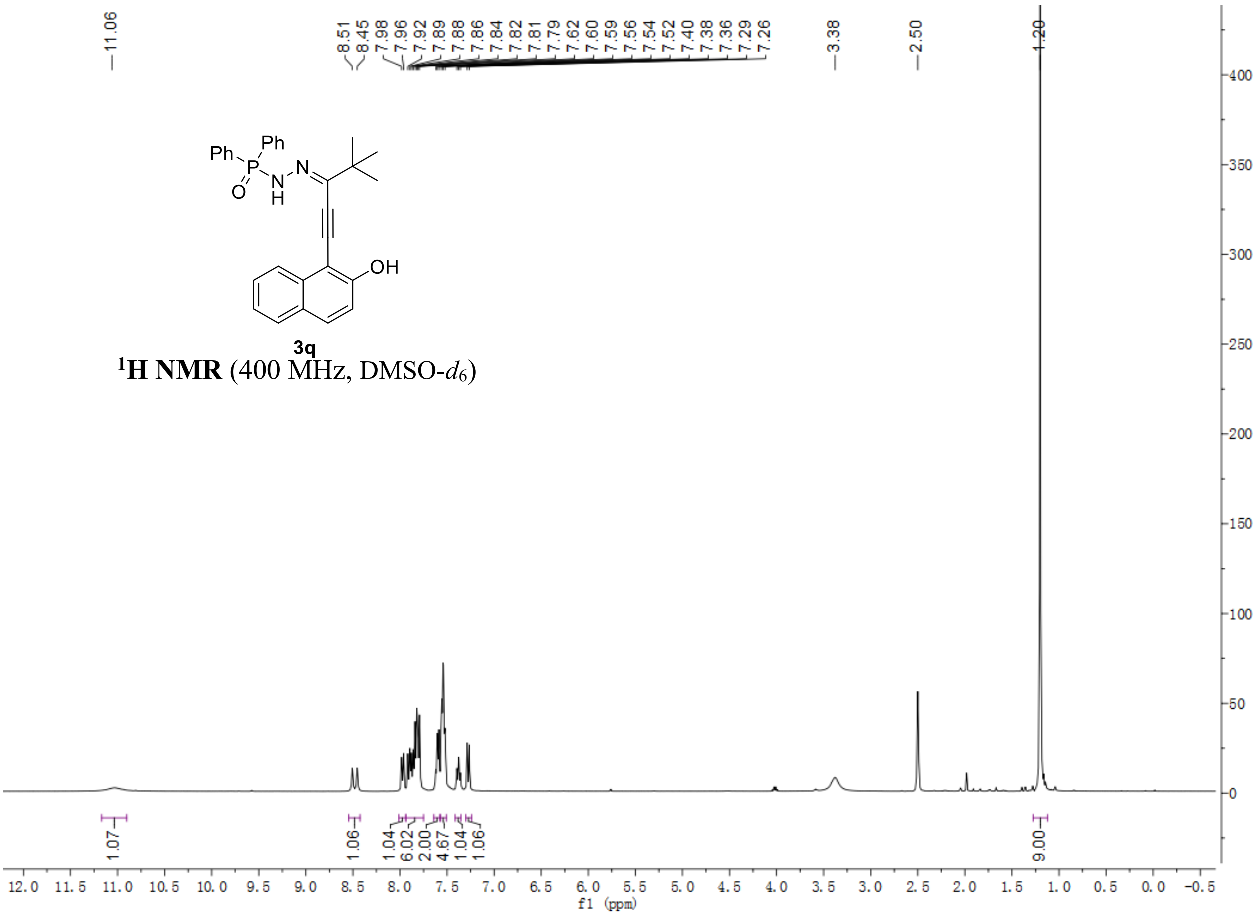

^{1}H NMR (400 MHz, DMSO-d_6)

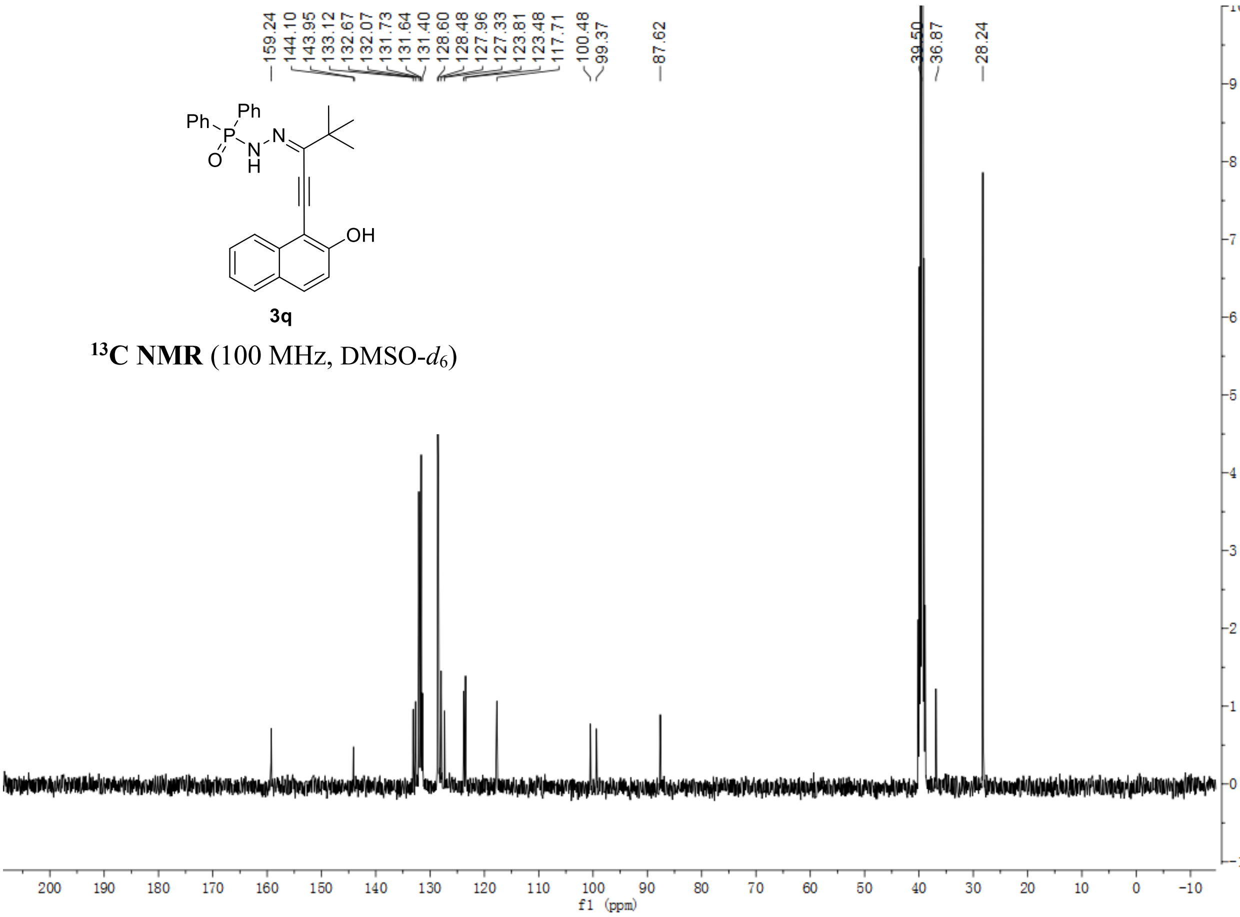

^{13}C NMR (100 MHz, DMSO-d_6)

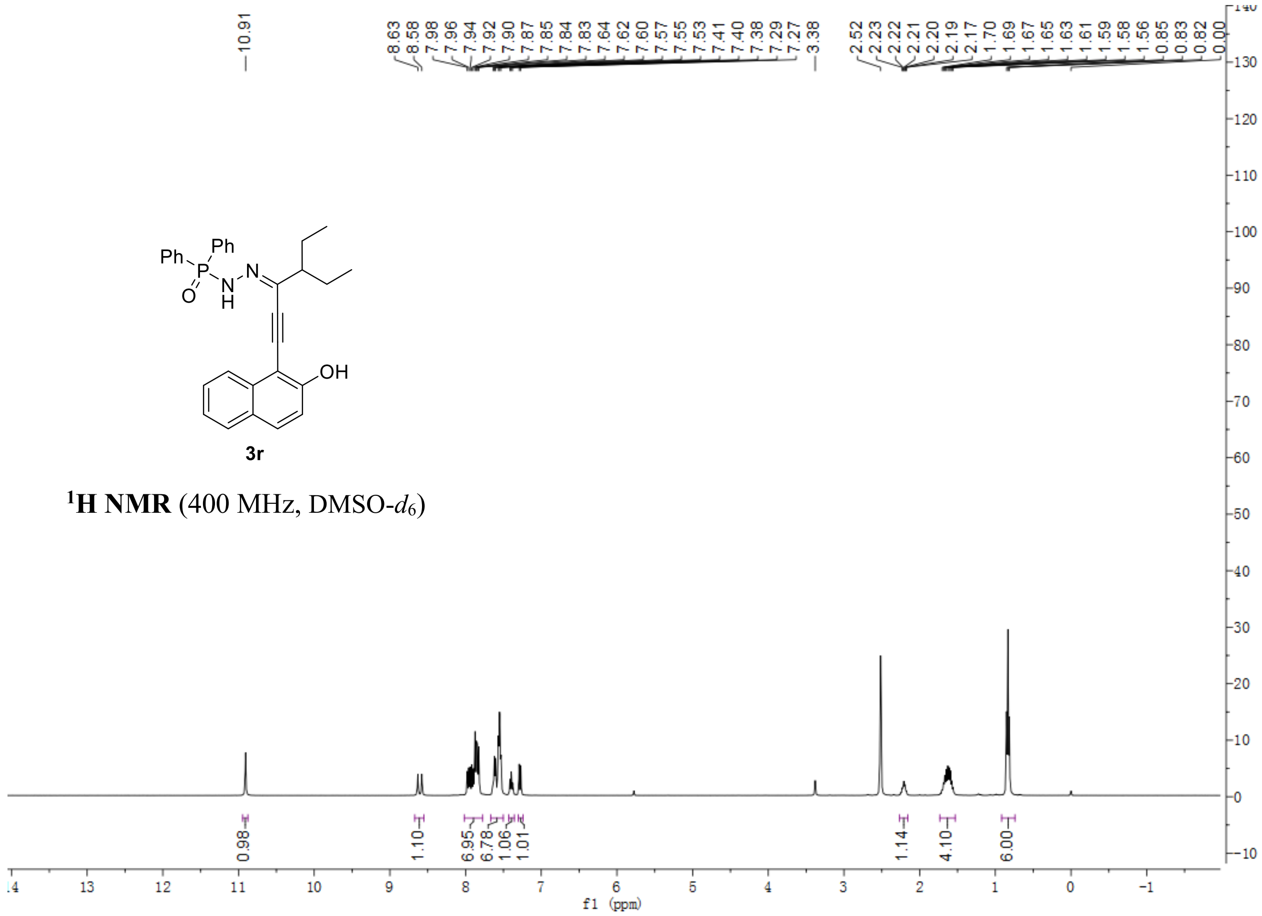

3r

¹H NMR (400 MHz, DMSO-d_6)

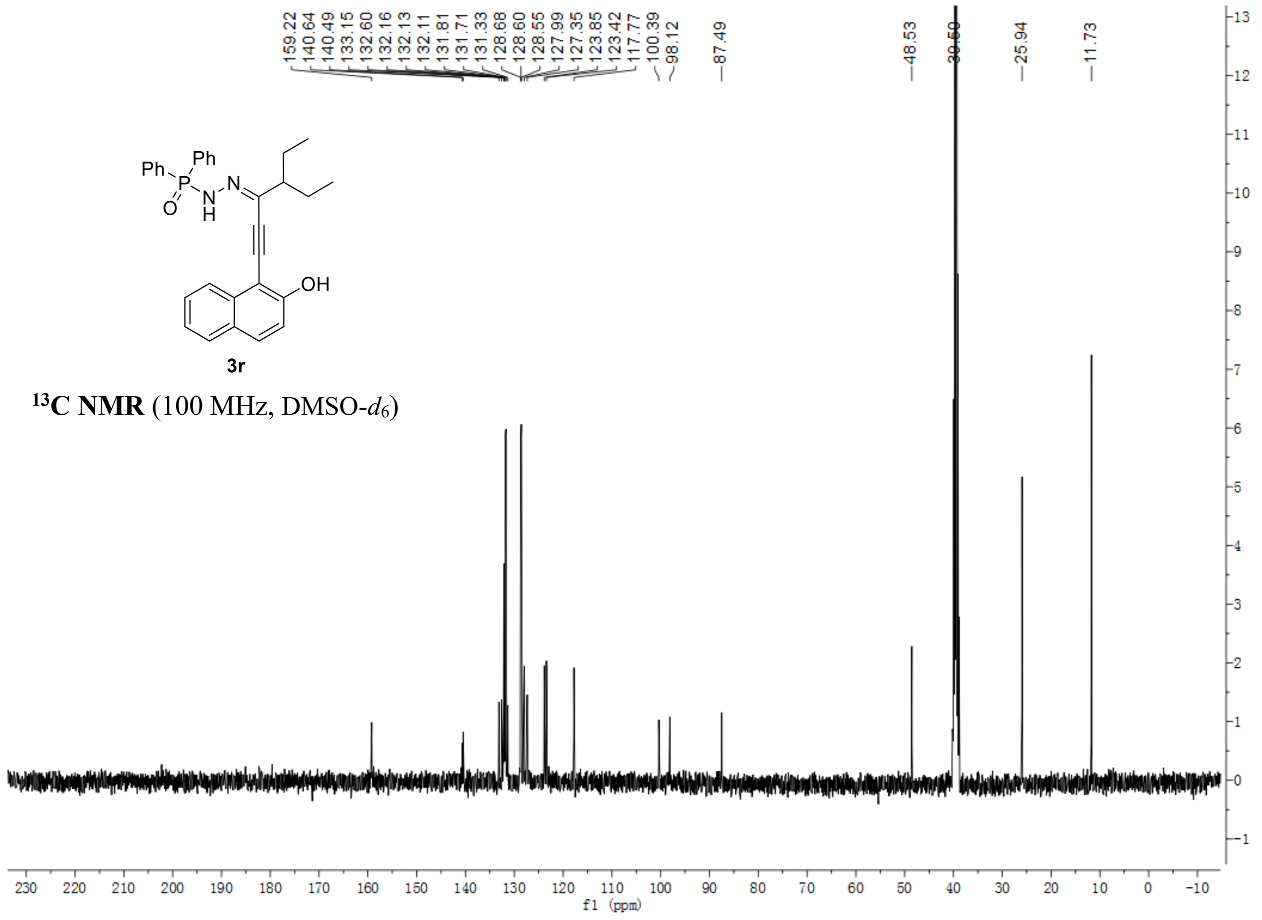

3r

¹³C NMR (100 MHz, DMSO-d_6)

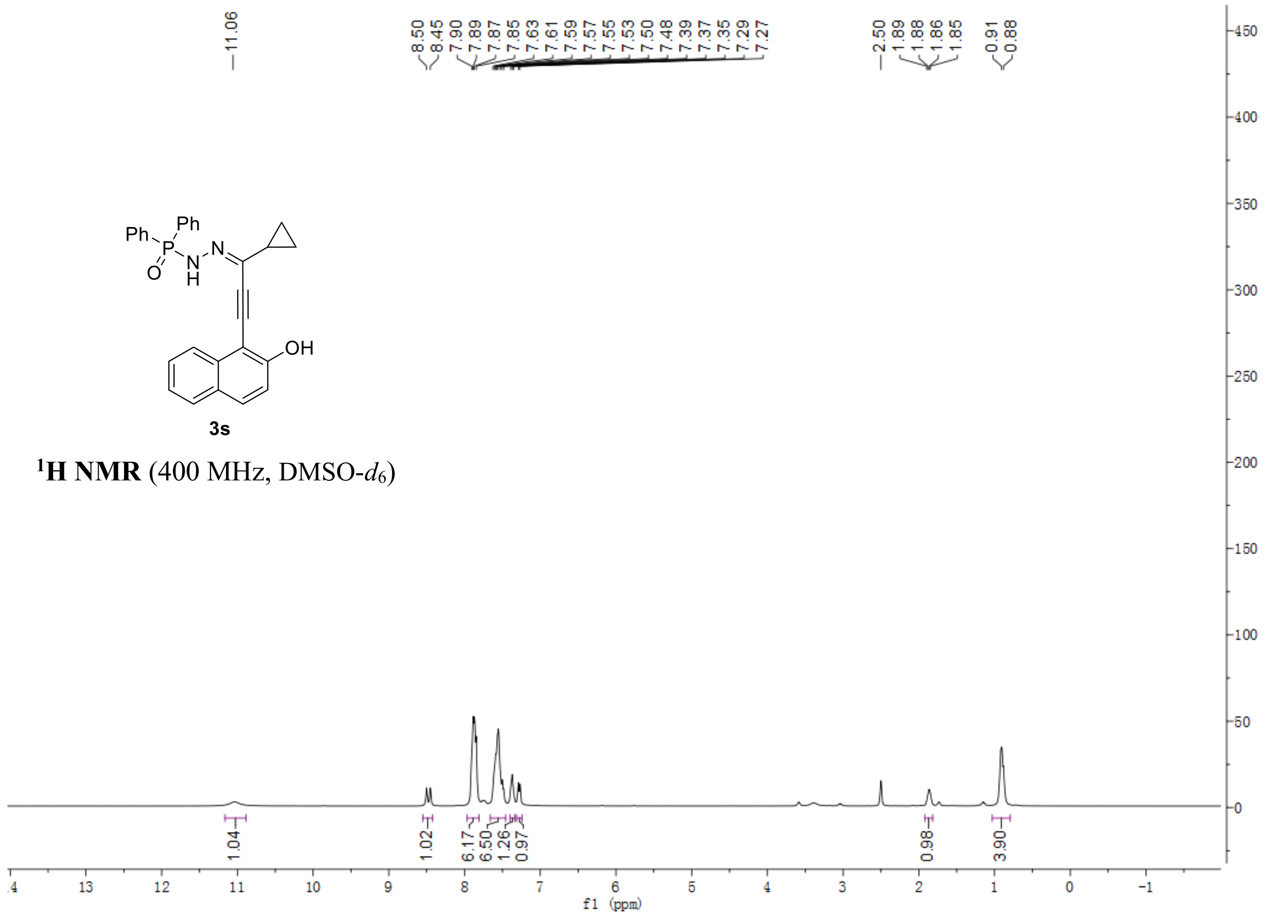
11.06
8.50
8.45
7.90
7.89
7.87
7.85
7.63
7.61
7.59
7.57
7.55
7.53
7.50
7.48
7.39
7.37
7.35
7.29
7.27
2.50
1.89
1.88
1.86
1.85
0.91
0.88
Ph
Ph
P
O
N
H
N
OH
3s
1H NMR (400 MHz, DMSO-d6)
1.04
1.02
6.17
6.50
1.26
0.97
0.98
3.90
f1 (ppm)

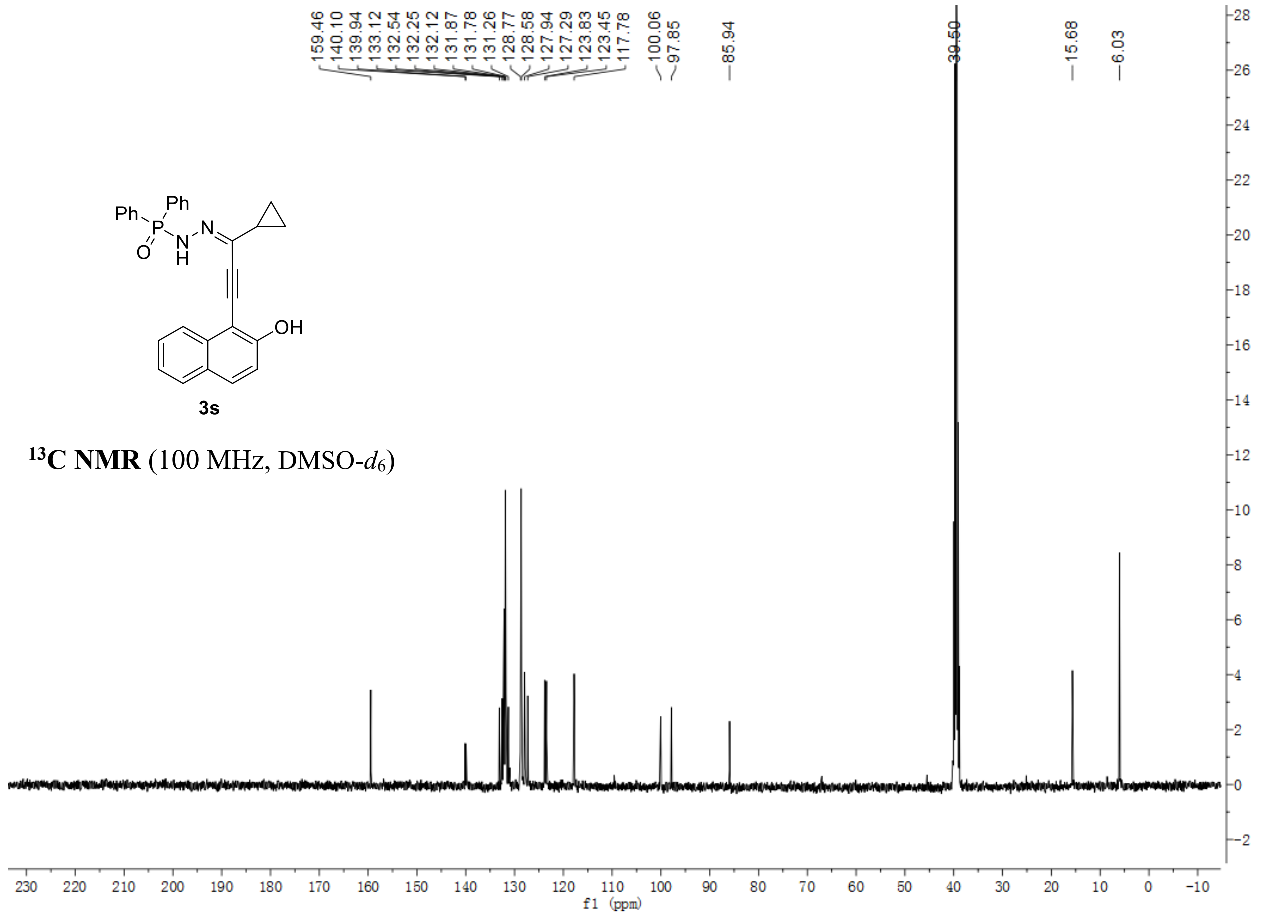
159.46
140.10
139.94
133.12
132.54
132.25
132.12
131.87
131.78
131.26
128.77
128.58
127.94
127.29
123.83
123.45
117.78
100.06
97.85
85.94
39.50
15.68
6.03
Ph
Ph
P
O
N
H
N
OH
3s
13C NMR (100 MHz, DMSO-d6)
f1 (ppm)

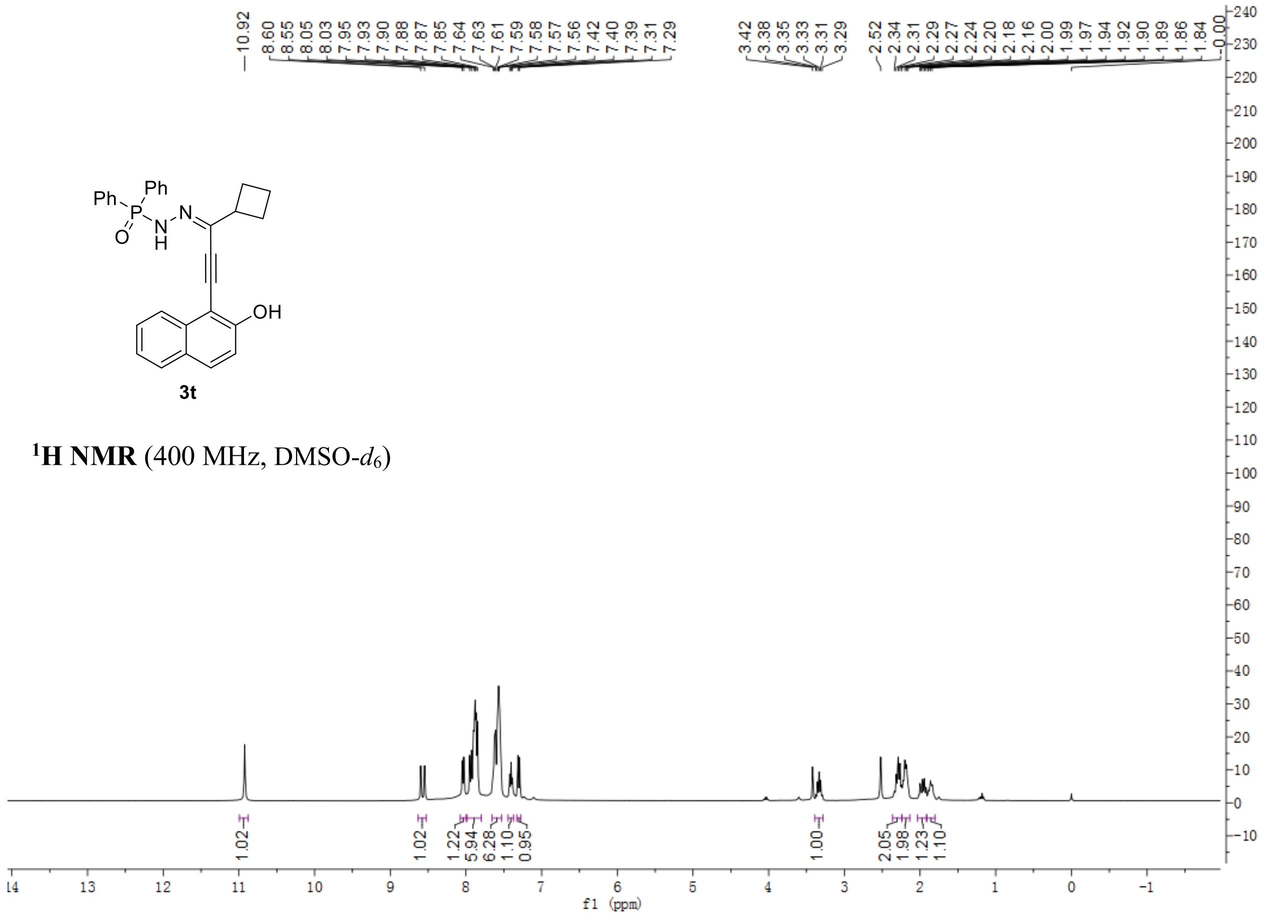

^{1}H NMR (400 MHz, DMSO-d_6)

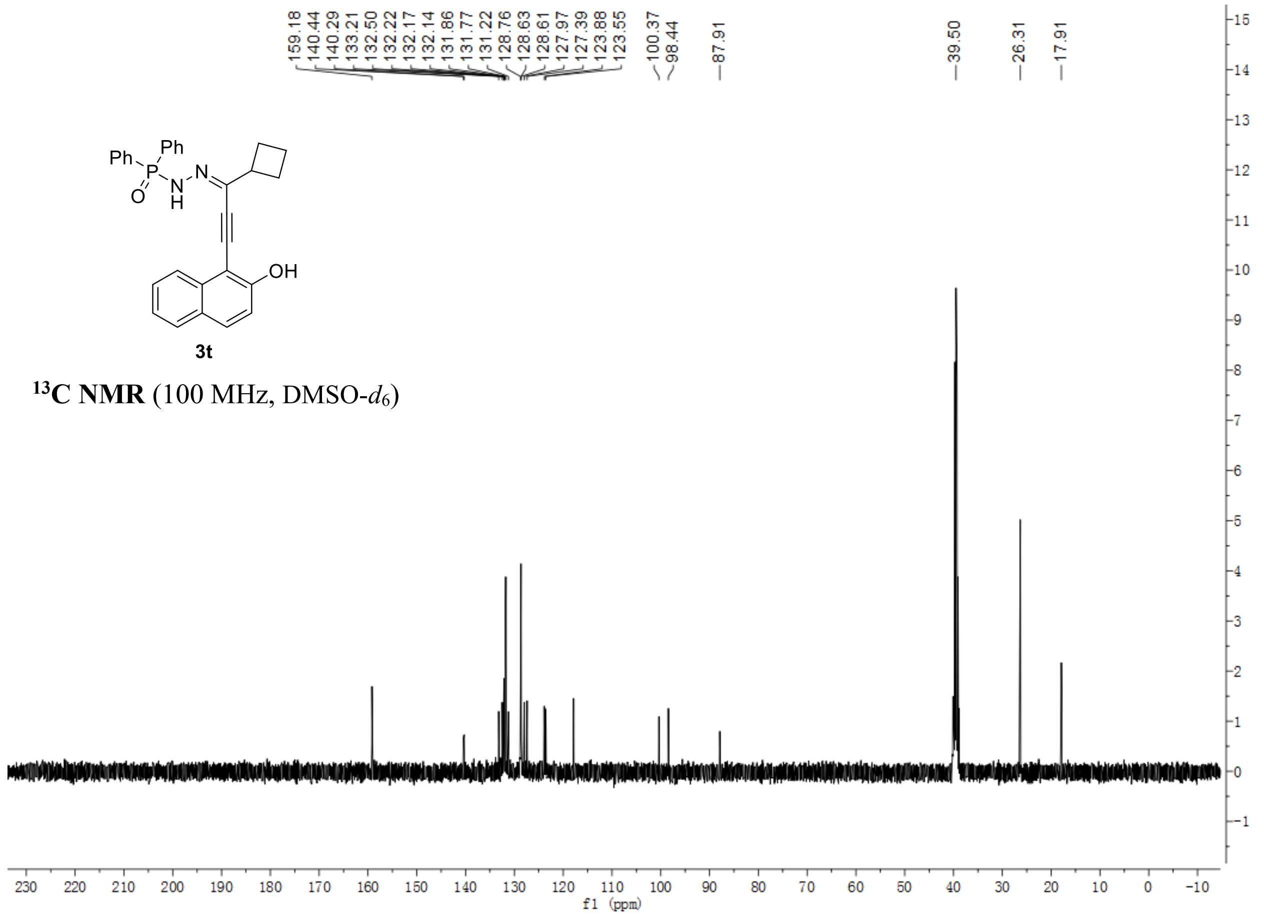

^{13}C NMR (100 MHz, DMSO-d_6)

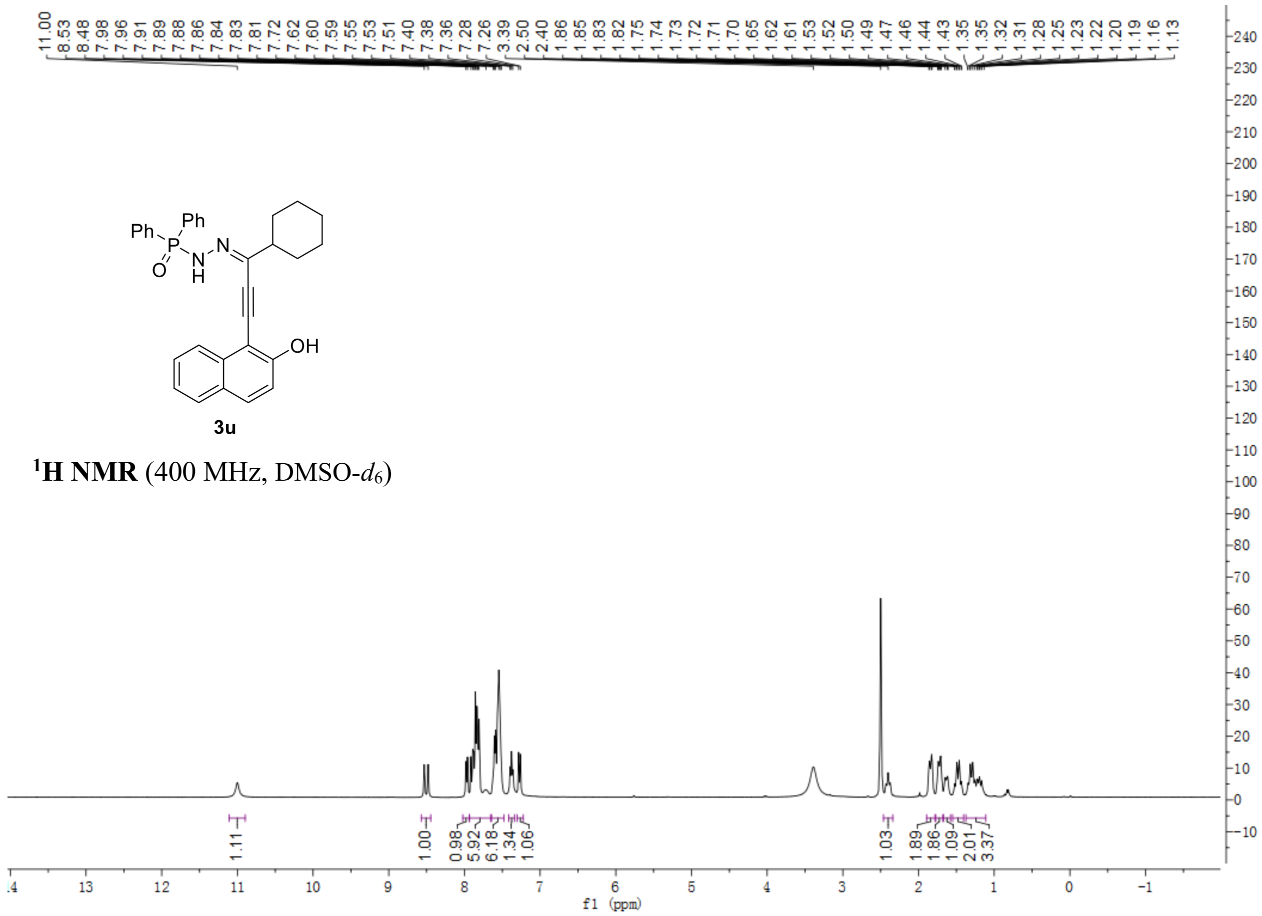

^{1}H NMR (400 MHz, DMSO-d_6)

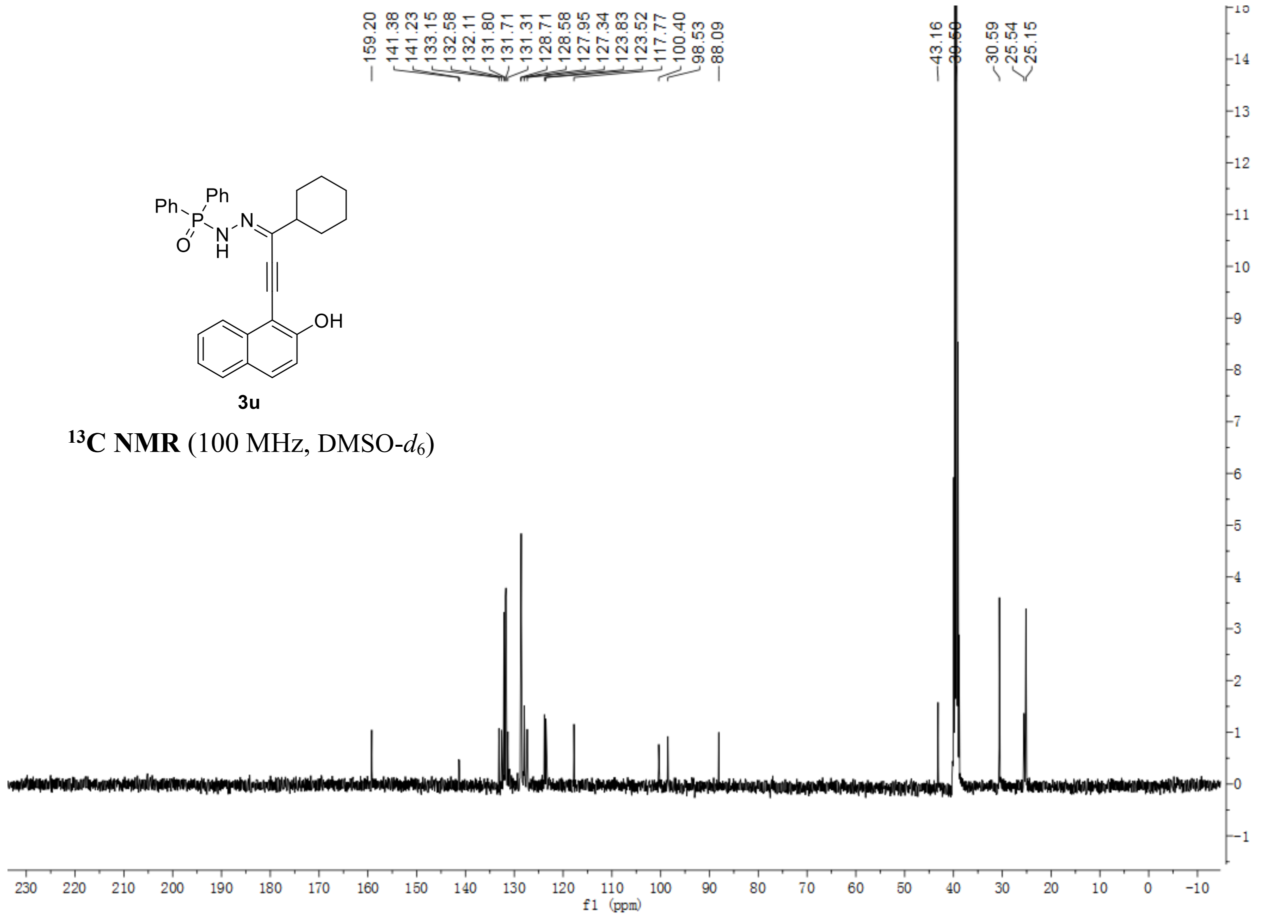

^{13}C NMR (100 MHz, DMSO-d_6)

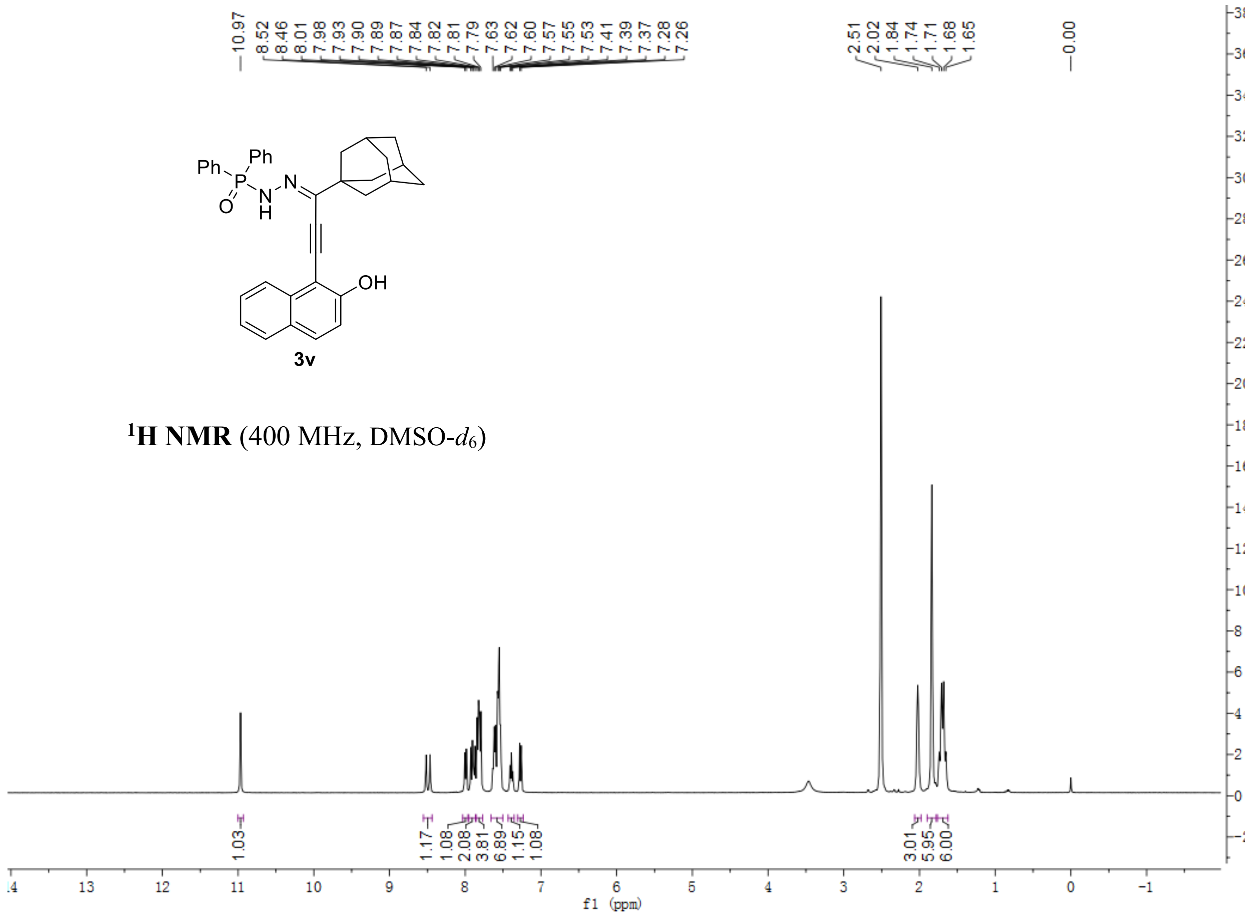

3v

^{1}H NMR (400 MHz, DMSO-d_6)

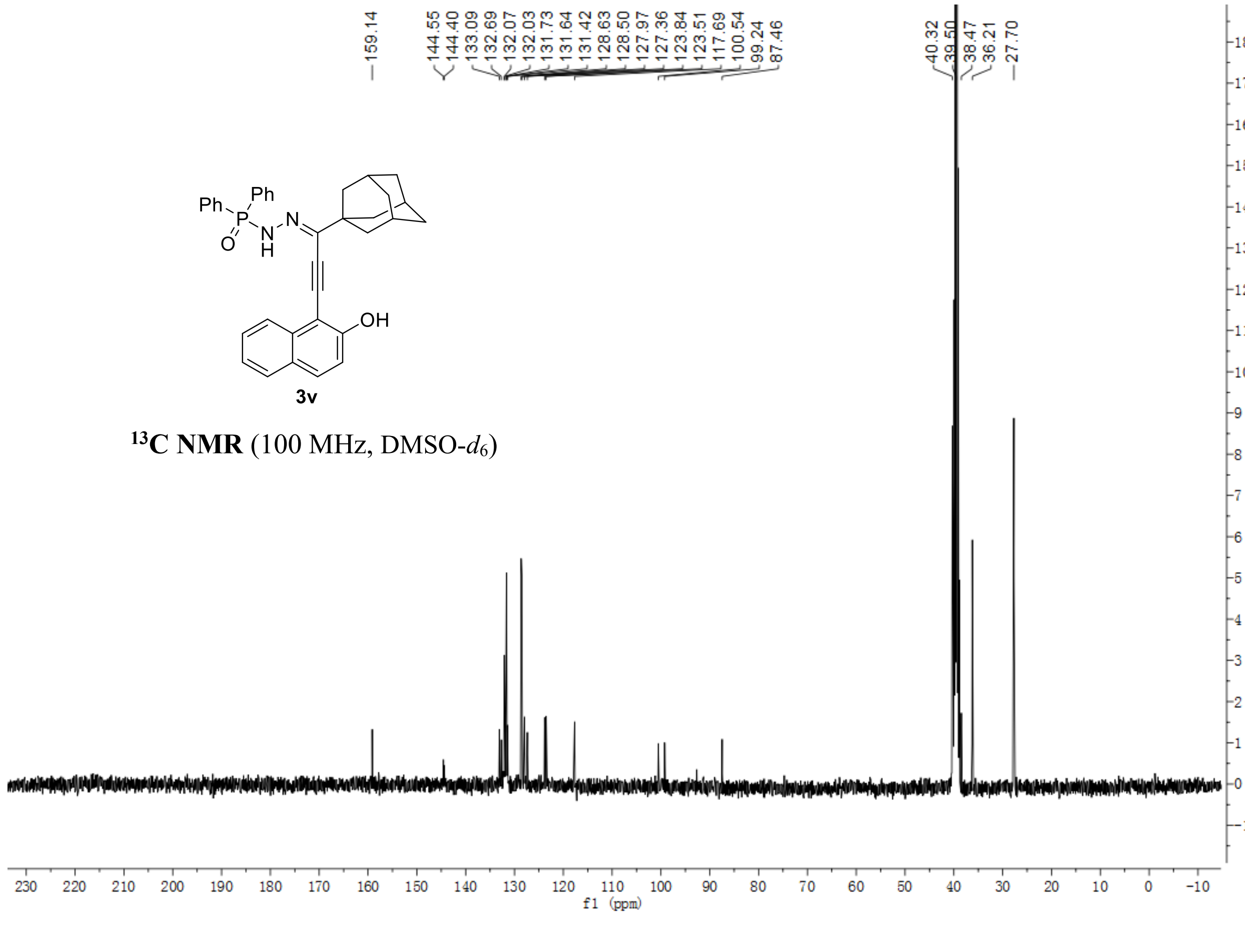

3v

^{13}C NMR (100 MHz, DMSO-d_6)

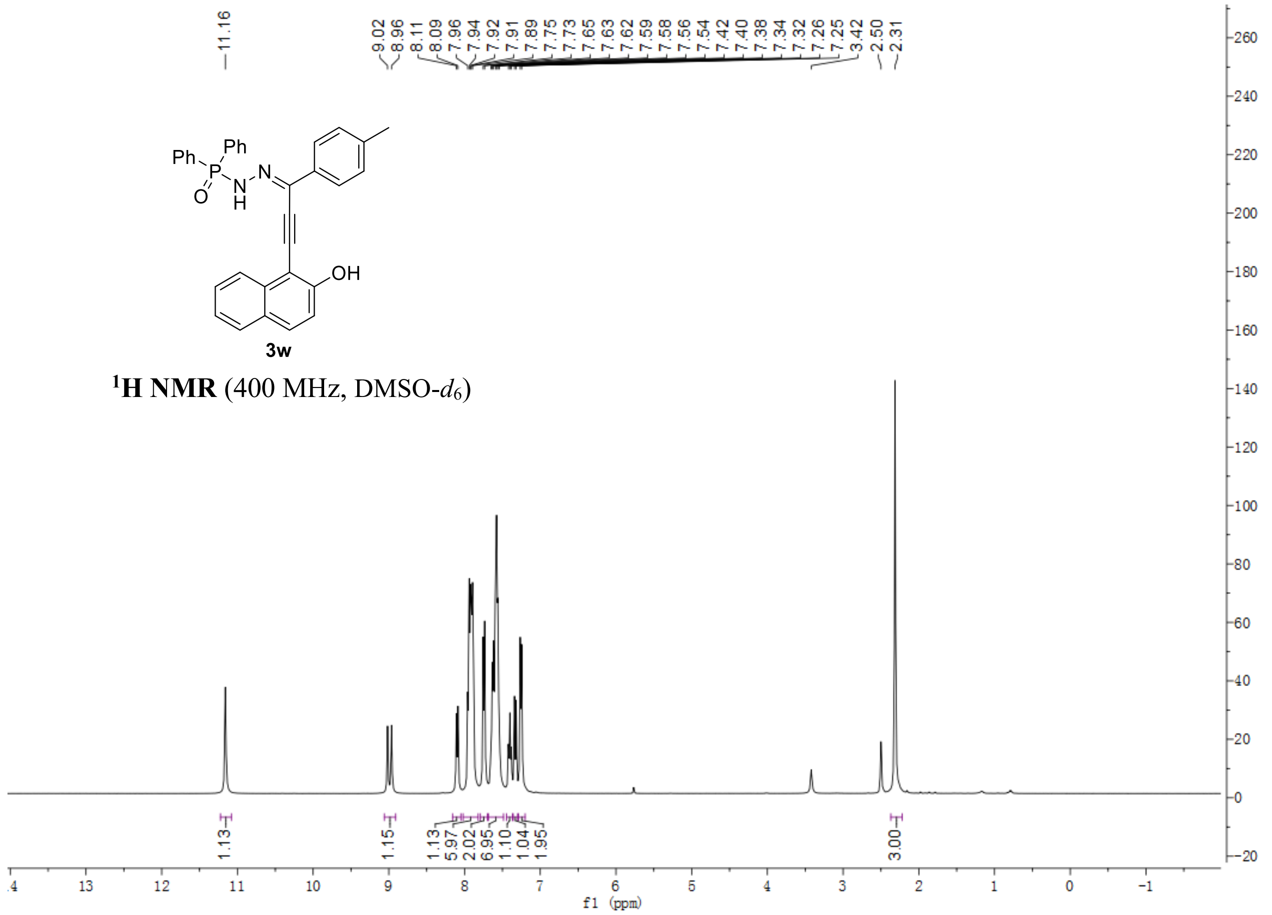

^{1}**H NMR** (400 MHz, DMSO-d_6)

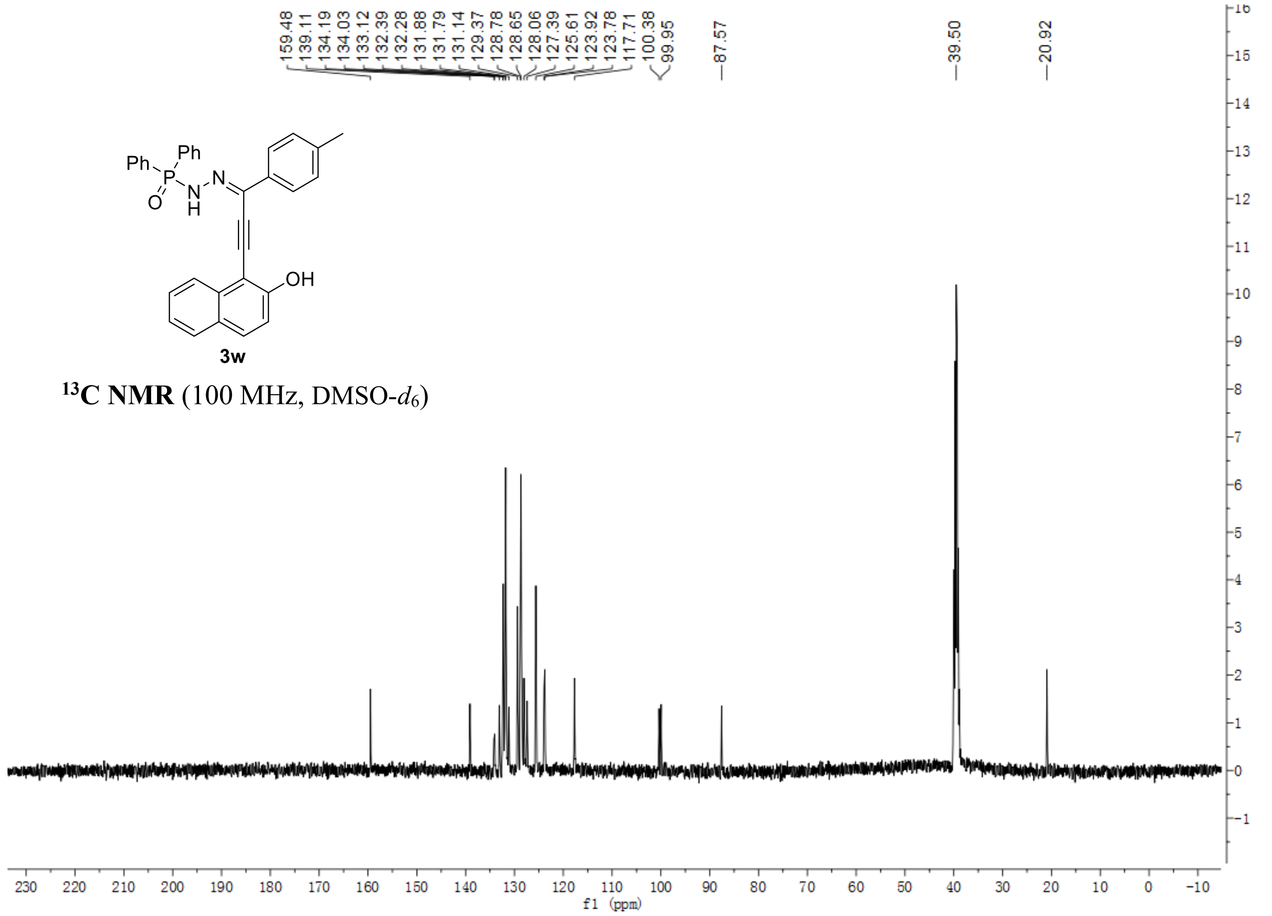

^{13}**C NMR** (100 MHz, DMSO-d_6)

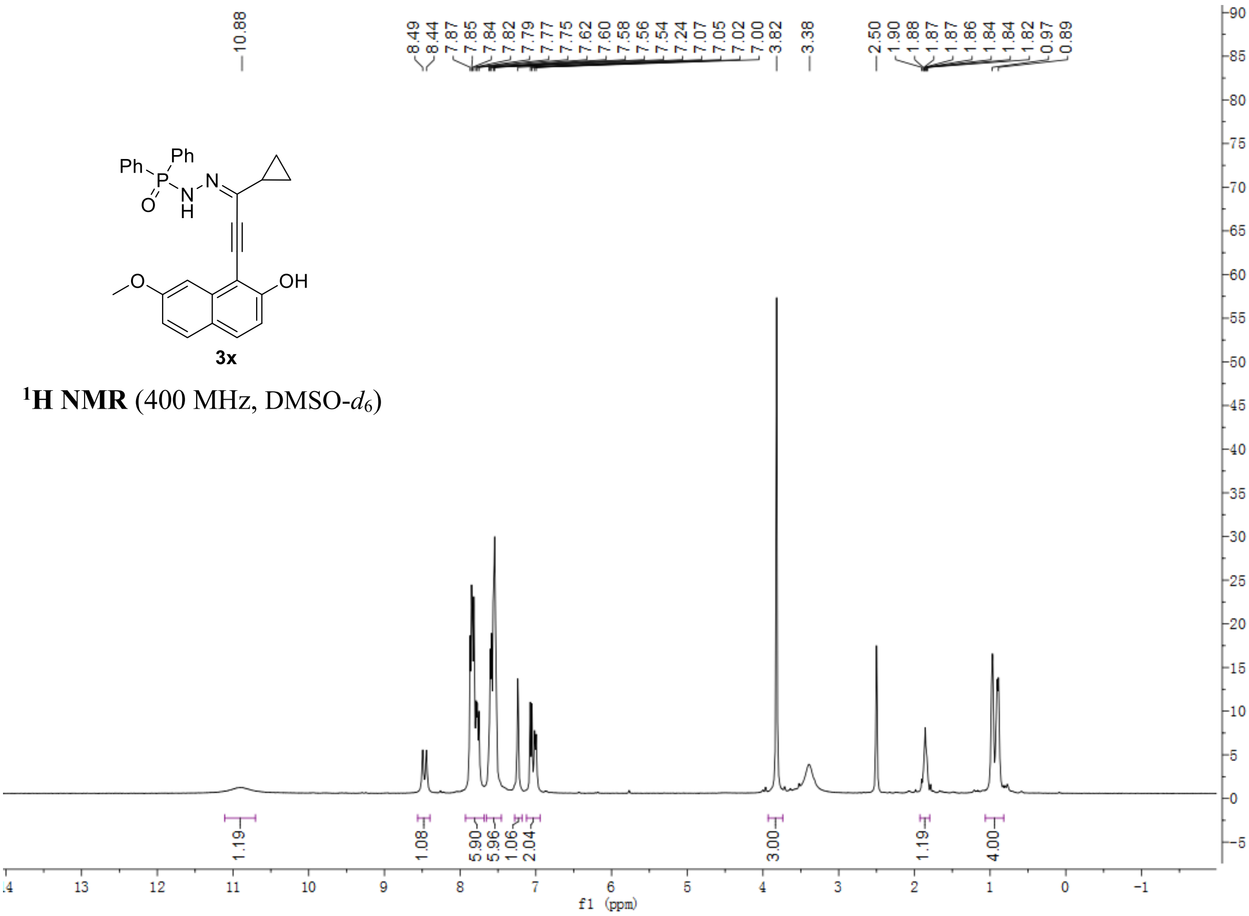

3x

^{1}H NMR (400 MHz, DMSO-d_6)

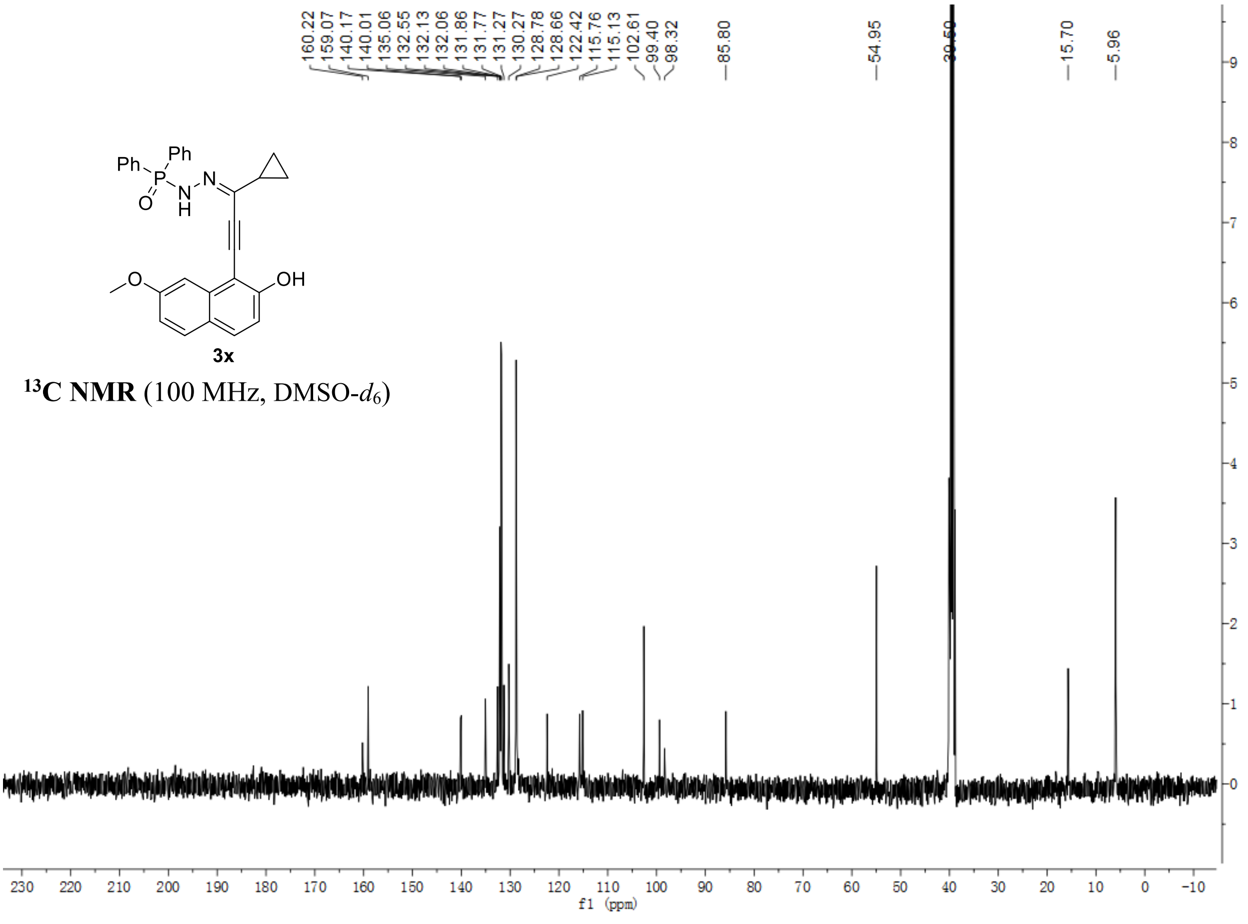

3x

^{13}C NMR (100 MHz, DMSO-d_6)

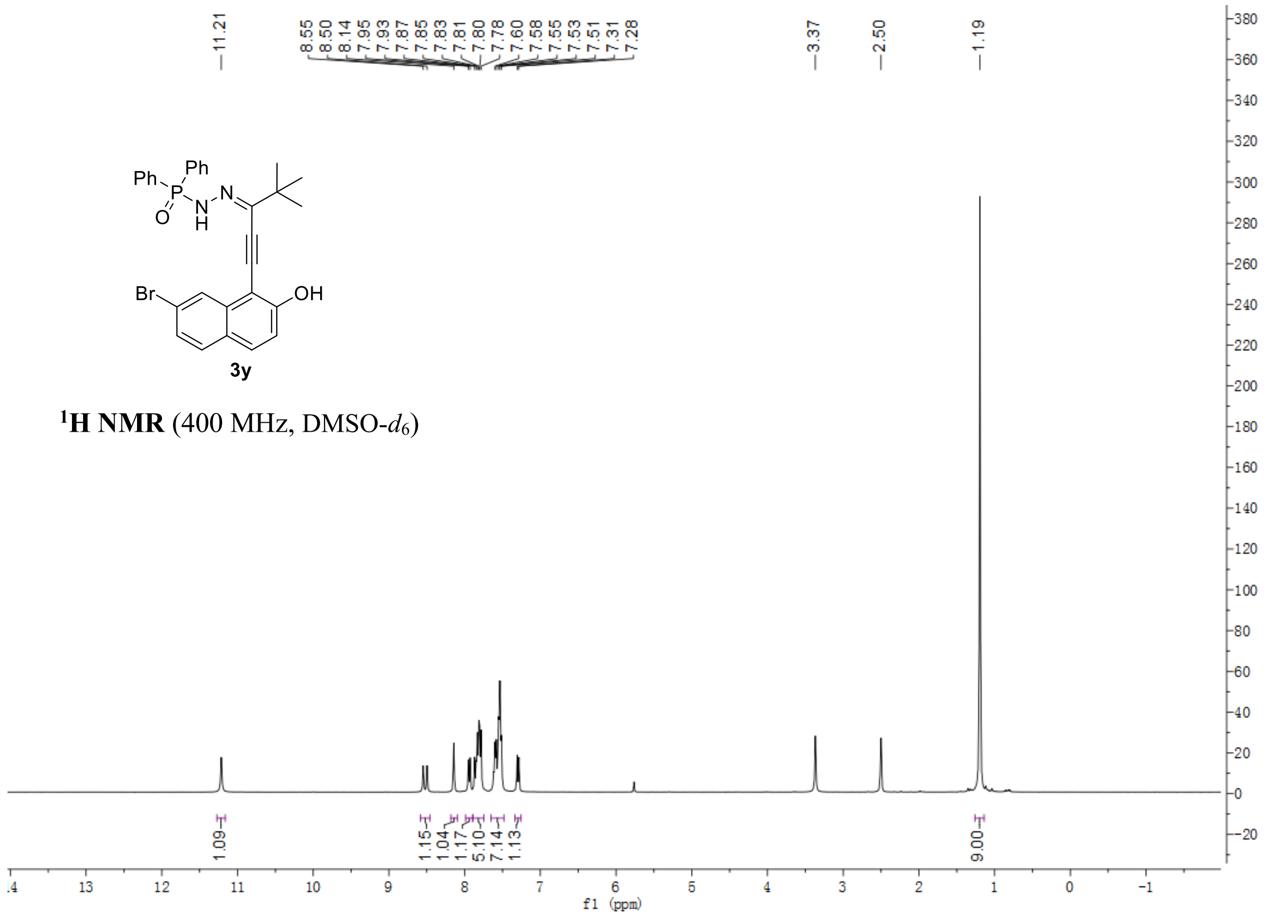

3y

^{1}H NMR (400 MHz, DMSO-d_6)

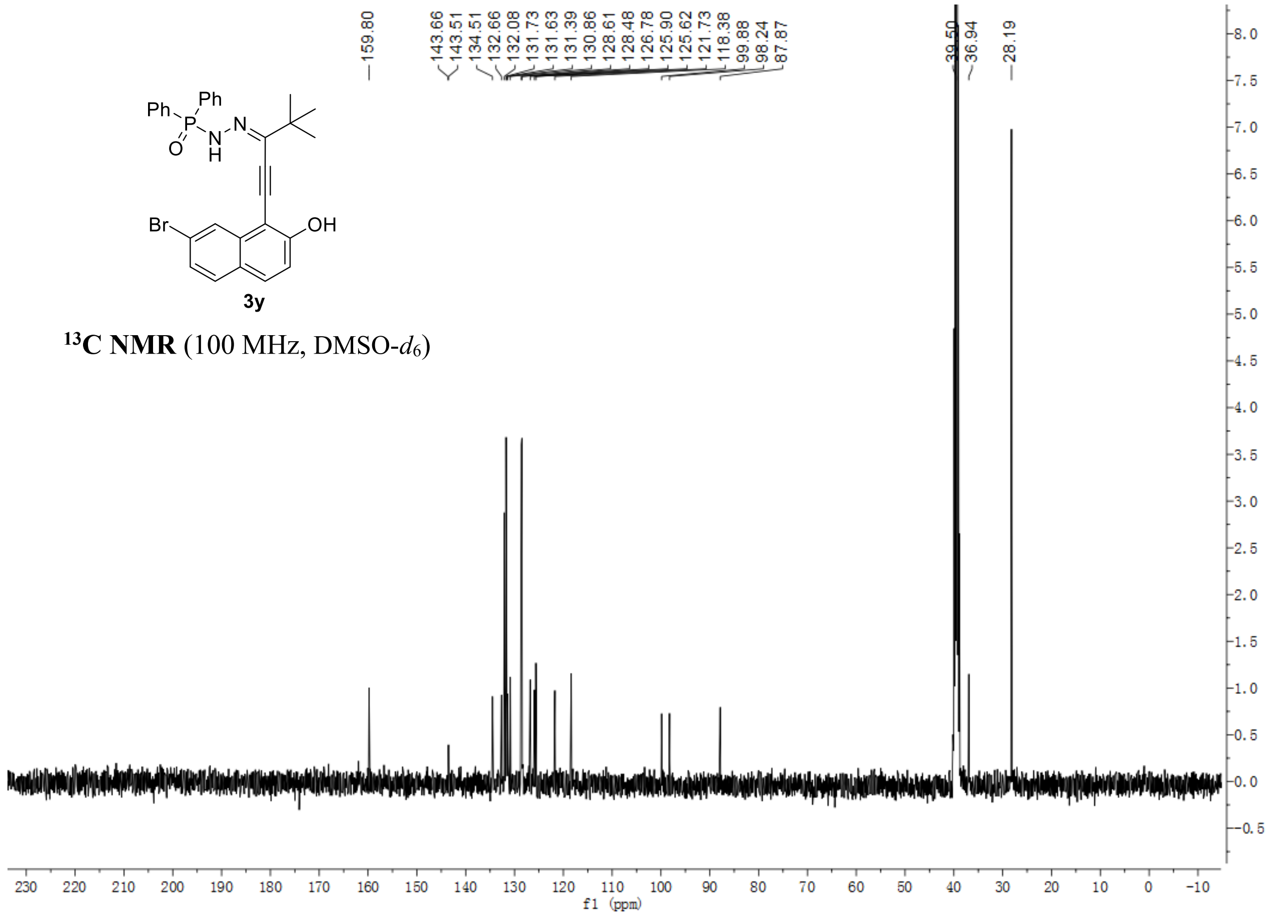

3y

^{13}C NMR (100 MHz, DMSO-d_6)

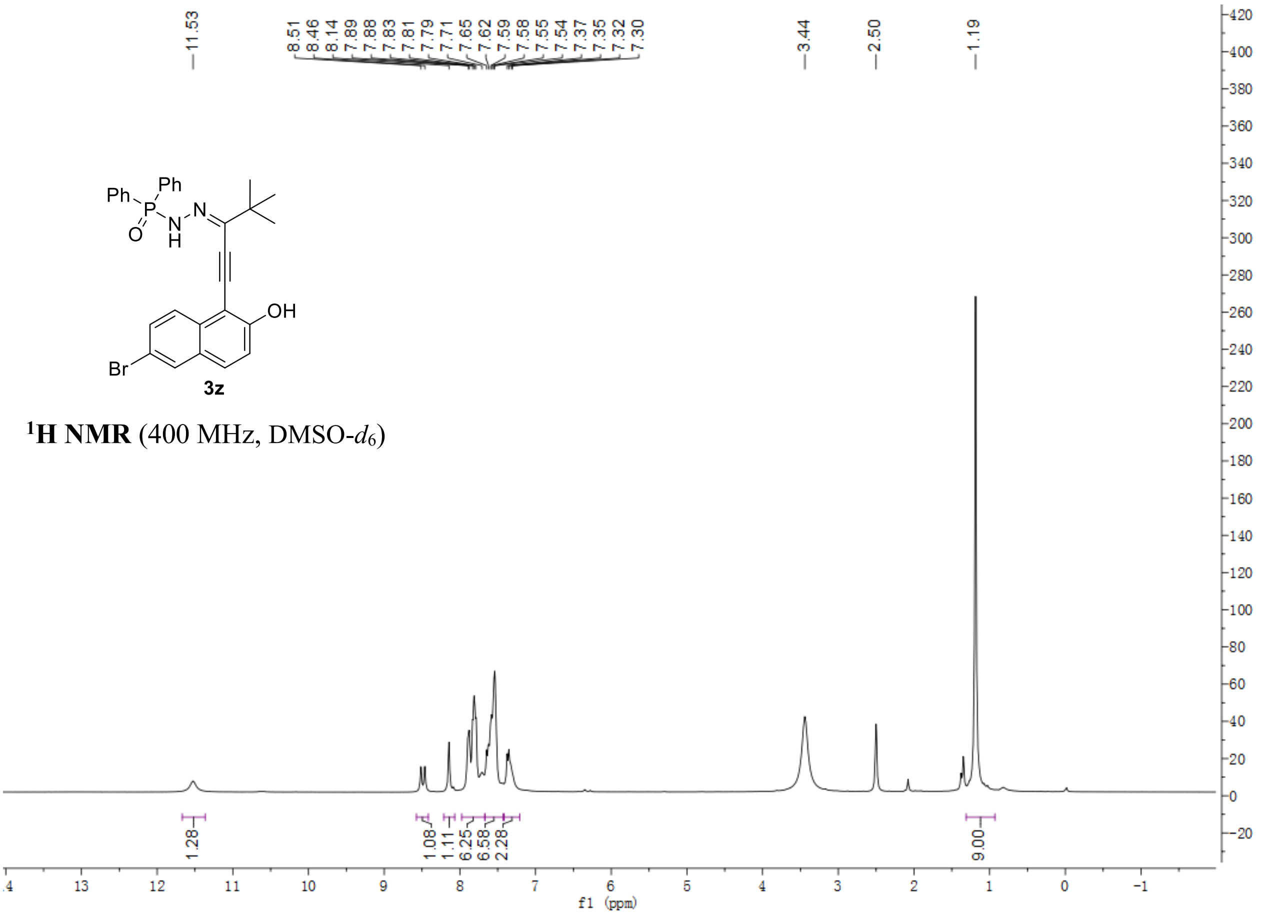

^{1}H NMR (400 MHz, DMSO-*d*$_6$)

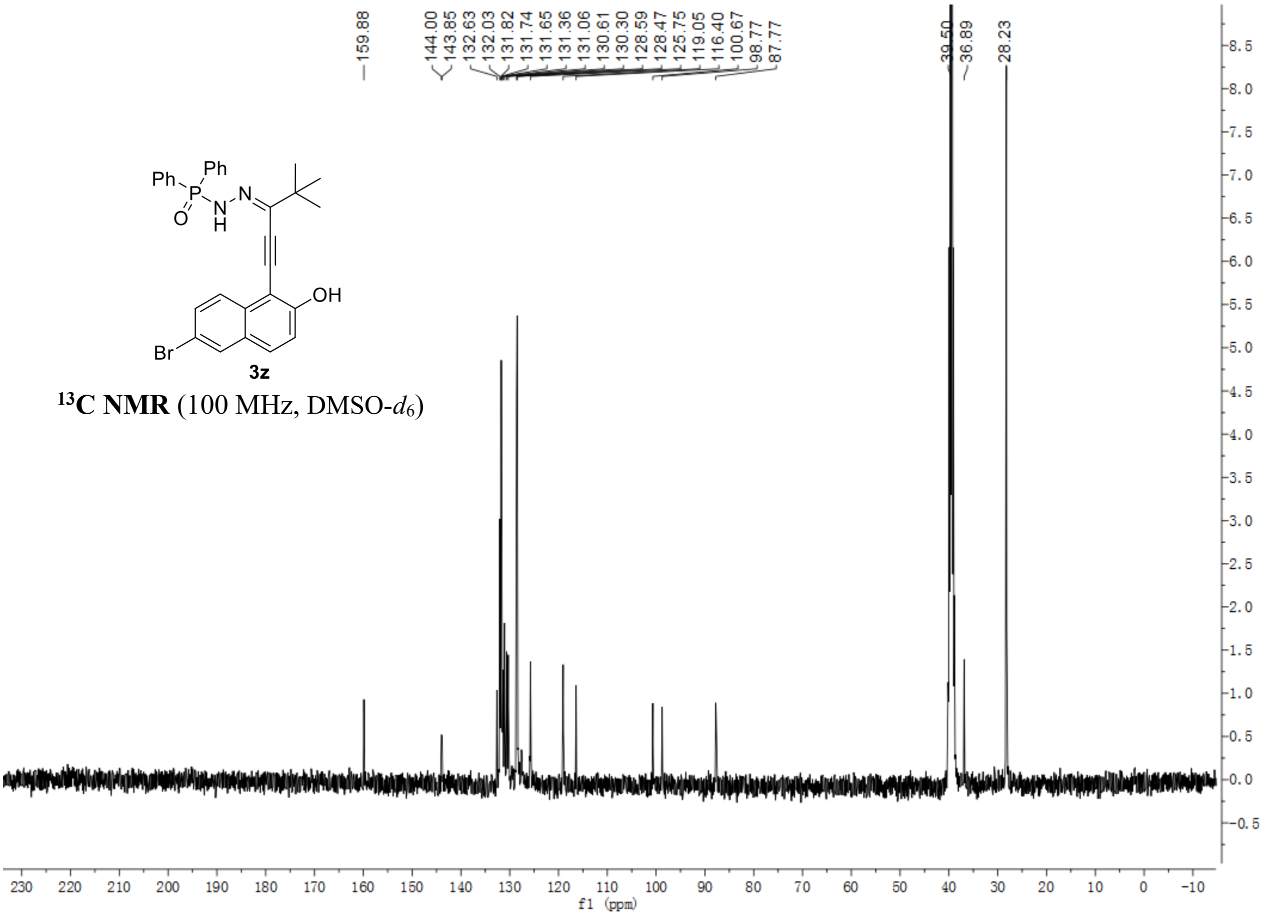

^{13}C NMR (100 MHz, DMSO-*d*$_6$)

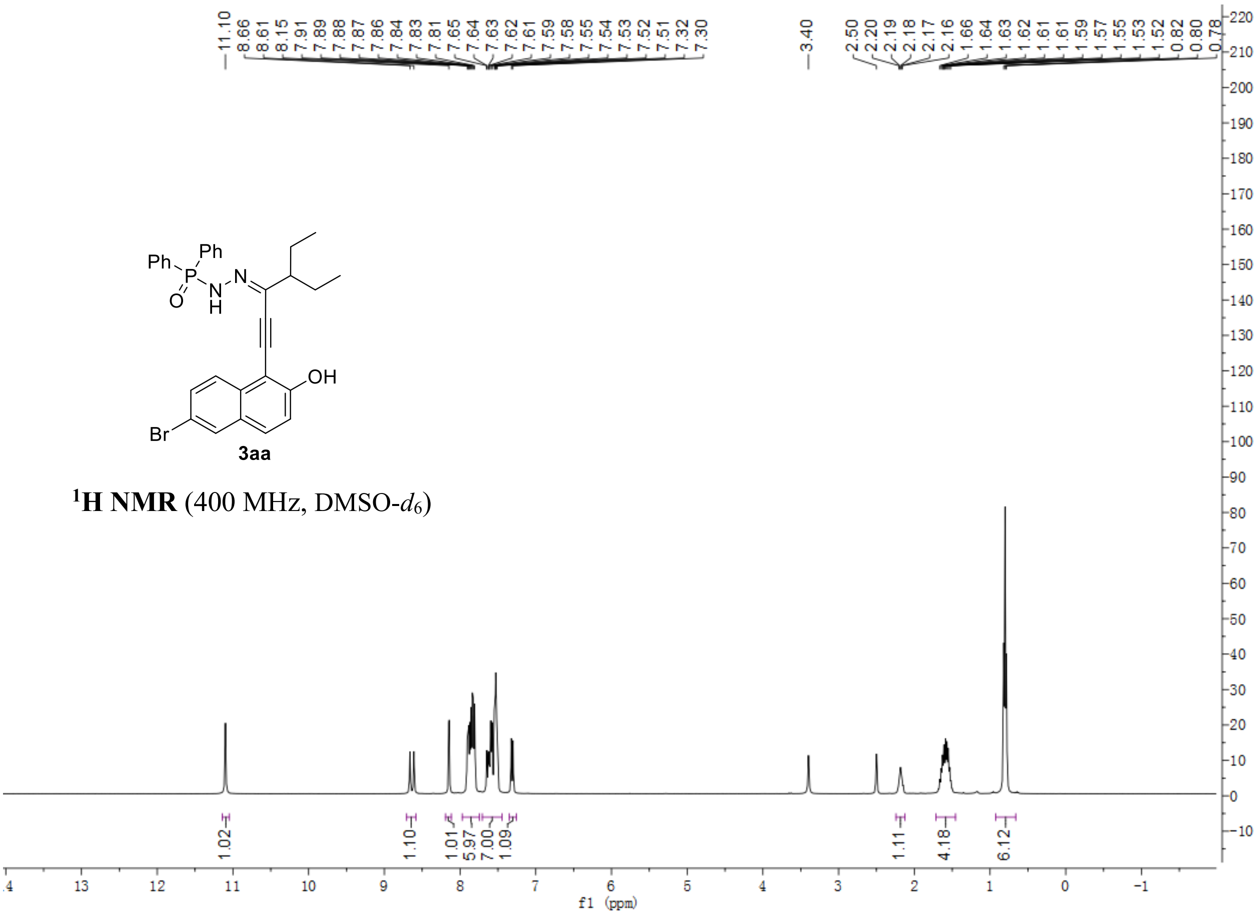

^{1}H NMR (400 MHz, DMSO-d_6)

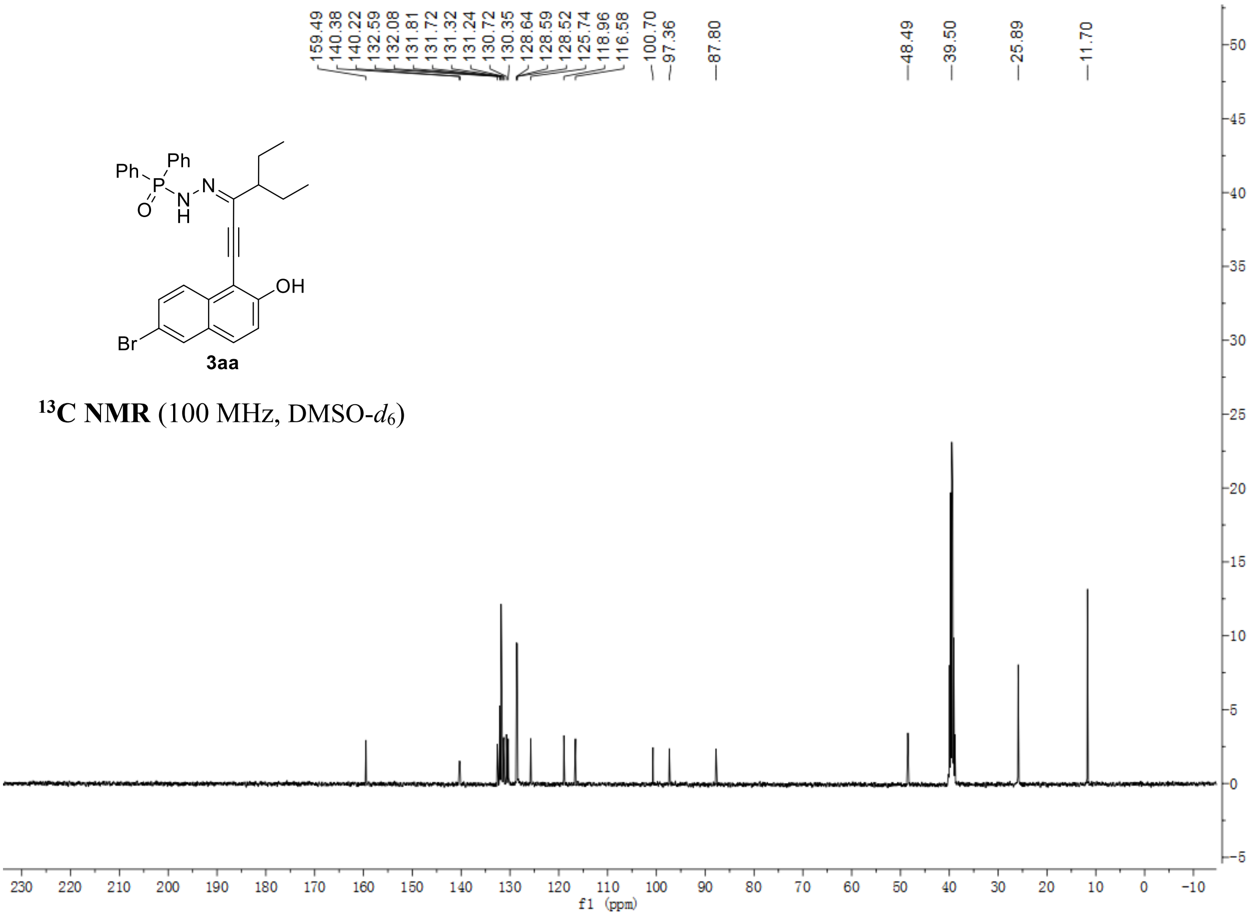

^{13}C NMR (100 MHz, DMSO-d_6)

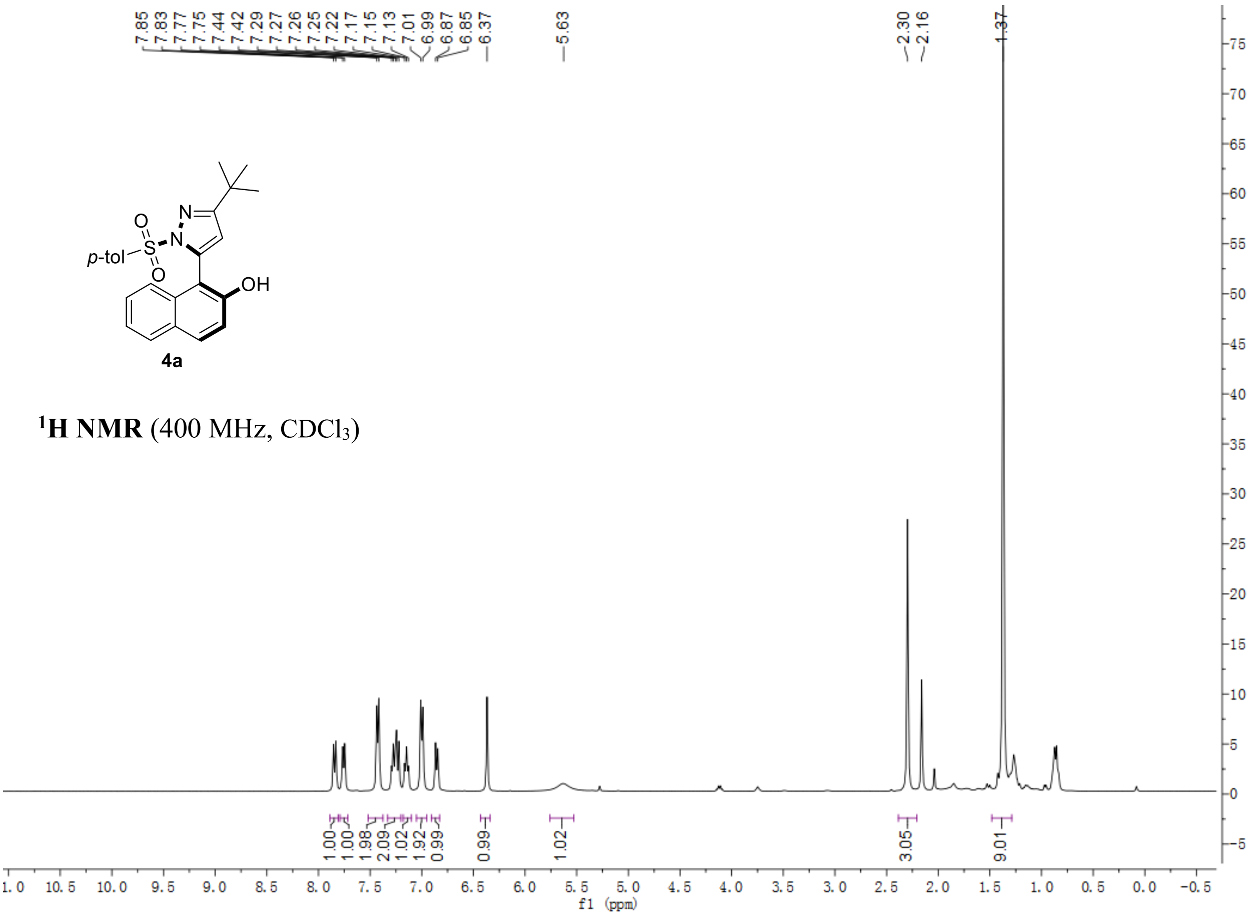

^{1}H NMR (400 MHz, $CDCl_3$)

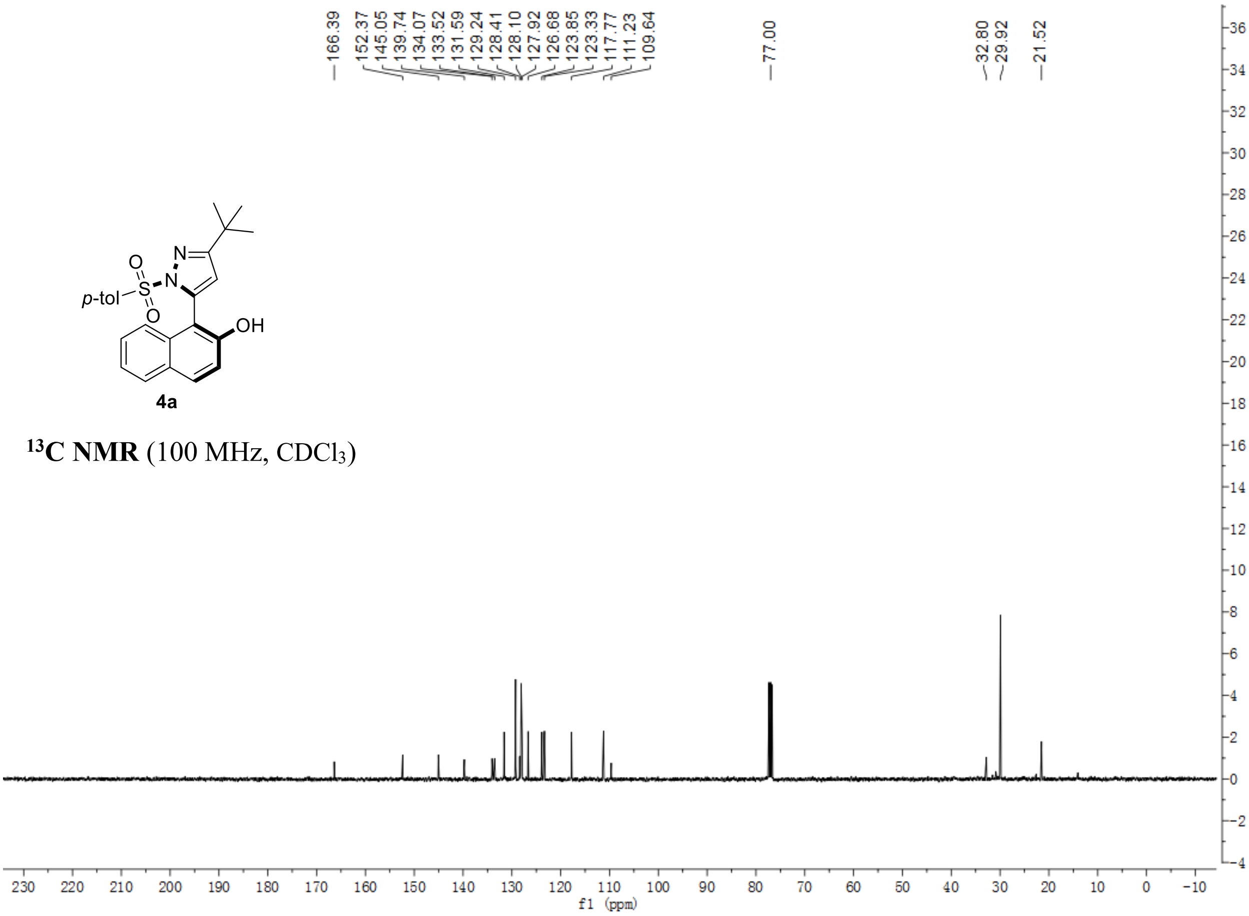

^{13}C NMR (100 MHz, $CDCl_3$)

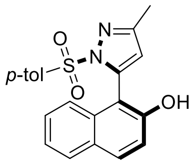

4b

^{1}H NMR (400 MHz, $CDCl_3$)

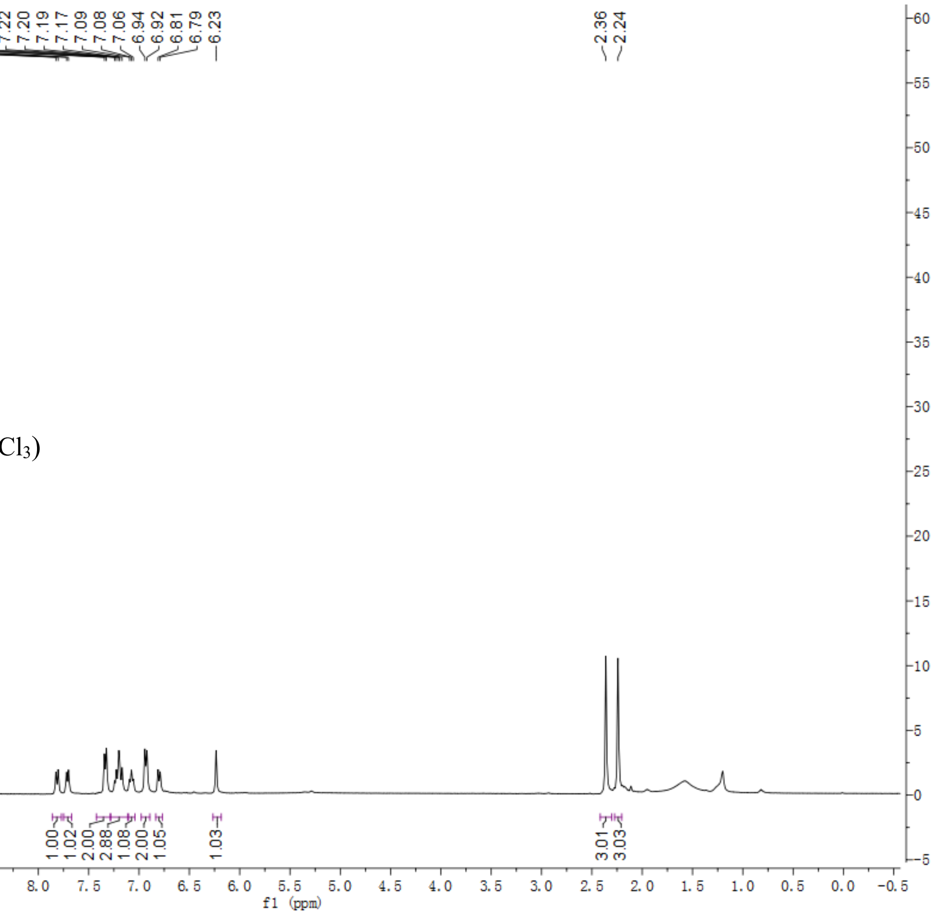

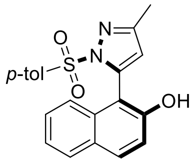

4b

^{13}C NMR (100 MHz, $CDCl_3$)

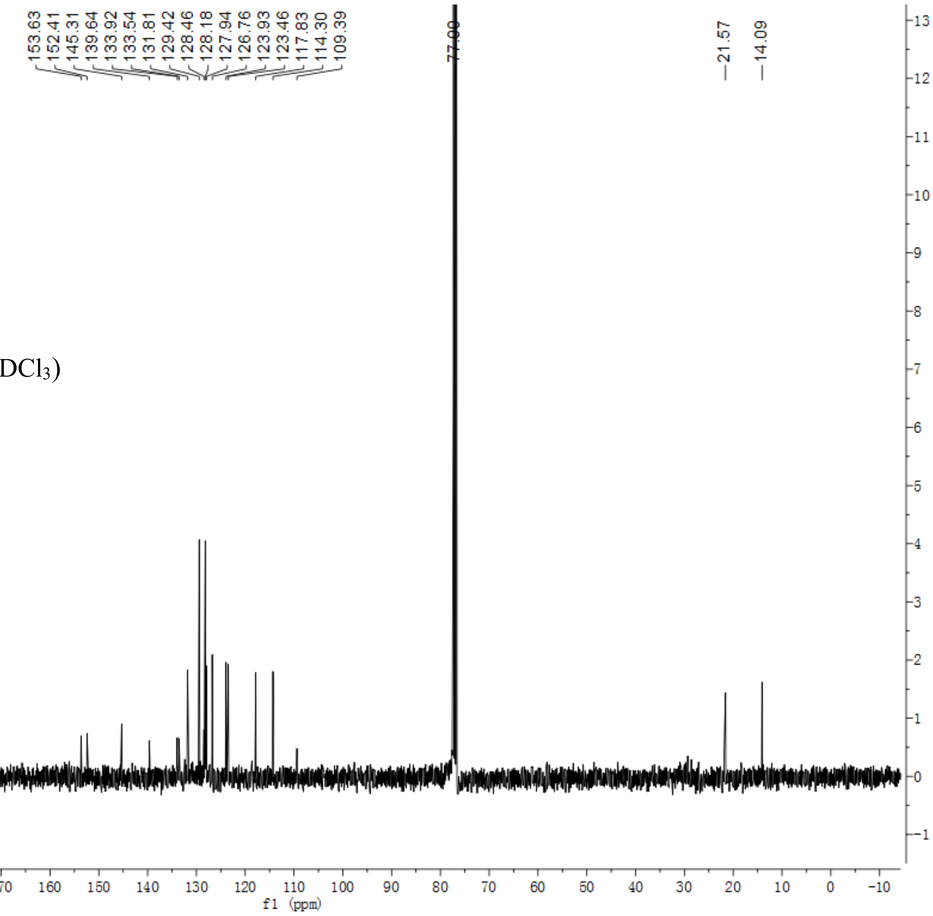

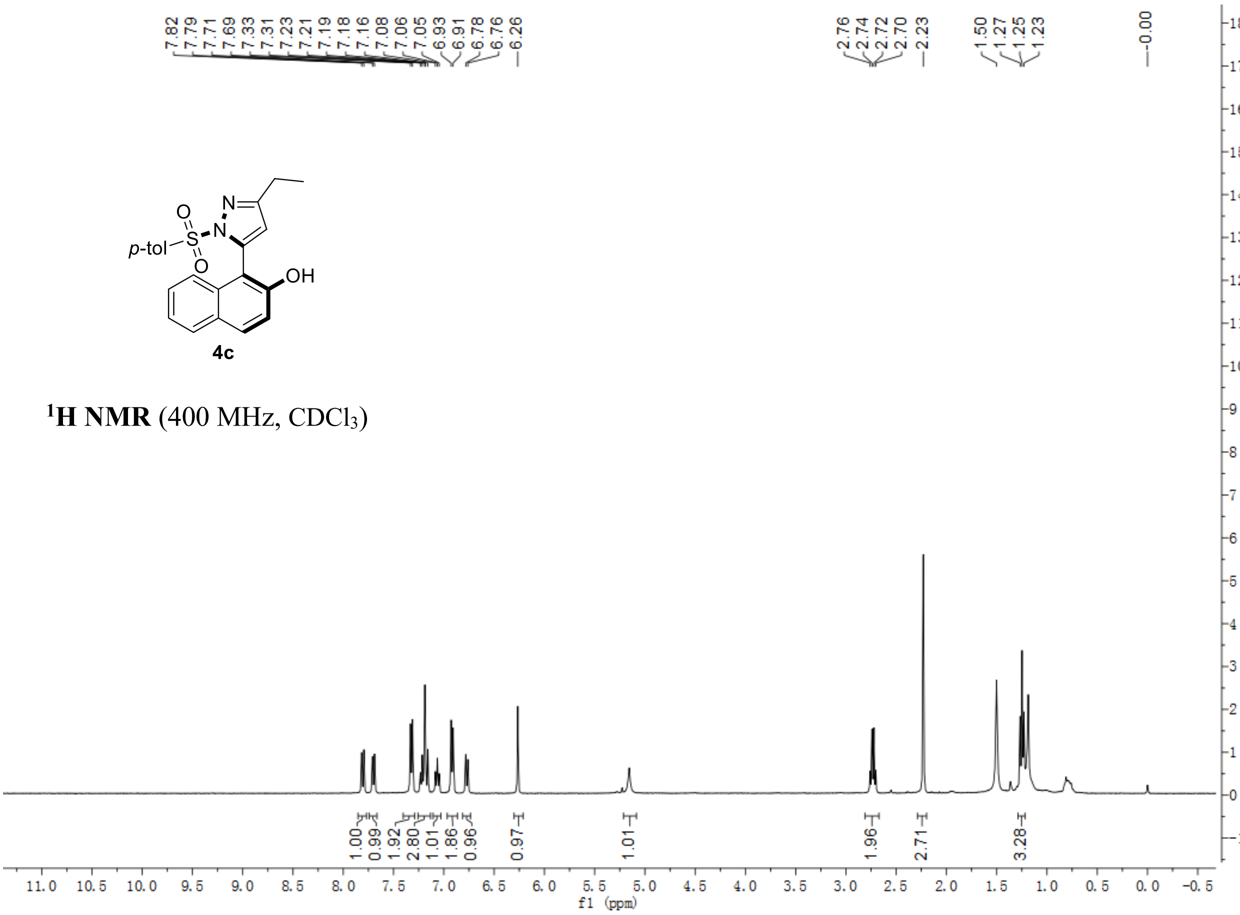
7.82 7.79 7.71 7.69 7.33 7.31 7.23 7.21 7.19 7.18 7.16 7.08 7.06 7.05 6.93 6.91 6.78 6.76 6.26
2.76 2.74 2.72 2.70 2.23
1.50 1.27 1.25 1.23
0.00
p-tol
OH
4c
1H NMR (400 MHz, CDCl3)
1.00 0.99 1.92 2.80 1.01 1.86 0.96 0.97 1.01 1.96 2.71 3.28
f1 (ppm)

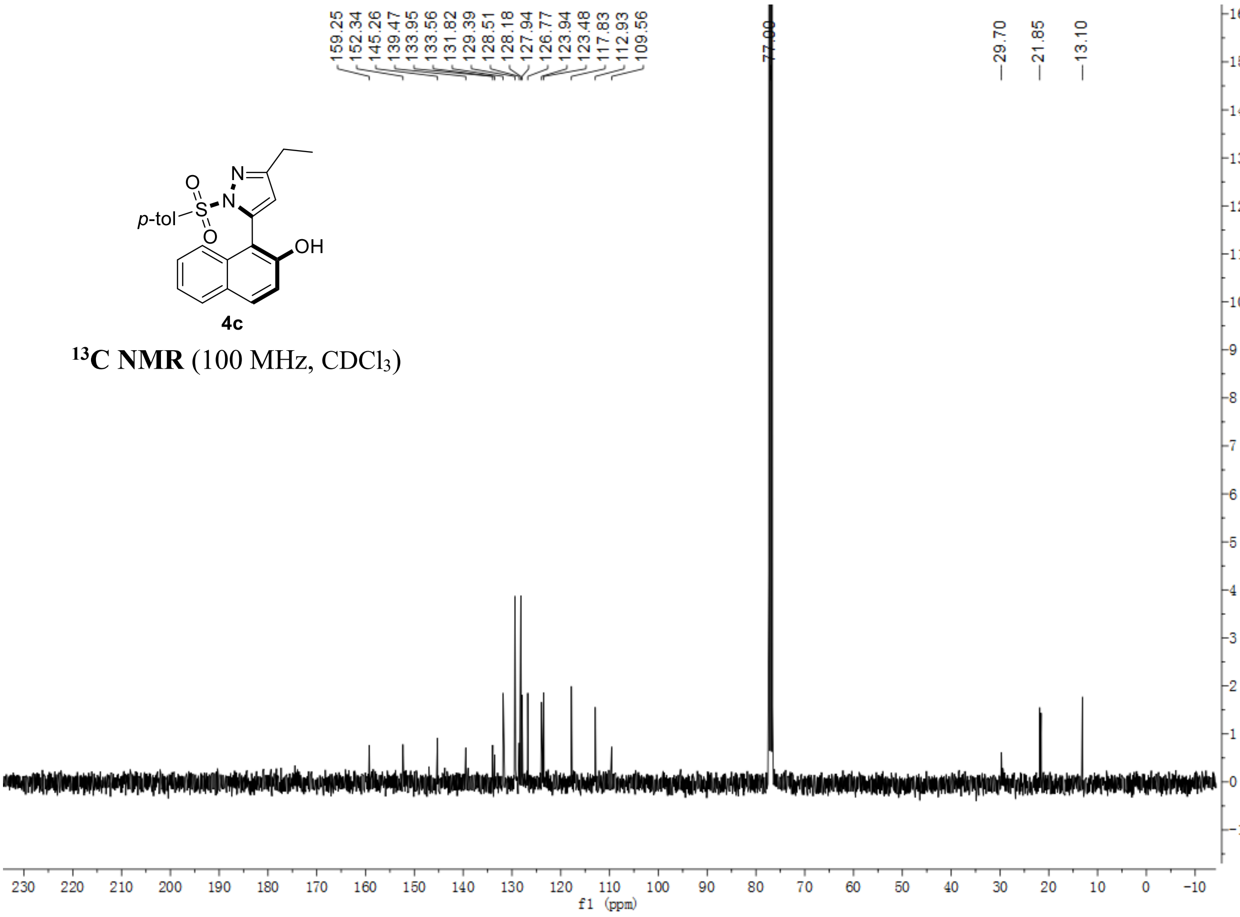
159.25 152.34 145.26 139.47 133.95 133.56 131.82 129.39 128.51 128.18 127.94 126.77 123.94 123.48 117.83 112.93 109.56
77.00
29.70 21.85 13.10
p-tol
OH
4c
13C NMR (100 MHz, CDCl3)
f1 (ppm)

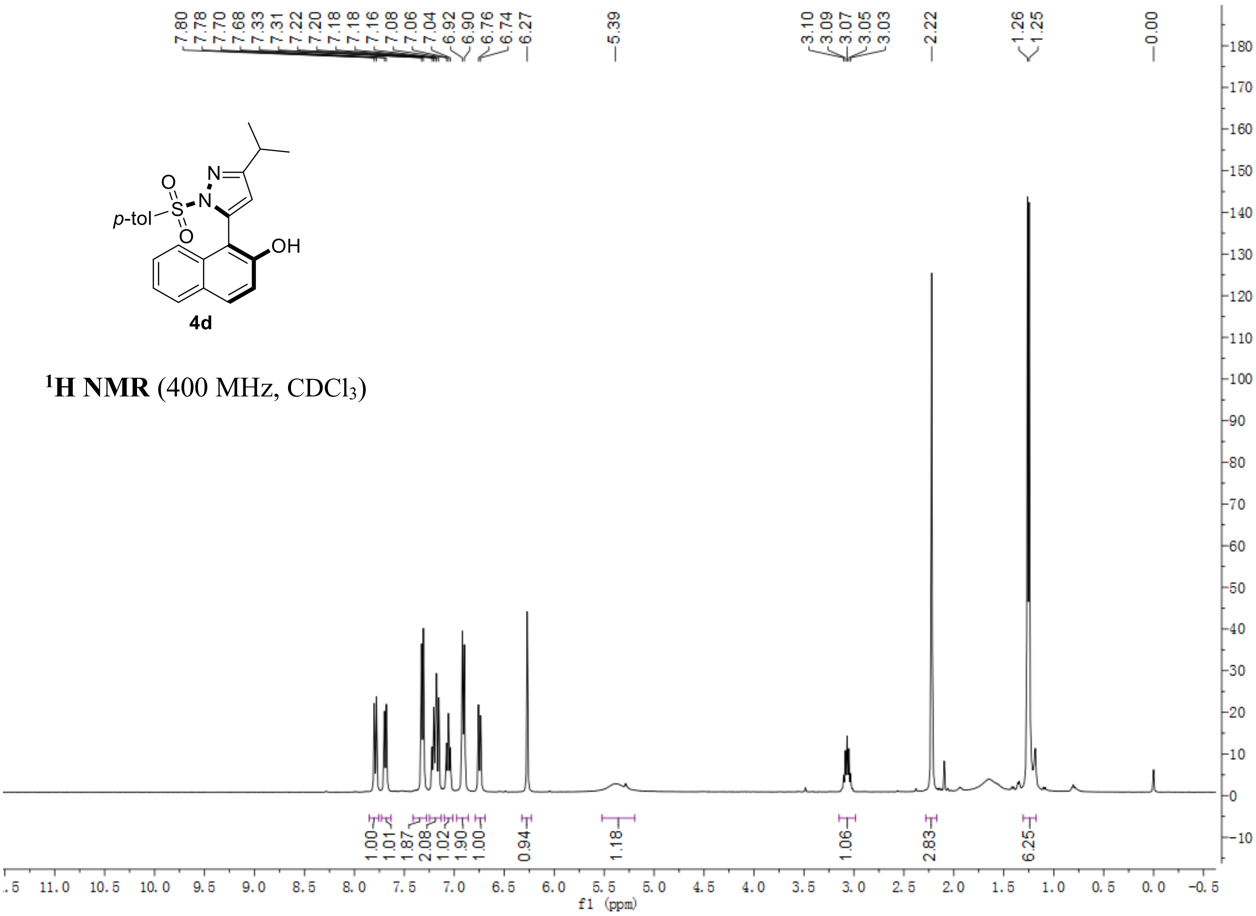

4d

^{1}H NMR (400 MHz, $CDCl_3$)

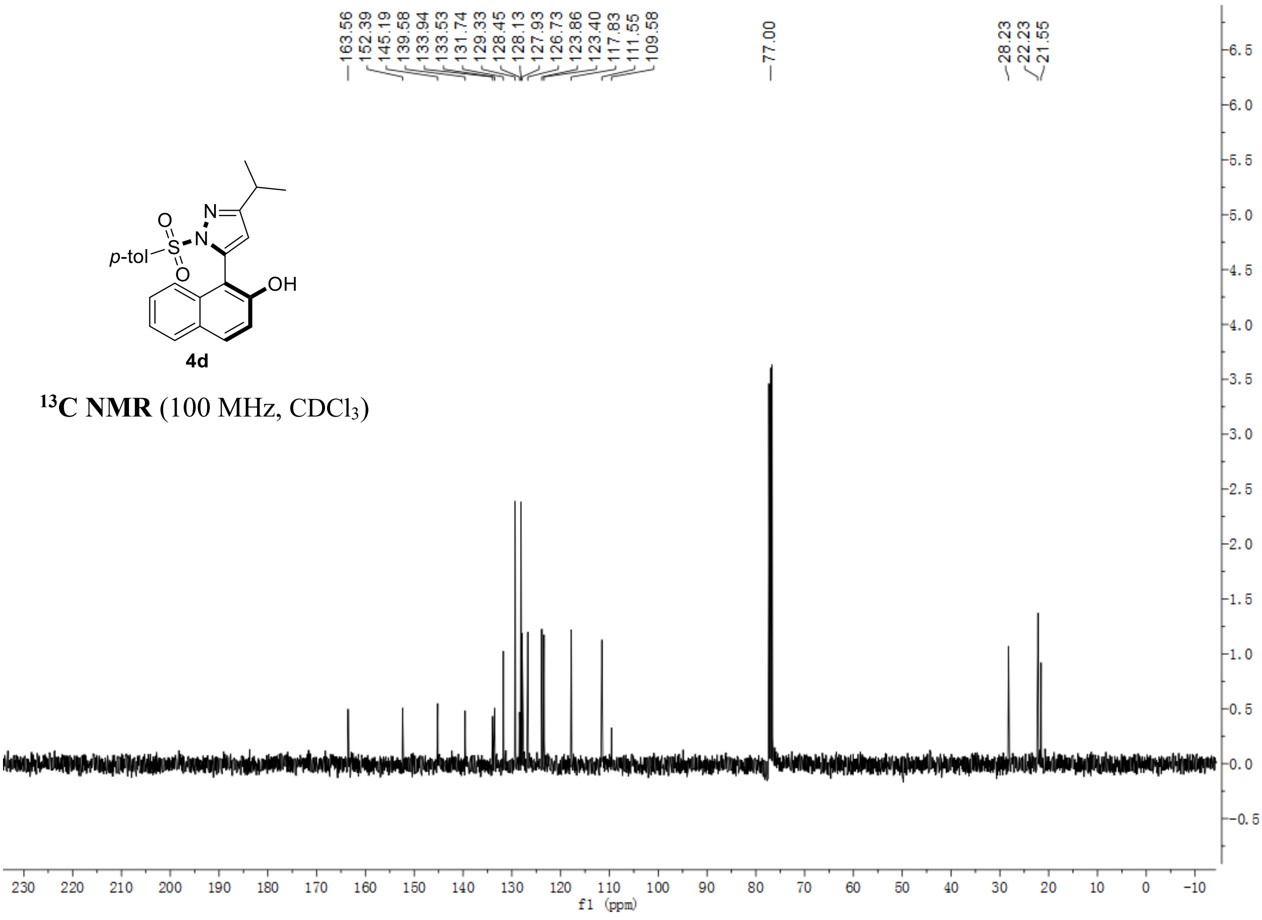

4d

^{13}C NMR (100 MHz, $CDCl_3$)

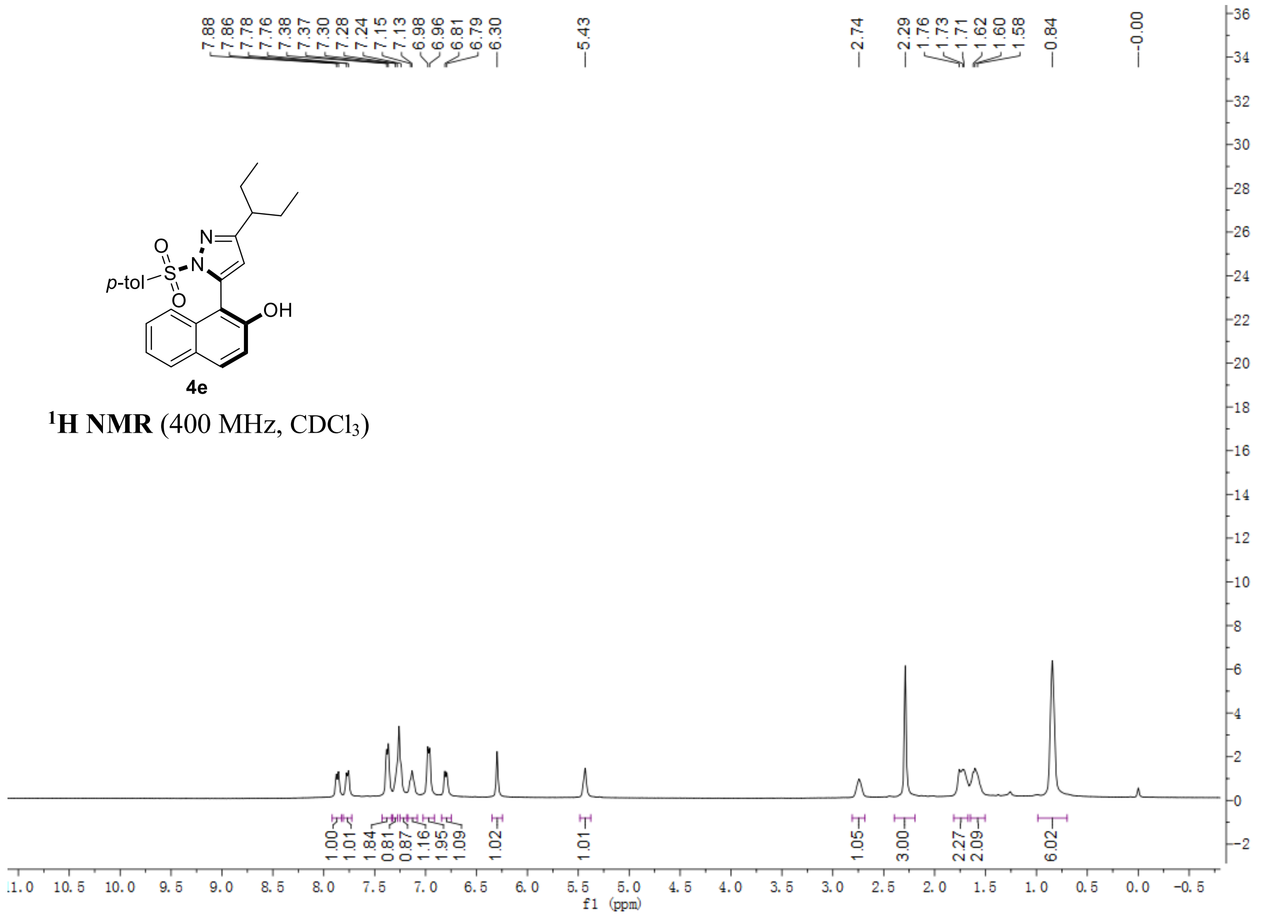

4e

^{1}H NMR (400 MHz, $CDCl_3$)

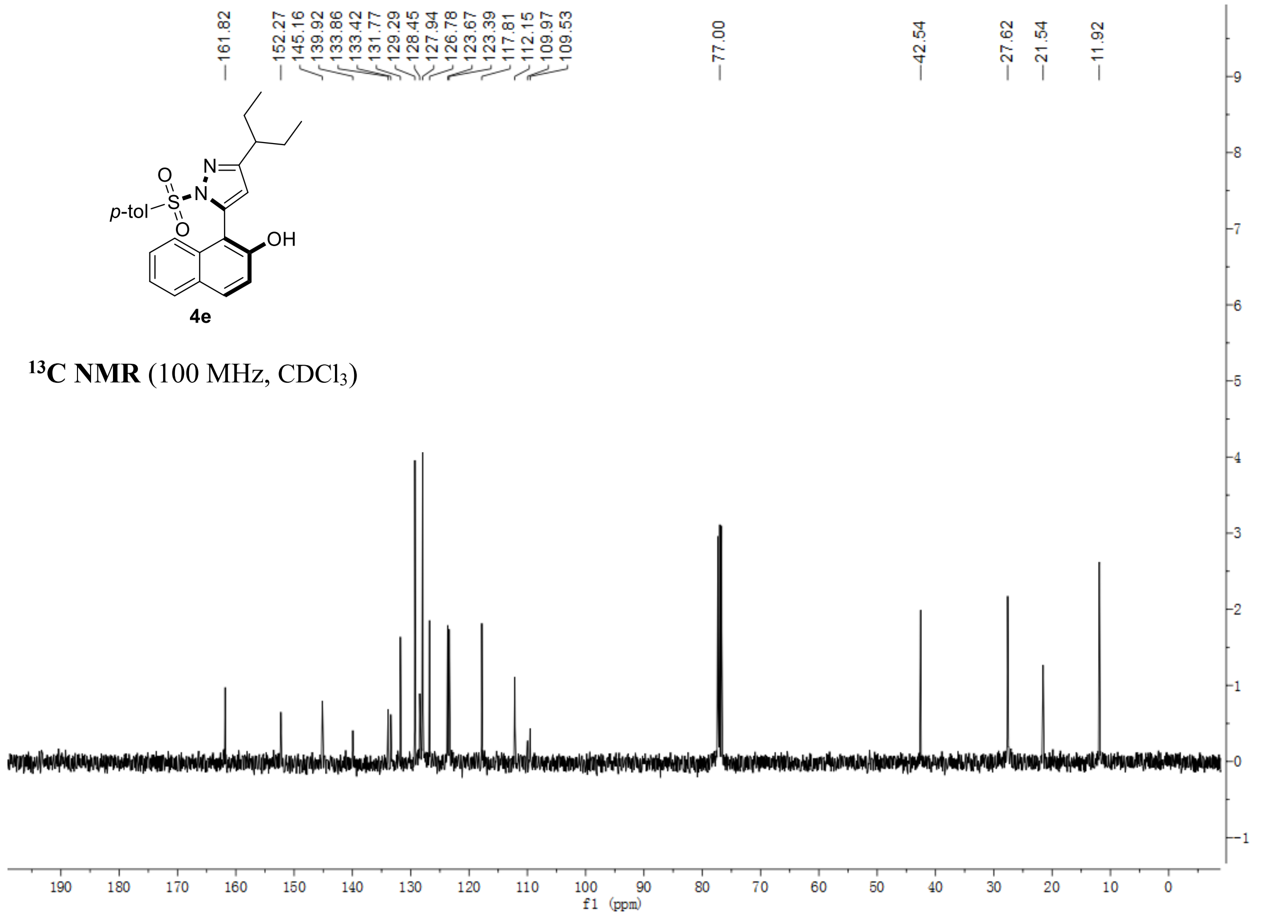

4e

^{13}C NMR (100 MHz, $CDCl_3$)

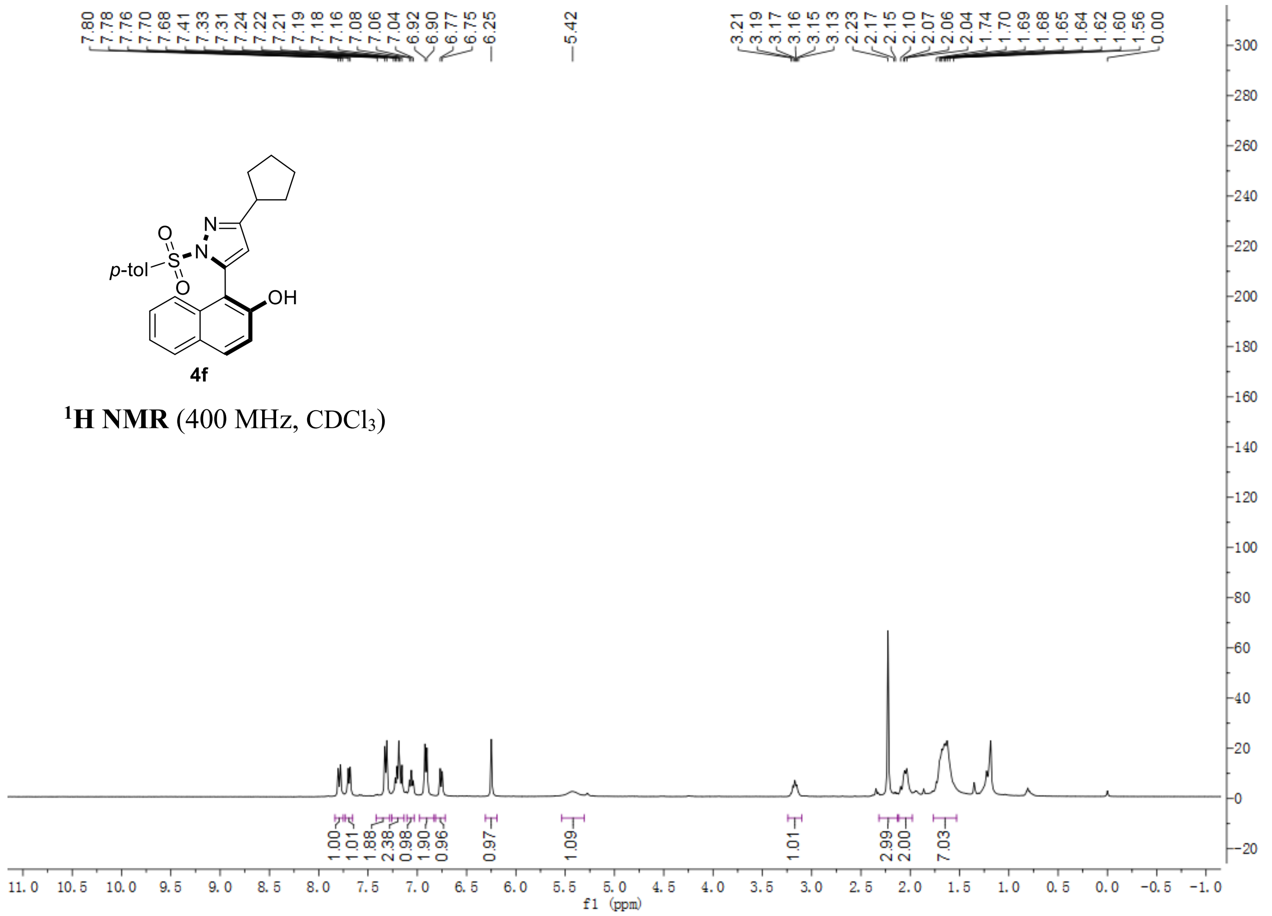

4f

^{1}H NMR (400 MHz, $CDCl_3$)

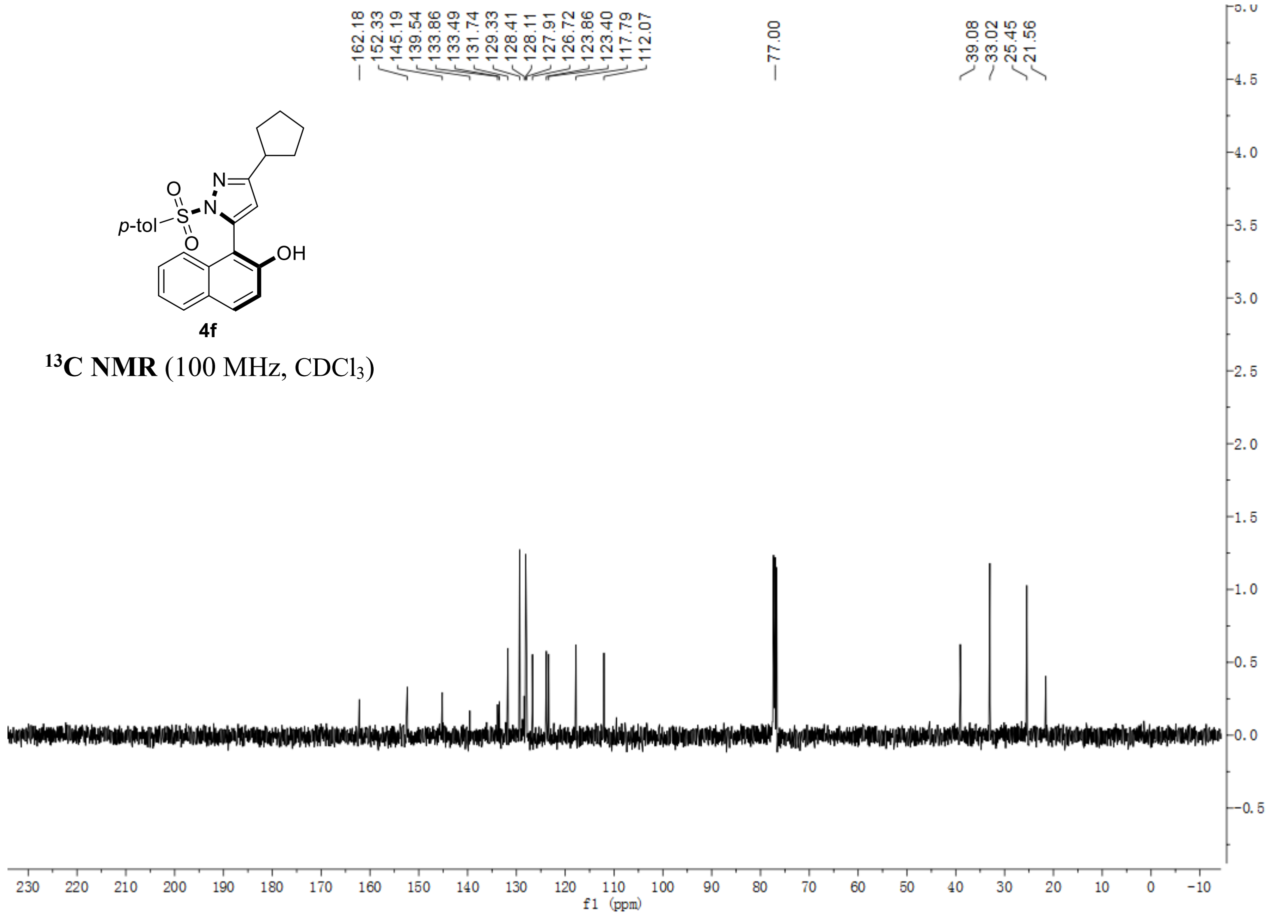

4f

^{13}C NMR (100 MHz, $CDCl_3$)

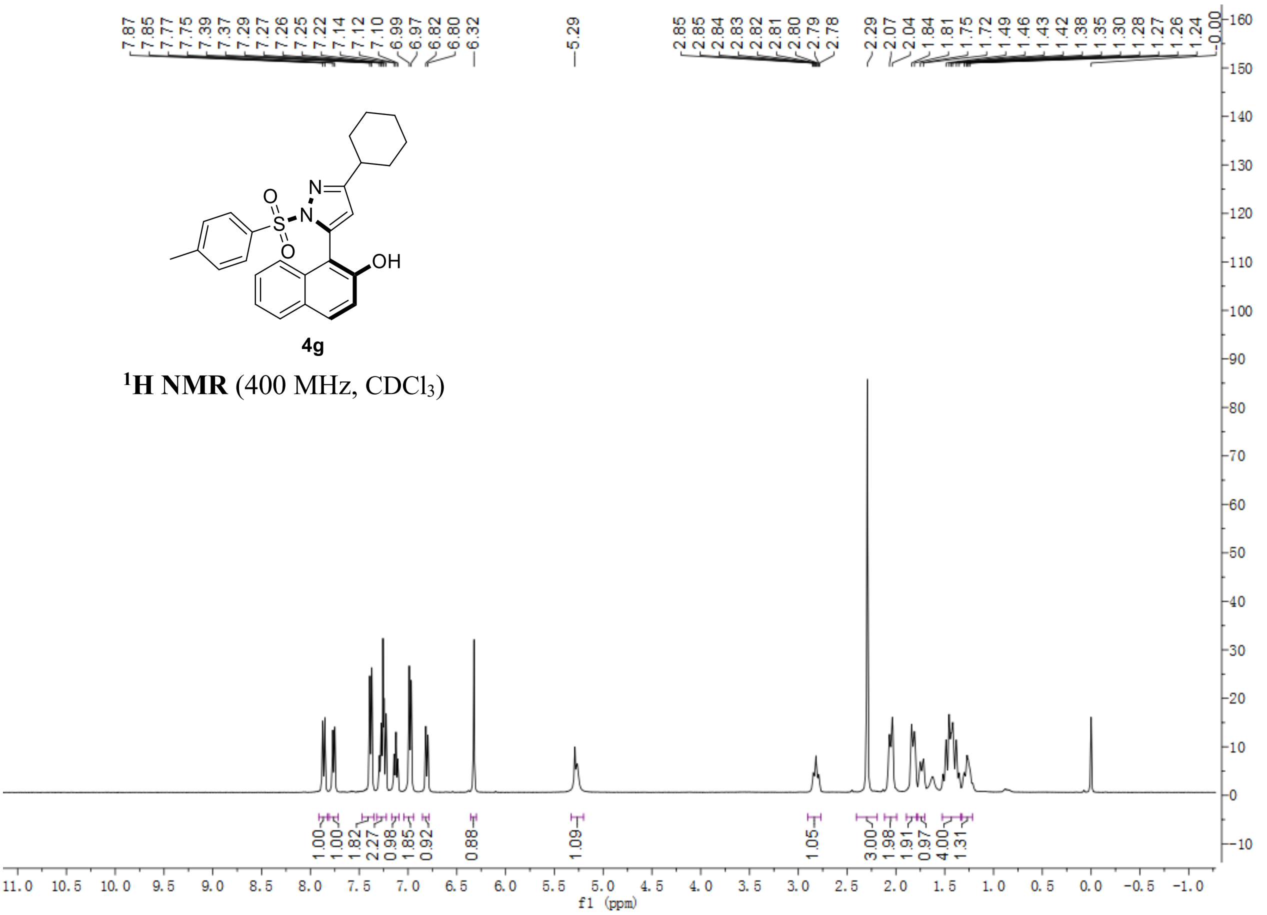
7.87 7.85 7.77 7.75 7.39 7.37 7.29 7.27 7.26 7.25 7.22 7.14 7.12 7.10 6.99 6.97 6.82 6.80 6.32
5.29
2.85 2.85 2.84 2.83 2.82 2.81 2.80 2.79 2.78
2.29 2.07 2.04 1.84 1.81 1.75 1.72 1.49 1.46 1.43 1.42 1.38 1.35 1.30 1.28 1.27 1.26 1.24 0.00
4g
1H NMR (400 MHz, CDCl3)
1.00 1.00 1.82 2.27 0.98 1.85 0.92 0.88 1.09 1.05 3.00 1.98 1.91 0.97 4.00 1.31
f1 (ppm)

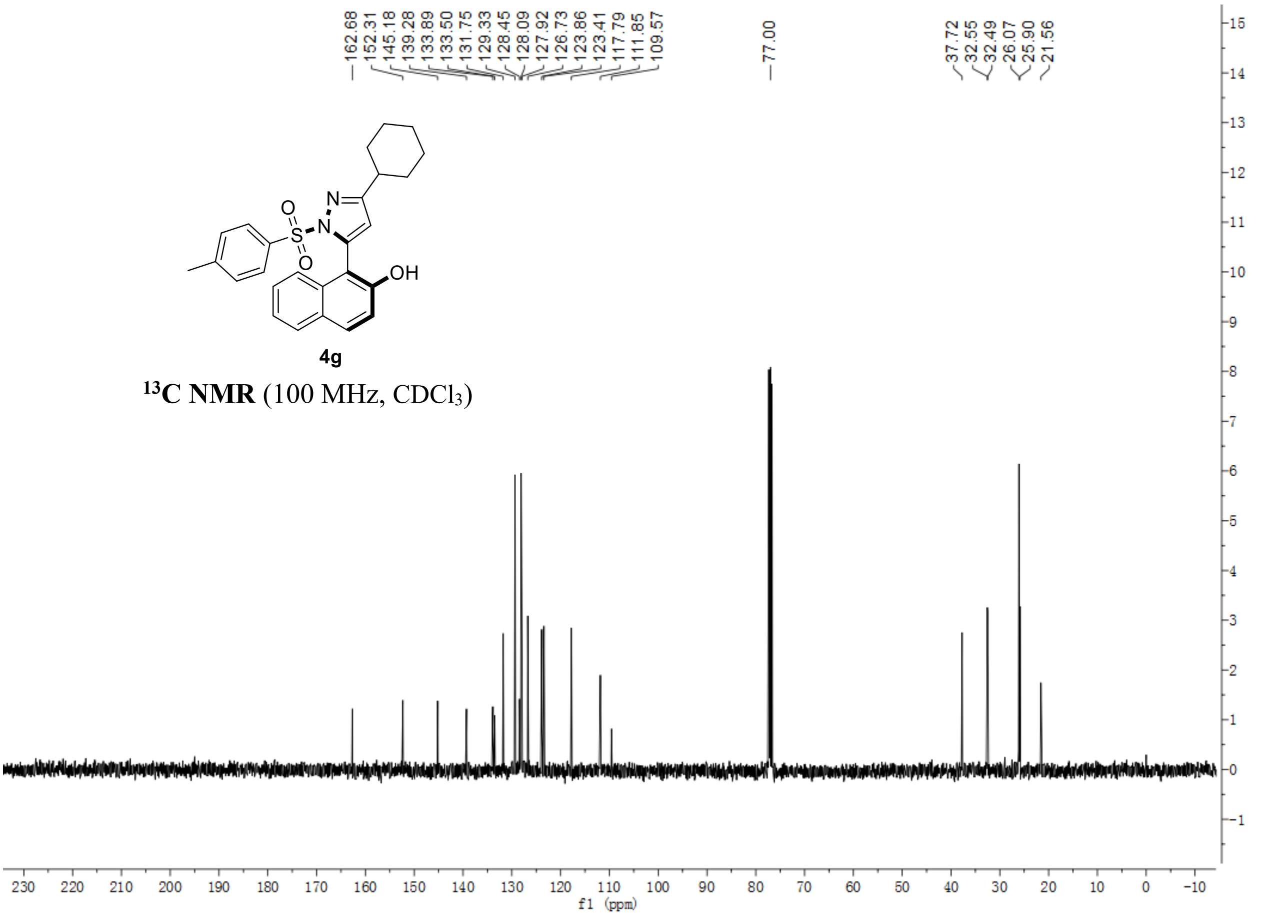
162.68 152.31 145.18 139.28 133.89 133.50 131.75 129.33 128.45 128.09 127.92 126.73 123.86 123.41 117.79 111.85 109.57
77.00
37.72 32.55 32.49 26.07 25.90 21.56
4g
13C NMR (100 MHz, CDCl3)
f1 (ppm)

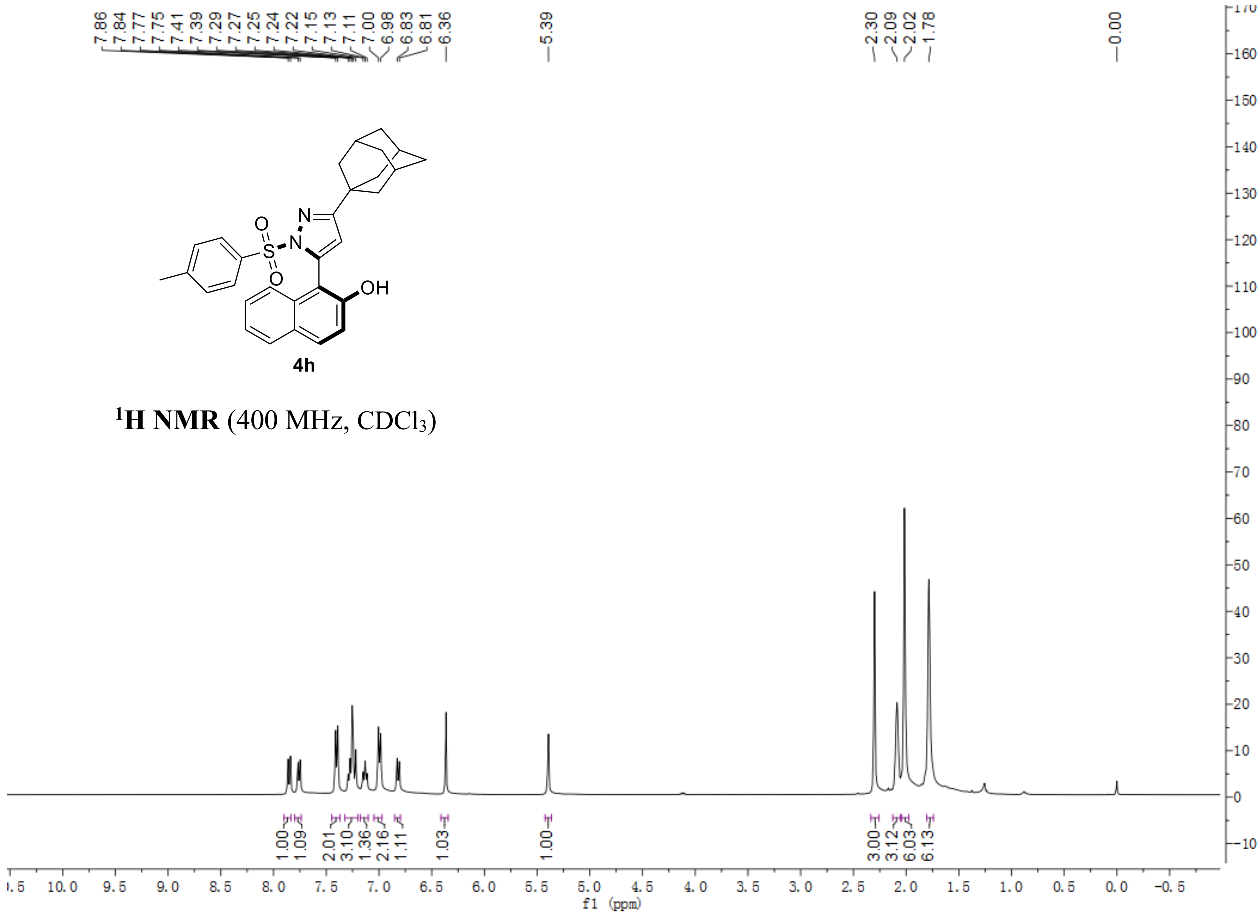
7.86 7.84 7.77 7.75 7.41 7.39 7.29 7.27 7.25 7.24 7.22 7.15 7.13 7.11 7.00 6.98 6.83 6.81 6.36
5.39
2.30 2.09 2.02 1.78
0.00
4h
1H NMR (400 MHz, CDCl3)
1.00 1.09 2.01 3.10 1.36 2.16 1.11 1.03 1.00 3.00 3.12 6.03 6.13
f1 (ppm)

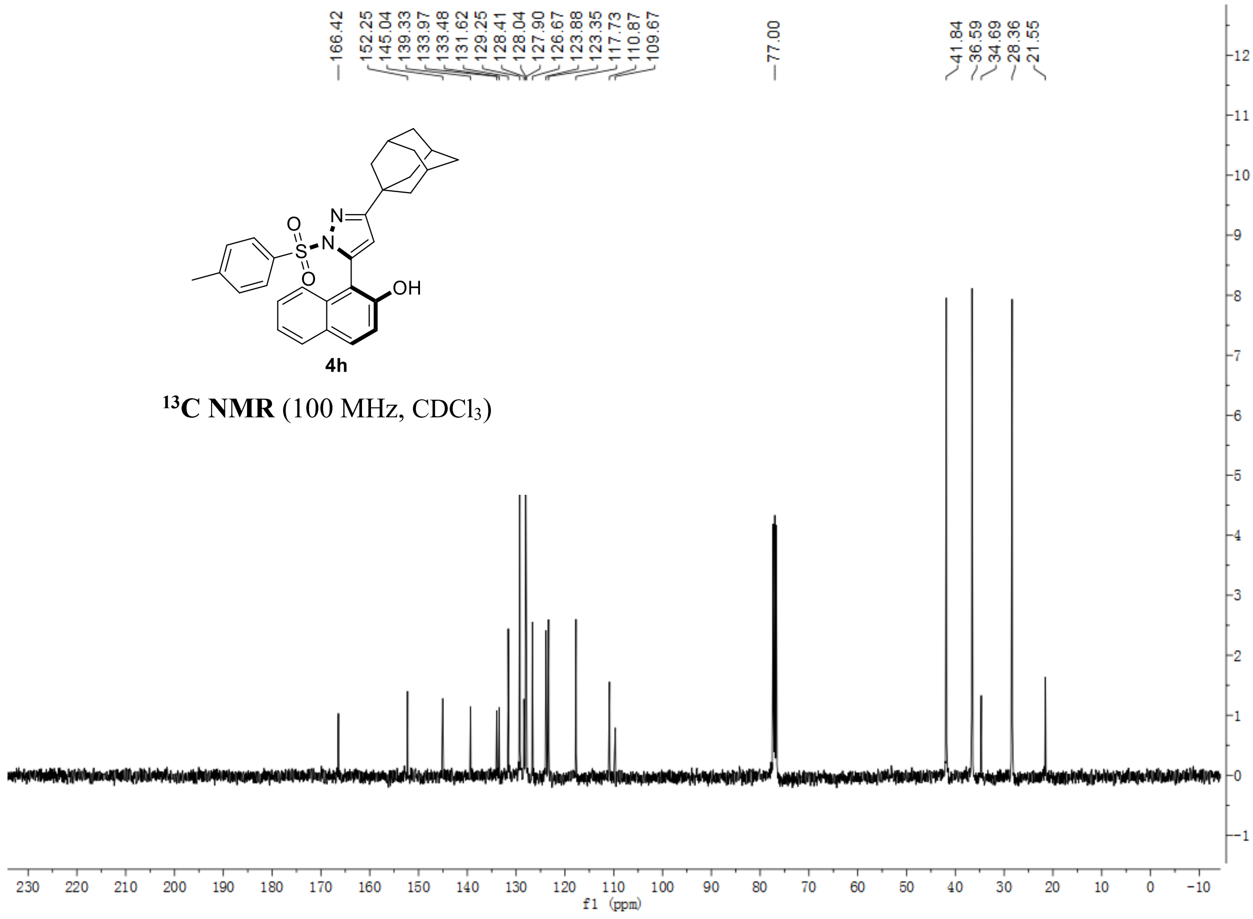
166.42
152.25 145.04 139.33 133.97 133.48 131.62 129.25 128.41 128.04 127.90 126.67 123.88 123.35 117.73 110.87 109.67
77.00
41.84 36.59 34.69 28.36 21.55
4h
13C NMR (100 MHz, CDCl3)
f1 (ppm)

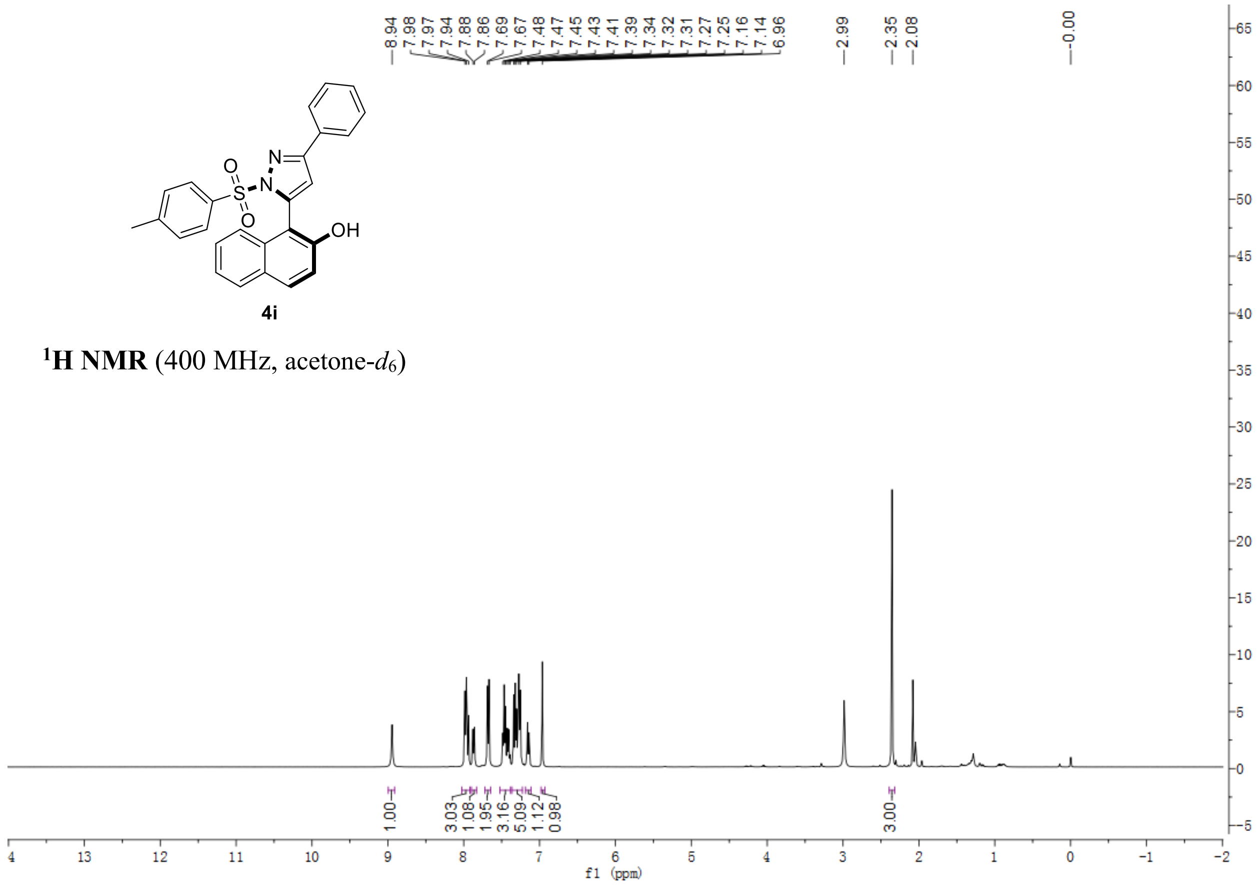
8.94
7.98
7.97
7.94
7.88
7.86
7.69
7.67
7.48
7.47
7.45
7.43
7.41
7.39
7.34
7.32
7.31
7.27
7.25
7.16
7.14
6.96
2.99
2.35
2.08
0.00
N
O
S
N
O
OH
4i
1H NMR (400 MHz, acetone-d6)
1.00
3.03
1.08
1.95
3.16
5.09
1.12
0.98
3.00
f1 (ppm)

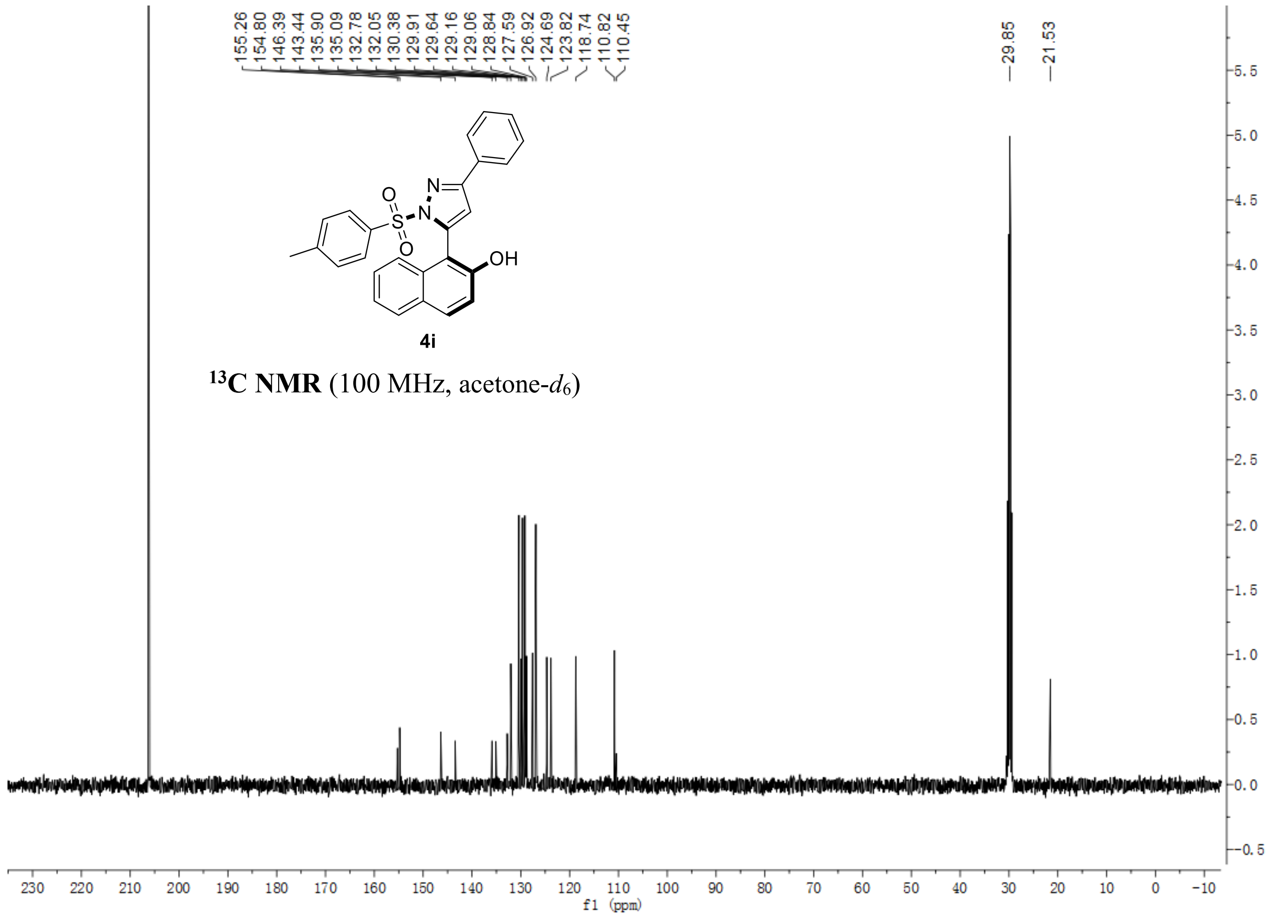
155.26
154.80
146.39
143.44
135.90
135.09
132.78
132.05
130.38
129.91
129.64
129.16
129.06
128.84
127.59
126.92
124.69
123.82
118.74
110.82
110.45
29.85
21.53
N
O
S
N
O
OH
4i
13C NMR (100 MHz, acetone-d6)
f1 (ppm)

4j

^{1}H NMR (400 MHz, $CDCl_3$)

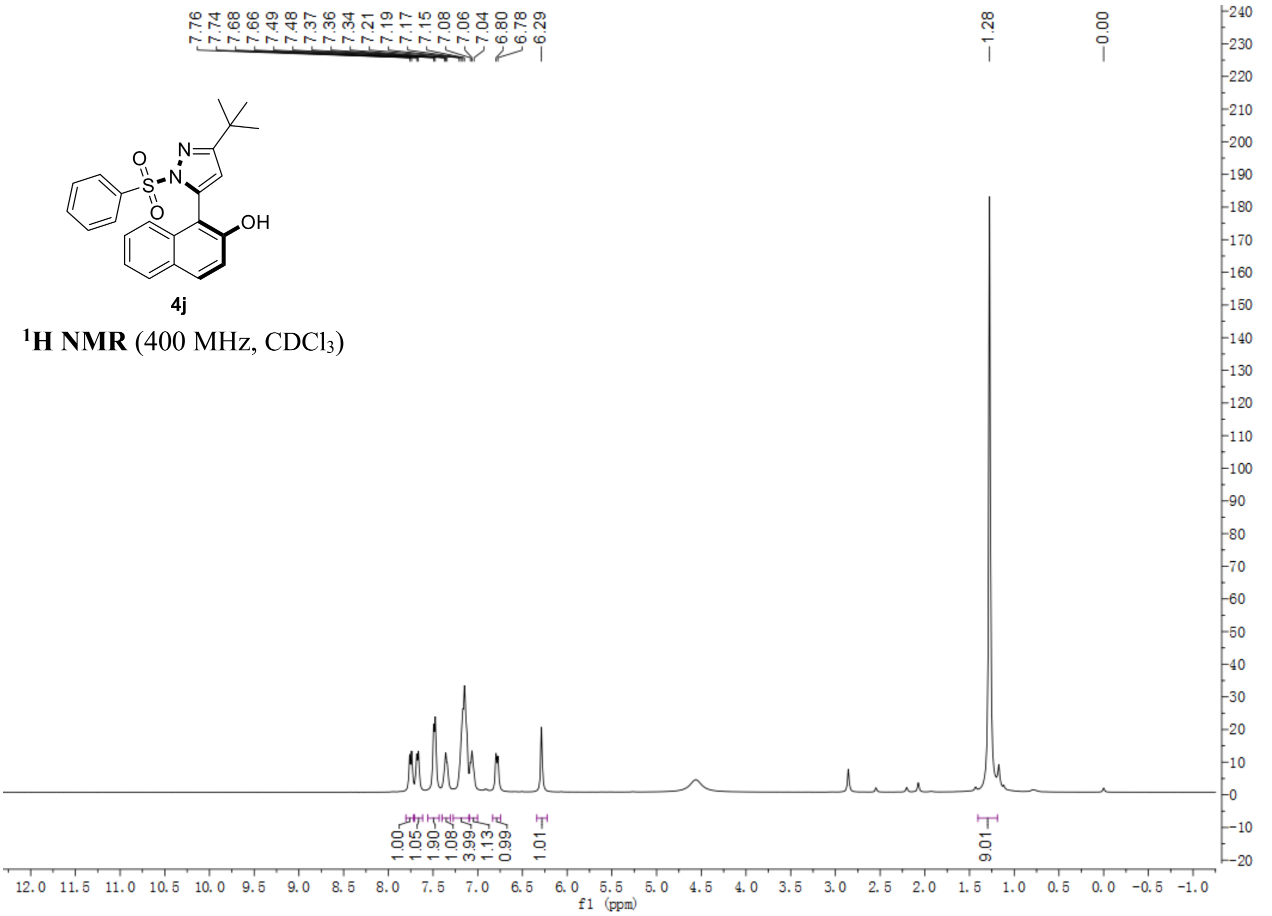

4j

^{13}C NMR (100 MHz, $CDCl_3$)

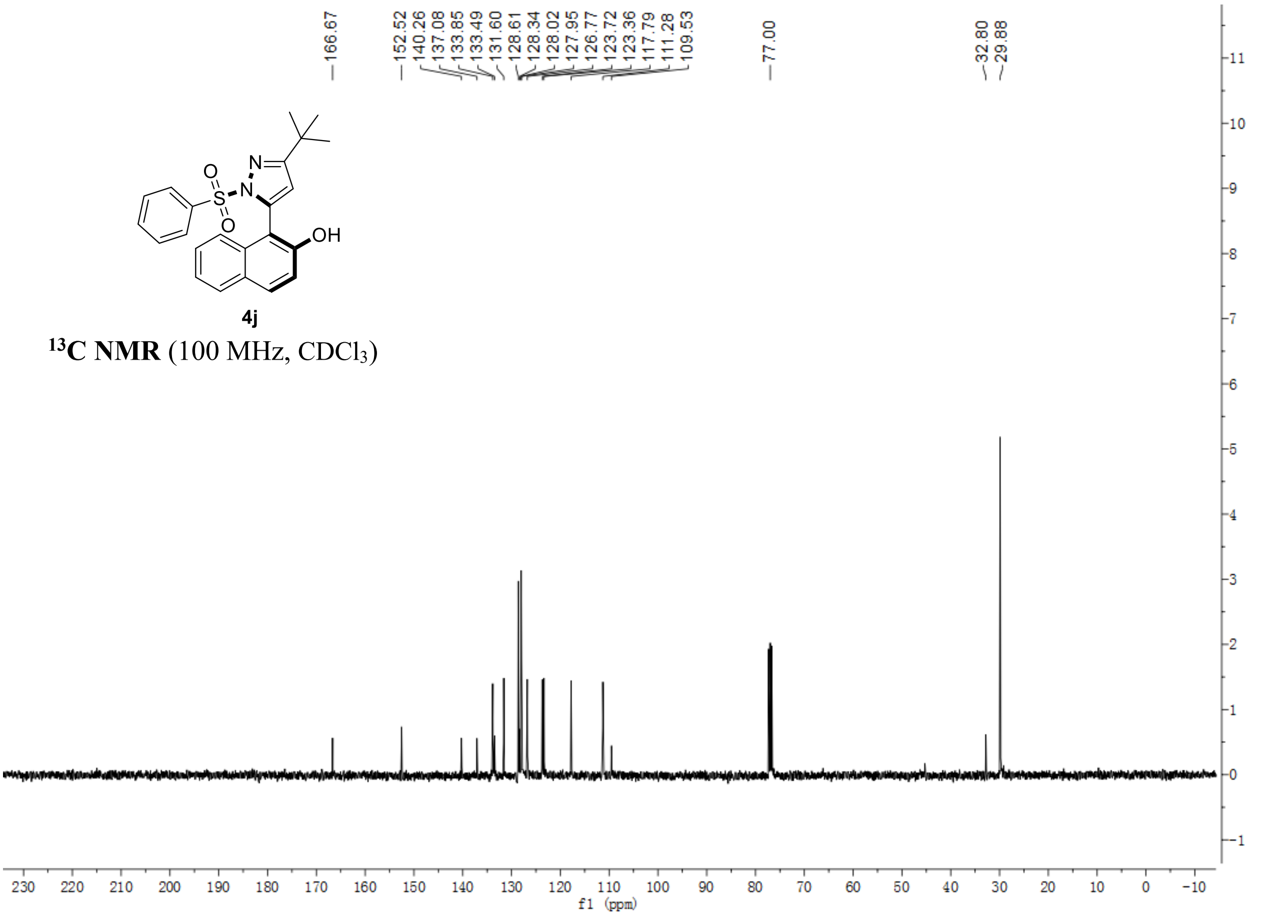

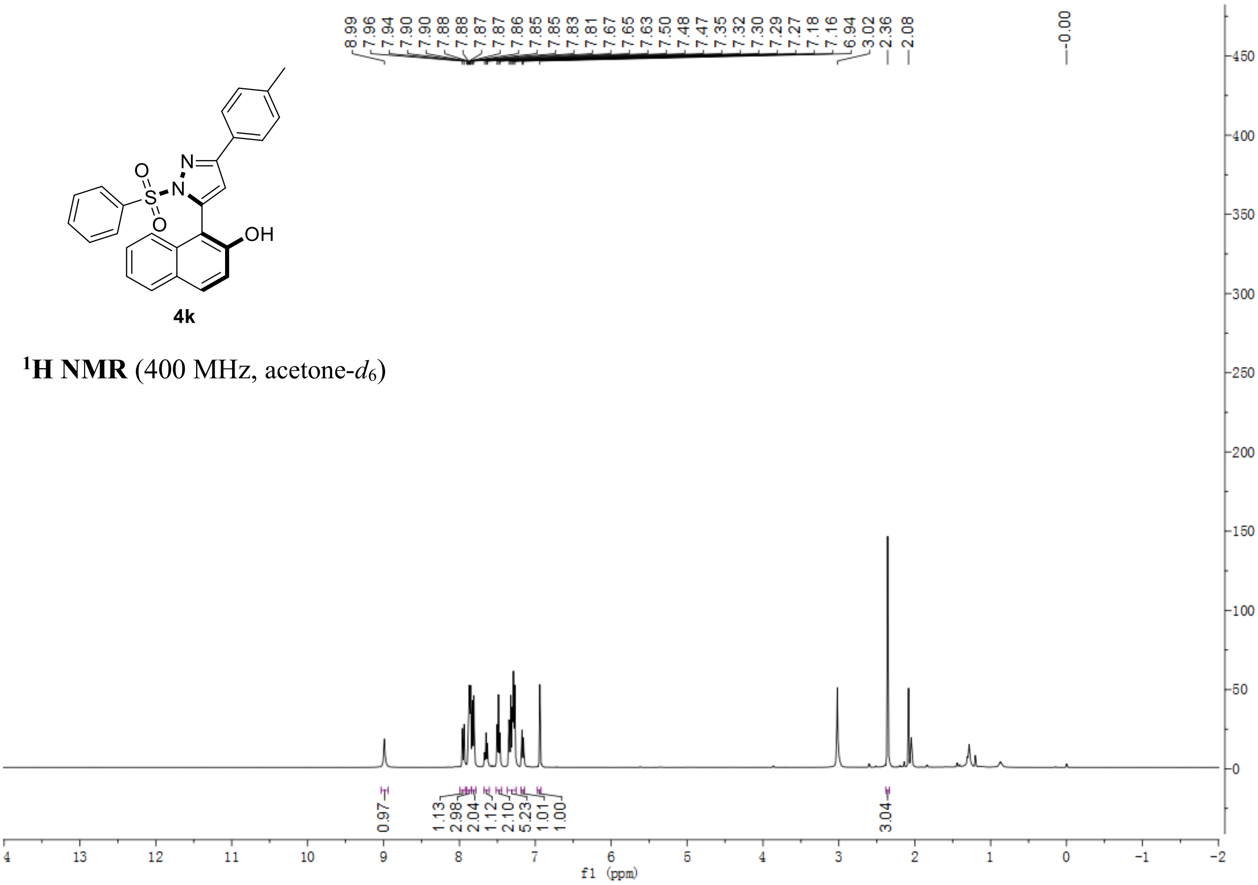

^{1}H NMR (400 MHz, acetone-d_6)

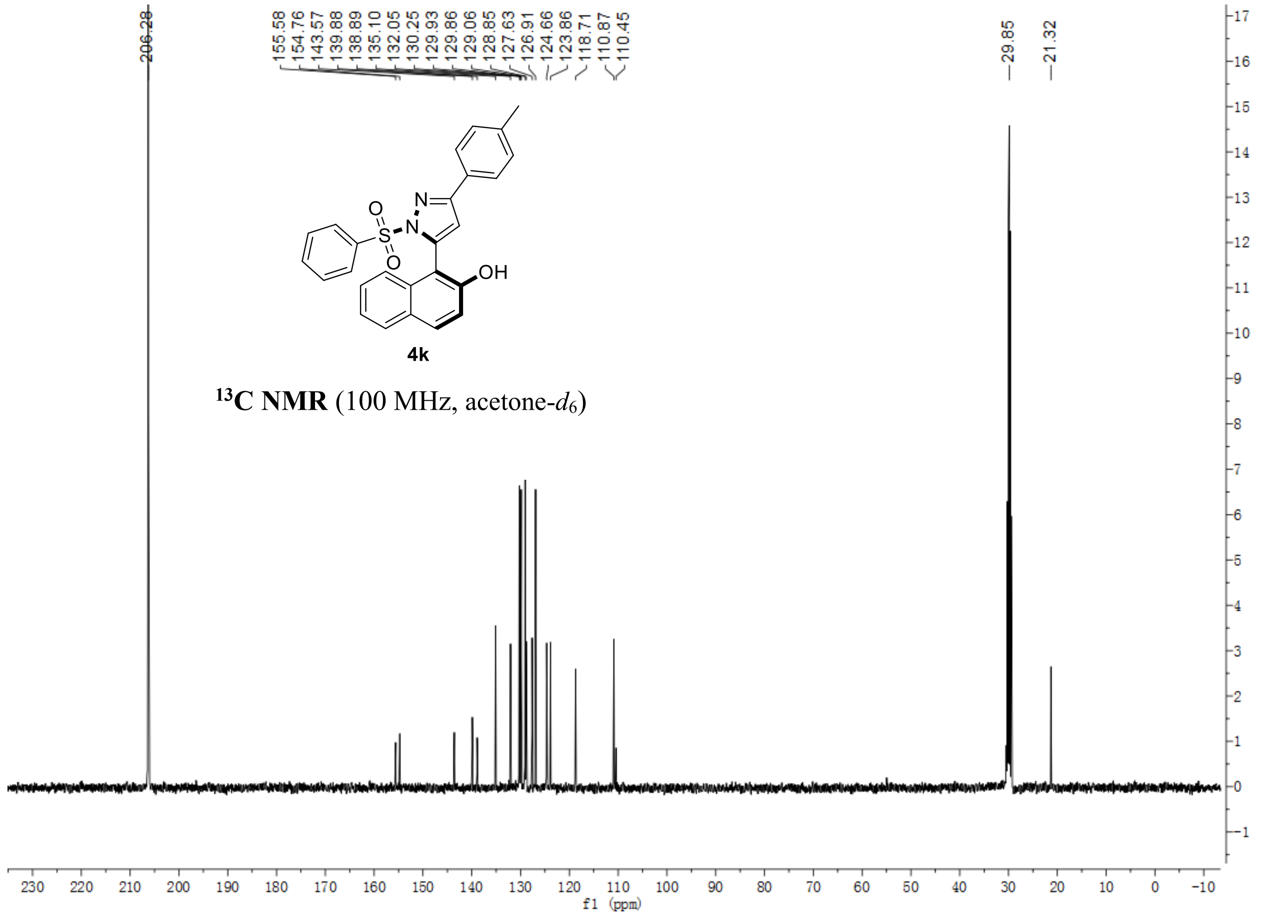

^{13}C NMR (100 MHz, acetone-d_6)

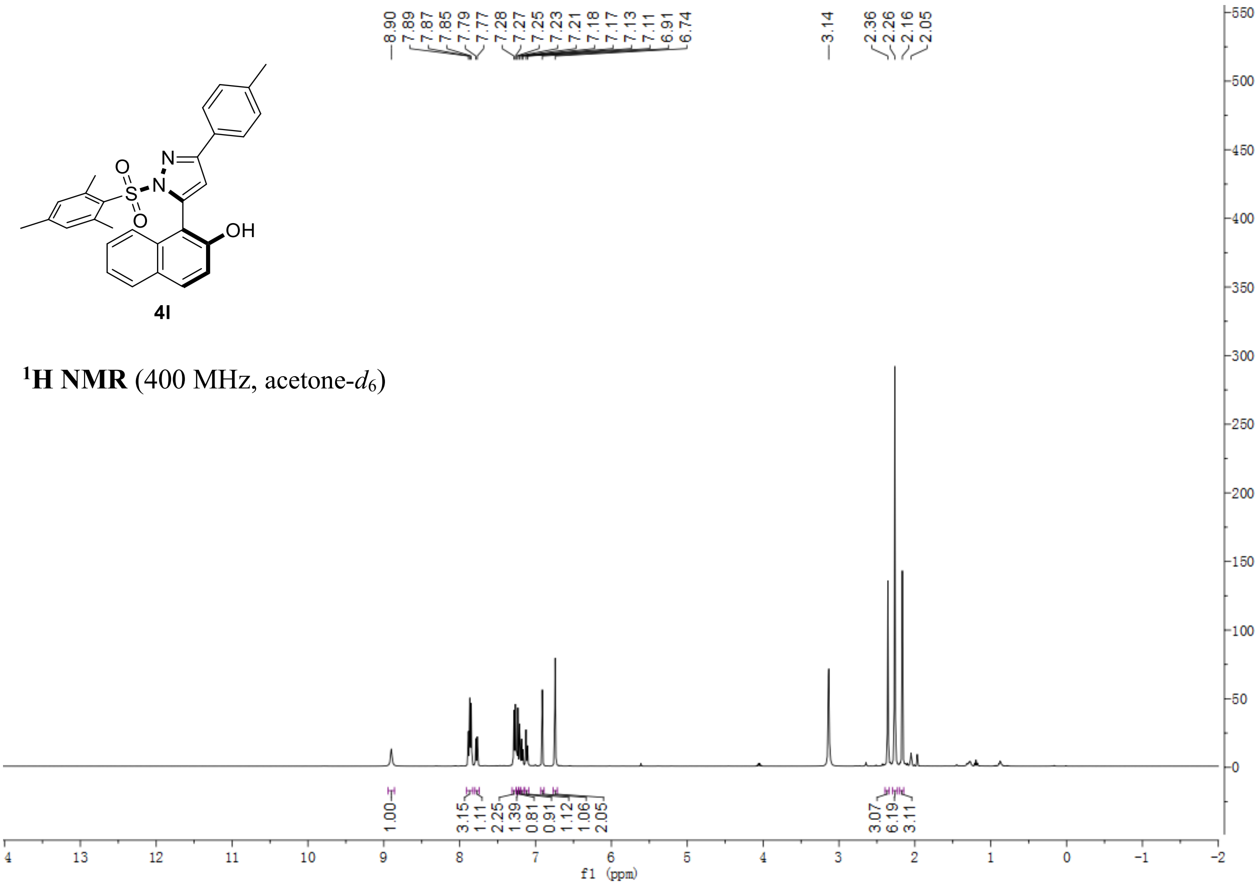

^{1}**H NMR** (400 MHz, acetone-d_6)

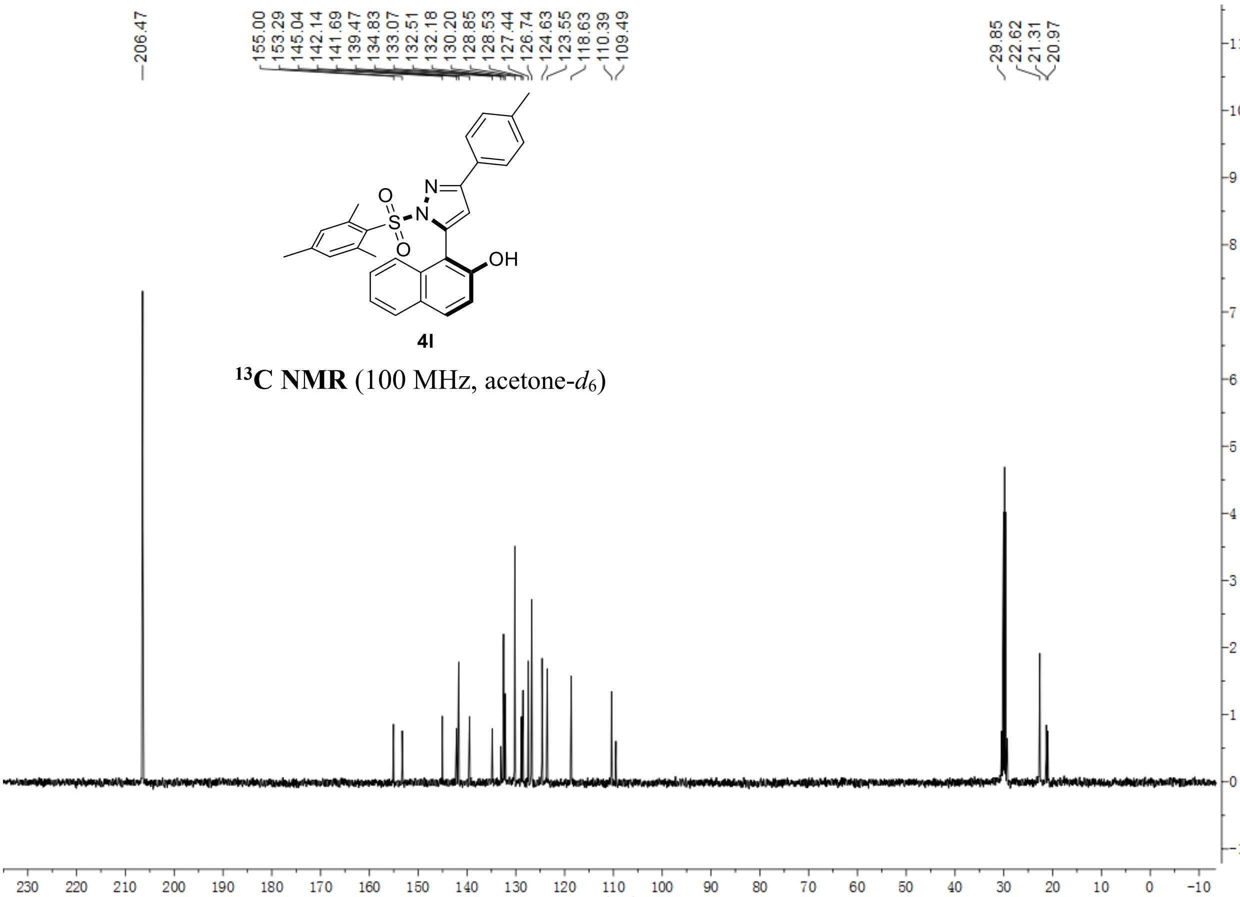

^{13}**C NMR** (100 MHz, acetone-d_6)

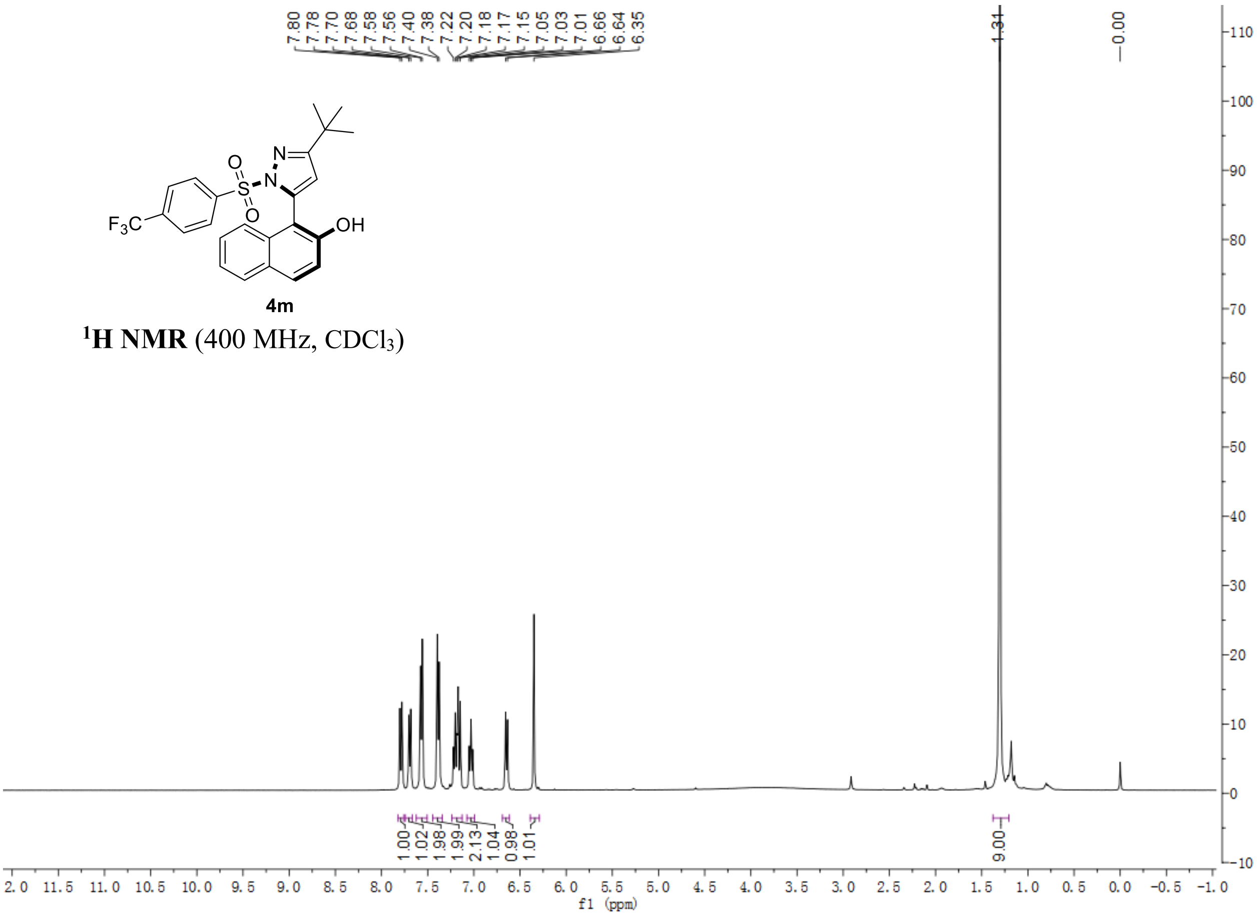
7.80
7.78
7.70
7.68
7.58
7.56
7.40
7.38
7.22
7.20
7.18
7.17
7.15
7.05
7.03
7.01
6.66
6.64
6.35
1.31
0.00
4m
^{1}H NMR (400 MHz, CDCl$_3$)
1.00
1.02
1.98
1.99
2.13
1.04
0.98
1.01
9.00
f1 (ppm)

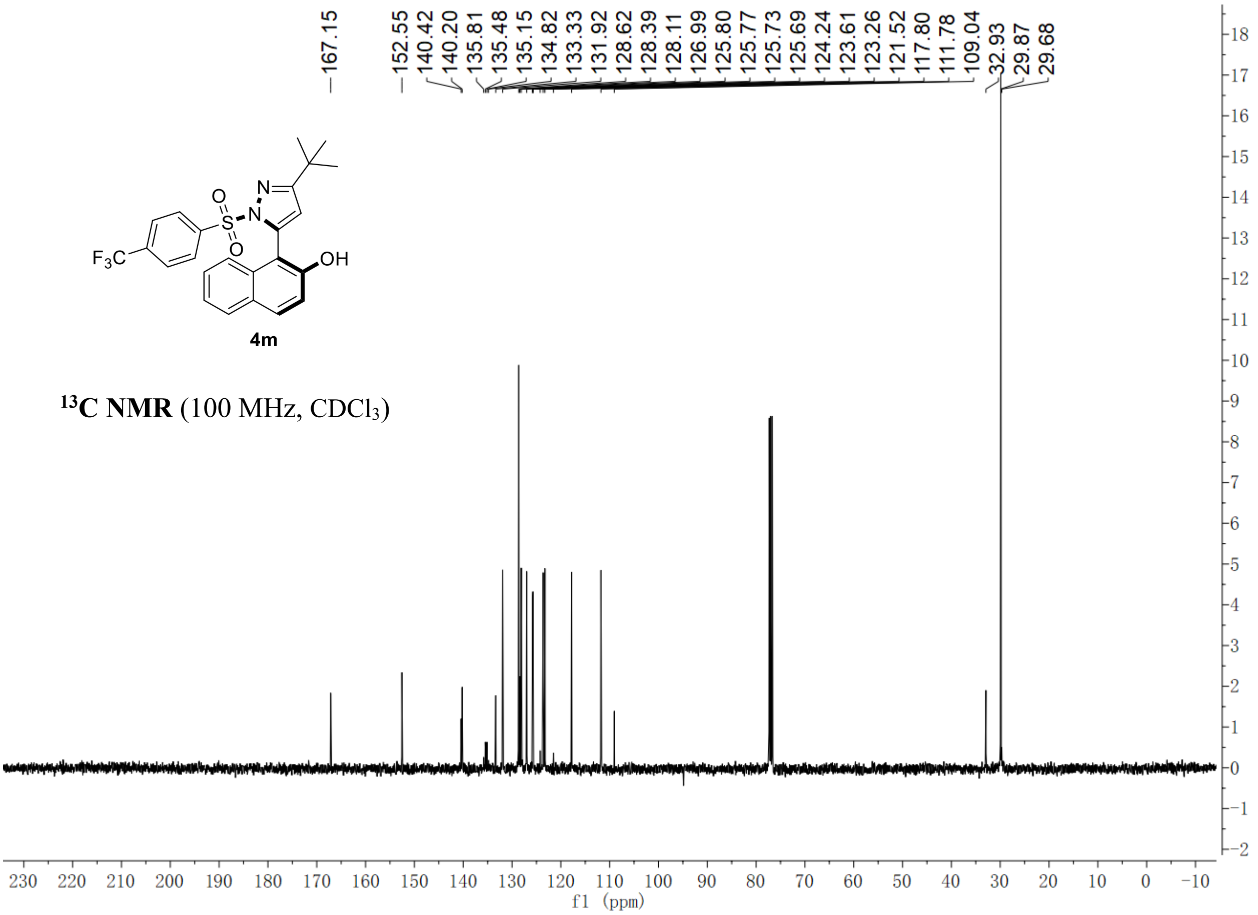
167.15
152.55
140.42
140.20
135.81
135.48
135.15
134.82
133.33
131.92
128.62
128.39
128.11
126.99
125.80
125.77
125.73
125.69
124.24
123.61
123.26
121.52
117.80
111.78
109.04
32.93
29.87
29.68
4m
^{13}C NMR (100 MHz, CDCl$_3$)
f1 (ppm)

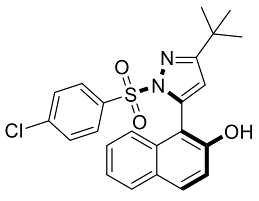

4n

^{1}H NMR (400 MHz, $CDCl_3$)

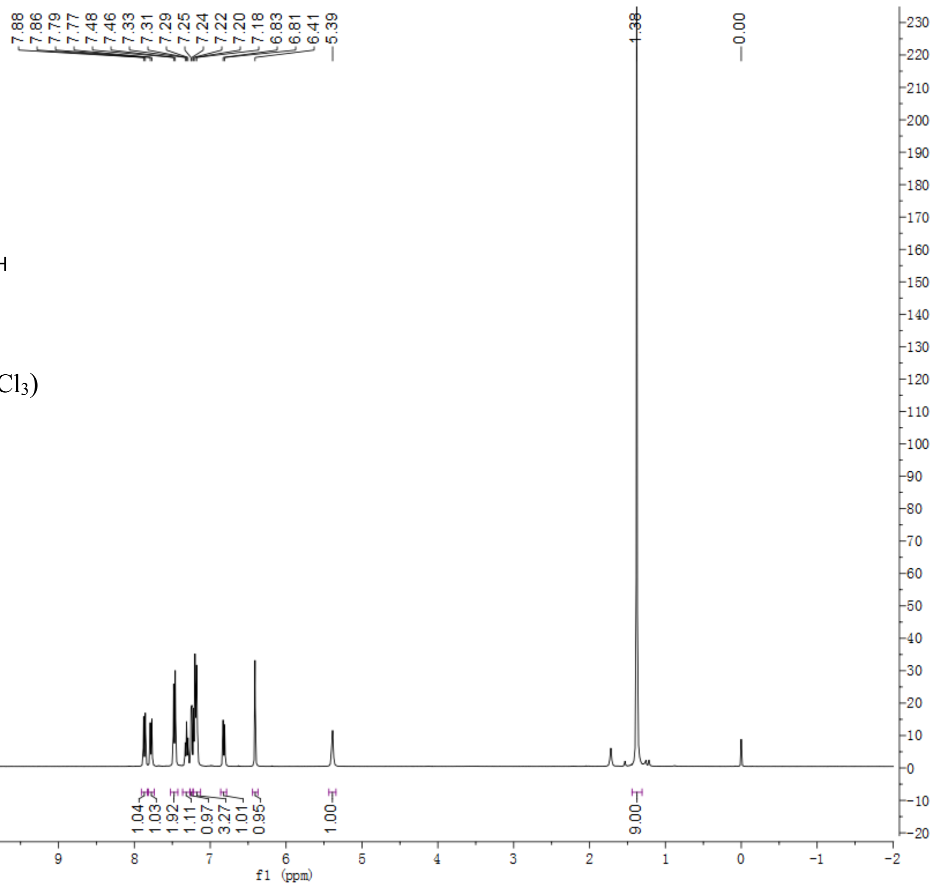

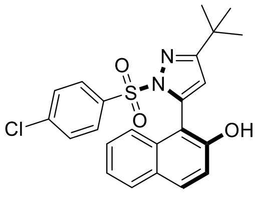

4n

^{13}C NMR (100 MHz, $CDCl_3$)

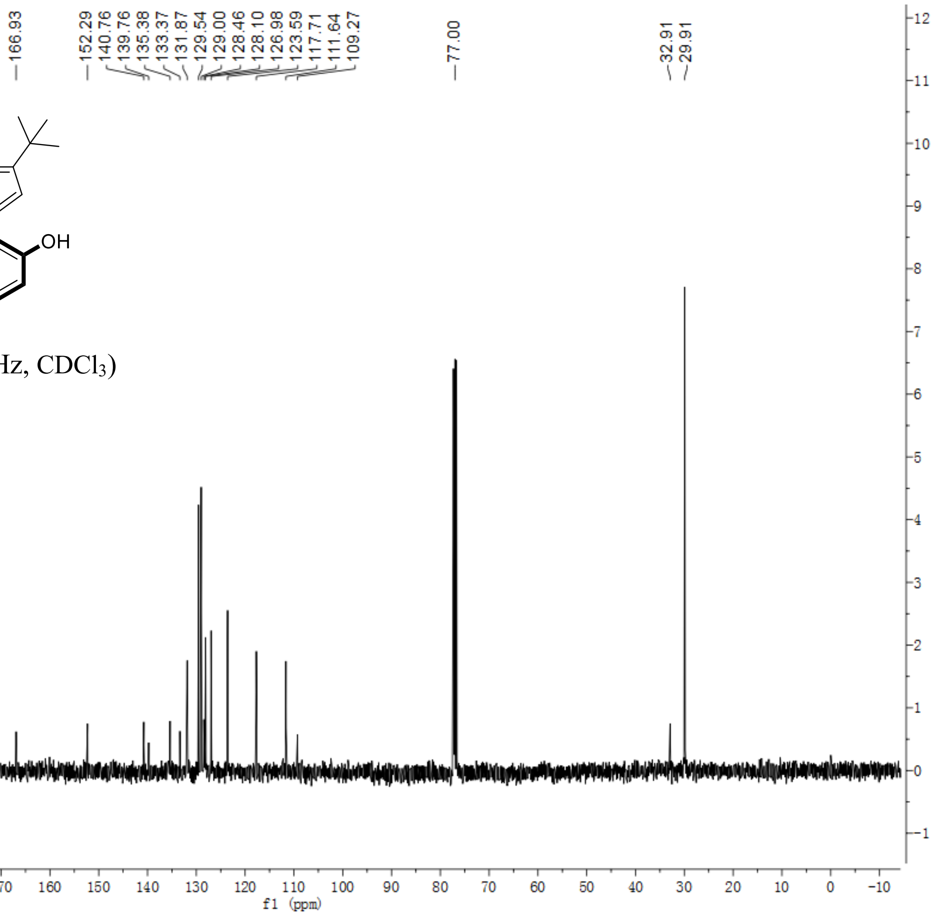

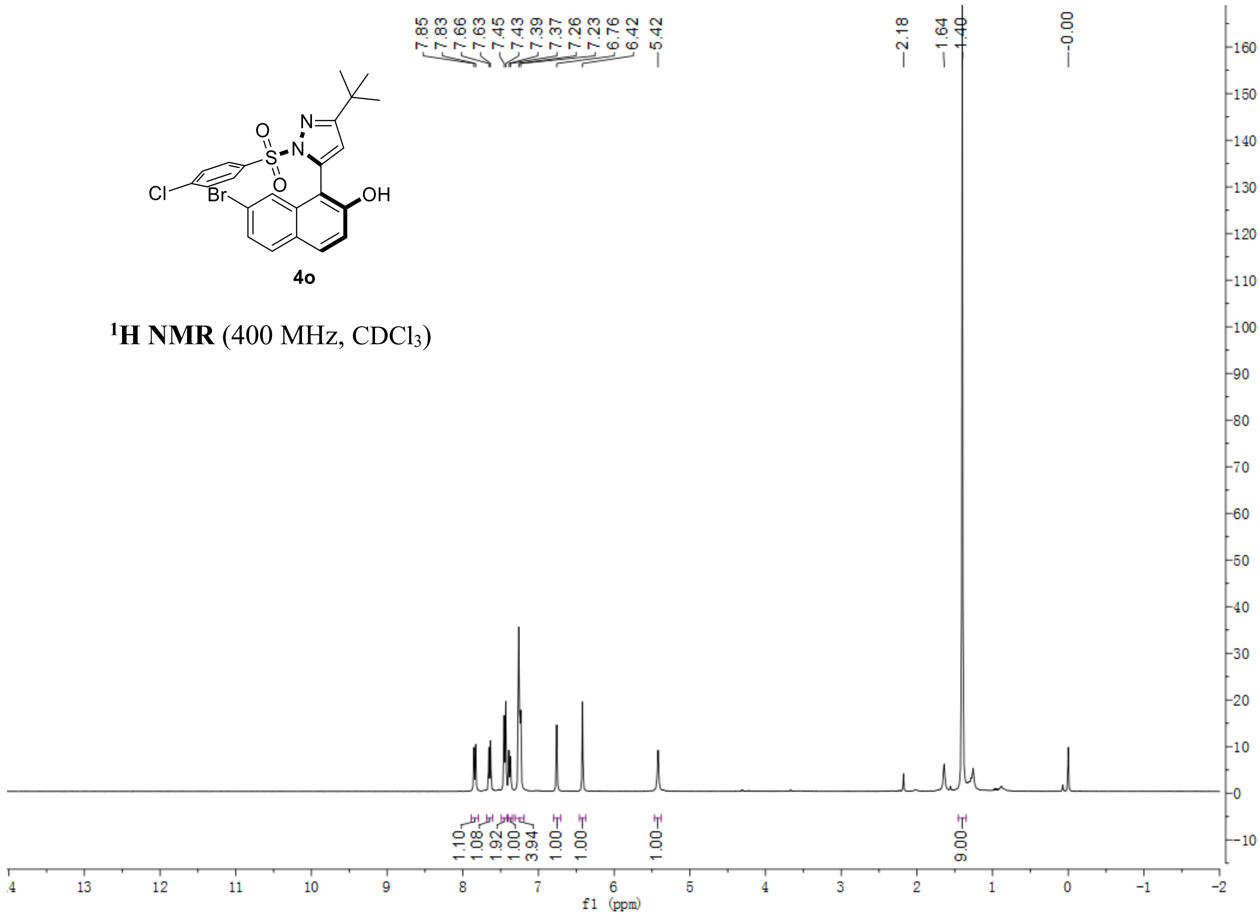
7.85
7.83
7.66
7.63
7.45
7.43
7.39
7.37
7.26
7.23
6.76
6.42
5.42
2.18
1.64
1.40
0.00
4o
^{1}H NMR (400 MHz, CDCl$_3$)
f1 (ppm)

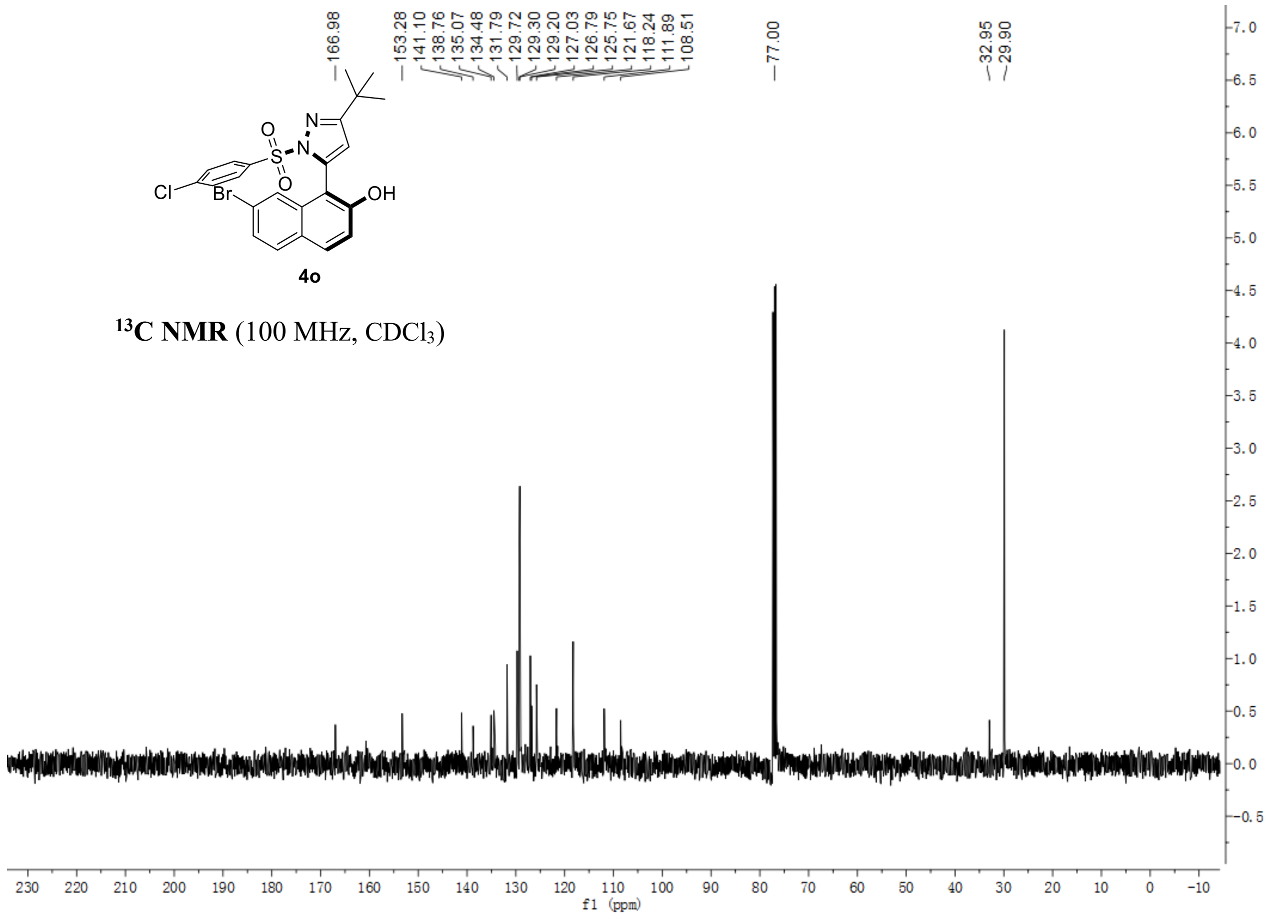
166.98
153.28
141.10
138.76
135.07
134.48
131.79
129.72
129.30
129.20
127.03
126.79
125.75
121.67
118.24
111.89
108.51
77.00
32.95
29.90
4o
^{13}C NMR (100 MHz, CDCl$_3$)
f1 (ppm)

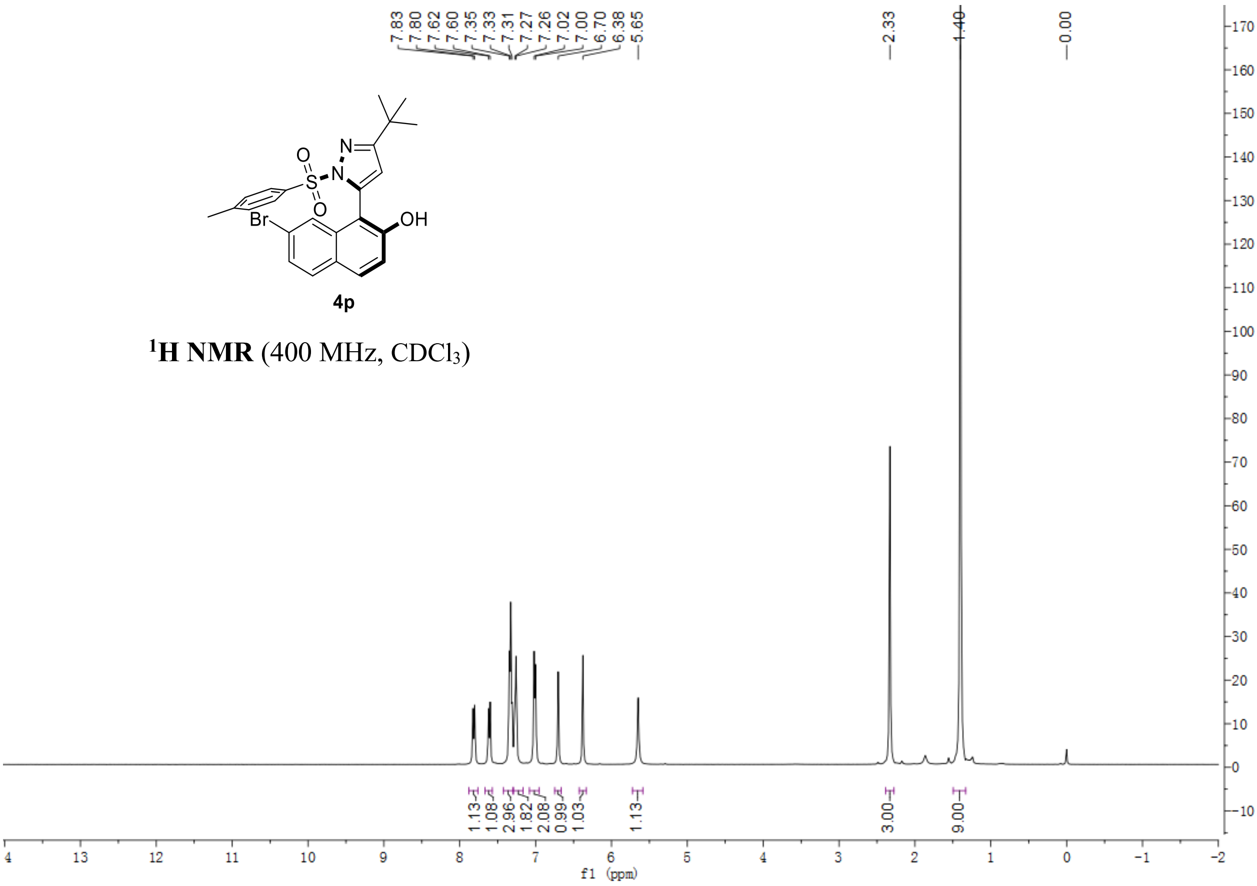

4p

^{1}H NMR (400 MHz, $CDCl_3$)

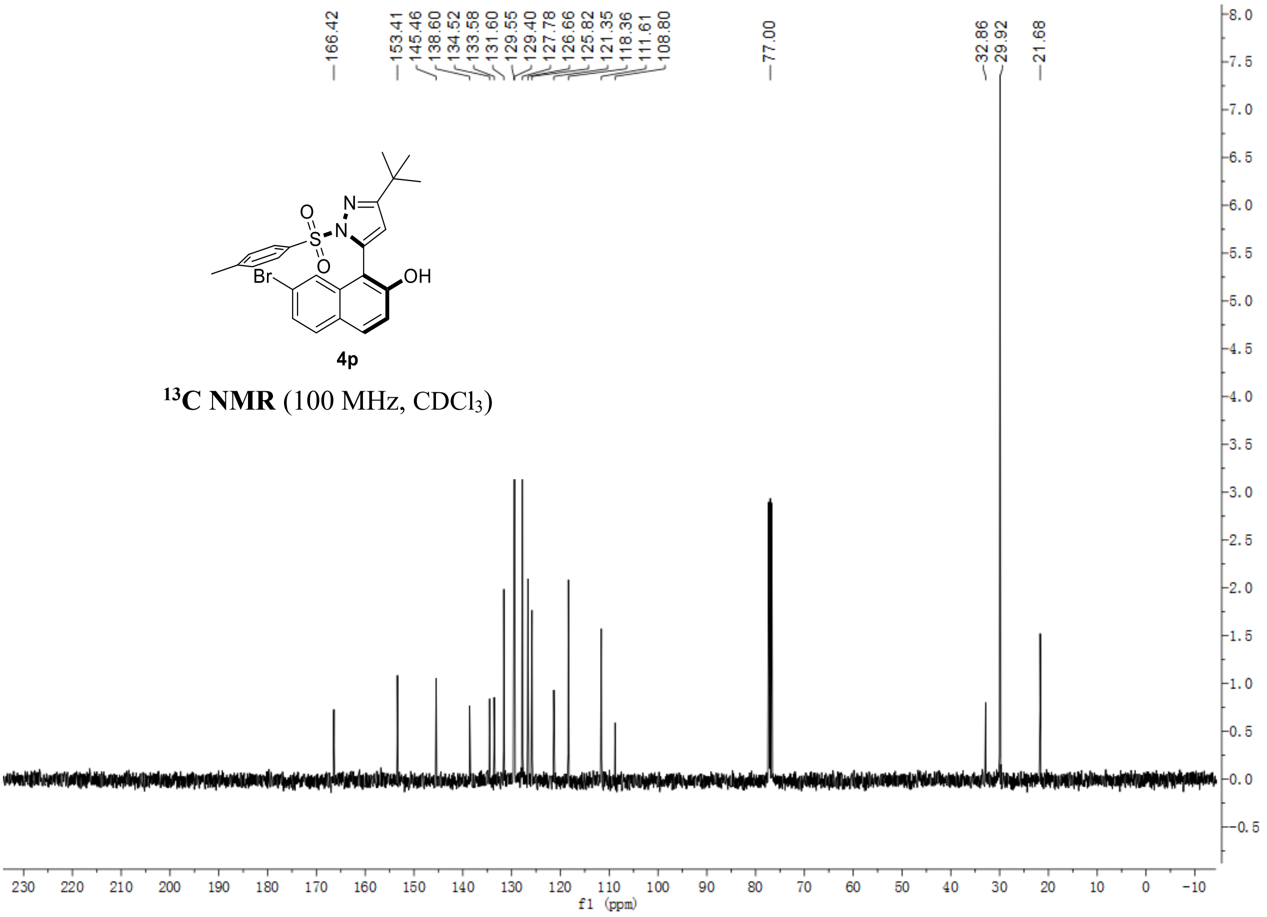

4p

^{13}C NMR (100 MHz, $CDCl_3$)

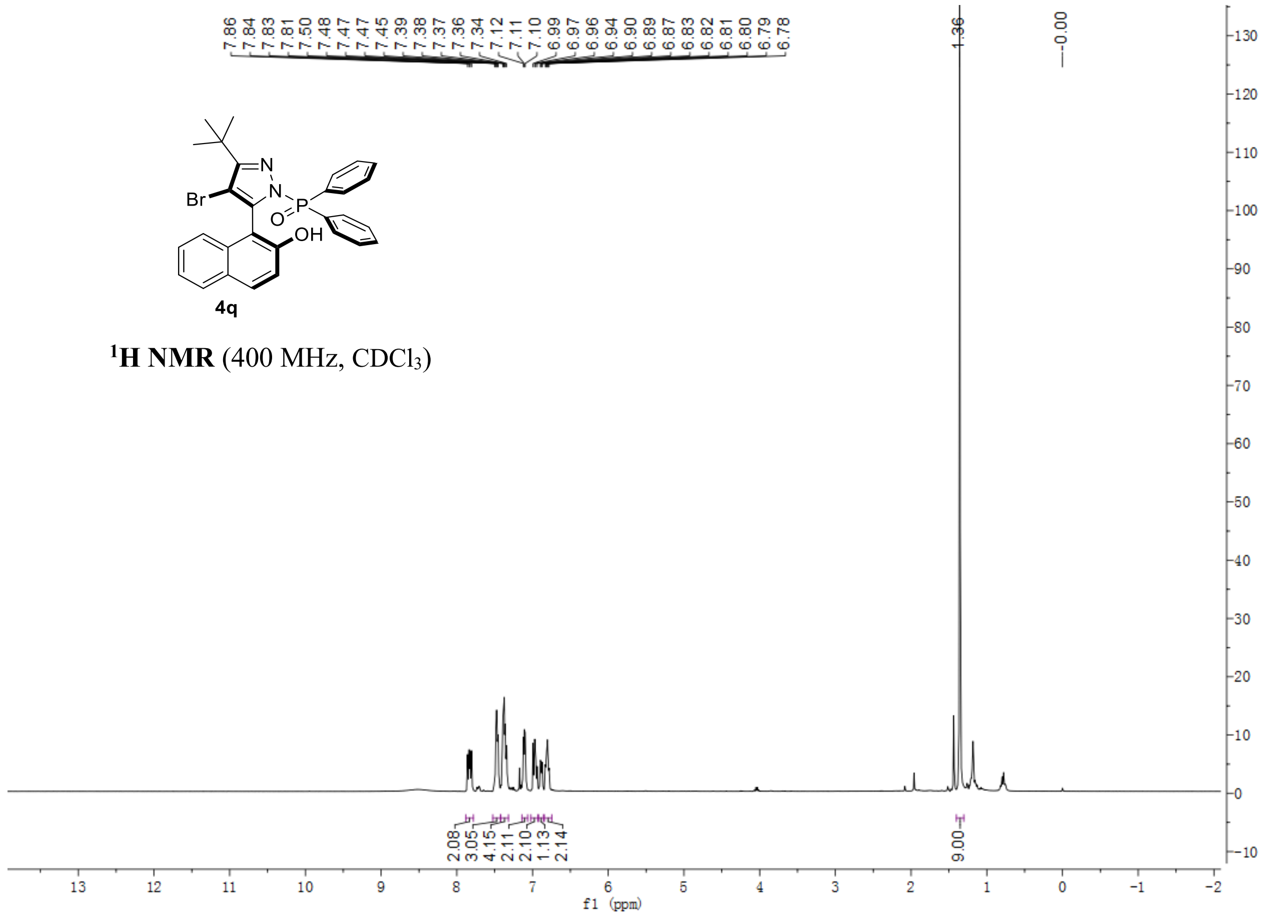
7.86 7.84 7.83 7.81 7.50 7.48 7.47 7.47 7.45 7.39 7.38 7.37 7.36 7.34 7.12 7.11 7.10 6.99 6.97 6.96 6.94 6.90 6.89 6.87 6.83 6.82 6.81 6.80 6.79 6.78
1.36
0.00
Br
N
N
O
P
OH
4q
¹H NMR (400 MHz, CDCl₃)
2.08 3.05 4.15 2.11 2.10 1.13 2.14
9.00
f1 (ppm)

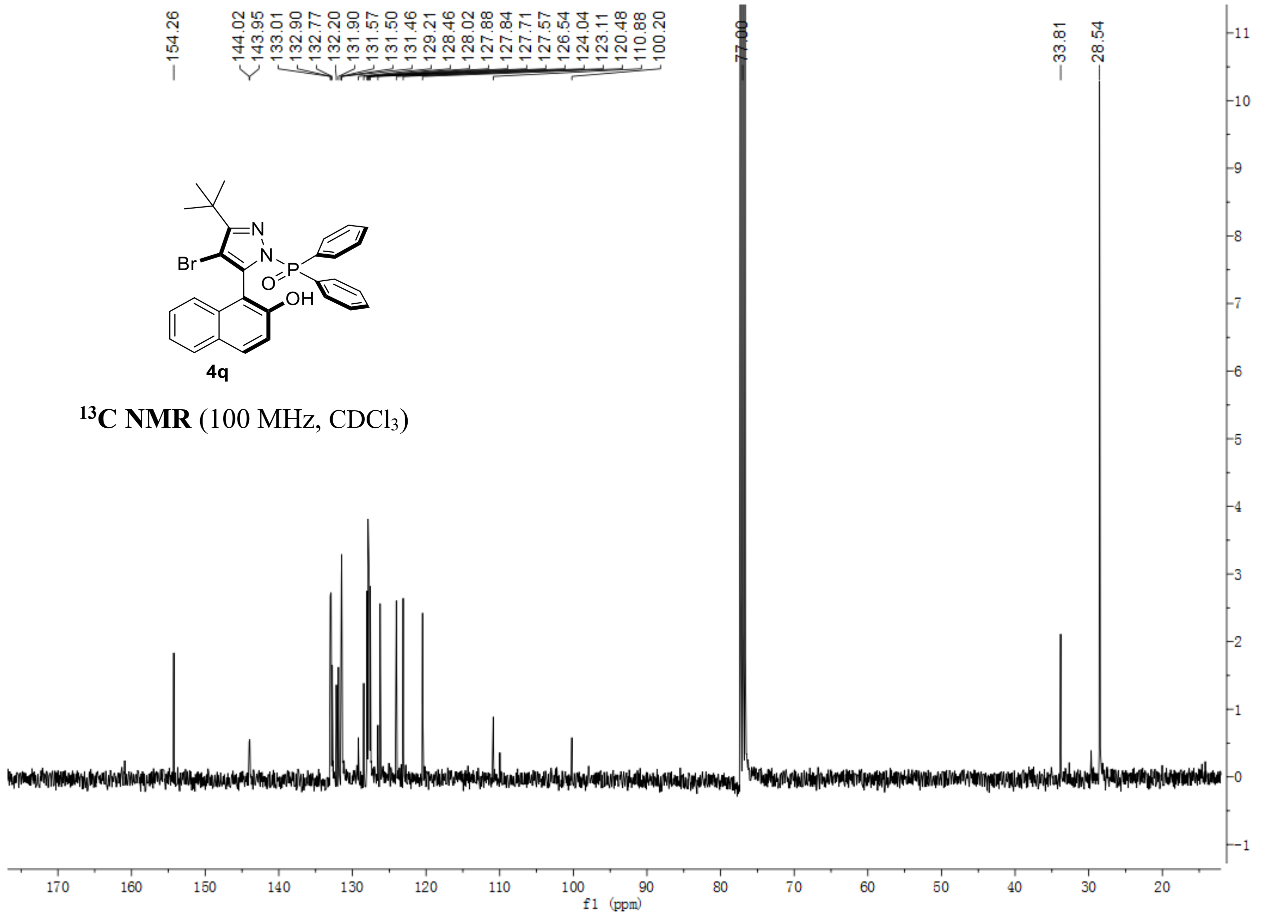
154.26
144.02 143.95 133.01 132.90 132.77 132.20 131.90 131.57 131.50 131.46 129.21 128.46 128.02 127.88 127.84 127.71 127.57 126.54 124.04 123.11 120.48 110.88 100.20
77.00
33.81 28.54
Br
N
N
O
P
OH
4q
¹³C NMR (100 MHz, CDCl₃)
f1 (ppm)

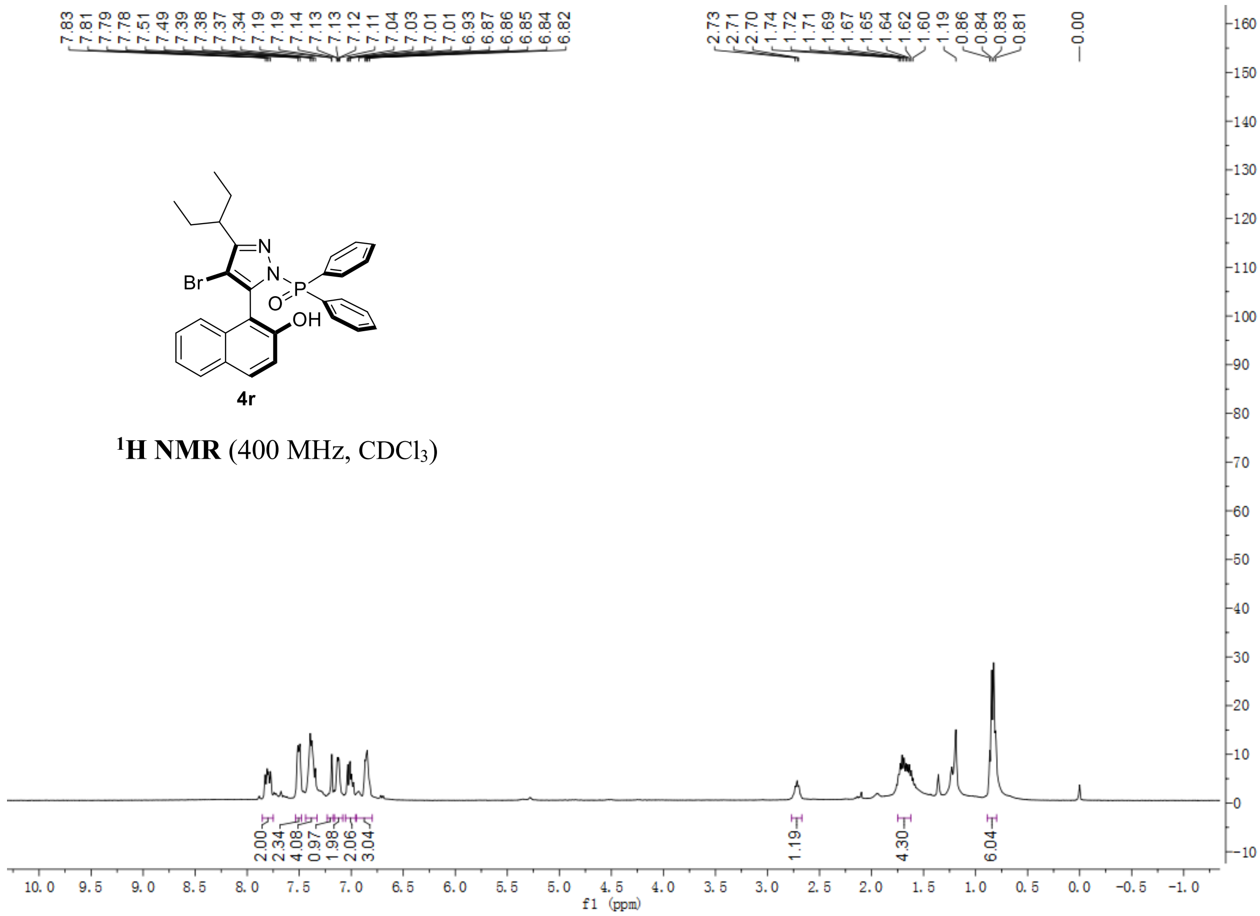

4r
^{1}H NMR (400 MHz, CDCl$_3$)

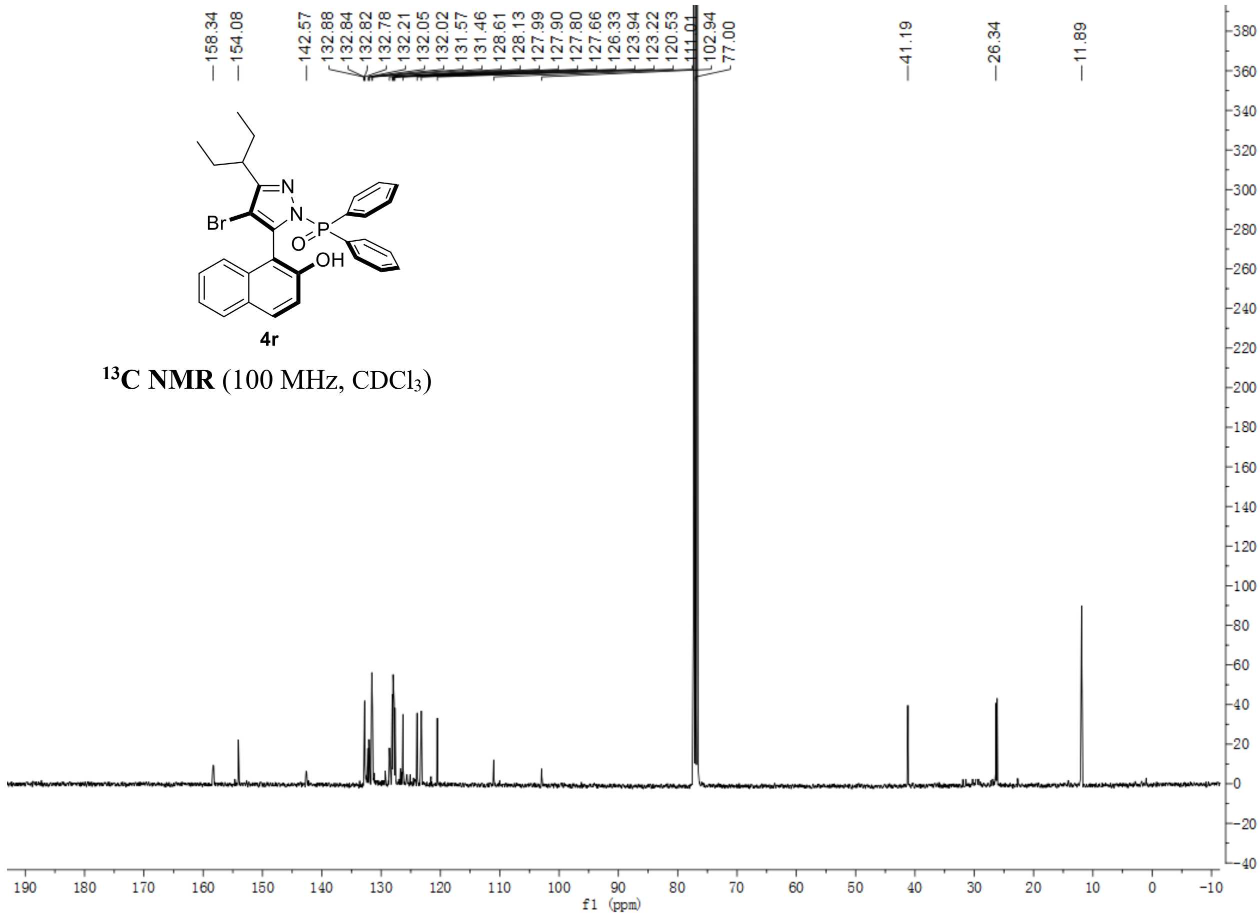

4r
^{13}C NMR (100 MHz, CDCl$_3$)

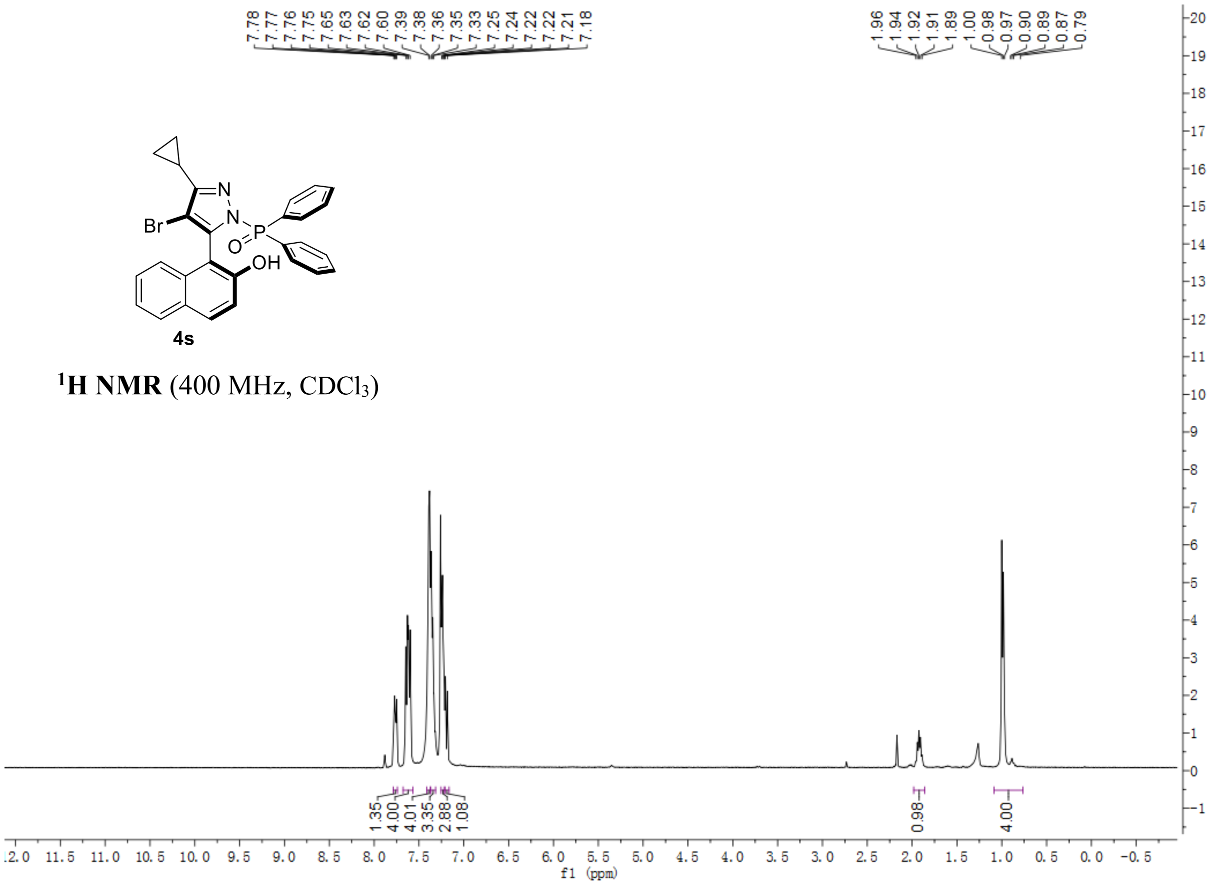
7.78 7.77 7.76 7.75 7.65 7.63 7.62 7.60 7.39 7.38 7.36 7.35 7.33 7.25 7.24 7.22 7.22 7.21 7.18
1.96 1.94 1.92 1.91 1.89 1.00 0.98 0.97 0.90 0.89 0.87 0.79
Br
N
N
P
O
OH
4s
^{1}H NMR (400 MHz, $CDCl_3$)
1.35 4.00 4.01 3.35 2.88 1.08
0.98
4.00
f1 (ppm)

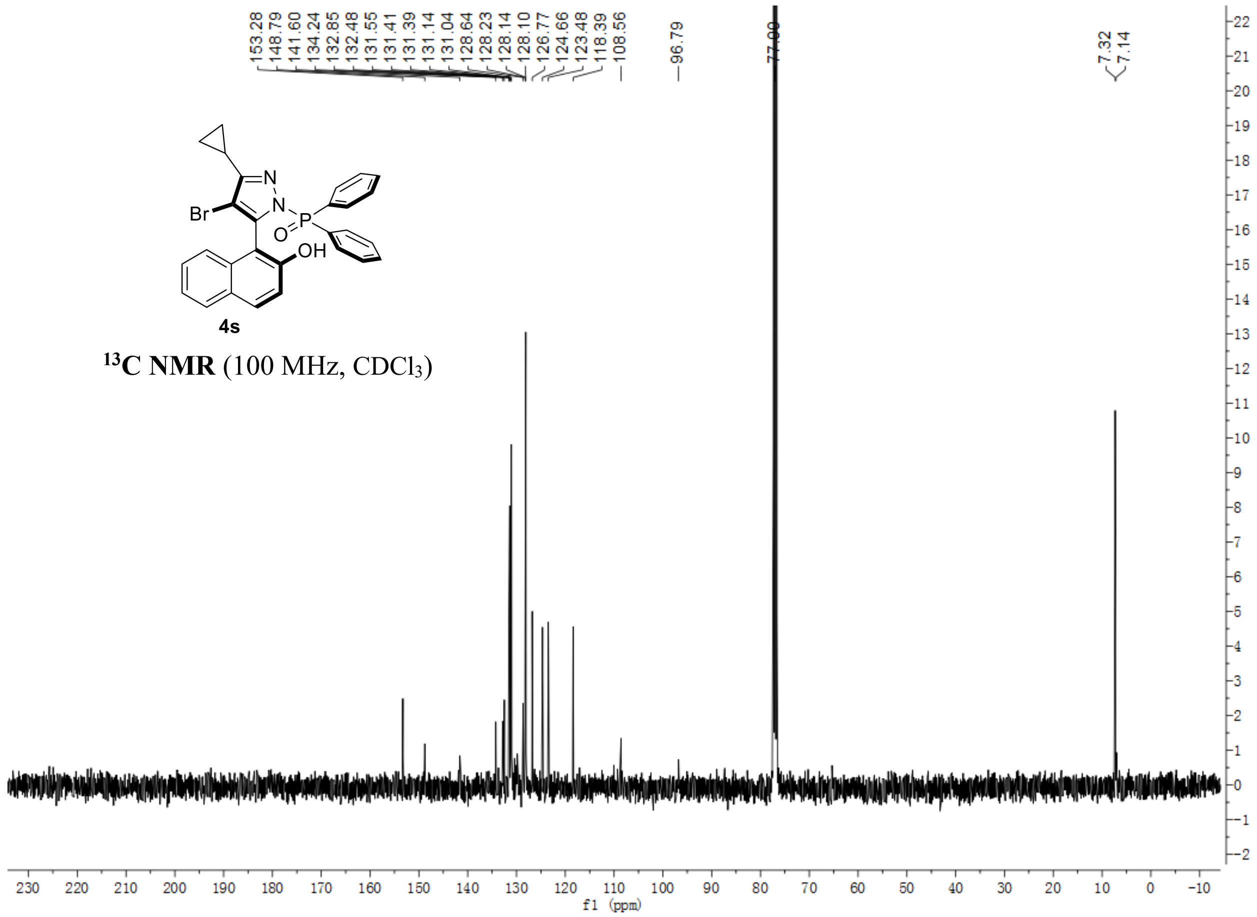
153.28 148.79 141.60 134.24 132.85 132.48 131.55 131.41 131.39 131.14 131.04 128.64 128.23 128.14 128.10 126.77 124.66 123.48 118.39 108.56
96.79
77.00
7.32 7.14
Br
N
N
P
O
OH
4s
^{13}C NMR (100 MHz, $CDCl_3$)
f1 (ppm)

4t

[1]**H NMR** (400 MHz, DMSO-d_6)

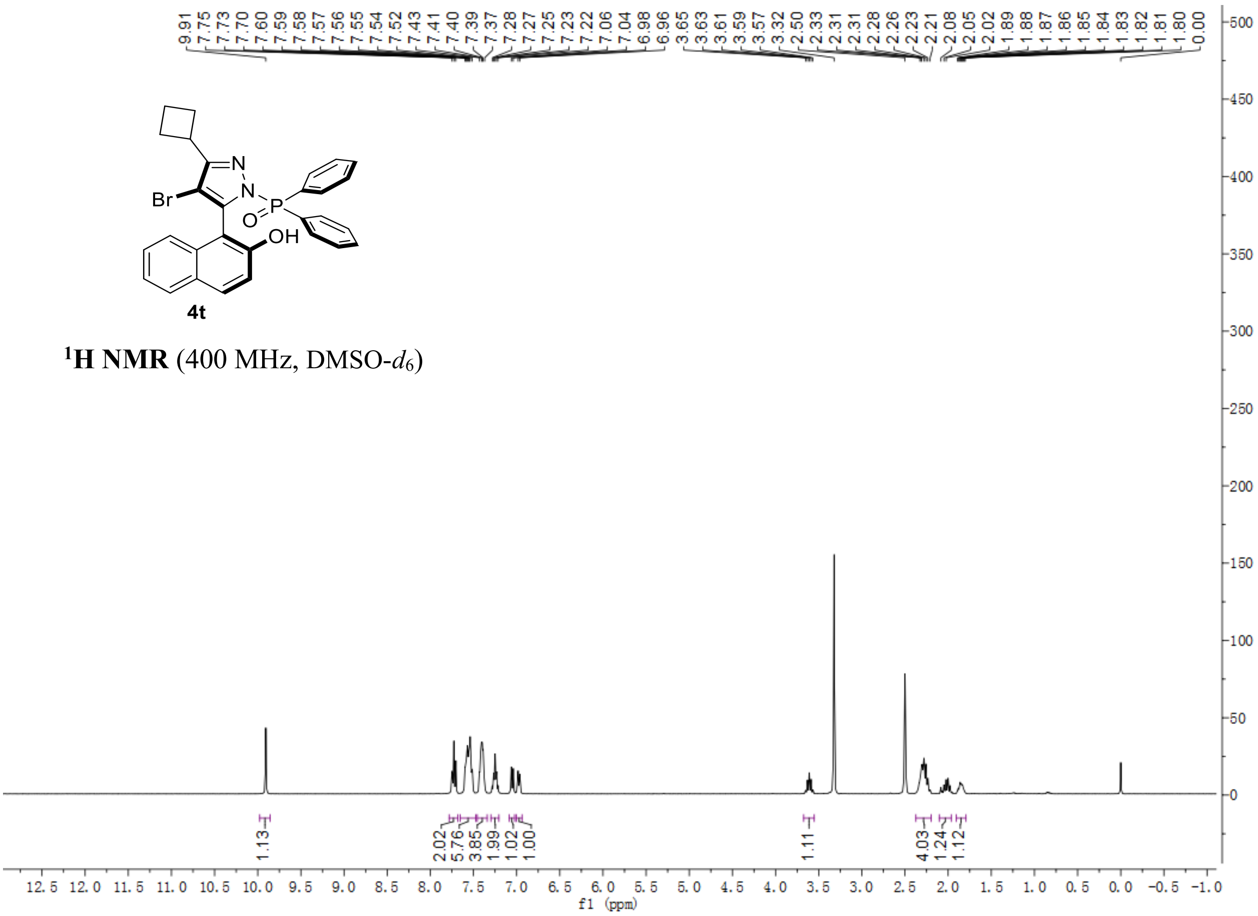

4t

[13]**C NMR** (100 MHz, DMSO-d_6)

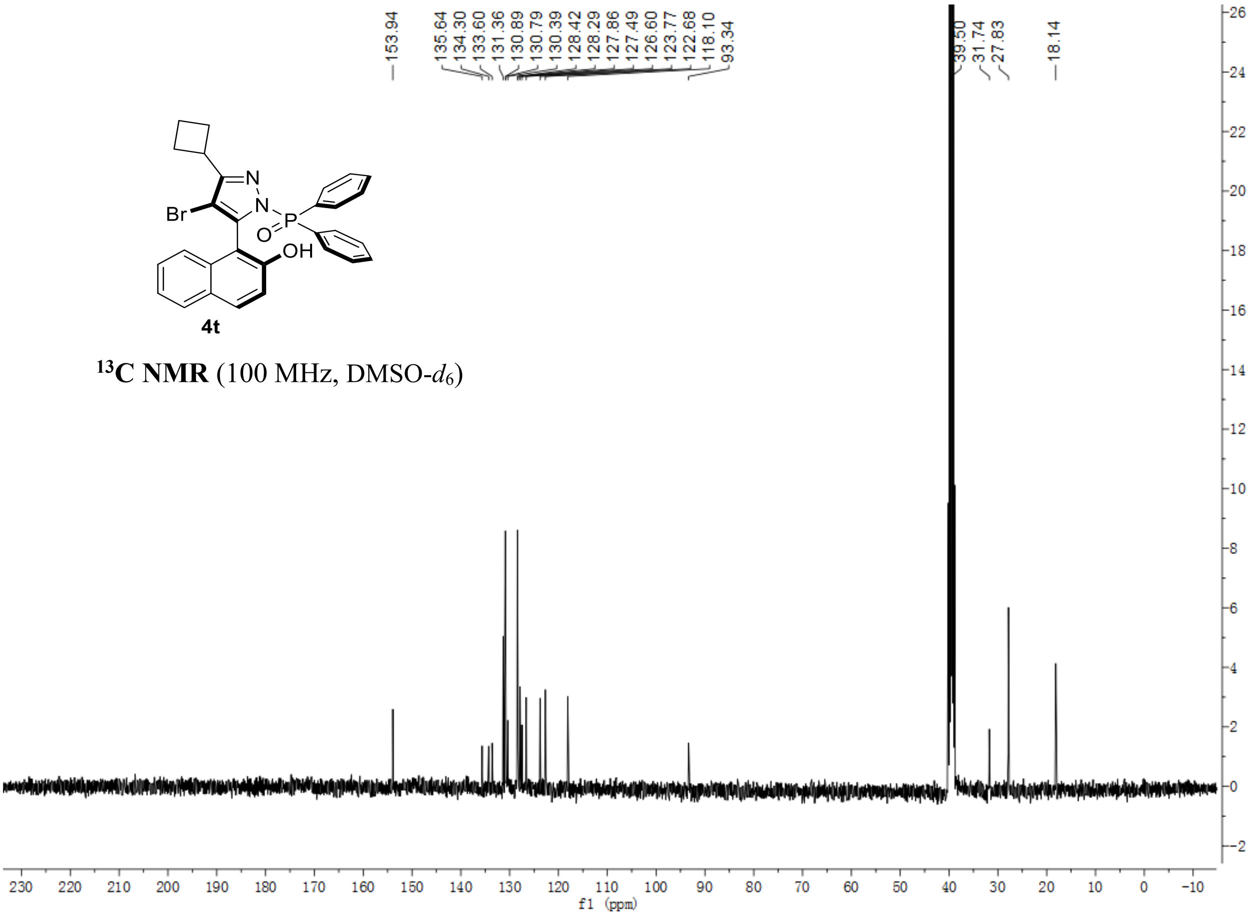

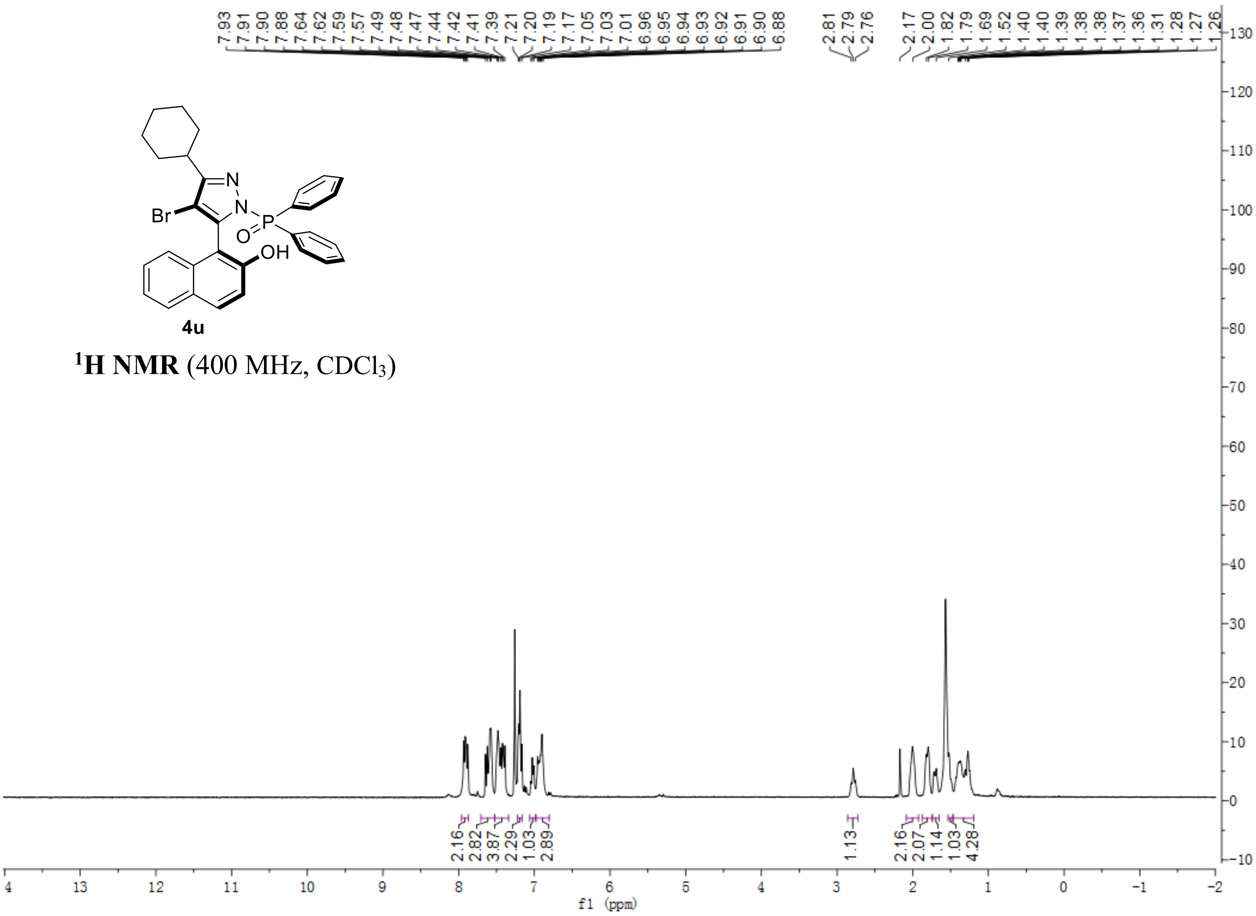
7.93
7.91
7.90
7.88
7.64
7.62
7.59
7.57
7.49
7.48
7.47
7.44
7.42
7.41
7.39
7.21
7.20
7.19
7.17
7.05
7.03
7.01
6.96
6.95
6.94
6.93
6.92
6.91
6.90
6.88
2.81
2.79
2.76
2.17
2.00
1.82
1.79
1.69
1.52
1.40
1.40
1.39
1.38
1.38
1.37
1.36
1.31
1.28
1.27
1.26
Br
N
N
P
O
OH
4u
¹H NMR (400 MHz, CDCl₃)
2.16
2.82
3.87
2.29
1.03
2.89
1.13
2.16
2.07
1.14
1.03
4.28
f1 (ppm)

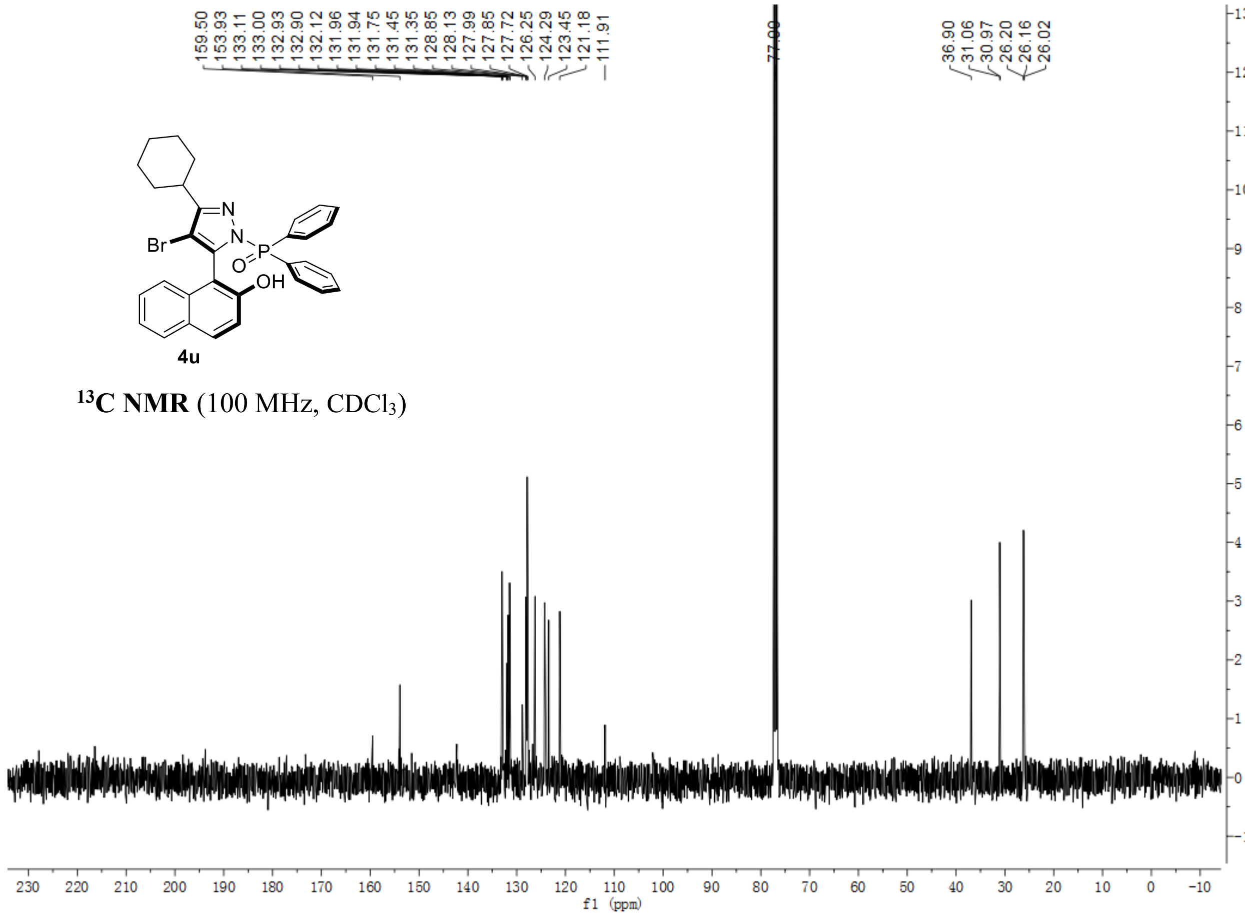
159.50
153.93
133.11
133.00
132.93
132.90
132.12
131.96
131.94
131.75
131.45
131.35
128.85
128.13
127.99
127.85
127.72
126.25
124.29
123.45
121.18
111.91
77.00
36.90
31.06
30.97
26.20
26.16
26.02
Br
N
N
P
O
OH
4u
¹³C NMR (100 MHz, CDCl₃)
f1 (ppm)

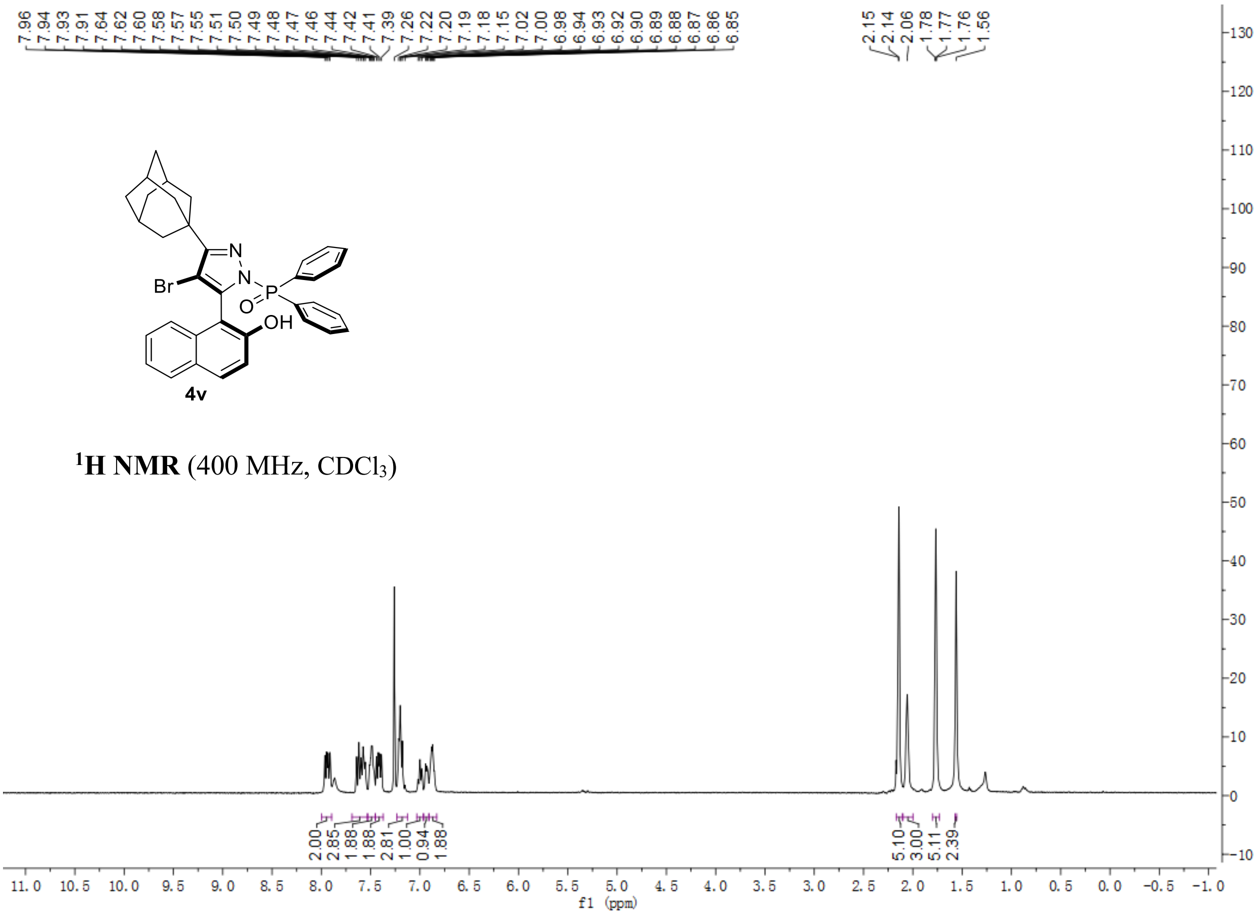
7.96 7.94 7.93 7.91 7.64 7.62 7.60 7.58 7.57 7.55 7.51 7.50 7.49 7.48 7.47 7.46 7.44 7.42 7.41 7.39 7.26 7.22 7.20 7.19 7.18 7.15 7.02 7.00 6.98 6.94 6.93 6.92 6.90 6.89 6.88 6.87 6.86 6.85
2.15 2.14 2.06 1.78 1.77 1.76 1.56
Br
OH
4v
^{1}H NMR (400 MHz, $CDCl_3$)
2.00 2.85 1.88 1.88 2.81 1.00 0.94 1.88
5.10 3.00 5.11 2.39
f1 (ppm)

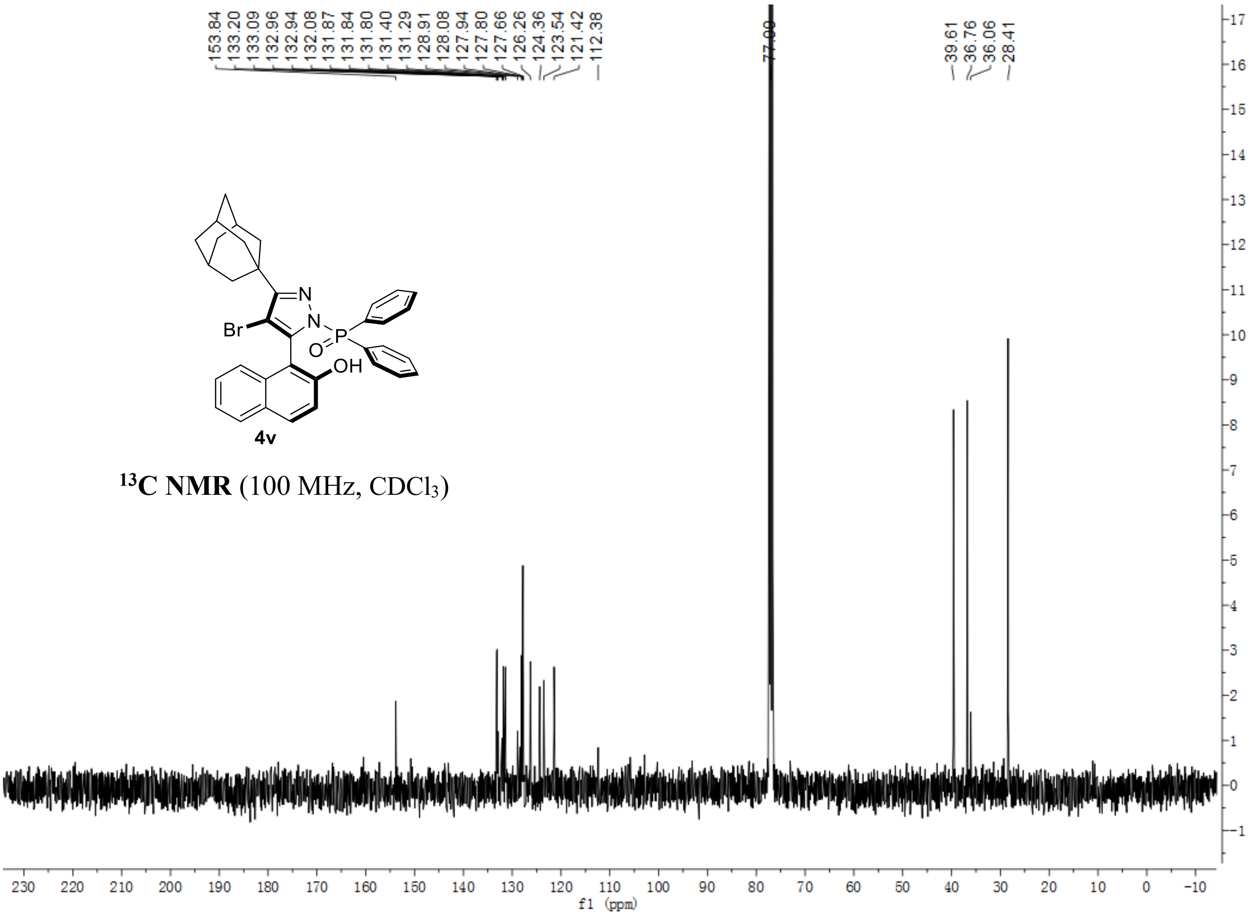
153.84 133.20 133.09 132.96 132.94 132.08 131.87 131.84 131.80 131.40 131.29 128.91 128.08 127.94 127.80 127.66 126.26 124.36 123.54 121.42 112.38
77.00
39.61 36.76 36.06 28.41
Br
OH
4v
^{13}C NMR (100 MHz, $CDCl_3$)
f1 (ppm)

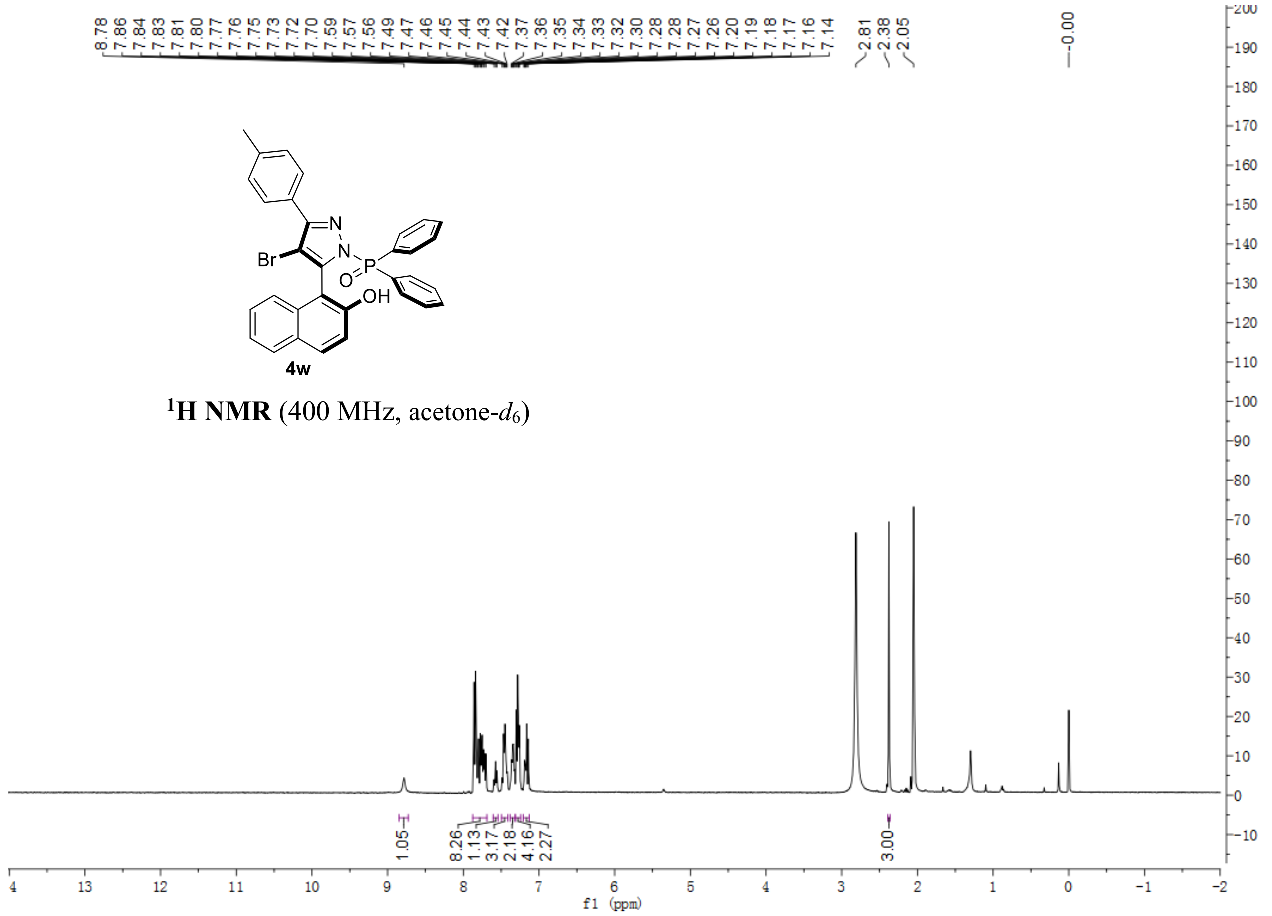

^{1}H NMR (400 MHz, acetone-d_6)

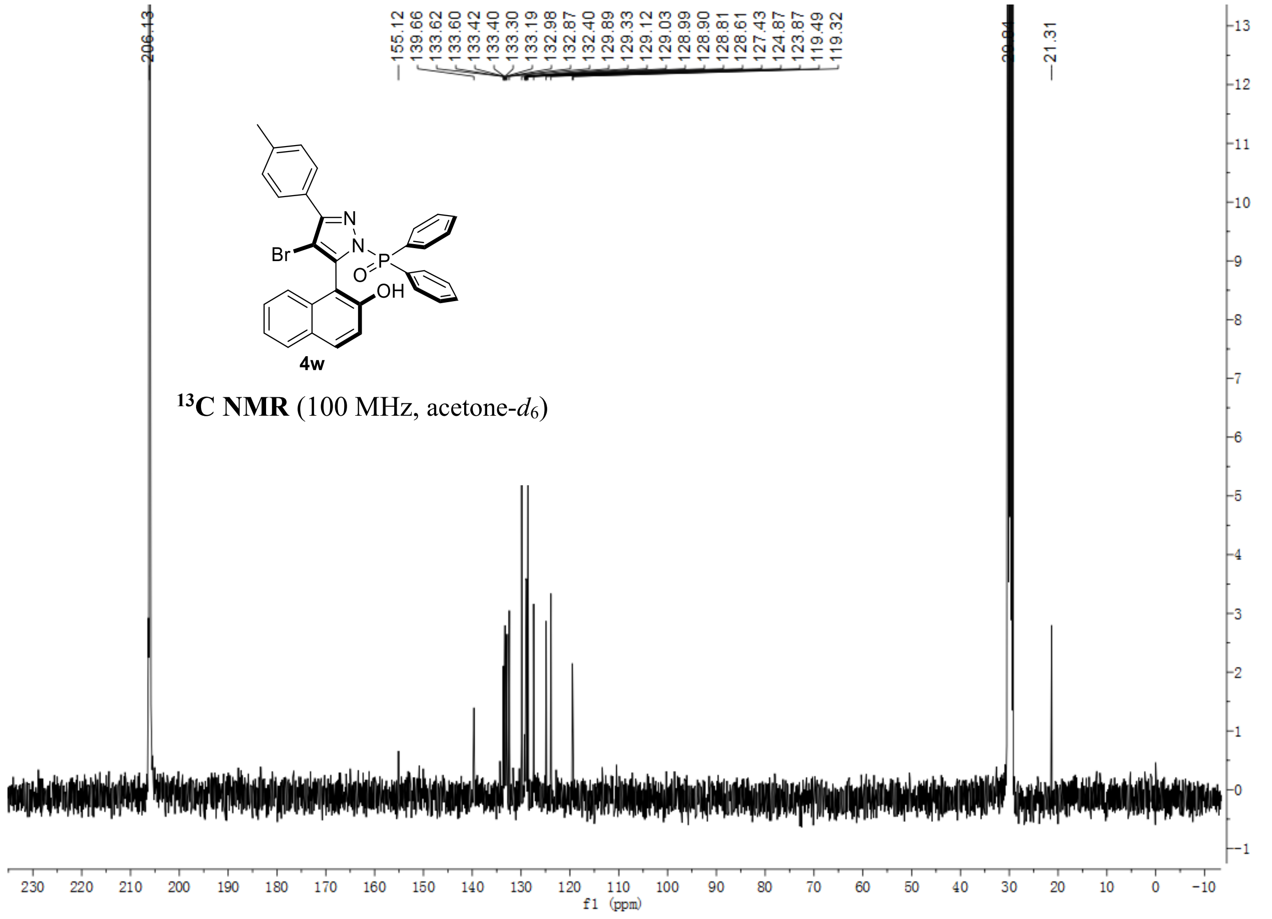

^{13}C NMR (100 MHz, acetone-d_6)

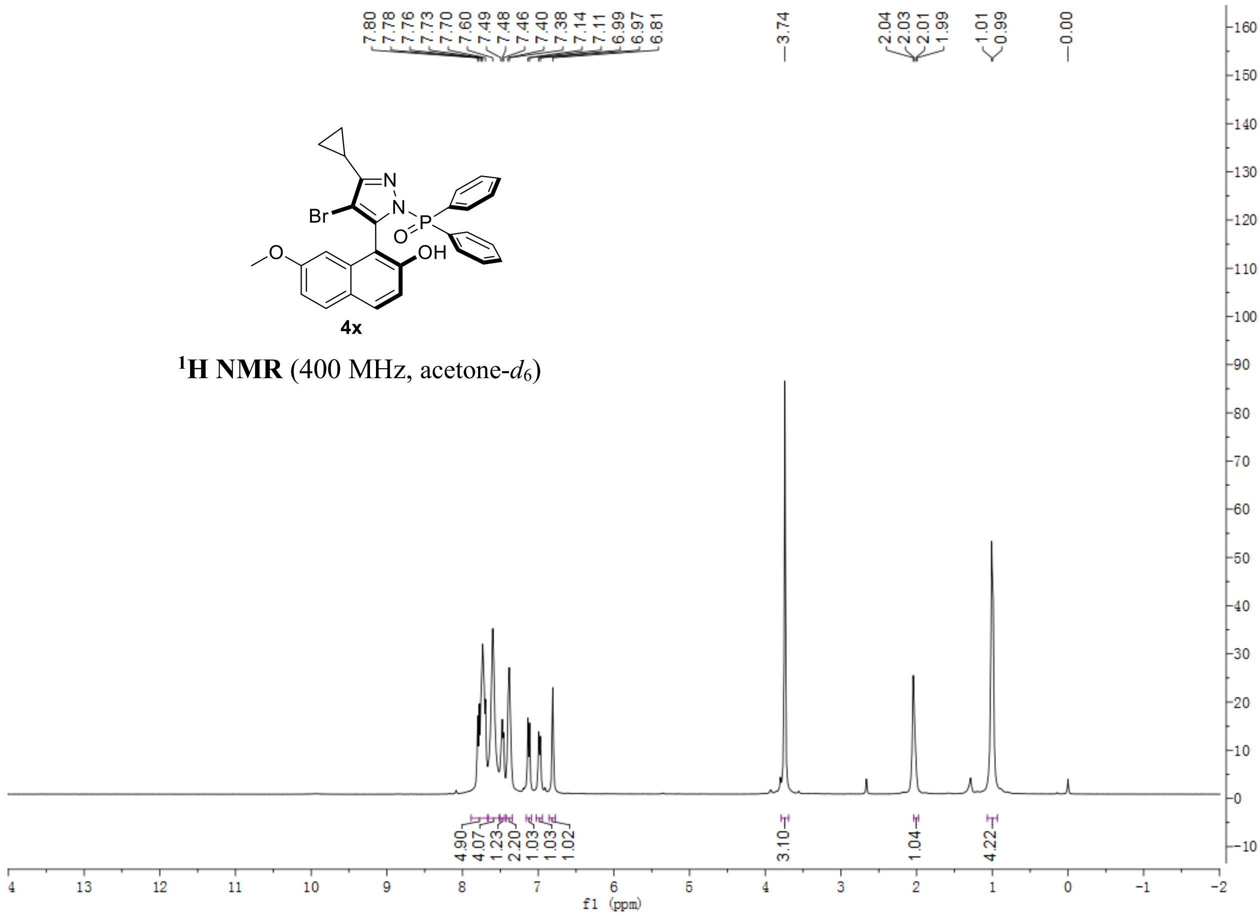
7.80 7.78 7.76 7.73 7.70 7.60 7.49 7.48 7.46 7.40 7.38 7.14 7.11 6.99 6.97 6.81
3.74
2.04 2.03 2.01 1.99
1.01 0.99
0.00
Br
N
N
P
O
OH
4x
1H NMR (400 MHz, acetone-*d*6)
4.90 4.07 1.23 2.20 1.03 1.03 1.03 1.02
3.10
1.04
4.22
f1 (ppm)

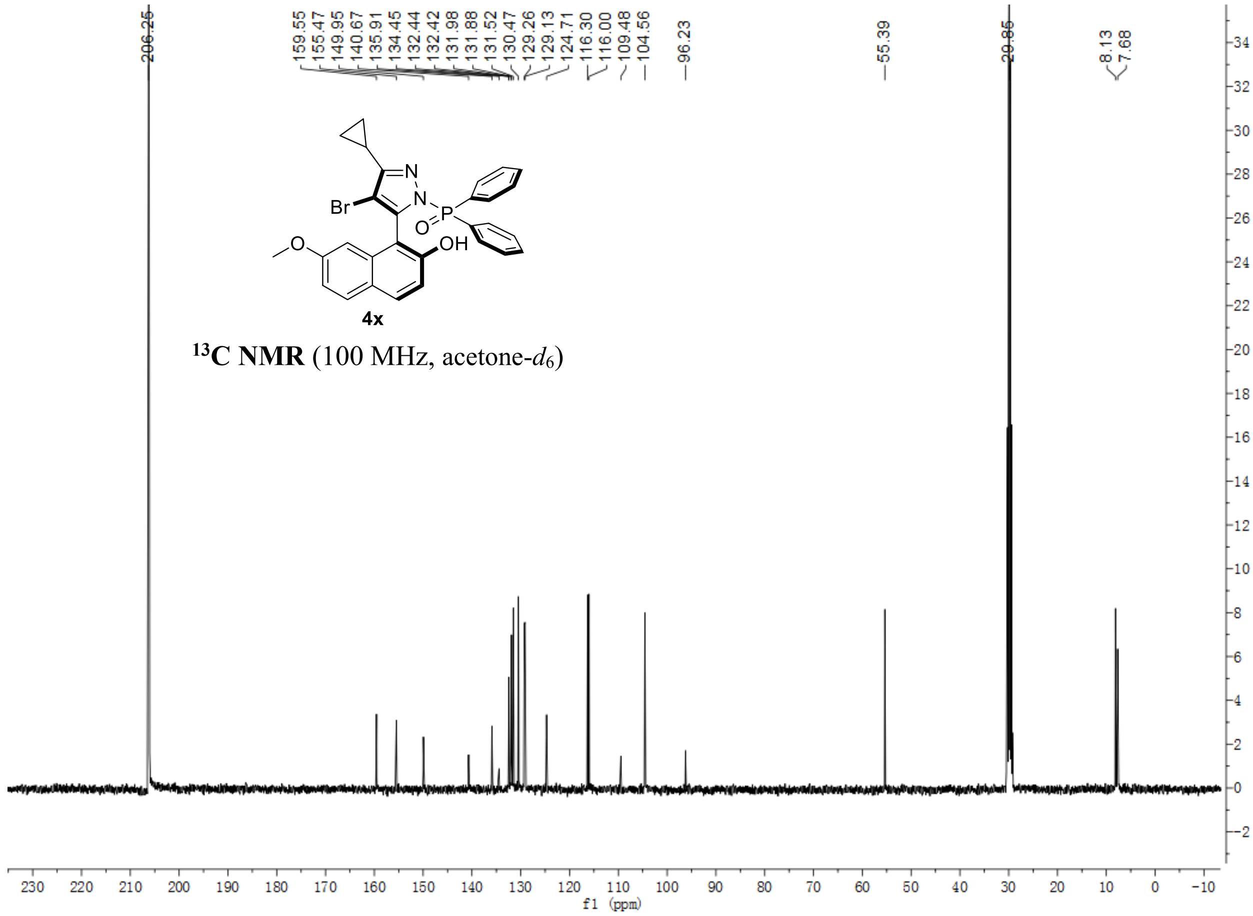
206.25
159.55 155.47 149.95 140.67 135.91 134.45 132.44 132.42 131.98 131.88 131.52 130.47 129.26 129.13 124.71 116.30 116.00 109.48 104.56
96.23
55.39
29.85
8.13 7.68
Br
N
N
P
O
OH
4x
13C NMR (100 MHz, acetone-*d*6)
f1 (ppm)

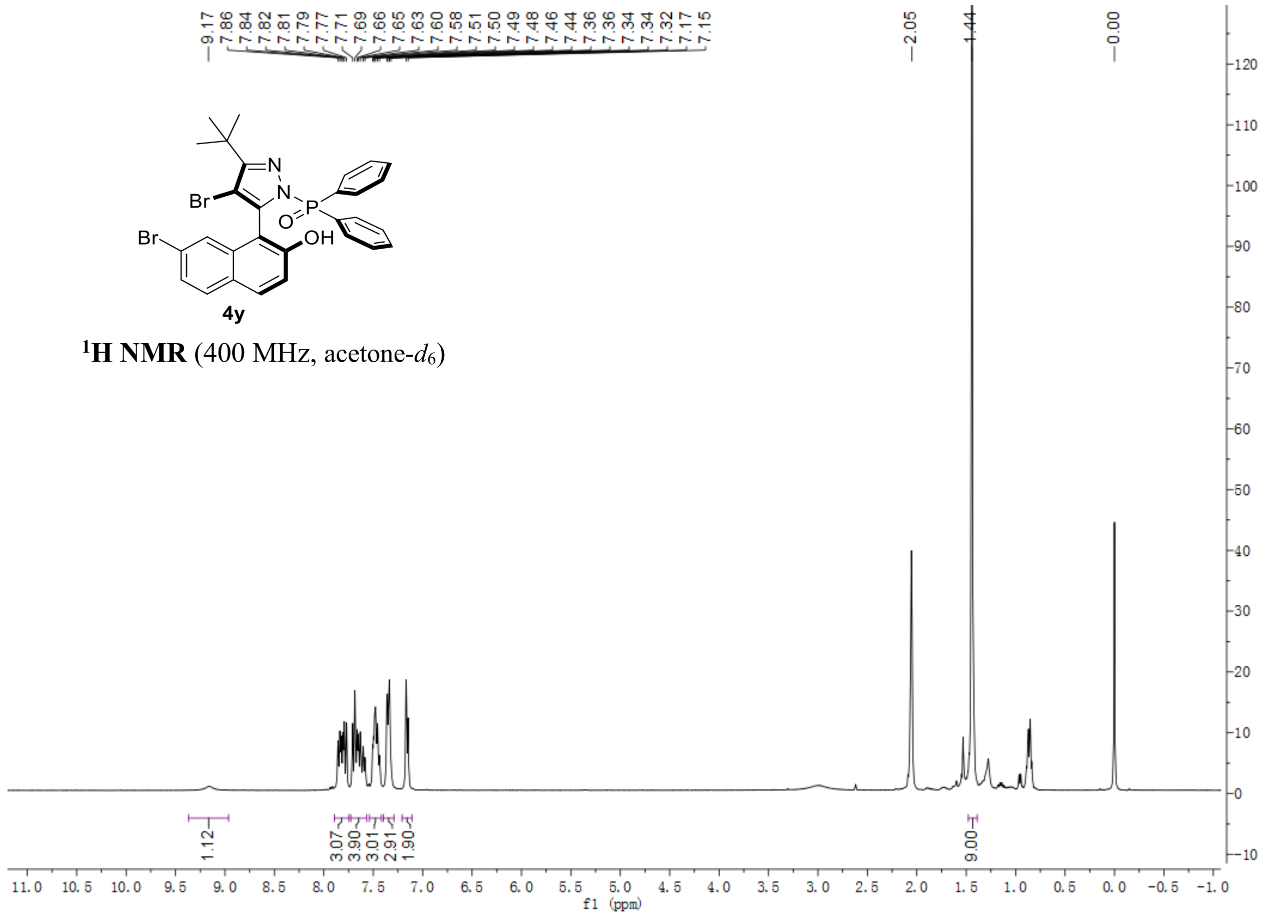

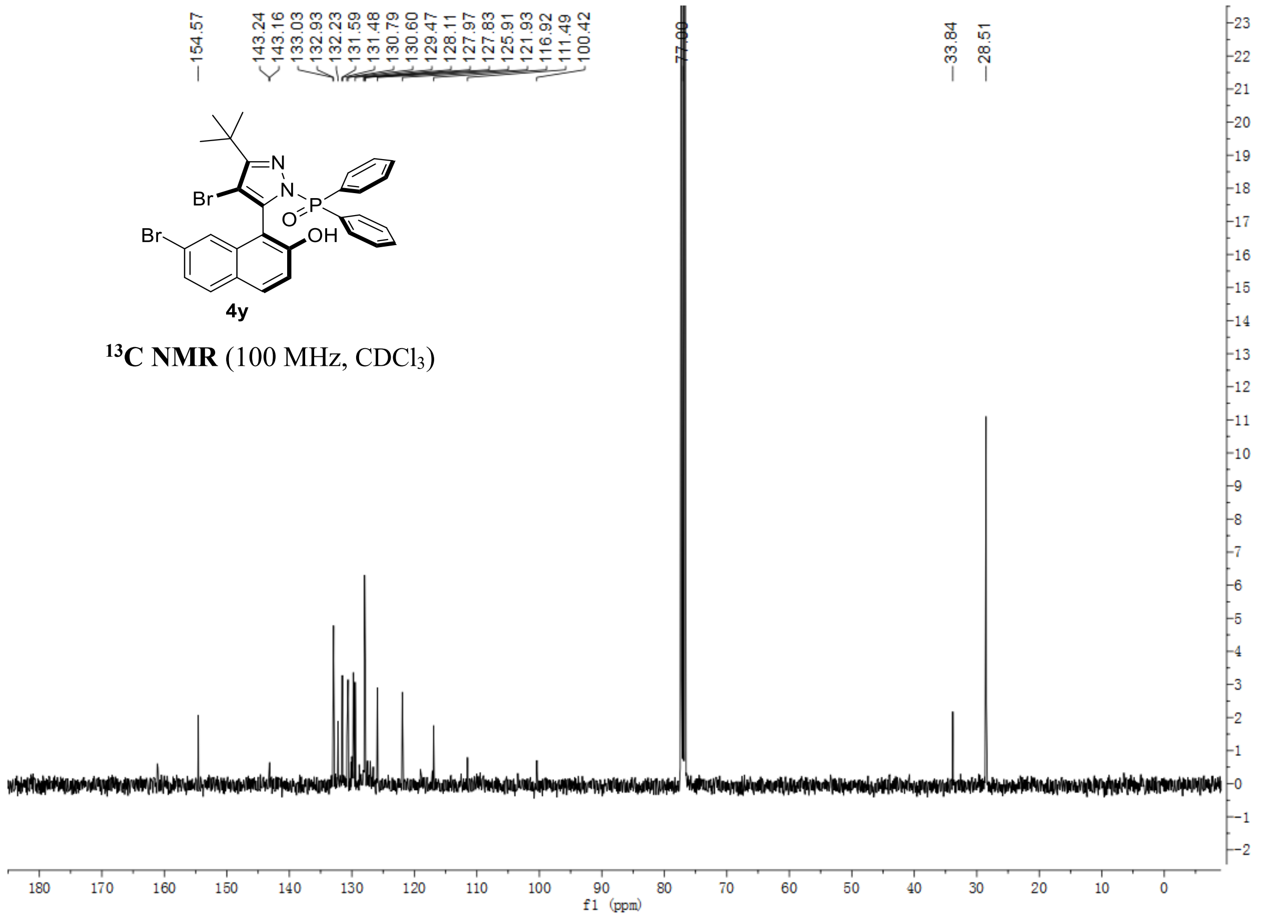

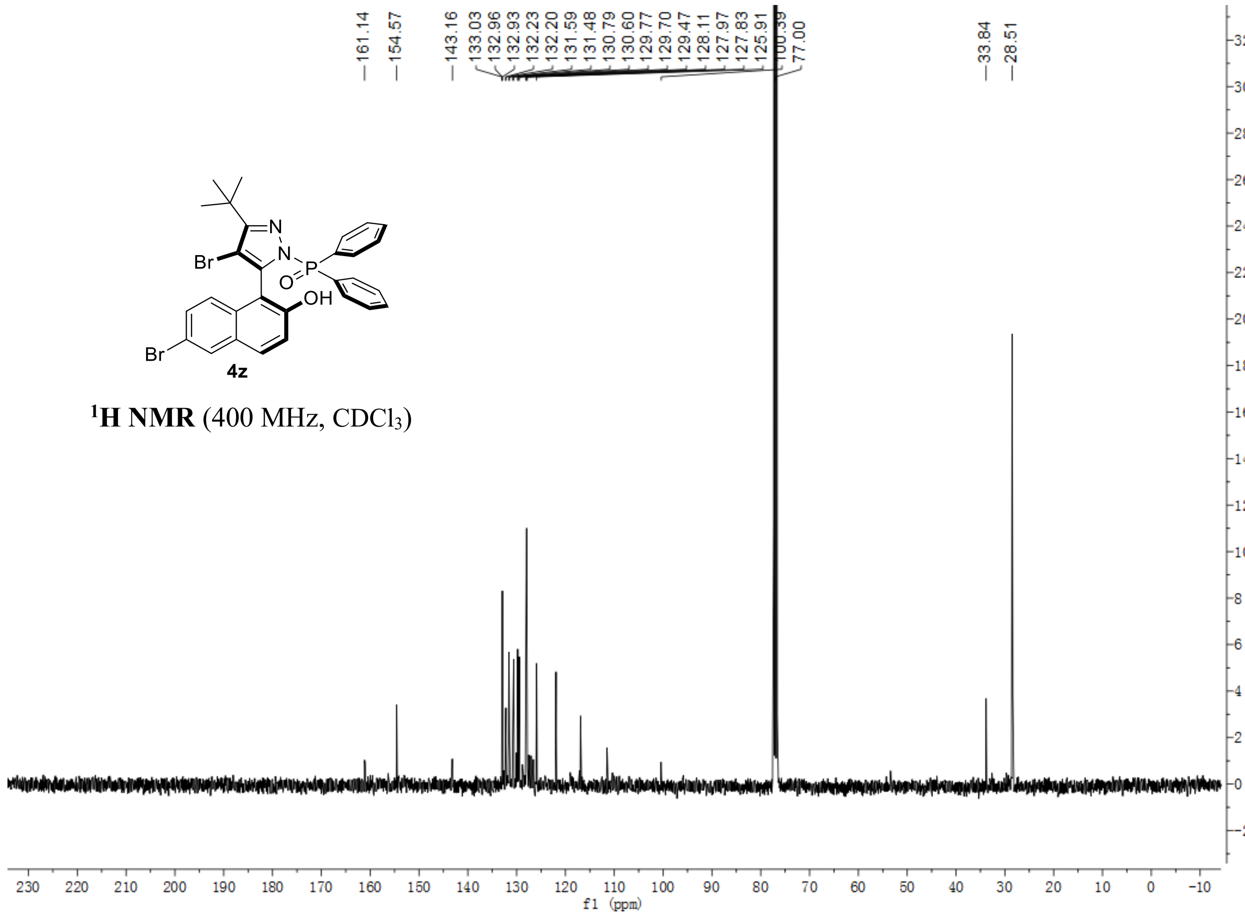

4z

^{1}H NMR (400 MHz, $CDCl_3$)

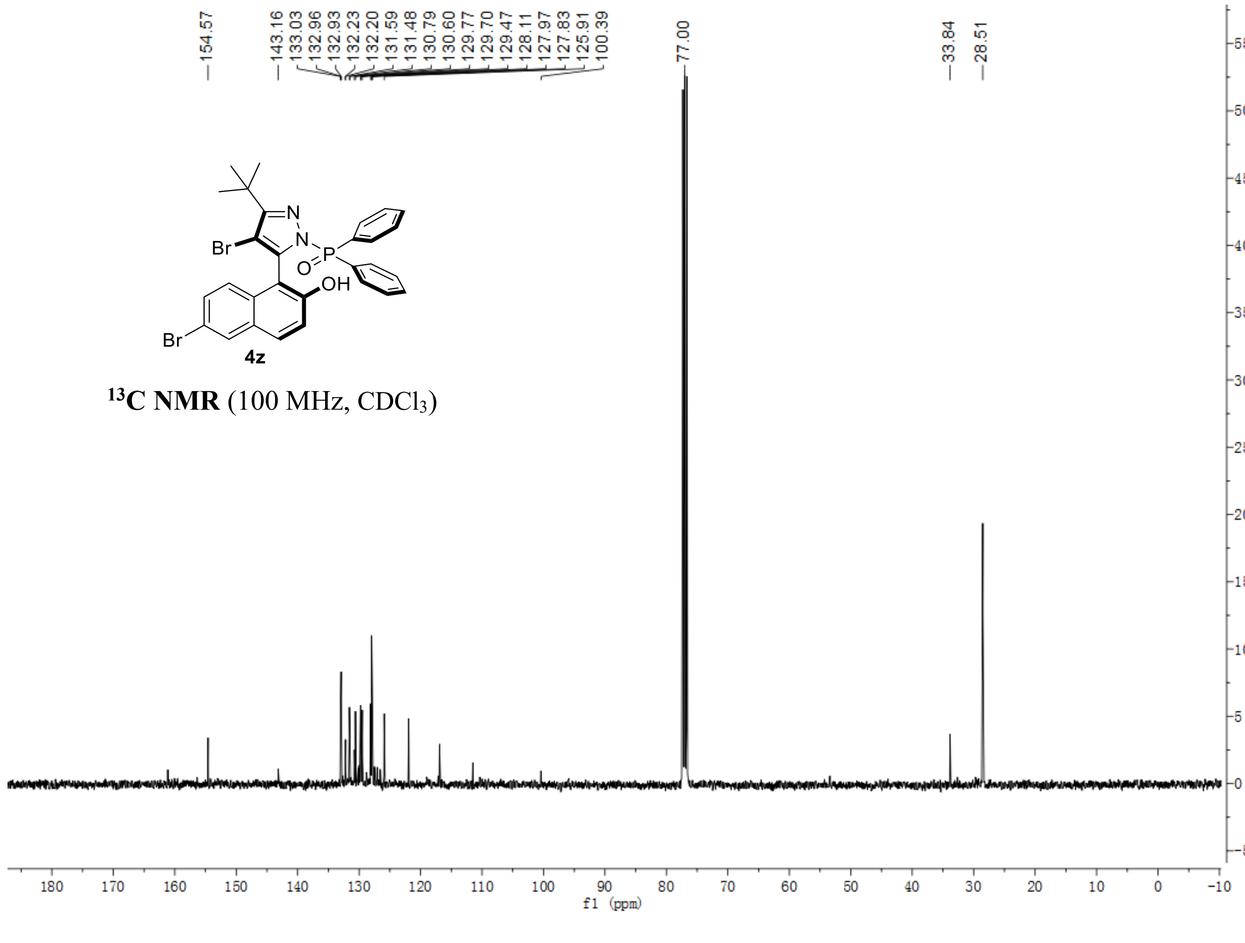

4z

^{13}C NMR (100 MHz, $CDCl_3$)

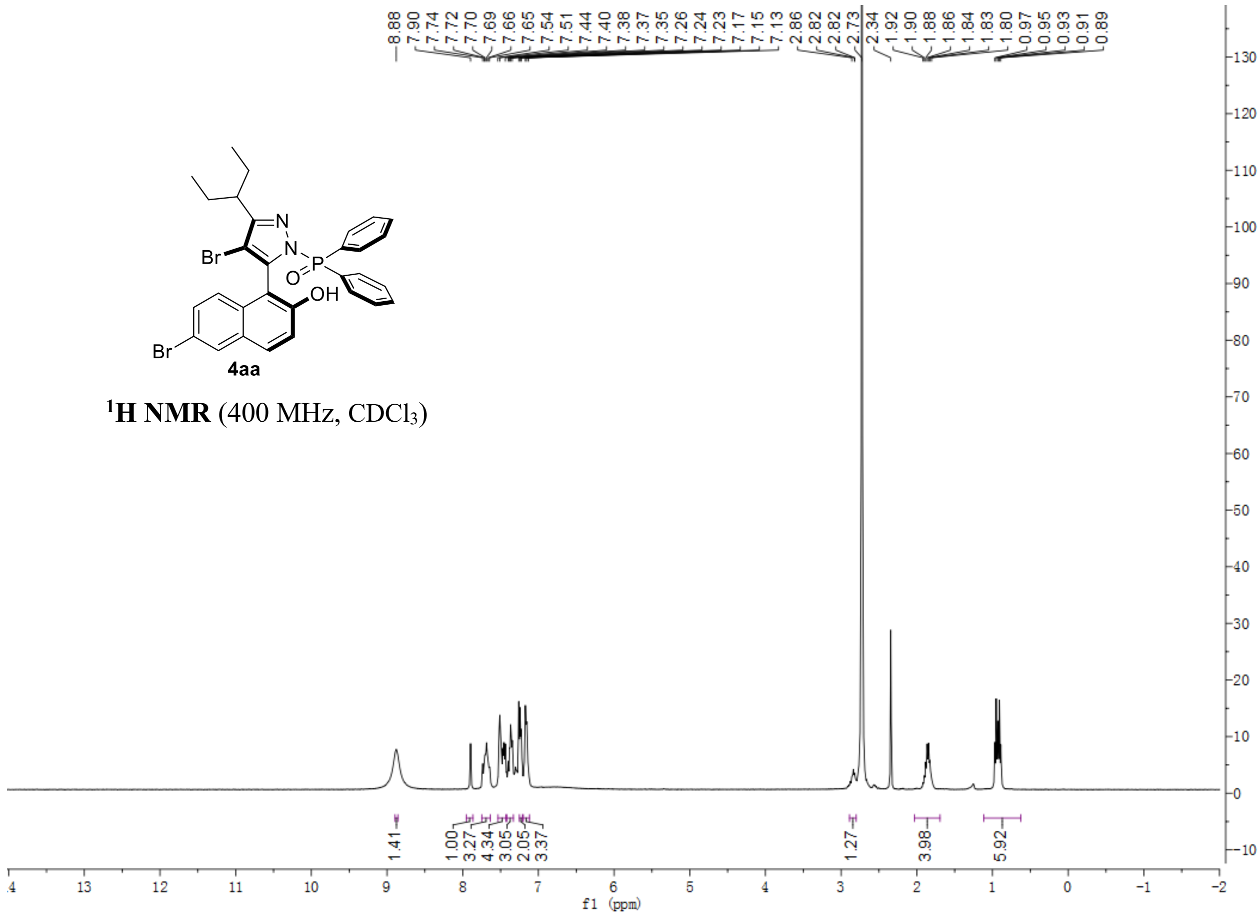
4aa
^{1}H NMR (400 MHz, CDCl$_3$)

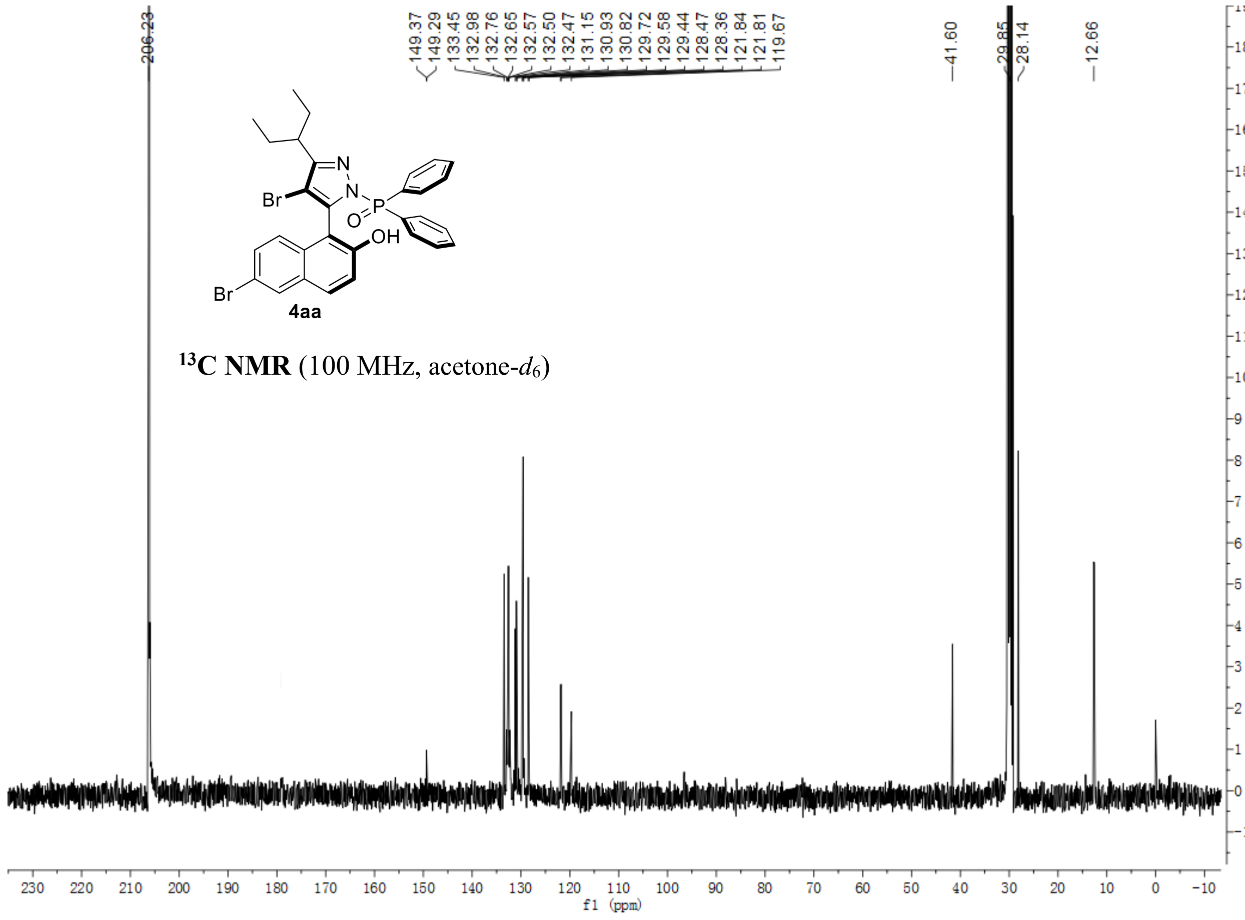
4aa
^{13}C NMR (100 MHz, acetone-d_6)

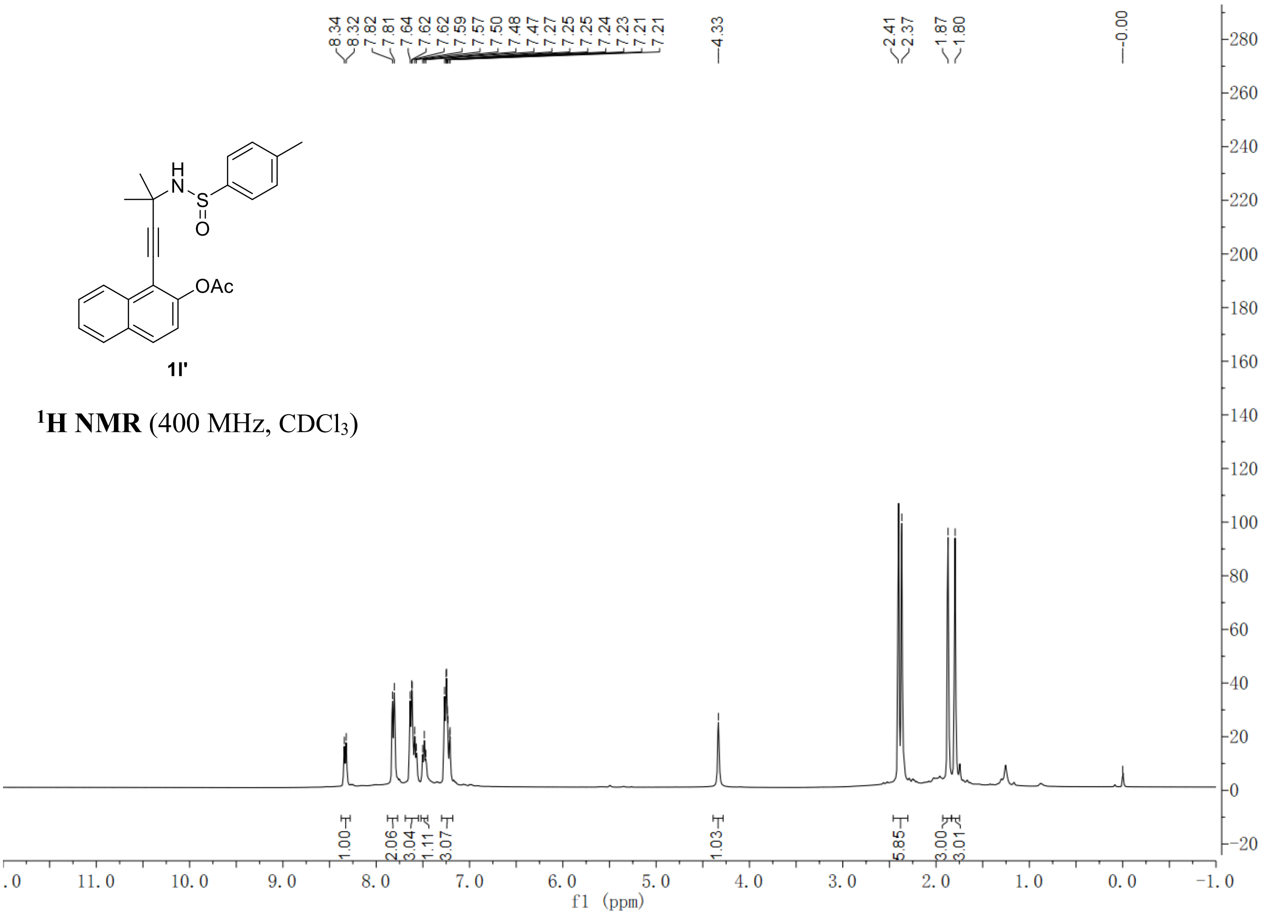

1l'

^{1}H NMR (400 MHz, $CDCl_3$)

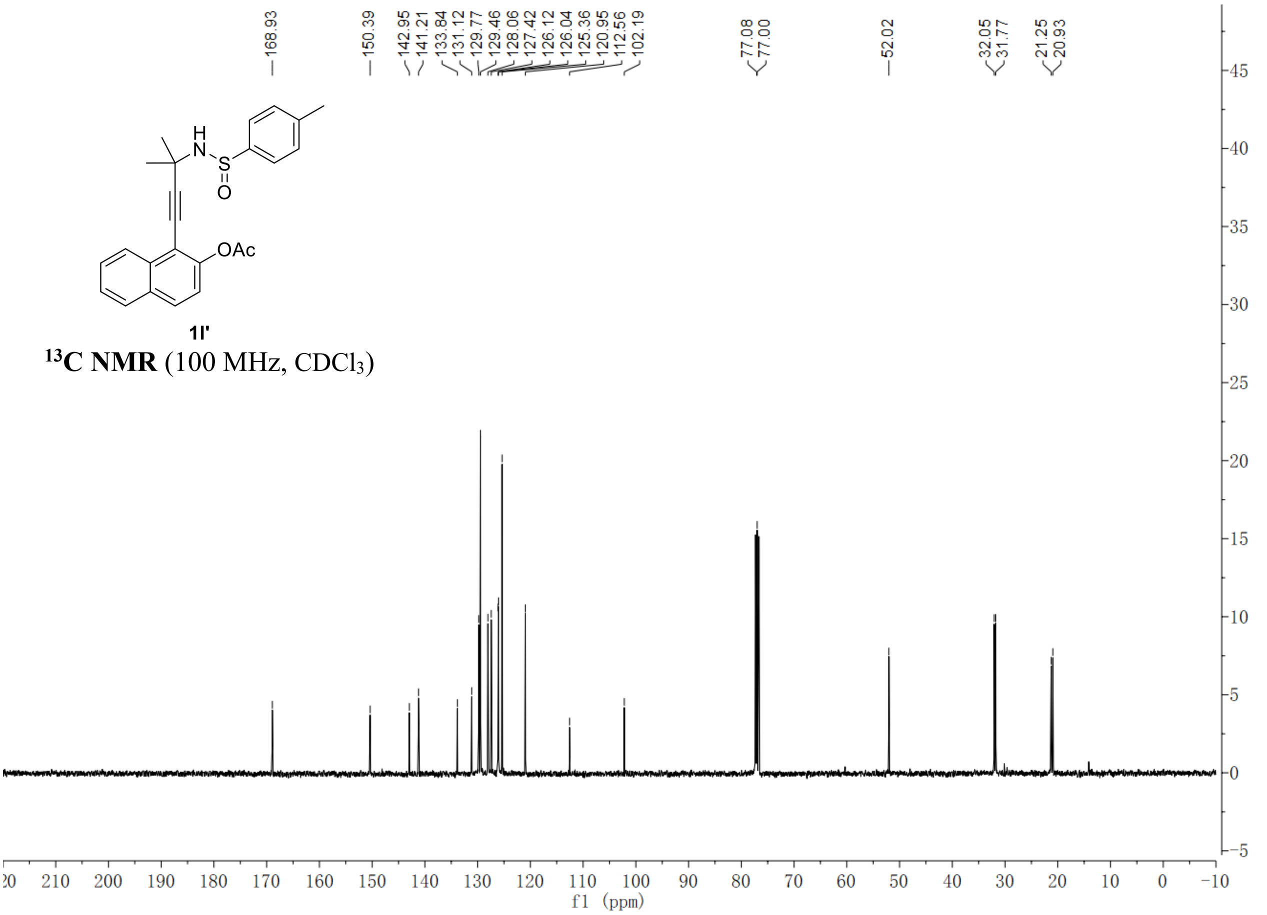

1l'

^{13}C NMR (100 MHz, $CDCl_3$)

1l''

^{1}H NMR (400 MHz, CDCl$_3$)

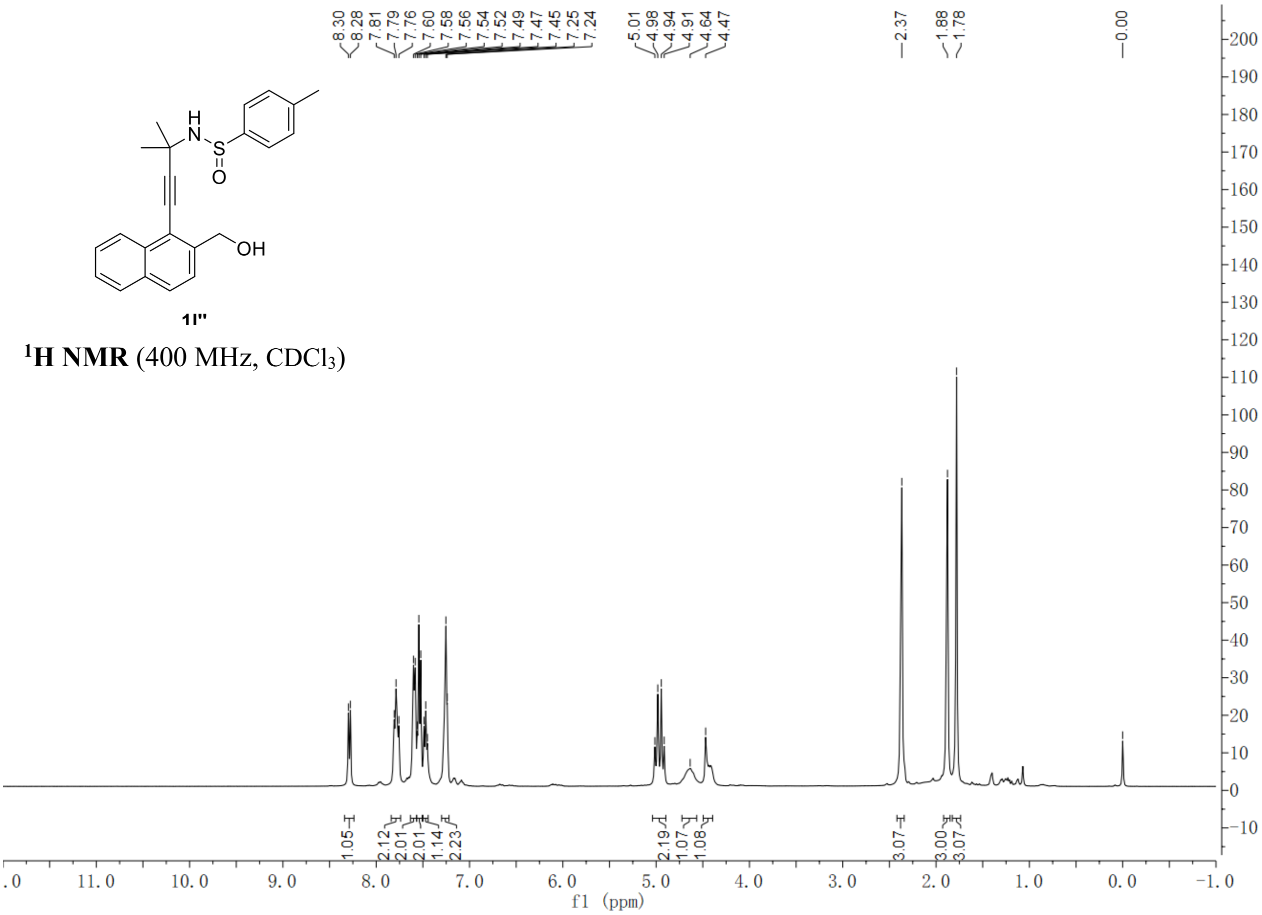

1l''

^{13}C NMR (100 MHz, CDCl$_3$)

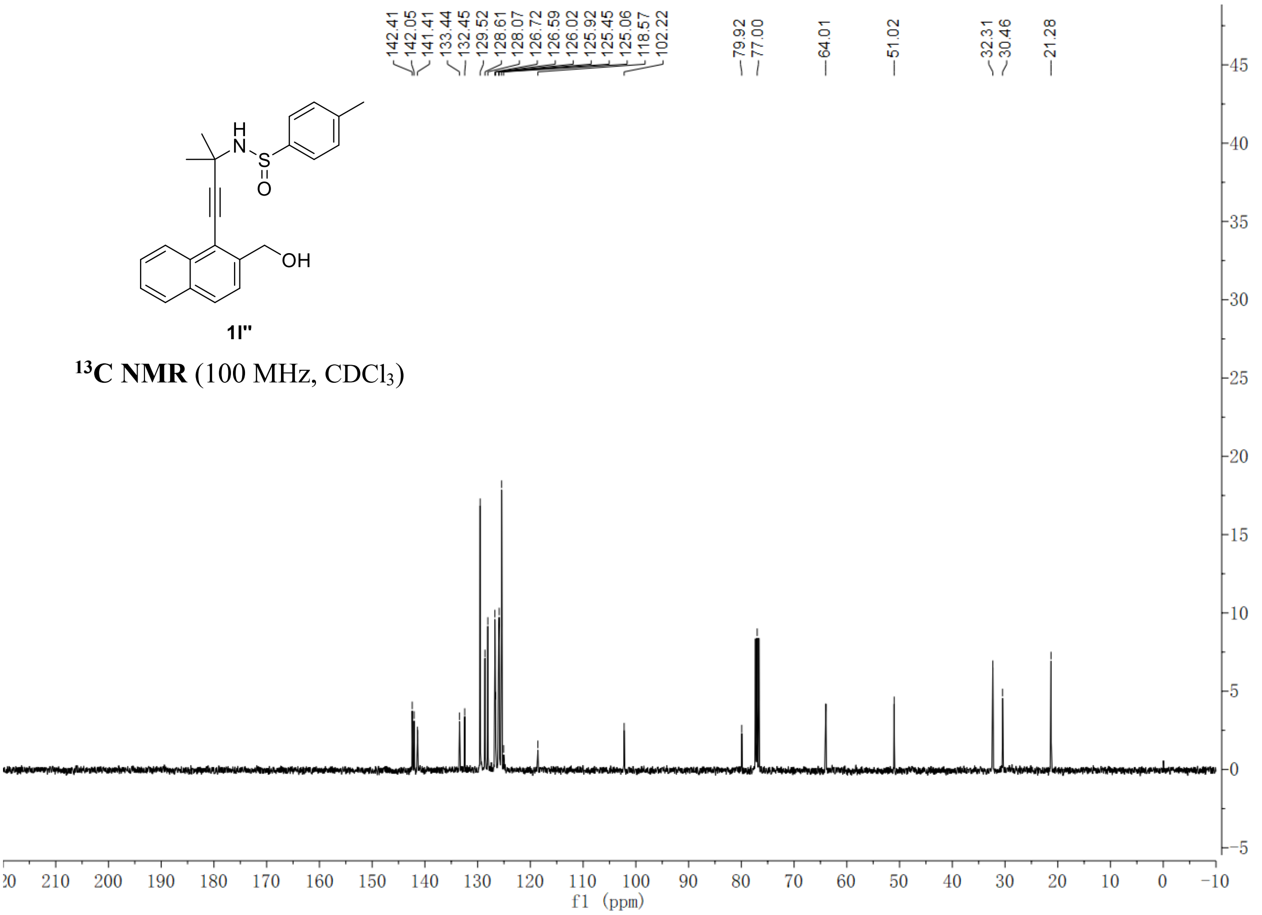

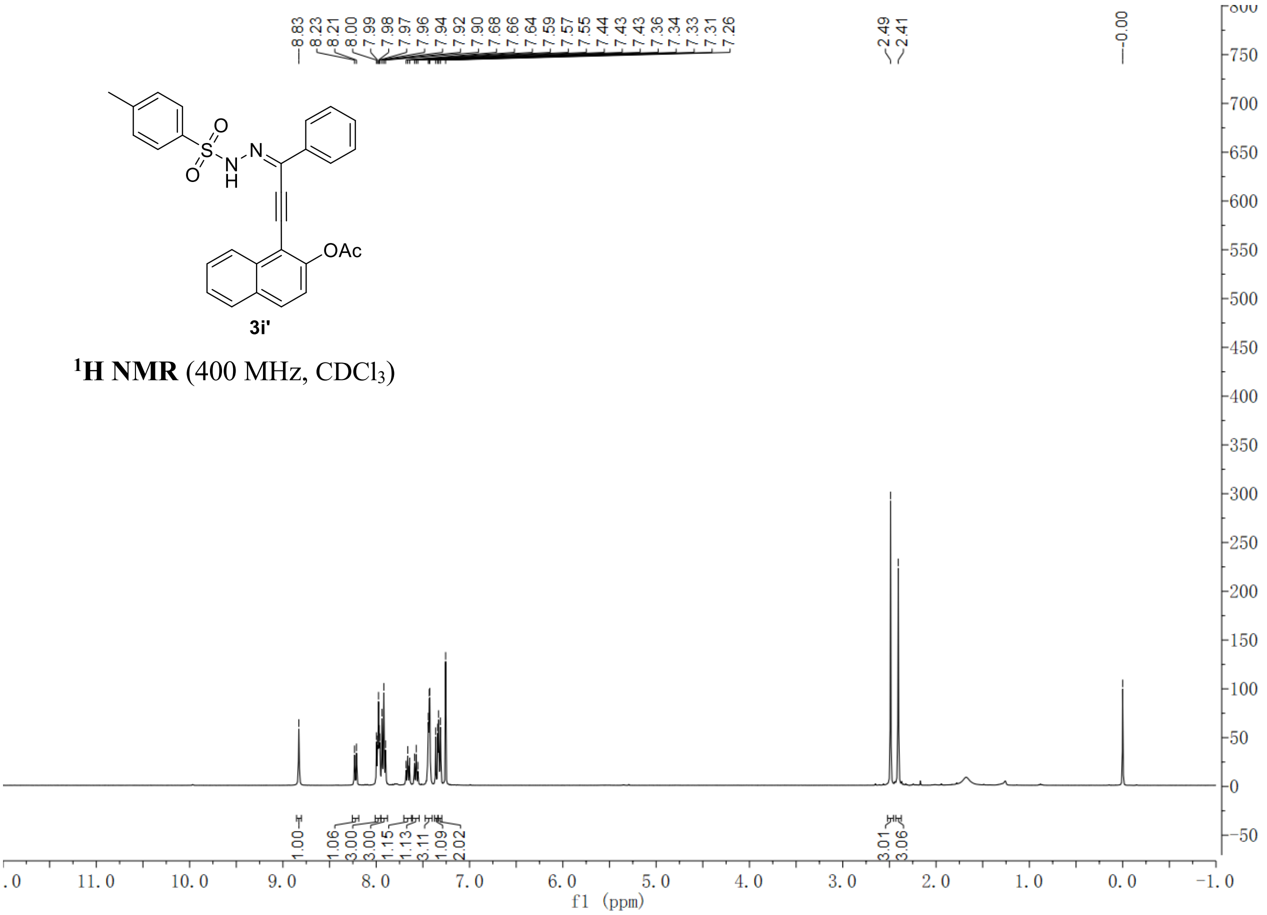

^{1}**H NMR** (400 MHz, $CDCl_3$)

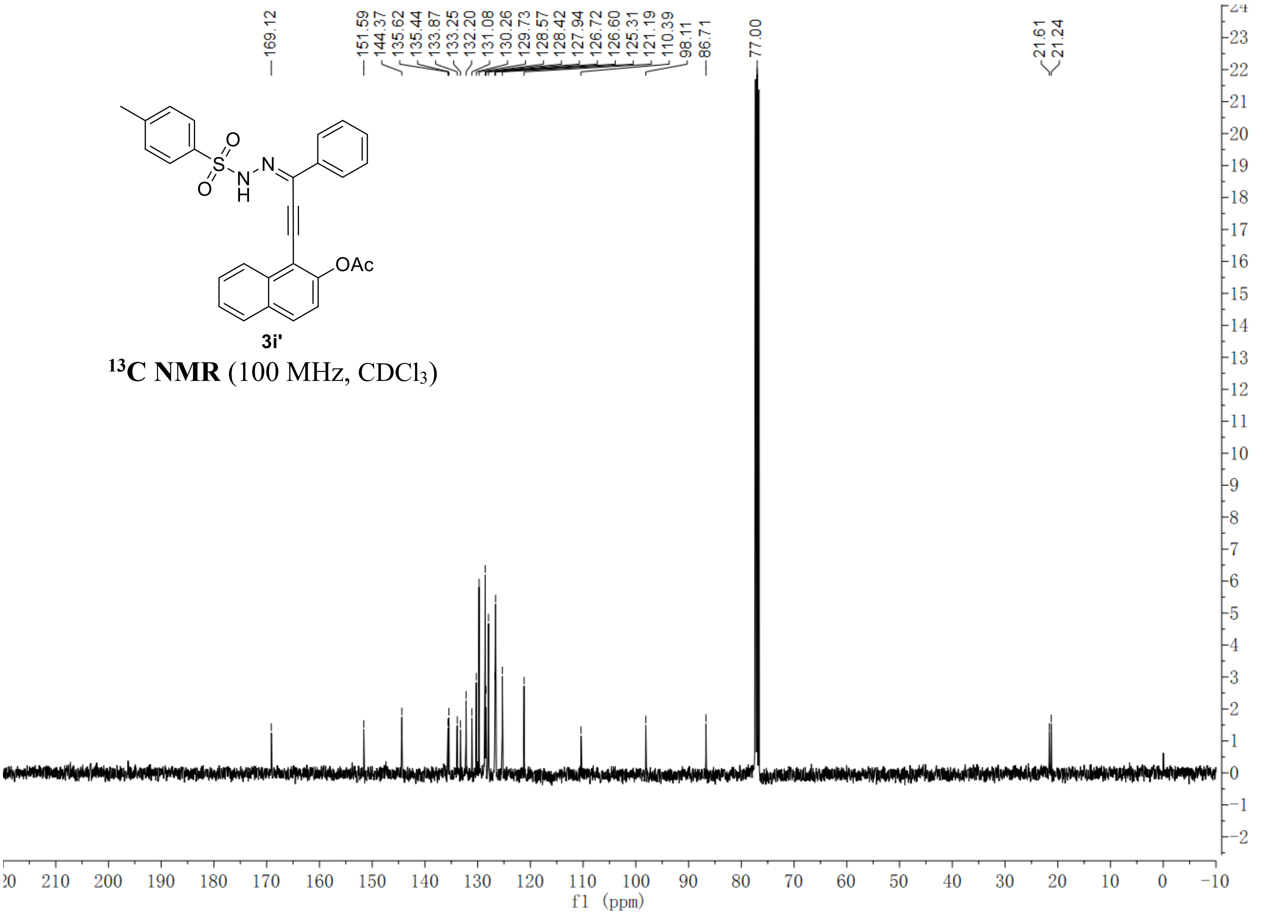

^{13}**C NMR** (100 MHz, $CDCl_3$)

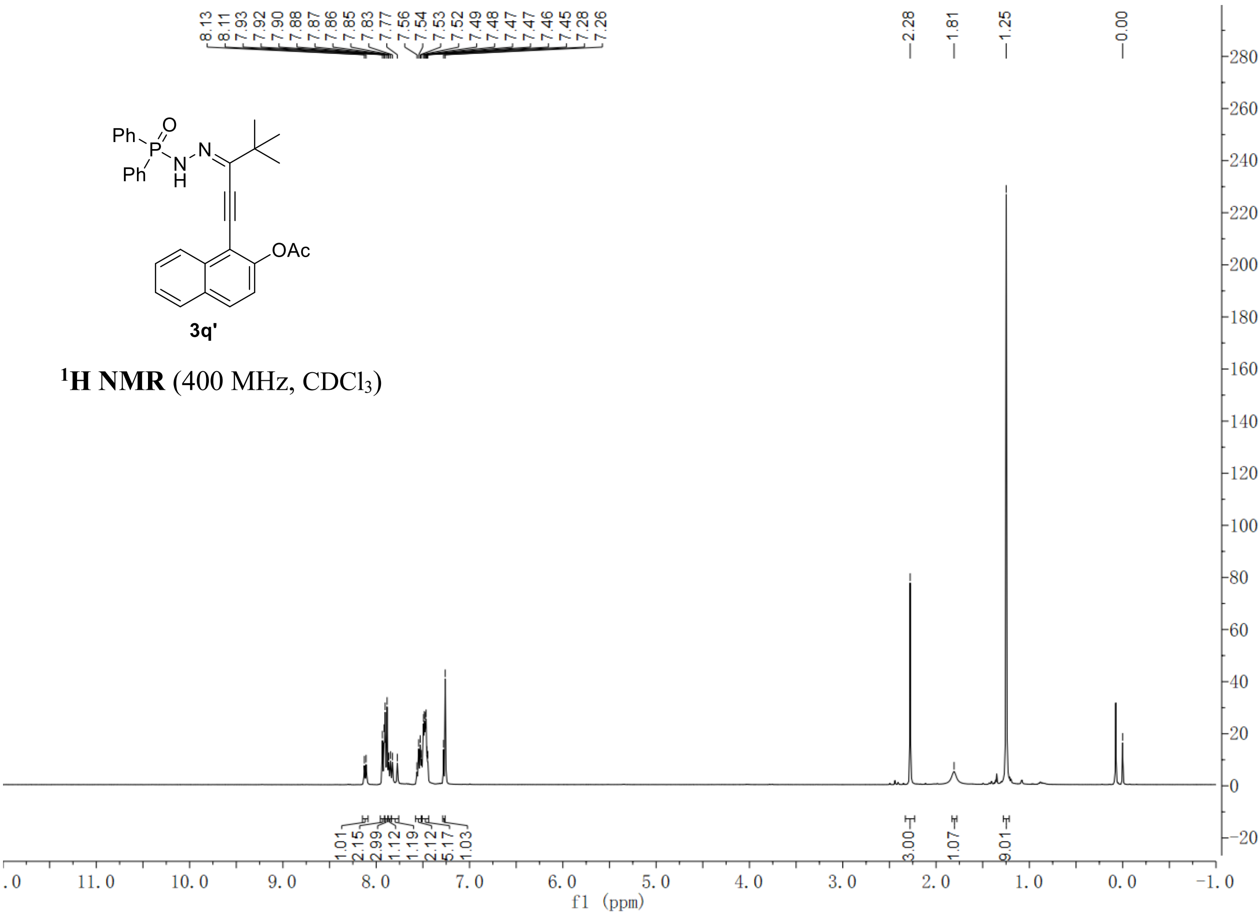
8.13 8.11 7.93 7.92 7.90 7.88 7.87 7.86 7.85 7.83 7.77 7.56 7.54 7.53 7.52 7.49 7.48 7.47 7.46 7.45 7.28 7.26
2.28
1.81
1.25
0.00
3q'
^{1}H NMR (400 MHz, $CDCl_3$)
f1 (ppm)

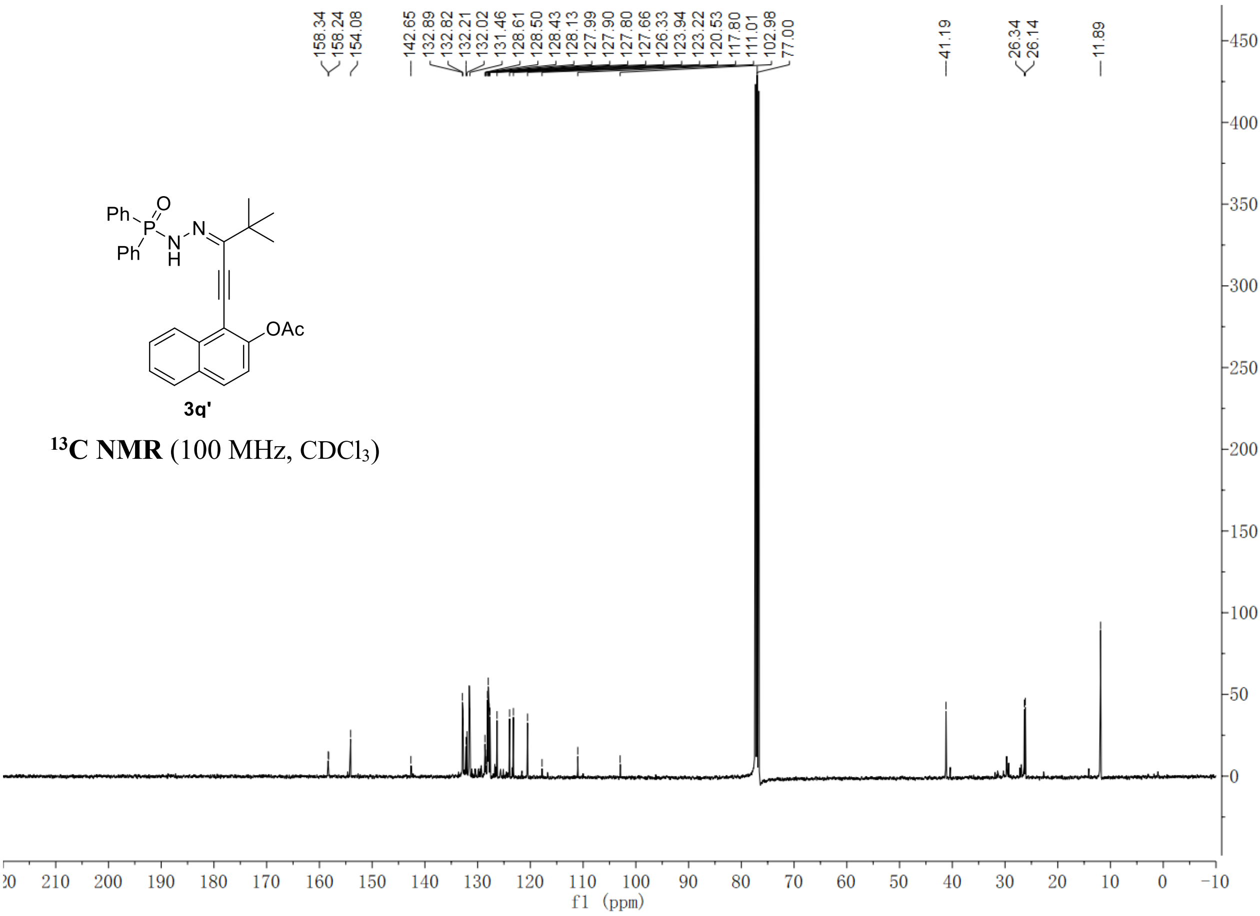
158.34 158.24 154.08
142.65 132.89 132.82 132.21 132.02 131.46 128.61 128.50 128.43 128.13 127.99 127.90 127.80 127.66 126.33 123.94 123.22 120.53 117.80 111.01 102.98 77.00
41.19
26.34 26.14
11.89
3q'
^{13}C NMR (100 MHz, $CDCl_3$)
f1 (ppm)

VIII. X-ray crystallographic information

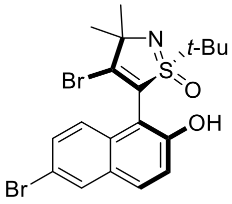

2e

≡

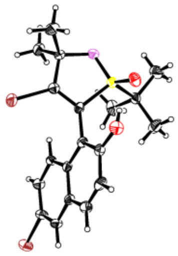

CCDC 2110586

Bond precision:	C-C = 0.0092 A		Wavelength = 0.71073
Cell:	a=8.4504(2)	b=11.8494(2)	c=22.1690(5)
	alpha=90	beta=93.171(3)	gamma=90
Temperature:	293 K		
	Calculated	Reported	
Volume	2216.43(8)	2216.43(8)	
Space group	P 21	P 1 21 1	
Hall group	P 2yb	P 2yb	
Moiety formula	C19 H21 Br2 N O2 S [+ solvent]	C19 H21 Br2 N O2 S	
Sum formula	C19 H21 Br2 N O2 S [+ solvent]	C19 H21 Br2 N O2 S	
Mr	487.23	487.25	
Dx,g cm-3	1.460	1.460	
Z	4	4	
Mu (mm-1)	3.762	3.762	
F000	976.0	976.0	
F000’	974.60		
h,k,lmax	11,16,30	11,16,30	
Nref	11815[6181]	10067	
Tmin,Tmax	0.312,0.336	0.757,1.000	
Tmin’	0.288		

Correction method= # Reported T Limits: Tmin=0.757 Tmax=1.000

AbsCorr = MULTI-SCAN

Data completeness= 1.63/0.85	Theta(max)= 29.032
R(reflections)= 0.0443(7331)	wR2(reflections)= 0.0976(10067)
S =1.013	Npar = 463

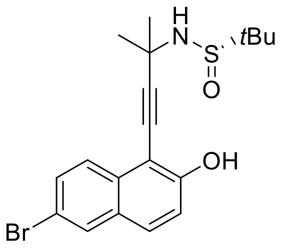

1e

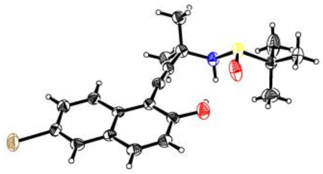

CCDC 2110585

Bond precision:	C-C = 0.0123 A		Wavelength = 0.71073
Cell:	a=9.6180(5)	b=20.7242(8)	c=10.1734(4)
	alpha=90	beta=106.209(5)	gamma=90
Temperature:	295 K		
	Calculated	Reported	
Volume	1947.21(16)	1947.21(15)	
Space group	P 21	P 1 21 1	
Hall group	P 2yb	P 2yb	
Moiety formula	C19 H22 Br N O2 S	2(C19 H22 Br N O2 S)	
Sum formula	C19 H22 Br N O2 S	C38 H44 Br2 N2 O4 S2	
Mr	408.34	816.69	
Dx,g cm-3	1.393	1.393	
Z	4	2	
Mu (mm-1)	2.229	2.229	
F000	840.0	840.0	
F000’	839.64		
h,k,lmax	13,28,13	13,28,13	
Nref	10405[5340]	8941	
Tmin,Tmax	0.495,0.536	0.667,1.000	
Tmin’	0.485		

Correction method= # Reported T Limits: Tmin=0.667 Tmax=1.000

AbsCorr = MULTI-SCAN

Data completeness= 1.67/0.86   Theta(max)= 29.057

R(reflections)= 0.0564(4176)   wR2(reflections)= 0.1389(8941)

S = 0.970   Npar= 451

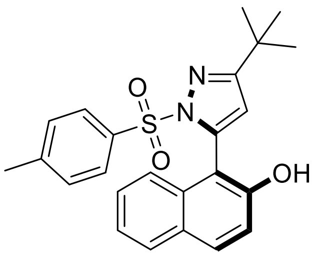

4a

≡

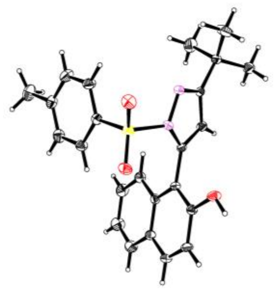

CCDC 2091783

Bond precision:	C-C = 0.0062 A		Wavelength= 0.71073
Cell:	a= 8.8686(4)	b= 10.6372(5)	c= 11.7142(7)
	alpha=90	beta=94.076(5)	gamma=90
Temperature:	293 K		
	Calculated	Reported	
Volume	1096.90(10)	1096.90(10)	
Space group	P 21	P 1 21 1	
Hall group	P 2yb	P 2yb	
Moiety formula	C24 H24 N2 O3 S	C24 H24 N2 O3 S	
Sum formula	C24 H24 N2 O3 S	C24 H24 N2 O3 S	
Mr	420.51	420.51	
Dx,g cm-3	1.273	1.273	
Z	2	2	
Mu (mm-1)	0.175	0.175	
F000	444.0	444.0	
F000’	444.43		
h,k,lmax	12,14,16	11,14,15	
Nref	5881[3092]	4972	
Tmin,Tmax	0.941,0.952	0.825,1.000	
Tmin’	0.941		

Correction method= # Reported T Limits: Tmin=0.825 Tmax=1.000
AbsCorr = MULTI-SCAN

Data completeness= 1.61/0.85	Theta(max)= 29.100
R(reflections)= 0.0494(3868)	wR2(reflections)= 0.1148(4972)
S = 1.068	Npar= 276

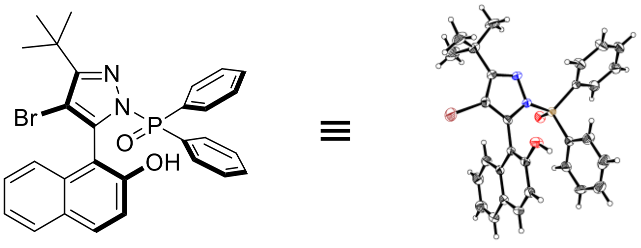

4q **CDCC 2092005**

Bond precision:	C-C = 0.0079 A		Wavelength=0.71073
Cell:	a=7.6050(4)	b=14.1761(5)	c=24.385(1)
	alpha=90	beta=90	gamma=90
Temperature:	293 K		
	Calculated	Reported	
Volume	2628.9(2)	2628.93(19)	
Space group	P 21 21 21	P 21 21 21	
Hall group	P 2ac 2ab	P 2ac 2ab	
Moiety formula	C29 H26 Br N2 O2 P	C29 H26 Br N2 O2 P	
Sum formula	C29 H26 Br N2 O2 P	C29 H26 Br N2 O2 P	
Mr	545.39	545.40	
Dx,g cm-3	1.378	1.378	
Z	4	4	
Mu (mm-1)	1.653	1.653	
F000	1120.0	1120.0	
F000’	1119.64		
h,k,lmax	10,19,33	10,19,33	
Nref	7070[3991]	5952	
Tmin,Tmax	0.615,0.640	0.683,1.000	
Tmin’	0.603		

Correction method= # Reported T Limits: Tmin=0.683 Tmax=1.000
AbsCorr = MULTI-SCAN

Data completeness= 1.49/0.84	Theta(max)= 29.136
R(reflections)= 0.0530(4105)	wR2(reflections)= 0.1268(5952)
S = 1.023	Npar= 32

Biological evaluation

IX. COX-2 inhibitory assay

The ability of compounds to inhibit COX-2 was determined using a COX-2 inhibitor screening assay kit according to the instruction manual (Biyuntian Biotech Co., Ltd., Shanghai, China). The kit measures the conversion of arachidonic acid to prostaglandin G2 by COX-2. In brief, a recombinant human COX-2 protein in a black 96-well plate was mixed with the test compounds at varying concentrations or with celecoxib, a selective COX-2 inhibitor as a positive control. Following a 10-min incubation at 37 °C, the plates were read on a Spectramax microplate reader (Molecular Devices, US) using a setting of ex/em at 570 nm and 590 nm, respectively. The inhibition rate on COX-2 activity was calculated following the manufacturer's instruction. Compound **4n** and **4l** did not show obvious inhibitory effect on COX-2 enzyme with doses up to 10 μM, while celecoxib was detected to inhibit the enzyme activity by 78.2% at 100 nM.

X. Cell culture

Six cancer cell lines A549, MIA PaCa-2, A375, MCF7, MDA-MB-231, Hela and human hepatocyte cell line L02 were used in this work. All cell lines were grown in Dulbecco’s modified Eagle’s medium (DMEM, Hyclone), except for A375, which was maintained in Gibco DMEM (High Glucose) with sodium pyruvate (Invitrogen) added. All media were supplemented with 10% foetal bovine serum (FBS, BI), 100 units/mL of penicillin–streptomycin (Sigma-Aldrich), incubated at 37 ºC in a humidified atmosphere containing 5% CO_2.

XI. Anti-proliferation assay

The anti-proliferative activities of the prepared compounds against the mentioned cell lines were evaluated using a standard (MTT)-based colorimetric assay with some modification. Cell lines were grown to log phase in DMEM supplemented with 10% foetal bovine serum. Cell suspensions were prepared and 100 μL/well dispensed into 96-well plates to give 2 x 10^3 cells/well. After incubation for 24 h, cells were treated with the target compounds at 1.56, 3.125, 6.25, 12.5, 25, 50 μM and incubated for 48 h at 37 ºC in a humidified atmosphere of 5% CO_2. Afterwards, cell viability was assessed by the conventional 3-(4, 5-dimethylthiazol-2-yl)-2, 5-diphenyltetrazolium bromide (MTT) reduction assay carried out strictly according to the manufacturer’s instructions (Sigma). The absorbance (OD_{570}) was read on a Spectramax microplate reader (Molecular Devices,

US). In all experiments, three replicate wells were used for each drug concentration. Each assay was performed at least three times.

XII. Cell apoptosis analysis

Approximately 10^5 cells/well of A375 cells were plated in a 12-well plate and allowed to adhere. Subsequently, the medium was replaced with fresh culture medium containing compound **4x** at different concentrations (0, 2, 4 and 8 μM) or 4 μM compound **4x** at different times (0, 12, 24 and 48 h). Non-treated wells received an equivalent volume of ethanol (<0.1%). After treatment, cells were trypsinized, washed in PBS and centrifuged at 2000 rpm for 5 min. The pellet was resuspended in 500 μL staining solution (containing 5 μL AnnexinV-FITC and 5 μL PI (5 mg/mL) in binding buffer), mixed gently and incubated for 15 min at room temperature in dark. The samples were then analyzed by a FACSCalibur flow cytometer (Becton Dickinson, US), plotting at least 10000 events in the gate area per sample. Forward and side scatter gating strategy was used in flow cytometry analysis to identify the cells of interest based on the relative size and to remove debris and other events that are not of interest. Cells were selected in FSC-A/SSC-A dot plot to remove debris.

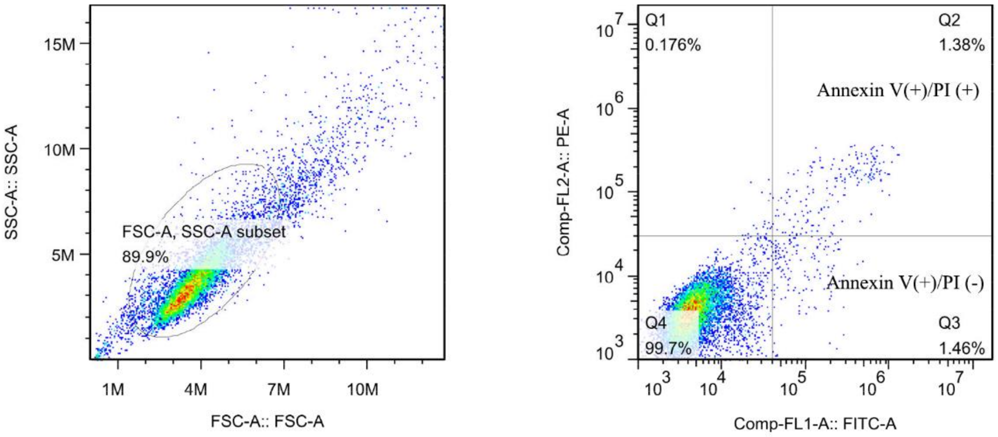

Supplementary Figure 9. The example of gating strategy in cell apoptosis. The percentage of early apoptotic cells in the lower right quadrant (Annexin V positive/PI negative cells), as well as late apoptotic cells located in the upper right quadrant (Annexin V positive/PI positive cells).

XIII. Cell cycle analysis

A375 Cells were plated in 6-well plates (10^6 cells per well) and, and serum-starved for 24 h. Cells were then incubated with compound **4x** at different concentrations (0, 2, 4 and 8 μM) or 4 μM compound **4x** at different times (0, 12, 24 and 48 h). After treatment, cells were centrifuged at 1500 rpm at 4 ºC for 5 min, fixed

in 70% ethanol at 4 ºC for at least 12 h and subsequently resuspended in phosphate buffered saline (PBS) containing 0.1 mg/mL RNase A and 5 mg/mL propidium iodide (PI). The cellular DNA content was measured by flow cytometry (Becton Dickinson, US) for cell cycle distribution analysis, plotting at least 20000 events in the gate area per sample. The percentage of cells in different phases of the cell cycle were determined using Flowjo 10 software. Forward and side scatter gating strategy was used in flow cytometry analysis to identify the cells of interest based on the relative size and to remove debris and other events that are not of interest. Cells were selected in FSC-A/SSC-A dot plot to remove debris; single cells were gated using the PE-A/PE-H dot plot.

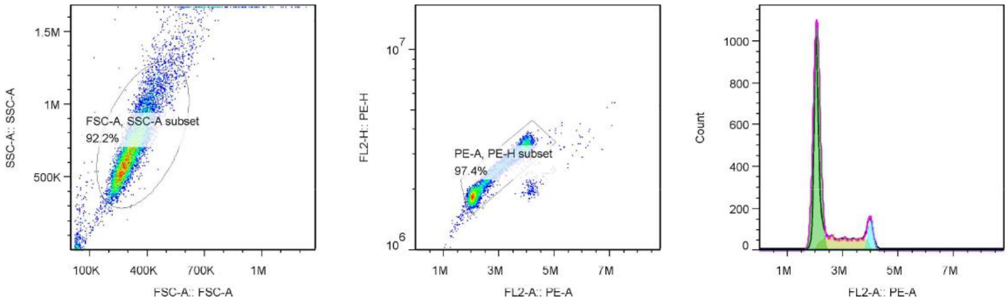

Supplementary Figure 10. The example of gating strategy in cell cycle analysis.

XIV. ROS measurement

Intracellular ROS levels were examined using 2,7-dichloro-fluorescein diacetate (DCFH-DA). A375 cells were seeded in 6-well plates (10^6 cells/well) for 24 h and then treated with compound **4x** (0, 2, 4 and 8 μM) for 12 h. After treatment, according to the manufacturer's instructions (Thermo Fisher Scientific, USA), 400 mL of 10 μM DCFH-DA was added to each well and incubated for 20 min. Stained cells were washed three times with serum-free culture medium and analyzed using a flow cytometer. The expression level of ROS is shown by fluorescence intensity. Forward and side scatter gating strategy was used in flow cytometry analysis to identify the cells of interest based on the relative size and to remove debris and other events that are not of interest. Cells were selected in FSC-A/SSC-A dot plot to remove debris.

XV. Mitochondrial membrane potential evaluation

Mitochondrial transmembrane potential ($\Delta\Psi m$) was detected using a JC-1 mitochondrial membrane potential assay kit (Beyotime Biotech). After the treatment of **4x**, the A375 cells were incubated at 37 ºC for 20 min with 5 μg/mL JC-1 (5,5,6,6-tetrachloro-1,1,3,3-tetraethylbenzimidazolylcarbocyanine iodide), then washed twice with PBS and placed in fresh medium without serum. The samples were analyzed using a FACSCalibur

cytometer (Becton Dickinson) equipped with a 488 nm argon laser. Forward and side scatter gating strategy was used in flow cytometry analysis to identify the cells of interest based on the relative size and to remove debris and other events that are not of interest. Cells were selected in FSC-A/SSC-A dot plot to remove debris.

XVI. Western blot analysis

After treatment with compound **4x** (0, 2, 4 and 8 μM) for 48 h, A375 cells were harvested and lysed in RIPA buffer for 30 min in an ice bath, and then centrifuged at 12000 rmp for 10 min at 4 ºC. The supernatant was obtained, and protein concentrations were then determined using a BCA assay kit and boiled for 10 min at 100 ºC. Equal amounts of protein (20 μg) were separated on a 10% or 15% SDS-PAGE gel and transferred onto polyvinylidene difluoride membranes. The membranes were blocked with 5% BSA and pro-bed with primary antibodies (1:500 dilution) at 4 ºC with gentle shaking overnight. Then, the membranes were incubated with a 1:2000 dilution of corresponding HRP-conjugated secondary antibodies (Horseradish Peroxidase labeled goat anti-rabbit IGG (H+L)) for 1 h. Immunoreactive bands were visualized using an ECL detection kit (Invitrogen, USA) following the manufacturer's instruction. The intensity of protein bands was quantified using ImageLab analysis software. The relative optical density value represents protein expression.

XVII. Druglikeness and activity cliff

The Lipinski's "rule of five" (RO5) defines physicochemical parameter ranges (MW ≤ 500, log P ≤ 5, H-bond donors ≤ 5, H-bond acceptors ≤ 10 and rotatable bonds ≤ 10) associated with orally active drugs.[10] Most approved drugs follow at least three rules despite the administration form, and RO5 works as a rule of thumb to evaluate the druglikeness or whether a chemical is likely to be developed as an orally active drug.[11] In our study, most sulfone-containing pyrazoles meet four or all RO5 requirements, with only **4p** violates two rules; for the pyrazoles with diphenylphosphine oxides, all compounds follow at least three rules. The property data of representative compounds are listed in Supplementary Table 7. An activity cliff is generally defined as a pair of analogs with similar structures but having a large difference in potency (with a threshold of 10- or 100-fold).[12] Compound **4x** is more active than analog **4s** (>10 folds) toward A375 cells, suggesting a key role of the OMe substituent in SAR. Besides, the sulfone-containing pyrazoles were generally more cytotoxic than the pyrazoles with diphenylphosphine oxides toward non-cancer cells L02, and **4x** turned to be the most potent

agent. Hence, in this study **4x** was selected to be investigated thoroughly.

Supplementary Table 7. IC50 values and physicochemical properties of representative axial chiral pyrazoles.

	**4a**	**4h**	**4s**	**4x**	**4x′**	**Celecoxib**
Compound	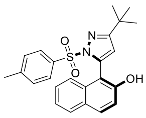	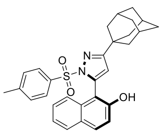	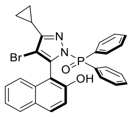	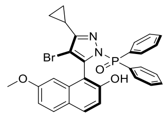	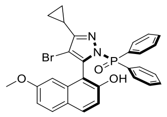	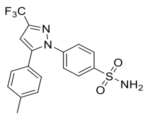
A375 IC_{50} (μM)[a]	10.22 ± 0.33	6.51 ± 0.32	28.15 ± 1.56	2.57 ± 0.004	3.6 ± 0.07	15.92 ± 0.81
MW	420.53	498.6	529.4	559.4	559.4	381.4
log P[b]	4.9	7.36	5.94	5.95	5.95	4.37
H-bond donors	1	1	1	1	1	1
H-bond acceptors	4	4	3	4	4	3
Rotatable bonds	4	4	5	6	6	4

[a]Data are presented as the mean ± SD from at least three independent experiments; [b]Calculated by ChemOffice 2020.

Supplementary Table 8. IC50 values of the tested compounds toward A375 cells.

Compound	IC_{50} (μM)[a]	Compound	IC_{50} (μM)[a]
**4a**	10.22 ± 0.33	**4q**	28.79 ± 1.62
**4c**	27.9 ± 1.83	**4r**	14.94 ± 0.67
**4d**	12.49 ± 0.66	**4s**	28.15 ± 1.56
**4e**	10.46 ± 0.41	**4u**	15.47 ± 0.71
**4f**	13.52 ± 0.55	**4v**	25.16 ± 1.28
**4g**	9.36 ± 0.31	**4w**	20.43 ± 0.98
**4h**	6.51 ± 0.32	**4x**	2.57 ± 0.004
**4i**	7.85 ± 0.35	**4y**	17.73 ± 0.99
**4k**	12.51 ± 0.33	**4z**	15.1 ± 0.84
**4l**	8.91 ± 0.28	**4aa**	8.19 ± 0.47
**4n**	11.11 ± 0.48		

[a]Data are presented as the mean values ± SD from at least three independent experiments.

Supplementary Table 9. IC_{50} values of 4x toward different cell lines.

Cell line	IC_{50} (μM)[a]
A375	2.57 ± 0.004
A549	2.98 ± 0.4
Hela	7.24 ± 0.14
MCF-7	8.66 ± 0.97
MIA Paca-2	11.93 ± 1.2
MDA-MB-231	13.96 ± 0.76
L02	25.01 ± 1.47

[a]Data are presented as the mean values ± SD from at least three independent experiments.

Statistical analysis

All assays, with established cell lines were repeated three times. All data are expressed as mean ± SD. The statistical analysis was performed by the Student's t-test, and if appropriate, by a one-way ANOVA test, using the statistical software GraphPad Prism 8.

XVIII. Supplementary References

[1] J. Liu; Y. Liu, *Org. Lett*. **2012**, *14*, 4742-4745.

[2] Y. Aota, Y. Maeda, T. Kano, K. Maruoka, *Chem. Eur. J.* **2019**, *25*, 15755-15758.

[3] L. Ye, W. He, L. Zhang, *Angew. Chem. Int. Ed.* **2011**, *50*, 3236-3239; *Angew. Chem.* **2011**, *123*, 3294 –3297.

[4] P. Shi, Y. Tu, D. Zhang, C. Wang, K.-N. Truong, K. Rissanen, C. Bolm, *Adv. Synth. Catal.* **2021**, *363*, 2552-2556.

[5] (a) G. Liu, T. Owens, T. Tang, J. Ellman, J. Org. Chem. 1998, 64, 1278-1284. (b) M. Robak, M. Herbage, J. Ellman, Chem. Rev. 2010, 110, 3600–3740. (c) G. Borg, D. Cogan, J. Ellman, Tetrahedron Lett. 1999, 40, 6709-6712. (d) J. Colyer, J. Tedrow, T. Soukup, M. Faul, J. Org. Chem. 2006, 71, 6859–6862. (e) C. Malapit, D. Caldwell, I. Luvaga, J. Reeves, I. Volchkov, N. Gonnella, Z. Han, C. Busacca, A. Howell, C. Senanayake, Angew. Chem. Int. Ed. 2017, 56, 6999-7002. (f) Y. Gui, S. Tian, Org. Lett. 2017, 19, 1554-1557.

[6] M. Zora, A. Kivrak, C. Yazici, *J. Org. Chem.* **2011**, *76*, 6726-6742.

[7] Z. Xia, J. Hu, Z. Shen, X. Wan, Q. Yao, Y. Lai, J.-M. Gao, W. Xie, *Org. Lett.* **2016**, 18, 80-83.

[8] J. Qian, Y. Liu, J. Zhu, B. Jiang, Z. Xu, *Org. Lett.* **2011**, *13*, 4220-4223.

[9] F. Li, J. Nie, L. Sun, Y. Zheng, J. A. Ma, *Angew. Chem., Int. Ed.* **2013**, *52*, 6255-6258; *Angew. Chem.* **2013**, *125*, 6375-6378.

[10] C. A. Lipinski, *Drug Discov. Today* **2004**, *1*, 337-341.

[11] (a) W. P. Walters, *Expert Opin. Drug Dis*. **2012**, *7*, 99-107; (b) R. Roskoski, *Pharmacol. Res.* **2020**, *152*, 104609.

[12] (a) D. Stumpfe, J. R. Bajorath, *J. Med. Chem.* **2012**, *55*, 2932-2942; (b) D. Stumpfe, H. Hu, J. R. Bajorath, *ACS Omega* **2019**, *4*, 14360-14368.
