# Supplementary figures and images for: Organocatalytic atroposelective construction of axially chiral N, N- and N, S-1,2-azoles through novel ring formation approach
Source: Nat Commun. 2022 Apr 11;13:1933. doi: 10.1038/s41467-022-29557-1 (PMC9001698; doi:10.1038/s41467-022-29557-1)

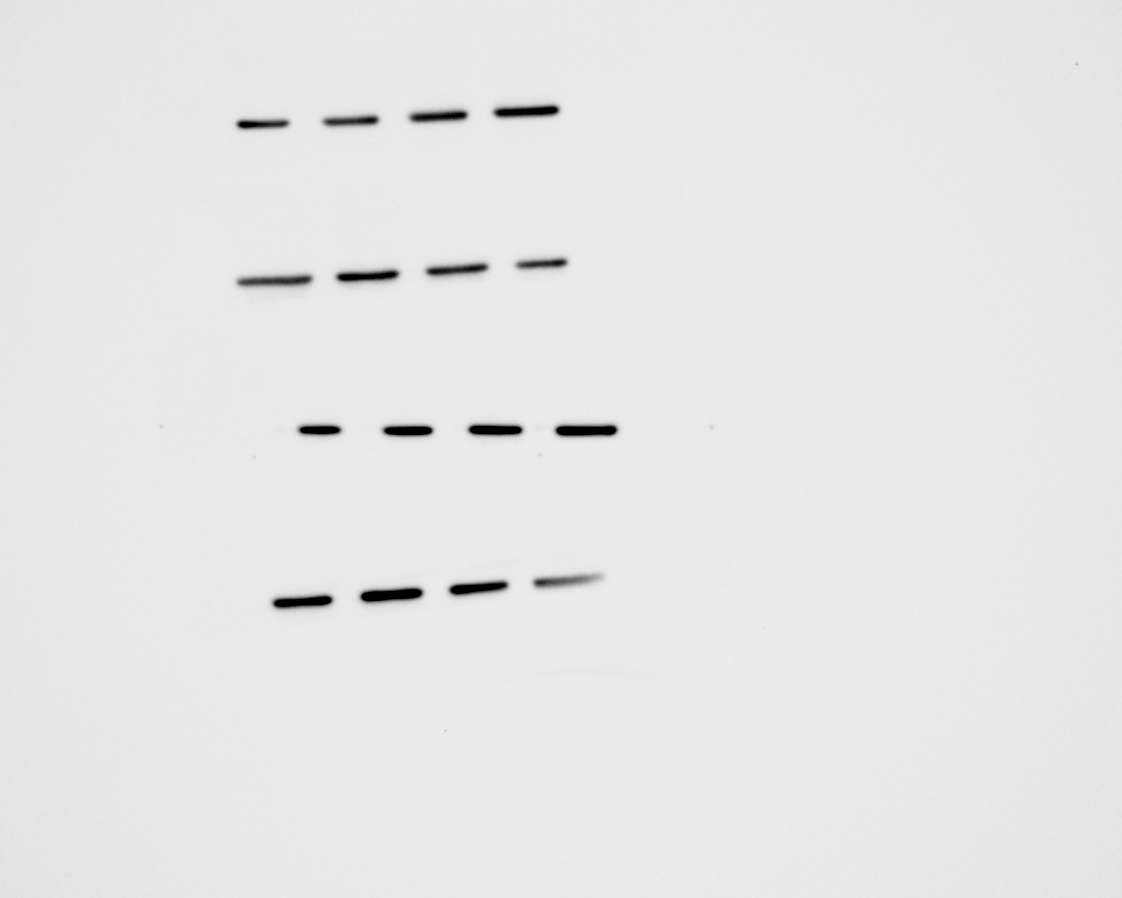

Supplement: Supplementary file 3 — Source Data [file 41467_2022_29557_MOESM3_ESM.zip › Source Data/western blot/Bax and Bcl-2 (stripe 1 and 2).tif]

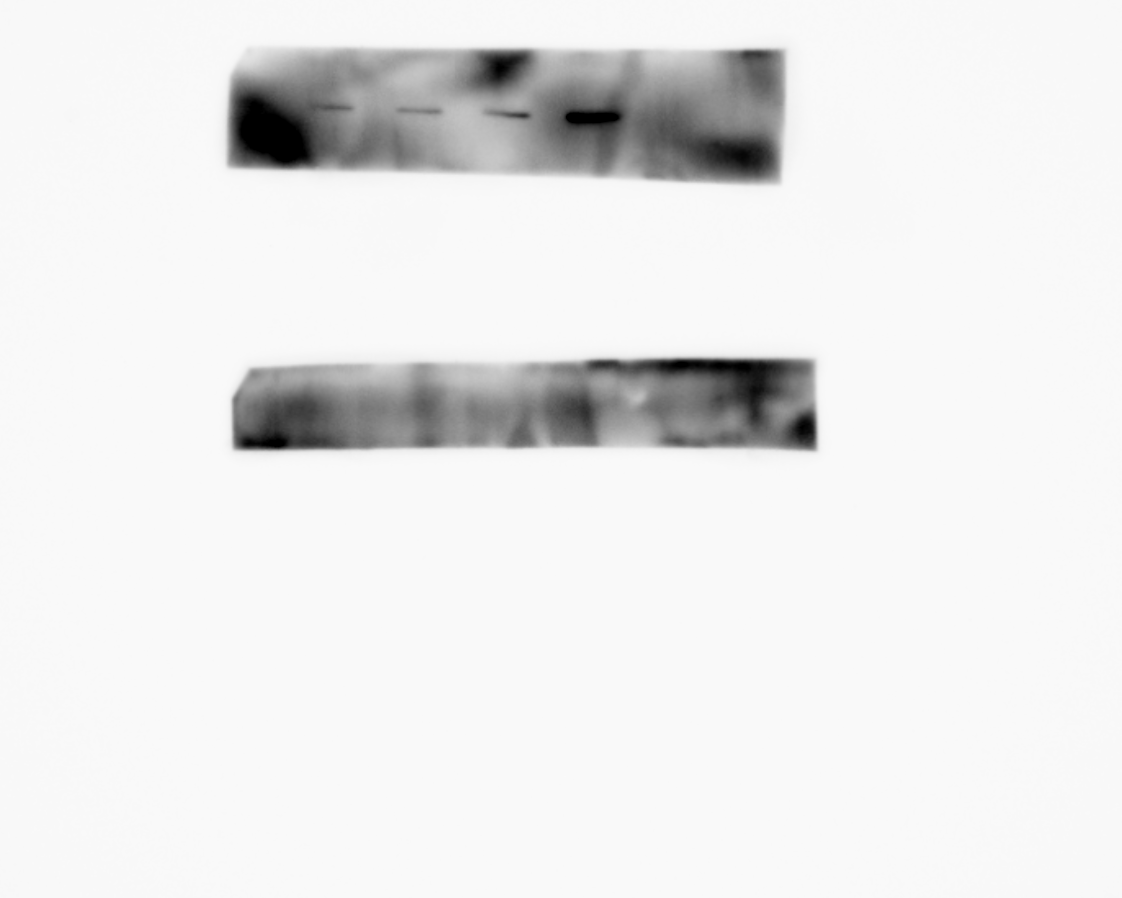

Supplement: Supplementary file 3 — Source Data [file 41467_2022_29557_MOESM3_ESM.zip › Source Data/western blot/Cleaved PARP ú¿stripe 1ú⌐.tif]

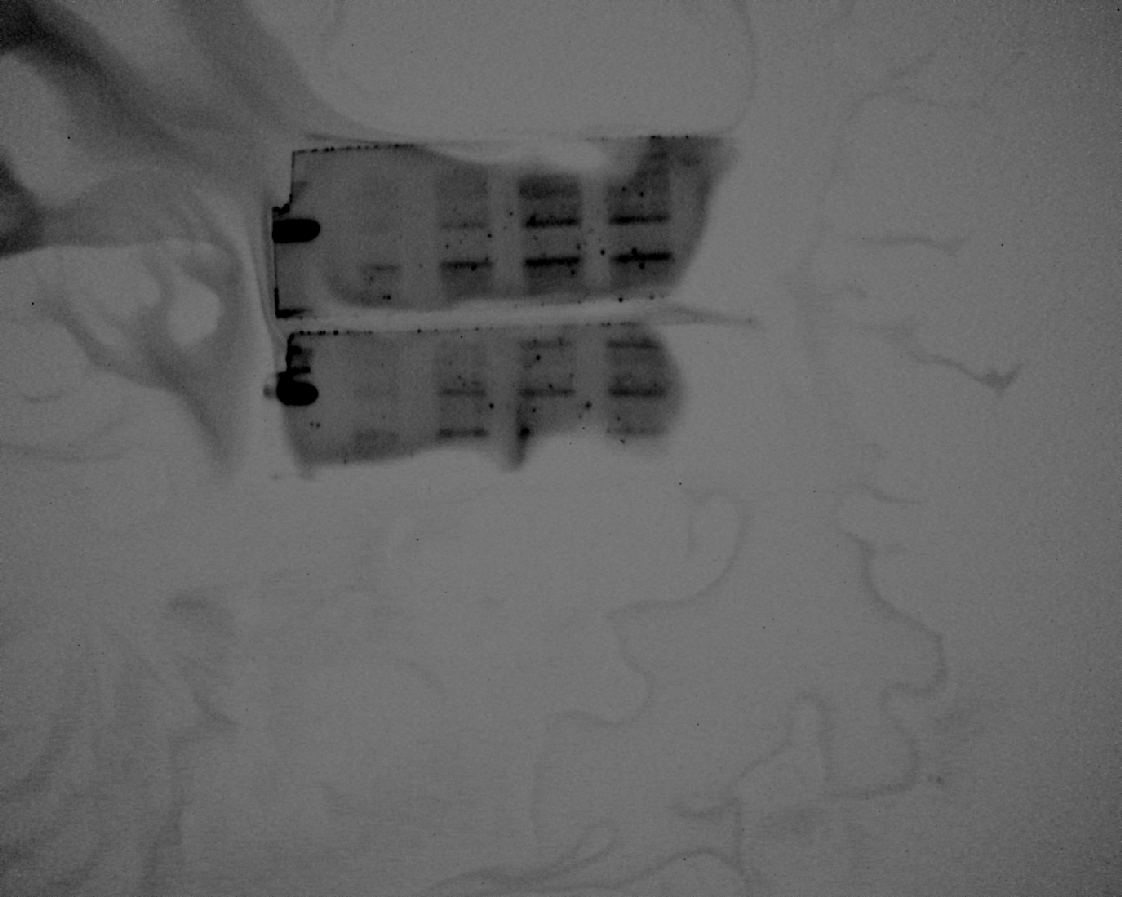

Supplement: Supplementary file 3 — Source Data [file 41467_2022_29557_MOESM3_ESM.zip › Source Data/western blot/Cleaved caspase 3 and Cleaved caspase 9.tif]

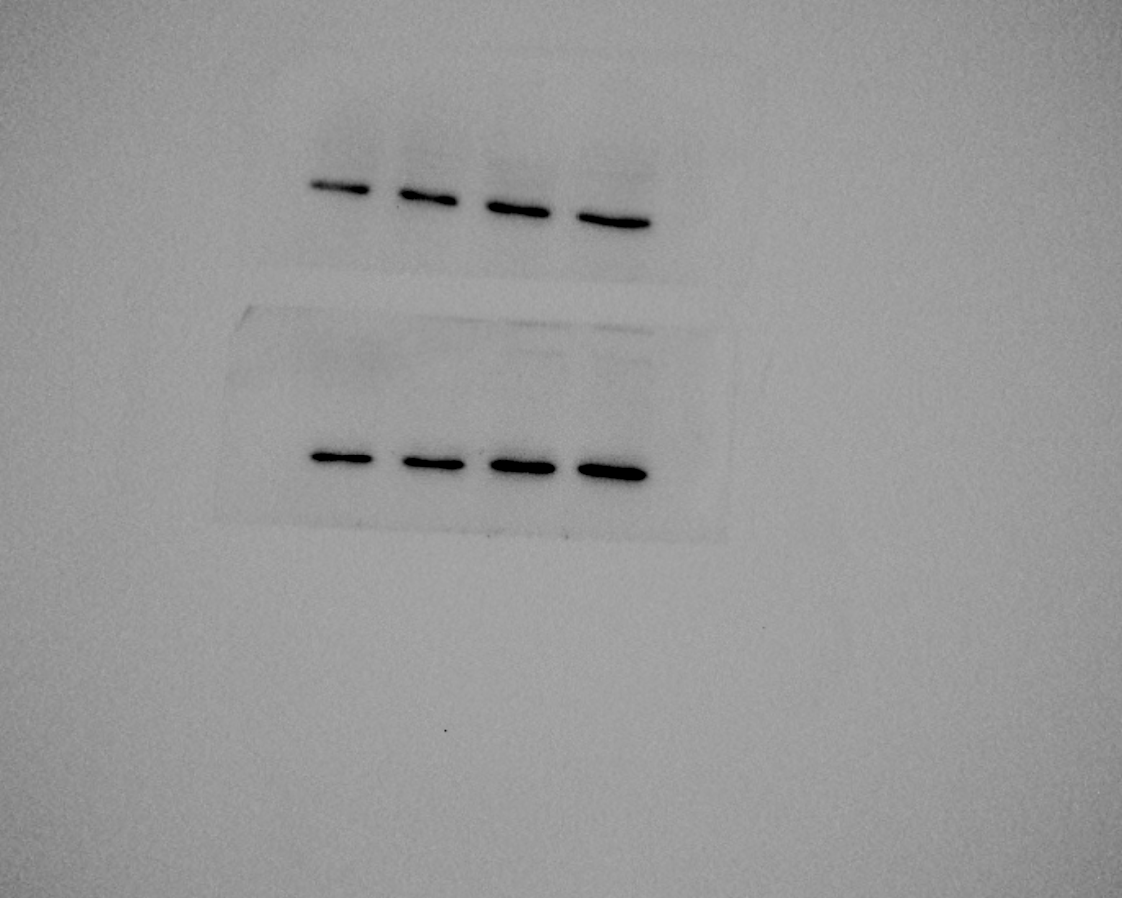

Supplement: Supplementary file 3 — Source Data [file 41467_2022_29557_MOESM3_ESM.zip › Source Data/western blot/Cyt-c and Bcl-xL.tif]

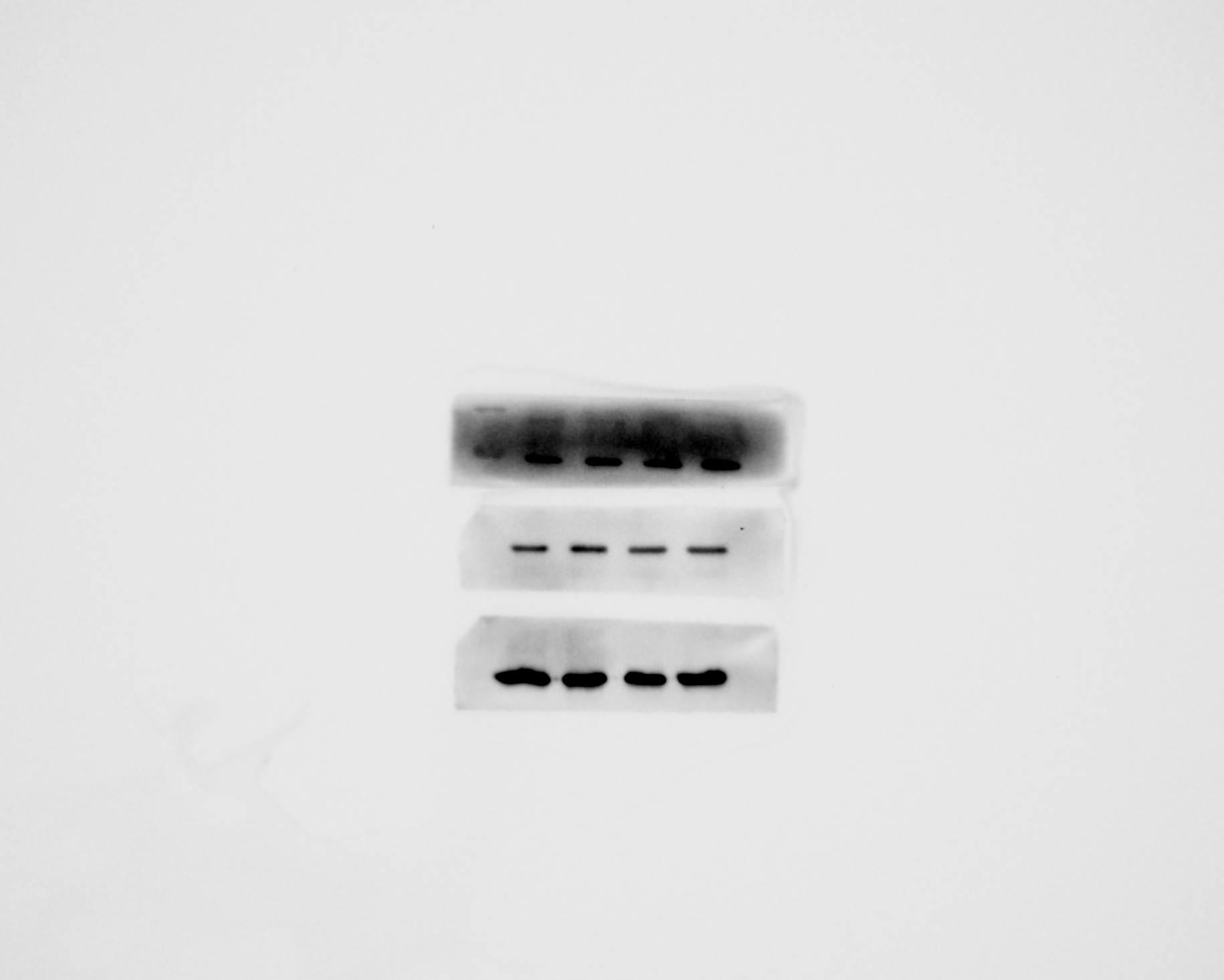

Supplement: Supplementary file 3 — Source Data [file 41467_2022_29557_MOESM3_ESM.zip › Source Data/western blot/GAPDH (stripe 2).tif]
